# Correction: Prognostic factors for severity and mortality in patients infected with COVID-19: A systematic review

**DOI:** 10.1371/journal.pone.0269291

**Published:** 2022-05-26

**Authors:** Ariel Izcovich, Martín Alberto Ragusa, Fernando Tortosa, María Andrea Lavena Marzio, Camila Agnoletti, Agustín Bengolea, Agustina Ceirano, Federico Espinosa, Ezequiel Saavedra, Verónica Sanguine, Alfredo Tassara, Candelaria Cid, Hugo Norberto Catalano, Arnav Agarwal, Farid Foroutan, Gabriel Rada

After this article [1] was published, concerns were raised that one of the articles included in the meta-analysis [2] was retracted [3] before the *PLOS ONE* article’s submission date, and was published online on 1^st^ May 2020, after the date reported for the end of the literature search in the Methods section of [1].

The authors clarify that the retracted article [2] was identified during a cross-reference search which was performed after the primary literature search on 28^th^ April 2020, but before [2] was retracted. The cross-reference literature search was described as a component of the original search strategy [1], and the authors provide the following additional information on how it was performed. All studies identified with the primary search strategy meeting criteria for inclusion were used as substrate for the cross-reference search. The Google Scholar “Cited by” function was used to identify publications that cited the corresponding study. All identified publications were considered for inclusion in the review.

The authors apologize that the article’s retracted status was not identified prior to the publication of [1]. As studies should not rely upon retracted research, the authors have undertaken re-analysis of the affected results excluding data from the retracted article [2].

Specifically, data from the retracted article [2] were removed from the assessment of the impact of the following variables on mortality outcome: age, gender, smoking, COPD, cardiovascular disease, immunocompromise, diabetes, arterial hypertension, dyslipidemia, and cardiac arrhythmia. The updated analyses are presented with this notice in S1 Appendix, and comparison of the original and updated results are presented in S1 File.

The results of the re-analysis revealed that after excluding the retracted study [2] the estimates of effects were no longer statistically significant for two variables (“dyslipidemia” and “immunocompromise”), and the certainty of the evidence was downgraded for two variables (“smoking” and “immunocompromise”). However, the study’s other results and overall conclusions were not significantly altered. See S1 Appendix and S1 File for details.

The re-analyses were assessed by a reviewer who advised that the findings in [1] are supported following the removal of the data from the retracted article [2], and that statistical methodology used in the re-analysis was appropriate.

We also provide the following addendum to the second paragraph of the “Strengths and limitations of the study” Discussion section:

Of the 207 studies included in the meta-analysis, 106 were available only as preprints at the time of the study, i.e. they had not been peer-reviewed or published. The conclusions of this study should be considered in the context of this study design limitation, which was reflective of the early stage of the COVID-19 pandemic at the time the literature searches were performed. The authors have not undertaken a full assessment of the current publication status of the included preprints, and furthermore did not conduct subgroup analyses based on publication status. However recent studies examining cohorts of COVID-19 preprints vs. articles suggested that conclusions were not widely changed following peer review [4,5].

## Supporting information

S1 Appendix Mortality forest plots. The Forest plots for all assessed candidate variables, including the updated Forest plots for age, gender, smoking, COPD, cardiovascular disease, immunocompromise, diabetes, arterial hypertension, dyslipidemia and cardiac arrhythmia. An additional table is included to identify the original reference for the study labels used in the Forest plots.
(DOCX)

S1 File Comparison of findings from S3 Table in [1] and re-analysis with data from [2] removed. Comparison of published findings in S3 Table and updated results for variables age, gender, smoking, COPD, cardiovascular disease, immunocompromise, diabetes, arterial hypertension, dyslipidemia and cardiac arrhythmia.
(DOCX)
