# Supplementary material for: Prognostic factors for severity and mortality in patients infected with COVID-19: A systematic review
Source: PLoS One. 2020 Nov 17;15(11):e0241955. doi: 10.1371/journal.pone.0241955 (PMC7671522; doi:10.1371/journal.pone.0241955)
Supplement: S1 Table — This table presents detailed information on individual included studies. (PDF) [file pone.0241955.s001.pdf]

Supplementary table 1. Characteristics of included studies

Ref. nr	Author/s, year	Center (country)	Number of participants	Population included			Candidate prognostic factors assessed					Outcomes			Adjusted analysis
				**Non-severe, %**	**Severe, %**	**Critical, %**	**Social-Demographic**	**Comorbidities**	**Clinical findings**	**Laboratory findings**	**Image findings**	**Mortality**	**Severe COVID-19 disease**	**Severe COVID-19 definition**	
30	Argenziano M, 2020	New York-Presbyterian/Columbia University Irving Medical Center (USA)	1000	NR	NR	NR	Yes	Yes	Yes	No	No	No	Yes	ICU	No
31	Auld S, 2020	Three Emory Healthcare acute-care hospitals in Atlanta (USA)	181	0	0	100	Yes	Yes	Yes	Yes	No	Yes	No	NA	No
32	Bai T, 2020	Jinyintan hospitals (China)	127	NR	NR	NR	Yes	Yes	Yes	Yes	No	Yes	No	NA	Yes
21	Bai X, 2020	Wuhan Pulmonary Hospital (China)	133	100	0	0	Yes	Yes	Yes	Yes	Yes	Yes	Yes	Severe (>= 30 breathings OR Sat <=93% OR OI < 300 OR ICU)	Yes
33	Benelli G, 2020	Hospital of Crema (Italy)	411	NR	NR	NR	Yes	Yes	Yes	Yes	Yes	Yes	No	NA	No

24	Bi Q, 2020	Shenzhen Third People's Hospital (China)	420	77.86	17.62	4.52	Yes	Yes	Yes	Yes	Yes	No	Yes	Progression in severity status	Yes
34	Cai Q, 2020	Third People's Hospital of Shenzhen (China)	298	80.50	19.50	0	Yes	Yes	Yes	Yes	Yes	Yes	Yes	Severe (>= 30 breathings OR OI <=250 OR IMV OR shock)	Yes
35	Cao M, 2020	Shanghai Public Health Clinical Centre (China)	198	90.40	9.60	0	Yes	No	Yes	Yes	Yes	No	Yes	Severe (>= 30 breathings OR Sat <93% OR OI <=300)	No
36	Cao W, 2020	Xiangyang No 1 Hospital, The Fourth affiliated Hospital of Hubei Medicine University (China)	128	83	17	0	Yes	Yes	Yes	Yes	Yes	No	Yes	Severe (> 30 breathings OR Sat <90%)	No
37	Chao C, 2020	Ningbo First Hospital and Jingzhou Central Hospital (China)	58	100	0	0	Yes	Yes	Yes	Yes	No	No	Yes	Progression in severity status	Yes
38	Chen G, 2020	Tongji Hospital (China)	21	47.62	52.38	0	Yes	Yes	Yes	Yes	Yes	Yes	Yes	Severe (>= 30 breathings OR Sat <=93%)	No

39	Chen J, Pan h, 2020	First Affiliated Hospital of Wenzhou Medical University (China)	46	NR	NR	NR	Yes	Yes	Yes	Yes	Yes	No	Yes	ICU	No
40	Chen J, Qi T, 2020	Shanghai Public Health Clinical Center (China)	249	NR	NR	NR	Yes	Yes	Yes	Yes	Yes	Yes	Yes	ICU	Yes
41	Chen L, 2020	Tongji Hospital (China)	29	52	31	17	Yes	Yes	Yes	Yes	Yes	No	Yes	Severe (>= 30 breathings OR Sat <=90% OR OI <=300)	No
42	Chen M, 2020	Fifth Affiliated Hospital of Sun Yat-Sen University (China)	97	73.20	26.80	0	Yes	Yes	Yes	Yes	Yes	No	Yes	Severe (OI >=150 AND <=300)	No
43	Chen M, Yongzhen F, 2020	Seventh Hospital of Wuhan City (China)	123	NR	NR	NR	Yes	Yes	Yes	Yes	No	Yes	No	NA	Yes
44	Chen R, 2020	575 hospitals (China)	1590	NR	NR	NR	Yes	Yes	Yes	Yes	No	Yes	No	NA	Yes
45	Chen TL, 2020	Zhongnan Hospital (China)	55	NR	NR	NR	Yes	Yes	Yes	Yes	Yes	Yes	No	Severe (> 30 breathings OR Sat <93%)	Yes
46	Chen W, 2020	Yongwu Hospital (China)	74	88	12	0	Yes	Yes	Yes	Yes	Yes	Yes	Yes	Severe (>= 30 breathings OR OI <=250 OR IMV OR shock)	No

47	Chen X, Liu Z, 2020	Tongji Hospital (China)	256	NR	NR	NR	Yes	Yes	Yes	Yes	No	Yes	No	NA	No
48	Chen X, Zhang Y 2020	Guangzhou Eighth People´s Hospital (China)	284	84	14	2	Yes	Yes	Yes	Yes	No	Yes	Yes	Severe (> 30 breathings OR Sat <90%)	No
49	Chen X, Zhao B 2020	General Hospital of Central Theater Command, PLA, a designated hospital in Wuhan (China)	48	43.75	20.83	35.42	Yes	Yes	Yes	Yes	Yes	Yes	Yes	Severe (SOB, >30 breathings, Sat <93, OI<300) Critical (IMV, shock, ICU)	No
50	Chen X, Zheng F, 2020	Hospital of Changshay Loudi Central Hospital (China)	291	82.82	17.18	0	Yes	Yes	Yes	Yes	Yes	Yes	Yes	Severe (>= 30 breathings OR Sat <=93% OR OI <=300)	NO
51	Chen Y, 2020	Multicenter- Fourth Clinical Medical College of Hebei Medical University (China)	37	0	59	41	Yes	Yes	Yes	Yes	Yes	No	Yes	Critical (Respiratory failure or shock, or other organ failure that required ICU)	No
52	Cheng Y, 2020	Tongji Hospital (China)	710	NR	NR	NR	Yes	No	Yes	Yes	No	Yes	No	NA	No
53	Chu J, 2020	Tongji hospital (China)	54	20.37	74.07	5.56	Yes	No	Yes	No	Yes	Yes	No	NA	No
54	Colaneri M, 2020	Policlinico San Matteo (Italia)	44	61.36	38.64	0	Yes	Yes	Yes	Yes	Yes	Yes	Yes	Severe (Sat <90%)	Yes

55	Colombi D, 2020	Guglielmo di Salicilato Hospital (Italia)	236	54.24	45.76	0	Yes	Yes	Yes	Yes	Yes	No	Yes	ICU	Yes
56	Cummings M, 2020	2 New York Presbyterian Hospitals (USA)	257	0	0	100	Yes	Yes	Yes	Yes	Yes	Yes	No	NA	Yes
57	Docherty AB, 2020	166 hospitals (UK)	6628	NR	NR	NR	Yes	Yes	Yes	No	No	Yes	No	NA	Yes
23	Dong J, 2020	Fifth Medical Center of Chinese PLA General Hospital (China)	208	NR	NR	NR	No	Yes	Yes	Yes	No	No	No	Severe (>= 30 breathings OR Sat <=93% OR OI <=300 OR IMV)	Yes
58	Dreher M, 2020	University Hospital Aachen (Germany)	50	0	52	48	Yes	Yes	Yes	No	Yes	Yes	Yes	ARDS	No
59	Du RH, 2020	Wuhan Pulmonary Hospital (China)	179	100	0	0	Yes	Yes	Yes	Yes	No	Yes	No	NA	No
60	Duan Q, 2020	Wuhan Pulmonary Hospital (China)	116	100	0	0	Yes	Yes	Yes	Yes	Yes	No	Yes	Critical (> 30 breathings OR ARDS OR Sat <93%)	No
61	Fan J, 2020	Zhongnan Hospital (China)	21	NR	NR	NR	Yes	Yes	Yes	Yes	No	Yes	No	NA	No

62	Fan T, 2020	Renmin Hospital (China)	317	51.73	11.99	36.28	Yes	Yes	Yes	Yes	No	No	Yes	Severe (> 30 breathings OR Sat <90%)	Yes
63	Fang L, 2020	General Hospital of Central Theater Command of People’s Liberation Army (China)	140	76.50	23.50	0	Yes	Yes	Yes	Yes	No	No	Yes	Severe (>= 30 breathings OR Sat <=93% OR OI <= 300 OR lung images with progression > 50% in 24-48 hs)	Yes
64	Fei J, 2020	Union Hospital of Huazhong University of Science and Technology (China)	192	NR	NR	NR	No	No	No	Yes	No	Yes	No	Severe (OI <300)	Yes
65	Feng Y, 2020	Jinyintan Hospital in Wuhan, Shanghai Public Health Clinical Center in Shanghai and Tongling People's Hospital (China)	476	74	11	15	Yes	Yes	Yes	Yes	Yes	Yes	Yes	Critical (IMV OR shock OR other organ failure with ICU requirement)	No

66	Feng Z, 2020	Third Xiangya Hospital, Changsha Public Health Treatment Center and Second People’s Hospital of Hunan (China)	141	100	0	0	Yes	Yes	Yes	Yes	Yes	No	Yes	Severe (clinical progression)	Yes
67	Fu L, Fei J, Xiang H, 2020	Union Hospital of Huazhong University of Science and Technology (China)	200	44.10	29.70	26.20	Yes	Yes	Yes	Yes	No	Yes	No	NA	Yes
68	Fu L, Fei J, Xu S, 2020	Union Hospital of Huazhong University (China)	350	60.30	25.10	14.60	Yes	Yes	Yes	Yes	No	Yes	No	NA	Yes
69	Gao L, 2020	Hubei General Hospital (China)	54	0	100	0	Yes	Yes	No	Yes	No	Yes	No	Severe (>= 30 breathings OR Sat <=93% OR OI <= 300)	Yes
70	Gao Y, 2020	Fuyang Second People's Hospital (China)	43	100	0	0	Yes	Yes	Yes	Yes	No	No	Yes	Severe (>= 30 breathings OR Sat <=93% OR OI <= 300)	No
27	Gong J, 2020	Three Centers in Guangzhou and Wuhan (China)	372	NR	NR	NR	Yes	Yes	Yes	Yes	No	No	Yes	Severe (>= 30 breathings OR Sat <=93% OR OI <= 300)	No

71	Grasselli G, 2020	Lombardy ICU Network (Italy)	1591	0	0	100	Yes	Yes	Yes	Yes	Yes	Yes	No	NA	No
72	Gu T, 2020	31 province-level regions (China)	275	NR	NR	NR	Yes	Yes	No	No	No	Yes	No	NA	Yes
73	Guan W, Liang W, 2020	575 hospitals (China)	1590	NR	NR	NR	Yes	Yes	Yes	No	Yes	Yes	No	NA	No
74	Guan W, Ni Z, 2020	National Health Commission of China (China)	1099	84.30	15.70	0	Yes	Yes	Yes	Yes	Yes	No	Yes	Severe (shock OR respiratory failure + IMV OR > 30 breathings OR OI <250)	No
75	Guo T, 2020	Seventh Hospital (China)	187	NR	NR	NR	No	No	No	Yes	No	Yes	No	NA	No
76	Guo TM, 2020	Union Dongxihu Hospital (China)	118	NR	NR	NR	Yes	Yes	Yes	Yes	Yes	Yes	No	NA	Yes
77	Guo W, 2020	Wuhan Union Hospital (China)	174	NR	NR	NR	No	No	No	No	No	Yes	No	NA	No
78	Han H, 2020	Renmin Hospital (China)	273	72.50	22	5.50	Yes	No	No	Yes	No	Yes	Yes	Critical (IMV OR shock OR other organ failure with ICU requirement)	No

79	Han M, 2020	Second People's Hospital (China)	154	79.22	20.78	0	Yes	Yes	Yes	Yes	No	No	Yes	Severe (>= 30 breathings OR Sat <=93% OR OI <= 300)	No
80	Han Y, 2020	Renmin Hospital of Wuhan University (China)	47	49	51	0	Yes	Yes	Yes	Yes	Yes	No	Yes	Severe (shock OR respiratory failure + IMV OR >30 breathings OR OI <250)	Yes
81	He W, 2020	3 hospitals (China)	31	65	19	16	Yes	Yes	Yes	Yes	Yes	Yes	No	NA	No
82	He XW, 2020	Tongji Hospital (China)	56	0	55	45	Yes	Yes	Yes	Yes	Yes	Yes	No	NA	No
83	Herold T, 2020	University Hospital (Germany)	40	NR	NR	NR	Yes	Yes	Yes	No	Yes	No	Yes	IMV	No
84	Hu B, 2020	Zhongnan Hospital of Wuhan University and Wuhan Pulmonary Hospital (China)	36	0	0	100	Yes	Yes	Yes	Yes	No	Yes	No	NA	Yes
85	Hu L, 2020	Tianyou Hospital (China)	323	46.75	45.20	8.05	Yes	Yes	Yes	Yes	Yes	No	Yes	Critical (Severe ARDS OR Shock OR IMV)	Yes

86	Hu X, 2020	Six hospitals of Chongqing (China)	164	83	17	0	No	No	Yes	Yes	Yes	No	Yes	Severe (> 30 breathings OR Sat <90%)	No
87	Hu Z, 2020	Tongji Hospital (China)	95	NR	NR	NR	Yes	Yes	Yes	Yes	Yes	Yes	No	NA	No
88	Huang C, 2020	Jin Yin-tan Hospital (China)	41	31.70	68.30	0	Yes	Yes	Yes	Yes	Yes	No	Yes	ICU	No
89	Huang H, 2020	Guangzhou Eighth People's Hospital (China)	125	74	26	0	Yes	Yes	Yes	Yes	No	No	Yes	Severe (> 30 breathings OR Sat <90%)	Yes
90	Huang M, 2020	12 hospitals (China)	60	0	100	0	Yes	Yes	Yes	Yes	Yes	Yes	Yes	Progression in severity status	No
91	Jiang X, 2020	Wuxi Fifth People’s Hospital (China)	55	85.50	14.50	0	Yes	Yes	Yes	Yes	Yes	No	Yes	Severe (> 30 breathings OR Sat <90%)	No
92	Jin JM, 2020	Wuhan Union Hospital (China)	43	NR	NR	NR	Yes	Yes	Yes	Yes	No	Yes	No	NA	No
93	Jin X, 2020	Multicenter in Zhejiang province (China)	651	90	10	0	Yes	Yes	Yes	Yes	Yes	No	Yes	Severe (> 30 breathings OR Sat <90%)	No
94	Kalligeros M, 2020	3 hospitals (USA)	103	57	43	0	Yes	Yes	No	No	No	No	Yes	ICU	Yes

95	Kuang Y, 2020	Taizhou Hospital Enze district, Wenling First People's Hospital, Sanmen People's Hospital and Tiantai People's Hospital (China)	143	76.92	23.08	0	Yes	No	Yes	Yes	Yes	Yes	Yes	Severe (> 30 breathings OR Sat <90%)	No
96	Lee H, 2020	Seoul National University College of Medicine (Korea)	8266	NR	NR	NR	Yes	Yes	No	No	No	Yes	No	NA	Yes
97	Lei L, 2020	Chongqing University Three Gorges Hospital (China)	51	86.30	7.80	5.90	Yes	Yes	Yes	Yes	Yes	No	Yes	Severe (> 30 breathings OR Sat <90%)	No
98	Lei S. 2020	Renmin Hospital, Zhongnan Hospital, Tongji Hospital and Central Hospital (China)	34	56	44	0	Yes	Yes	Yes	Yes	No	No	Yes	ICU	No
99	Li H. 2020	Tianyou Hospital (China)	132	46	42	12	Yes	No	Yes	Yes	Yes	Yes	Yes	Severe (> 30 breathings OR Sat <90%)	No

100	Li J, Long X, 2020	Wuhan Red Cross Hospital (China)	161	NR	NR	NR	Yes	Yes	Yes	Yes	Yes	Yes	No	NA	No
101	Li J, Meng L, 2020	Central Hospital of Wuhan (China)	134	34	22	44	Yes	Yes	Yes	Yes	Yees	Yes	Yes	Critical (Severe ARDS OR Shock OR IMV)	Yes
102	Li J, Wang X, 2020	Central Hospital of Wuhan (China)	362	52	48	0	Yes	Yes	No	No	No	Yes	No	Severe (> 30 breathings OR Sat <90%)	No
103	Li K, 2020	Affiliated Hospital of Chongqing Medical University (China)	83	69.90	30.10	0	Yes	Yes	Yes	Yes	Yes	No	Yes	Critical (Severe ARDS OR Shock OR IMV)	No
25	Li X, 2020	Sino-French New City Branch of Tongji Hospital (China)	548	51	49	0	Yes	Yes	Yes	Yes	Yes	Yes	Yes	Severe (> 30 breathings OR Sat <90%)	Yes
104	Li Y, 2020	Tongji Hospital (China)	77	76.63	5.19	18.18	Yes	Yes	Yes	Yes	Yes	No	No	NA	Yes
105	Li YK, 2020	Tongji Hospital (China)	25	64	36	0	Yes	Yes	Yes	Yes	No	Yes	Yes	Severe (> 30 breathings OR Sat <90%)	No
106	Lian J, 2020	Health Commission of Zhejiang province (China)	788	90.10	7.74	2.16	No	No	No	No	No	No	Yes	Severe (> 30 breathings OR Sat <90%)	No

107	Liang W, 2020	575 hospitals (China)	1590	100	0	0	No	No	No	No	No	No	Yes	Critical (Severe ARDS OR Shock OR IMV)	No
108	Liao X, 2020	Sichuan Provincial Department of Health (China)	81	0	100	0	No	No	No	No	No	No	Yes	IMV	No
109	Liu F, 2020	Xixi hospital (China)	10	70	30	0	Yes	Yes	Yes	Yes	Yes	No	Yes	Progression in severity status	No
110	Liu J, 2020	Jinyintan Hospital (China)	122	35	65	0	No	Yes	Yes	Yes	Yes	Yes	Yes	Severe (> 30 breathings OR Sat <90%)	No
111	Liu J, Li S, 2020	Wuhan Union Hospital and Tongji Medical College of Huazhong University of Science and Technology (China)	40	67.50	32.50	0	Yes	Yes	Yes	Yes	No	Yes	Yes	Severe (> 30 breathings OR Sat <90%)	No
112	Liu J, Liu Y, 2020	Beijing Ditan Hospital (China)	61	72	28	0	Yes	Yes	Yes	Yes	Yes	No	Yes	Critical (Severe ARDS OR Shock OR IMV)	No
113	Liu KC, 2020	6 Hospitals in Anhui (China)	73	67.12	28.77	4.11	Yes	No	Yes	No	Yes	No	Yes	Critical (Severe ARDS OR Shock OR IMV)	No

114	Liu R, 2020	Central Hospital of Wuhan (China)	41	NR	NR	NR	Yes	Yes	Yes	Yes	Yes	No	Yes	IMV	Yes
115	Liu T, 2020	Union Hospital (China)	80	13.75	86.25	0	Yes	Yes	Yes	Yes	Yes	No	Yes	Critical (Severe ARDS OR Shock OR IMV)	No
116	Liu W, 2020	1 tertiary hospital in Wuhan (China)	78	89.74	10.26	0	No	No	No	No	No	No	Yes	Progression in severity status	Yes
117	Liu Y, 2020	Shangai Pulmonary Hospital (China)	221	83.70	12.20	4.10	No	Yes	Yes	Yes	Yes	No	Yes	Severe (> 30 breathings OR Sat <90%)	No
118	Liu Y, Bi L, 2020	Shenyang Chest Hospital (China)	36	75	25	0	Yes	Yes	Yes	Yes	No	No	Yes	Severe (> 30 breathings OR Sat <90%)	No
119	Liu Y, Du X, 2020	Hospital of Wuhan University (China)	245	97	3	0	Yes	Yes	Yes	Yes	Yes	Yes	No	NA	Yes
120	Liu Y, Sun W, Guo Y, 2020	Central Hospital of Wuhan (China)	383	NR	NR	NR	No	No	No	No	No	Yes	No	NA	Yes
121	Liu Y, Sun W, Li J, 2020	Central Hospital of Wuhan (China)	109	51.40	48.60	0	Yes	Yes	Yes	Yes	Yes	No	Yes	ARDS	No

122	Liu Y, Yang Y, 2020	Shenzen Third People's Hospital (China)	12	NR	NR	NR	Yes	Yes	Yes	Yes	Yes	No	Yes	Critical (Severe ARDS OR Shock OR IMV)	No
123	Lu J, 2020	Wuhan Hankou Hospital (China)	438	77	23	0	Yes	Yes	Yes	Yes	Yes	Yes	Yes	Severe (SOB + >30 breathings OR Sat <93 OR OI <300)	Yes
124	Luo XM, 2020	Renmin Hospital (China)	298	47.30	52.70	0	Yes	Yes	Yes	Yes	No	Yes	No	NA	Yes
125	Luo XM, 2020	Renmin Hospital (China)	403	49.10	50.90	0	Yes	Yes	Yes	Yes	NR	Yes	Yes	Severe (> 30 breathings OR Sat <90%)	No
126	Lv X, 2020	Central Hospital of Wuhan City and other hospitals in Hunan (China)	208	73.56	26.44	0	Yes	Yes	Yes	Yes	Yes	Yes	Yes	Severe (> 30 breathings OR Sat <90%)	No
127	Ma J, 2020	Renmin Hospital of Wuhan University (China)	37	46	54	0	Yes	Yes	Yes	Yes	No	No	Yes	Progression in severity status	No
128	Ma K, 2020	Yongchuan Hospital (China)	84	76	24	0	Yes	Yes	Yes	Yes	Yes	No	Yes	Severe (> 30 breathings OR Sat <90%)	Yes
129	Ma Y, 2020	43 hospitals in 10 provinces (China)	635	90	10	0	Yes	Yes	Yes	Yes	Yes	No	Yes	Severe (> 30 breathings OR Sat <90%)	No

130	Mehra MR, 2020	169 hospitals (Asia, Europe and North America)	8910	NR	NR	NR	Yes	Yes	No	No	No	Yes	No	NA	Yes
131	Mo P, 2020	Zhongnan Hospital (China)	155	40.60	35.50	23.90	Yes	Yes	Yes	Yes	Yes	No	Yes	Critical (Severe ARDS OR Shock OR IMV)	Yes
132	Niu S, 2020	Beijing Emergency Medical Center, (China)	141	69	31	0	Yes	Yes	Yes	No	No	Yes	Yes	Severe (> 30 breathings OR Sat <90%)	No
133	Pan L, 2020	Wuhan Hanan Hospital, Wuhan Union Hospital, and Huanggang Central Hospital (China)	103	65.70	0	34.30	Yes	Yes	Yes	Yes	No	No	Yes	Critical (Severe ARDS OR Shock OR IMV)	No
134	Paranjpe I, 2020	MSHCS (USA)	1078	NR	NR	NR	Yes	Yes	Yes	Yes	No	Yes	No	ICU	No
135	Peng YD, 2020	Wuhan Union Hospital (China)	112	85.71	14.29	0	Yes	Yes	Yes	Yes	No	Yes	Yes	Severe (SOB, >30 breathings, Sat<93, OII<300) Critical IMV, shock, ICU)	No
136	Qi D, 2020	3 hospitals in Chongqing (China)	267	81.27	18.73	0	Yes	Yes	Yes	Yes	Yes	No	Yes	Severe (> 30 breathings OR Sat <90%)	No

137	Qi X, 2020	5 designated hospitals in Ankang, Lishui, Zhenjiang, Lanzhou, and Linxia (China)	31	NR	NR	NR	Yes	Yes	Yes	Yes	Yes	No	Yes	OTHER (Long term stay ->10 days)	No
138	Qin X, 2020	Shishou People's Hospital (China)	89	60.70	39.30	0	Yes	Yes	Yes	Yes	No	No	Yes	ICU	No
139	Qu R, 2020	Huizhou Municipal Central Hospital (China)	30	NR	NR	NR	Yes	Yes	Yes	Yes	Yes	No	Yes	Severe (SOB, >30 breathings, Sat <93, OI <300,) Critical (IMV, ICU)	No
140	Ran J, 2020	Tongji Hospital (China)	144	77.78	22.22	0	Yes	Yes	Yes	Yes	No	No	Yes	ICU	Yes
141	Rentsch CT, 2020	Veteran Affairs (USA)	419	NR	NR	NR	Yes	Yes	Yes	Yes	No	No	Yes	ICU	Yes
142	Rossi PG, 2020	Reggio Emilia, (Italy)	2242	NR	NR	NR	Yes	Yes	No	No	No	Yes	No	NA	Yes
143	Ruan Q, 2020	Jin Yin-tan Hospital and Tongji Hospital (China)	150	NR	NR	NR	NR	NR	NR	NR	NR	Yes	No	NA	No
144	Sakar J, 2020	CSIR-Indian Institute of Chemical Biology (India)	222	NR	NR	NR	No	No	No	No	No	Yes	No	NA	No

145	Shi H, 2020	The First and Second affiliated Hospitals of Wenzhou Medical University (China)	148	80.40	19.60	0	Yes	Yes	Yes	Yes	Yes	Yes	Yes	Severe (> 30 breathings OR Sat <90%)	Yes
146	Shi S, 2020	Renmin Hospital (China)	416	NR	NR	NR	Yes	Yes	Yes	Yes	No	Yes	No	NA	Yes
147	Shi W, 2020	Shanghai Public Health Clinical Center (China)	196	77	14	9	Yes	Yes	Yes	Yes	Yes	No	Yes	Severe (> 30 breathings OR Sat <90%)	No
148	Shi Y, 2020	Cohort from Zhejiang Province (China)	487	89.94	10.06	0	Yes	Yes	No	No	No	No	Yes	NA	Yes
149	Song C, 2020	First Affiliated Hospital, School of Medicine, Zhejiang University (China)	73	43	57	0	Yes	Yes	Yes	Yes	No	No	Yes	Severe (> 30 breathings OR Sat <90%)	No
150	Sun F, 2020	Zhongnan Hospital (China)	165	84.24	15.76	0	Yes	Yes	Yes	Yes	No	No	Yes	Severe (> 30 breathings OR Sat <90%)	No

151	Sun X, 2020	Peking Union Medical College Hospital (China)	69	0	0	100	Yes	Yes	Yes	Yes	No	Yes	No	NA	No
152	Sun Y, 2020	Hospitals in Beijing (China)	63	69.80	15.90	14.30	Yes	Yes	Yes	Yes	Yes	No	Yes	OTHER	Yes
153	Tabata S, 2020	Self-Defense Forces Central Hospital (Japan)	104	80.70	19.30	0	Yes	Yes	Yes	Yes	Yes	No	Yes	Severe (> 30 breathings OR Sat <90%)	Yes
154	Tan L, 2020	General Hospital of Central Theater Command (China)	30	NR	NR	NR	Yes	Yes	Yes	Yes	Yes	Yes	No	NA	No
155	Tang N, 2020	Tongji Hospital (China)	183	NR	NR	NR	Yes	NR	NR	Yes	NR	Yes	No	NA	No
156	Tian S, 2020	57 hospitals (China)	262	82.44	17.56	0	Yes	No	Yes	No	No	Yes	Yes	Severe (> 30 breathings OR Sat <90%)	No
157	Tu WJ, 2020	Zhongnan Hospital (China)	174	NR	NR	NR	Yes	Yes	Yes	Yes	No	Yes	No	NA	No
158	Wan S, 2020	Three Gorges Hospital (China)	123	83	17	0	Yes	Yes	Yes	Yes	Yes	Yes	Yes	Severe (> 30 breathings OR Sat <90%)	No
159	Wang D, Hu B, 2020	Zhongnan Hospital (China)	138	73.91	0	26.09	Yes	Yes	Yes	Yes	Yes	No	Yes	ICU	No

160	Wang D, Yin Y, 2020	Wuhan and Xishui Hospital (China)	107	NR	NR	NR	Yes	Yes	Yes	Yes	Yes	Yes	No	NA	Yes
161	Wang G, 2020	Public Health Treatment Center of Changsha (China)	242	84.71	15.29	0	Yes	Yes	Yes	Yes	Yes	No	Yes	Severe (>= 30 breathings OR Sat <=93% OR OI <= 300 OR IMV OR ICU)	No
162	Wang L, 2020	Renmin Hospital (China)	339	29.50	46.90	23.60	No	Yes	Yes	Yes	No	Yes	Yes	NA	No
163	Wang L, 2020 (2)	People's Hospital of Wuhan University (China)	202	38	38	24	Yes	Yes	No	Yes	No	Yes	No	NA	Yes
164	Wang L, Li X, 2020	Renmin Hospital (China)	116	50.80	39.70	9.50	Yes	Yes	No	No	No	No	No	ARDS	No
165	Wang L, Zou A, 2020	Shanghai Pulmonary Hospital (China)	90	85	15	0	Yes	Yes	Yes	Yes	Yes	No	Yes	Severe (> 30 breathings OR Sat <90%)	No
166	Wang R, 2020	N° 2 People’s Hospital of Fuyang City (China)	125	80	20	0	Yes	Yes	Yes	Yes	Yes	No	Yes	Critical (Severe ARDS OR Shock OR IMV)	No
167	Wang X, 2020	Dongxihu Fangcang Hospital (China)	1012	NR	NR	NR	Yes	Yes	Yes	Yes	Yes	Yes	Yes	Progression in severity status	No

168	Wang Y, 2020	24 hospitals in Jiangsu province (China)	620	91.45	8.55	0	Yes	Yes	Yes	Yes	Yes	No	Yes	Severe (> 30 breathings OR Sat <90%)	Si
169	Wang Y, Lu X, 2020	Tongji Hospital (China)	344	0	0	100	Yes	Yes	Yes	Yes	Yes	Yes	No	NA	No
170	Wang Y, Zhou Y, 2020	The Central Hospital of Wuhan (China)	110	65.45	34.55	0	No	Yes	Yes	Yes	No	No	Yes	Severe (> 30 breathings OR Sat <90%)	Yes
171	Wang Z, 2020	Union Hospital (China)	69	79.70	20.30	0	Yes	Yes	Yes	Yes	No	Yes	No	Severe (> 30 breathings OR Sat <90%)	No
172	Wen C, 2020	Jinyintan Hospital (China)	61	NR	NR	NR	Yes	Yes	Yes	Yes	No	Yes	No	NA	Yes
173	Wen Y, 2020	National Infectious Disease Information System (China)	417	92	0	8	Yes	Yes	Yes	Yes	No	No	Yes	Severe (>30 breathings, Sat ≤93%, OI <300 and/or lung infiltrates >50%) Critical (Severe ARDS OR Shock OR IMV)	Yes
174	Whang S, 2020	Multicenter. Fujian province (China)	165	83	14	3	Yes	No	No	No	No	No	Yes	Critical (Severe ARDS OR Shock OR IMV)	No

175	Wu C, 2020	Wuhan Jinyintan Hospital (China)	201	17.90	82.10	0	Yes	Yes	Yes	Yes	No	No	Yes	ARDS	No
176	Wu J, 2020	The First Affiliated Hospital (China)	280	70	0	30	Yes	Yes	Yes	Yes	Yes	Yes	No	Critical (Severe ARDS OR Shock OR IMV)	Yes
177	Xie H, 2020	Wuhan Jinyintan Hospital (China)	79	65	35	0	Yes	Yes	Yes	No	No	No	No	Severe (> 30 breathings OR Sat <90%)	No
178	Xie J, Covassin N, 2020	Union Hospital, (China)	140	31	52	17	Yes	Yes	Yes	Yes	No	Yes	Yes	Severe (> 30 breathings OR Sat <90%)	Yes
179	Xie J, Hungerford D, 2020	Tongji Hospital and Jinyintan Hospital (China)	444	NR	NR	NR	Yes	Yes	Yes	Yes	No	Yes	No	NA	Yes
180	Xu S, 2020	Union Hospital of Huazhong University and Second People's Hospital of Fuyang City (China)	200	63	17	20	Yes	Yes	Yes	Yes	No	Yes	No	NA	Yes
181	Xu W, 2020	Affiliated Infectious Diseases Hospital (China)	87	50	46	4	Yes	Yes	Yes	Yes	No	No	Yes	OTHER	No

182	Xu Y, 2020	7 Hospitals in Guangzhou (China)	45	0	0	100	Yes	Yes	Yes	Yes	No	Yes	Yes	IMV	No
183	Xu YH, 2020	Fifth Medical Center of Chinese PLA General Hospital (China)	50	74	20	6	Yes	No	Yes	Yes	Yes	NA	Yes	Critical (Severe ARDS OR Shock OR IMV)	No
184	Yan S, 2020	Multiple hospitals in Wuhan (China)	168	78.57	21.43	0	Yes	Yes	Yes	Yes	Yes	Yes	Yes	Critical (Severe ARDS OR Shock OR IMV)	No
185	Yan X, 2020	Hunan Normal University (China)	218	82.57	11.01	6.42	Yes	Yes	Yes	Yes	Yes	Yes	No	Progression in severity status	Yes
186	Yang A, 2020	Zhejiang Xiaoshan Hospital (China)	93	74	26	0	Yes	Yes	No	Yes	No	No	No	Severe (> 30 breathings OR Sat <90%)	Yes
187	Yang G, 2020	Hubei Provincial Hospital of Traditional Chinese Medicine (China)	251	65.74	19.52	14.74	Yes	Yes	Yes	Yes	No	Yes	Yes	Severe (> 30 breathings OR Sat <90%)	No
188	Yang J, 2020	Beijing Tongren Hospital (China)	69	33	29	38	Yes	Yes	Yes	Yes	No	Yes	Yes	Severe (> 30 breathings OR Sat <90%)	Yes
189	Yang L, 2020	Yichang Central People's Hospital (China)	200	85.50	14.50	0	Yes	Yes	Yes	Yes	Yes	Yes	Yes	ICU	No

190	Yang X, Yang Q, 2020	Jinyintan Hospital (China)	1476	NR	NR	NR	Yes	No	No	Yes	No	Yes	No	NA	No
191	Yang X, Yu Y, 2020	Wuhan Jin Yin-tan Hospital (China)	52	0	0	100	Yes	Yes	Yes	Yes	No	Yes	No	NA	No
192	Yin Y, 2020	3 hospitals (China)	112	0	0	100	Yes	Yes	No	Yes	No	Yes	No	NA	Yes
193	Young B E, 2020	4 hospitals (Singapur)	18	100	0	0	Yes	No	Yes	Yes	Yes	Yes	Yes	Severe (Sat <92)	No
194	Yu C, 2020	Tongji Hospital (China)	1663	48.50	51.50	0	Yes	Yes	Yes	Yes	Yes	Yes	Yes	Severe (> 30 breathings OR Sat <90%)	No
195	Yu M, 2020	Zhongnan Hospital of Wuhan University (China)	70	71	29	0	Yes	Yes	Yes	No	Yes	Yes	No	Severe (> 30 breathings OR Sat <90%)	No
196	Yu T, 2020	Dongguan People's Hospital and Nanfang Hospital, Southern Medical University (China)	95	100	0	0	Yes	Yes	Yes	Yes	No	No	Yes	ARDS	Yes
197	Yuan M, 2020	Central Hospital of Wuhan (China)	27	100	0	0	Yes	Yes	Yes	No	Yes	Yes	No	NA	No

198	Zang L, 2020	Wuhan Asia General Hospital (China)	343	NR	NR	NR	Yes	Yes	No	Yes	No	Yes	No	NA	No
199	Zeng L, 2020	Shenzhen Third People's hospital (China)	338	100	0	0	Yes	Yes	Yes	Yes	Yes	Yes	Yes	Severe (> 30 breathings OR Sat <90%)	Yes
200	Zeng Z, 2020	Hankou Hospital (China)	274	NR	NR	NR	No	Yes	No	No	No	Yes	Yes	Severe (> 30 breathings OR Sat <90%)	No
201	Zhang F, 2020	Wuhan No.1 Hospital (China)	48	54.20	45.80	0	Yes	Yes	Yes	Yes	No	Yes	No	NA	Yes
202	Zhang G, Chang H, 2020	Zhongnan Hospital (China)	221	75.11	24.89	0	Yes	Yes	Yes	Yes	Yes	Yes	Yes	Severe (> 30 breathings OR Sat <90%)	No
203	Zhang G, Zhang J, 2020	Wuhan Xinzhou District People's Hospital (China)	95	66.32	33.68	0	Yes	No	Yes	Yes	No	No	Yes	Severe (> 30 breathings OR Sat <90%)	No
204	Zhang H, 2020	Chongqing Public Health Medical Center (China)	43	67.44	32.56	0	Yes	Yes	Yes	Yes	No	No	Yes	Severe (> 30 breathings OR Sat <90%)	No

205	Zhang HY 2020	Zhongnan Hospital , the Fifth Hospital of Wuhan, the Seventh Hospital of Wuhan, Wuhan Hankou Hospital (China)	67	52.20	47.80	0	Yes	Yes	Yes	Yes	No	Yes	Yes	Severe (dyspnea or respiratory failure)	No
206	Zhang J, 2020	Liyuan Hospital (China)	19	0	100	0	Yes	No	Yes	Yes	Yes	Yes	No	NA	No
207	Zhang JJ, 2020	Zhongnan Hospital of Wuhan University, (China)	140	59	41	0	Yes	Yes	Yes	Yes	Yes	No	Yes	Severe (> 30 breathings OR Sat <90%)	No
208	Zhang L, Tao B, 2020	Wuhan Union Hospital (China)	514	54.86	31.52	13.62	No	Yes	Yes	Yes	Yes	No	No	Progression in severity status	No
209	Zhang L, Tao B, 2020 (2)	Xiaohua Hou Wuhan Union Hospital (China)	564	NR	NR	NR	No	Yes	Yes	Yes	No	Yes	Yes	Severe (> 30 breathings OR Sat <90%)	No
210	Zhang L, Wenwu S, 2020	Central Hospital of Wuhan (China)	319	NR	NR	NR	Yes	Yes	Yes	Yes	No	No	No	NA	Yes

211	Zhang L, Zhu F, 2020	Tongji Sino-French New Town Hospital, Union Red Cross Hospital and Union West Hospital (China)	28	NR	NR	NR	Yes	Yes	Yes	Yes	Yes	No	Yes	Critical (Severe ARDS OR Shock OR IMV)	Yes
212	Zhang P, 2020	Nine hospitals in Hubei (China)	1128	NR	NR	NR	Yes	Yes	Yes	Yes	Yes	Yes	No	NA	Yes
213	Zhang R, 2020	Renmin Hospital (China)	120	75	25	0	Yes	Yes	Yes	Yes	Yes	Yes	Yes	Severe (> 30 breathings OR Sat <90%)	Yes
214	Zhang S, 2020	Wuhan Union Hospital West Area (China)	315	43.49	56.51	0	Yes	Yes	Yes	Yes	Yes	Yes	No	NA	Yes
215	Zhang S, Zhao J, 2020	Jinyintan Hospital (China)	262	NR	NR	NR	Yes	Yes	Yes	Yes	Yes	Yes	Yes	Critical (Severe ARDS OR Shock OR IMV)	No
216	Zhang X, Guo W, 2020	11 hospitals (China)	282	NR	NR	NR	Yes	Yes	Yes	No	No	Yes	No	NA	Yes
217	Zhang X, Huan C 2020	Hospitals in Zhejiang province (China)	645	NR	NR	NR	Yes	Yes	Yes	Yes	Yes	No	Yes	Severe (> 30 breathings OR Sat <90%)	No
218	Zhang Y, 2020	West Court of Union Hospital of Huazhong (China)	258	33.71	44.97	21.32	Yes	Yes	Yes	Yes	Yes	Yes	Yes	Critical (Severe ARDS OR Shock OR IMV)	Yes

219	Zhao W, 2020	Second Xiangya Hospital (China)	118	91.53	8.47	0	Yes	Yes	Yes	Yes	Yes	Yes	Yes	Progression in severity status	No
220	Zhao W, 2020	Beijing YouAn Hospital (China)	77	74	26	0	Yes	Yes	Yes	Yes	Yes	No	Yes	ARDS	No
221	Zhen L, 2020	Four hospitals in Hubei province and Chongqing city (China)	258	66.32	33.68	0	Yes	Yes	Yes	Yes	Yes	Yes	Yes	Severe (> 30 breathings OR Sat <90%)	No
222	Zheng F, 2020	North Hospital of Changsha First Hospital (China)	161	81	19	0	Yes	Yes	Yes	Yes	Yes	No	Yes	Severe (> 30 breathings OR Sat <90%)	No
223	Zheng X, 2020	Fifth Affiliated Hospital of Sun Yat-Sen University (China)	52	58	42	0	Yes	Yes	Yes	Yes	Yes	No	Yes	ICU	Yes
224	Zhou B, 2020	First Affiliated Hospital of Xi'an Jiaotong University (China)	34	0	77	23	No	No	Yes	Yes	No	No	Yes	Critical (Severe ARDS OR Shock OR IMV)	No
225	Zhou F, 2020	Jinyintan Hospital and Wuhan Pulmonary Hospital (China)	191	37.70	34.55	27.75	Yes	Yes	Yes	Yes	No	Yes	No	NA	Yes

226	Zhou H, 2020	Wuhan Union Hospital (China)	230	NR	NR	NR	No	yes	Yes	Yes	No	No	No	ICU	No
227	Zhou H, Wanxin C, 2020	Union Hospital (China)	271	89	11	0	Yes	Yes	Yes	Yes	No	Yes	Yes	ICU	No
228	Zhou M, 2020	3 hospitals (China)	240	0	53	47	Yes	Yes	Yes	Yes	Yes	Yes	No	Critical (Severe ARDS OR Shock OR IMV)	Yes
229	Zhou Y, 2020	Central Hospital of Wuhan (China)	377	68.97	31.03	0	Yes	Yes	Yes	Yes	No	No	Yes	Severe (> 30 breathings OR Sat <90%)	Yes
230	Zhu B, 2020	Beijing Tiantan Hospital (China)	107	NR	NR	NR	No	Yes	No	Yes	No	Yes	No	NA	Yes
231	Zhu Q, 2020	Wuhan University Zhongnan Hospital (China)	325	NR	NR	NR	No	Yes	Yes	Yes	Yes	No	No	NA	Yes

Glossary

NA: Not Applicable
ICU: Intensive Care Unit
Sat: oxygen saturation
OI: Oxygenation Index
IMV: invasive mechanical ventilation
SOB: Shortness of Breath
ARDS: Adult Respiratory Distress Syndrome
