# Supplementary material for: Prognostic factors for severity and mortality in patients infected with COVID-19: A systematic review
Source: PLoS One. 2020 Nov 17;15(11):e0241955. doi: 10.1371/journal.pone.0241955 (PMC7671522; doi:10.1371/journal.pone.0241955)
Supplement: S2 Table — This table contains a detailed RoB assessment of included studies. (PDF) [file pone.0241955.s002.pdf]

Table S2. Risk of bias of included studies

Ref. nr.	Author	Study participation	Study Attrition Summary	Prognostic Factor Measurement	Outcome Measurement Summary	Study Confounding Summary	Statistical Analysis and Presentation Summary	Overall risk of bias
30	Argenziano M, 2020	Low	Low	High	High	High	High	High
31	Auld S, 2020	Low	Moderate	Moderate	Low	High	High	High
32	Bai T, 2020	Moderate	Low	Moderate	Low	Moderate	High	Moderate
21	Bai X, 2020	Low	Low	Moderate	Moderate	Low	Low	Low
33	Benelli G, 2020	Low	Low	High	Low	High	High	High
24	Bi Q, 2020	Low	Low	Low	Moderate	Low	Low	Low
34	Cai Q, 2020	Low	Low	High	High	High	High	High

(red) = High risk of bias, (orange) = Moderate risk of bias, (green) = Low risk of bias

Ref. nr.	Author	Study participation	Study Attrition Summary	Prognostic Factor Measurement	Outcome Measurement Summary	Study Confounding Summary	Statistical Analysis and Presentation Summary	Overall risk of bias
35	Cao M, 2020	🟢	🟢	🔴	🔴	🔴	🔴	🔴
36	Cao W, 2020	🟢	🟢	🔴	🔴	🔴	🔴	🔴
37	Chao C, 2020	🟢	🟢	🟠	🟠	🟠	🟠	🟠
38	Chen G, 2020	🟢	🟢	🔴	🔴	🔴	🔴	🔴
39	Chen J, Pan h, 2020	🟢	🟢	🟠	🔴	🔴	🔴	🔴
40	Chen J, Qi T, 2020	🟢	🟢	🟠	🔴	🟠	🟠	🟠
41	Chen L, 2020	🟢	🟢	🔴	🔴	🔴	🔴	🔴
42	Chen M, 2020	🟢	🟢	🔴	🔴	🔴	🔴	🔴
43	Chen M, Yongzhen F, 2020	🟢	🟠	🔴	🟢	🔴	🔴	🔴
44	Chen R, 2020	🟢	🔴	🔴	🟢	🟢	🟠	🔴

🔴 = High risk of bias, 🟠 = Moderate risk of bias, 🟢 = Low risk of bias

Ref. nr.	Author	Study participation	Study Attrition Summary	Prognostic Factor Measurement	Outcome Measurement Summary	Study Confounding Summary	Statistical Analysis and Presentation Summary	Overall risk of bias
45	Chen TL, 2020	🟢	🟢	🟠	🟢	🟠	🟠	🟠
46	Chen W, 2020	🟢	🟢	🔴	🔴	🔴	🔴	🔴
47	Chen X, Liu Z, 2020	🟠	🟢	🟠	🟢	🔴	🟢	🔴
48	Chen X, Zhang Y 2020	🟢	🟢	🔴	🔴	🔴	🔴	🔴
49	Chen X, Zhao B 2020	🟢	🟢	🔴	🔴	🔴	🔴	🔴
50	Chen X, Zheng F, 2020	🟢	🟢	🔴	🔴	🔴	🔴	🔴
51	Chen Y, 2020	🟢	🟢	🔴	🔴	🔴	🔴	🔴
52	Cheng Y, 2020	🟢	🟢	🟠	🟢	🔴	🔴	🔴
53	Chu J, 2020	🟢	🟢	🔴	🟠	🔴	🔴	🔴
54	Colaneri M, 2020	🟢	🟢	🟠	🔴	🟠	🟠	🔴
55	Colombi D, 2020	🟢	🟢	🟠	🟠	🟠	🟠	🟠

🔴 = High risk of bias, 🟠 = Moderate risk of bias, 🟢 = Low risk of bias

Ref. nr.	Author	Study participation	Study Attrition Summary	Prognostic Factor Measurement	Outcome Measurement Summary	Study Confounding Summary	Statistical Analysis and Presentation Summary	Overall risk of bias
56	Cummings M, 2020	🟢	🟢	🟢	🟢	🟠	🟢	🟢
57	Docherty AB, 2020	🟢	🟠	🟢	🟢	🟢	🟢	🟢
23	Dong J, 2020	🟢	🟢	🟠	🟠	🟠	🟠	🟠
58	Dreher M, 2020	🟢	🟢	🔴	🔴	🔴	🔴	🔴
59	Du RH, 2020	🟢	🟢	🟢	🟢	🟠	🟠	🟢
60	Duan Q, 2020	🟢	🟢	🟠	🟢	🟠	🟠	🟠
61	Fan J, 2020	🟢	🟢	🔴	🟢	🔴	🔴	🔴
62	Fan T, 2020	🟢	🔴	🟠	🟠	🟠	🟢	🟠
63	Fang L, 2020	🟢	🟢	🔴	🔴	🟠	🟠	🔴
64	Fei J, 2020	🟢	🟢	🟠	🟢	🔴	🔴	🔴

🔴 = High risk of bias, 🟠 = Moderate risk of bias, 🟢 = Low risk of bias

Ref. nr.	Author	Study participation	Study Attrition Summary	Prognostic Factor Measurement	Outcome Measurement Summary	Study Confounding Summary	Statistical Analysis and Presentation Summary	Overall risk of bias
65	Feng Y, 2020	Low	Low	Moderate	Low	Moderate	Moderate	Moderate
66	Feng Z, 2020	Low	Low	Moderate	Moderate	Moderate	Moderate	Moderate
67	Fu L, Fei J, Xiang H, 2020	Low	Low	Moderate	Low	High	Moderate	High
68	Fu L, Fei J, Xu S, 2020	Low	Low	Low	Low	High	High	High
69	Gao L, 2020	Low	Low	Moderate	Low	Moderate	Moderate	Moderate
70	Gao Y, 2020	Low	Low	Moderate	High	High	High	High
27	Gong J, 2020	Low	Low	High	High	High	Moderate	High
71	Grasselli G, 2020	Low	High	High	Low	High	High	High
72	Gu T, 2020	Low	Low	Moderate	Low	Moderate	Low	Moderate
73	Guan W, Liang W, 2020	Low	Low	Moderate	Low	Moderate	Low	Moderate

● = High risk of bias, ● = Moderate risk of bias, ● = Low risk of bias

Ref. nr.	Author	Study participation	Study Attrition Summary	Prognostic Factor Measurement	Outcome Measurement Summary	Study Confounding Summary	Statistical Analysis and Presentation Summary	Overall risk of bias
74	Guan W, Ni Z, 2020	🟢	🟢	🟠	🟠	🔴	🔴	🔴
75	Guo T, 2020	🟠	🟢	🟠	🟢	🔴	🔴	🔴
76	Guo TM, 2020	🟢	🟢	🟠	🟢	🟢	🟢	🟠
77	Guo W, 2020	🟠	🟢	🟠	🟢	🔴	🔴	🔴
78	Han H, 2020	🔴	🟢	🟢	🔴	🔴	🔴	🔴
79	Han M, 2020	🟢	🟢	🔴	🔴	🔴	🔴	🔴
80	Han Y, 2020	🟢	🟢	🔴	🔴	🔴	🟢	🔴
81	He W, 2020	🟢	🟢	🔴	🟢	🔴	🔴	🔴
82	He XW, 2020	🟢	🔴	🟠	🟢	🔴	🔴	🔴
83	Herold T, 2020	🟢	🟢	🔴	🟢	🔴	🔴	🔴

🔴 = High risk of bias, 🟠 = Moderate risk of bias, 🟢 = Low risk of bias

Ref. nr.	Author	Study participation	Study Attrition Summary	Prognostic Factor Measurement	Outcome Measurement Summary	Study Confounding Summary	Statistical Analysis and Presentation Summary	Overall risk of bias
84	Hu B, 2020	🟢	🟢	🟠	🟢	🔴	🔴	🔴
85	Hu L, 2020	🟢	🟢	🟠	🟠	🟢	🟢	🟠
86	Hu X, 2020	🟢	🟢	🔴	🔴	🔴	🔴	🔴
87	Hu Z, 2020	🔴	🟢	🔴	🟢	🔴	🔴	🔴
88	Huang C, 2020	🟢	🟢	🟢	🔴	🔴	🔴	🔴
89	Huang H, 2020	🟢	🟢	🟠	🟠	🟠	🟠	🟠
90	Huang M, 2020	🟢	🟢	🟠	🟠	🔴	🔴	🔴
91	Jiang X, 2020	🟢	🟢	🔴	🔴	🔴	🔴	🔴
92	Jin JM, 2020	🟢	🟢	🟠	🟢	🔴	🔴	🔴
93	Jin X, 2020	🟠	🟢	🔴	🔴	🔴	🟠	🔴

🔴 = High risk of bias, 🟠 = Moderate risk of bias, 🟢 = Low risk of bias

Ref. nr.	Author	Study participation	Study Attrition Summary	Prognostic Factor Measurement	Outcome Measurement Summary	Study Confounding Summary	Statistical Analysis and Presentation Summary	Overall risk of bias
94	Kalligeros M, 2020	Low	Low	Moderate	Low	Moderate	Low	Moderate
95	Kuang Y, 2020	Low	Low	High	High	High	High	High
96	Lee H, 2020	Low	Low	High	Low	Moderate	Low	High
97	Lei L, 2020	Low	Low	High	High	High	High	High
98	Lei S, 2020	Low	Low	High	High	High	High	High
99	Li H. 2020	Low	Low	Moderate	High	High	Low	High
100	Li J, Long X, 2020	Low	Low	High	Low	High	High	High
101	Li J, Meng L, 2020	Low	Low	Moderate	Low	Low	Moderate	Moderate
102	Li J, Wang X, 2020	Moderate	Low	High	Low	High	High	High
103	Li K, 2020	Low	Low	Moderate	High	High	High	High

(red) = High risk of bias, (orange) = Moderate risk of bias, (green) = Low risk of bias

Ref. nr.	Author	Study participation	Study Attrition Summary	Prognostic Factor Measurement	Outcome Measurement Summary	Study Confounding Summary	Statistical Analysis and Presentation Summary	Overall risk of bias
25	Li X, 2020	Low	Low	Moderate	Low	Moderate	Low	Moderate
104	Li Y, 2020	Low	Low	Low	Low	High	High	High
105	Li YK, 2020	Low	Low	High	Low	High	High	High
106	Lian J, 2020	Low	Low	High	High	High	High	High
107	Liang W, 2020	Low	High	Low	High	Moderate	Low	Moderate
108	Liao X, 2020	Low	High	Moderate	Moderate	High	High	High
109	Liu F, 2020	Low	Low	Moderate	High	High	High	High
110	Liu J, 2020	Low	Low	Moderate	Moderate	High	Moderate	High
111	Liu J, Li S, 2020	Low	Low	High	High	High	High	High
112	Liu J, Liu Y, 2020	Low	Low	Moderate	Moderate	Moderate	Moderate	Moderate

= High risk of bias, = Moderate risk of bias, = Low risk of bias

Ref. nr.	Author	Study participation	Study Attrition Summary	Prognostic Factor Measurement	Outcome Measurement Summary	Study Confounding Summary	Statistical Analysis and Presentation Summary	Overall risk of bias
113	Liu KC, 2020	🟢	🟢	🔴	🟠	🔴	🔴	🔴
114	Liu R, 2020	🟢	🟢	🟠	🟠	🟠	🟠	🟠
115	Liu T, 2020	🟢	🟢	🔴	🔴	🔴	🔴	🔴
116	Liu W, 2020	🟢	🟢	🟠	🟠	🟢	🟠	🟠
117	Liu Y, 2020	🟢	🟢	🟢	🟠	🔴	🔴	🔴
118	Liu Y, Bi L, 2020	🟢	🟢	🔴	🔴	🔴	🔴	🔴
119	Liu Y, Du X, 2020	🟠	🟢	🟠	🟢	🟢	🟢	🟠
120	Liu Y, Sun W, Guo Y, 2020	🟢	🟢	🟠	🟢	🟠	🟠	🟠
121	Liu Y, Sun W, Li J, 2020	🟠	🟢	🔴	🔴	🔴	🔴	🔴
122	Liu Y, Yang Y, 2020	🟢	🟢	🟠	🟢	🔴	🔴	🔴

🔴 = High risk of bias, 🟠 = Moderate risk of bias, 🟢 = Low risk of bias

Ref. nr.	Author	Study participation	Study Attrition Summary	Prognostic Factor Measurement	Outcome Measurement Summary	Study Confounding Summary	Statistical Analysis and Presentation Summary	Overall risk of bias
123	Lu J, 2020	🟠	🟢	🟠	🟢	🟠	🟠	🟠
124	Luo XM, 2020	🟠	🟢	🟠	🟢	🟠	🟠	🟠
125	Luo XM, 2020	🟠	🟢	🟠	🟢	🔴	🔴	🔴
126	Lv X, 2020	🟢	🟢	🟠	🔴	🔴	🔴	🔴
127	Ma J, 2020	🟢	🟢	🔴	🔴	🔴	🔴	🔴
128	Ma K, 2020	🟢	🟢	🔴	🔴	🟢	🟠	🔴
129	Ma Y, 2020	🟢	🟢	🟠	🔴	🟠	🔴	🟠
130	Mehra MR, 2020	🟠	🟢	🟢	🟢	🟠	🟢	🟢
131	Mo P, 2020	🟢	🟢	🟠	🔴	🟢	🟠	🟠
132	Niu S, 2020	🟢	🔴	🟢	🟢	🔴	🔴	🔴

🔴 = High risk of bias, 🟠 = Moderate risk of bias, 🟢 = Low risk of bias

Ref. nr.	Author	Study participation	Study Attrition Summary	Prognostic Factor Measurement	Outcome Measurement Summary	Study Confounding Summary	Statistical Analysis and Presentation Summary	Overall risk of bias
133	Pan L, 2020	Moderate	Low	Moderate	Low	High	High	High
134	Paranjpe I, 2020	Low	High	Moderate	Low	High	High	High
135	Peng YD, 2020	Moderate	Low	Moderate	Low	High	High	High
136	Qi D, 2020	Low	Moderate	High	High	High	High	High
137	Qi X, 2020	Moderate	High	Moderate	High	High	High	High
138	Qin X, 2020	Low	Low	High	High	High	High	High
139	Qu R, 2020	Moderate	Low	High	Moderate	Moderate	Moderate	High
140	Ran J, 2020	Low	Low	High	High	High	Moderate	High
141	Rentsch CT, 2020	Low	Low	Moderate	High	Low	Low	Moderate
142	Rossi PG, 2020	Low	Low	Low	Low	Moderate	Low	Low

(red) = High risk of bias, (orange) = Moderate risk of bias, (green) = Low risk of bias

Ref. nr.	Author	Study participation	Study Attrition Summary	Prognostic Factor Measurement	Outcome Measurement Summary	Study Confounding Summary	Statistical Analysis and Presentation Summary	Overall risk of bias
143	Ruan Q, 2020	🟢	🟢	🔴	🟢	🔴	🔴	🔴
144	Sakar J, 2020	🟢	🔴	🔴	🔴	🔴	🟠	🔴
145	Shi H, 2020	🟠	🟢	🔴	🔴	🟢	🟠	🔴
146	Shi S, 2020	🟠	🟠	🟠	🟢	🟢	🟢	🟠
147	Shi W, 2020	🟢	🟢	🔴	🔴	🔴	🔴	🔴
148	Shi Y, 2020	🟢	🟢	🔴	🔴	🟠	🔴	🔴
149	Song C, 2020	🟢	🟢	🔴	🔴	🔴	🔴	🔴
150	Sun F, 2020	🟢	🟢	🔴	🔴	🔴	🔴	🔴
151	Sun X, 2020	🟢	🟢	🟠	🟢	🟠	🟠	🟠
152	Sun Y, 2020	🟢	🟢	🟠	🔴	🔴	🟠	🔴

🔴 = High risk of bias, 🟠 = Moderate risk of bias, 🟢 = Low risk of bias

Ref. nr.	Author	Study participation	Study Attrition Summary	Prognostic Factor Measurement	Outcome Measurement Summary	Study Confounding Summary	Statistical Analysis and Presentation Summary	Overall risk of bias
153	Tabata S, 2020	🟢	🟢	🔴	🔴	🟢	🔴	🔴
154	Tan L, 2020	🔴	🟢	🔴	🔴	🔴	🔴	🔴
155	Tang N, 2020	🟢	🟢	🟠	🟢	🔴	🔴	🔴
156	Tian S, 2020	🟢	🟢	🔴	🔴	🔴	🔴	🔴
157	Tu WJ, 2020	🔴	🟢	🔴	🟢	🔴	🔴	🔴
158	Wan S, 2020	🟢	🟢	🔴	🔴	🔴	🔴	🔴
159	Wang D, Hu B, 2020	🟢	🟢	🔴	🔴	🔴	🔴	🔴
160	Wang D, Yin Y, 2020	🔴	🟢	🔴	🟢	🟠	🟠	🔴
161	Wang G, 2020	🟢	🟢	🔴	🔴	🔴	🔴	🔴
162	Wang L, 2020	🟢	🔴	🟠	🟢	🟠	🟠	🟠

🔴 = High risk of bias, 🟠 = Moderate risk of bias, 🟢 = Low risk of bias

Ref. nr.	Author	Study participation	Study Attrition Summary	Prognostic Factor Measurement	Outcome Measurement Summary	Study Confounding Summary	Statistical Analysis and Presentation Summary	Overall risk of bias
163	Wang L, 2020 (2)	🟢	🟢	🟠	🟢	🟠	🟠	🟠
164	Wang L, Li X, 2020	🟢	🟢	🔴	🔴	🔴	🔴	🔴
165	Wang L, Zou A, 2020	🟢	🟢	🔴	🔴	🔴	🔴	🔴
166	Wang R, 2020	🟢	🟢	🔴	🔴	🔴	🔴	🔴
167	Wang X, 2020	🔴	🟢	🟠	🟠	🔴	🔴	🔴
168	Wang Y, 2020	🟢	🟢	🟠	🟠	🟢	🟠	🟠
169	Wang Y, Lu X, 2020	🟢	🟢	🔴	🟢	🔴	🔴	🔴
170	Wang Y, Zhou Y, 2020	🟢	🟢	🔴	🔴	🟠	🔴	🔴
171	Wang Z, 2020	🟢	🟢	🟠	🟢	🔴	🔴	🔴
172	Wen C, 2020	🟢	🟢	🟠	🟢	🟠	🟠	🟠

🔴 = High risk of bias, 🟠 = Moderate risk of bias, 🟢 = Low risk of bias

Ref. nr.	Author	Study participation	Study Attrition Summary	Prognostic Factor Measurement	Outcome Measurement Summary	Study Confounding Summary	Statistical Analysis and Presentation Summary	Overall risk of bias
173	Wen Y, 2020	🟢	🟢	🔴	🔴	🟠	🟠	🔴
174	Whang S, 2020	🟢	🟢	🟠	🟢	🔴	🔴	🔴
175	Wu C, 2020	🟢	🟠	🟠	🟢	🔴	🔴	🔴
176	Wu J, 2020	🟢	🟢	🔴	🔴	🟠	🟠	🔴
177	Xie H, 2020	🟢	🟢	🔴	🔴	🔴	🔴	🔴
178	Xie J, Covassin N, 2020	🟢	🟢	🟠	🟢	🟠	🟢	🟠
179	Xie J, Hungerford D, 2020	🟢	🟠	🟠	🟢	🟠	🟠	🟠
180	Xu S, 2020	🟢	🟢	🔴	🔴	🟠	🟠	🔴
181	Xu W, 2020	🟢	🔴	🟠	🟢	🔴	🔴	🔴
182	Xu Y, 2020	🟢	🟢	🟠	🟢	🔴	🔴	🔴

🔴 = High risk of bias, 🟠 = Moderate risk of bias, 🟢 = Low risk of bias

Ref. nr.	Author	Study participation	Study Attrition Summary	Prognostic Factor Measurement	Outcome Measurement Summary	Study Confounding Summary	Statistical Analysis and Presentation Summary	Overall risk of bias
183	Xu YH, 2020	Moderate	Low	High	High	High	High	High
184	Yan S, 2020	Low	Low	High	High	High	High	High
185	Yan X, 2020	Low	Low	Moderate	Moderate	Moderate	Moderate	Moderate
186	Yang A, 2020	Low	Low	High	High	Moderate	Low	High
187	Yang G, 2020	Low	High	High	Low	High	High	High
188	Yang J, 2020	High	Low	High	Low	Moderate	Low	High
189	Yang L, 2020	Low	Low	High	High	High	High	High
190	Yang X, Yang Q, 2020	Low	Low	High	Low	Moderate	Low	High
191	Yang X, Yu Y, 2020	Moderate	Low	Moderate	Low	High	High	High
192	Yin Y, 2020	Low	Low	Moderate	Low	Moderate	Moderate	Moderate

(red) = High risk of bias, (orange) = Moderate risk of bias, (green) = Low risk of bias

Ref. nr.	Author	Study participation	Study Attrition Summary	Prognostic Factor Measurement	Outcome Measurement Summary	Study Confounding Summary	Statistical Analysis and Presentation Summary	Overall risk of bias
193	Young B E, 2020	🟢	🟢	🔴	🔴	🔴	🔴	🔴
194	Yu C, 2020	🟢	🟢	🟠	🔴	🔴	🔴	🔴
195	Yu M, 2020	🟢	🟢	🔴	🔴	🔴	🔴	🔴
196	Yu T, 2020	🟢	🟢	🔴	🔴	🟠	🟠	🔴
197	Yuan M, 2020	🟢	🟢	🟠	🟢	🔴	🔴	🔴
198	Zang L, 2020	🔴	🟢	🟠	🟢	🟠	🟠	🔴
199	Zeng L, 2020	🟢	🔴	🟠	🟠	🟠	🟠	🟠
200	Zeng Z, 2020	🟢	🟢	🔴	🔴	🔴	🔴	🔴
201	Zhang F, 2020	🟠	🟢	🟠	🟢	🟠	🟢	🟠
202	Zhang G, Chang H, 2020	🟢	🟢	🔴	🔴	🔴	🔴	🔴

🔴 = High risk of bias, 🟠 = Moderate risk of bias, 🟢 = Low risk of bias

Ref. nr.	Author	Study participation	Study Attrition Summary	Prognostic Factor Measurement	Outcome Measurement Summary	Study Confounding Summary	Statistical Analysis and Presentation Summary	Overall risk of bias
203	Zhang G, Zhang J, 2020	Moderate	Low	Moderate	Moderate	High	High	High
204	Zhang H, 2020	Low	Low	High	High	High	High	High
205	Zhang HY 2020	Low	Low	Moderate	Low	High	High	High
206	Zhang J, 2020	Low	Low	High	Low	High	High	High
207	Zhang JJ, 2020	Low	Low	High	High	High	High	High
208	Zhang L, Tao B, 2020	Low	Low	High	High	High	High	High
209	Zhang L, Tao B, 2020 (2)	Low	Low	High	Low	High	High	High
210	Zhang L, Wenwu S, 2020	Low	Low	Moderate	Low	Moderate	Moderate	Moderate
211	Zhang L, Zhu F, 2020	Low	Moderate	Moderate	Moderate	Moderate	Low	Moderate
212	Zhang P, 2020	Moderate	Low	High	Low	Moderate	Low	High

● = High risk of bias, ● = Moderate risk of bias, ● = Low risk of bias

Ref. nr.	Author	Study participation	Study Attrition Summary	Prognostic Factor Measurement	Outcome Measurement Summary	Study Confounding Summary	Statistical Analysis and Presentation Summary	Overall risk of bias
213	Zhang R, 2020	🟢	🟢	🔴	🔴	🟠	🟠	🔴
214	Zhang S, 2020	🟢	🟢	🟠	🟢	🟠	🟠	🟠
215	Zhang S, Zhao J, 2020	🟢	🟢	🟠	🟢	🔴	🔴	🔴
216	Zhang X, Guo W, 2020	🟢	🟢	🟠	🟢	🟠	🔴	🟠
217	Zhang X, Huan C , 2020	🟢	🟢	🔴	🔴	🟠	🟠	🔴
218	Zhang Y, 2020	🟢	🟢	🟠	🟢	🟠	🟢	🟠
219	Zhao W, 2020	🟢	🟢	🟠	🟠	🔴	🔴	🔴
220	Zhao W, 2020	🟢	🟠	🟠	🟠	🟠	🟠	🟠
221	Zhen L, 2020	🟢	🟢	🔴	🔴	🔴	🔴	🔴
222	Zheng F, 2020	🟢	🟢	🟠	🔴	🔴	🔴	🔴

🔴 = High risk of bias, 🟠 = Moderate risk of bias, 🟢 = Low risk of bias

Ref. nr.	Author	Study participation	Study Attrition Summary	Prognostic Factor Measurement	Outcome Measurement Summary	Study Confounding Summary	Statistical Analysis and Presentation Summary	Overall risk of bias
223	Zheng X, 2020	🔴	🟢	🟠	🔴	🟠	🟠	🔴
224	Zhou B, 2020	🟢	🟢	🔴	🔴	🔴	🔴	🔴
225	Zhou F, 2020	🟢	🔴	🟠	🟢	🟢	🟢	🟠
226	Zhou H, 2020	🟢	🟢	🔴	🔴	🔴	🔴	🔴
227	Zhou H, Wanxin C, 2020	🟢	🟢	🔴	🔴	🔴	🔴	🔴
228	Zhou M, 2020	🟠	🟢	🟠	🟠	🔴	🟠	🔴
229	Zhou Y, 2020	🟢	🟢	🔴	🔴	🟠	🟠	🔴
230	Zhu B, 2020	🟢	🟢	🟠	🟢	🟠	🟠	🟠
231	Zhu Q, 2020	🟢	🟠	🟢	🟢	🟠	🟢	🟠

🔴 = High risk of bias, 🟠 = Moderate risk of bias, 🟢 = Low risk of bias
