# Supplementary material for: Prognostic factors for severity and mortality in patients infected with COVID-19: A systematic review
Source: PLoS One. 2020 Nov 17;15(11):e0241955. doi: 10.1371/journal.pone.0241955 (PMC7671522; doi:10.1371/journal.pone.0241955)
Supplement: S3 Table — This table presents the complete results including all assessed candidate variables. (DOCX) [file pone.0241955.s003.docx]

Supplementary table 3. Summary of findings of all candidate variables

| **Prognostic factor** | **Mortality** | | | | | **Severe COVID-19 disease** | | | | |
| --- | --- | --- | --- | --- | --- | --- | --- | --- | --- | --- |
|  | **Number of patients (studies)** | **Odds ratio (95%CI)** | **Risk without prognostic factor** | **Risk with prognostic factor** | **Certainty of the evidence** | **Number of patients (studies)** | **Odds ratio (95%CI)** | **Risk without prognostic factor** | **Risk with prognostic factor** | **Certainty of the evidence** |
| **Socio-demographic characteristics** | | | | | | | | | | |
| Age  Definition: Older than 50 - 65 years | 27616 (29) | 4.43 (3.39-5.81) | 1.9% | 8.1% | ⨁⨁⨁⨁  HIGH | 14456 (53) | 4.08 (3.29-5.07) | 7.7% | 25% | ⨁⨁⨁⨁  HIGH |
|  |  |  | 6.2% increase in mortality. Between 4.7% more and 7.4 more. | |  |  |  | 17.3% increase severe COVID-19 disease. Between 14.5% increase and 28.5 increase. | |  |
| Age  Definition: 10 years increase | 11962 (19) | 1.80 (1.54-2.10) | 9% | 15.1% | ⨁⨁⨁⨁  HIGH | 14456 (53) | 1.63 (1.47-1.80) | 13% | 19.6% | ⨁⨁⨁⨁  HIGH |
|  |  |  | 6.1% increase in mortality. Between 4.2% more and 8.2 more. | |  |  |  | 6.6% increase severe COVID-19 disease. Between 5% increase and 8.2% increase. | |  |
| Sex  Definition:  Male | 31948 (58) | 1.72 (1.5-1.98) | 8% | 13% | ⨁⨁⨁◯ ^d^  MODERATE | 25032 (122) | 1.53 (1.4-1.67) | 10.8% | 15.5% | ⨁⨁⨁⨁  HIGH |
|  |  |  | 5% increase in mortality. Between 4% more and 7% more | |  |  |  | 4.7% increase severe COVID-19 disease. Between 3.7% more and 5.6% more | |  |
| Smoking  Definition: Active, present smoker | 12025 (16) | 1.57 (1.19-2.07) | 8.7% | 13% | ⨁⨁⨁⨁  HIGH | 9147 (45) | 1.65 (1.25-2.17) | 12.1% | 18.4% | ⨁⨁⨁◯  MODERATE ^d^ |
|  |  |  | 4.3% increase in mortality. Between 1.5% more and 7.5% more | |  |  |  | 6.3% increase severe COVID-19 disease. Between 2.7% more and 10.2% more | |  |
| **Medical illness and patient history** | | | | | | | | | | |
| Cardiovascular disease  Definition:  Coronary heart disease or congestive heart failure | 37156 (51) | 2.12 (1.77-2.56) | 8.1% | 15.5% | ⨁⨁⨁◯  MODERATE ^d^ | 16679 (73) | 3.34 (2.71-4.1) | 12.2% | 31.3% | ⨁⨁⨁◯  MODERATE^d^ |
|  |  |  | 7.5% increase in mortality. Between 5.4% more and 9.7% more | |  |  |  | 19.1% increase severe COVID-19 disease. Between 15.1% more and 23.1% more | |  |
| Cardiac arrhythmia: as previous condition or new clinical finding | 12729 (6) | 2.13 (1.72-2.65) | 7% | 13.6% | ⨁⨁⨁⨁  HIGH | 747 (4) | 16.51 (6.69-40.77) | 6.5% | 35.5% | ⨁⨁◯◯  LOW ^a,c,e^ |
|  |  |  | 6.5% increase in mortality. Between 4.7% more and 8.4% more | |  |  |  | 29% increase severe COVID-19 disease.  Between 22.6% more and 32.3% more | |  |
| Cerebrovascular disease  Definition:  History of stroke or CNS disease | 15294 (26) | 2.85 (2.02-4.01) | 8.7% | 21.3% | ⨁⨁⨁⨁  HIGH | 11050 (42) | 2.67 (1.84-3.87) | 12.7% | 27.8% | ⨁⨁⨁◯  MODERATE ^d^ |
|  |  |  | 12.6% increase in mortality. Between 7.5% more and 18.5% more | |  |  |  | 15.1% increase severe COVID-19 disease. Between 8.4% more and 22.8% more | |  |
| Diabetes | 30303 (52) | 1.84 (1.61-2.1) | 7.9% | 13.6% | ⨁⨁⨁⨁  HIGH | 21381 (97) | 2.51 (2.2-2.87) | 12% | 25.2% | ⨁⨁⨁⨁  HIGH |
|  |  |  | 5.6% increase in mortality. Between 4.3% more and 7% more | |  |  |  | 13.2% increase severe COVID-19 disease. Between 11% more and 15.5% more | |  |
| Arterial hypertension | 31341 (52) | 2.02 (1.71-2.38) | 7% | 13% | ⨁⨁⨁⨁  HIGH | 20817 (94) | 2.5 (2.21-2.92) | 11.1% | 23.3% | ⨁⨁⨁◯  MODERATE^d^ |
|  |  |  | 6% increase in mortality. Between 4.5% more and 7.3% more | |  |  |  | 12.1% increase severe COVID-19 disease. Between 10.4% more and 14.4% more | |  |
| Obesity: BMI > 25-30 | 9127 (3) | 1.41 (1.15-1.74) | 8.5% | 11.5% | ⨁⨁⨁⨁  HIGH | 1140 (8) | 3.74 (2.37-5.89) | 10.2% | 35% | ⨁⨁⨁⨁  HIGH |
|  |  |  | 3.1% increase in mortality. Between 1.2% more and 5.1% more | |  |  |  | 16.7% increase in severe COVID-19 disease. Between 9.6% more and 24.7% more | |  |
| Chronic kidney disease  Definition: KDIGO definition of CKD | 23448 (28) | 2.27 (1.69-3.05) | 8.5% | 17.2% | ⨁⨁⨁⨁  HIGH | 12056 (42) | 2.21 (1.51-3.24) | 12.8% | 24.5% | ⨁⨁◯◯  LOW ^a,d^ |
|  |  |  | 8.8% increase in mortality. Between 5.1% more and 12.9% more | |  |  |  | 11.7% increase severe COVID-19 disease. Between 5.4% more and 19.2% more | |  |
| Asthma | 1371 (3) | 0.89 (0.56-1.42) | 9.1% | 8.2% | ⨁⨁◯◯  LOW ^a,b^ | 2244 (6) | 1.18 (0.79-1.76) | 13% | 15% | ⨁⨁◯◯  LOW ^a,b^ |
|  |  |  | 0.9% decrease in mortality. Between 4% less and 3% more. | |  |  |  | 2% increase severe COVID-19 disease. Between 2% less and 7.8% more. | |  |
| COPD | 34759 (41) | 2.43 (1.88-3.14) | 8.5% | 18.4% | ⨁⨁⨁⨁  HIGH | 15468 (65) | 2.7 (2.14-3.4) | 12.6% | 27.9% | ⨁⨁⨁⨁  HIGH |
|  |  |  | 9.8% increase in mortality. Between 6.4% more and 13.6% more. | |  |  |  | 15..3% increase in mortality. Between 11% more and 20% more. | |  |
| Tuberculosis | 700 (4) | 2.52 (0.68-9.43) | 8.8% | 19.6% | ⨁⨁◯◯  LOW ^a,b^ | 1666 (16) | 1.004 (0.43-2.31) | 13% | 13% | ⨁⨁◯◯  LOW ^a,b^ |
|  |  |  | 10.7 increase in mortality. Between 2.7% less and 36% more. | |  |  |  | 0% increase severe COVID-19 disease. Between 5% less and 9% more. | |  |
| Cancer  Definition: Solid or active haematologic cancer | 22734 (25) | 1.35 (1.17-1.55) | 8.9% | 11.6% | ⨁⨁⨁⨁  HIGH | 15156 (58) | 2.06 (1.64-2.58) | 12.8% | 23.2% | ⨁⨁⨁◯  MODERATE^d^ |
|  |  |  | 2.7% increase in mortality. Between 1.4% more and 4.2% more | |  |  |  | 10.4% increase severe COVID-19 disease. Between 6.6% more and 14.5% more | |  |
| HIV infection | 0 (0) | NA | NA | NA | NA | 612 (4) | 0.73 (0.03-17.99) | 13% | 9.9% | ⨁⨁◯◯  LOW |
|  |  |  | NA | |  |  |  | 3.2% decrease severe COVID-19 disease. Between 12.7% less and 56.7% more | |  |
| Immunocompromised  Definition: As defined by the authors including patients on immunosuppressive medications and/or with immunosuppressive medical conditions | 8977 (2) | 1.65 (1.07-2.55) | 9% | 14% | ⨁⨁◯◯  LOW | 2768 (9) | 1.67 (0.6-4.63) | 13% | 19.9% | ⨁⨁◯◯  LOW |
|  |  |  | 5% increase in mortality. Between 0.6% more and 11.1% more | |  |  |  | 7% increase severe COVID-19 disease. Between 4.8% less and 27.7% more | |  |
| Autoimmune disease | 803 (4) | 1.82 (0.36-9.15) | 9% | 15.2% | ⨁◯◯◯  VERY LOW | 1663 (3) | 1.12 (0.52-.4) | 13% | 14.3% | ⨁⨁◯◯  LOW |
|  |  |  | 6.2% increase in mortality. Between 5.6% less and 38% more | |  |  |  | 1.3% increase severe COVID-19 disease. Between 5.8% less and 13.4% more | |  |
| Malnutrition | 52 (1) | 5.81 (0.01-4090) | 9% | 36.5% | ⨁◯◯◯  VERY LOW | 0 (0) | NA | NA | NA | NA |
|  |  |  | 27.5% increase in mortality. Between 8.9% less and 90.8% more | |  |  |  | NA | |  |
| Dementia | 8922 (3) | 1.54 (1.31-1.81) | 9% | 13.2% | ⨁⨁⨁⨁  HIGH | 0 (0) | NA | NA | NA | NA |
|  |  |  | 4.2% increase in mortality. Between 2.5% more and 6.2% more | |  |  |  | NA | |  |
| Chronic liver disease | 2149 (12) | 1.7 (0.97-2.99) | 8.8% | 14.2% | ⨁⨁◯◯  LOW | 10358 (44) | 1.53 (1.18-1.99) | 12.8% | 18.4% | ⨁⨁◯◯  LOW |
|  |  |  | 5.3% increase in mortality. Between 0.2% less and 13.2% more | |  |  |  | 5.5% increase severe COVID-19 disease. Between 2% more and 9.7% more | |  |
| Thyroid disease | 0 (0) | NA | NA | NA | NA | 731 (7) | 2.62 (0.85-8.02) | 12.8% | 27.8% | ⨁⨁◯◯  LOW |
|  |  |  | NA | |  |  |  | 14.9% increase severe COVID-19 disease. Between 1.7% less and 40% more. | |  |
| Chronic gastric disease  Definition: History of peptic ulcer or gastritis | 841 (5) | 0.76 (0.42-1.39) | 9.2% | 7.2% | ⨁⨁◯◯  LOW | 1643 (9) | 1.55 (1.1-2.18) | 12.6% | 18.2% | ⨁⨁◯◯  LOW |
|  |  |  | 2.1% decrease in mortality. Between 5.4% less and 3% more | |  |  |  | 5.6% increase severe COVID-19 disease. Between 1.1% more and 10.8% more. | |  |
| Dyslipidemia | 11273 (4) | 1.26 (1.06-1.5) | 8.9% | 11% | ⨁⨁⨁◯  MODERATE^b^ | 559 (4) | 0.63 (0.22-1.83) | 13.1% | 8.7% | ⨁⨁◯◯  LOW ^a,b^ |
|  |  |  | 2.1% increase in mortality. Between 0.5% more and 3.9% more. | |  |  |  | 4.4% decrease severe COVID-19 disease. Between 10% less and 8.3% more. | |  |
| Any chronic condition or comorbidities | 4406 (16) | 3.3 (2.18-5) | 5.9% | 16.2% | ⨁⨁⨁◯ ^a^  MODERATE | 6640 (40) | 3.16 (2.71-3.68) | 8.2% | 20.1% | ⨁⨁⨁⨁  HIGH |
|  |  |  | 10.3% increase in mortality. Between 6.8% more and 13.4% more | |  |  |  | 12% increase severe COVID-19 disease. Between 10.6% more and 13.2% more | |  |
| **Symptoms, vital signs and physical examination** | | | | | | | | | | |
| Respiratory failure  Definition: increased respiratory rate, abnormal blood gases (hypoxemia, hypercapnia, or both), and evidence of increased work of breathing | 1887 (8) | 21.17 (4.9-91.3) | 3.1% | 23.4% | ⨁⨁⨁◯  MODERATE^a^ | 1156 (7) | 23.21 (12.07 - 44.62) | NA | NA | NA |
|  |  |  | 20.3% increase in mortality. Between 13.4% more and 22.4% more | |  |  |  | NA | |  |
| Tachypnea  Definition:  More than 20-24 bpm | 202 (1) | 1.21 (1.12-1.31) | 7.6% | 9% | ⨁⨁⨁◯  MODERATE^a^ | 518 (7) | 7.51 (1.66-33.91) | 13% | 52.9% | ⨁⨁⨁◯  MODERATE^d^ |
|  |  |  | 1.4% increase in mortality. Between 0.9% more and 1.9% more | |  |  |  | 39.9% increase severe COVID-19 disease. Between 6.9% more and 70.5% more | |  |
| Hypoxemia  Definition:  Low digital saturation (below 90-93%) | 1047 (5) | 5.46 (2.05-14.53) | 2.3% | 9.1% | ⨁⨁⨁◯  MODERATE^a^ | 1331 (5) | 4.69 (1.56-14.09) | NA | NA | NA |
|  |  |  | 6.7% increase in mortality. Between 4.2% more and 7.7% more | |  |  |  | NA | |  |
| Dyspnea  Definition: Dyspnea or shortness of breath | 6613 (28) | 3.45 (2.72-4.38) | 4.9% | 13.8% | ⨁⨁⨁⨁  HIGH | 16803 (78) | 4.23 (3.32-5.38) | 9.3% | 27.8% | ⨁⨁◯◯  LOW^a,d^ |
|  |  |  | 8.9% increase in mortality. Between 7.5% more and 10.2% more | |  |  |  | 18.5 % increase severe COVID-19 disease. Between 15.4% more and 21.3% more | |  |
|  |  |  |  |  |  |  |  |  |  |  |
| Chest pain  Definition: History of any chest pain or discomfort | 1540 (8) | 1.26 (0.65-2.5) | 8.8% | 10.9% | ⨁⨁◯◯  LOW^f^ | 7537 (35) | 1.85 (1.4-2.43) | 12.6% | 20.9% | ⨁⨁◯◯  LOW ^a,d^ |
|  |  |  | 2% increase in mortality. Between 3% less and 9.9% more | |  |  |  | 8.4 % increase severe COVID-19 disease. Between 4.2% more and 12.9% more | |  |
| Tachycardia  Definition:  More than 90-100 bpm | 1269 (2) | 2.61 (1.62-4.22) | 9% | 20.5% | ⨁⨁⨁◯  MODERATE^a^ | 78 (1) | 1.54 (0.31-7.58) | 13% | 18.7% | ⨁◯◯◯  VERY LOW^a,f^ |
|  |  |  | 11.5% increase in mortality. Between 4.8% more and 20.4% more | |  |  |  | 5.7 % increase severe COVID-19 disease. Between 8.6% less and 40.1% more | |  |
| Low blood pressure  Definition  SBP less than 90-100 mmHg | 1269 (2) | 6.7 (3.14-14.33) | 9% | 39.9% | ⨁⨁⨁◯  MODERATE^a^ | 480 (2) | 1.29 (0.72-2.29) | NA | NA | NA |
|  |  |  | 30.9% increase in mortality. Between 14.7% more and 49.6% more | |  |  |  | NA | |  |
| High fever. Definition:More than 39°C | 600 (4) | 1.08 (0.29-3.97) | 9% | 9.6% | ⨁⨁◯◯  LOW ^a,d^ | 4693 (21) | 1.78 (1.17--2.7) | 12.3% | 19.9% | ⨁⨁◯◯  LOW ^a,d^ |
|  |  |  | 0.6% increase in mortality. Between 6.4% less and 17.7% more. | |  |  |  | 7.6% increase severe COVID-19 disease. Between 1.9% more and 14.2% more. | |  |
| Fever. Definition: More than 37.5°C | 6154 (31) | 1.04 (0.77-1.4) | 8.7% | 9.1% | ⨁⨁◯◯  LOW^a,b^ | 20026 (102) | 1.84 (1.54-2.21) | 9.3% | 15.4% | ⨁⨁⨁◯  MODERATE^d^ |
|  |  |  | 0.3% increase in mortality. Between 2.3% less and 2.5% more. | |  |  |  | 6.1% increase severe COVID-19 disease. Between 4.5% more and 7.6% more. | |  |
| Rhinorrhea | 314 (2) | 1.42 (0.34-5.87) | 8.9% | 12.1% | ⨁⨁◯◯  LOW ^a,b^ | 9367 (33) | 0.89 (0.6-1.31) | 13.1% | 11.8% | ⨁⨁◯◯  LOW ^a,d^ |
|  |  |  | 3.3% increase in mortality. Between 6% less and 24.6% more. | |  |  |  | 1.3% decrease severe COVID-19 disease. Between 4.9% less and 3.3% more. | |  |
| Odynophagia | 1608 (6) | 0.57 (0.26-1.25) | 9.2% | 5.4% | ⨁⨁◯◯  LOW ^a,b^ | 14261 (57) | 0.95 (0.74-1.21) | 13.1% | 12.5% | ⨁⨁◯◯  LOW ^a,b^ |
|  |  |  | 3% decrease in mortality. Between 6.7% less and 20% more. | |  |  |  | 0.6% decrease severe COVID-19 disease. Between 2% less and 1.7% more. | |  |
| Conjunctivitis  Definition: conjunctival congestion or conjunctival redness | 0 (0) | NA | NA | NA | NA | 1116 (3) | 7.45 (0.76-73.3) | 12.7% | 51.4% | ⨁◯◯◯  VERY LOW ^a,f^ |
|  |  |  | NA | |  |  |  | 38.7% increase severe COVID-19 disease. Between 2.8% less and 75% more. | |  |
| Cough  Definition: Any cough or dry cough | 5152 (30)) | 0.84 (0.66-1.07) | 10% | 8.5% | ⨁⨁⨁◯  MODERATE^d^ | 19075 (95) | 1.31 (1.17-1.46) | 11.4% | 14.4% | ⨁⨁◯◯  LOW ^g^ |
|  |  |  | 1.5% decrease in mortality. Between 3.6% less and 0.5% more. | |  |  |  | 3% increase severe COVID-19 disease. Between 1.7% more and 4.1% more. | |  |
| Productive cough  Definition: Productive cough or sputum production | 1845 (12) | 1.38 (0.97-1.85) | 8.1% | 10.5% | ⨁⨁◯◯  LOW^a,d^ | 661 (2) | 0.98 (0.4- 2.39) | 13.1% | 12.8% | ⨁⨁◯◯  LOW ^f^ |
|  |  |  | 2.4% increase in mortality. Between 0.2% less and 5% more. | |  |  |  | 0.2% decrease severe COVID-19 disease. Between 8.7% less and 10.5% more. | |  |
| Haemoptysis | 781 (5) | 2.91 (0.74-11.4) | 8.3% | 20.6% | ⨁⨁◯◯  LOW^a,b^ | 3317 (14) | 4.39 (2.18--8.81) | 12.7% | 38.6% | ⨁⨁⨁◯  MODERATE^a^ |
|  |  |  | 12.3% increase in mortality. Between 2.2% less and 34.6% more. | |  |  |  | 25.9% increase severe COVID-19 disease. Between 11.4% more and 42.1% more. | |  |
| Fatigue | 3725 (21) | 1.66 (1.27-2.17) | 7.1% | 11.1% | ⨁⨁◯◯  LOW^a,d^ | 13262 (71) | 1.41 (1.19-1.68) | 11.6% | 15.5% | ⨁⨁⨁◯  MODERATE^d^ |
|  |  |  | 4% increase in mortality. Between 1.9% more and 5.9% more. | |  |  |  | 3.9% increase severe COVID-19 disease. Between 2% more and 5.9% more. | |  |
| Enlarged lymph nodes | 0 (0) | NA | NA | NA | NA | 1099 (1) | 15.62 (0.97-252.5) | 13% | 70% | ⨁◯◯◯  VERY LOW^a,f^ |
|  |  |  | NA | |  |  |  | 56% increase severe COVID-19 disease. Between 0.3% less and 83% more. | |  |
| Myalgia/arthralgia  Definition: myalgia and/or arthralgias | 3436 (18) | 0.96 (0.77-1.23) | 9.1% | 8.7% | ⨁⨁◯◯  LOW^a,b^ | 13814 (61) | 1.29 (1.03-1.61) | 12.5% | 15.6% | ⨁⨁⨁◯  MODERATE^d^ |
|  |  |  | 0.3% decrease in mortality. Between 2% less and 1.8% more. | |  |  |  | 3% increase severe COVID-19 disease. Between 0.3% more and 5.9% more. | |  |
| Rash  Definition:  Any skin rash | 0 (0) | NA | NA | NA | NA | 1099 (1) | 0.84 (0.0001-485.09) | 13% | 11.2% | ⨁◯◯◯  VERY LOW^a,f^ |
|  |  |  | NA | |  |  |  | 1.8% decrease severe COVID-19 disease. Between 13% less and  84% more. | |  |
| Headache | 2249 (11) | 0.8 (0.39-1.64) | 9.2% | 7.5% | ⨁⨁◯◯  LOW^a,b^ | 13698 (61) | 0.89 (0.71-1.12) | 13.1% | 11.8% | ⨁⨁◯◯  LOW^a,b^ |
|  |  |  | 1.7% decrease in mortality. Between 5.7% less and 4.7% more. | |  |  |  | 1.3% decrease severe COVID-19 disease. Between 3.5% less and  1.3 more. | |  |
| Vomits | 1867 (8) | 1.12 (0.5-2.51) | 8.9% | 10% | ⨁⨁◯◯  LOW^a,d^ | 10692 (40) | 1.38 (1.007-1.9) | 12.8% | 16.9% | ⨁⨁◯◯  LOW^a,b^ |
|  |  |  | 1.1% increase in mortality. Between 4.5% less and 9.8% more. | |  |  |  | 4% increase severe COVID-19 disease. Between 0.1% more and 8.8% more. | |  |
| Diarrhea | 4224 (19) | 1.14 (0.86-1.51) | 8.7% | 9.8% | ⨁⨁◯◯  LOW^a,b^ | 16486 (74) | 1.28 (1.1-1.48) | 12.7% | 15.7% | ⨁⨁◯◯  LOW^a,d^ |
|  |  |  | 1.1% increase in mortality. Between 1.2% less and 3.6% more. | |  |  |  | 3% increase severe COVID-19 disease. Between 1.1% more and 4.9% more. | |  |
| Anorexia | 1483 (8) | 2.16 (1.14-4.12) | 7.3% | 14.4% | ⨁⨁⨁◯  MODERATE^c^ | 5495 (26) | 2.86 (2.16-3.84) | 10.4% | 24% | ⨁⨁⨁◯  MODERATE^a^ |
|  |  |  | 7% increase in mortality. Between 1.1% more and 13.1% more. | |  |  |  | 13.6% increase severe COVID-19 disease. Between 9.8% more and 17.5% more. | |  |
| Abdominal pain | 1127 (5) | 1.06 (0.53-2.11) | 9% | 9.5% | ⨁⨁◯◯  LOW^a,b^ | 4896 (22) | 1.95 (1.36-2.79) | 12.7% | 22% | ⨁⨁⨁◯  MODERATE^a^ |
|  |  |  | 5% increase in mortality. Between 4% less and 8% more. | |  |  |  | 9.4% increase severe COVID-19 disease. Between 4% more and 15.8% more. | |  |
| **Laboratory measures (blood or plasma)** | | | | | | | | | | |
| Anemia  Definition: Haemoglobin G/L, ≤ 110-115 | 642 (3) | 3.33 (1.86-5.97) | 7.1% | 19.7% | ⨁⨁◯◯  LOW^a,c^ | 3480 (10) | 1.24 (0.95-1.61) | 12.6% | 15.2% | ⨁⨁◯◯  LOW^a,d^ |
|  |  |  | 12.5% increase in mortality. Between 5.9% more and 19.2% more | |  |  |  | 2.6% increase severe COVID-19 disease. Between 0.6% less and 6% more | |  |
| High WBC  Definition: greater than 10.0 x 10^9^/L | 2870 (10) | 4.06 (2.7-6.12) | 7.8% | 24.7% | ⨁⨁⨁◯  MODERATE^d^ | 9331 (32) | 4.67 (3.17-6.88) | 11.2% | 35.6% | ⨁⨁⨁⨁  HIGH |
|  |  |  | 16.9% increase in mortality. Between 11% more and 23.3% more | |  |  |  | 24.3% increase severe COVID-19 disease. Between 17.3% more and 31.2% more | |  |
| High Neutrophil count  Definition: greater than 6.3 x 10^9^/L | 727 (2) | 6.78 (3.07-14.97) | 5.2% | 23% | ⨁⨁◯◯  LOW^a,c^ | 4945 (16) | 5.66 (3.71-8.63) | 9% | 31% | ⨁⨁⨁◯  MODERATE^a^ |
|  |  |  | 17.8% increase in mortality. Between 10% more and 23% more. | |  |  |  | 22% increase severe COVID-19 disease. Between 17% more and 27% more. | |  |
| Increase in neutrophil count  Definition: 10 x 10^9^ U/L increase | 1321 (6) | 1.48 (0.43-5.23) | 9% | 12.8% | ⨁⨁◯◯  LOW^g^ | 796 (5) | 7.30 (1.48-41.08) | 13% | 52.2% | ⨁⨁◯◯  LOW^g^ |
|  |  |  | 3.8% increase in mortality. Between 4.9% less and 25.1% more | |  |  |  | 39.2% increase severe COVID-19 disease. Between 5.1% more and 73% more | |  |
| Low neutrophil count  Definition: Less than 1.8 x 10^9^/L | 727 (2) | 0.29 (0.027-3.27) | 9.8% | 3% | ⨁⨁◯◯  LOW^a,b^ | 4649 (15) | 0.54 (0.35-0.81) | 14% | 8% | ⨁⨁◯◯  LOW^a,d^ |
|  |  |  | 6.8% decrease in mortality. Between 1% less and 12% more. | |  |  |  | 6% decrease severe COVID-19 disease. Between 9% less and 2.3% less. | |  |
| Leukopenia  Definition: Less than 3.5-4 x 10^9^/L | 3313 (10) | 0.64 (0.33-1.22) | 9.8% | 6.5% | ⨁⨁◯◯  LOW ^b,d^ | 8830 (33) | 0.7 (0.53-0.93) | 14% | 10.2% | ⨁⨁◯◯  LOW^a,d^ |
|  |  |  | 3.3% decrease in mortality. Between 6.9% less and 1.7% more | |  |  |  | 3.8% decrease severe COVID-19 disease. Between 6.4% less and 0.8% less | |  |
| Low Lymphocyte count  Definition:  Less than 0.8-1.5x 10^9^/L | 3799 (13) | 2.64 (1.67-4.18) | 5.4% | 12.5% | ⨁⨁◯◯  LOW ^g^ | 10678 (47) | 3.47 (2.91-4.14) | 7.1% | 18.6% | ⨁⨁⨁⨁  HIGH |
|  |  |  | 7.1% increase in mortality. Between 4% more and 9.5% more | |  |  |  | 11.5% increase severe COVID-19 disease. Between 10.3% more and 12.6% more | |  |
| Decrease Lymphocyte count  Definition:  per 1 x 10^9^ U/L decrease | 544(3) | 3.57 (2-6.67) | 9% | 26.1% | ⨁⨁⨁◯  MODERATE^d^ | 1909 (7) | 2.28 (1.21-4.30) | 13% | 25.4% | ⨁⨁⨁◯  MODERATE^d^ |
|  |  |  | 17.1% increase in mortality. Between 7.5% more and 30.7% more | |  |  |  | 12.4% increase severe COVID-19 disease. Between 2.3% more and 26.1% more | |  |
| Low platelet count  Definition: Less than 100-150 x 10^9^/L | 3676 (10) | 5.43 (2.55-11.56) | 5% | 19.3% | ⨁⨁⨁⨁  HIGH | 8081 (32) | 1.93 (1.52-2.46) | 11.1% | 19.2% | ⨁⨁◯◯  LOW^a,d^ |
|  |  |  | 14.3% increase in mortality. Between 8.3% more and 18.6% more | |  |  |  | 8% increase severe COVID-19 disease. Between 5% more and 11.1% more | |  |
| High creatinine  Definition: More than 1.5 mg% | 5241 (11) | 3.91 (2.89-5.28) | 5.8% | 17.9% | ⨁⨁⨁◯  MODERATE^d^ | 6978 (24) | 2.95 (2.16--4.02) | 11.9% | 28% | ⨁⨁◯◯  LOW^a,d^ |
|  |  |  | 12.1% increase in mortality. Between 9.5% more and 14.5% more | |  |  |  | 16.1% increase severe COVID-19 disease. Between 10.8% more and 21.8% more | |  |
| Increase in creatinine  Definition: per 1 mg% increase | 1508 (9) | 1.14 (1.02-1.28) | 9% | 10.1% | ⨁⨁⨁◯  MODERATE^b^ | 1116 (4) | 1.89 (0.87-4.10) | 13% | 22% | ⨁⨁⨁◯  MODERATE^b^ |
|  |  |  | 1.1% increase in mortality. Between 0.2% more and 2.3% more. | |  |  |  | 9% increase severe COVID-19 disease. Between 1.5% less and 25% more. | |  |
| Acute kidney injury  Definition: Increase in serum creatinine to 1.5 times baseline or more | 3491 (15) | 8.77 (4.32-17.8) | 5.4% | 28.1% | ⨁⨁◯◯  LOW^a,d^ | 3381 (19) | 10.83 (5.75-20.37) | 9.5% | 45% | ⨁⨁⨁◯  MODERATE^a^ |
|  |  |  | 22.7% increase in mortality. Between 15.3% more and 28% more | |  |  |  | 26% increase severe COVID-19 disease. Between 19.6% more and 31.7% more | |  |
| Decrease in glomerular filtration rate  Definition: 10 mL/min decrease | 0 (0) | NA | NA | NA | NA | 668(2) | 3.11 (0.25-44.01) | 13% | 31.7% | ⨁⨁◯◯  LOW ^f^ |
|  |  |  | NA | |  |  |  | 18.7% decrease severe COVID-19 disease. Between 9.4% less and 73.8% more. | |  |
| Increase in blood urea  Definition: per 10 mmol/L increase | 477 (3) | 2.64 (1.32-5.27) | 9% | 20.7% | ⨁⨁◯◯  LOW^a,c^ | 248 (2) | 17.32 (0.54-581.59) | 13% | 72.1% | ⨁◯◯◯  VERY LOW ^a,f^ |
|  |  |  | 11.7% increase in mortality. Between 2.6% more and 25.3% more | |  |  |  | 59.1% increase severe COVID-19 disease. Between 5.6% less and 85.9% more | |  |
| High BUN  Definition: mmol/L, > 5.2-9.5 | 1258 (2) | 10.56 (6.76-16.48) | 5.2% | 29.6% | ⨁⨁◯◯  LOW^a,c,e^ | 3890 (10) | 3.66 (2.82-4.74) | 11.1% | 30.2% | ⨁⨁⨁◯  MODERATE^a^ |
|  |  |  | 24.4% increase in mortality. Between 20.2% more and 27.7% more | |  |  |  | 19.1% increase severe COVID-19 disease. Between 14.8% more and 23.4% more | |  |
| Increase in blood Cystatin C  Definition: per 1 mg/L increase | 84 (1) | 2.54 (1.44-4.94) | 9% | 20.1% | ⨁◯◯◯  VERY LOW ^a,f^ | 201 (1) | 2.08 (1.44-3.16) | 13% | 23.7% | ⨁⨁◯◯  LOW^a,c^ |
|  |  |  | 11.1% increase in mortality. Between 3.5% more and 24% more. | |  |  |  | 10.7% increase severe COVID-19 disease. Between 4.7% more and 19.1% more. | |  |
| High LDH  Definition:  More than 240-250 U/L | 1440 (6) | 4.09 (1.18-14.17) | 4.7% | 15.2% | ⨁⨁⨁◯  MODERATE^d^ | 7955 (26) | 4.48 (3.21-6.25) | 7.8% | 23.9% | ⨁⨁⨁◯  MODERATE^d^ |
|  |  |  | 10.4% increase in mortality. Between 1.4% more and 15.3% more | |  |  |  | 16.2% increase severe COVID-19 disease. Between 13.1% more and 18.8% more | |  |
| Increase in LDH  Definition:  per 100 U/L increase | 1869 (10) | 1.31 (1.20-1.43) | 9% | 11.5% | ⨁⨁⨁⨁  HIGH | 1044 (7) | 1.91 (1.34-2.73) | 13% | 22.2% | ⨁⨁⨁◯  MODERATE^d^ |
|  |  |  | 2.5% increase in mortality. Between 1.6% more and 3.4% more | |  |  |  | 9.2% increase severe COVID-19 disease. Between 3.6% more and 16% more | |  |
| High CK  Definition:  More than 185-200 U/L | 407 (3) | 1.35 (0.58-3.14) | 8.8% | 11.5% | ⨁⨁◯◯  LOW^a,b^ | 3292 (13) | 3.1 (2.32-4.16) | 11.5% | 28.1% | ⨁⨁⨁◯  MODERATE^a^ |
|  |  |  | 2.7% increase in mortality. Between 3.7% less and 13% more | |  |  |  | 16.5% increase severe COVID-19 disease. Between 11.7% more and 21.6% more | |  |
| Myocardial injury  Definition:  Reported as myocardial injury or as increase in blood troponins | 3855 (21) | 10.89 (5.39-22.04) | 3.5% | 20.4% | ⨁⨁⨁◯  MODERATE^d^ | 3627 (20) | 10 (6.84-14.62) | 11.1% | 51.3% | ⨁⨁⨁⨁  HIGH |
|  |  |  | 16.9% increase in mortality. Between 13.4% more and 19% more | |  |  |  | 40.2% increase severe COVID-19 disease. Between 33.1% more and 46.4% more | |  |
| High CK-MB  More than 25 U/L | 0 (0) | NA | NA | NA | NA | 1663 (7) | 5.73 (3.71-8.85) | 10.7% | 37.8% | ⨁⨁⨁◯  MODERATE^a^ |
|  |  |  | NA | |  |  |  | 27.2% increase severe COVID-19 disease. Between 19.7% more and 34% more | |  |
| Increase in CK-MB. Definition per 60 U/L increase | 564 (4) | 1.33 (0.79-2.25) | 9% | 11.6% | ⨁⨁◯◯  LOW ^b,d^ | 289 (3) | 1.71 (0.85-3.42) | 13% | 20.4% | ⨁⨁◯◯  LOW^a,d^ |
|  |  |  | 2.6% increase in mortality. Between 1.7% less and 9.2% more. | |  |  |  | 7.4% increase severe COVID-19 disease. Between 1.7% less and 9.2% more. | |  |
| High BNP: More than 500-900 pg/mL | 1283 (6) | 3.27 (1.24-8.63) | 7% | 19% | ⨁⨁◯◯  LOW^a,d^ | 1086 (1) | 4.99 (3.2-7.77) | 9.4% | 30.9% | ⨁⨁⨁◯  MODERATE^a^ |
|  |  |  | 12% increase in mortality. Between 1.9% more and 21.9% more. | |  |  |  | 21.5% increase severe COVID-19 disease. Between 15.5% more and 26.7% more. | |  |
| High D-dimer  Definition:  More than 500-1000 ng/ml | 4361 (17) | 4.81 (3.15-7.34) | 4.3% | 15.6% | ⨁⨁⨁◯  MODERATE^d^ | 6356 (24) | 3.27 (2.46-4.36) | 8.2% | 20.7% | ⨁⨁⨁◯  MODERATE^d^ |
|  |  |  | 11.2% increase in mortality. Between 8.8% more and 13.1% more. | |  |  |  | 12.5% increase severe COVID-19 disease. Between 9.8% more and 14.8% more. | |  |
| Increase in D-dimer  Definition:per  100 ng/mL increase | 1047 (5) | 1.18 (1.06-1.31) | 9% | 10.4% | ⨁⨁⨁◯  MODERATE^b^ | 1245 (4) | 1.54 (1.29-1.84) | 13% | 18.7% | ⨁⨁⨁⨁  HIGH |
|  |  |  | 1.4% increase in mortality. Between 0.5% more and 2.5% more. | |  |  |  | 5.7% increase severe COVID-19 disease. Between 3.1% more and 8.6% more. | |  |
| High Fibrinogen degradation products  Definition: More than 4.1 g/L | 0 (0) | NA | NA | NA | NA | 240 (1) | 2.05 (1.19-3.55) | 13% | 23.4% | ⨁⨁◯◯  LOW^a,c^ |
|  |  |  | NA | |  |  |  | 10.4% increase severe COVID-19 disease. Between 2.1% more and 21.7% more. | |  |
| Prolonged PT  Definition: more than 13.2-15 sec. | 318 (2) | 7 (1.51-32.41) | 6.3% | 28.6 | ⨁⨁◯◯  LOW ^f^ | 2798 (6) | 2.2 (1.36-3.54) | 11.8% | 22.5% | ⨁⨁◯◯  LOW ^a,d^ |
|  |  |  | 22.3% increase in mortality. Between 3.8% more and 35.3% more. | |  |  |  | 10.7% increase severe COVID-19 disease. Between 3.8% more and 18.3% more. | |  |
| Increase in PT  Definition: per 10 seconds increase | 548 (4) | 3.39 (1.18-9.31) | 9% | 25.1% | ⨁⨁◯◯  LOW ^a,e^ | 248 (2) | 1.89 (1.5-2.38) | 13% | 22% | ⨁⨁◯◯  LOW ^a,c^ |
|  |  |  | 16.1% increase in mortality. Between 15% more and 38.9% more. | |  |  |  | 9% increase severe COVID-19 disease. Between 5.3% more and 13.2% more. | |  |
| Prolonged APTT time  Definition: more than 35-45 sec | 0 (0) | NA | NA | NA | NA | 2673 (6) | 1.41 (0.79-2.52) | 12.3% | 16.4% | ⨁◯◯◯  VERY LOW  ^a,g^ |
|  |  |  | NA | |  |  |  | 4.2% increase severe COVID-19 disease. Between 2.5% less and 12% more. | |  |
| Increase in APTT time  Definition: per 10 seconds increase | 530 (3) | 0.90 (0.66-1.22) | 9% | 8.2% | ⨁⨁◯◯  LOW ^a,b^ | 248 (2) | 1.13 (0.78-1.63) | 9% | 10.1% | ⨁◯◯◯  VERY LOW  ^a,c,d^ |
|  |  |  | 0.8% decrease in mortality. Between 2.8% less and 1.8% more | |  |  |  | 1.1% increase severe COVID-19 disease. Between 1.8% less and 4.9% more. | |  |
| High ferritin  Definition:  More than 300-500 ng/mL | 848 (4) | 5.71 (2.36-13.82) | 4.2% | 16.7% | ⨁⨁◯◯  LOW ^a,e^ | 1035 (5) | 3.81 (2.43-5.95) | 7.7% | 21.5% | ⨁⨁◯◯  LOW ^a,e^ |
|  |  |  | 12.5% increase in mortality. Between 7% more and 15.8% more | |  |  |  | 13.8% increase severe COVID-19 disease. Between 9.7% more and 17% more. | |  |
| High CRP  Definition: More than 1-100 mg/l | 2107 (8) | 6.6 (3.36-12.99) | 2.3% | 10.3% | ⨁⨁⨁◯  MODERATE^d^ | 9094 (37) | 4.5 (3.1-6.23) | 6.3% | 19.5% | ⨁⨁⨁⨁  HIGH |
|  |  |  | 7.9% increase in mortality. Between 6.4% more and 8.7% more | |  |  |  | 13.2% increase severe COVID-19 disease. Between 10.8% more and 14.9% more | |  |
| Increase in CRP  Definition: per 10 mg/L increase | 1306 (7) | 1.12 (0.95-1.32) | 9% | 10% | ⨁⨁⨁◯  MODERATE^b^ | 2259 (9) | 1.11 (1.00-1.22) | 13% | 14.2% | ⨁⨁⨁◯  MODERATE^b^ |
|  |  |  | 1% increase in mortality. Between 0.4% less and 2.6% more. | |  |  |  | 1.2% increase severe COVID-19 disease. Between 0% more and 2.4% more. | |  |
| High ESR  Definition More than 10-20 mm/H | 628 (3) | 0.89 (0.54-1.45) | 9.7% | 8.7% | ⨁⨁◯◯  LOW^a,b^ | 2557 (12) | 3.08 (2.04-4.65) | 6.6% | 15.6% | ⨁⨁⨁◯  MODERATE^a^ |
|  |  |  | 1% decrease in mortality. Between 5.6% less and 2.8% more. | |  |  |  | 9.4% increase severe COVID-19 disease. Between 6.7% more and 11.3% more. | |  |
| Increase in ESR  Definition: per 10 mm/H increase | 0 (0) | NA | NA | NA | NA | 450 (2) | 1.005 (0.99-1.01) | 13% | 13.6% | ⨁⨁◯◯  LOW^a,b^ |
|  |  |  | NA | |  |  |  | 0.6% increase severe COVID-19 disease. Between 0.6% less and 1.9% more. | |  |
| High interleukin-6  Definition:  More than 5-20 pg/ml | 436 (4) | 1.31 (0.14-12.27) | 8.1% | 10.3% | ⨁⨁◯◯  LOW^a,b^ | 1211 (7) | 7.36 (2.97-18.27) | 6.5% | 26.2% | ⨁⨁⨁◯  MODERATE^a^ |
|  |  |  | 2.2% increase in mortality. Between 11.6% less and 15% more | |  |  |  | 19.7% increase severe COVID-19 disease. Between 12.2% more and 23.9% more | |  |
| Increase in interleukin-6  Definition:  per 10 pg/mL increase | 484 (3) | 1.09 (0.94-1.27) | 9% | 9.2% | ⨁⨁⨁◯  MODERATE^b^ | 499 (2) | 1.34 (1.09-1.64) | 13% | 16.7% | ⨁⨁◯◯  LOW^a,c^ |
|  |  |  | 0.2% increase in mortality. Between 0% more and 5% more | |  |  |  | 3.7% increase severe COVID-19 disease. Between 1% more and 6.7% more. | |  |
| High procalcitonin  Definition:  More than 0.01-05 ng/ml | 4735 (10) | 12.42 (7.18-21.5) | 6.3% | 38.5% | ⨁⨁⨁◯  MODERATE^c^ | 7923 (28) | 5.13 (3.16-8.35) | 10.6% | 35.4% | ⨁⨁◯◯  LOW^a,d^ |
|  |  |  | 32.3% increase in mortality. Between 25% more and 38.1% more. | |  |  |  | 24.8% increase severe COVID-19 disease. Between 16.7% more and 32.3% more | |  |
| Increase in procalcitonin  Definition:  per 0.5 ng/ml increase | 470 (3) | 1.69 (1.40-2.10) | 9% | 14.3% | ⨁⨁⨁⨁  HIGH | 0 (0) | NA | NA | NA | NA |
|  |  |  | 5.3% increase in mortality. Between 3.2% more and 8.2% more. | |  |  |  | NA | |  |
| Increase in cholinesterase  Definition: per 1 x 10^3 U/L increase | 84 (1) | 0.79 (0.65-0.96) | 9% | 7.2% | ⨁◯◯◯  VERY LOW  ^a,f^ | 201 (1) | 0.77 (0.67-0.87) | 13% | 10.3% | ⨁⨁◯◯  LOW^a,c^ |
|  |  |  | 1.8% decrease in mortality. Between 3% less and 0.3% less. | |  |  |  | 2.7% decrease severe COVID-19 disease. Between 3.9% less and 1.5% ess | |  |
| High total bilirubin  Definition: More than17-21pg/ml | 2715 (3) | 3.03 (1.87-4.92) | 8.1% | 20.7% | ⨁⨁◯◯  LOW^a,c^ | 5098 (14) | 2.94 (2.18--3.97) | 12.5% | 29.3% | ⨁⨁⨁◯  MODERATE^a^ |
|  |  |  | 12.6% increase in mortality. Between 6.3% more and 19.9% more. | |  |  |  | 16.8% increase severe COVID-19 disease. Between 11.3% more and 22.9% more | |  |
| Increase in total bilirubin  Definition: per 5 μM increase | 710 (4) | 1.47 (1.28-1.69) | 9% | 12.7% | ⨁⨁◯◯  LOW^a,c^ | 201 (1) | 1.40 (1.16-1.69) | 13% | 17.3% | ⨁⨁◯◯  LOW^a,c^ |
|  |  |  | 3.7% increase in mortality. Between 2.2% more and 5.3% more. | |  |  |  | 4.3% increase severe COVID-19 disease. Between 1.8% more and 7.1% more | |  |
| High AST level  Definition: More than 32-40 U/l | 2969 (7) | 3.5 (1.59-7.71) | 6% | 17.1% | ⨁⨁⨁◯  MODERATE^d^ | 9179 (32) | 3.41 (2.7-4.3) | 9.9% | 25.8% | ⨁⨁⨁◯  MODERATE^a^ |
|  |  |  | 11.1% increase in mortality. Between 4% more and 16.8% more | |  |  |  | 15.8% increase severe COVID-19 disease. Between 12.7% more and 18.8% more | |  |
| Increase in AST level  Definition: per 50 U/L increase | 1161 (6) | 1.37 (0.95-1.97) | 9% | 11.9% | ⨁⨁◯◯  LOW^a,b^ | 747 (5) | 3.29 (0.89-12.20) | 13% | 33.5% | ⨁⨁◯◯  LOW^a,b^ |
|  |  |  | 2.9% increase in mortality. Between 0.4% less and 7.3% more | |  |  |  | 20.5% increase severe COVID-19 disease. Between 9.1% less and 73.1% more | |  |
| High ALT level  Definition: More than 35-50 U/L | 1197 (7) | 2.45 (1.07-5.59) | 7.1% | 15.4% | ⨁⨁◯◯  LOW^a,d^ | 8776 (27) | 2.44 (1.85-3.21) | 10.9% | 22.5% | ⨁⨁◯◯  LOW^a,d^ |
|  |  |  | 8.3% increase in mortality. Between 0.6% more and 15.9% more | |  |  |  | 11.5% increase severe COVID-19 disease. Between 7.7% more and 15.3% more | |  |
| Increase in ALT level  Definition: per 50 U/L increase | 1294 (6) | 1.23 (0.84-1.80) | 9% | 10.9% | ⨁⨁◯◯  LOW^a,b^ | 592 (4) | 1.69 (0.87-3.26) | 13% | 20.1% | ⨁◯◯◯  VERY LOW  ^a,c,d^ |
|  |  |  | 1.9% increase in mortality. Between 1.3% less and 6.1% more | |  |  |  | 7.1% increase severe COVID-19 disease. Between 1.5% less and 19.7% more | |  |
| High globulin  Definition: 10 g/L increase | 84 (1) | 1.83 (1.01-12.1) | 9% | 15.3% | ⨁◯◯◯  VERY LOW  ^a,f^ | 201 (1) | 3.5 (1.66-8.98) | 13% | 34.3% | ⨁⨁◯◯  LOW^a,c^ |
|  |  |  | 6.3% increase in mortality. Between 0.1% more and 45% more | |  |  |  | 21.3% increase severe COVID-19 disease. Between 6.9% more and 44.3% more | |  |
| High pre-albumin  Definition: 1 mg/ L increase | 84 (1) | 1 (0.99-1) | 9% | 9% | ⨁◯◯◯  VERY LOW  ^a,f^ | 201 (1) | 0.99 (0.97-0.99) | 13% | 12.9% | ⨁⨁◯◯  LOW^a,c^ |
|  |  |  | 0% incrless and 0% moreease in mortality. Between 0.1% | |  |  |  | 0.1% decrease severe COVID-19 disease. Between 0.3% less and 0.1% less | |  |
| Low albumin  Definition: less than 35-40 g/l | 1972 (5) | 6.05 (3.89-9.61) | 3.2% | 13.2% | ⨁⨁◯◯  LOW^a,e^ | 4958 (14) | 5.41 (3.21-9.1) | 6.9% | 23.6% | ⨁⨁⨁⨁  HIGH |
|  |  |  | 10% increase in mortality. Between 8.4% more and 11.1% more. | |  |  |  | 16.7% increase severe COVID-19 disease. Between 12.5% more and 19.7% more. | |  |
|  |  |  |  |  |  |  |  |  |  |  |
| Decrease in albumin: Definition: 20 g/L decrease | 336 (3) | 1.53 (1.32-1.78) | 9% | 13.2% | ⨁⨁⨁◯  MODERATE^c^ | 1266 (5) | 1.11 (1.01-1.21) | 13% | 14.2% | ⨁⨁⨁◯  MODERATE^b^ |
|  |  |  | 4.2% increase in mortality. Between 2.5% more and 6% more. | |  |  |  | 1.2% increase severe COVID-19 disease. Between 0.1% more and 2.3% more. | |  |
| High glucose  Definition: More than 6 mmol/l | 600 (2) | 1.67 (1.01-2.79) | 7.6% | 11.9% | ⨁⨁◯◯  LOW^f^ | 1044 (3) | 3.01 (2.28-4) | 8.6% | 20.5% | ⨁◯◯◯  VERY LOW  ^a,c,e^ |
|  |  |  | 4.3% increase in mortality. Between 0.1% more and 8.6% more. | |  |  |  | 11.9% increase severe COVID-19 disease. Between 9.2% more and 14.4% more. | |  |
| Increase in glucose  Definition: 20 mmol/L increase | 218 (2) | 1.40 (0.17-11.30) | 9% | 12.2% | ⨁⨁◯◯  LOW^f^ | 201 (1) | 23.11 (1.22-101.72) | 13% | 77.5% | ⨁◯◯◯  VERY LOW  ^a,f^ |
|  |  |  | 3.2% increase in mortality. Between 7.3% less and 43.8% more. | |  |  |  | 64.5% increase severe COVID-19 disease. Between 2.4% more and 80.8% more. | |  |
| High lactate: Definition More than  1.5-2.2 mmol/L | 1078 (1) | 3.66 (2.26-5.94) | 7.3% | 21.7% | ⨁⨁⨁◯  MODERATE^a^ | 812 (3) | 3.74 (0.69-20.16) | 12.1% | 33.3% | ⨁◯◯◯  VERY LOW ^a,b,d^ |
|  |  |  | 14.3% increase in mortality. Between 8.3% more and 20.6% more. | |  |  |  | 21.2% increase severe COVID-19 disease. Between 3.7% less and 51.4% more. | |  |
| Increase IN lactate:  Definition: 5 mmol/L increase | 431 (2) | 1.05 (0.76-1.44) | 9% | 9.4% | ⨁⨁◯◯  LOW^a,b^ | 344 (2) | 2.09 (0.47-9.4) | 13% | 23.8% | ⨁◯◯◯  VERY LOW ^a,f^ |
|  |  |  | 4% increase in mortality. Between 2% less and 3.4% more. | |  |  |  | 10.8% decrease severe COVID-19 disease. Between 6.5% less and 4.5% more. | |  |
| High alfa-HBDH. Definition: 100 U/L unit increase | 84 (1) | 1.55 (1.19-2.06) | 9% | 13.3% | ⨁◯◯◯  VERY LOW ^a,f^ | 201 (1) | 2.18 (1.78-2.7) | 13% | 24.6% | ⨁⨁◯◯  LOW^a,c^ |
|  |  |  | 4.3% increase in mortality. Between 1.5% more and 7.9% more. | |  |  |  | 11.6% increase severe COVID-19 disease. Between 8% more and 15.7% more. | |  |
| Increase LDL  Definition: 1 mM unit increase | 84 (1) | 0.79 (0.45-1.49) | 9% | 7.2% | ⨁◯◯◯  VERY LOW ^a,f^ | 201 (1) | 0.56 (0.37-0.85) | 13% | 7.7% | ⨁⨁◯◯  LOW^a,c^ |
|  |  |  | 1.8% decrease in mortality. Between 4.7% less and 3.8% more. | |  |  |  | 5% decrease severe COVID-19 disease. Between 7.8% less and 1.7% less | |  |
| High triglycerides  Definition: More than 1.7 mg/L | 0 (0) | NA | NA | NA | NA | 291 (1) | 0.73 (0.27-1.96) | 13.4% | 10.1% | ⨁⨁◯◯  LOW^a,b^ |
|  |  |  | NA | |  |  |  | 3% decrease in mortality. Between 10% less and 8.8% more. | |  |
| **Radiological signs** | | | | | | | | | | |
| Any abnormal Radiologic finding  Definition:  Any lung parenchyma abnormality on X ray or CT assessment | 403 (1) | 1.49 (0.55-4.04) | 7.6% | 10.8% | ⨁◯◯◯  VERY LOW ^a,b, e^ | 2556 (8) | 2.31 (1.49-3.58) | 9.1% | 17.9% | ⨁⨁◯◯  LOW ^,a, e^ |
|  |  |  | 3.2% increase in mortality. Between 4.7% less and 9.9% more. | |  |  |  | 8.8% increase severe COVID-19 disease. Between 4.4% more and 12.5% more. | |  |
| Interstitial pattern  Definition: X ray or CT assessment | 27 (1) | 0.63 (0.12-3.22) | 10.9% | 7.1% | ⨁◯◯◯  VERY LOW ^a,f^ | 2840 (14) | 3.66 (1.8-7.41) | 8.1% | 22% | ⨁⨁◯◯  LOW ^,a, d^ |
|  |  |  | 3.8% decrease in mortality. Between 3.8% less and 8.2% more. | |  |  |  |  |  |  |
|  |  |  |  |  |  |  |  | 14% increase severe COVID-19 disease. Between 6.7% more and 19.2% more. | |  |
| Ground glass opacity  Definition: CT assessment | 1303 (6) | 0.73 (0.32-1.71) | 10.7% | 8% | ⨁⨁◯◯  LOW ^,a, b^ | 7604 (32) | 1.52 (1.08-2.13) | 10.4% | 14.8% | ⨁⨁◯◯  LOW ^,a, b^ |
|  |  |  | 2.7% decrease in mortality. Between 10% less and 4% more. | |  |  |  | 4.4% increase severe COVID-19 disease. Between 0.9% more and 7.4% more. | |  |
| Crazy paving pattern  Definition: CT assessment | 0 (0) | NA | NA | NA | NA | 1415 (11) | 2.67 (1.84-3.87) | 10.3% | 22.5% | ⨁⨁◯◯  LOW ^,a, d^ |
|  |  |  | NA | |  |  |  | 12.3% increase severe COVID-19 disease. Between 7.5% more and 16.8% more. | |  |
| Consolidation pattern  Definition: X ray or CT assessment | 795 (4) | 1.93 (1.31-2.84) | 7.5% | 13% | ⨁⨁◯◯  LOW^a,b^ | 6133 (27) | 2.46 (1.54-3.93) | 11.2% | 23.2% | ⨁⨁⨁◯  MODERATE^d^ |
|  |  |  | 5.8% increase in mortality. Between 2.3% more and 9.4% more. | |  |  |  | 12% increase severe COVID-19 disease. Between 5.4% more and 18.8% more. | |  |
| Enlarged lymph nodes  Definition: X ray or CT assesment | 342 (2) | 0.47 (0.11-2.01) | 9.4% | 4.6% | ⨁◯◯◯  VERY LOW ^a,e,f^ | 1984 (10) | 2.09 (1.36-3.2) | 12.7% | 23.3% | ⨁⨁◯◯  LOW^a,d^ |
|  |  |  | 4.8% decrease in mortality. Between 8.5% less and 7.1% more. | |  |  |  | 10.6% increase severe COVID-19 disease. Between 4% more and 1.9% more. | |  |
| Pleural effusion  Definition: X ray or CT assessment | 820 (5) | 1.38 (0.63-3.06) | 8.8% | 11.7% | ⨁⨁◯◯  LOW^a,b^ | 5289 (23) | 3.31 (2.03-5.38) | 12.5% | 32% | ⨁⨁⨁◯  MODERATE^d^ |
|  |  |  | 3% increase in mortality. Between 3% less and 13% more. | |  |  |  | 19% increase severe COVID-19 disease. Between 10% more and 30% more. | |  |
| Bilateral compromise  Definition: X ray or CT assessment | 1954 (10) | 2.26 (1.29-4) | 7% | 14% | ⨁⨁◯◯  LOW^a,d^ | 8349 (38) | 2.99 (1.83-4.87) | 7.7% | 18.3% | ⨁⨁◯◯  LOW^a,d^ |
|  |  |  | 7% increase in mortality. Between 2.2% more and 11.8% more. | |  |  |  | 10.6% increase severe COVID-19 disease. Between 6.4% more and 13.7% more. | |  |
| **Others** | | | | | | | | | | |
| High APACHE score  Definition:  More than 8 | 148 (2) | 1.1 (1.02-1.19) | 9% | 9.8% | ⨁⨁◯◯  LOW^a,f^ | 107 (2) | 1.4 (0.72-2.73) | 13% | 17.3% | ⨁◯◯◯  VERY LOW ^a,c,d^ |
|  |  |  | 0.8% increase in mortality. Between 0.2% more and 1.5% more. | |  |  |  | 4.3% increase severe COVID-19 disease. Between 3.3% more and 16% more. | |  |
| High SOFA score  Definition:  More than 2 | 585 (3) | 1.97 (1.22-3.2) | 9% | 16.3% | ⨁⨁⨁◯  MODERATE^d^ | 92 (2) | 21.31 (6.26-72.6) | 13% | 76.1% | ⨁⨁◯◯  LOW^a,c^ |
|  |  |  | 7.3% increase in mortality. Between 1.8% more and 15% more. | |  |  |  | 63% increase severe COVID-19 disease. Between 35.3% more and 78.6% more. | |  |
| High qSOFA score  Definition:  More than 2 | 191 (1) | 12 (5.06-28.43) | 9% | 54.3% | ⨁⨁◯◯  LOW | 0 (0) | NA | NA | NA | NA |
|  |  |  | 45.3% increase in mortality. Between 24.4% more and 64.8% more. | |  |  |  | NA | |  |

**Glossary**

ALT: Alanine aminotransferase

APTT: activated partial thromboplastin time

APACHE: Acute Physiology And Chronic Health Evaluation II

AST: Aspartate aminotransferase

BUN: Blood urea nitrogen

FDP: Fibrin Degradation Product

NA: Not applicable

PT: prothrombin time

qSOFA: quick sepsis related organ failure assessment

SOFA: The sequential organ failure assessment score

NA: Not applicable, either because there is no information or because the addressed variable does not represent a potential prognostic factor in that clinical scenario.

**Explanations**

1. Risk of bias due to study limitations (unadjusted estimates, inappropriate prognostic factor or outcome assessment, inappropriate population inclusion criteria, study attrition)
2. Imprecision: confidence interval includes significant and/or non-significant risk increase
3. Imprecision due to fragility: less than 200 events
4. Inconsistency: unexplained visual heterogeneity
5. Risk of selective reporting: most of the pooled estimate weight from studies that performed multivariable analysis but did not report adjusted estimates.
6. Very serious imprecision: very wide confidence interval
7. Very serious inconsistency: very important unexplained visual heterogeneity
