# Supplementary material for: Prognostic factors for severity and mortality in patients infected with COVID-19: A systematic review
Source: PLoS One. 2020 Nov 17;15(11):e0241955. doi: 10.1371/journal.pone.0241955 (PMC7671522; doi:10.1371/journal.pone.0241955)
Supplement: S1 Text — This file contains additional details on methods. (DOCX) [file pone.0241955.s004.docx]

**S1 text. Supplementary methods**

**Search strategy**

*Search strategy for Pubmed/MEDLINE*

Search date: 29.04.2020

#1 coronavir*

#2 coronovirus*

#3 "corona virus"

#4 "virus corona"

#5 "corono virus"

#6 "virus corono"

#7 hcov*

#8 "covid-19"

#9 covid19*

#10 "covid 19"

#11 “2019-nCoV“

#12 cv19*

#13 "cv-19"

#14 "cv 19"

#15 "n-cov"

#16 ncov*

#17 "sars-cov-2"

#18 wuhan* AND (virus OR viruses OR viral)

#19 covid* AND (virus OR viruses OR viral)

#20 "sars-cov"

#21 "sars cov"

#22 "sars-coronavirus"

#23 "severe acute respiratory syndrome"

#24 "mers-cov"

#25 "mers cov"

#26 "middle east respiratory syndrome"

#27 "middle-east respiratory syndrome"

#28 "covid-19-related"

#29 "SARS-CoV-2-related"

#30 "SARS-CoV2-related"

#31 "2019-nCoV-related"

#32 "cv-19-related"

#33 "n-cov-related"

#34 #1 OR #2 OR #3 OR #4 OR #5 OR #6 OR #7 OR #8 OR #9 OR #10 OR #11 OR #12 OR #13 OR #14 OR #15 OR #16 OR #17 OR #18 OR #19 OR #20 OR #21 OR #22 OR #23 OR #24 OR #25 OR #26 OR #27 OR #28 OR #29 OR #30 OR #31 OR #32 OR #33

#35 incidence[MeSH:noexp]

#36 mortality[MeSH Terms]

#37 follow up studies[MeSH:noexp]

#38 prognos*[Text Word]

#39 predict*[Text Word]

#40 course*[Text Word]

#41 #35 OR #36 OR #37 OR #38 OR #39 OR #40

#42 ("2019/10/01"[Date - Publication] : "3000"[Date - Publication]

#43 #34 AND #41 AND #42

*Search strategy for EMBASE (Elsevier)*

Search date:29.04.2020

#1. coronovirus*

#2. coronavir*

#3. 'corona virus'

#4. 'virus corona'

#5. 'corono virus'

#6. 'virus corono'

#7. hcov*

#8. 'covid-19'

#9. covid19*

#10. 'covid 19'

#11. '2019-ncov'

#12. cv19*

#13. 'cv-19'

#14. 'cv 19'

#15. 'n-cov'

#16. ncov*

#17. 'sars-cov-2'

#18. wuhan*:ti,ab AND (virus OR viruses OR viral)

#19. covid* AND (virus OR viruses OR viral)

#20. 'sars-cov'

#21. 'sars cov'

#22. 'sars-coronavirus'

#23. 'severe acute respiratory syndrome'

#24. 'mers-cov'

#25. 'mers cov'

#26. 'middle east respiratory syndrome'

#27. 'middle-east respiratory syndrome'

#28. "covid-19-related"

#29. "SARS-CoV-2-related"

#30. "SARS-CoV2-related"

#31. "2019-nCoV-related"

#32. "cv-19-related"

#33. "n-cov-related"

#34. #1 OR #2 OR #3 OR #4 OR #5 OR #6 OR #7 OR #8 OR #9 OR #10 OR #11 OR #12 OR #13 OR #14 OR #15 OR #16 OR #17 OR #18 OR #19 OR #20 OR #21 OR #22 OR #23 OR #24 OR #25 OR #26 OR #27 OR #28 OR #29 OR #30 OR #31 OR #32 OR #33

#35.'incidence'/de

#36. mortality:lnk

#37. 'follow up studies'/de

#38. prognos*:ti,ab,kw

#39. predict*:ti,ab,kw

#40. course*:ti,ab,kw

#41. #35 OR #36 OR #37 OR #38 OR #39 OR #40

#42.2020:py

#43. #34 AND #41 AND #42

*Search strategy for CENTRAL (The Cochrane Library)*

Search date:29.04.2020

#1 coronavir*:ti,ab,kw

#2 coronovirus*:ti,ab,kw

#3 "corona virus":ti,ab,kw

#4 "virus corona":ti,ab,kw

#5 "corono virus":ti,ab,kw

#6 "virus corono":ti,ab,kw

#7 hcov*:ti,ab,kw

#8 "covid-19":ti,ab,kw

#9 covid19*:ti,ab,kw

#10 "covid 19":ti,ab,kw

#11 “2019-nCoV“:ti,ab,kw

#12 cv19* :ti,ab,kw

#13 "cv-19":ti,ab,kw

#14 "cv 19":ti,ab,kw

#15 "n-cov":ti,ab,kw

#16 ncov*:ti,ab,kw

#17 "sars-cov-2"

#18 (wuhan* AND (virus OR viruses OR viral):ti,ab,kw

#19 covid* AND (virus OR viruses OR viral):ti,ab,kw

#20 "sars-cov":ti,ab,kw

#21 "sars cov" :ti,ab,kw

#22 "sars-coronavirus":ti,ab,kw

#23 "severe acute respiratory syndrome":ti,ab,kw

#24 "mers-cov":ti,ab,kw

#25 "mers cov":ti,ab,kw

#26 "middle east respiratory syndrome":ti,ab,kw

#27 "middle-east respiratory syndrome":ti,ab,kw

#28 "covid-19-related":ti,ab,kw

#29 "SARS-CoV-2-related":ti,ab,kw

#30 "SARS-CoV2-related":ti,ab,kw

#31 "2019-nCoV-related":ti,ab,kw

#32 "cv-19-related":ti,ab,kw

#33 "n-cov-related":ti,ab,kw

#34 #1 OR #2 OR #3 OR #4 OR #5 OR #6 OR #7 OR #8 OR #9 OR #10 OR #11 OR #12 OR #13 OR #14 OR #15 OR #16 OR #17 OR #18 OR #19 OR #20 OR #21 OR #22 OR #23 OR #24 OR #25 OR #26 OR #27 OR #28 OR #29 OR #30 OR #31 OR #32 OR #33

#35 MeSH descriptor: [Incidence] this term only

#36 MeSH descriptor: [Mortality] explode all trees

#37 MeSH descriptor: [Follow-Up Studies] this term only

#38 (prognos*):ti,ab,kw

#39 (predict*):ti,ab,kw

#40 (course*):ti,ab,kw

#41 #35 OR #36 OR #37 OR #38 OR #39 OR #40

#42 2020

#43 #34 AND #41 AND #42

**Certainty of the evidence assessment**

***Risk of bias***

We used the Quality in Prognosis Studies tool (QUIPS) for prognostic factor studies.^11^ To be rated as low risk of bias studies needed to be prospective, have appropriately assessed prognostic factors (measured at baseline) and outcomes and analyzed the information by considering at least age, one comorbidity and one parameter of disease severity as potential confounders. To be rated as moderate risk of bias studies needed to have appropriately assessed prognostic factors and outcomes and analyzed the information by considering at least one of the pre-defined core set of variables: age, one comorbidity or one parameter of disease severity as potential confounders. The remaining studies were categorized as high risk of bias. RoB was assessed on a study basis but the domain related to considering potential confounder was also assessed on a prognostic factor basis as some studies provided adjusted estimates for some but not all prognostic factors. For the primary analysis we downgraded the certainty of the evidence for risk of bias when no studies with moderate or low risk of bias providing adjusted estimates were available or when subgroup analysis showed inconsistency between moderate/low risk of bias adjusted estimates and high risk of bias studies and the overall pooled estimate of effects was used. We also performed a sensitivity analysis in which we downgraded for risk of bias only when adjusted estimates were not available or when subgroup analysis showed inconsistency between adjusted and unadjusted estimates and the overall pooled estimate was used.

***Inconsistency***

We used visual inspection of the forest plots and the I2 statistic to assess inconsistency. In doing so we considered the variability in point estimates and confidence interval overlap in relation to the thresholds set (see contextualization).

***Imprecision***

We rated down for imprecision when the 95%CI of the pooled estimates crossed the thresholds set (see contextualization). Additionally, we rated down for imprecision if the number of events was less than 200 as we assumed that the optimal information size was not met.^s1^

***Selective reporting bias***

In cases when most of the weight of the pooled estimates were provided by studies in which a multivariable analysis was performed but no adjusted estimates were provided for that particular variable, we considered rating down for selective reporting.

***Publication bias***

Given the nature of our research question (no interventions involved) and the facilities for reporting research results in this specific context (most of identified studies were published as preprint at the moment we performed the search). We assumed that publication bias was not a major issue and did not explore it while addressing certainty of the evidence.

**References**

s1. Guyatt GH, Oxman AD, Kunz R, et al. GRADE Guidelines 6. Rating the Quality of Evidence—Imprecision. *J Clin Epidemiol* 2011; 64(12): 1283–93.<https://doi.org/10.1016/j.jclinepi.2011.01.012>
