# Supplementary material for: Prognostic factors for severity and mortality in patients infected with COVID-19: A systematic review
Source: PLoS One. 2020 Nov 17;15(11):e0241955. doi: 10.1371/journal.pone.0241955 (PMC7671522; doi:10.1371/journal.pone.0241955)

Forest plot comparison: Covid 19 mortality risk

Glossary

ADJ=0: crude estimate

ADJ=1: adjust estimate

LRB=0: moderate and high subgroup analysis by risk of bias

LRB=1: low subgroup analysis by risk of bias

MRB=0: high subgroup analysis by risk of bias

MRB=1: moderate and low subgroup analysis by risk of bias

ALT: Alanine aminotransferase

APTT: activated partial thromboplastin time

APACHE: Acute Physiology And Chronic Health Evaluation II

AST: Aspartate aminotransferase

BUN: Blood urea nitrogen

PT: prothrombin time

SOFA: The sequential organ failure assessment score

FDP: Fibrin Degradation Product

PT: prothrombin time

Candidate variable: Age (older than 50 - 65 years), outcome: mortality, subgroup analysis by risk of bias (moderate/high vs low)

Study	TE	seTE	OR	95%-CI	Weight (fixed)	Weight (random)
**LRB = 0**						
Wu C_WJH	4.44	1.5990	84.60	[3.68; 1942.84]	0.1%	0.7%
Yang X_WJY	1.13	0.5942	3.10	[0.97; 9.92]	1.0%	3.1%
Lu Jiatao_WHH	2.17	0.8361	8.72	[1.69; 44.90]	0.5%	2.0%
Xiao M_ECRH	2.23	0.2796	9.32	[5.39; 16.12]	4.5%	5.5%
Shuai Z_UHTMC	1.23	0.6969	3.41	[0.87; 13.36]	0.7%	2.5%
Tan L_GHCTC	1.16	0.9131	3.18	[0.53; 19.05]	0.4%	1.7%
Cheng Y_TH	1.03	0.2586	2.79	[1.68; 4.63]	5.2%	5.7%
Fu L_UH	1.09	0.4075	2.98	[1.34; 6.63]	2.1%	4.4%
Li J_WRCH	2.38	0.3859	10.77	[5.06; 22.94]	2.3%	4.6%
Wang S_ZH	3.78	3.3397	43.71	[0.06; 30437.09]	0.0%	0.2%
Sun X_PUMCH	1.43	0.6678	4.18	[1.13; 15.48]	0.8%	2.7%
FY_JH, SPHCC, TPH	1.94	0.8132	6.94	[1.41; 34.16]	0.5%	2.1%
NS_BEMC	1.98	1.1093	7.27	[0.83; 63.97]	0.3%	1.3%
Chen R_Mu	1.27	0.5542	3.57	[1.20; 10.57]	1.1%	3.3%
Li J_CHW	0.93	0.3389	2.54	[1.31; 4.94]	3.0%	5.0%
WangY_TH	1.39	0.2457	4.00	[2.47; 6.48]	5.8%	5.9%
Paranjpel_MSHCS	2.01	0.2434	7.46	[4.63; 12.03]	5.9%	5.9%
Zhu Q_WUZH	3.14	1.0354	23.15	[3.04; 176.13]	0.3%	1.4%
Wang D_WH/XH	2.90	0.6786	18.13	[4.80; 68.57]	0.8%	2.6%
Xie J_UHW	1.33	0.9937	3.78	[0.54; 26.50]	0.4%	1.5%
Auld S_TEHA	1.24	0.3503	3.44	[1.73; 6.84]	2.8%	4.9%
Grasselli G_LIN	1.20	0.1362	3.32	[2.54; 4.34]	18.8%	6.7%
Li H_TH	1.22	0.6559	3.40	[0.94; 12.30]	0.8%	2.7%
LiX_TH	0.67	0.2585	1.96	[1.18; 3.25]	5.2%	5.7%
Li Y_TH	4.52	3.2280	92.14	[0.16; 51539.66]	0.0%	0.2%
**Fixed effect model**			4.04	[3.50; 4.68]	63.4%	--
**Random effects model**			4.51	[3.47; 5.86]	--	82.4%
Heterogeneity: $I^2 = 53\%$, $\tau^2 = 0.1718$, $p < 0.01$						
**LRB = 1**						
Du Rong-Hui_WPH	1.33	0.6930	3.77	[0.97; 14.66]	0.7%	2.6%
Rossi P_	1.48	0.5617	4.40	[1.46; 13.24]	1.1%	3.3%
MandeepR_MC	0.66	0.1045	1.93	[1.57; 2.37]	31.9%	6.9%
Docherty MB_MC	2.10	0.3521	8.19	[4.11; 16.33]	2.8%	4.9%
**Fixed effect model**			2.24	[1.85; 2.71]	36.6%	--
**Random effects model**			3.88	[1.62; 9.32]	--	17.6%
Heterogeneity: $I^2 = 83\%$, $\tau^2 = 0.6038$, $p < 0.01$						
**Fixed effect model**			**3.26**	**[2.90; 3.66]**	**100.0%**	**--**
**Random effects model**			**4.44**	**[3.39; 5.81]**	**--**	**100.0%**
Heterogeneity: $I^2 = 70\%$, $\tau^2 = 0.2640$, $p < 0.01$						
Residual heterogeneity: $I^2 = 61\%$, $p < 0.01$						

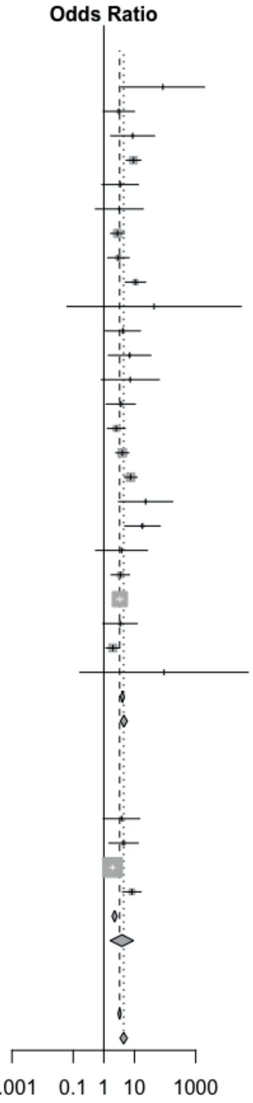

Candidate variable: Age increase (per 1 year), outcome: mortality, subgroup analysis by risk of bias: (high vs moderate/low)

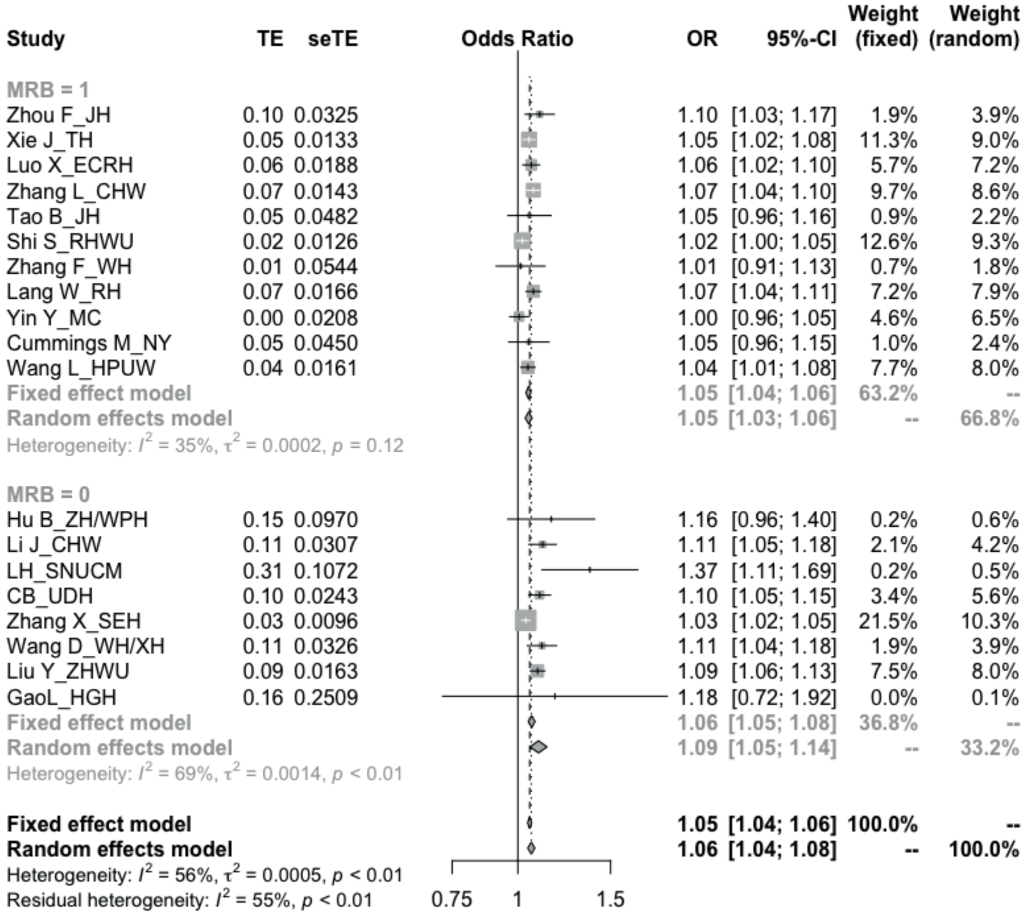

Candidate variable: Male gender, outcome: mortality, subgroup analysis by risk of bias: (high vs moderate/low)

Study	TE	seTE	OR	95%-CI	Weight (fixed)	Weight (random)
**MRB = 0**						
Zhou F_JH	0.49	0.3458	1.64	[0.83; 3.23]	1.4%	2.0%
Wu C_WJH	-0.58	0.4945	0.56	[0.21; 1.48]	0.7%	1.3%
He XW_TMC	-0.12	0.5640	0.89	[0.29; 2.68]	0.5%	1.1%
Ruan Q_JY	0.34	0.3555	1.41	[0.70; 2.83]	1.3%	2.0%
Tang N_TH	1.14	0.5359	3.12	[1.09; 8.92]	0.6%	1.2%
Yang X_WJY	-0.20	0.6137	0.82	[0.25; 2.72]	0.4%	1.0%
Yuan M_CH	-0.29	0.8079	0.75	[0.15; 3.65]	0.3%	0.6%
Du Rong-Hui_WPH	-0.30	0.4653	0.74	[0.30; 1.85]	0.8%	1.5%
Xie J_TH	-0.30	0.2323	0.74	[0.47; 1.16]	3.1%	2.8%
Xie J_TH	-0.15	0.3498	0.86	[0.43; 1.71]	1.4%	2.0%
Lu Jiatao_WHH	0.10	0.3334	1.10	[0.57; 2.11]	1.5%	2.1%
Chen M_SH- WC	1.20	0.4484	3.32	[1.38; 8.00]	0.8%	1.5%
Xiao M_ECRH	0.49	0.2327	1.63	[1.03; 2.57]	3.1%	2.8%
Shuai Z_UHTMC	1.40	0.3903	4.04	[1.88; 8.68]	1.1%	1.8%
Tan L_GHCTC	2.85	1.1509	17.29	[1.81; 164.95]	0.1%	0.3%
Zhang L_CHW	1.13	0.3430	3.09	[1.58; 6.06]	1.4%	2.1%
Cheng Y_TH	0.99	0.2792	2.70	[1.56; 4.66]	2.2%	2.5%
Fu L_UH	-0.12	0.3770	0.89	[0.42; 1.86]	1.2%	1.9%
Hu B_ZH/WPH	0.17	0.7146	1.18	[0.29; 4.81]	0.3%	0.8%
Jin JM_WUH	0.86	0.3651	2.36	[1.15; 4.82]	1.3%	1.9%
Li J_WRCH	1.03	0.3329	2.79	[1.45; 5.35]	1.5%	2.1%
Wen C_JH	0.53	0.5264	1.70	[0.61; 4.78]	0.6%	1.2%
Zhang F_WH	0.13	0.6565	1.14	[0.32; 4.14]	0.4%	0.9%
Zhang H_ZH	1.05	0.6355	2.85	[0.82; 9.91]	0.4%	0.9%
Zhang S_JH	0.01	0.2690	1.01	[0.60; 1.71]	2.3%	2.5%
Jit S_MC	0.87	0.3476	2.38	[1.21; 4.71]	1.4%	2.0%
Lang W_RH	0.54	0.2751	1.72	[1.00; 2.95]	2.2%	2.5%
Sun X_PUMCH	1.72	0.6822	5.60	[1.47; 21.33]	0.4%	0.8%
LH_SNUCM	0.58	0.1906	1.79	[1.23; 2.61]	4.6%	3.1%
CB_UDH	1.15	0.3872	3.17	[1.48; 6.77]	1.1%	1.8%
Chen X_TH	1.04	0.3322	2.83	[1.47; 5.42]	1.5%	2.1%
Zhang X_SEH	0.42	0.2821	1.53	[0.88; 2.65]	2.1%	2.5%
Rossi P_	0.50	0.1547	1.65	[1.22; 2.24]	7.0%	3.4%
Li J_CHW	0.67	0.2666	1.95	[1.15; 3.28]	2.4%	2.6%
Yin Y_MC	0.38	0.4135	1.46	[0.65; 3.29]	1.0%	1.7%
WangY_TH	0.24	0.2223	1.27	[0.82; 1.96]	3.4%	2.9%
Paranjpel_MSHCS	0.20	0.1376	1.22	[0.93; 1.60]	8.9%	3.5%
HuZ_TH	0.79	0.4454	2.21	[0.92; 5.28]	0.8%	1.5%
Wen J_ZH	1.55	0.4972	4.70	[1.77; 12.45]	0.7%	1.3%
Benelli G_HC	0.38	0.2846	1.47	[0.84; 2.56]	2.1%	2.4%
Yang J_WUH	1.89	0.6994	6.60	[1.68; 26.00]	0.3%	0.8%
Zhang J_LH	0.33	0.9487	1.39	[0.22; 8.92]	0.2%	0.5%
Zhu Q_WUZH	1.34	0.5389	3.84	[1.33; 11.03]	0.6%	1.2%
Zhu B_BTH	1.03	0.5600	2.79	[0.93; 8.37]	0.5%	1.1%
Wang D_WH/XH	1.98	0.8757	7.22	[1.30; 40.20]	0.2%	0.6%
Xie J_UHW	1.03	0.3181	2.80	[1.50; 5.22]	1.7%	2.2%
MandeepR_MC	0.24	0.0952	1.27	[1.06; 1.53]	18.6%	3.8%
Auld S_TEHA	-0.46	0.3197	0.63	[0.34; 1.18]	1.6%	2.2%
Liu Y_ZHWU	1.47	0.4305	4.35	[1.87; 10.11]	0.9%	1.6%
FanJ_ZH	-0.12	1.1118	0.89	[0.10; 7.86]	0.1%	0.4%
Li H_TH	0.15	0.4946	1.17	[0.44; 3.08]	0.7%	1.3%
Li Y_TH	1.79	1.2076	6.00	[0.56; 63.98]	0.1%	0.3%
Wang L_HPUW	1.42	0.5168	4.12	[1.50; 11.36]	0.6%	1.3%
**Fixed effect model**			1.56	[1.44; 1.69]	94.7%	--
**Random effects model**			1.74	[1.51; 2.02]	--	92.7%
Heterogeneity: $I^2 = 56\%$, $\tau^2 = 0.1259$, $p < 0.01$						
**MRB = 1**						
Tao B_JH	-0.09	1.2407	0.91	[0.08; 10.39]	0.1%	0.3%
Tian G_ MC	0.00	0.3122	1.00	[0.54; 1.84]	1.7%	2.3%
Cummings M_NY	0.38	0.3469	1.46	[0.74; 2.88]	1.4%	2.0%
LiX_TH	0.61	0.2932	1.83	[1.03; 3.26]	2.0%	2.4%
TieLong C_ZH	2.62	1.1658	13.80	[1.40; 135.58]	0.1%	0.3%
**Fixed effect model**			1.47	[1.03; 2.08]	5.3%	--
**Random effects model**			1.51	[0.94; 2.42]	--	7.3%
Heterogeneity: $I^2 = 33\%$, $\tau^2 = 0.0888$, $p = 0.20$						
**Fixed effect model**			**1.55**	**[1.43; 1.68]**	**100.0%**	**--**
**Random effects model**			**1.72**	**[1.50; 1.98]**	**--**	**100.0%**
Heterogeneity: $I^2 = 54\%$, $\tau^2 = 0.1204$, $p < 0.01$						
Residual heterogeneity: $I^2 = 55\%$, $p < 0.01$						

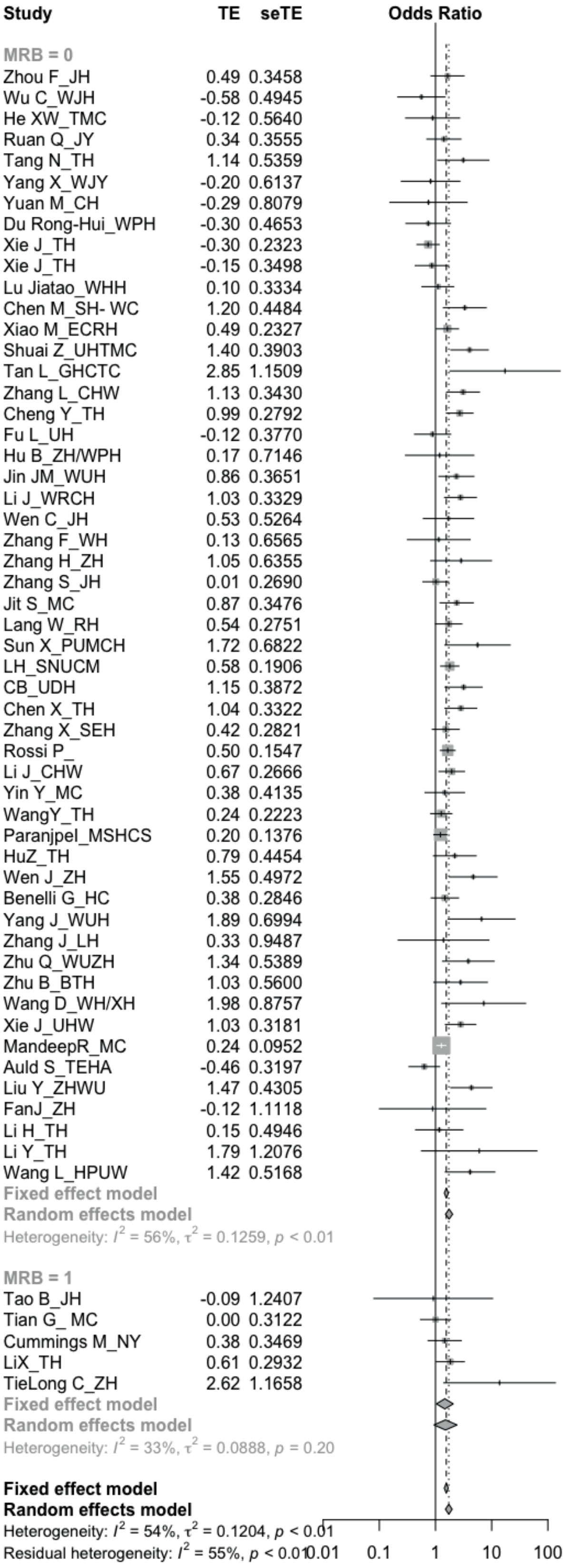

Candidate variable: Smoking (Active, present smoker), outcome: mortality, subgroup analysis: (crude vs adjusted)

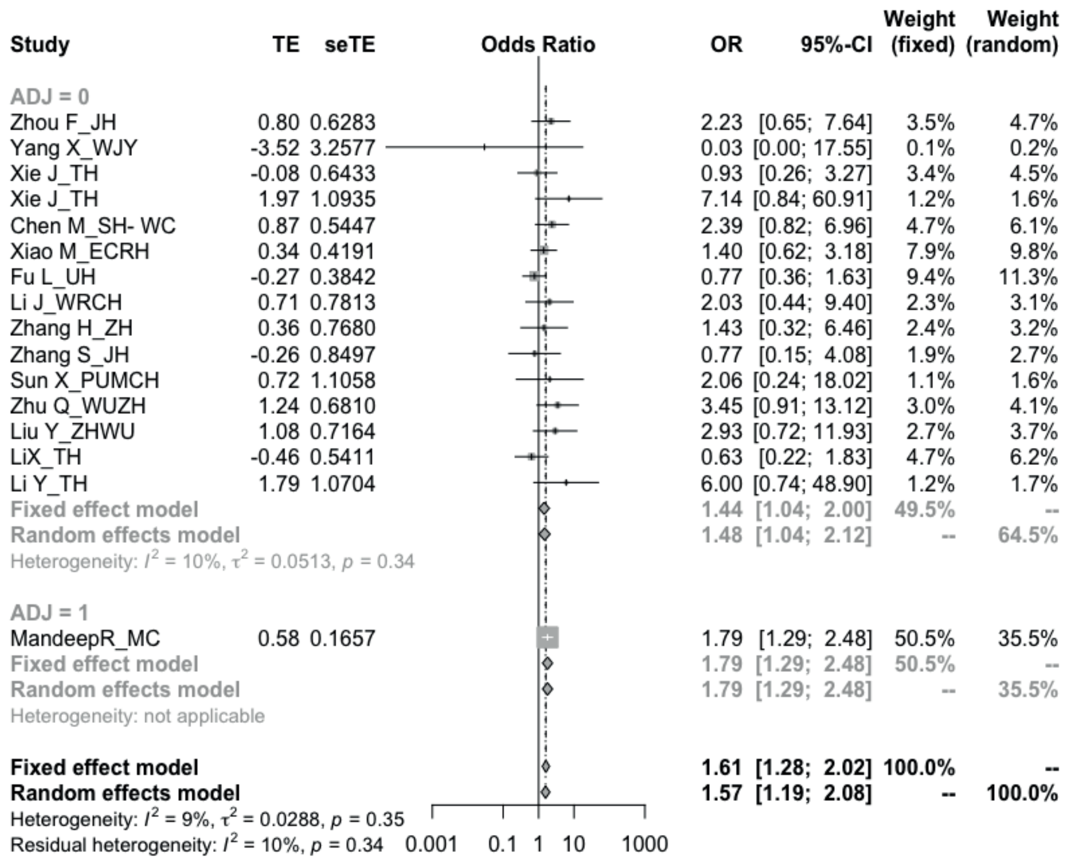

Candidate variable: Cardiovascular disease (coronary heart disease or congestive heart failure), outcome: mortality, subgroup analysis by risk of bias: (moderate/high vs low)

Study	TE	seTE	OR	95%-CI	Weight (fixed)	Weight (random)
**LRB = 0**						
Zhou F_JH	0.76	1.0780	2.14	[0.26; 17.70]	0.2%	0.7%
Wu C_WJH	-0.11	0.7435	0.90	[0.21; 3.86]	0.4%	1.3%
He XW_TMC	0.86	0.7893	2.37	[0.50; 11.13]	0.3%	1.2%
Ruan Q_JY	5.26	3.1792	192.33	[0.38; 97770.81]	0.0%	0.1%
Yang X_WJY	-0.07	0.9609	0.93	[0.14; 6.12]	0.2%	0.8%
Yuan M_CH	4.25	3.2475	70.43	[0.12; 40926.83]	0.0%	0.1%
Xie J_TH	2.48	1.0475	12.00	[1.54; 93.50]	0.2%	0.7%
Xie J_TH	2.31	3.3374	10.06	[0.01; 6972.51]	0.0%	0.1%
Chen M_SH- WC	1.89	0.5224	6.63	[2.38; 18.46]	0.7%	2.1%
Xiao M_ECRH	0.99	0.3577	2.70	[1.34; 5.43]	1.6%	3.2%
Shuai Z_UHTMC	-0.06	0.5112	0.94	[0.35; 2.57]	0.8%	2.2%
Tan L_GHCTC	5.18	3.2236	177.00	[0.32; 98145.76]	0.0%	0.1%
Zhang L_CHW	1.40	0.5943	4.04	[1.26; 12.95]	0.6%	1.8%
Tao B_JH	1.67	1.2412	5.29	[0.46; 60.29]	0.1%	0.5%
Fu L_UH	-0.39	0.7805	0.68	[0.15; 3.13]	0.3%	1.2%
Hu B_ZH/WPH	5.03	3.2102	153.33	[0.28; 82832.42]	0.0%	0.1%
Li J_WRCH	1.74	0.3567	5.70	[2.83; 11.47]	1.6%	3.2%
Li J_CHW	1.19	0.6011	3.30	[1.02; 10.72]	0.6%	1.7%
Shi S_RHWU	0.37	0.4394	1.44	[0.61; 3.41]	1.0%	2.6%
Zhang F_WH	-0.28	0.6953	0.75	[0.19; 2.94]	0.4%	1.4%
Zhang H_ZH	2.46	0.8782	11.75	[2.10; 65.70]	0.3%	1.0%
Zhang S_JH	1.16	0.5118	3.18	[1.17; 8.66]	0.8%	2.2%
Lang W_RH	0.74	0.3722	2.09	[1.01; 4.33]	1.4%	3.1%
Sun X_PUMCH	0.85	1.1010	2.34	[0.27; 20.25]	0.2%	0.7%
FY_JH, SPHCC, TPH	-0.54	0.9608	0.58	[0.09; 3.81]	0.2%	0.8%
LH_SNUCM	0.12	0.2295	1.13	[0.72; 1.77]	3.8%	4.3%
CB_UDH	0.47	0.4775	1.60	[0.63; 4.08]	0.9%	2.3%
Chen R_Mu	1.51	0.7399	4.51	[1.06; 19.24]	0.4%	1.3%
Zhang X_SEH	-0.05	0.3151	0.96	[0.51; 1.77]	2.0%	3.5%
Li J_CHW	0.80	0.3065	2.23	[1.22; 4.07]	2.1%	3.6%
Yin Y_MC	0.31	0.5276	1.36	[0.48; 3.82]	0.7%	2.1%
WangY_TH	0.75	0.3394	2.13	[1.09; 4.13]	1.7%	3.3%
Paranjpel_MSHCS	1.09	0.1727	2.98	[2.12; 4.18]	6.7%	4.9%
HuZ_TH	0.41	0.6701	1.50	[0.40; 5.58]	0.4%	1.5%
Benelli G_HC	0.99	0.2783	2.68	[1.55; 4.63]	2.6%	3.9%
Zhang J_LH	4.16	3.2612	63.80	[0.11; 38084.54]	0.0%	0.1%
Zhu Q_WUZH	2.31	0.7550	10.03	[2.28; 44.06]	0.4%	1.2%
Wang D_WH/XH	0.17	0.9575	1.19	[0.18; 7.76]	0.2%	0.8%
Auld S_TEHA	0.72	0.3366	2.06	[1.07; 3.98]	1.8%	3.3%
Liu Y_ZHWU	1.87	0.5201	6.46	[2.33; 17.90]	0.7%	2.1%
FanJ_ZH	0.12	1.1118	1.12	[0.13; 9.94]	0.2%	0.6%
GaoL_HGH	0.20	0.5412	1.22	[0.42; 3.52]	0.7%	2.0%
LiX_TH	1.01	0.3983	2.73	[1.25; 5.97]	1.3%	2.9%
TieLong C_ZH	1.05	0.6898	2.86	[0.74; 11.06]	0.4%	1.4%
Li Y_TH	0.35	1.2814	1.42	[0.11; 17.46]	0.1%	0.5%
Wang L_HPUW	0.84	0.6006	2.33	[0.72; 7.55]	0.6%	1.7%
**Fixed effect model**			2.24	[1.95; 2.57]	39.6%	--
**Random effects model**			2.27	[1.84; 2.79]	--	80.0%
Heterogeneity: $I^2 = 39\%$, $\tau^2 = 0.1544$, $p < 0.01$						
**LRB = 1**						
Du Rong-Hui_WPH	0.90	0.6018	2.46	[0.76; 8.00]	0.6%	1.7%
Rossi P_	0.53	0.1872	1.70	[1.18; 2.45]	5.7%	4.7%
Cummings M_NY	-0.44	0.4241	0.65	[0.28; 1.48]	1.1%	2.7%
MandeepR_MC	0.99	0.1335	2.70	[2.08; 3.51]	11.2%	5.2%
Docherty MB_MC	0.34	0.0692	1.40	[1.22; 1.60]	41.8%	5.6%
**Fixed effect model**			1.60	[1.43; 1.79]	60.4%	--
**Random effects model**			1.65	[1.11; 2.46]	--	20.0%
Heterogeneity: $I^2 = 84\%$, $\tau^2 = 0.1408$, $p < 0.01$						
**Fixed effect model**			**1.82**	**[1.67; 1.99]**	**100.0%**	**--**
**Random effects model**			**2.13**	**[1.77; 2.56]**	**--**	**100.0%**

Heterogeneity: $I^2 = 55\%$, $\tau^2 = 0.1541$, $p < 0.01$

Residual heterogeneity: $I^2 = 50\%$, $p < 0.01$

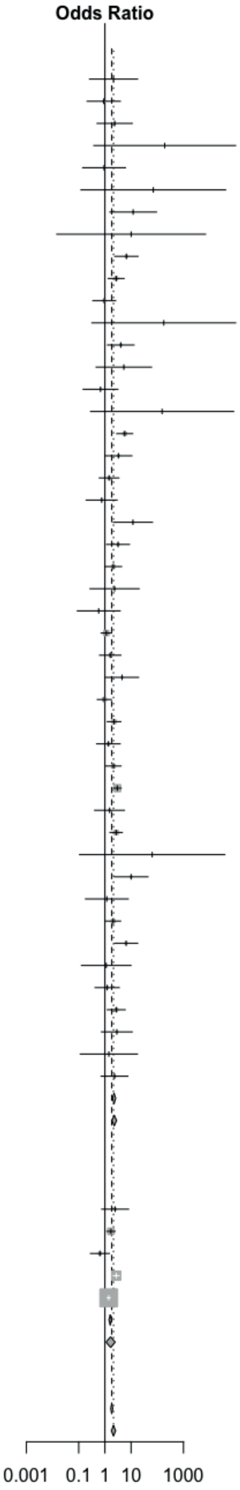

Candidate variable: Cardiac arrhythmia (as previous condition or new clinical finding), outcome: mortality, subgroup analysis: (crude vs adjusted)

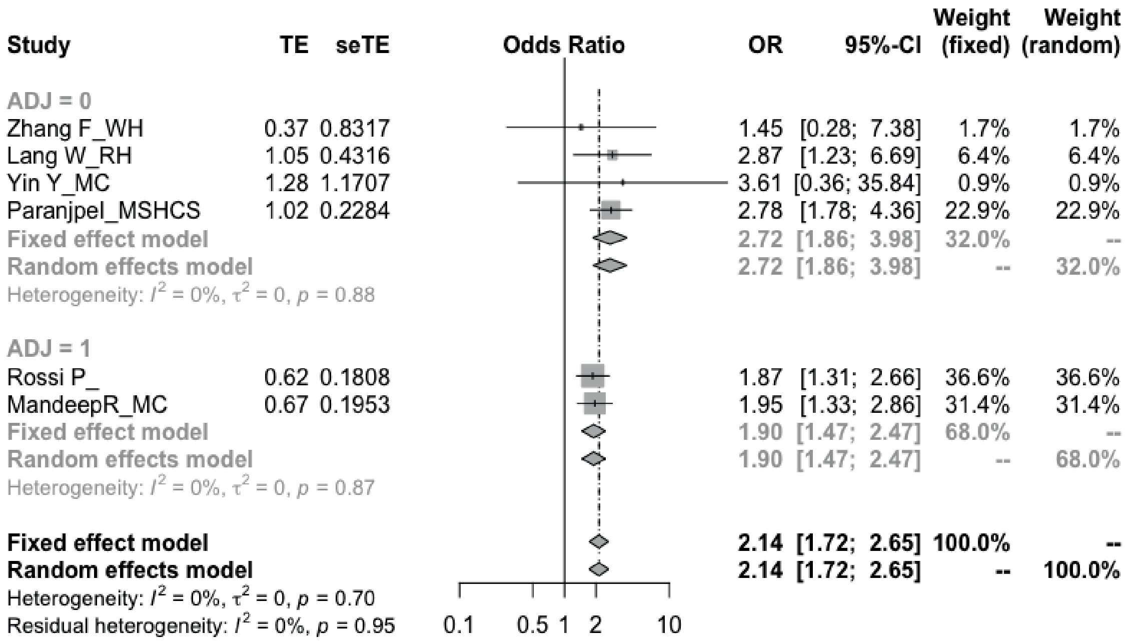

Candidate variable: Cerebrovascular disease (History of stroke or CNS disease), outcome: mortality, subgroup analysis by risk of bias: (high vs moderate/low)

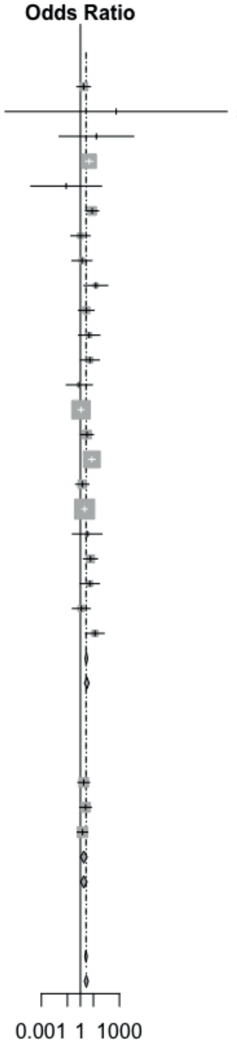

Study	TE	seTE	OR	95%-CI	Weight (fixed)	Weight (random)
**MRB = 0**						
Ruan Q_JY	0.57	0.6101	1.77	[0.53; 5.84]	3.0%	4.5%
Yang X_WJY	6.33	10.0115	559.20	[0.00; 185925668960.32]	0.0%	0.0%
Yuan M_CH	2.83	3.3587	17.00	[0.02; 12284.28]	0.1%	0.3%
Xiao M_ECRH	1.56	0.3473	4.75	[2.40; 9.37]	9.2%	7.1%
Shuai Z_UHTMC	-2.52	3.1890	0.08	[0.00; 41.70]	0.1%	0.3%
Zhang L_CHW	2.08	0.5821	7.99	[2.55; 25.00]	3.3%	4.7%
Tao B_JH	0.01	0.8608	1.01	[0.19; 5.47]	1.5%	2.9%
Hu B_ZH/WPH	0.27	0.8958	1.31	[0.23; 7.57]	1.4%	2.7%
Li J_WRCH	2.73	1.0675	15.27	[1.88; 123.71]	1.0%	2.1%
Zhang F_WH	1.04	0.7043	2.84	[0.71; 11.28]	2.2%	3.8%
Zhang H_ZH	1.55	0.9598	4.70	[0.72; 30.84]	1.2%	2.5%
Zhang S_JH	1.68	0.8474	5.37	[1.02; 28.27]	1.6%	3.0%
Sun X_PUMCH	-0.19	1.1661	0.83	[0.08; 8.16]	0.8%	1.8%
LH_SNUCM	0.10	0.2682	1.11	[0.66; 1.88]	15.5%	8.0%
Chen R_Mu	1.17	0.5745	3.21	[1.04; 9.89]	3.4%	4.8%
Li J_CHW	2.03	0.2970	7.58	[4.23; 13.57]	12.6%	7.7%
Yin Y_MC	0.34	0.5918	1.40	[0.44; 4.47]	3.2%	4.6%
Paranjpel_MSHCS	0.74	0.2474	2.09	[1.29; 3.40]	18.2%	8.2%
Zhang J_LH	1.20	1.3291	3.33	[0.25; 45.11]	0.6%	1.5%
Zhu Q_WUZH	1.79	0.6295	5.98	[1.74; 20.53]	2.8%	4.3%
Wang D_WH/XH	1.67	0.8608	5.31	[0.98; 28.71]	1.5%	2.9%
TieLong C_ZH	0.15	0.7925	1.16	[0.25; 5.50]	1.8%	3.2%
Wang L_HPUW	2.59	0.8185	13.30	[2.67; 66.15]	1.7%	3.1%
**Fixed effect model**			2.86	[2.29; 3.58]	86.7%	--
**Random effects model**			3.10	[2.10; 4.59]	--	83.8%
Heterogeneity: $I^2 = 54\%$, $\tau^2 = 0.3690$, $p < 0.01$						
**MRB = 1**						
Shi S_RHWU	0.59	0.5142	1.80	[0.66; 4.93]	4.2%	5.3%
Tian G_ MC	0.92	0.5233	2.50	[0.90; 6.97]	4.1%	5.2%
Lang W_RH	0.36	0.4713	1.44	[0.57; 3.63]	5.0%	5.7%
**Fixed effect model**			1.83	[1.04; 3.23]	13.3%	--
**Random effects model**			1.83	[1.04; 3.23]	--	16.2%
Heterogeneity: $I^2 = 0\%$, $\tau^2 = 0$, $p = 0.74$						
**Fixed effect model**			**2.70**	**[2.19; 3.32]**	**100.0%**	**--**
**Random effects model**			**2.85**	**[2.02; 4.01]**	**--**	**100.0%**

Heterogeneity: $I^2 = 50\%$, $\tau^2 = 0.3079$, $p < 0.01$
Residual heterogeneity: $I^2 = 50\%$, $p < 0.01$

Candidate variable: Diabetes, outcome: mortality, subgroup analysis by risk of bias: (moderate/high vs low)

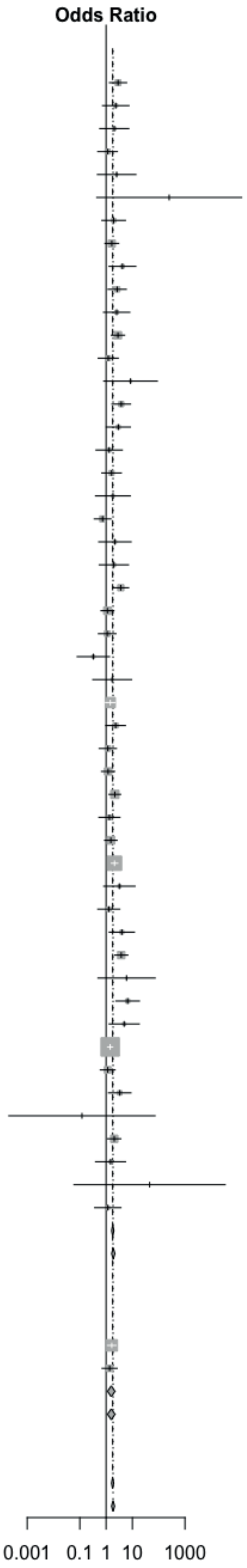

Study	TE	seTE	OR	95%-CI	Weight (fixed)	Weight (random)
**LRB = 0**						
Zhou F_JH	1.05	0.3826	2.85	[1.35; 6.03]	1.7%	2.2%
Wu C_WJH	0.85	0.5914	2.33	[0.73; 7.44]	0.7%	1.1%
He XW_TMC	0.72	0.6512	2.04	[0.57; 7.33]	0.6%	1.0%
Ruan Q_JY	0.13	0.4389	1.14	[0.48; 2.69]	1.3%	1.8%
Yang X_WJY	0.92	0.8593	2.52	[0.47; 13.58]	0.3%	0.6%
Yuan M_CH	5.52	3.2370	250.75	[0.44; 142741.06]	0.0%	0.0%
Du Rong-Hui_WPH	0.66	0.5272	1.94	[0.69; 5.45]	0.9%	1.4%
Xie J_TH	0.50	0.3057	1.65	[0.90; 3.00]	2.6%	3.0%
Xie J_TH	1.43	0.5989	4.18	[1.29; 13.51]	0.7%	1.1%
Lu Jiatao_WHH	0.97	0.4050	2.64	[1.19; 5.84]	1.5%	2.1%
Chen M_SH- WC	0.92	0.5861	2.52	[0.80; 7.95]	0.7%	1.1%
Xiao M_ECRH	1.04	0.2971	2.82	[1.58; 5.05]	2.8%	3.1%
Shuai Z_UHTMC	0.19	0.4489	1.20	[0.50; 2.90]	1.2%	1.8%
Tan L_GHCTC	2.14	1.1974	8.50	[0.81; 88.85]	0.2%	0.3%
Zhang L_CHW	1.33	0.4136	3.77	[1.68; 8.48]	1.4%	2.0%
Zhang Y_WCUH	1.07	0.5395	2.92	[1.01; 8.41]	0.8%	1.3%
Tao B_JH	0.27	0.5870	1.31	[0.41; 4.13]	0.7%	1.1%
Fu L_UH	0.48	0.4366	1.61	[0.68; 3.79]	1.3%	1.8%
Hu B_ZH/WPH	0.60	0.7768	1.82	[0.40; 8.33]	0.4%	0.7%
Shi S_RHWU	-0.31	0.3723	0.74	[0.35; 1.53]	1.8%	2.3%
Zhang F_WH	0.77	0.7223	2.17	[0.53; 8.93]	0.5%	0.8%
Zhang H_ZH	0.68	0.6529	1.97	[0.55; 7.09]	0.6%	0.9%
Zhang S_JH	1.29	0.3506	3.62	[1.82; 7.20]	2.0%	2.5%
Tian G_ MC	0.12	0.2869	1.12	[0.64; 1.97]	3.0%	3.3%
Lang W_RH	0.10	0.3933	1.10	[0.51; 2.38]	1.6%	2.2%
Sun X_PUMCH	-1.12	0.7214	0.33	[0.08; 1.34]	0.5%	0.8%
FY_JH, SPHCC, TPH	0.54	0.8669	1.71	[0.31; 9.35]	0.3%	0.6%
LH_SNUCM	0.36	0.2131	1.44	[0.95; 2.19]	5.4%	4.4%
CB_UDH	0.83	0.4395	2.30	[0.97; 5.44]	1.3%	1.8%
Chen X_TH	0.15	0.3850	1.16	[0.55; 2.47]	1.6%	2.2%
Zhang X_SEH	0.17	0.2934	1.19	[0.67; 2.11]	2.8%	3.2%
Li J_CHW	0.76	0.2613	2.15	[1.29; 3.58]	3.6%	3.6%
Yin Y_MC	0.29	0.4689	1.34	[0.53; 3.35]	1.1%	1.7%
WangY_TH	0.42	0.2795	1.52	[0.88; 2.62]	3.1%	3.4%
Paranjpel_MSHCS	0.74	0.1505	2.09	[1.56; 2.81]	10.7%	5.7%
GuoW_WUH	1.16	0.6985	3.20	[0.81; 12.58]	0.5%	0.8%
HuZ_TH	0.23	0.4904	1.26	[0.48; 3.31]	1.0%	1.5%
Wen J_ZH	1.38	0.5634	3.96	[1.31; 11.95]	0.8%	1.2%
Benelli G_HC	1.32	0.2974	3.76	[2.10; 6.74]	2.8%	3.1%
Zhang J_LH	1.79	1.2780	6.00	[0.49; 73.45]	0.1%	0.3%
Zhu Q_WUZH	1.89	0.5238	6.60	[2.36; 18.42]	0.9%	1.4%
Wang D_WH/XH	1.59	0.6710	4.88	[1.31; 18.18]	0.5%	0.9%
MandeepR_MC	0.35	0.1170	1.43	[1.13; 1.79]	17.8%	6.4%
Auld S_TEHA	0.14	0.3288	1.15	[0.60; 2.19]	2.3%	2.8%
Liu Y_ZHWU	1.19	0.4990	3.30	[1.24; 8.78]	1.0%	1.5%
FanJ_ZH	-2.11	3.2659	0.12	[0.00; 72.71]	0.0%	0.0%
LiX_TH	0.71	0.2961	2.04	[1.14; 3.65]	2.8%	3.2%
TieLong C_ZH	0.39	0.6699	1.48	[0.40; 5.50]	0.5%	0.9%
Li Y_TH	3.81	3.3780	45.00	[0.06; 33771.25]	0.0%	0.0%
Wang L_HPUW	0.15	0.5888	1.16	[0.36; 3.67]	0.7%	1.1%
**Fixed effect model**			**1.79**	**[1.62; 1.98]**	**91.1%**	--
**Random effects model**			**1.87**	**[1.62; 2.16]**	--	**92.4%**
Heterogeneity: I^2 = 33%, τ^2 = 0.0677, p = 0.02						
**LRB = 1**						
Rossi P_	0.50	0.1896	1.64	[1.13; 2.38]	6.8%	4.9%
Cummings M_NY	0.31	0.3371	1.36	[0.70; 2.64]	2.1%	2.7%
**Fixed effect model**			**1.57**	**[1.14; 2.17]**	**8.9%**	--
**Random effects model**			**1.57**	**[1.14; 2.17]**	--	**7.6%**
Heterogeneity: I^2 = 0%, τ^2 = 0, p = 0.63						
**Fixed effect model**			**1.77**	**[1.60; 1.95]**	**100.0%**	**--**
**Random effects model**			**1.84**	**[1.61; 2.10]**	**--**	**100.0%**
Heterogeneity: I^2 = 31%, τ^2 = 0.0581, p = 0.02						
Residual heterogeneity: I^2 = 31%, p = 0.02						

Candidate variable: arterial hypertension, outcome: mortality, subgroup analysis by risk of bias: (moderate/high vs low)

Study	TE	seTE	OR	95%-CI	Weight (fixed)	Weight (random)
**LRB = 0**						
Zhou F_JH	1.12	0.3386	3.05	[1.57; 5.92]	1.5%	2.3%
Wu C_WJH	0.99	0.5209	2.69	[0.97; 7.48]	0.7%	1.5%
He XW_TMC	0.13	0.5483	1.14	[0.39; 3.35]	0.6%	1.4%
Ruan Q_JY	0.65	0.3472	1.91	[0.97; 3.77]	1.5%	2.2%
Yuan M_CH	5.12	3.2346	166.60	[0.29; 94396.56]	0.0%	0.1%
Du Rong-Hui_WPH	1.41	0.4832	4.08	[1.58; 10.52]	0.8%	1.7%
Xie J_TH	0.79	0.2396	2.20	[1.38; 3.52]	3.1%	2.8%
Xie J_TH	1.94	0.4453	6.93	[2.90; 16.60]	0.9%	1.8%
Lu Jiatao_WHH	0.55	0.3613	1.74	[0.86; 3.53]	1.4%	2.2%
Chen M_SH- WC	0.87	0.4275	2.38	[1.03; 5.50]	1.0%	1.9%
Xiao M_ECRH	1.96	0.2540	7.08	[4.30; 11.64]	2.7%	2.7%
Shuai Z_UHTMC	0.30	0.3496	1.35	[0.68; 2.68]	1.5%	2.2%
Tan L_GHCTC	1.59	0.8150	4.90	[0.99; 24.21]	0.3%	0.8%
Zhang L_CHW	1.95	0.3403	7.01	[3.60; 13.66]	1.5%	2.3%
Tao B_JH	0.87	0.4197	2.38	[1.05; 5.42]	1.0%	1.9%
Fu L_UH	0.70	0.3911	2.02	[0.94; 4.35]	1.2%	2.0%
Hu B_ZH/WPH	0.41	0.6770	1.50	[0.40; 5.65]	0.4%	1.1%
Zhang F_WH	0.28	0.6513	1.32	[0.37; 4.73]	0.4%	1.1%
Zhang H_ZH	0.73	0.5759	2.08	[0.67; 6.44]	0.5%	1.3%
Zhang S_JH	0.96	0.2852	2.61	[1.50; 4.57]	2.2%	2.6%
Tian G_ MC	0.32	0.2583	1.37	[0.83; 2.28]	2.7%	2.7%
Lang W_RH	0.46	0.3091	1.59	[0.87; 2.91]	1.9%	2.4%
Sun X_PUMCH	0.59	0.6673	1.80	[0.49; 6.66]	0.4%	1.1%
FY_JH, SPHCC, TPH	0.46	0.7096	1.58	[0.39; 6.35]	0.4%	1.0%
LH_SNUCM	0.22	0.2767	1.25	[0.73; 2.15]	2.3%	2.6%
CB_UDH	-0.36	0.3930	0.70	[0.32; 1.51]	1.1%	2.0%
Chen X_TH	0.28	0.3111	1.32	[0.72; 2.43]	1.8%	2.4%
Yang G_HPHTCM	0.52	0.4683	1.68	[0.67; 4.21]	0.8%	1.7%
Zhang X_SEH	-0.09	0.2440	0.92	[0.57; 1.48]	3.0%	2.8%
Yin Y_MC	0.62	0.3857	1.86	[0.88; 3.97]	1.2%	2.1%
WangY_TH	0.73	0.2263	2.08	[1.34; 3.24]	3.5%	2.9%
Paranjpel_MSHCS	0.64	0.1385	1.89	[1.44; 2.48]	9.2%	3.3%
ZengZ_HH	0.31	0.4841	1.36	[0.53; 3.51]	0.8%	1.6%
HuZ_TH	0.10	0.4220	1.11	[0.48; 2.54]	1.0%	1.9%
Wen J_ZH	1.52	0.4564	4.58	[1.87; 11.20]	0.9%	1.8%
Benelli G_HC	0.98	0.2730	2.68	[1.57; 4.57]	2.4%	2.6%
Zhang J_LH	0.33	0.9487	1.39	[0.22; 8.92]	0.2%	0.6%
Zhu Q_WUZH	1.91	0.5050	6.75	[2.51; 18.15]	0.7%	1.6%
Wang D_WH/XH	0.09	0.7279	1.10	[0.26; 4.58]	0.3%	1.0%
Xie J_UHW	0.18	0.4150	1.20	[0.53; 2.71]	1.0%	1.9%
MandeepR_MC	-0.06	0.1044	0.94	[0.77; 1.16]	16.3%	3.4%
Auld S_TEHA	0.05	0.3446	1.05	[0.53; 2.06]	1.5%	2.3%
Grasselli G_LIN	0.81	0.1397	2.24	[1.71; 2.95]	9.1%	3.3%
Liu Y_ZHWU	1.37	0.3941	3.94	[1.82; 8.53]	1.1%	2.0%
FanJ_ZH	0.12	1.1118	1.12	[0.13; 9.94]	0.1%	0.5%
GaoL_HGH	0.48	0.5125	1.61	[0.59; 4.40]	0.7%	1.5%
LiX_TH	0.93	0.2491	2.54	[1.56; 4.14]	2.9%	2.7%
TieLong C_ZH	0.59	0.5797	1.80	[0.58; 5.61]	0.5%	1.3%
Li Y_TH	1.56	1.5174	4.75	[0.24; 92.96]	0.1%	0.3%
Wang L_HPUW	1.03	0.4377	2.80	[1.19; 6.60]	0.9%	1.8%
Fixed effect model			1.80	[1.65; 1.96]	91.7%	--
Random effects model			2.02	[1.70; 2.41]	--	95.2%
Heterogeneity: $I^2 = 68\%$, $\tau^2 = 0.2168$, $p < 0.01$						
**LRB = 1**						
Rossi P_	0.50	0.1532	1.64	[1.22; 2.22]	7.6%	3.2%
Cummings M_NY	1.01	0.4957	2.75	[1.04; 7.26]	0.7%	1.6%
Fixed effect model			1.72	[1.29; 2.29]	8.3%	--
Random effects model			1.72	[1.29; 2.29]	--	4.8%
Heterogeneity: $I^2 = 0\%$, $\tau^2 = 0$, $p = 0.32$						
**Fixed effect model**			**1.79**	**[1.65; 1.95]**	**100.0%**	**--**
**Random effects model**			**2.02**	**[1.71; 2.38]**	**--**	**100.0%**
Heterogeneity: $I^2 = 67\%$, $\tau^2 = 0.1962$, $p < 0.01$						
Residual heterogeneity: $I^2 = 68\%$, $p < 0.01$						

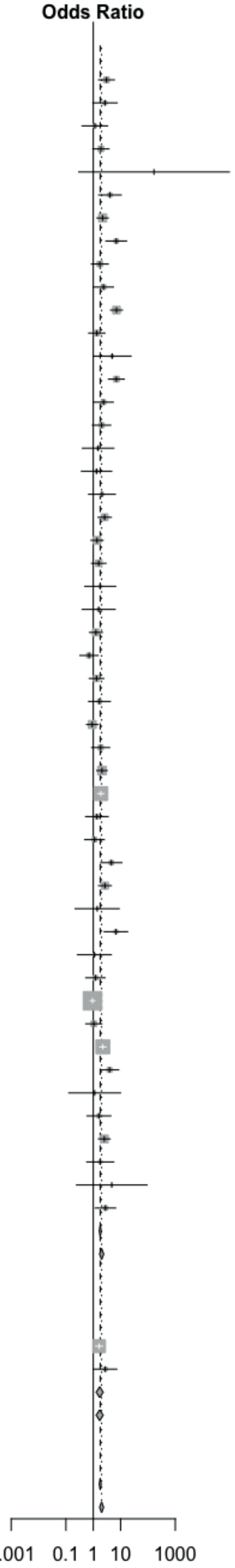

Candidate variable: obesity, outcome: mortality, subgroup analysis: (crude vs adjusted)

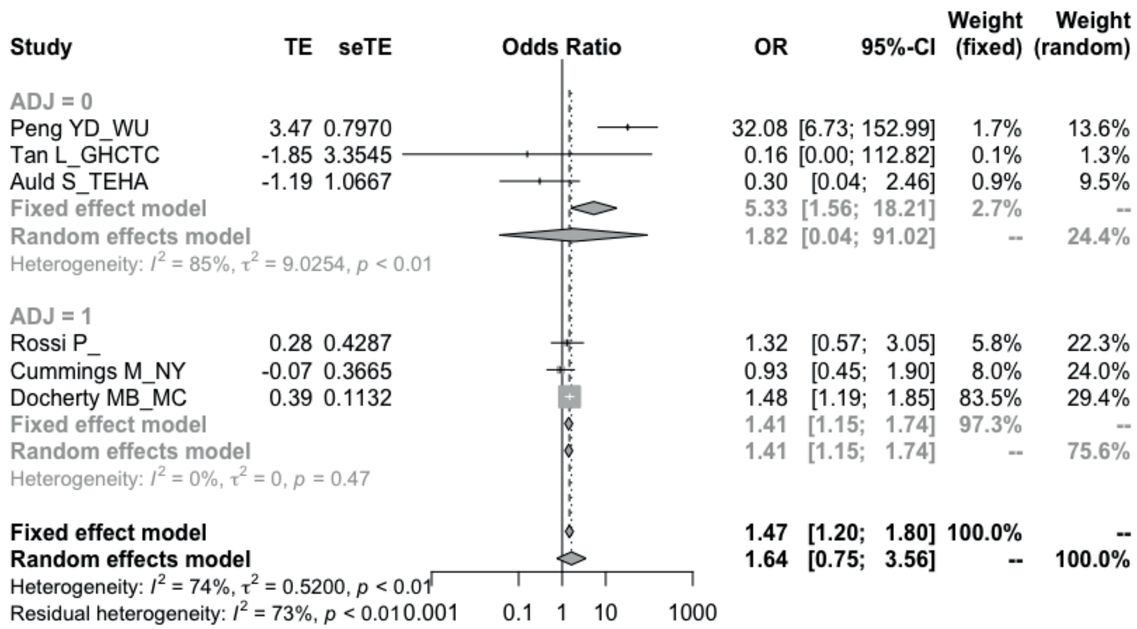

Candidate variable: chronic kidney disease, outcome: mortality, subgroup analysis by risk of bias: (moderate/high vs low)

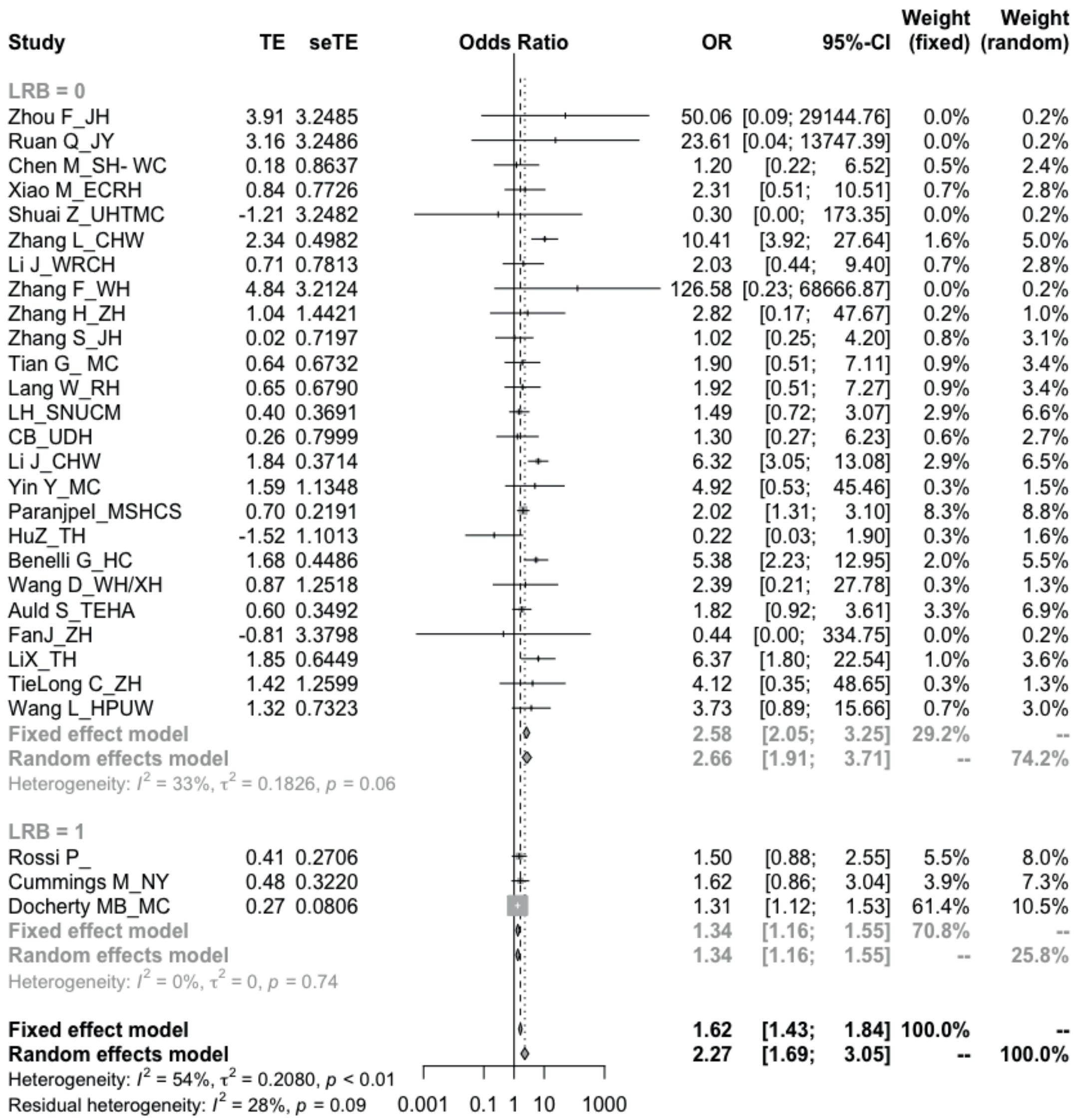

Candidate variable: asthma, outcome: mortality, subgroup analysis: (crude vs adjusted)

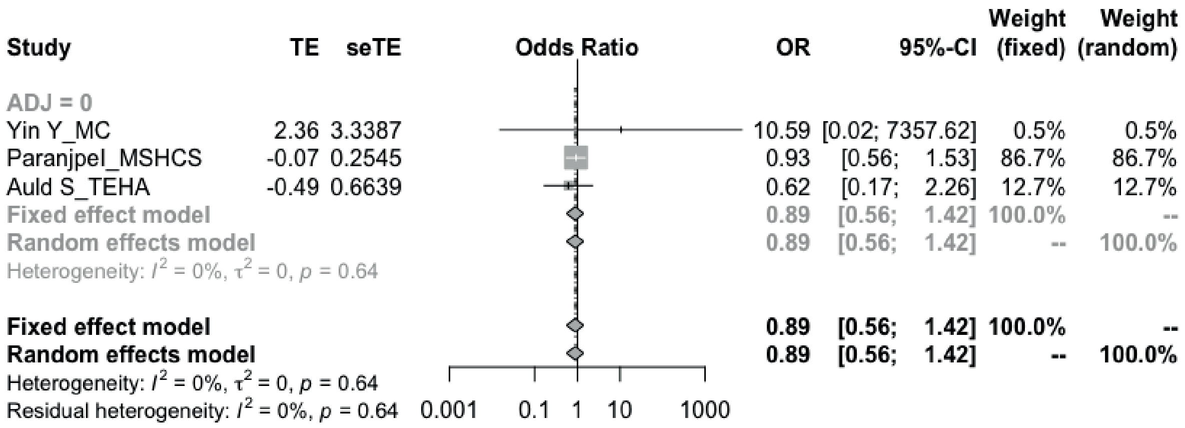

Candidate variable: COPD, outcome: mortality, subgroup analysis by risk of bias: (moderate/high vs low)

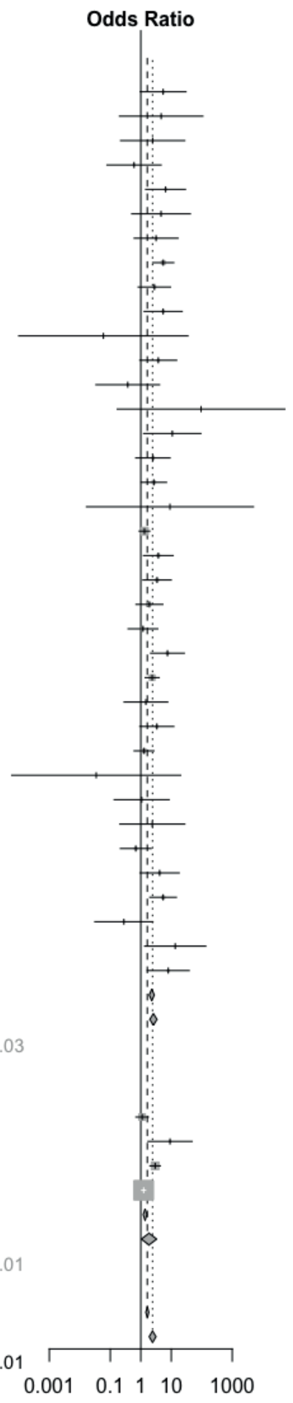

Study	TE	seTE	OR	95%-CI	Weight (fixed)	Weight (random)
**LRB = 0**						
Zhou F_JH	1.69	0.8814	5.40	[0.96; 30.39]	0.4%	1.7%
He XW_TMC	1.54	1.6062	4.66	[0.20; 108.53]	0.1%	0.6%
Peng YD_WU	0.90	1.2359	2.45	[0.22; 27.67]	0.2%	1.0%
Yang X_WJY	-0.51	1.0435	0.60	[0.08; 4.64]	0.3%	1.3%
Xie J_TH	1.87	0.7688	6.50	[1.44; 29.33]	0.5%	2.0%
Xie J_TH	1.53	1.1308	4.62	[0.50; 42.34]	0.3%	1.1%
Chen M_SH- WC	1.16	0.8447	3.18	[0.61; 16.64]	0.4%	1.8%
Xiao M_ECRH	1.69	0.4064	5.44	[2.45; 12.06]	1.9%	4.2%
Zhang L_CHW	1.03	0.6235	2.79	[0.82; 9.48]	0.8%	2.7%
Fu L_UH	1.69	0.7345	5.40	[1.28; 22.78]	0.6%	2.2%
Hu B_ZH/WPH	-2.83	3.2625	0.06	[0.00; 35.44]	0.0%	0.2%
Li J_WRCH	1.32	0.7100	3.74	[0.93; 15.05]	0.6%	2.3%
Shi S_RHWU	-0.99	1.2251	0.37	[0.03; 4.11]	0.2%	1.0%
Zhang H_ZH	4.55	3.2298	94.73	[0.17; 53174.31]	0.0%	0.2%
Zhang S_JH	2.38	1.1036	10.80	[1.24; 93.96]	0.3%	1.2%
Tian G_ MC	0.92	0.6626	2.50	[0.68; 9.16]	0.7%	2.5%
Lang W_RH	0.99	0.4879	2.68	[1.03; 6.97]	1.3%	3.6%
Sun X_PUMCH	2.20	3.2174	9.06	[0.02; 4961.83]	0.0%	0.2%
LH_SNUCM	0.28	0.2040	1.32	[0.88; 1.97]	7.7%	6.0%
Chen X_TH	1.32	0.5713	3.76	[1.23; 11.52]	1.0%	3.0%
Zhang X_SEH	1.23	0.5464	3.42	[1.17; 9.97]	1.1%	3.2%
Li J_CHW	0.65	0.5174	1.92	[0.70; 5.30]	1.2%	3.3%
Yin Y_MC	0.16	0.5717	1.18	[0.38; 3.61]	1.0%	3.0%
WangY_TH	2.02	0.6507	7.51	[2.10; 26.89]	0.8%	2.6%
Paranjpel_MSHCS	0.86	0.2699	2.36	[1.39; 4.01]	4.4%	5.4%
HuZ_TH	0.39	0.8446	1.47	[0.28; 7.71]	0.4%	1.8%
Wen J_ZH	1.21	0.6555	3.36	[0.93; 12.13]	0.7%	2.5%
Benelli G_HC	0.24	0.3818	1.28	[0.60; 2.70]	2.2%	4.4%
Zhang J_LH	-3.37	3.2549	0.03	[0.00; 20.33]	0.0%	0.2%
Zhu Q_WUZH	0.07	1.0605	1.07	[0.13; 8.55]	0.3%	1.2%
Wang D_WH/XH	0.87	1.2518	2.39	[0.21; 27.78]	0.2%	0.9%
Auld S_TEHA	-0.38	0.5924	0.68	[0.21; 2.19]	0.9%	2.9%
Liu Y_ZHWU	1.42	0.7562	4.14	[0.94; 18.23]	0.6%	2.1%
LiX_TH	1.69	0.5112	5.39	[1.98; 14.69]	1.2%	3.4%
TieLong C_ZH	-1.28	1.1205	0.28	[0.03; 2.50]	0.3%	1.1%
Li Y_TH	2.60	1.1785	13.50	[1.34; 135.98]	0.2%	1.0%
Wang L_HPUW	2.07	0.8093	7.94	[1.62; 38.76]	0.5%	1.9%
Fixed effect model			2.29	[1.89; 2.78]	33.6%	--
Random effects model			2.59	[1.98; 3.39]	--	79.5%
Heterogeneity: $I^2 = 32\%$, $\tau^2 = 0.1777$, $p = 0.03$						
**LRB = 1**						
Rossi P_	0.10	0.2379	1.10	[0.69; 1.76]	5.7%	5.7%
Cummings M_NY	2.21	0.8493	9.11	[1.72; 48.12]	0.4%	1.8%
MandeepR_MC	1.09	0.2011	2.96	[2.00; 4.39]	7.9%	6.1%
Docherty MB_MC	0.22	0.0782	1.24	[1.06; 1.45]	52.4%	6.9%
Fixed effect model			1.38	[1.20; 1.58]	66.4%	--
Random effects model			1.86	[1.04; 3.33]	--	20.5%
Heterogeneity: $I^2 = 86\%$, $\tau^2 = 0.2583$, $p < 0.01$						
**Fixed effect model**			**1.64**	**[1.46; 1.83]**	**100.0%**	**--**
**Random effects model**			**2.43**	**[1.89; 3.14]**	**--**	**100.0%**

Heterogeneity: $I^2 = 57\%$, $\tau^2 = 0.2403$, $p < 0.01$

Residual heterogeneity: $I^2 = 48\%$, $p < 0.01$

Candidate variable: Tuberculosis, outcome: mortality

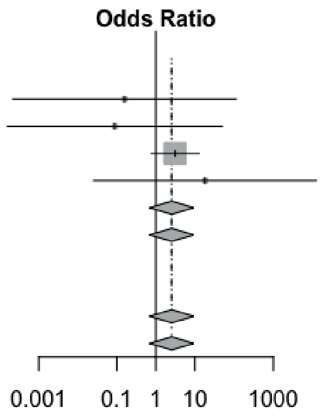

Study	TE	seTE	OR	95%-CI	Weight (fixed)	Weight (random)
ADJ = 0						
Tan L_GHCTC	-1.85	3.3545	0.16	[0.00; 112.82]	4.0%	4.0%
Zhang H_ZH	-2.43	3.2283	0.09	[0.00; 49.32]	4.3%	4.3%
LiX_TH	1.13	0.7180	3.09	[0.76; 12.63]	87.6%	87.6%
TieLong C_ZH	2.89	3.3457	18.00	[0.03; 12681.74]	4.0%	4.0%
Fixed effect model			2.53	[0.68; 9.43]	100.0%	--
Random effects model			2.53	[0.68; 9.43]	--	100.0%
Heterogeneity: $I^2 = 0\%$, $\tau^2 = 0$, $p = 0.53$						
**Fixed effect model**			**2.53**	**[0.68; 9.43]**	**100.0%**	**--**
**Random effects model**			**2.53**	**[0.68; 9.43]**	**--**	**100.0%**

Heterogeneity: $I^2 = 0\%$, $\tau^2 = 0$, $p = 0.53$

Residual heterogeneity: $I^2 = 0\%$, $p = 0.53$

Candidate variable: Cancer, solid or active haematologic cancer, outcome: mortality, subgroup analysis by risk of bias: (moderate/high vs low)

Study	TE	seTE	OR	95%-CI	Weight (fixed)	Weight (random)
LRB = 0						
Zhou F_JH	-2.03	3.2484	0.13	[0.00; 76.59]	0.0%	0.0%
Ruan Q_JY	0.90	1.2359	2.45	[0.22; 27.67]	0.3%	0.3%
Yang X_WJY	-0.49	1.4439	0.61	[0.04; 10.39]	0.3%	0.3%
Yuan M_CH	-1.73	3.3576	0.18	[0.00; 128.18]	0.0%	0.0%
Du Rong-Hui_WPH	0.95	1.1789	2.58	[0.26; 26.04]	0.4%	0.4%
Chen M_SH- WC	1.21	0.6708	3.35	[0.90; 12.46]	1.2%	1.2%
Shuai Z_UHTMC	1.11	0.6341	3.02	[0.87; 10.48]	1.3%	1.3%
Tao B_JH	1.40	0.9353	4.05	[0.65; 25.30]	0.6%	0.6%
Hu B_ZH/WPH	2.48	3.3507	12.00	[0.02; 8537.38]	0.0%	0.0%
Li J_WRCH	3.37	3.2484	28.95	[0.05; 16855.53]	0.0%	0.0%
Shi S_RHWU	-0.21	0.8360	0.81	[0.16; 4.16]	0.8%	0.8%
Zhang S_JH	0.32	0.6593	1.38	[0.38; 5.03]	1.2%	1.2%
Lang W_RH	-0.02	0.7055	0.98	[0.25; 3.91]	1.1%	1.1%
LH_SNUCM	0.02	0.3007	1.02	[0.57; 1.84]	5.8%	5.8%
Li J_CHW	1.17	0.6198	3.23	[0.96; 10.88]	1.4%	1.4%
Yin Y_MC	0.15	1.0184	1.16	[0.16; 8.54]	0.5%	0.5%
Paranjpel_MSHCS	0.42	0.2675	1.53	[0.90; 2.58]	7.3%	7.3%
HuZ_TH	2.09	1.1169	8.09	[0.91; 72.21]	0.4%	0.4%
Benelli G_HC	0.63	0.4145	1.88	[0.83; 4.23]	3.1%	3.1%
Liu Y_ZHWU	0.64	0.8214	1.89	[0.38; 9.45]	0.8%	0.8%
LiX_TH	-0.16	0.6297	0.86	[0.25; 2.94]	1.3%	1.3%
TieLong C_ZH	-0.81	1.1562	0.44	[0.05; 4.29]	0.4%	0.4%
Wang L_HPUW	0.40	0.8254	1.49	[0.30; 7.53]	0.8%	0.8%
Fixed effect model			1.54	[1.18; 2.00]	29.0%	--
Random effects model			1.54	[1.18; 2.00]	--	29.0%
Heterogeneity: $I^2 = 0\%$, $\tau^2 = 0$, $p = 0.84$						
LRB = 1						
Rossi P_	0.35	0.1884	1.43	[0.99; 2.06]	14.8%	14.8%
Docherty MB_MC	0.22	0.0967	1.24	[1.03; 1.50]	56.2%	56.2%
Fixed effect model			1.28	[1.08; 1.51]	71.0%	--
Random effects model			1.28	[1.08; 1.51]	--	71.0%
Heterogeneity: $I^2 = 0\%$, $\tau^2 = 0$, $p = 0.51$						
**Fixed effect model**			**1.35**	**[1.17; 1.55]**	**100.0%**	**--**
**Random effects model**			**1.35**	**[1.17; 1.55]**	**--**	**100.0%**

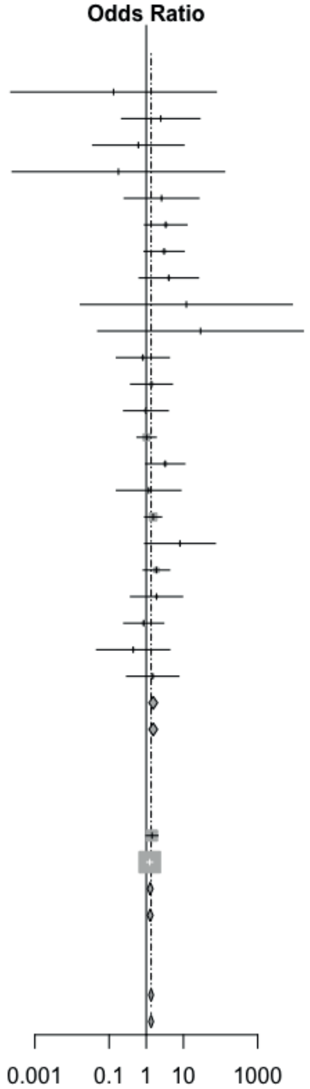

Heterogeneity: $I^2 = 0\%$, $\tau^2 = 0$, $p = 0.84$
Residual heterogeneity: $I^2 = 0\%$, $p = 0.86$

Candidate variable: Immunocompromised, outcome: mortality

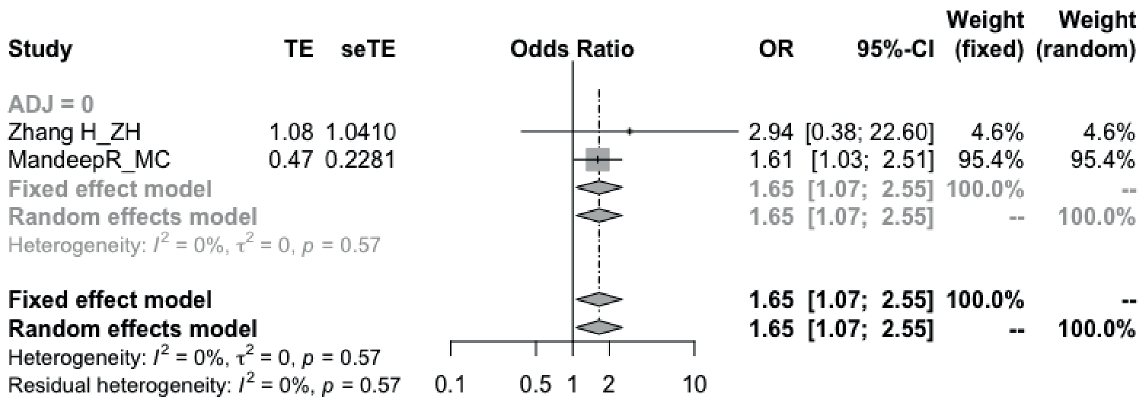

Candidate variable: Autoimmune disease, outcome: mortality

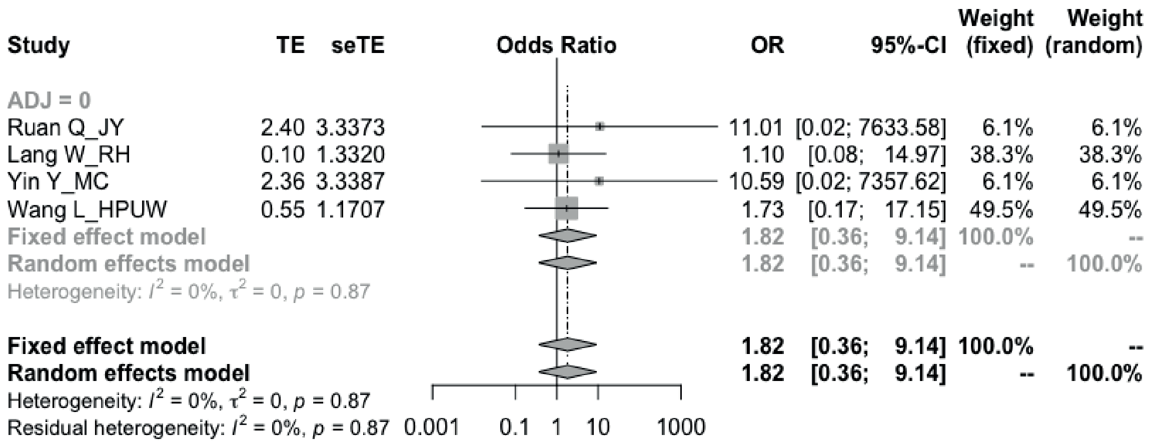

Candidate variable: Dementia, outcome: mortality, subgroup analysis by risk of bias: (moderate/high vs low)

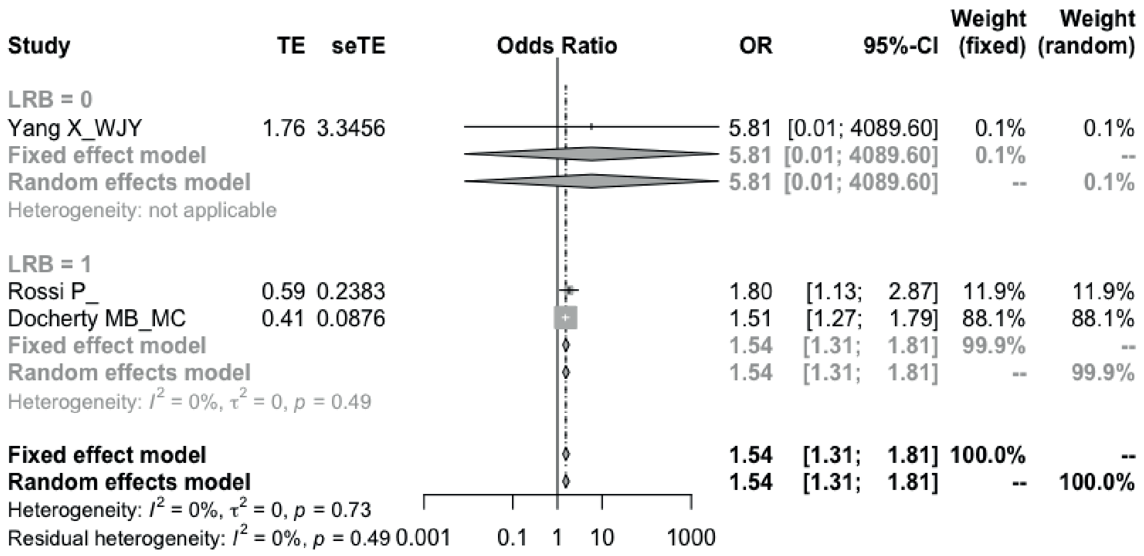

Candidate variable: Chronic liver disease, outcome: mortality

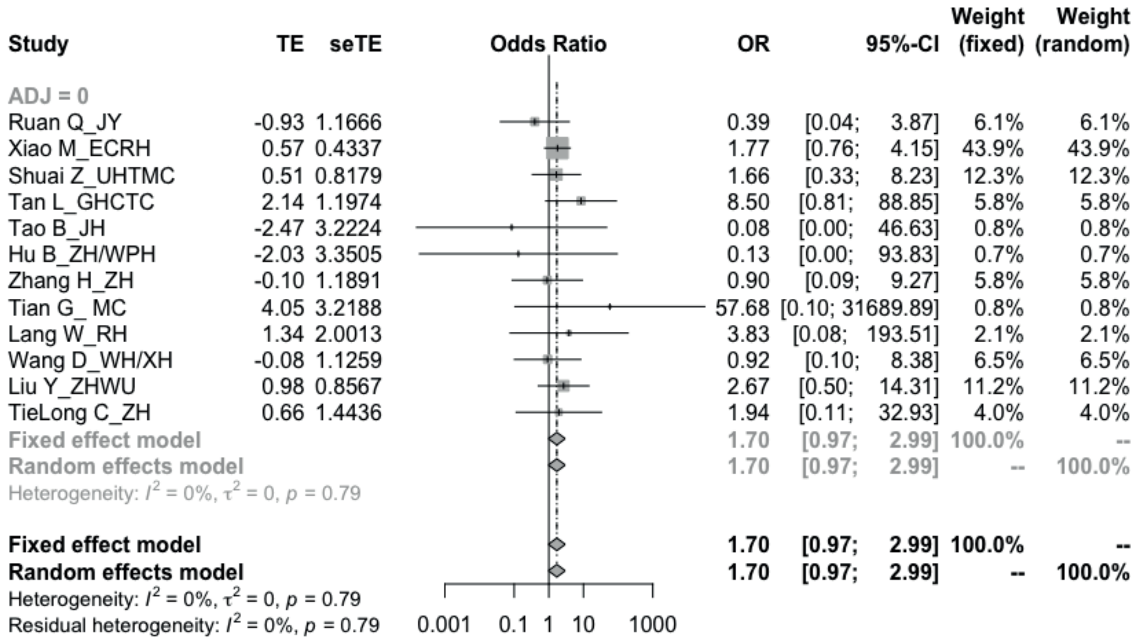

Candidate variable: chronic gastric disease (history of peptic ulcer or gastritis), outcome: mortality

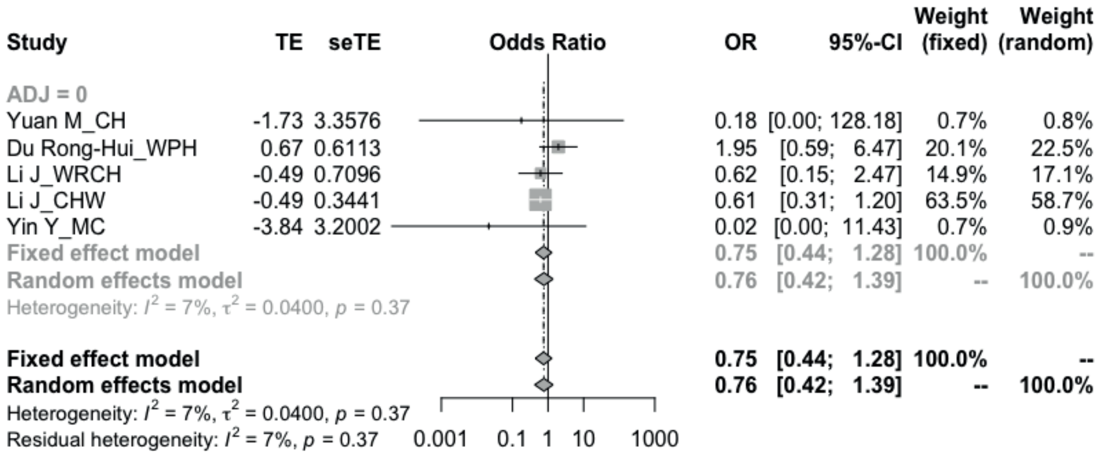

Candidate variable: Dyslipidemia, outcome: mortality, subgroup analysis: (crude vs adjusted)

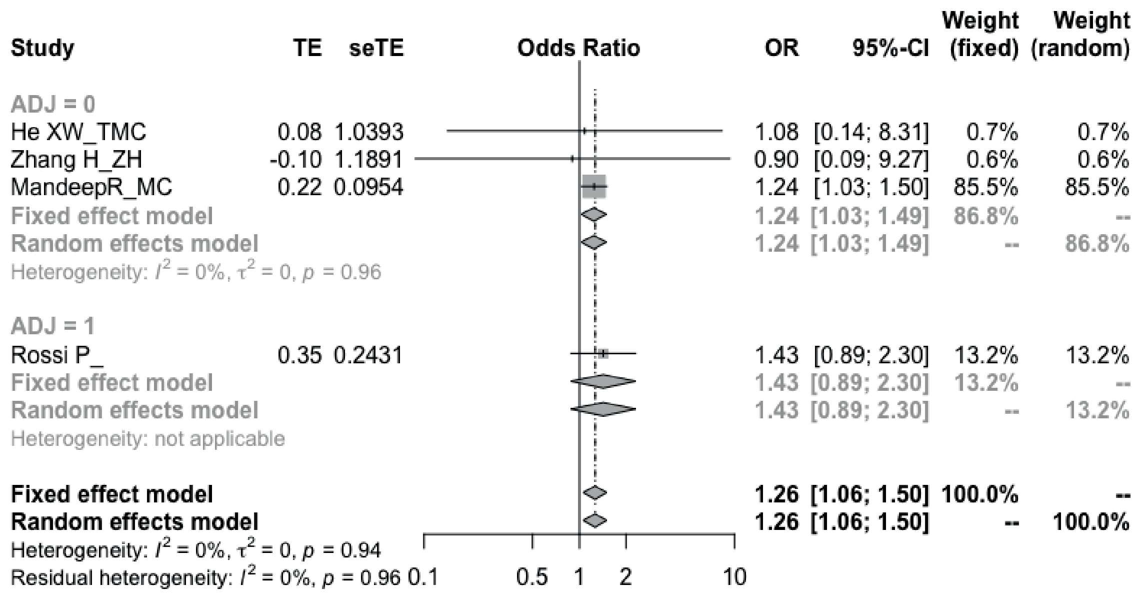

Candidate variable: Any chronic condition or comorbidities, outcome: mortality, subgroup analysis by risk of bias: (high vs moderate/low)

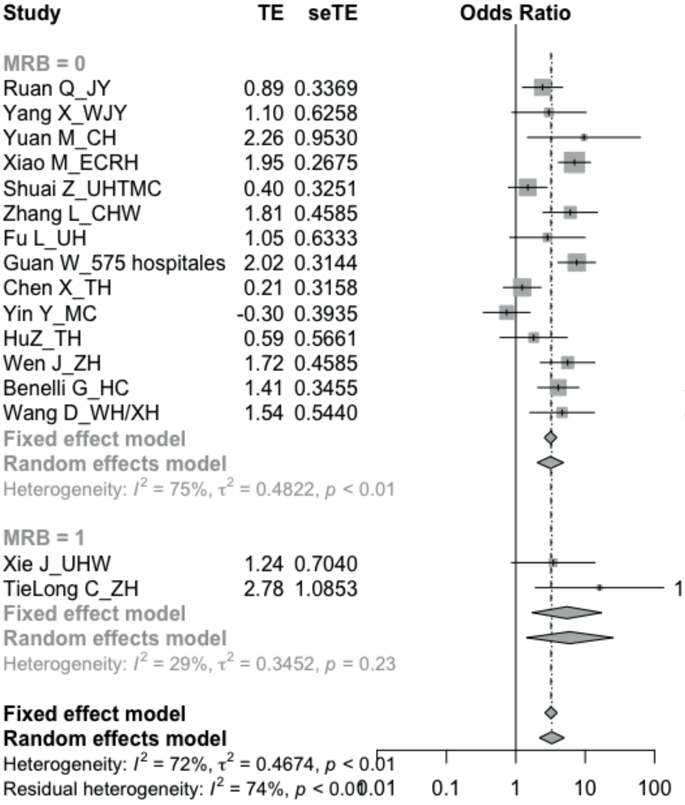

Study	TE	seTE	OR	95%-CI	Weight (fixed)	Weight (random)
**MRB = 0**						
Ruan Q_JY	0.89	0.3369	2.43	[1.25; 4.70]	9.6%	7.7%
Yang X_WJY	1.10	0.6258	3.00	[0.88; 10.23]	2.8%	5.2%
Yuan M_CH	2.26	0.9530	9.60	[1.48; 62.16]	1.2%	3.2%
Xiao M_ECRH	1.95	0.2675	7.00	[4.15; 11.83]	15.2%	8.3%
Shuai Z_UHTMC	0.40	0.3251	1.49	[0.79; 2.81]	10.3%	7.8%
Zhang L_CHW	1.81	0.4585	6.09	[2.48; 14.96]	5.2%	6.6%
Fu L_UH	1.05	0.6333	2.86	[0.83; 9.90]	2.7%	5.1%
Guan W_575 hospitales	2.02	0.3144	7.54	[4.07; 13.96]	11.0%	7.9%
Chen X_TH	0.21	0.3158	1.23	[0.66; 2.29]	10.9%	7.9%
Yin Y_MC	-0.30	0.3935	0.74	[0.34; 1.60]	7.0%	7.2%
HuZ_TH	0.59	0.5661	1.81	[0.60; 5.48]	3.4%	5.7%
Wen J_ZH	1.72	0.4585	5.58	[2.27; 13.71]	5.2%	6.6%
Benelli G_HC	1.41	0.3455	4.10	[2.08; 8.06]	9.1%	7.6%
Wang D_WH/XH	1.54	0.5440	4.64	[1.60; 13.49]	3.7%	5.9%
**Fixed effect model**			3.16	[2.57; 3.89]	96.9%	--
**Random effects model**			3.15	[2.04; 4.87]	--	92.6%
Heterogeneity: $I^2 = 75\%$, $\tau^2 = 0.4822$, $p < 0.01$						
**MRB = 1**						
Xie J_UHW	1.24	0.7040	3.46	[0.87; 13.75]	2.2%	4.6%
TieLong C_ZH	2.78	1.0853	16.10	[1.92; 135.10]	0.9%	2.7%
**Fixed effect model**			5.46	[1.71; 17.36]	3.1%	--
**Random effects model**			5.98	[1.41; 25.30]	--	7.4%
Heterogeneity: $I^2 = 29\%$, $\tau^2 = 0.3452$, $p = 0.23$						
**Fixed effect model**			**3.22**	**[2.62; 3.94]**	**100.0%**	**--**
**Random effects model**			**3.31**	**[2.18; 5.00]**	**--**	**100.0%**
Heterogeneity: $I^2 = 72\%$, $\tau^2 = 0.4674$, $p < 0.01$						
Residual heterogeneity: $I^2 = 74\%$, $p < 0.01$						

Candidate variable: Respiratory failure, outcome: mortality, subgroup analysis by risk of bias: (high vs moderate/low)

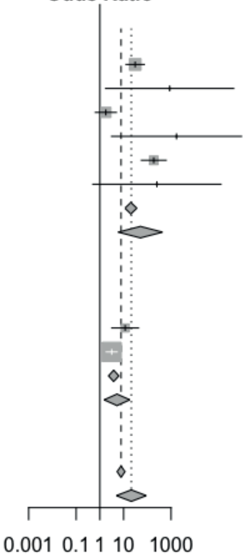

Study	TE	seTE	OR	95%-CI	Weight (fixed)	Weight (random)
**MRB = 0**						
Ruan Q_JY	3.43	0.4568	30.78	[12.58; 75.36]	18.0%	17.8%
Li J_WRCH	6.77	3.1736	872.47	[1.74; 438667.29]	0.4%	4.3%
Wen C_JH	0.58	0.5210	1.79	[0.64; 4.96]	13.8%	17.4%
Zhang H_ZH	7.44	3.2159	1702.75	[3.12; 930088.64]	0.4%	4.2%
WangY_TH	5.23	0.6134	186.81	[56.15; 621.59]	10.0%	16.9%
Xie J_UHW	5.53	3.1729	253.18	[0.50; 127110.89]	0.4%	4.3%
**Fixed effect model**			20.29	[11.36; 36.22]	42.8%	--
**Random effects model**			51.09	[5.90; 442.29]	--	64.9%
Heterogeneity: $I^2 = 87\%$, $\tau^2 = 4.5489$, $p < 0.01$						
**MRB = 1**						
Shi S_RHWU	2.46	0.6632	11.66	[3.18; 42.78]	8.5%	16.6%
LiX_TH	1.15	0.2775	3.15	[1.83; 5.43]	48.7%	18.6%
**Fixed effect model**			3.83	[2.32; 6.32]	57.2%	--
**Random effects model**			5.28	[1.51; 18.48]	--	35.1%
Heterogeneity: $I^2 = 70\%$, $\tau^2 = 0.5980$, $p = 0.07$						
**Fixed effect model**			**7.82**	**[5.35; 11.42]**	**100.0%**	**--**
**Random effects model**			**21.17**	**[4.91; 91.32]**	**--**	**100.0%**
Heterogeneity: $I^2 = 89\%$, $\tau^2 = 2.9183$, $p < 0.01$						
Residual heterogeneity: $I^2 = 86\%$, $p < 0.01$						

Candidate variable: Tachypnea, outcome: mortality, subgroup analysis by risk of bias: (high vs moderate/low)

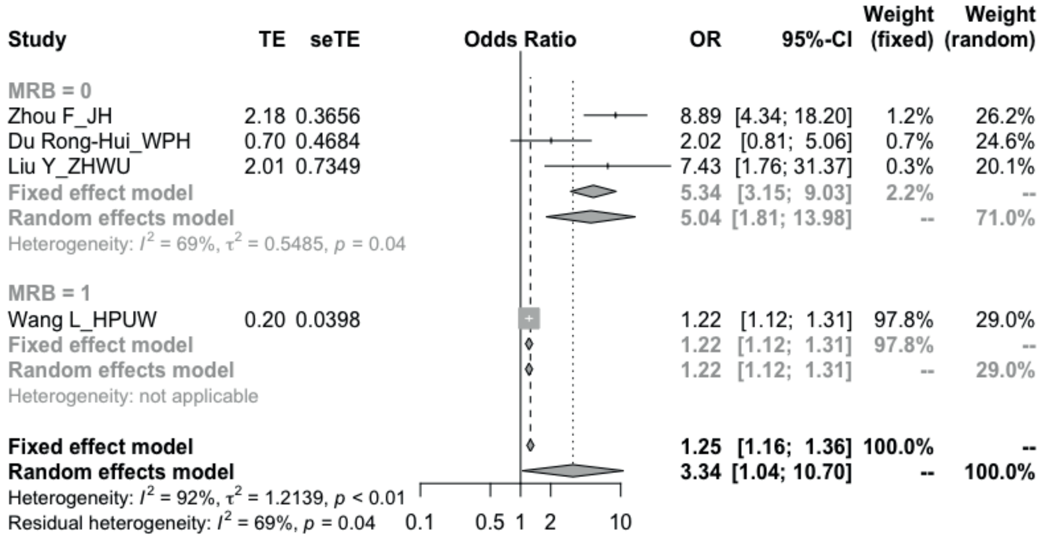

Candidate variable: Hypoxemia, outcome: mortality, subgroup analysis by risk of bias: (high vs moderate/low)

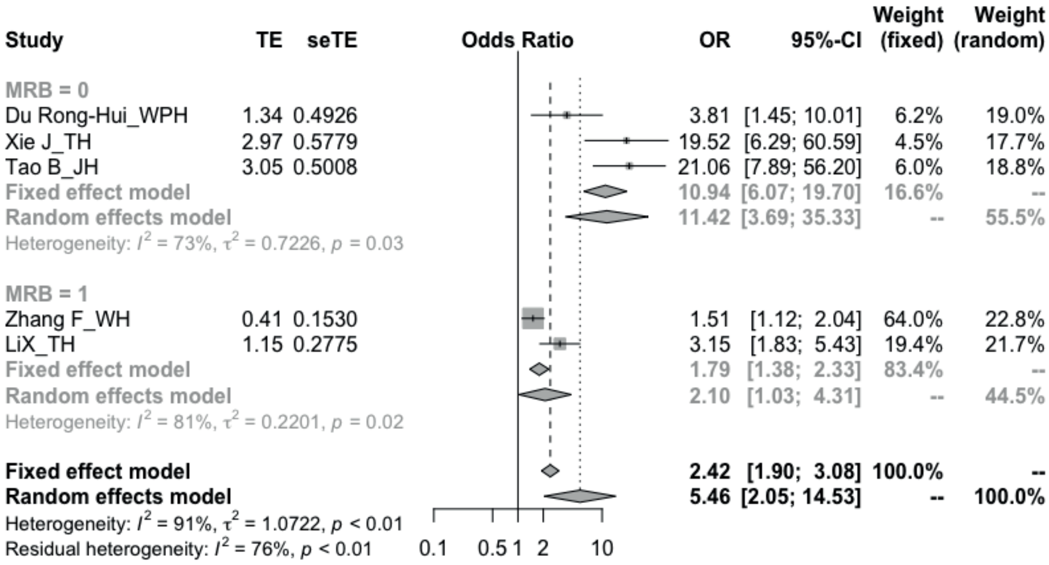

Candidate variable: Dyspnea, outcome: mortality, subgroup analysis by risk of bias: (high vs moderate/low)

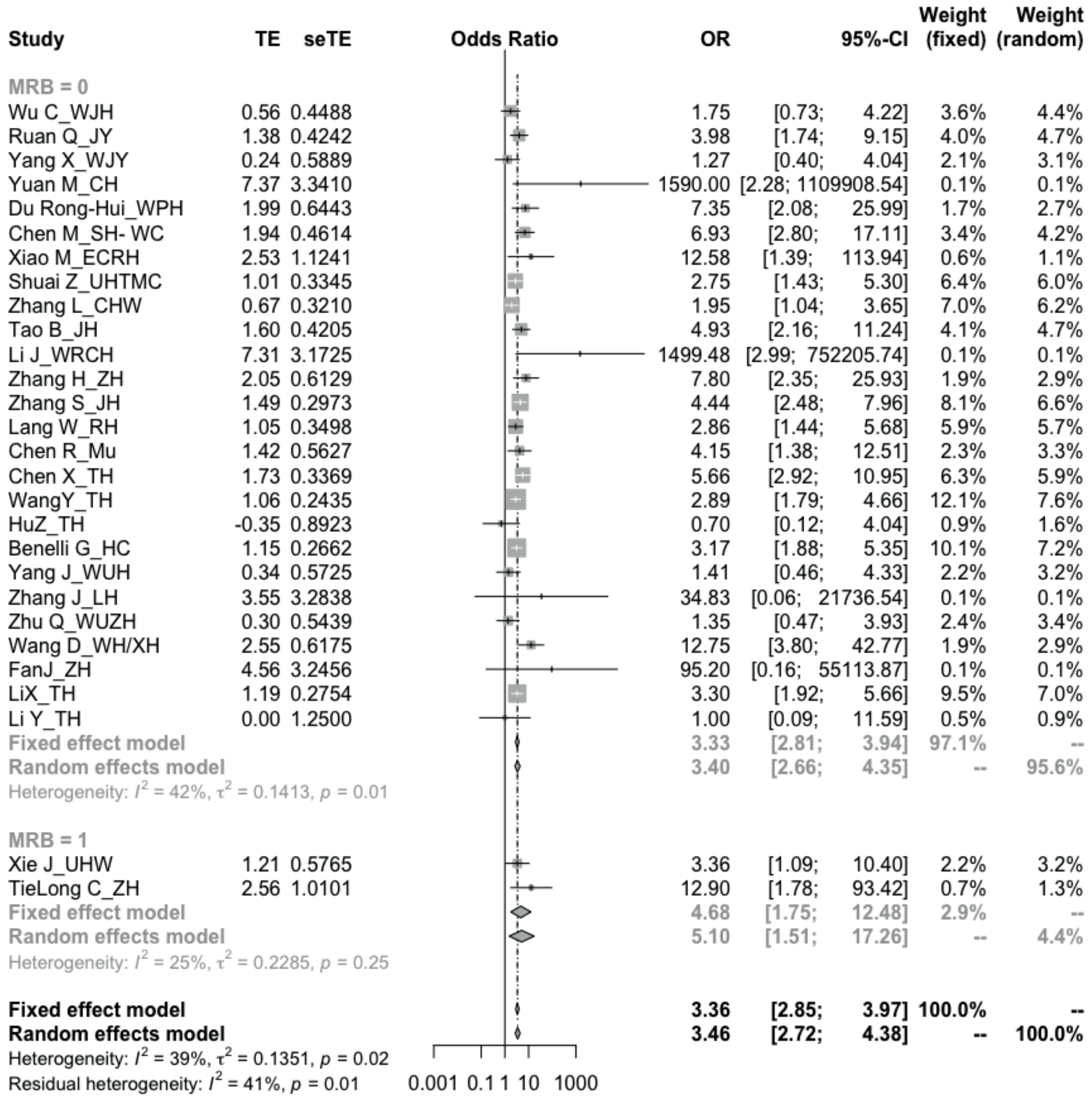

Candidate variable: Chest pain, outcome: mortality, subgroup analysis: (crude vs adjusted)

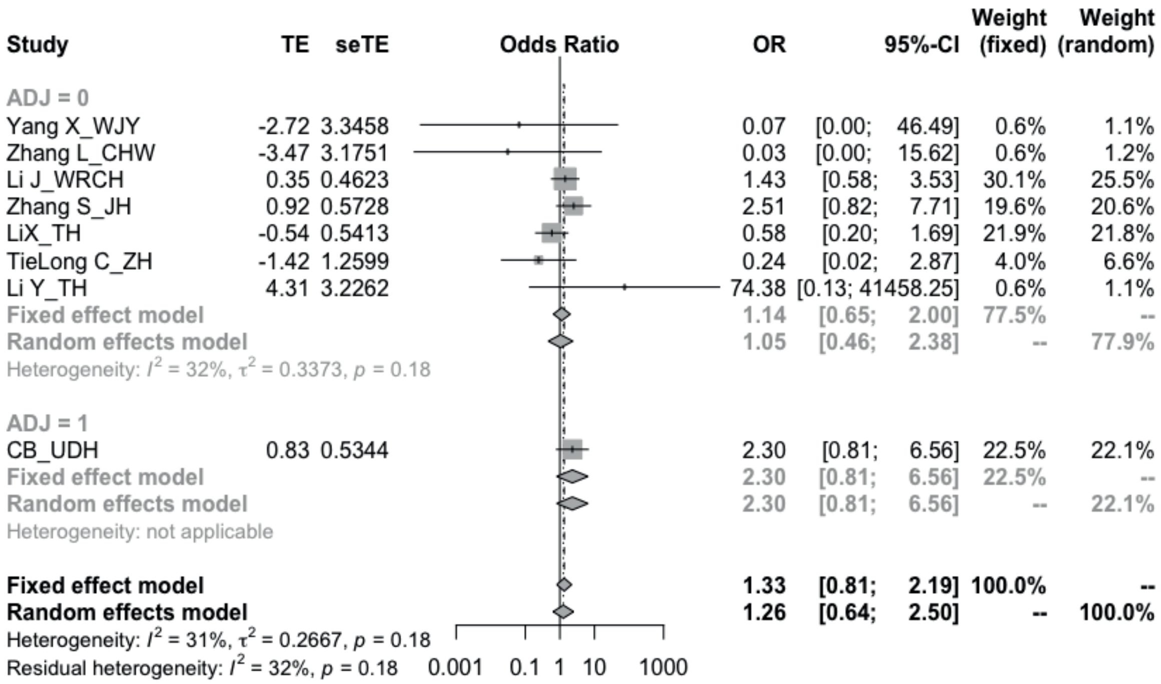

Candidate variable: Tachycardia, outcome: mortality,

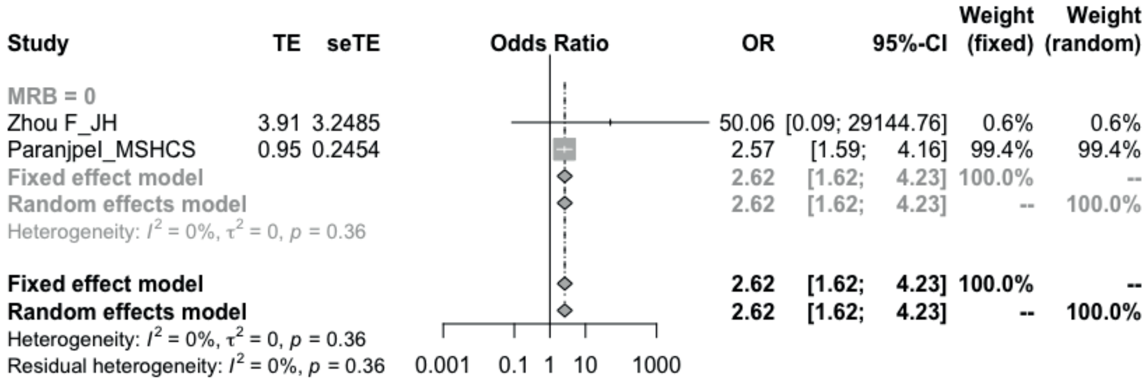

Candidate variable: Low blood pressure, outcome: mortality

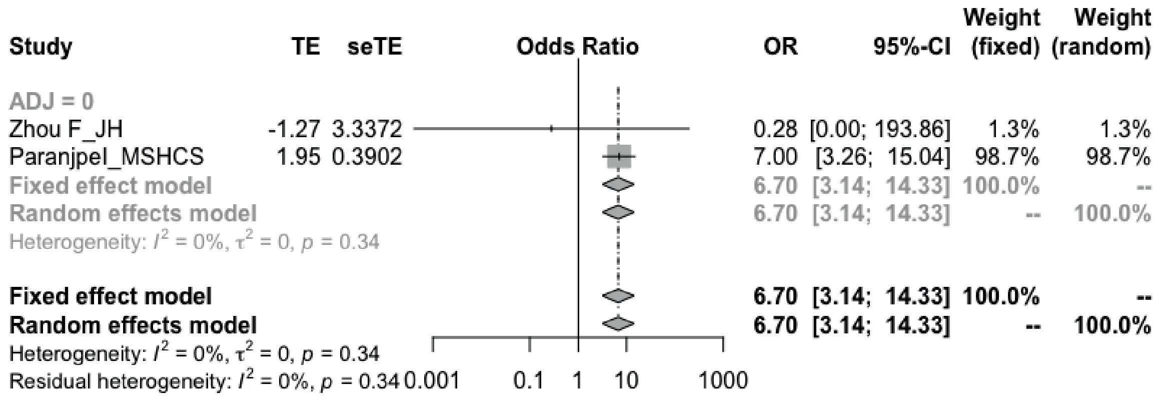

Candidate variable: High fever (more than 39°C), outcome: mortality

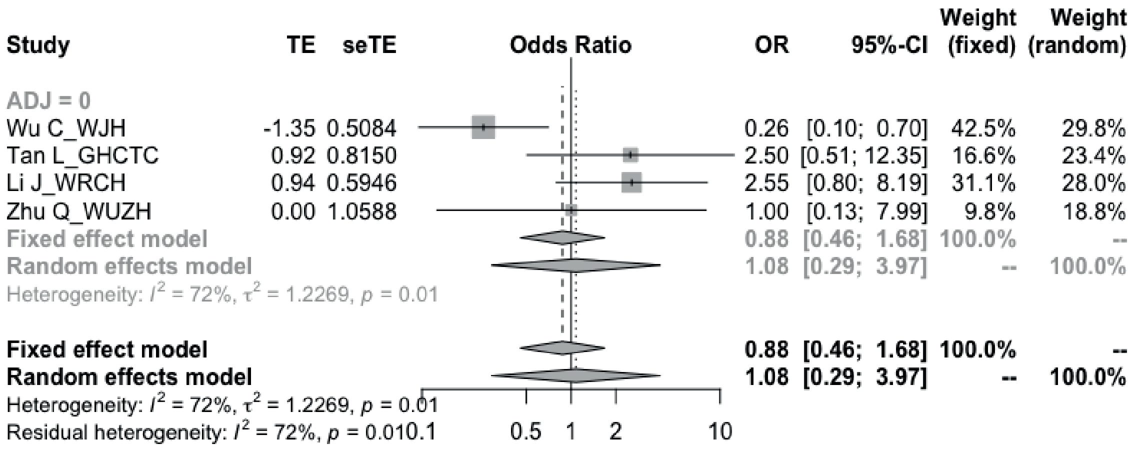

Candidate variable: Fever, outcome: mortality, subgroup analysis: (crude vs adjusted)

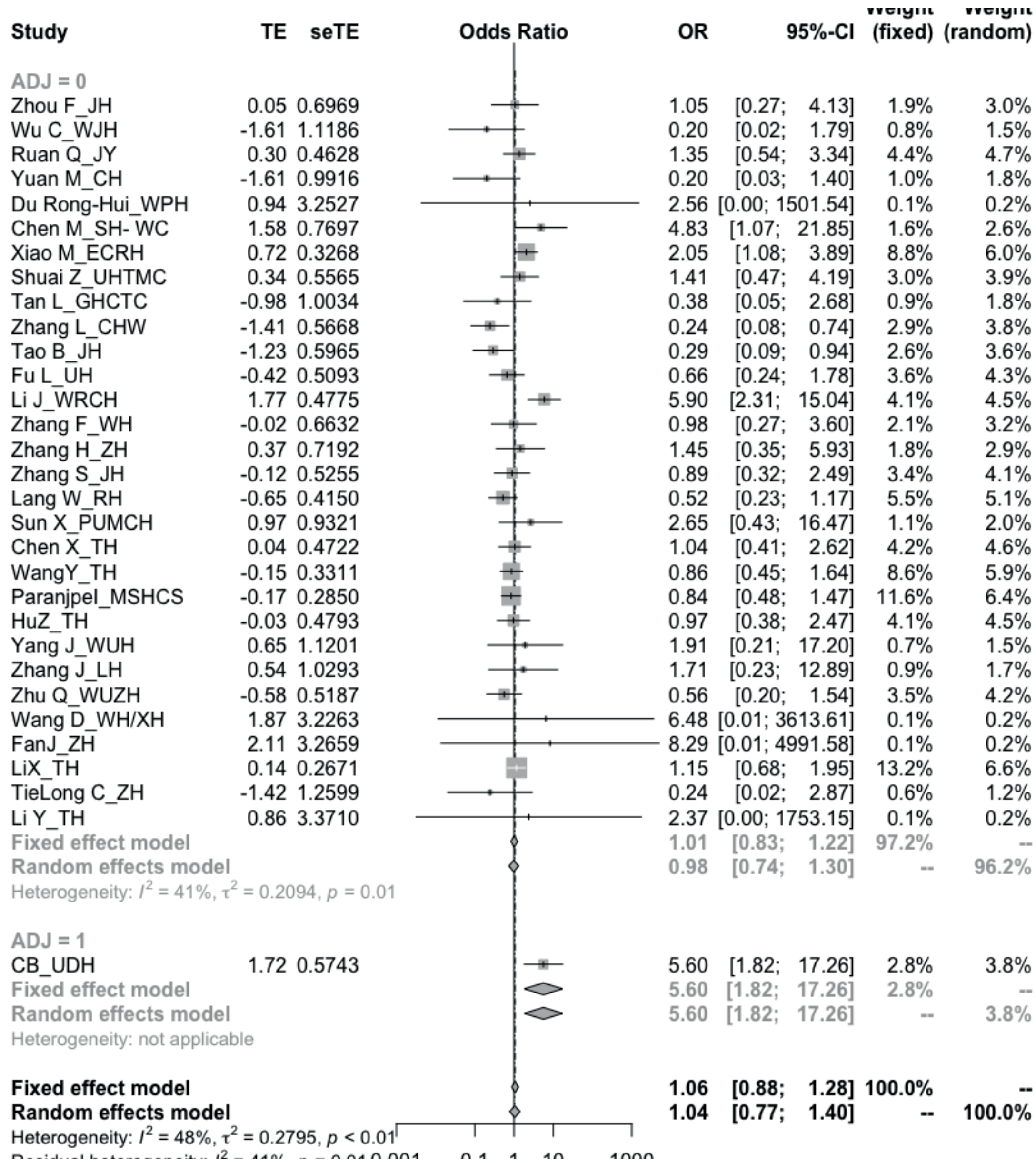

Candidate variable: Rhinorrhea, outcome: mortality

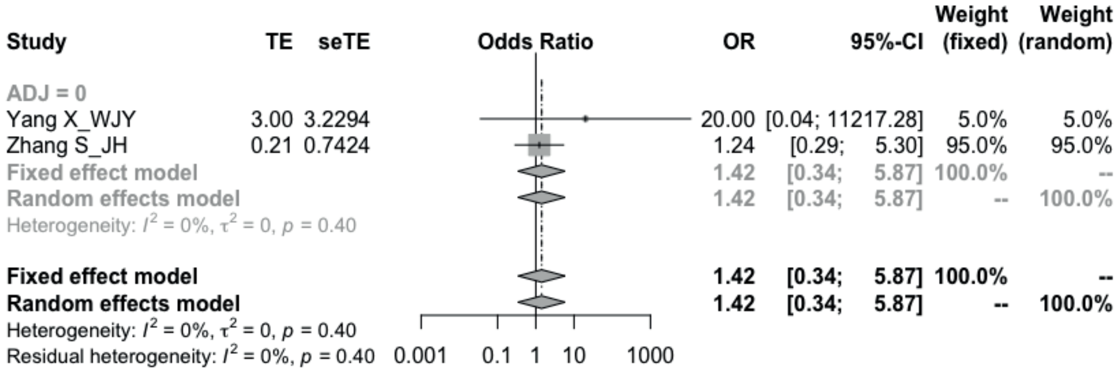

Candidate variable: Odynophagia, outcome: mortality

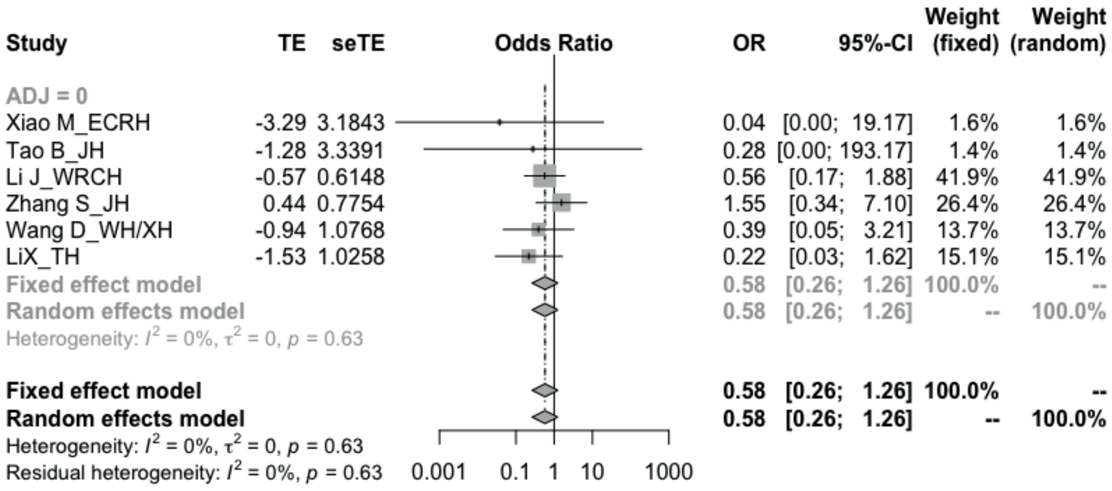

Candidate variable: cough, outcome: mortality, subgroup analysis by risk of bias: (high vs moderate/low)

Study	TE	seTE	OR	95%-CI	Weight (fixed)	Weight (random)
**MRB = 0**						
Zhou F_JH	-0.54	0.3758	0.58	[0.28; 1.21]	4.7%	4.5%
Wu C_WJH	-0.85	0.5914	0.43	[0.13; 1.37]	1.9%	2.7%
Ruan Q_JY	0.16	0.3726	1.17	[0.56; 2.43]	4.7%	4.6%
Yang X_WJY	0.17	0.6705	1.19	[0.32; 4.43]	1.5%	2.2%
Yuan M_CH	-0.61	0.8109	0.55	[0.11; 2.67]	1.0%	1.7%
Du Rong-Hui_WPH	-0.93	0.5102	0.39	[0.14; 1.07]	2.5%	3.2%
Chen M_SH- WC	0.12	0.4713	1.13	[0.45; 2.85]	3.0%	3.6%
Xiao M_ECRH	-1.21	0.5396	0.30	[0.10; 0.86]	2.3%	3.0%
Shuai Z_UHTMC	-0.62	0.3221	0.54	[0.29; 1.01]	6.3%	5.2%
Tan L_GHCTC	0.22	0.7472	1.25	[0.29; 5.41]	1.2%	1.9%
Zhang L_CHW	0.71	0.3227	2.04	[1.08; 3.83]	6.3%	5.2%
Tao B_JH	0.05	0.3989	1.05	[0.48; 2.29]	4.1%	4.3%
Fu L_UH	-0.13	0.3825	0.87	[0.41; 1.85]	4.5%	4.5%
Li J_WRCH	-0.19	0.3402	0.83	[0.43; 1.62]	5.7%	5.0%
Zhang H_ZH	-0.17	0.6223	0.84	[0.25; 2.86]	1.7%	2.5%
Zhang S_JH	-0.09	0.3088	0.91	[0.50; 1.67]	6.9%	5.4%
Sun X_PUMCH	-0.18	0.8442	0.84	[0.16; 4.37]	0.9%	1.6%
Chen X_TH	0.00	0.4490	1.00	[0.41; 2.41]	3.3%	3.8%
WangY_TH	0.34	0.2414	1.40	[0.87; 2.25]	11.3%	6.3%
HuZ_TH	0.62	0.6454	1.85	[0.52; 6.57]	1.6%	2.4%
Benelli G_HC	-1.76	0.3908	0.17	[0.08; 0.37]	4.3%	4.4%
Yang J_WUH	1.05	0.6994	2.84	[0.72; 11.20]	1.3%	2.1%
Zhang J_LH	-0.33	0.9487	0.72	[0.11; 4.62]	0.7%	1.3%
Zhu Q_WUZH	-0.83	0.4892	0.43	[0.17; 1.13]	2.8%	3.4%
Wang D_WH/XH	-0.24	0.5148	0.79	[0.29; 2.16]	2.5%	3.2%
FanJ_ZH	0.81	3.3798	2.25	[0.00; 1694.69]	0.1%	0.1%
LiX_TH	-0.01	0.2849	0.99	[0.57; 1.74]	8.1%	5.7%
TieLong C_ZH	0.99	0.6565	2.68	[0.74; 9.70]	1.5%	2.3%
Li Y_TH	0.77	1.2123	2.15	[0.20; 23.18]	0.4%	0.8%
**Fixed effect model**			0.88	[0.75; 1.03]	97.2%	--
**Random effects model**			0.85	[0.67; 1.08]	--	96.5%
Heterogeneity: $I^2 = 46\%$, $\tau^2 = 0.1688$, $p < 0.01$						
**MRB = 1**						
CB_UDH	-0.22	0.4840	0.80	[0.31; 2.07]	2.8%	3.5%
**Fixed effect model**			0.80	[0.31; 2.07]	2.8%	--
**Random effects model**			0.80	[0.31; 2.07]	--	3.5%
Heterogeneity: not applicable						
**Fixed effect model**			**0.87**	**[0.74; 1.02]**	**100.0%**	**--**
**Random effects model**			**0.85**	**[0.68; 1.07]**	**--**	**100.0%**

Heterogeneity: $I^2 = 44\%$, $\tau^2 = 0.1569$, $p < 0.01$

Residual heterogeneity: $I^2 = 46\%$, $p < 0.01$

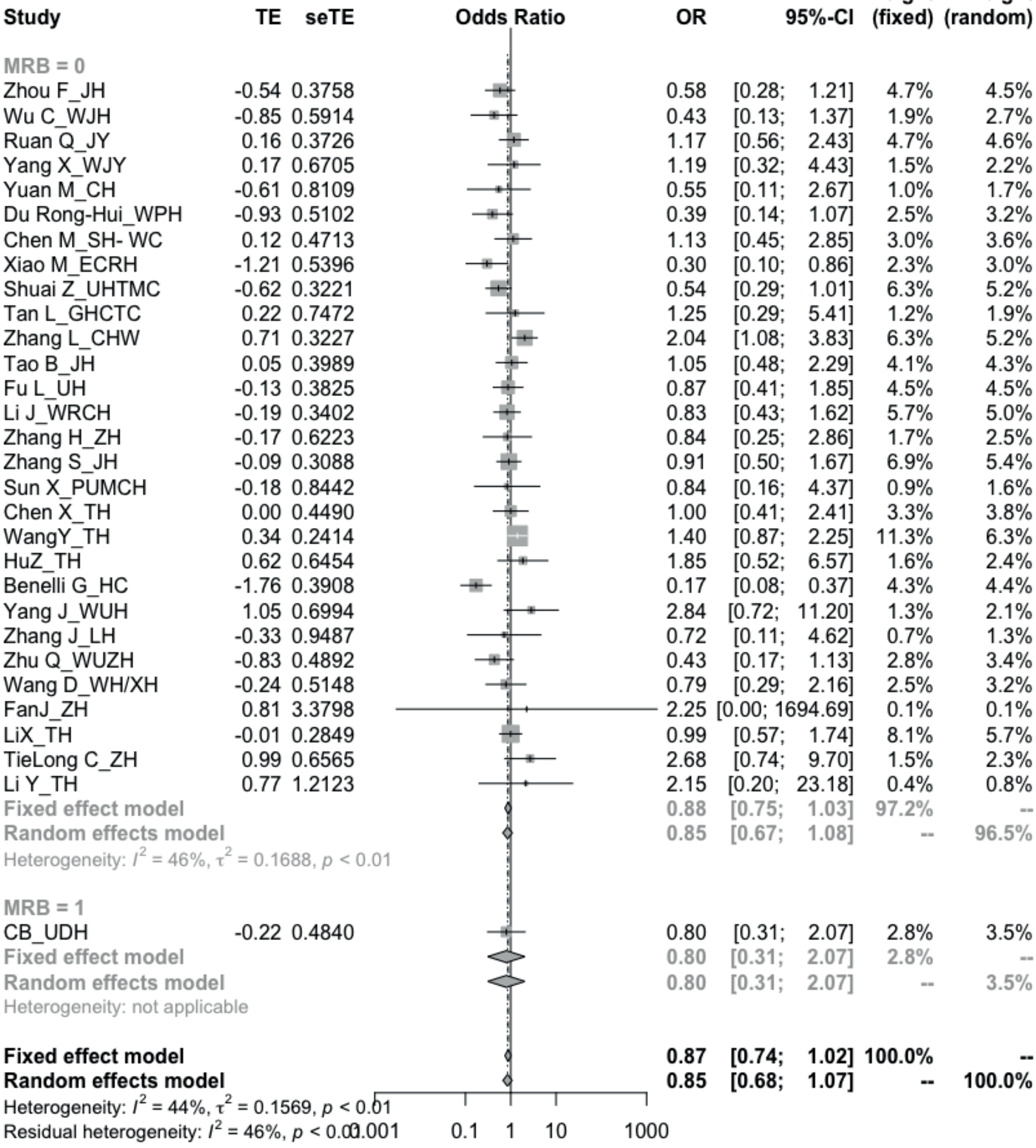

Candidate variable: productive cough, outcome: mortality, subgroup analysis: (crude vs adjusted)

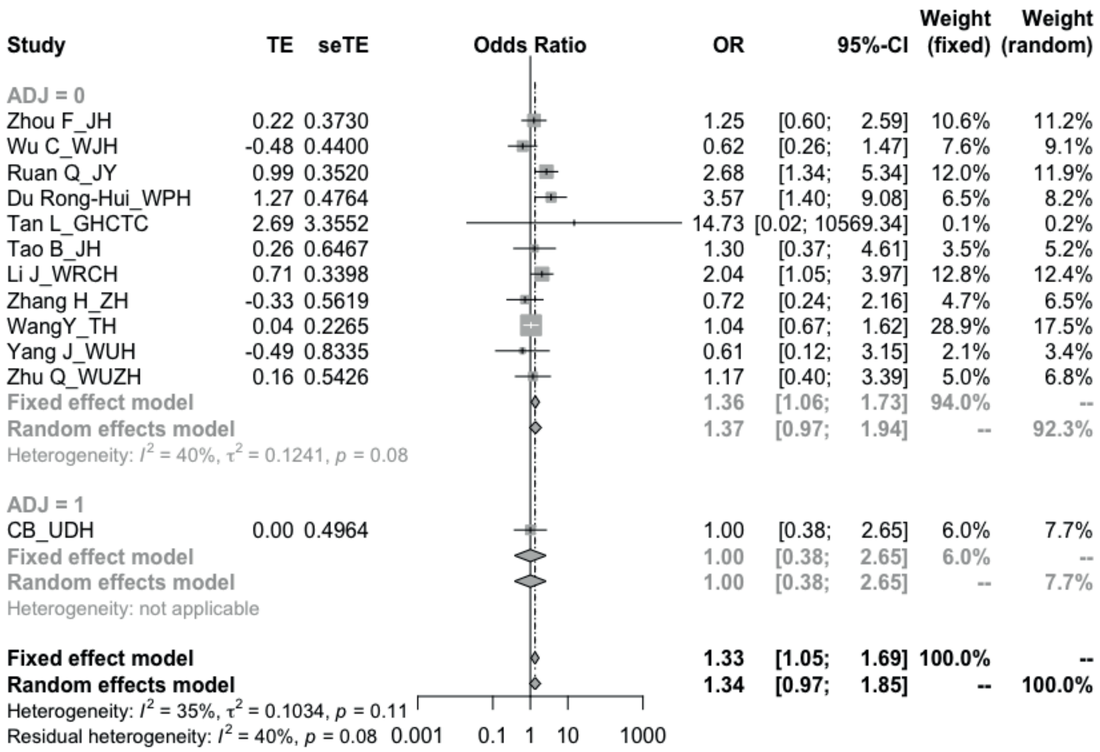

Candidate variable: hemoptysis, outcome: mortality

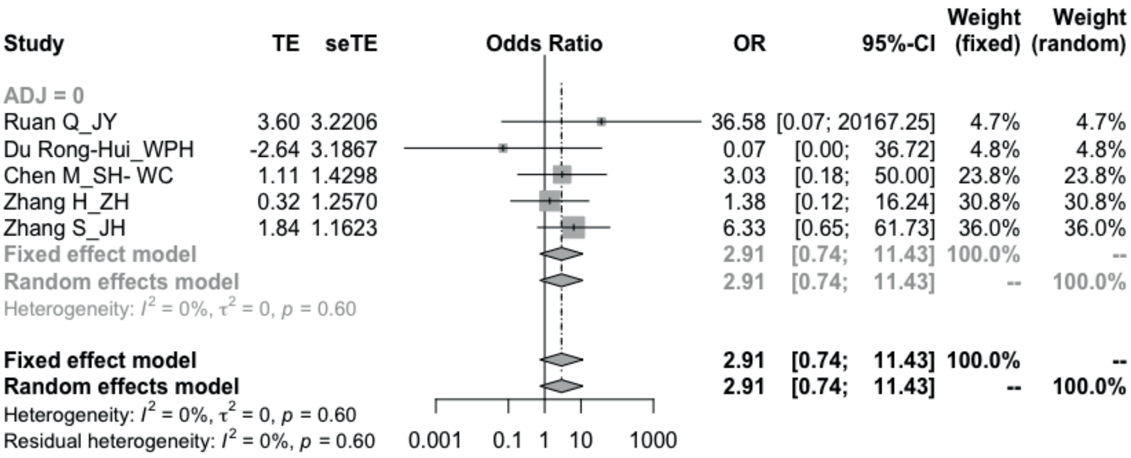

Candidate variable: fatigue, outcome: mortality

Study	TE	seTE	OR	95%-CI	Weight (fixed)	Weight (random)
ADJ = 0						
Zhou F_JH	0.36	0.3688	1.43	[0.70; 2.95]	6.5%	7.1%
Ruan Q_JY	-0.26	0.3843	0.77	[0.36; 1.64]	6.0%	6.8%
Du Rong-Hui_WPH	1.03	0.4779	2.80	[1.10; 7.14]	3.9%	5.2%
Chen M_SH- WC	0.56	0.4829	1.74	[0.68; 4.49]	3.8%	5.2%
Xiao M_ECRH	-0.52	0.7833	0.59	[0.13; 2.76]	1.4%	2.5%
Tan L_GHCTC	-2.66	3.2668	0.07	[0.00; 42.35]	0.1%	0.2%
Zhang L_CHW	0.54	0.3202	1.72	[0.92; 3.22]	8.6%	8.1%
Tao B_JH	-0.71	1.1136	0.49	[0.06; 4.36]	0.7%	1.4%
Fu L_UH	0.11	0.3796	1.12	[0.53; 2.36]	6.1%	6.9%
Li J_WRCH	1.95	0.4133	7.05	[3.14; 15.85]	5.2%	6.3%
Zhang H_ZH	0.91	0.5654	2.48	[0.82; 7.52]	2.8%	4.2%
Zhang S_JH	0.85	0.2692	2.34	[1.38; 3.97]	12.2%	9.4%
WangY_TH	0.02	0.2215	1.02	[0.66; 1.58]	18.0%	10.6%
Yang J_WUH	0.66	0.5780	1.94	[0.63; 6.04]	2.6%	4.0%
Zhang J_LH	-2.83	3.2806	0.06	[0.00; 36.71]	0.1%	0.2%
Zhu Q_WUZH	0.56	0.4968	1.76	[0.66; 4.65]	3.6%	5.0%
Wang D_WH/XH	0.77	0.6075	2.16	[0.66; 7.12]	2.4%	3.7%
FanJ_ZH	3.07	3.2419	21.45	[0.04; 12332.26]	0.1%	0.2%
LiX_TH	0.68	0.2505	1.98	[1.21; 3.24]	14.1%	9.9%
TieLong C_ZH	0.26	0.9604	1.29	[0.20; 8.50]	1.0%	1.8%
Li Y_TH	-0.44	1.0351	0.64	[0.08; 4.89]	0.8%	1.5%
Fixed effect model			1.64	[1.36; 1.97]	100.0%	--
Random effects model			1.66	[1.27; 2.17]	--	100.0%
Heterogeneity: I^2 = 39%, τ^2 = 0.1250, p = 0.04						
**Fixed effect model**			**1.64**	**[1.36; 1.97]**	**100.0%**	**--**
**Random effects model**			**1.66**	**[1.27; 2.17]**	**--**	**100.0%**

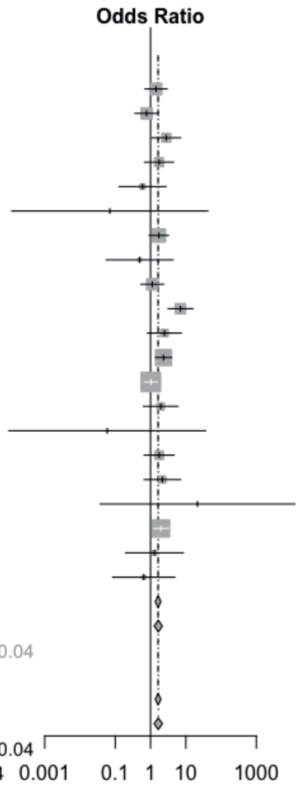

Heterogeneity: I^2 = 39%, τ^2 = 0.1250, p = 0.04
Residual heterogeneity: I^2 = 39%, p = 0.04

Candidate variable: myalgia/arthralgia, outcome: mortality, subgroup analysis: (crude vs adjusted)

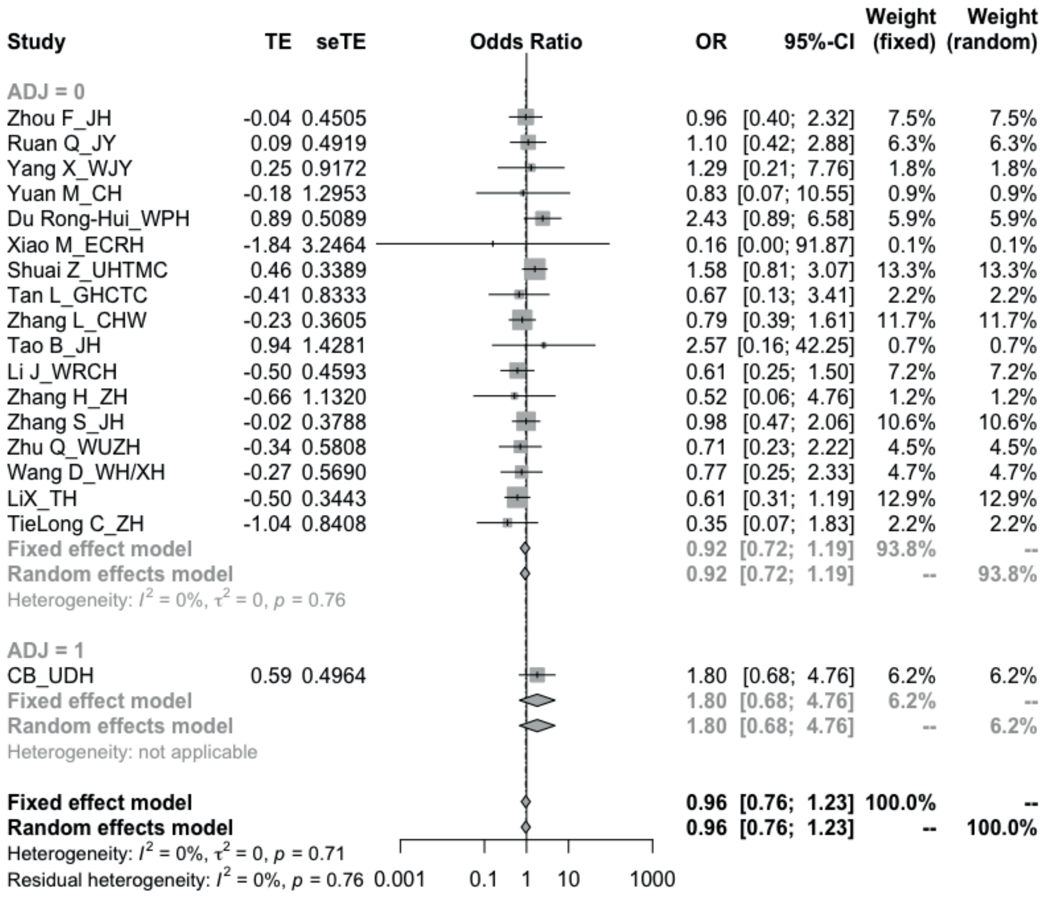

Candidate variable: headache, outcome: mortality, subgroup analysis: (crude vs adjusted)

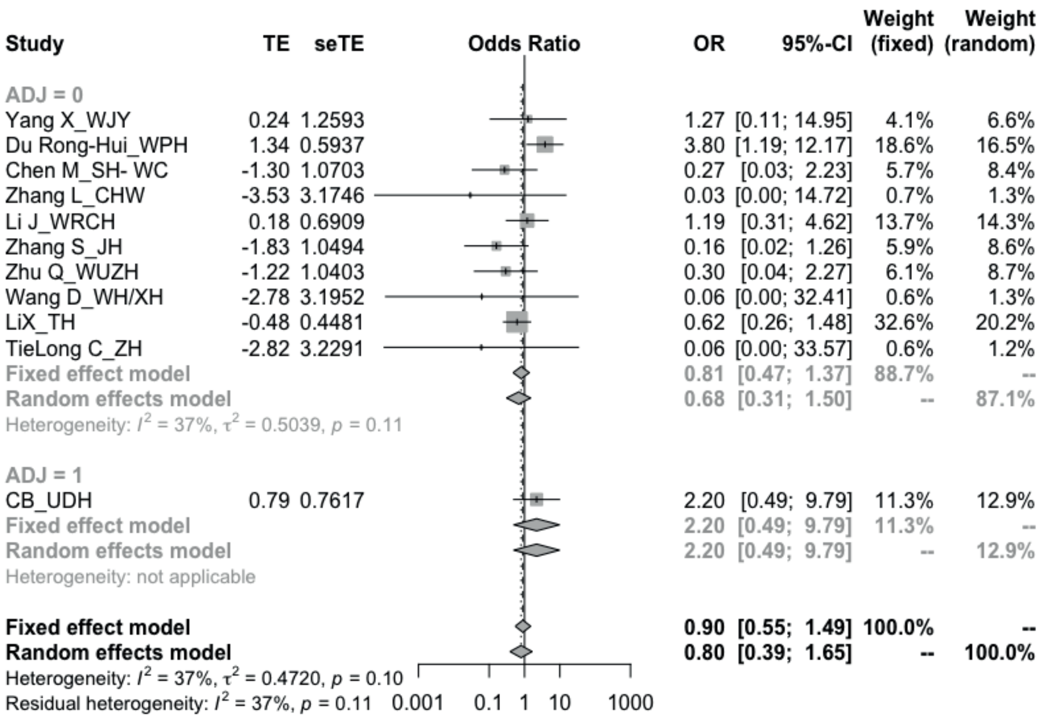

Candidate variable: vomits, outcome: mortality

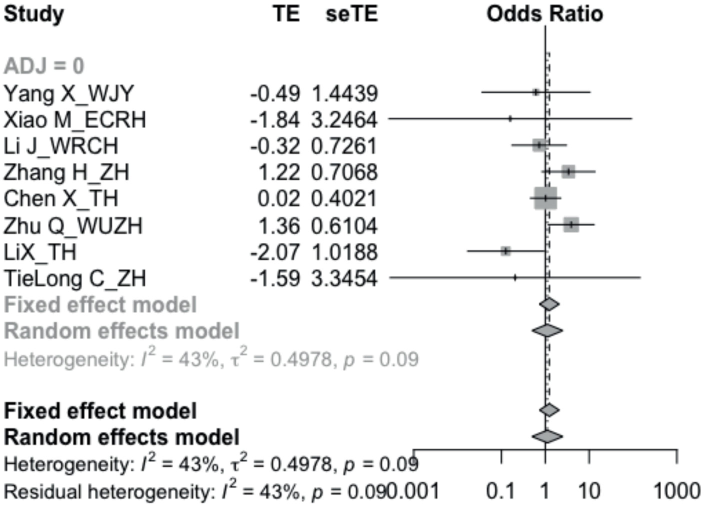

Study	TE	seTE	OR	95%-CI	Weight (fixed)	Weight (random)
ADJ = 0						
Yang X_WJY	-0.49	1.4439	0.61	[0.04; 10.39]	3.3%	6.6%
Xiao M_ECRH	-1.84	3.2464	0.16	[0.00; 91.87]	0.7%	1.5%
Li J_WRCH	-0.32	0.7261	0.73	[0.17; 3.01]	13.2%	16.7%
Zhang H_ZH	1.22	0.7068	3.38	[0.85; 13.53]	13.9%	17.1%
Chen X_TH	0.02	0.4021	1.02	[0.46; 2.24]	43.0%	25.9%
Zhu Q_WUZH	1.36	0.6104	3.89	[1.18; 12.87]	18.6%	19.6%
LiX_TH	-2.07	1.0188	0.13	[0.02; 0.93]	6.7%	11.1%
TieLong C_ZH	-1.59	3.3454	0.20	[0.00; 144.11]	0.6%	1.5%
Fixed effect model			1.24	[0.74; 2.07]	100.0%	--
Random effects model			1.12	[0.50; 2.51]	--	100.0%
Heterogeneity: $I^2 = 43\%$, $\tau^2 = 0.4978$, $p = 0.09$						
**Fixed effect model**			**1.24**	**[0.74; 2.07]**	**100.0%**	**--**
**Random effects model**			**1.12**	**[0.50; 2.51]**	**--**	**100.0%**

Heterogeneity: $I^2 = 43\%$, $\tau^2 = 0.4978$, $p = 0.09$
Residual heterogeneity: $I^2 = 43\%$, $p = 0.09$

Candidate variable: diarrhea, outcome: mortality

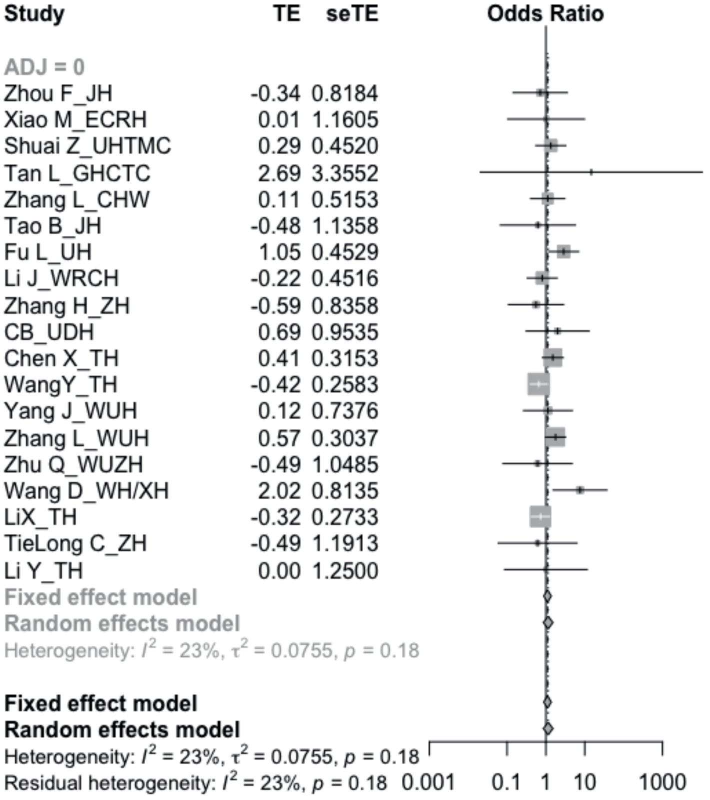

Study	TE	seTE	OR	95%-CI	Weight (fixed)	Weight (random)
ADJ = 0						
Zhou F_JH	-0.34	0.8184	0.71	[0.14; 3.55]	1.9%	2.7%
Xiao M_ECRH	0.01	1.1605	1.01	[0.10; 9.82]	0.9%	1.4%
Shuai Z_UHTMC	0.29	0.4520	1.34	[0.55; 3.24]	6.2%	7.3%
Tan L_GHCTC	2.69	3.3552	14.73	[0.02; 10569.34]	0.1%	0.2%
Zhang L_CHW	0.11	0.5153	1.11	[0.40; 3.05]	4.8%	5.9%
Tao B_JH	-0.48	1.1358	0.62	[0.07; 5.76]	1.0%	1.5%
Fu L_UH	1.05	0.4529	2.86	[1.18; 6.95]	6.2%	7.2%
Li J_WRCH	-0.22	0.4516	0.80	[0.33; 1.95]	6.2%	7.3%
Zhang H_ZH	-0.59	0.8358	0.56	[0.11; 2.86]	1.8%	2.6%
CB_UDH	0.69	0.9535	2.00	[0.31; 12.96]	1.4%	2.1%
Chen X_TH	0.41	0.3153	1.51	[0.81; 2.80]	12.7%	11.6%
WangY_TH	-0.42	0.2583	0.66	[0.39; 1.09]	19.0%	14.3%
Yang J_WUH	0.12	0.7376	1.13	[0.27; 4.79]	2.3%	3.3%
Zhang L_WUH	0.57	0.3037	1.77	[0.98; 3.21]	13.7%	12.1%
Zhu Q_WUZH	-0.49	1.0485	0.61	[0.08; 4.76]	1.2%	1.7%
Wang D_WH/XH	2.02	0.8135	7.56	[1.53; 37.21]	1.9%	2.8%
LiX_TH	-0.32	0.2733	0.73	[0.43; 1.24]	17.0%	13.5%
TieLong C_ZH	-0.49	1.1913	0.61	[0.06; 6.31]	0.9%	1.4%
Li Y_TH	0.00	1.2500	1.00	[0.09; 11.59]	0.8%	1.2%
Fixed effect model			1.10	[0.88; 1.37]	100.0%	--
Random effects model			1.14	[0.86; 1.51]	--	100.0%
Heterogeneity: $I^2 = 23\%$, $\tau^2 = 0.0755$, $p = 0.18$						
**Fixed effect model**			**1.10**	**[0.88; 1.37]**	**100.0%**	**--**
**Random effects model**			**1.14**	**[0.86; 1.51]**	**--**	**100.0%**

Heterogeneity: $I^2 = 23\%$, $\tau^2 = 0.0755$, $p = 0.18$
Residual heterogeneity: $I^2 = 23\%$, $p = 0.18$

Candidate variable: anorexia, outcome: mortality, subgroup analysis: (crude vs adjusted)

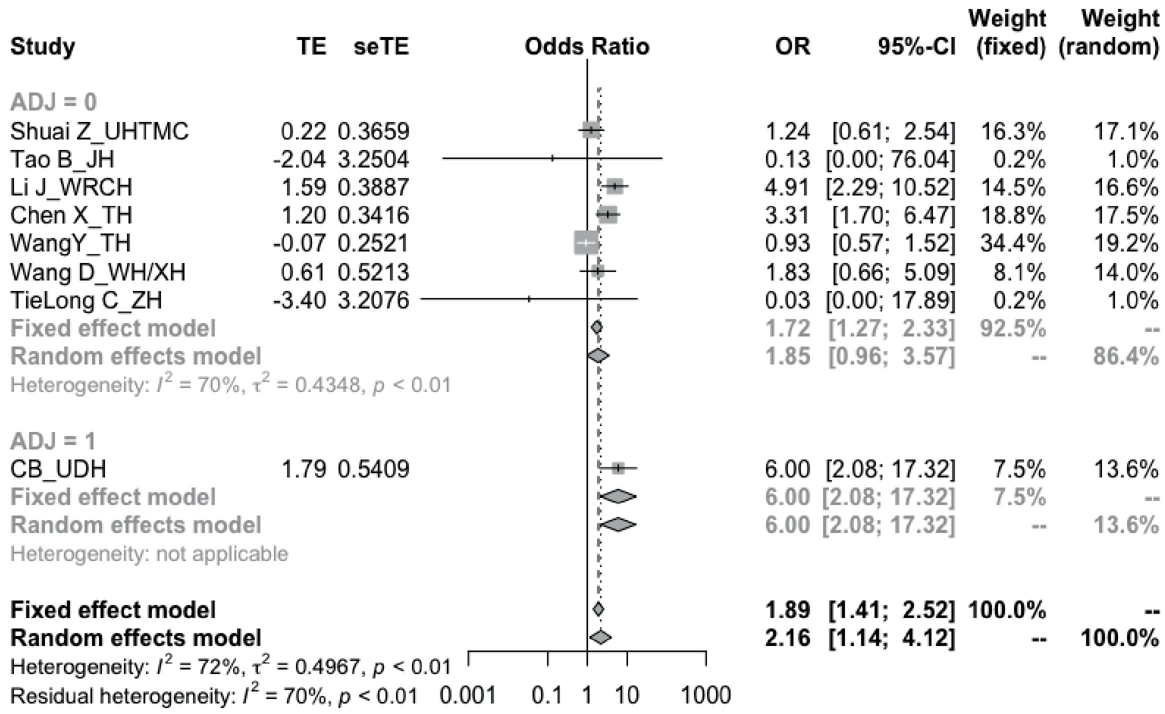

Candidate variable: abdominal pain, outcome: mortality

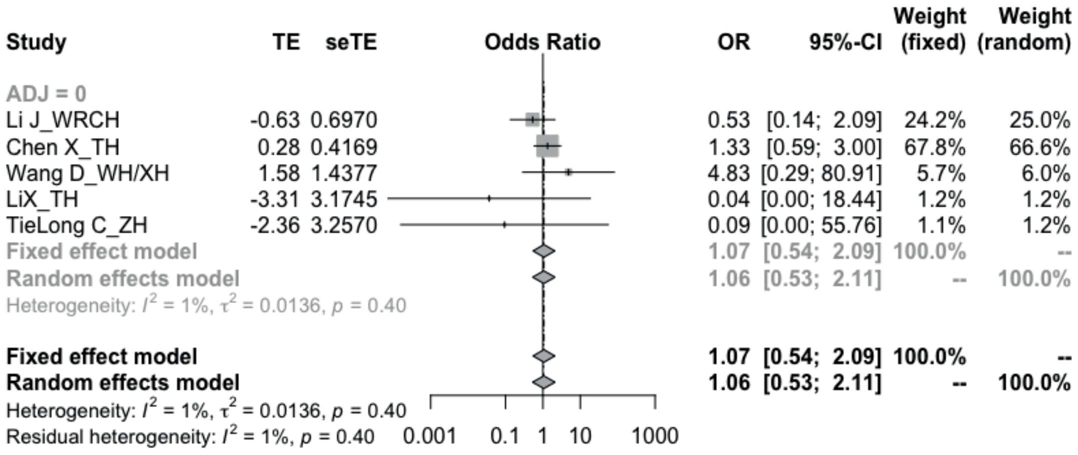

Candidate variable: anemia, outcome: mortality

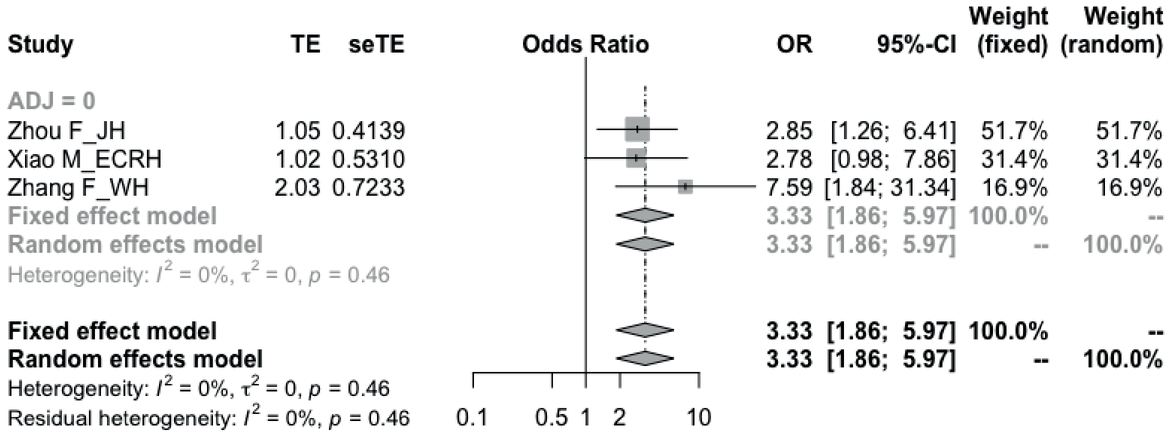

Candidate variable: High WBC (greater than 10.0 x 109/L), outcome: mortality, subgroup analysis by risk of bias: (high vs moderate/low)

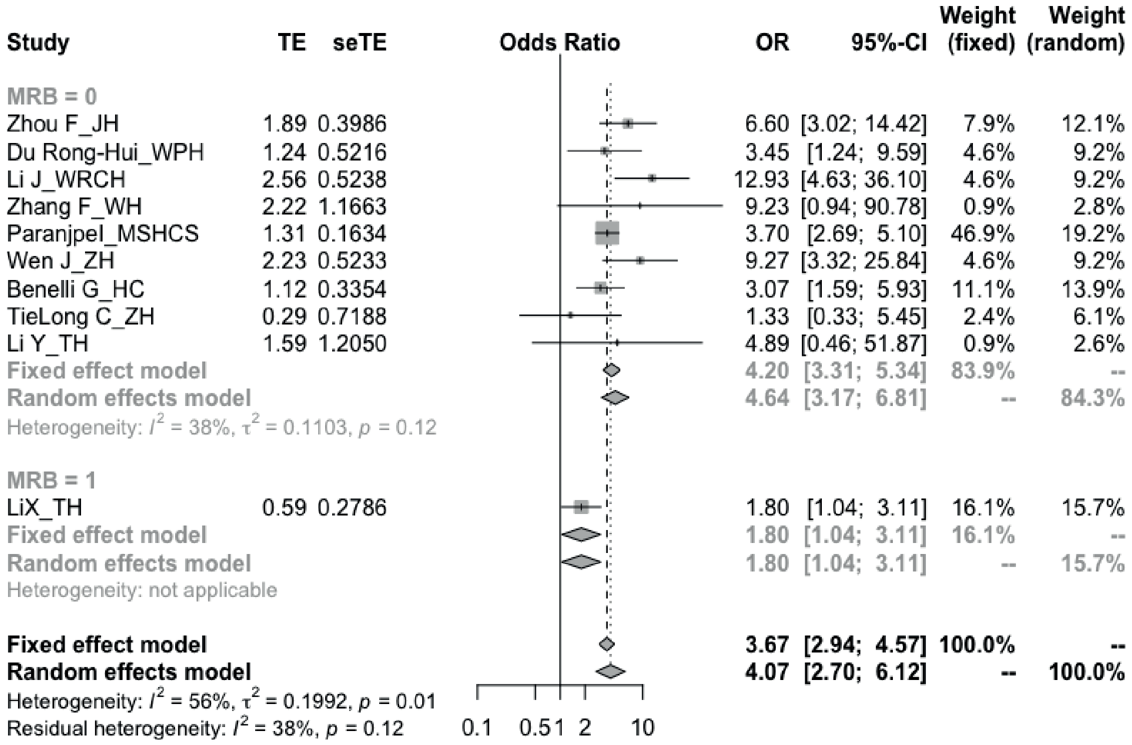

Candidate variable: High Neutrophil count (greater than 6.3 x 109/L), outcome: mortality

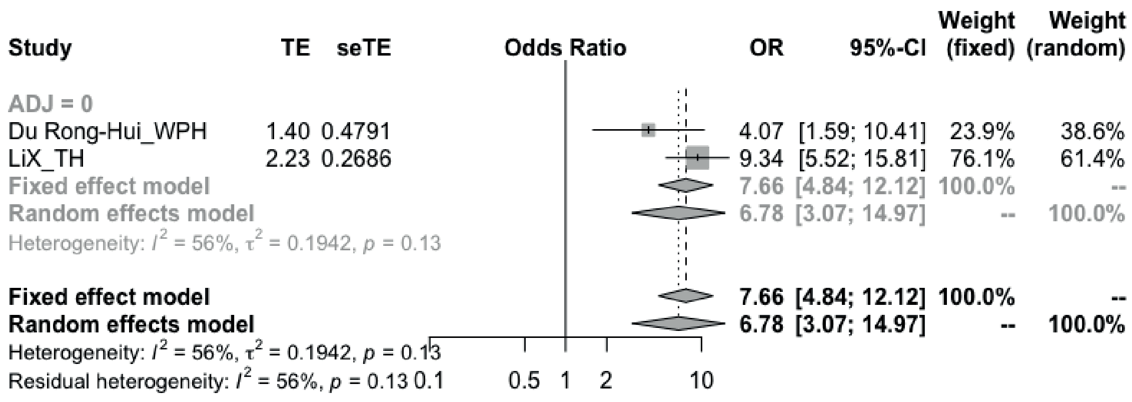

Candidate variable: Neutrophil count increase(per 1 x 109 U/L), outcome: mortality, subgroup analysis by risk of bias: (high vs moderate/low)

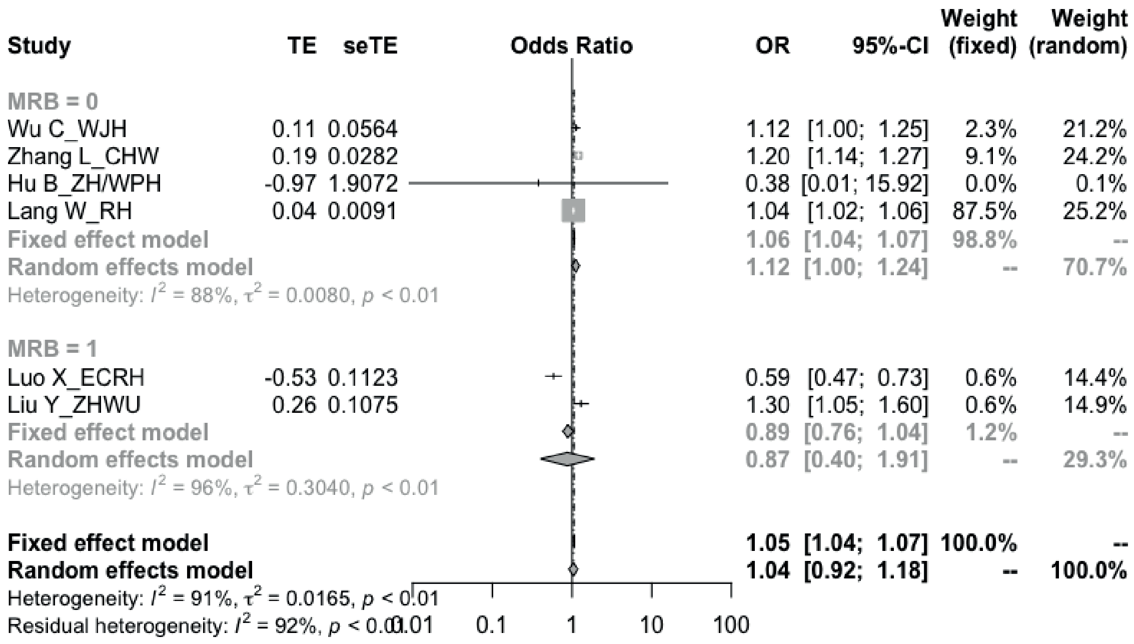

Candidate variable: Low neutrophil count (less than 1.8 x 109/L), outcome: mortality

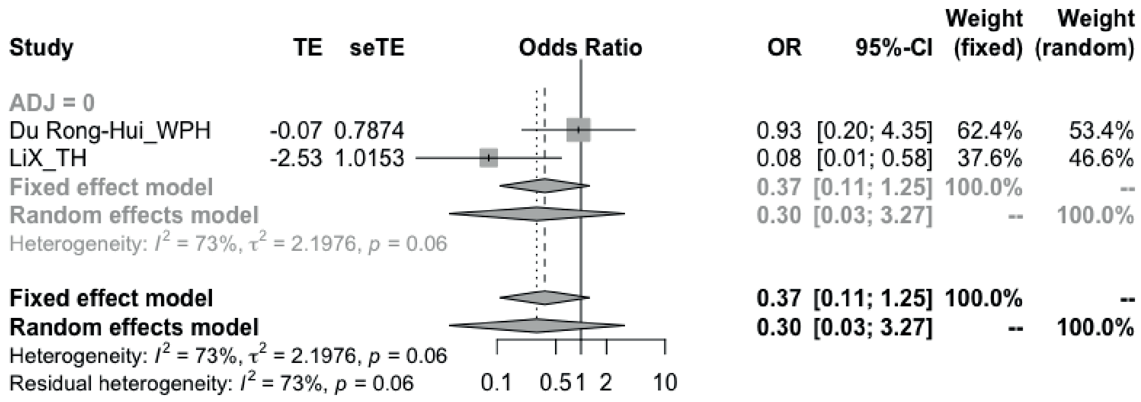

Candidate variable: Leukopenia (less than 3.5-4 x 10^9/L), outcome: mortality, subgroup analysis: (crude vs adjusted)

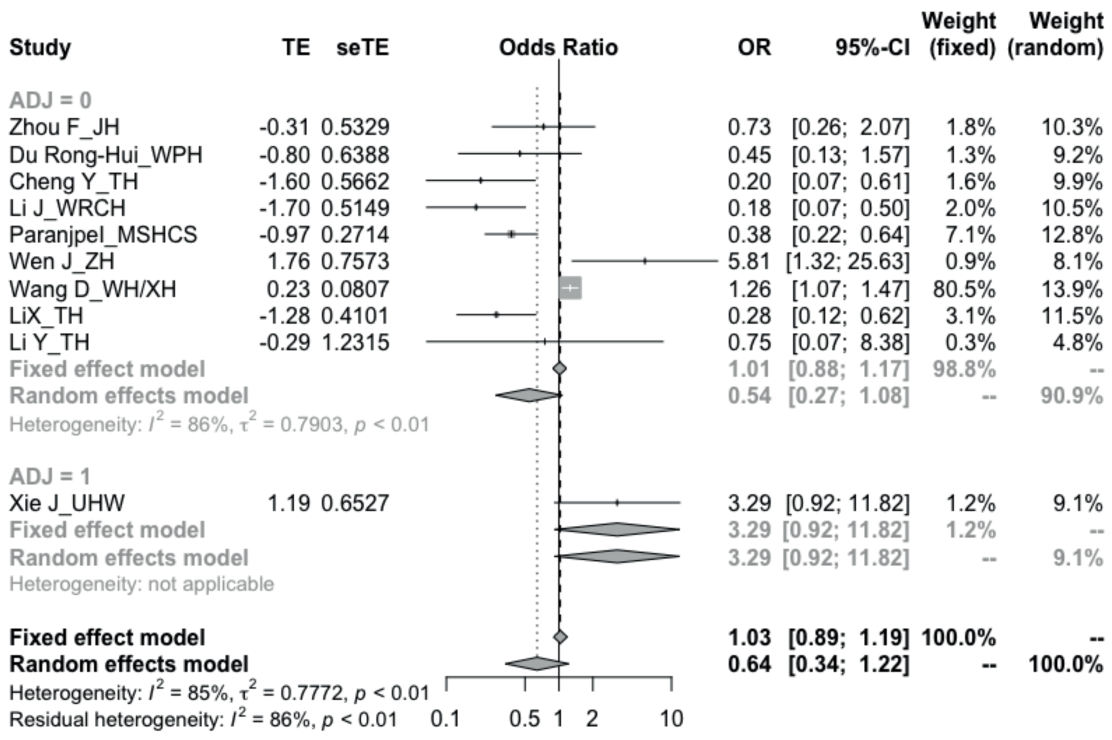

Candidate variable: Low Lymphocyte count (less than 0.8-1.5x 10^9/L), outcome: mortality, subgroup analysis: (crude vs adjusted)

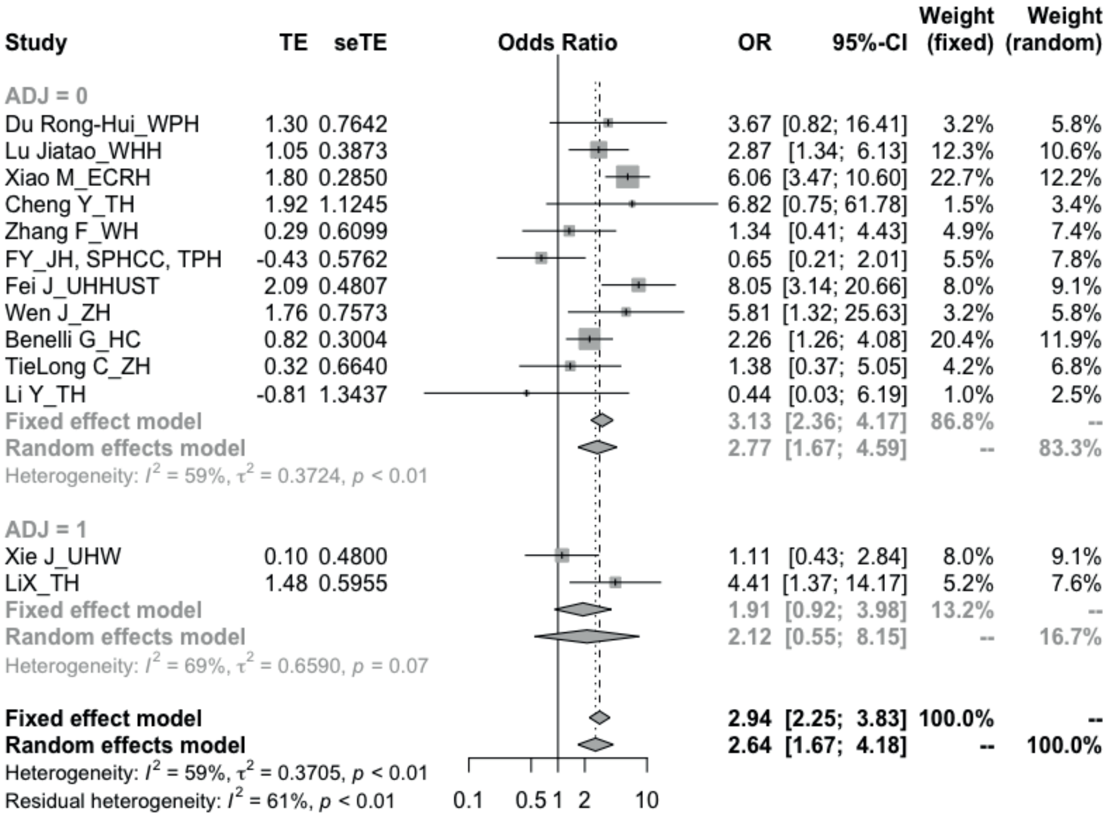

Candidate variable: Lymphocyte count increase (per 1 x 10^9 U/L), outcome: mortality, subgroup analysis: (crude vs adjusted)

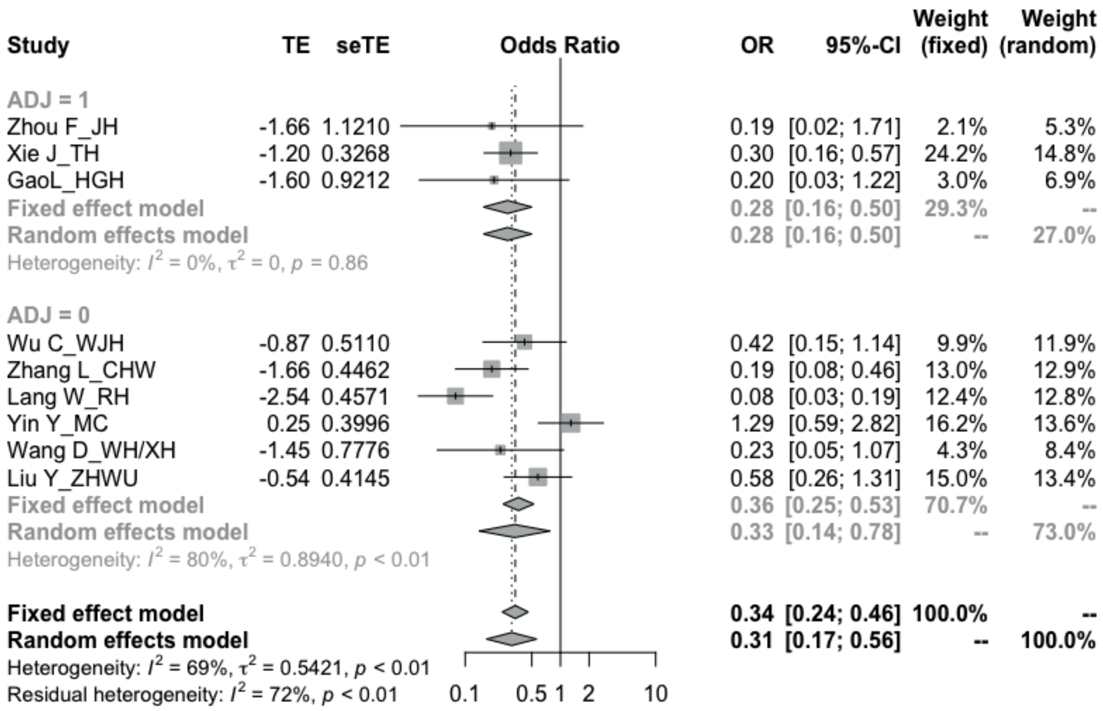

Candidate variable: Low platelet count (less than 100-150 x 10^9/L), outcome: mortality, subgroup analysis by risk of bias: (high vs moderate/low)

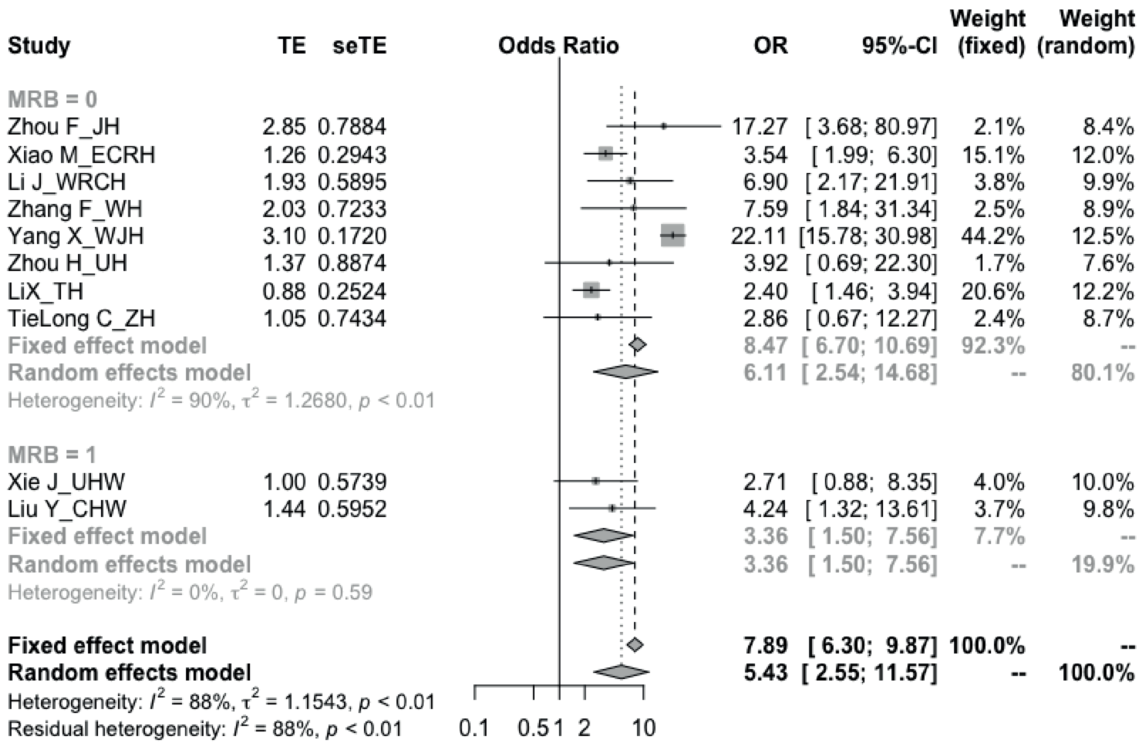

Candidate variable: High plasma creatinine (more than 1.5 mg%), outcome: mortality, subgroup analysis by risk of bias: (high vs moderate/low)

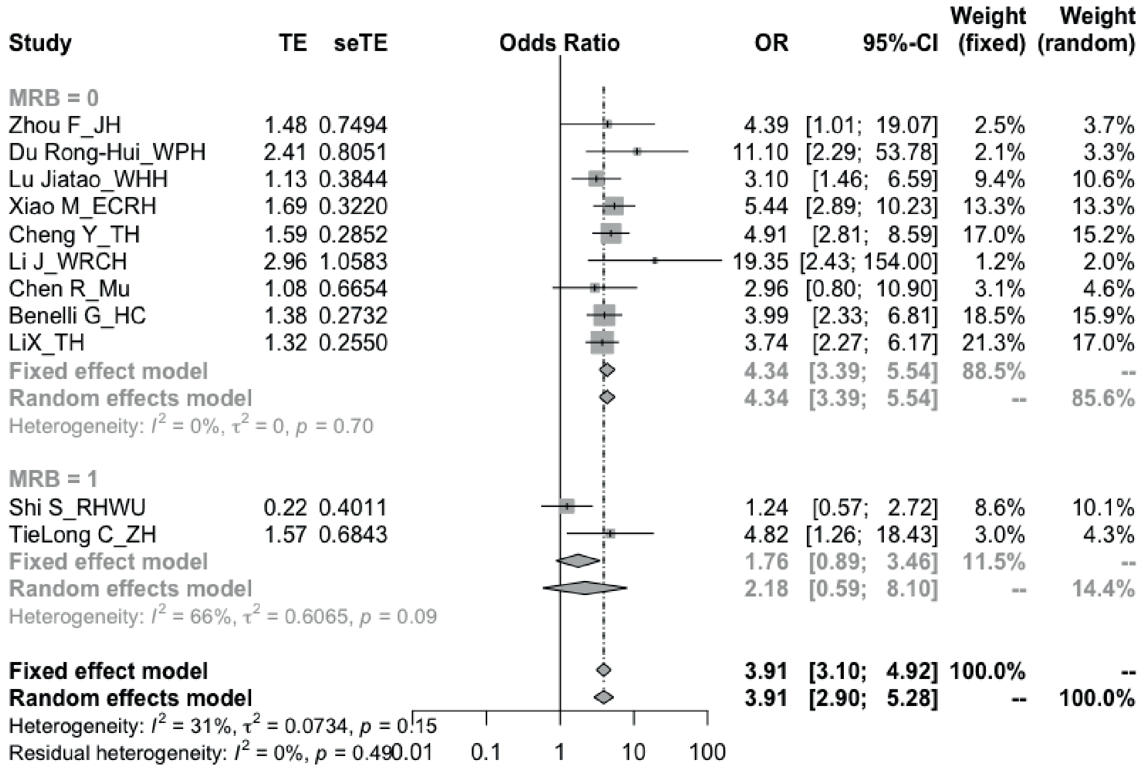

Candidate variable: Creatinine increase (per 0.1 mg%), outcome: mortality, subgroup analysis by risk of bias: (high vs moderate/low)

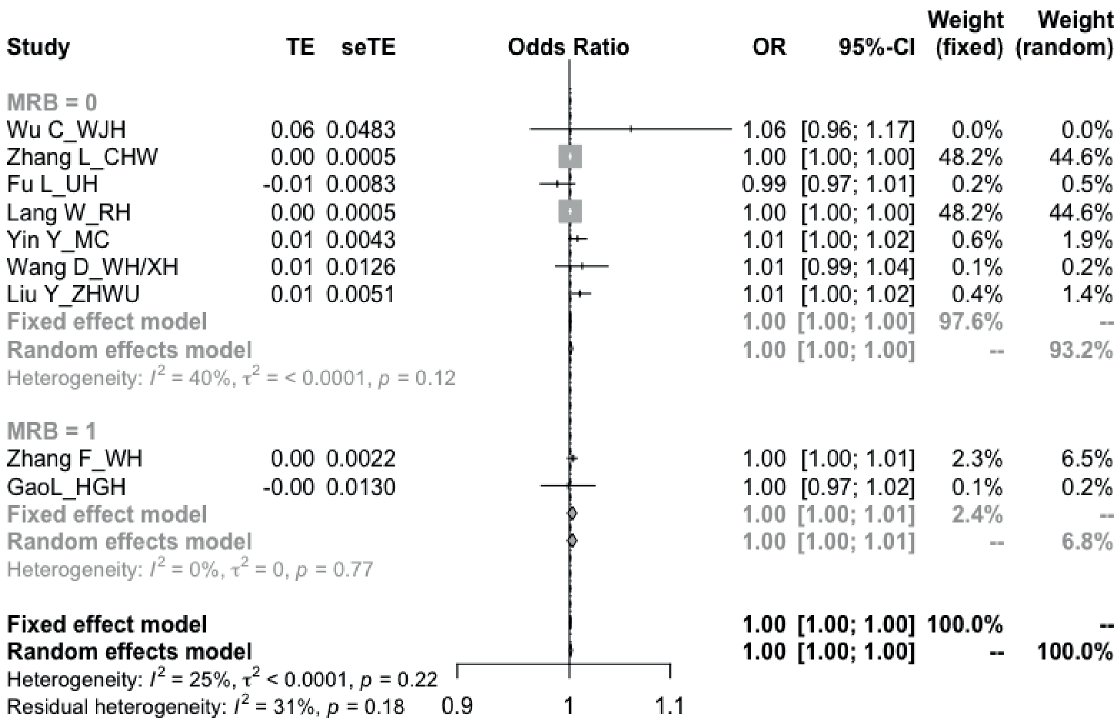

Candidate variable: Acute kidney injury, outcome: mortality, subgroup analysis: (crude vs adjusted)

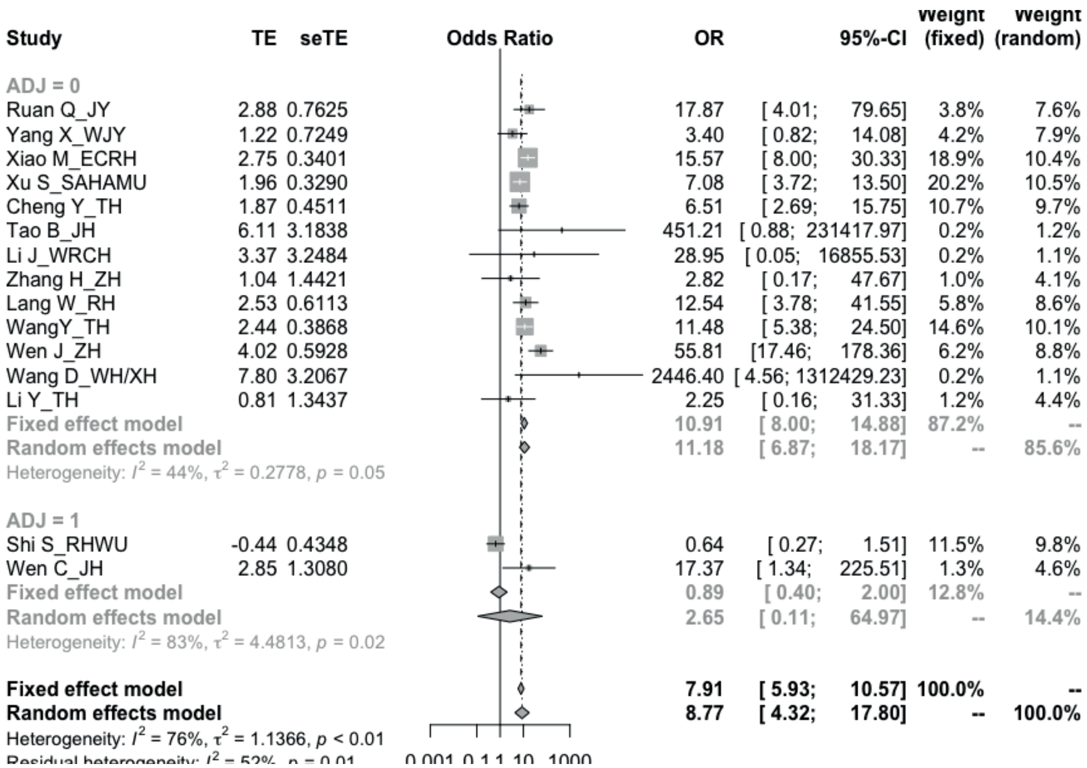

Candidate variable: High BUN (more than 5.2-9.5 mmol/L), outcome: mortality

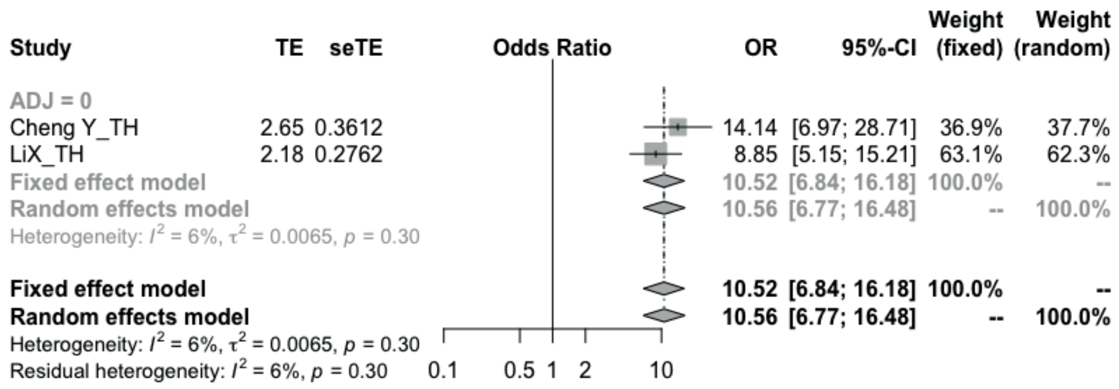

Candidate variable: High LDH (more than 240-250 U/L), outcome: mortality, subgroup analysis by risk of bias: (high vs moderate/low)

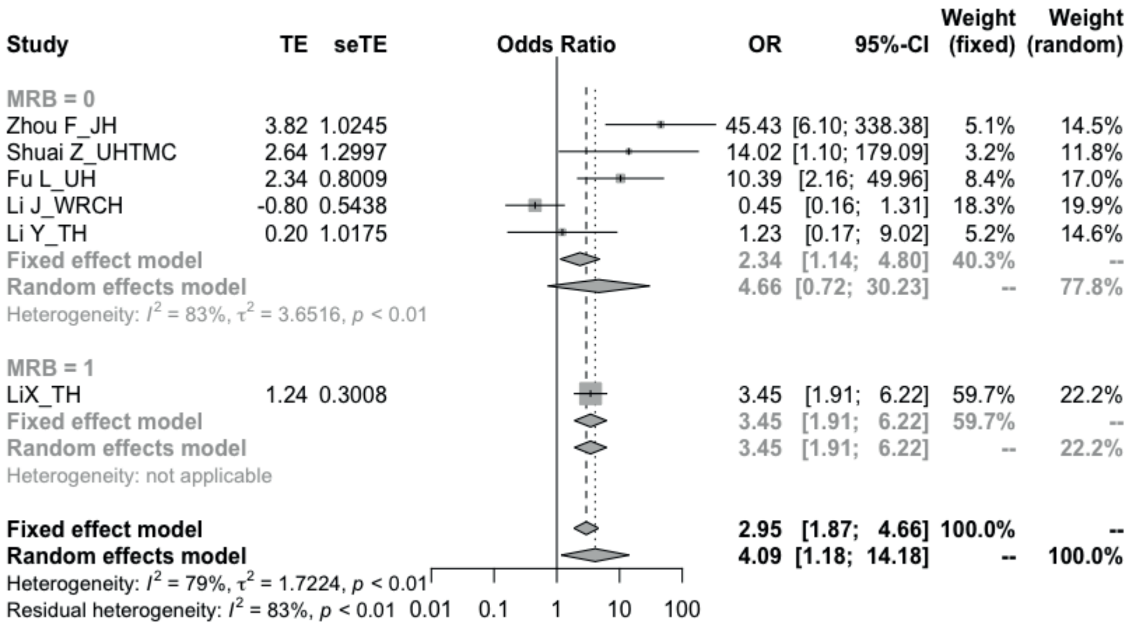

Candidate variable: LDH increase (per 1 U/L), outcome: mortality, subgroup analysis by risk of bias: (high vs moderate/low)

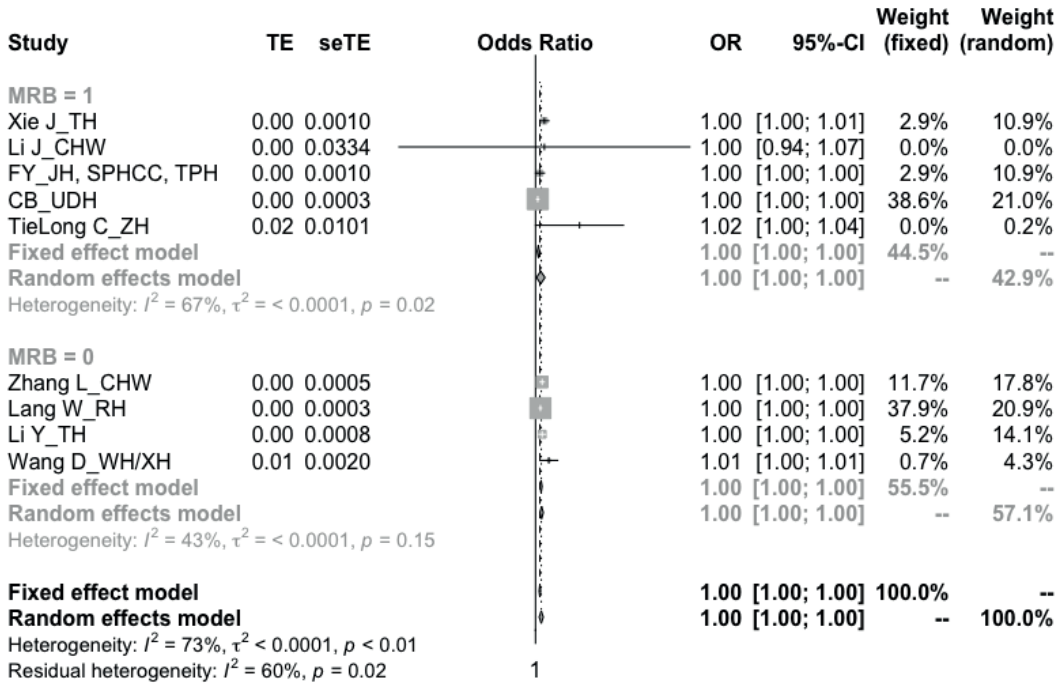

Candidate variable: High CK (more than 185-200 U/L), outcome: mortality

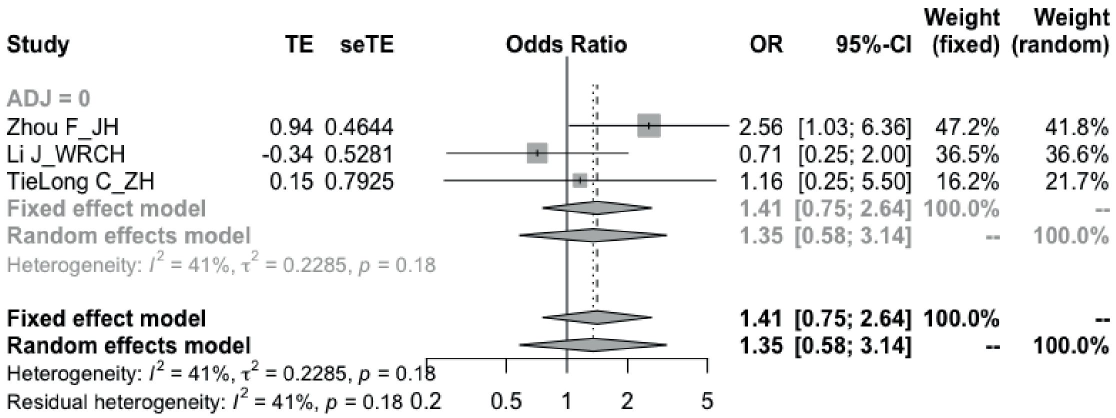

Candidate variable: Myocardial injury, outcome: mortality, subgroup analysis by risk of bias: (moderate/high vs low)

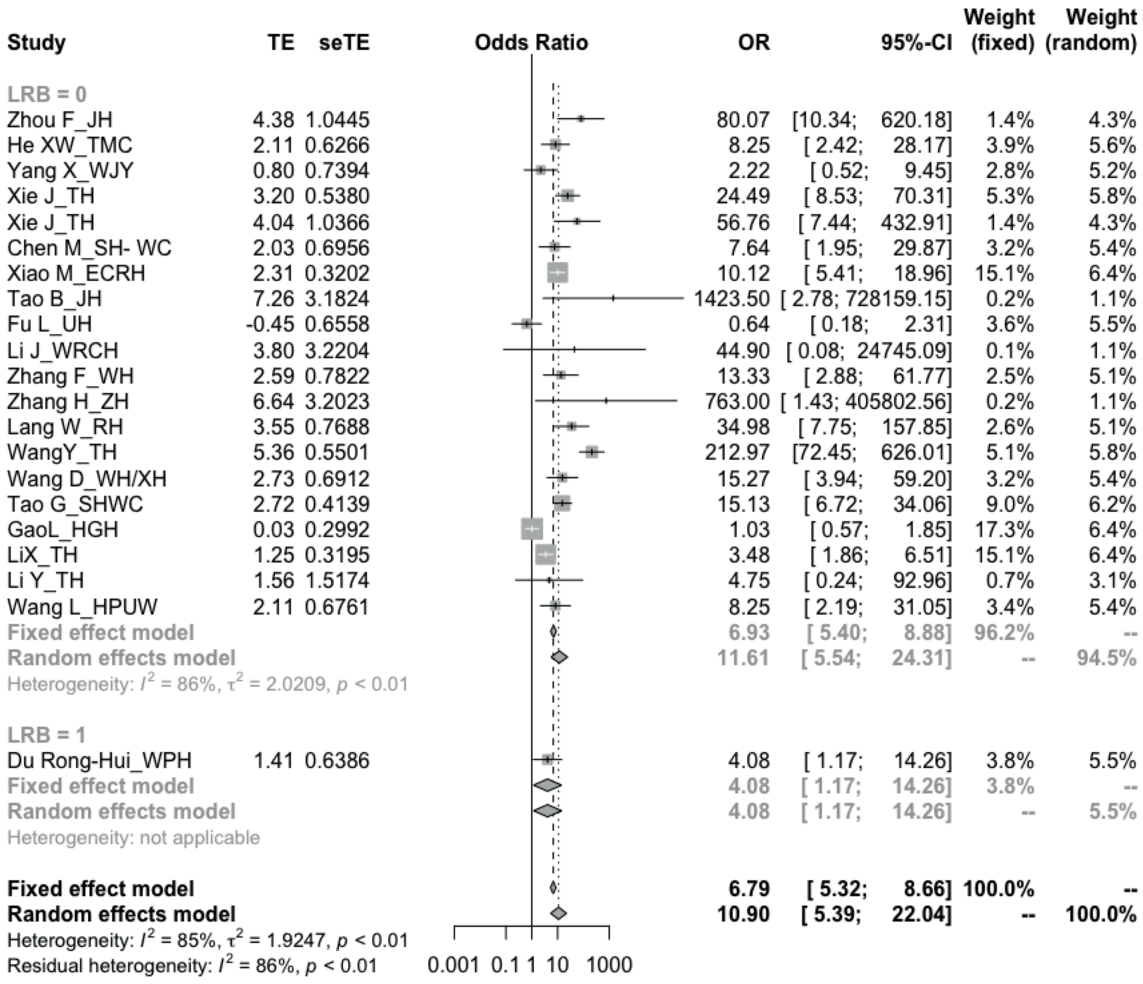

Candidate variable: CK-MB increase (per 1 U/L), outcome: mortality, subgroup analysis: (crude vs adjusted)

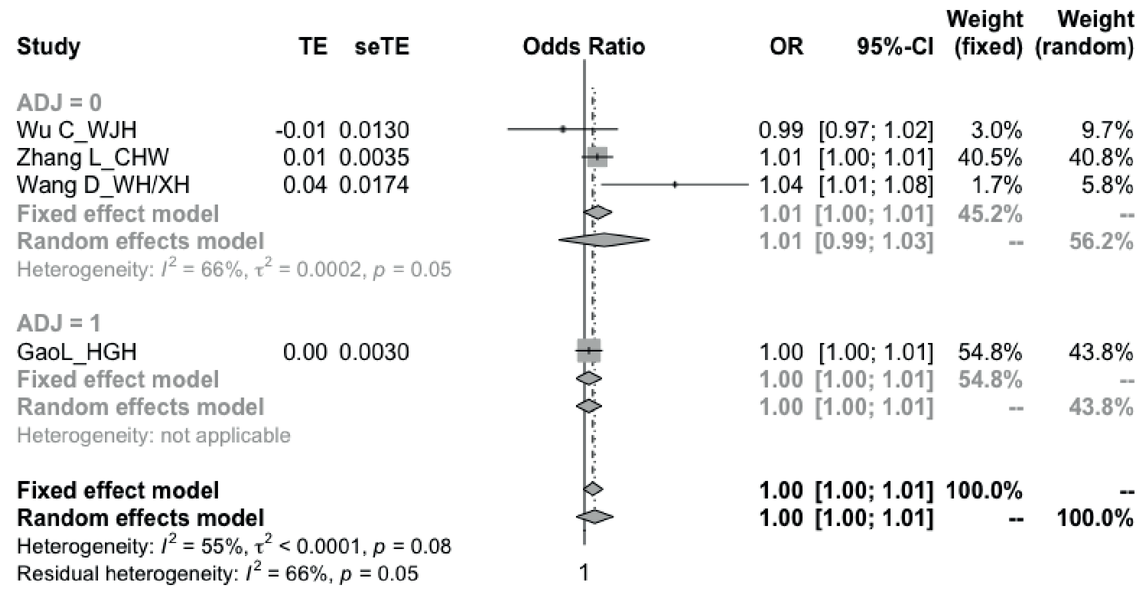

Candidate variable: Urea increase (per 1 mmol/L), outcome: mortality, subgroup analysis: (crude vs adjusted)

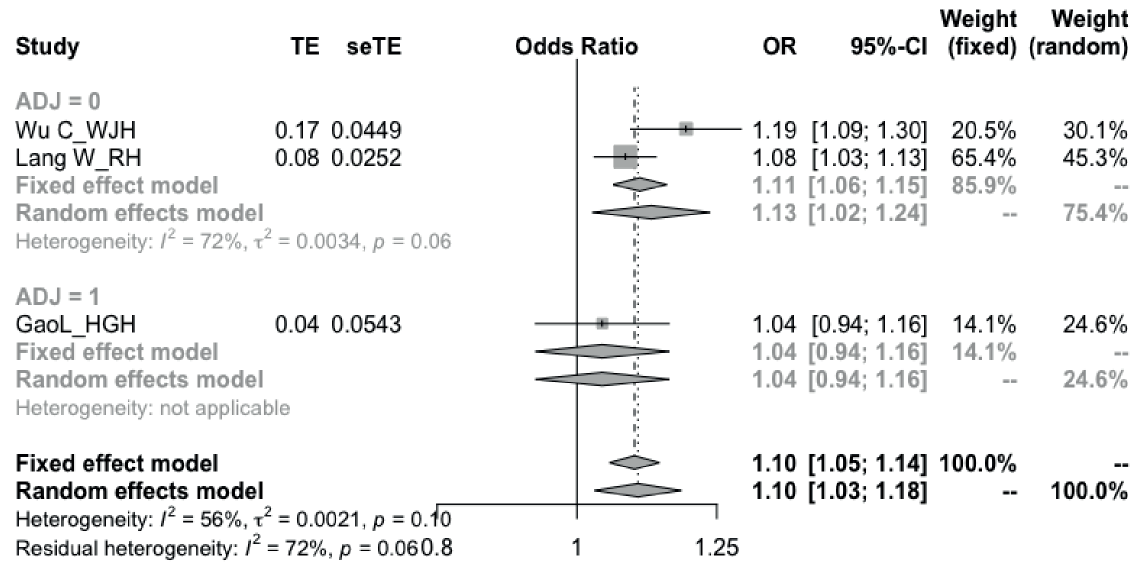

Candidate variable: High BNP (more than 500-900 pg/mL), outcome: mortality, subgroup analysis: (crude vs adjusted)

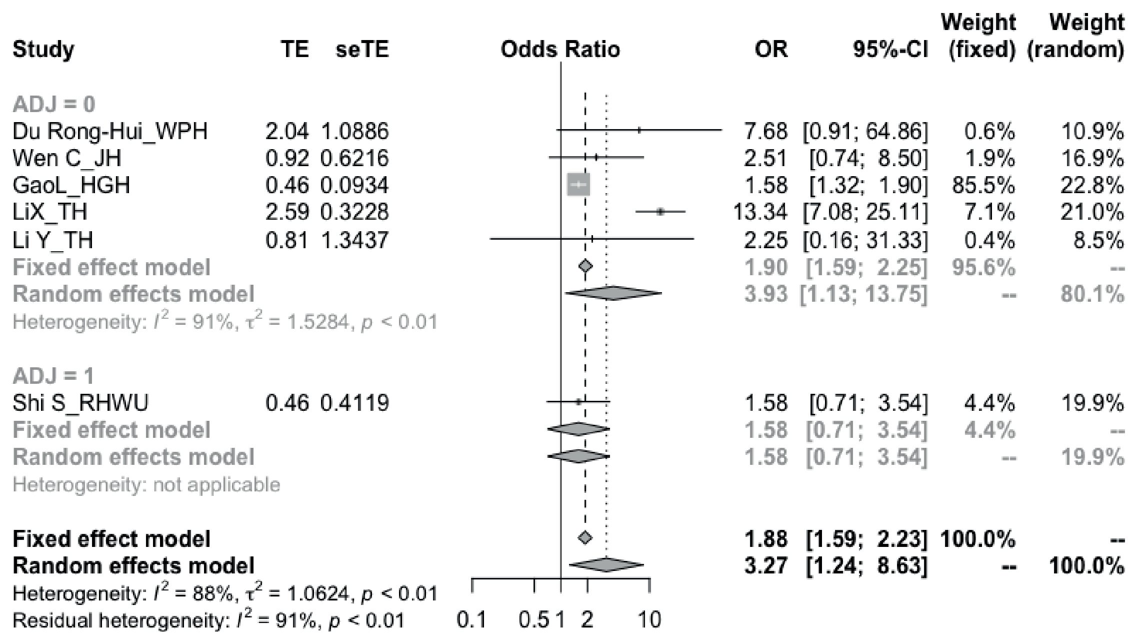

Candidate variable: High D-dimer (more than 500-1000 ng/ml), outcome: mortality, subgroup analysis by risk of bias: (high vs moderate/low)

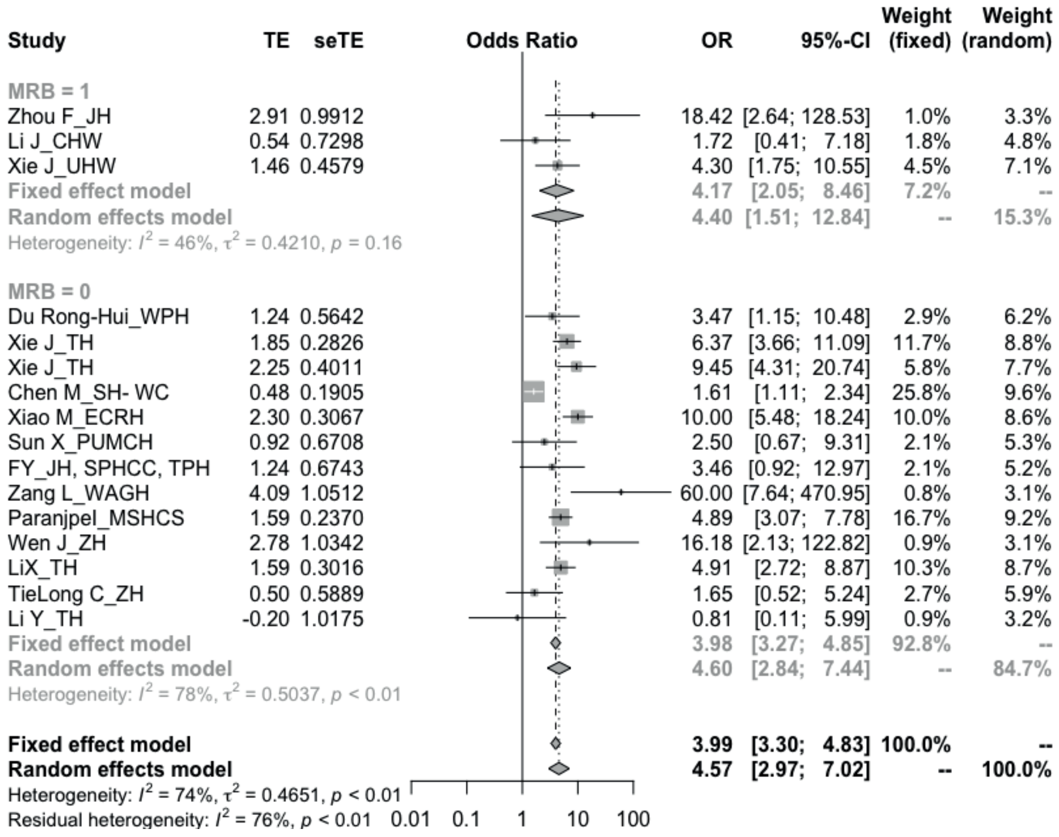

Candidate variable: D-dimer increase (per 10 ng/mL), outcome: mortality, subgroup analysis by risk of bias: (moderate/high vs low)

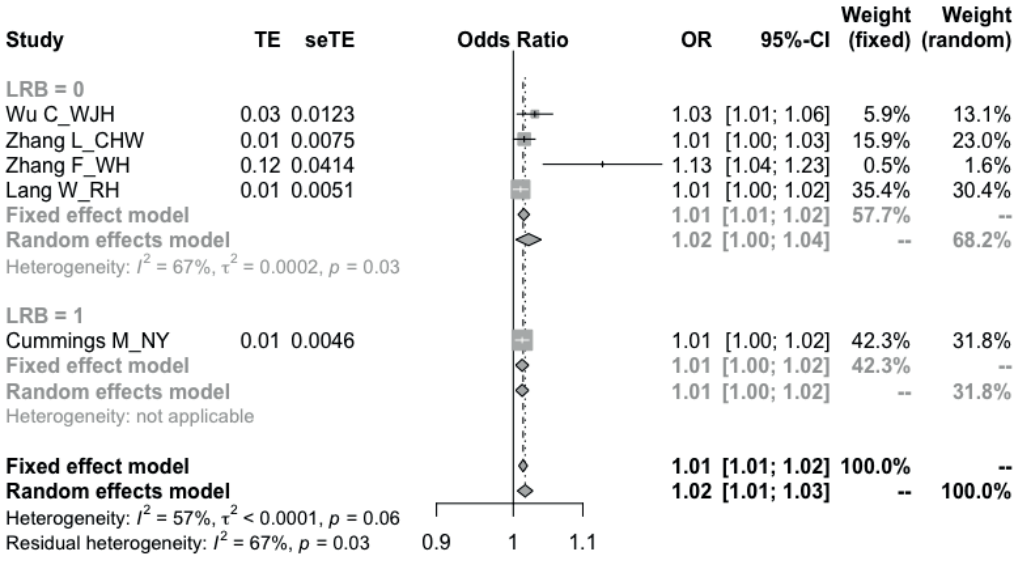

Candidate variable: Prolonged PT (more than 13.2-15 seconds), outcome: mortality, subgroup analysis: (crude vs adjusted)

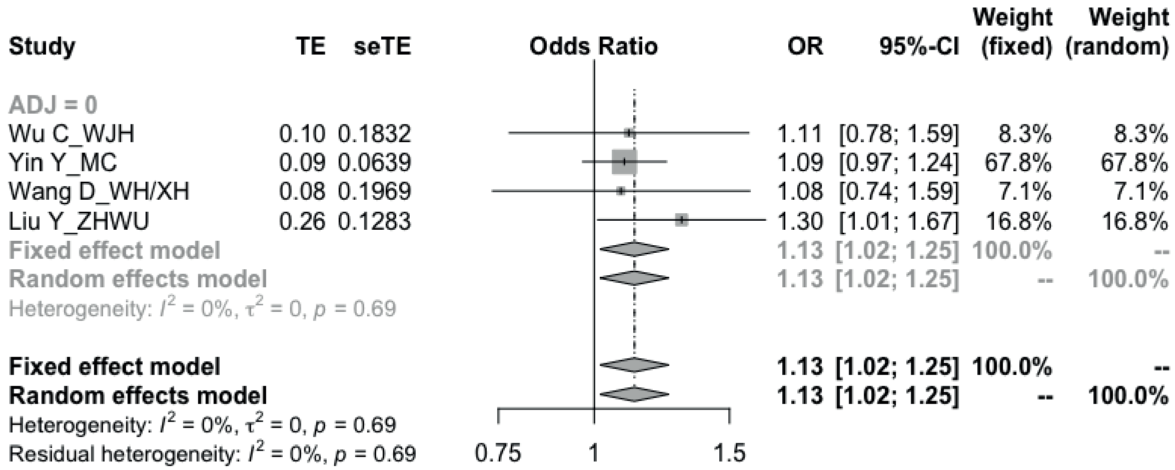

Candidate variable: PT increase (per 1 second), outcome: mortality

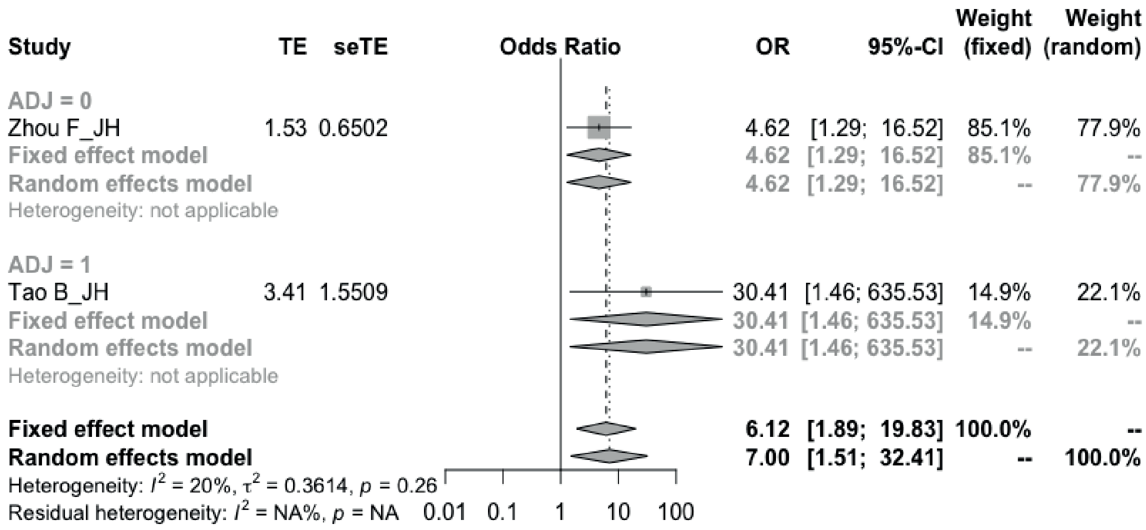

Candidate variable: APTT time increase (per 1 second), outcome: mortality

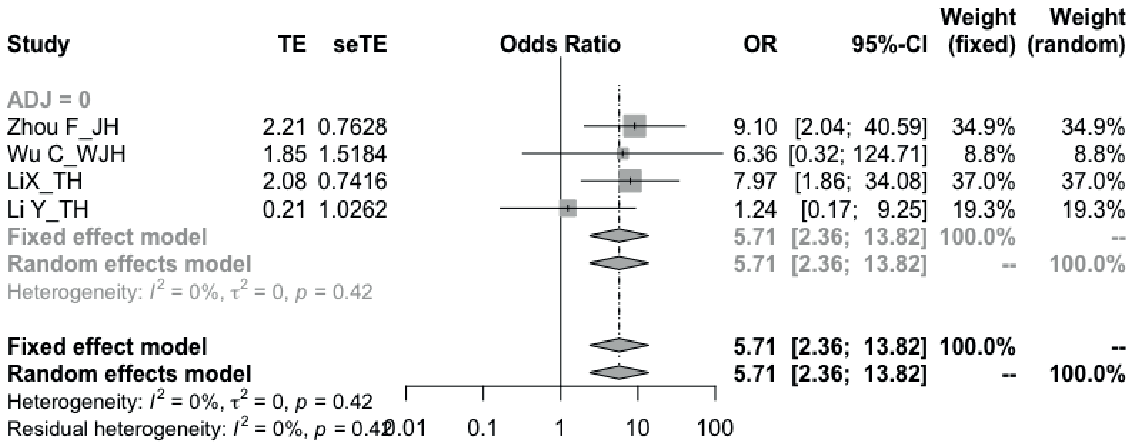

Candidate variable: High ferritin (more than 300-500 ng/mL), outcome: mortality

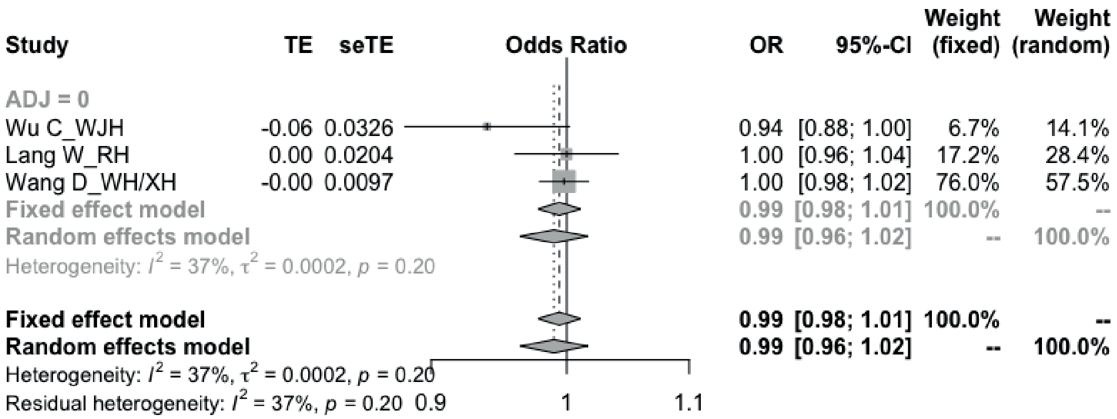

Candidate variable: High CRP (more than 1-100 mg/l), outcome: mortality, subgroup analysis: (crude vs adjusted)

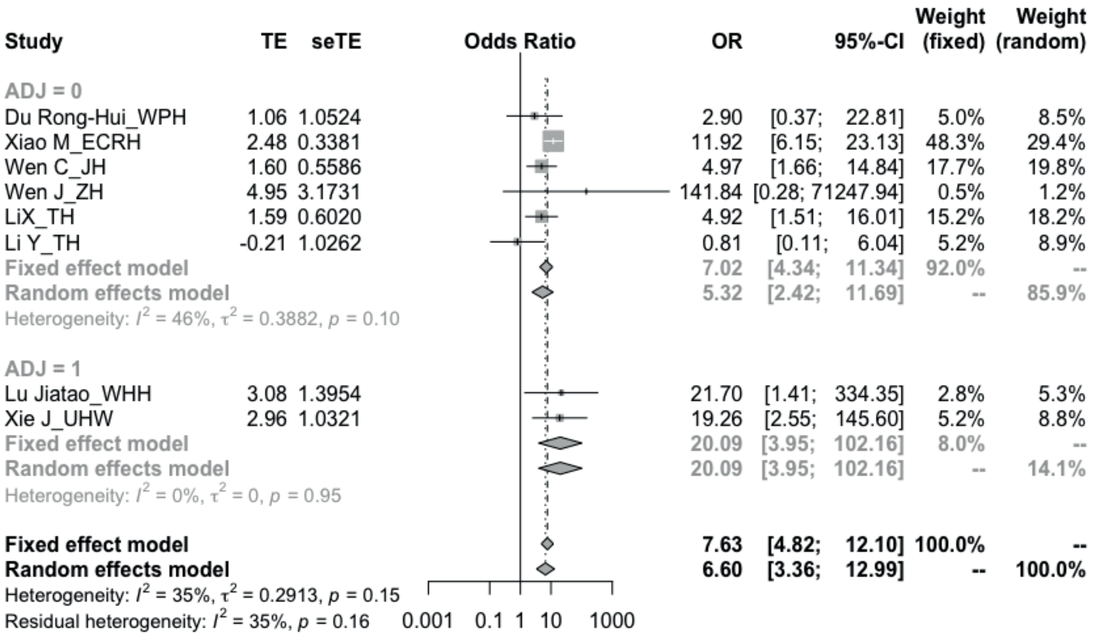

Candidate variable: CRP increase (per 1 mg/L), outcome: mortality, subgroup analysis by risk of bias: (high vs moderate/low)

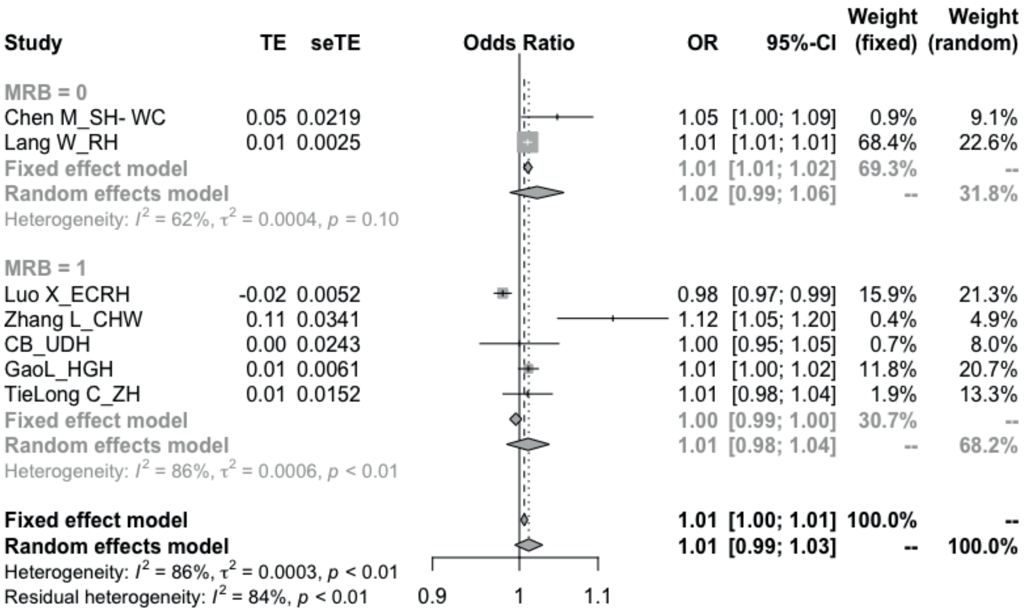

Candidate variable: High ESR (more than 10-20 mm/H), outcome: mortality

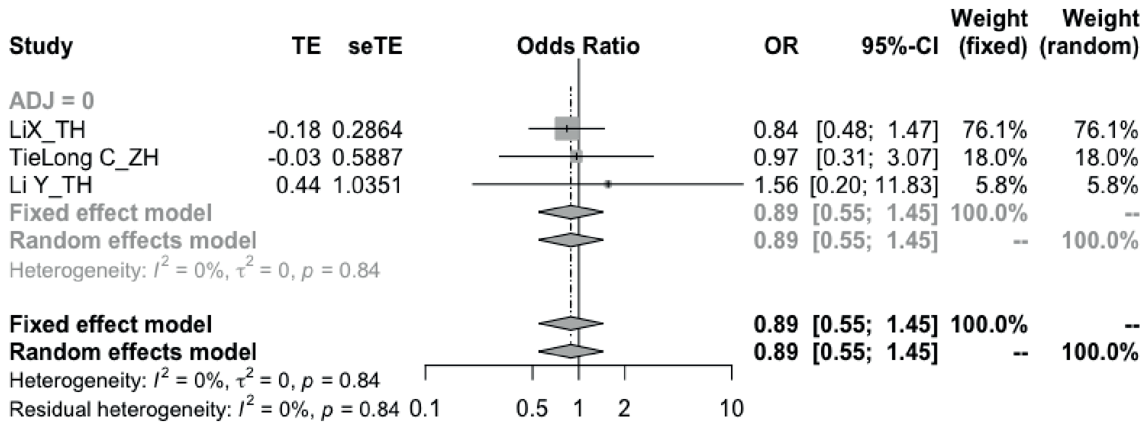

Candidate variable: High IL-6 (more than 5-20 pg/ml), outcome: mortality

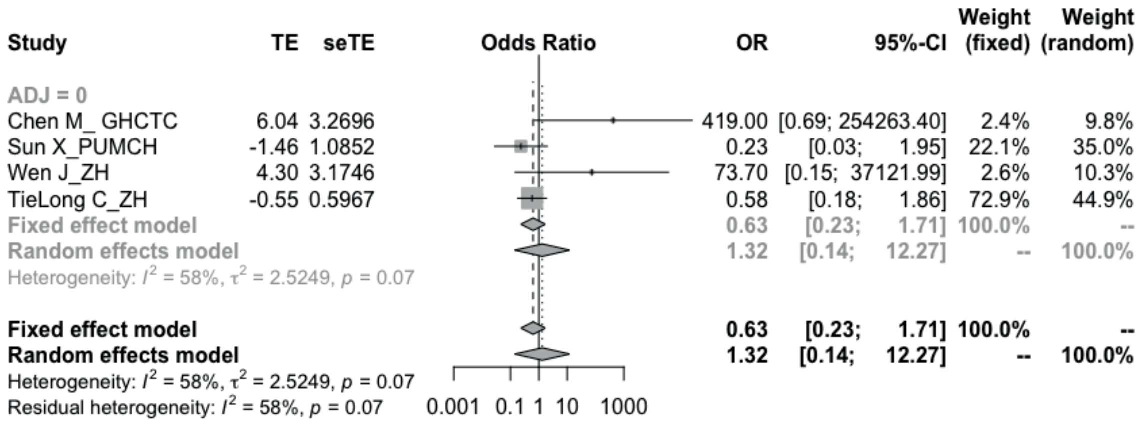

Candidate variable: IL-6 increase (per 1 pg/mL), outcome: mortality, subgroup analysis: (crude vs adjusted)

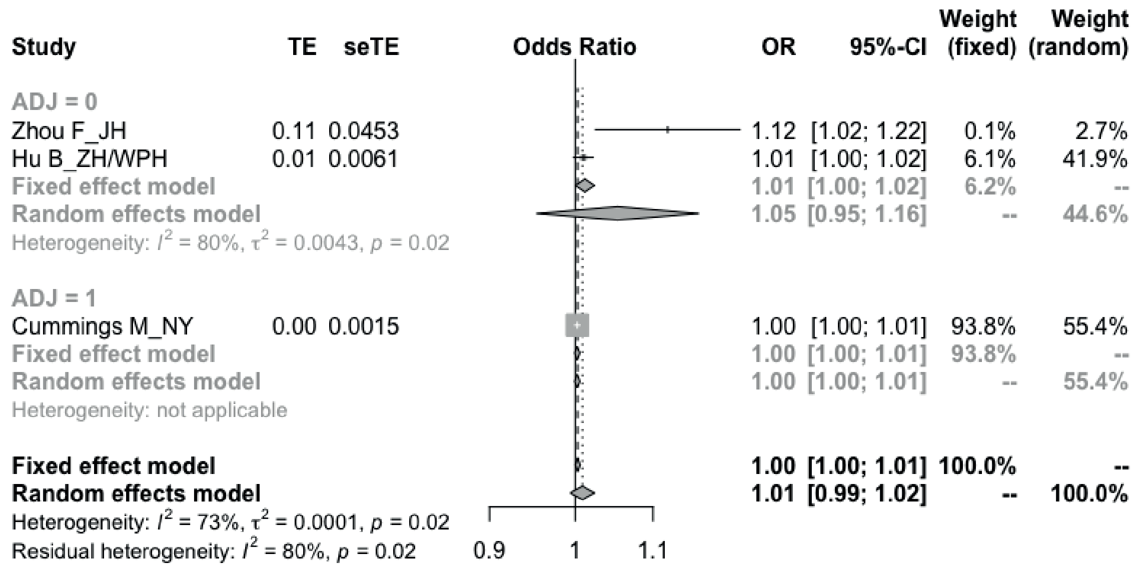

Candidate variable: High procalcitonin (more than 0.01-05 ng/ml), outcome: mortality, subgroup analysis by risk of bias: (high vs moderate/low)

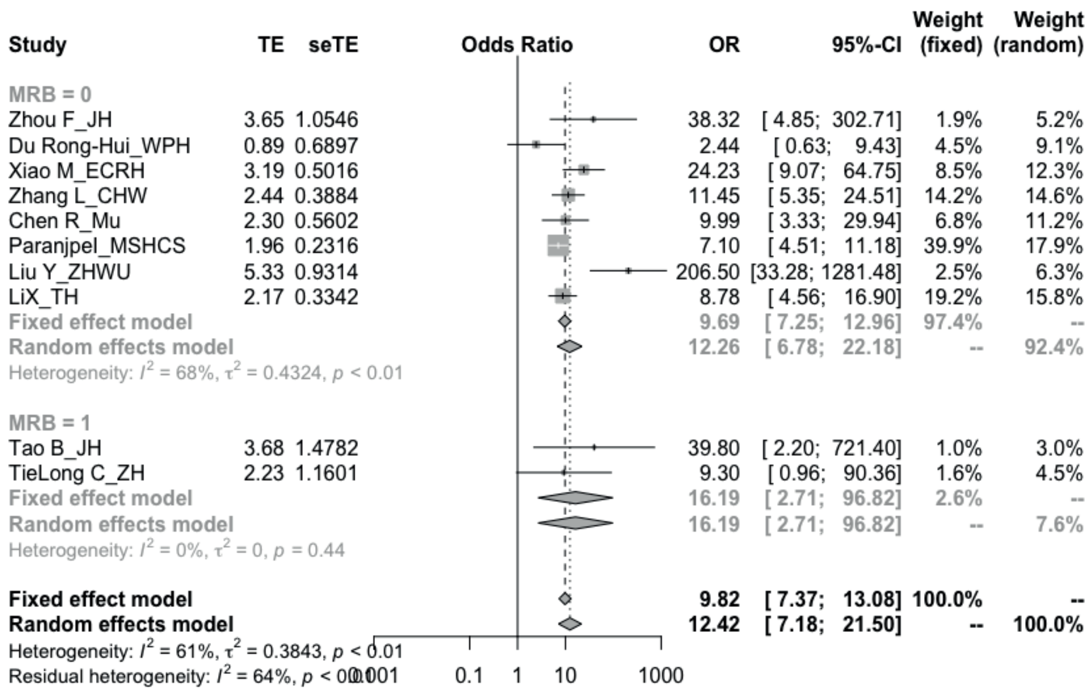

Candidate variable: Porcalcitonin increase (per 0.1 ng/ml), outcome: mortality, subgroup analysis by risk of bias: (high vs moderate/low)

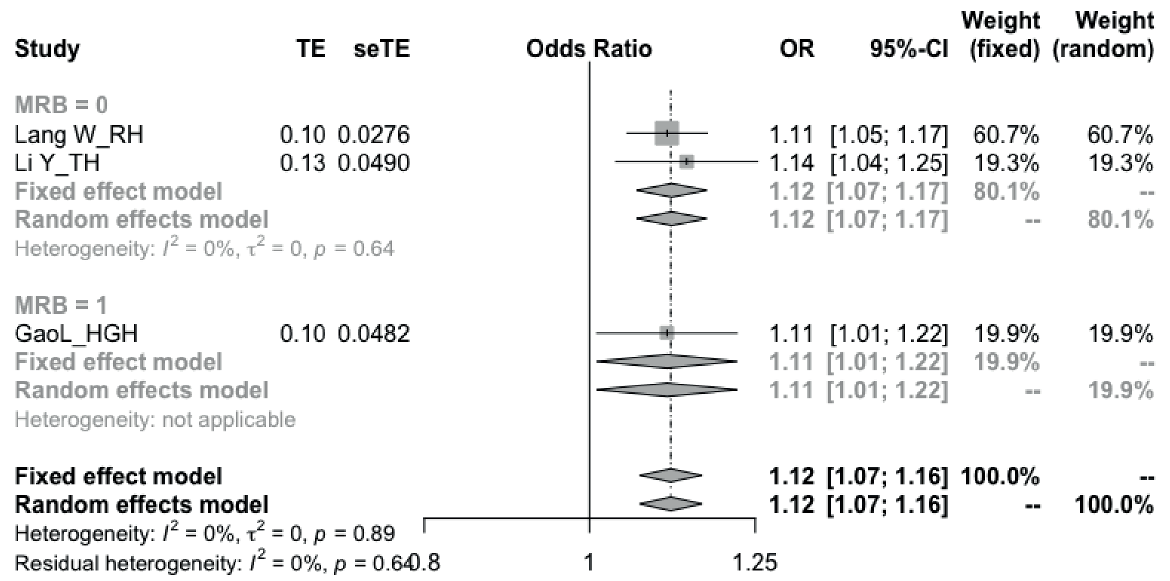

Candidate variable: High total bilirubin (more than 17-21pg/ml), outcome: mortality, subgroup analysis: (crude vs adjusted)

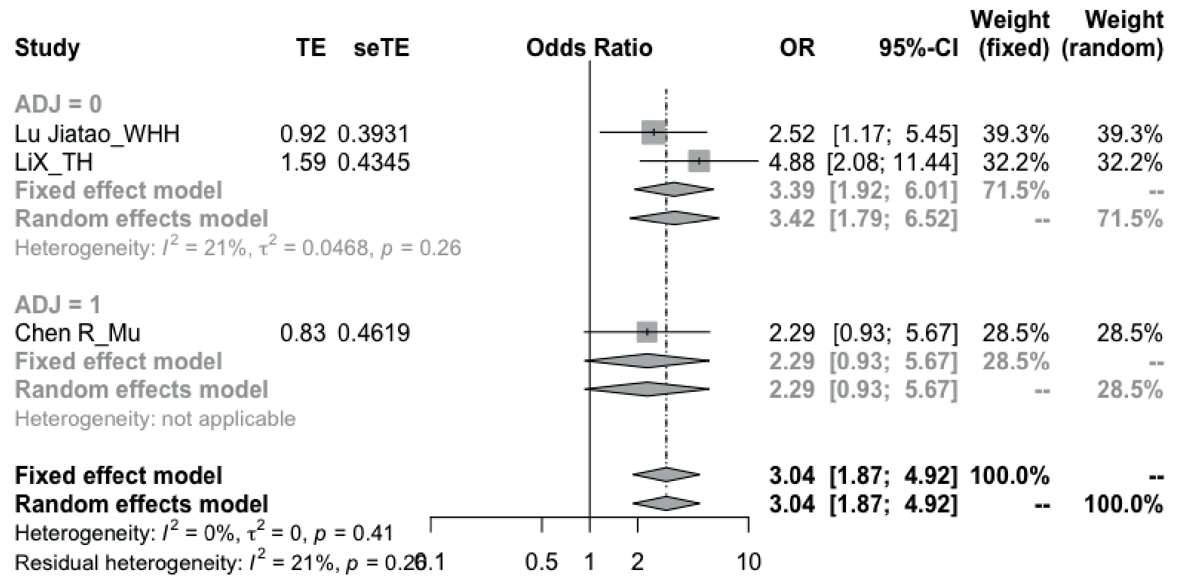

Candidate variable: Total bilirubin increase (per 1 μM), outcome: mortality, subgroup analysis: (crude vs adjusted)

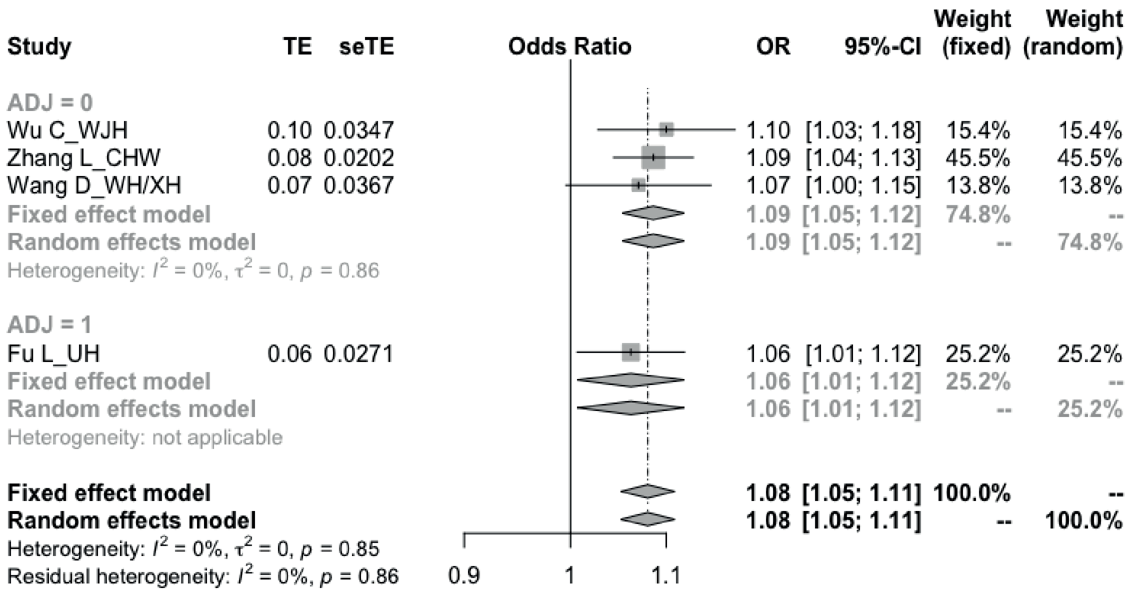

Candidate variable: High AST level (more than 32-40 U/l). outcome: mortality. subgroup analysis by risk of bias: (high vs moderate/low)

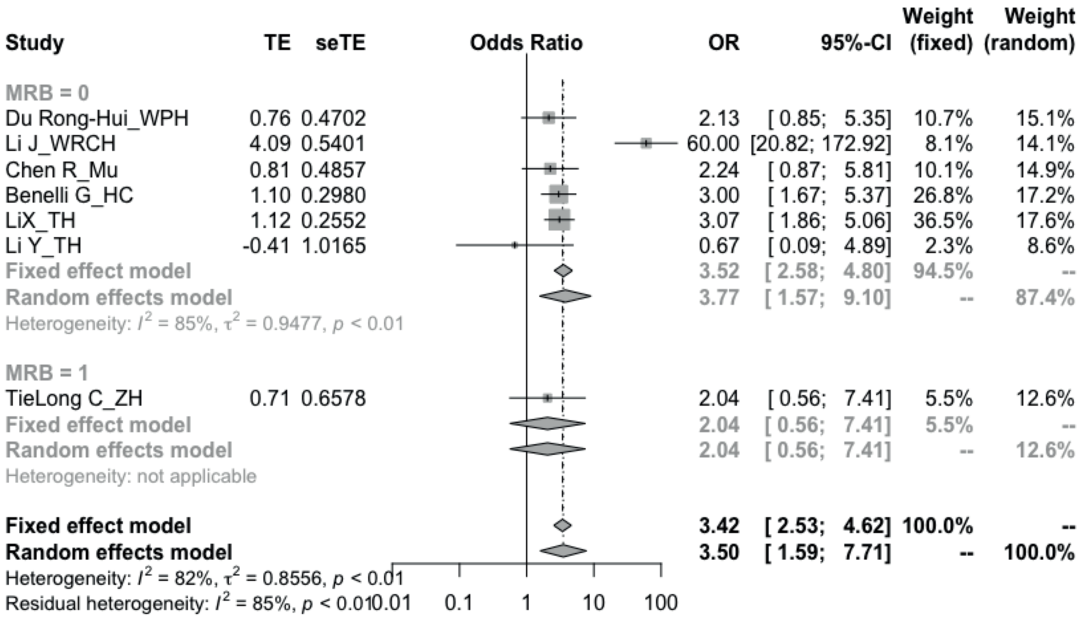

Candidate variable: AST increase (per 1 U/L). outcome: mortality. subgroup analysis: (crude vs adjusted)

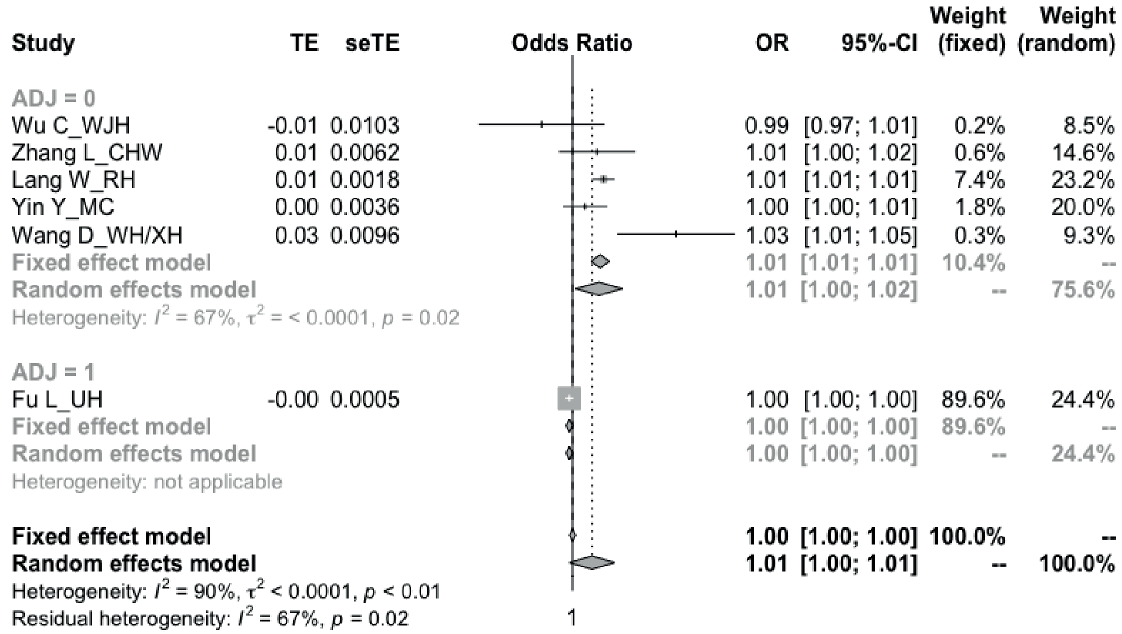

Candidate variable: High ALT level (more than 35-50 U/L), outcome: mortality.

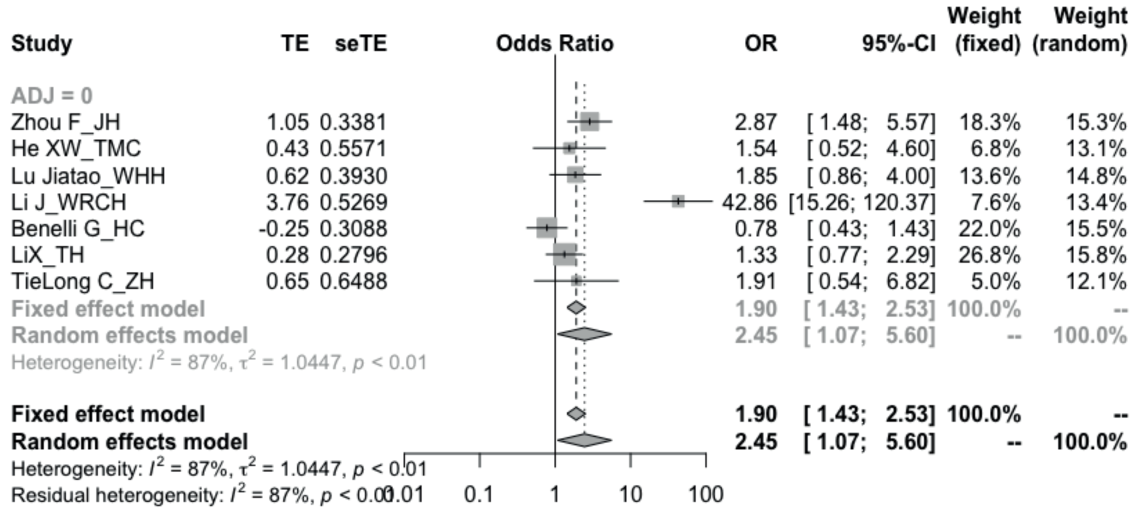

Candidate variable: ALT increase (per 1 U/L). outcome: mortality. subgroup analysis: (crude vs adjusted)

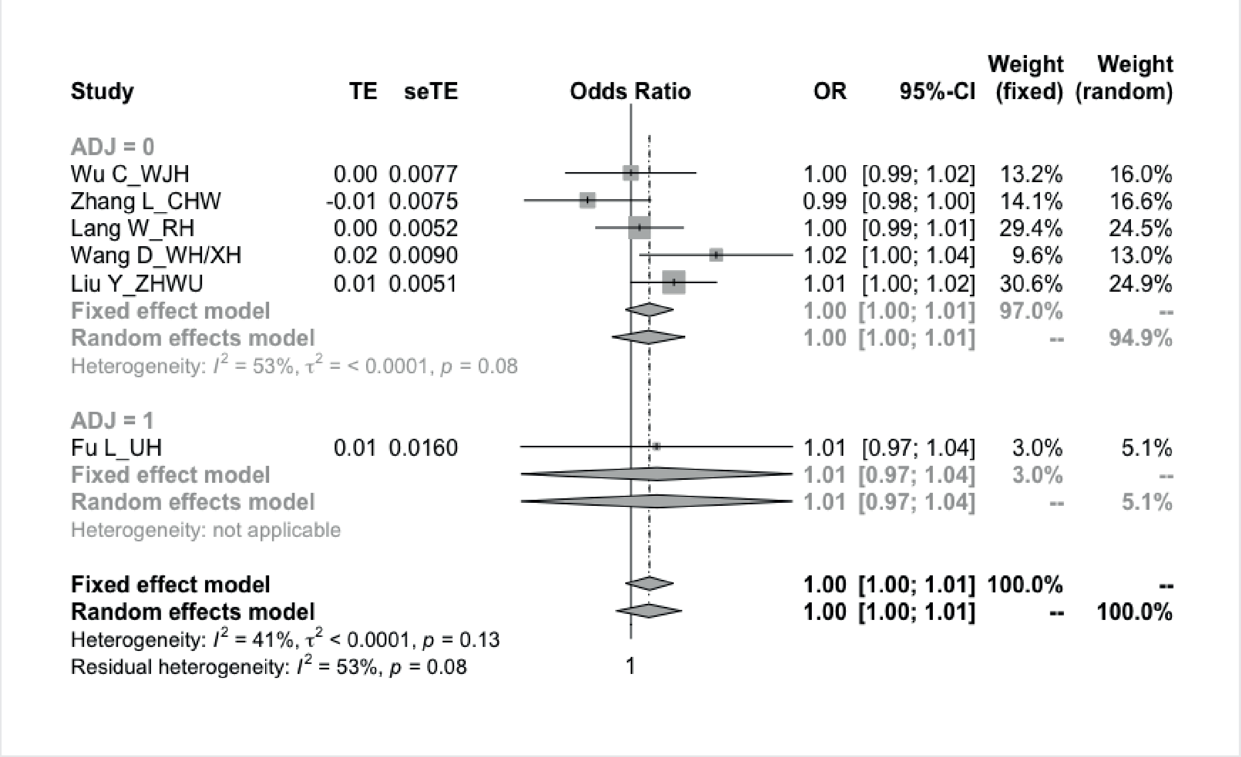

Candidate variable: Low albumin. outcome: mortality

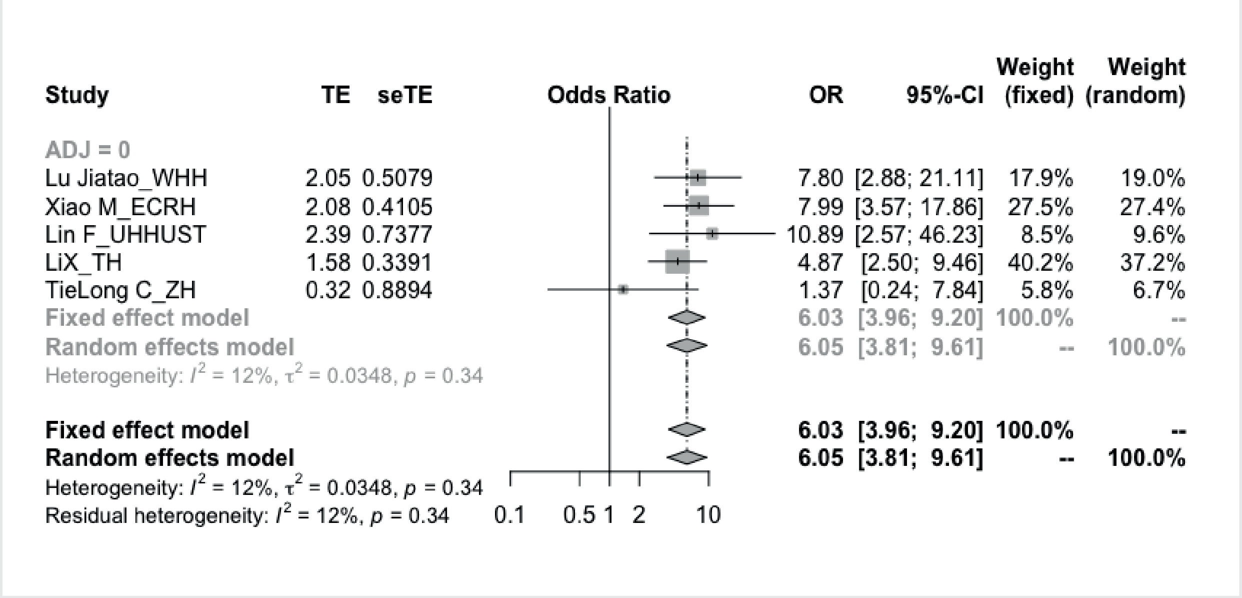

Candidate variable: Albumin increase (per 10 g/L). outcome: mortality

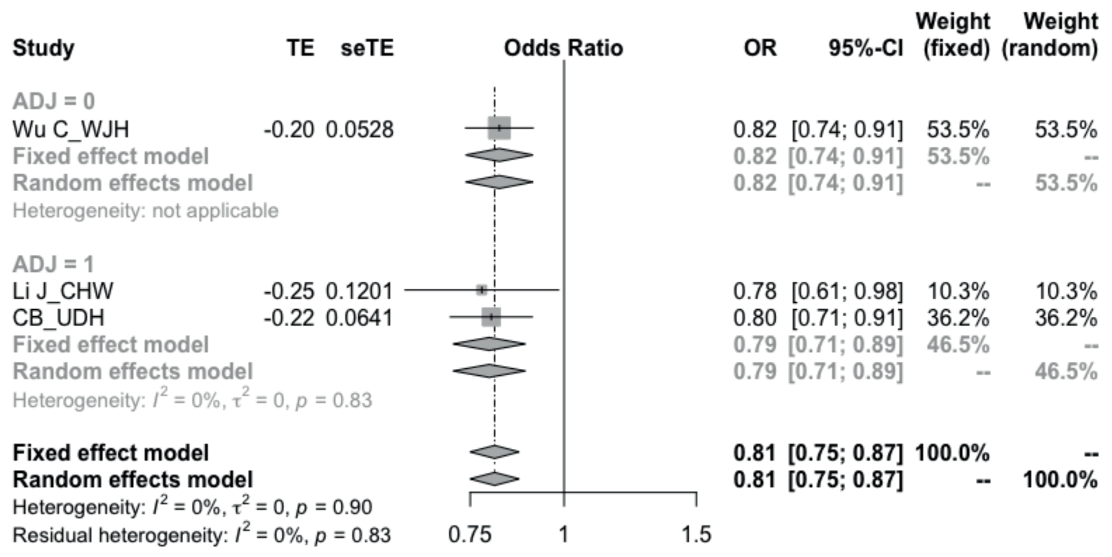

Candidate variable: High glucose (more than 6 mmol/l), outcome: mortality, subgroup analysis: (crude vs adjusted)

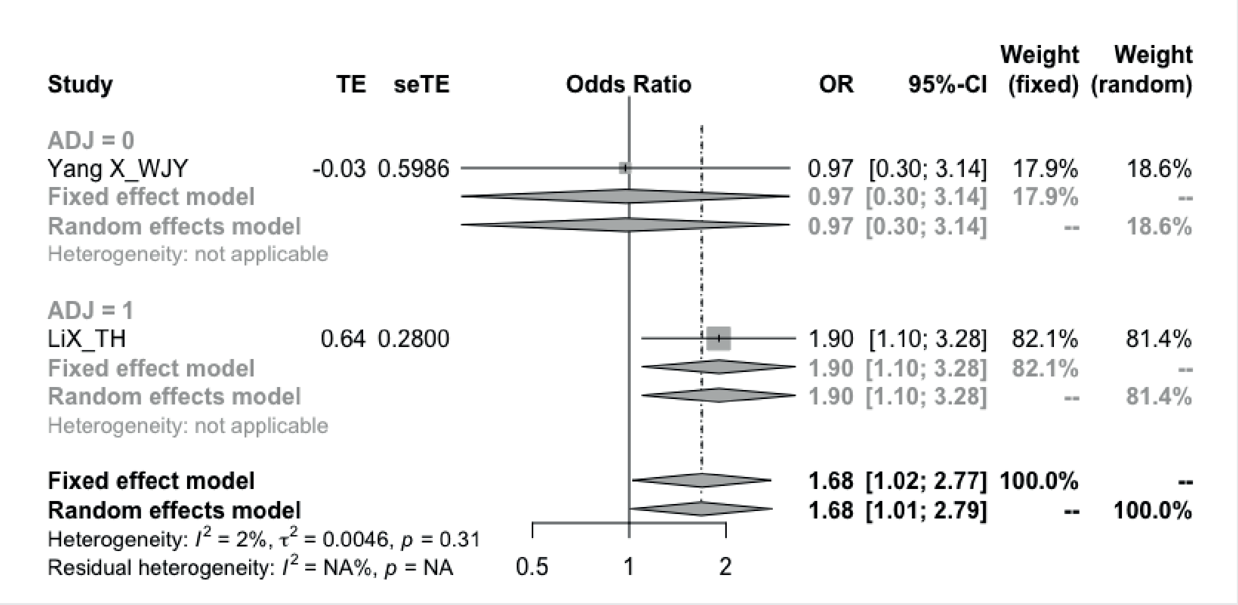

Candidate variable: Glucose increase (per 1 mmol/L), outcome: mortality, subgroup analysis: (crude vs adjusted)

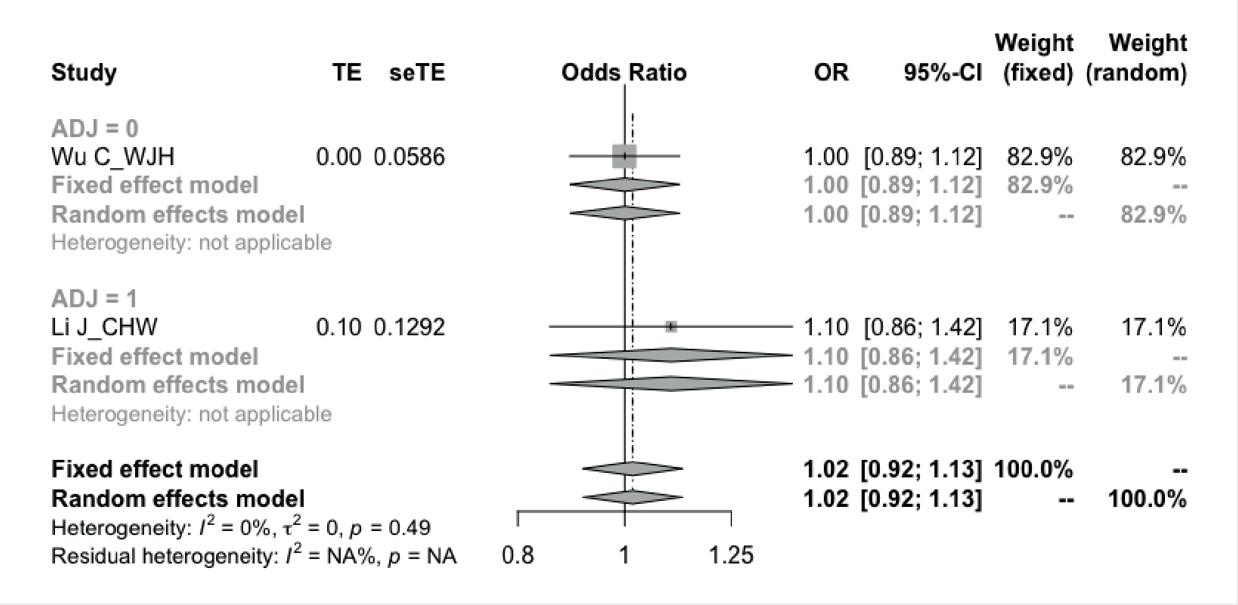

Candidate variable: Lactate increase (per 1 mmol/L), outcome: mortality

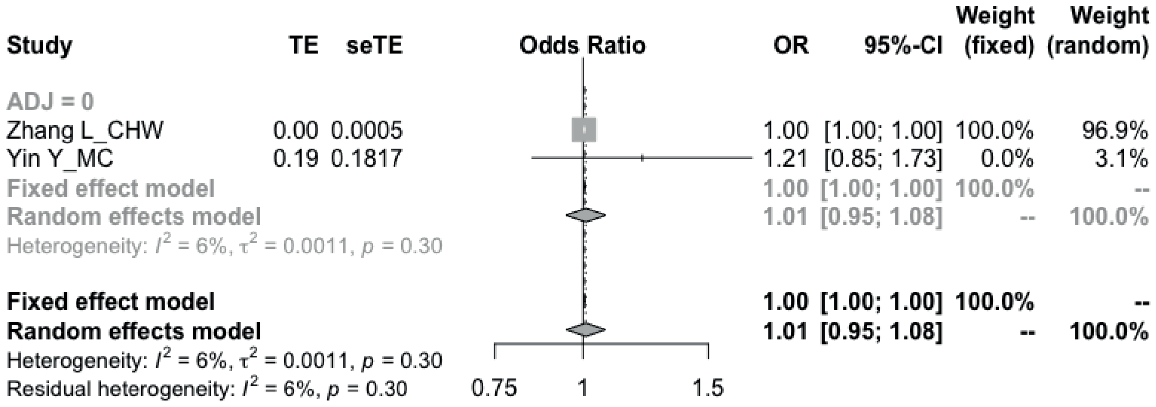

Candidate variable: Ground glass opacity. outcome: mortality

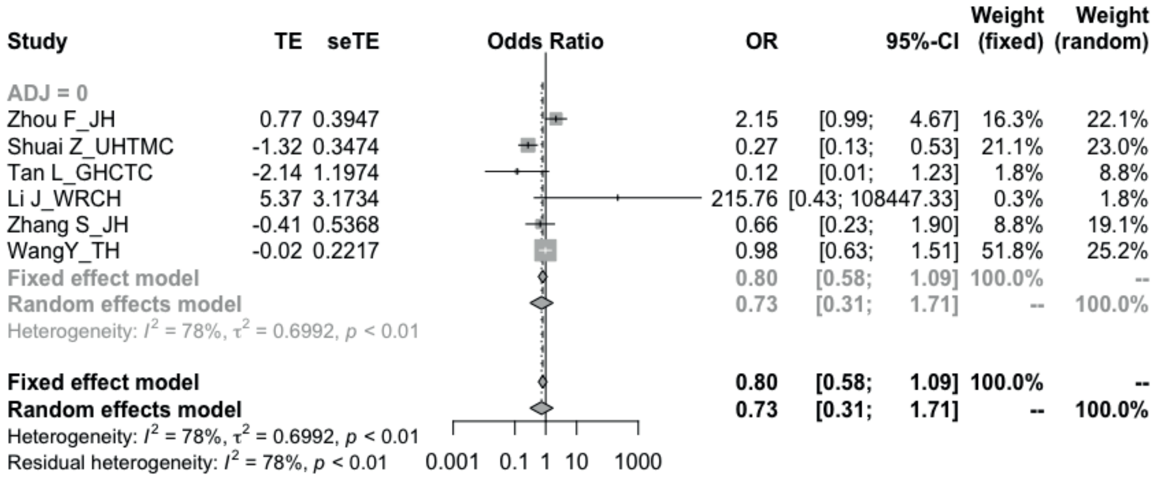

Candidate variable: Consolidation pattern. outcome: mortality

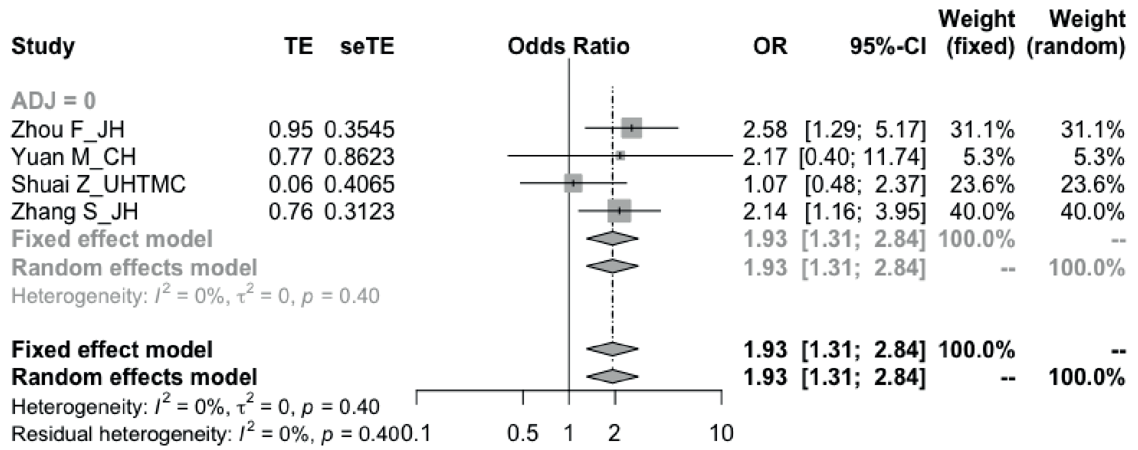

Candidate variable: Enlarged lymph nodes, outcome: mortality

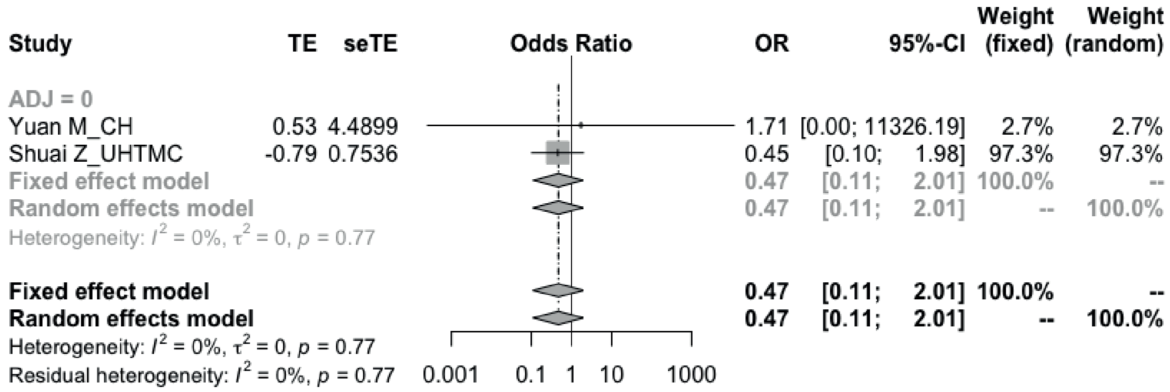

Candidate variable: Pleural effusion (X ray or CT assessment). outcome: mortality

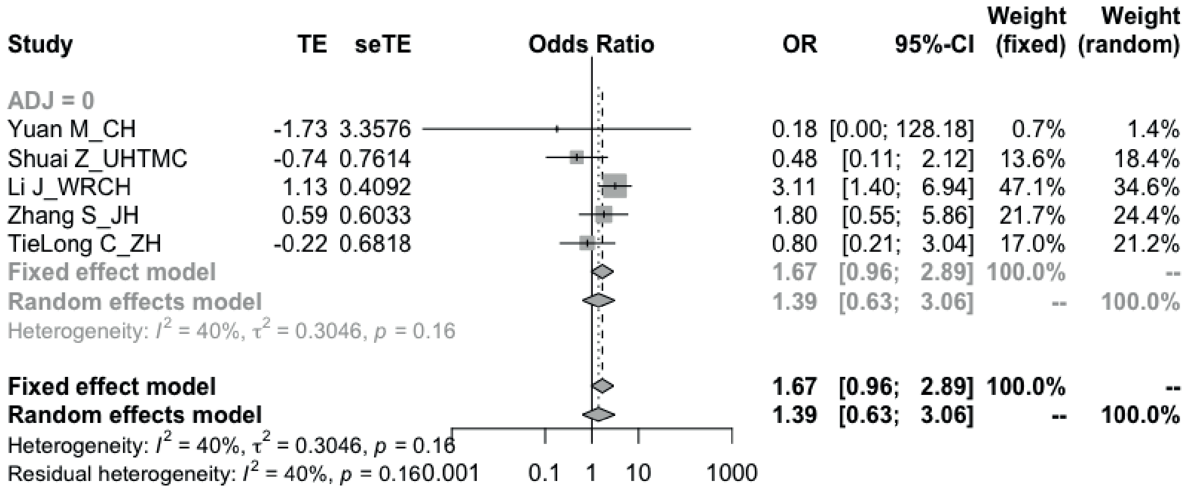

Candidate variable: Bilateral infiltrates. outcome: mortality

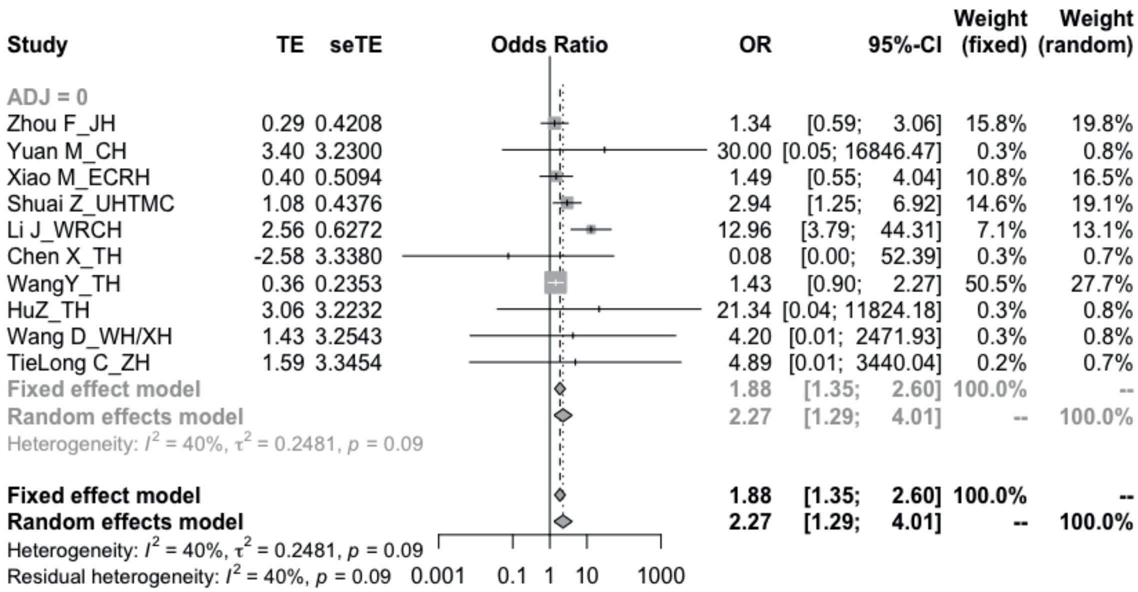

Candidate variable: High APACHE score (more than 8), outcome: mortality. subgroup analysis by risk of bias: (high vs moderate/low)

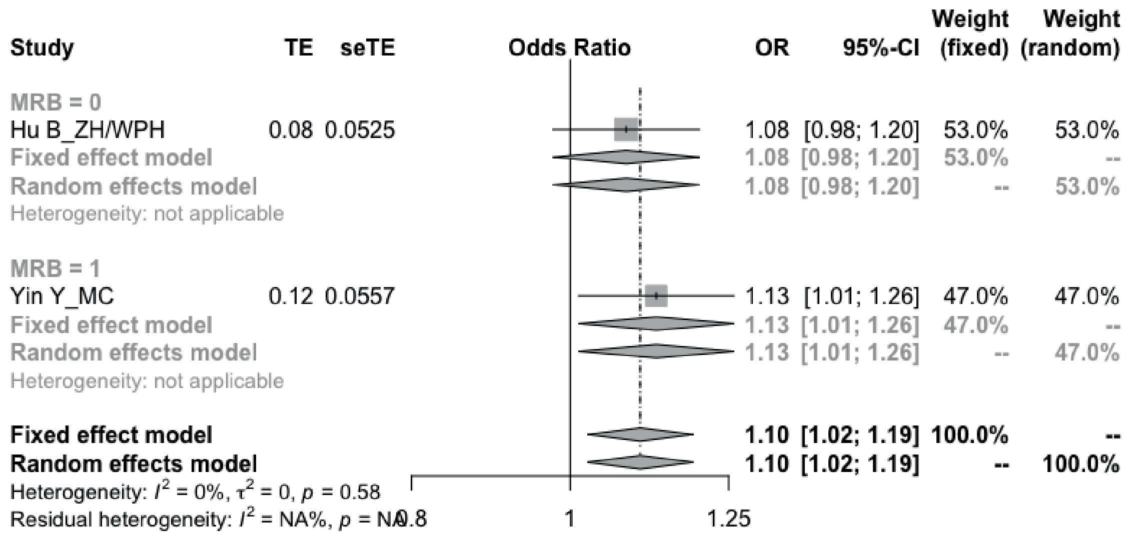

Candidate variable: High SOFA score (more than 2). outcome: mortality. subgroup analysis by risk of bias: (high vs moderate/low)

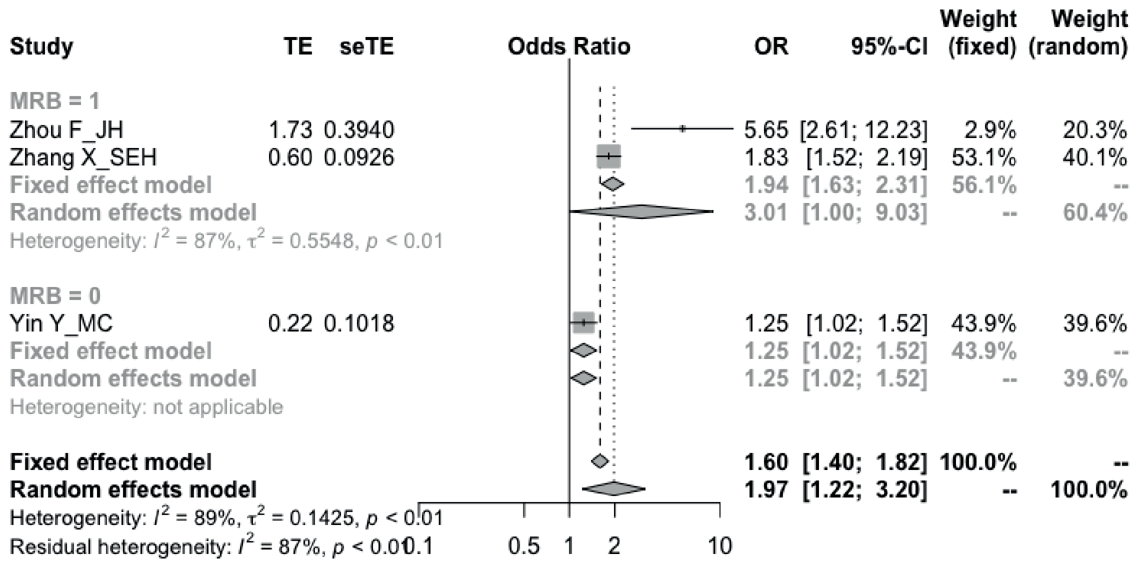

Supplement: S1 Appendix — This file contains the Forest plots for all assessed candidate variables. (PDF) [file pone.0241955.s006.pdf]
