# Supplementary material for: Prognostic factors for severity and mortality in patients infected with COVID-19: A systematic review
Source: PLoS One. 2020 Nov 17;15(11):e0241955. doi: 10.1371/journal.pone.0241955 (PMC7671522; doi:10.1371/journal.pone.0241955)

Forest plot comparison graphics: Risk of severe Covid-19 disease

Glossary

ADJ=0: crude estimate

ADJ=1: adjust estimate

LRB=0: moderate and high risk of bias

LRB=1: low risk of bias

MRB=0: High risk of bias

MRB=1: moderate and low risk of bias

ALT: Alanine aminotransferase

APTT: activated partial thromboplastin time

APACHE: Acute Physiology and Chronic Health Evaluation II

AST: Aspartate aminotransferase

BUN: Blood urea nitrogen

PT: prothrombin time

SOFA: The sequential organ failure assessment score

Candidate variable: Age (older than 50 - 65 years), outcome: severe Covid-19 disease, subgroup analysis by risk of bias (high vs moderate/low)

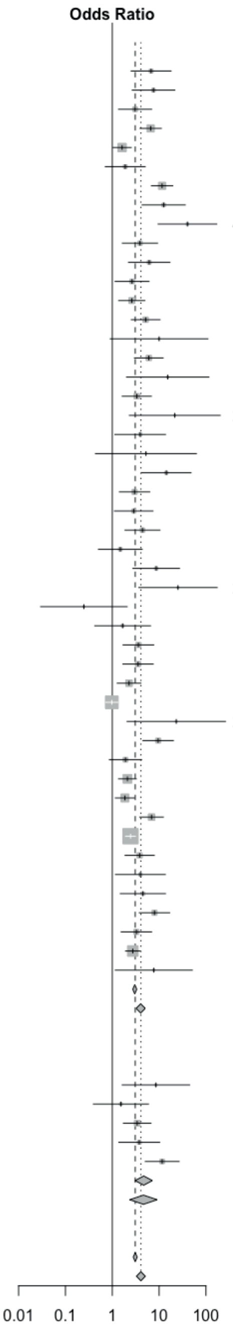

Study	TE	seTE	OR	95%-CI	Weight (fixed)	Weight (random)
**MRB = 0**						
Wu C_WJH	1.91	0.5014	6.74	[2.52; 18.01]	0.9%	1.9%
Li K_CMU	2.03	0.5349	7.59	[2.66; 21.65]	0.8%	1.8%
Jin-Jin Z_MC	1.12	0.4148	3.07	[1.36; 6.93]	1.4%	2.1%
Wei-jie G_NHC	1.89	0.2665	6.61	[3.92; 11.15]	3.3%	2.6%
Lu Jiatao_WHH	0.48	0.2289	1.62	[1.03; 2.54]	4.5%	2.7%
Cao W_ X-1 H	0.64	0.5017	1.89	[0.71; 5.05]	0.9%	1.9%
Fan T_ RHWU	2.45	0.2685	11.57	[6.84; 19.59]	3.3%	2.6%
Lian J_	2.53	0.5412	12.52	[4.34; 36.17]	0.8%	1.8%
Qi D_multicentrico	3.70	0.7352	40.30	[9.54; 170.24]	0.4%	1.3%
Sun F_ZHWU	1.36	0.4417	3.90	[1.64; 9.27]	1.2%	2.0%
Ying W_ MC	1.82	0.5198	6.15	[2.22; 17.03]	0.9%	1.8%
Shijiao Y_HHMU	0.97	0.4262	2.64	[1.15; 6.09]	1.3%	2.1%
Zhang G_ZHWU	0.96	0.3287	2.61	[1.37; 4.97]	2.2%	2.4%
Tian S_57 hospitales	1.64	0.3598	5.16	[2.55; 10.46]	1.8%	2.3%
Chen G_TH	2.30	1.2247	10.00	[0.91; 110.28]	0.2%	0.6%
Chen X_FHC/LCH	1.80	0.3600	6.03	[2.98; 12.21]	1.8%	2.3%
Hu L_TH	2.73	1.0332	15.27	[2.02; 115.73]	0.2%	0.8%
Li J_CHW	1.21	0.3659	3.34	[1.63; 6.84]	1.8%	2.3%
Wang S_ZH(Multicéntrico)	3.07	1.1390	21.60	[2.32; 201.37]	0.2%	0.7%
Xu Y_FAHG	1.37	0.6353	3.95	[1.14; 13.71]	0.6%	1.5%
Xu Y_GH	1.65	1.2674	5.23	[0.44; 62.71]	0.1%	0.6%
Zhao W_BYH	2.66	0.6193	14.29	[4.25; 48.11]	0.6%	1.5%
Yang L_SH	1.10	0.3808	2.99	[1.42; 6.32]	1.6%	2.2%
Wentao X_IDH	1.05	0.4829	2.87	[1.11; 7.40]	1.0%	1.9%
Kuang Y_MC	1.48	0.4383	4.41	[1.87; 10.42]	1.2%	2.0%
Zhang G_WXDPH	0.39	0.5493	1.48	[0.51; 4.35]	0.8%	1.7%
Cao M_SPHCC	2.16	0.5858	8.68	[2.75; 27.36]	0.7%	1.6%
Wang Y_CHW	3.23	0.9834	25.31	[3.68; 173.92]	0.2%	0.9%
Liao Xuelian_MC	-1.40	1.0849	0.25	[0.03; 2.07]	0.2%	0.8%
Chao C_NFHJCH	0.51	0.6992	1.67	[0.42; 6.56]	0.5%	1.4%
FY_JH, SPHCC, TPH	1.28	0.3855	3.61	[1.70; 7.69]	1.6%	2.2%
NS_BEMC	1.27	0.3806	3.55	[1.69; 7.49]	1.6%	2.2%
MY_multicenter 43 hosp	0.83	0.2974	2.28	[1.27; 4.09]	2.7%	2.5%
Argenziano M_NYP/CUIMC	-0.02	0.1516	0.98	[0.73; 1.31]	10.3%	2.9%
Colaneri M_PSM	3.15	1.2338	23.26	[2.07; 261.09]	0.2%	0.6%
Xin L_CHWC/hospitales en Hunan	2.26	0.3830	9.54	[4.50; 20.21]	1.6%	2.2%
Jun R_TH	0.65	0.4052	1.91	[0.87; 4.24]	1.4%	2.2%
Wang X_DFH	0.74	0.2221	2.10	[1.36; 3.25]	4.8%	2.7%
Li J_CHW	0.62	0.2391	1.86	[1.17; 2.98]	4.1%	2.7%
Zhou M_MC	1.93	0.2961	6.90	[3.86; 12.33]	2.7%	2.5%
YuC_TH	0.90	0.1255	2.45	[1.92; 3.14]	15.0%	2.9%
Xudan C_GEPH	1.35	0.3676	3.84	[1.87; 7.90]	1.7%	2.3%
Yang J_WUH	1.38	0.6252	3.99	[1.17; 13.59]	0.6%	1.5%
Yan X_HNU	1.51	0.5663	4.51	[1.49; 13.68]	0.7%	1.7%
Wu J_TFAH	2.08	0.3807	7.98	[3.78; 16.82]	1.6%	2.2%
Li H_TH	1.19	0.3835	3.27	[1.54; 6.94]	1.6%	2.2%
LiX_TH	1.01	0.1819	2.74	[1.92; 3.91]	7.1%	2.8%
Li Y_TH	2.04	0.9663	7.70	[1.16; 51.17]	0.3%	0.9%
Fixed effect model			3.02	[2.74; 3.33]	95.0%	--
Random effects model			4.04	[3.22; 5.07]	--	91.3%
Heterogeneity: $I^2 = 77\%$, $\tau^2 = 0.4045$, $p < 0.01$						
**MRB = 1**						
Liu W_MC	2.15	0.8457	8.55	[1.63; 44.85]	0.3%	1.1%
Zhang L_TH	0.43	0.6908	1.53	[0.40; 5.93]	0.5%	1.4%
Colombi D_GdSH	1.22	0.3460	3.40	[1.73; 6.70]	2.0%	2.3%
Dong J_FMC	1.33	0.5131	3.77	[1.38; 10.31]	0.9%	1.8%
Bi Q_STPH	2.45	0.4244	11.64	[5.07; 26.74]	1.3%	2.1%
Fixed effect model			4.70	[3.07; 7.19]	5.0%	--
Random effects model			4.63	[2.35; 9.11]	--	8.7%
Heterogeneity: $I^2 = 54\%$, $\tau^2 = 0.3081$, $p = 0.07$						
**Fixed effect model**			**3.09**	**[2.81; 3.40]**	**100.0%**	**--**
**Random effects model**			**4.09**	**[3.29; 5.07]**	**--**	**100.0%**
Heterogeneity: $I^2 = 76\%$, $\tau^2 = 0.4014$, $p < 0.01$						
Residual heterogeneity: $I^2 = 76\%$, $p < 0.01$						

Candidate variable: Age increase (per 1 year), outcome: severe Covid-19 disease, subgroup analysis by risk of bias (high vs moderate/low)

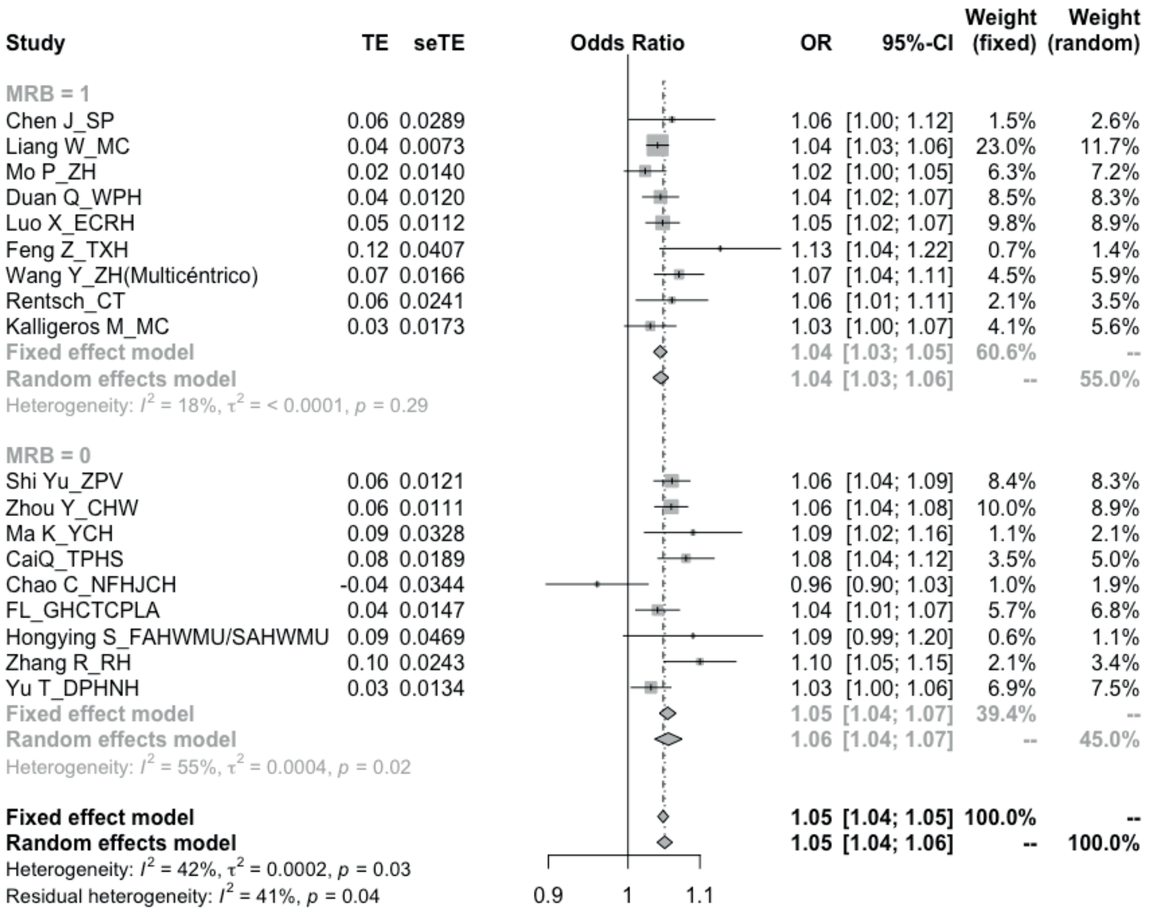

Candidate variable: Male gender, outcome: severe Covid-19 disease, subgroup analysis by risk of bias (high vs moderate/low)

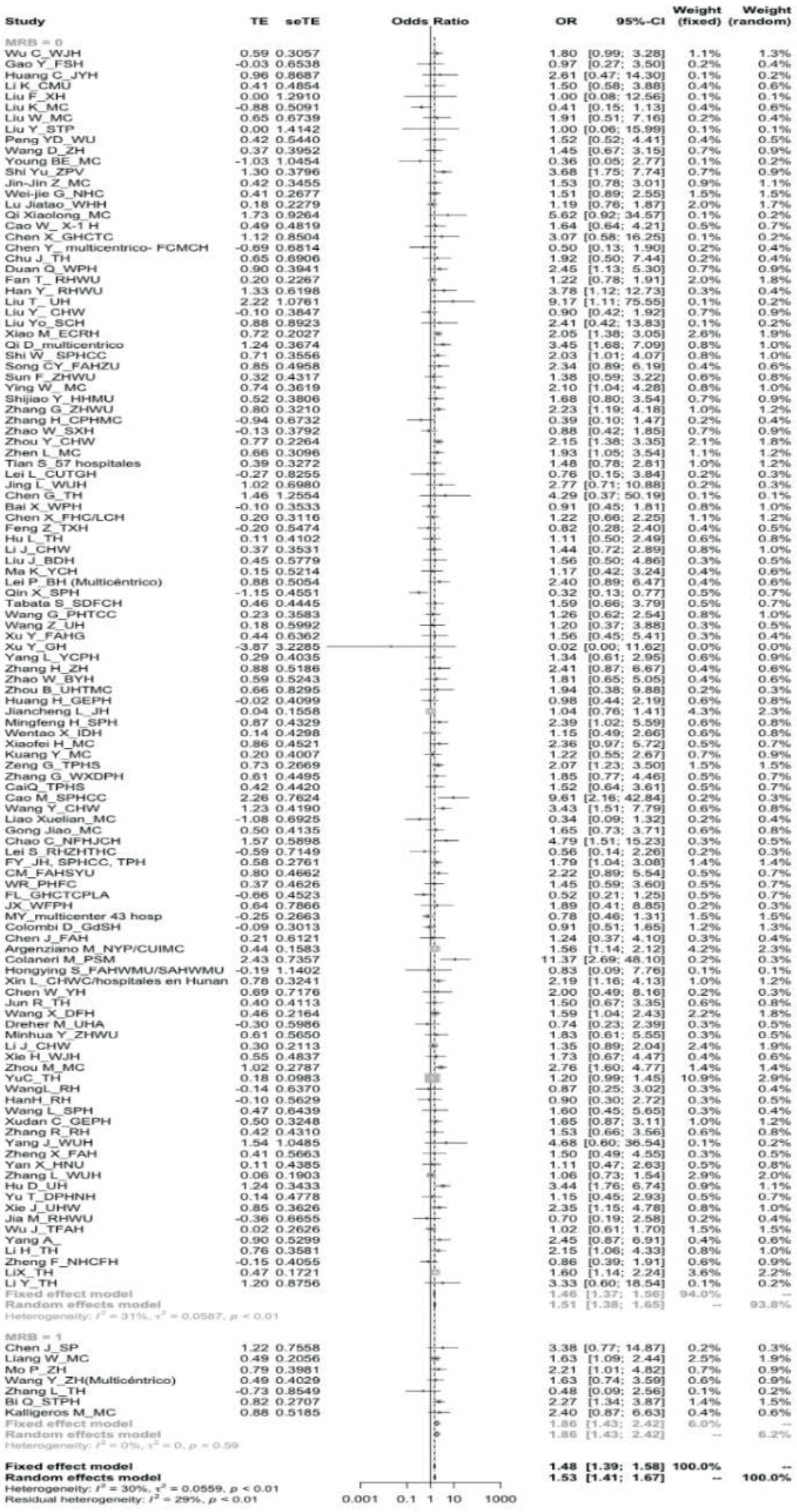

Candidate variable: Smoking (Active, present smoker), outcome: severe Covid-19 disease, subgroup analysis by risk of bias (high vs moderate/low)

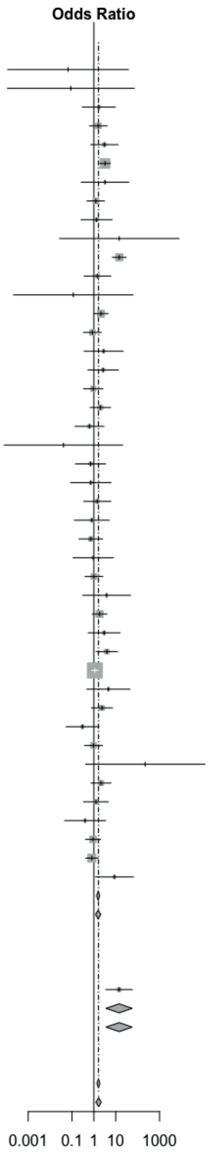

Study	TE	seTE	OR	95%-CI	Weight (fixed)	Weight (random)
**MRB = 0**						
Huang C_JYH	-2.71	3.2344	0.07	[0.00; 37.56]	0.1%	0.2%
Liu F_XH	-2.42	3.4001	0.09	[0.00; 69.67]	0.1%	0.2%
Mo P_ZH	0.52	0.8815	1.68	[0.30; 9.45]	0.8%	1.7%
Shi Yu_ZPV	0.47	0.4710	1.60	[0.64; 4.04]	3.0%	3.3%
Jin-Jin Z_MC	1.11	0.7293	3.04	[0.73; 12.69]	1.2%	2.2%
Wei-jie G_NHC	1.18	0.2769	3.25	[1.89; 5.59]	8.6%	4.4%
Chen Y_ multicentrico- FCMCH	1.17	1.2746	3.23	[0.27; 39.28]	0.4%	1.0%
Duan Q_WPH	0.20	0.4670	1.22	[0.49; 3.04]	3.0%	3.4%
Han Y_ RHWU	0.29	0.8266	1.33	[0.26; 6.74]	1.0%	1.9%
Liu T_ UH	2.67	3.1987	14.43	[0.03; 7619.45]	0.1%	0.2%
Qi D_multicentrico	2.67	0.3681	14.46	[7.03; 29.75]	4.8%	3.9%
Shi W_ SPHCC	0.38	0.7120	1.47	[0.36; 5.93]	1.3%	2.3%
Feng Z_TXH	-2.16	3.1968	0.11	[0.00; 60.50]	0.1%	0.2%
Hu L_TH	0.75	0.3817	2.12	[1.00; 4.48]	4.5%	3.8%
Li J_CHW	-0.15	0.4735	0.86	[0.34; 2.17]	2.9%	3.3%
Liu J_BDH	1.03	1.0442	2.80	[0.36; 21.68]	0.6%	1.4%
Ma K_YCH	0.97	0.8117	2.65	[0.54; 12.99]	1.0%	1.9%
Qin X_SPH	-0.07	0.5132	0.93	[0.34; 2.55]	2.5%	3.1%
Tabata S_SDFCH	0.68	0.5448	1.97	[0.68; 5.73]	2.2%	3.0%
Wang G_PHTCC	-0.46	0.7699	0.63	[0.14; 2.86]	1.1%	2.1%
Wang Y_ZH(Multicéntrico)	-3.19	3.1718	0.04	[0.00; 20.58]	0.1%	0.2%
Xu Y_FAHG	-0.35	0.8013	0.71	[0.15; 3.39]	1.0%	2.0%
Yang L_YCPH	-0.32	1.0802	0.73	[0.09; 6.05]	0.6%	1.3%
Zhang H_ZH	0.36	0.7206	1.44	[0.35; 5.89]	1.3%	2.2%
Jiancheng L_JH	-0.21	0.9332	0.81	[0.13; 5.04]	0.8%	1.6%
Kuang Y_MC	-0.32	0.6281	0.72	[0.21; 2.48]	1.7%	2.6%
Cao M_SPHCC	-0.06	1.0777	0.94	[0.11; 7.76]	0.6%	1.3%
Wang Y_CHW	0.00	0.4718	1.00	[0.40; 2.53]	3.0%	3.3%
Liao Xuelian_MC	1.34	1.2750	3.83	[0.32; 46.65]	0.4%	1.0%
FY_JH, SPHCC, TPH	0.60	0.3857	1.82	[0.86; 3.88]	4.4%	3.8%
CM_FAHSYU	1.08	0.8514	2.96	[0.56; 15.68]	0.9%	1.8%
WR_PHFC	1.37	0.5661	3.93	[1.30; 11.93]	2.0%	2.9%
Argenziano M_NYP/CUIMC	0.10	0.1762	1.11	[0.79; 1.57]	21.1%	5.0%
Hongying S_FAHWMU/SAHWMU	1.53	1.1517	4.61	[0.48; 44.06]	0.5%	1.2%
Jun R_TH	0.86	0.5612	2.35	[0.78; 7.07]	2.1%	2.9%
Dreher M_UHA	-1.19	0.8730	0.30	[0.05; 1.68]	0.9%	1.7%
Xudan C_GEPH	-0.03	0.4772	0.97	[0.38; 2.46]	2.9%	3.3%
Zhang R_RH	5.40	3.1972	221.25	[0.42; 116498.14]	0.1%	0.2%
Dong J_FMC	0.74	0.5286	2.10	[0.75; 5.93]	2.4%	3.0%
Yan X_HNU	0.22	0.6608	1.24	[0.34; 4.54]	1.5%	2.5%
Yu T_DPHNH	-0.92	1.0963	0.40	[0.05; 3.41]	0.5%	1.3%
Kalligeros M_MC	-0.08	0.3995	0.92	[0.42; 2.02]	4.1%	3.7%
LiX_TH	-0.21	0.3271	0.81	[0.43; 1.54]	6.1%	4.2%
Li Y_TH	2.17	1.0106	8.75	[1.21; 63.43]	0.6%	1.4%
**Fixed effect model**			1.57	[1.34; 1.84]	98.7%	--
**Random effects model**			1.57	[1.21; 2.05]	--	97.7%
Heterogeneity: $I^2 = 50\%$, $\tau^2 = 0.3087$, $p < 0.01$						
**MRB = 1**						
Liu W_MC	2.66	0.7045	14.28	[3.59; 56.80]	1.3%	2.3%
**Fixed effect model**			14.28	[3.59; 56.80]	1.3%	--
**Random effects model**			14.28	[3.59; 56.80]	--	2.3%
Heterogeneity: not applicable						
**Fixed effect model**			**1.62**	**[1.38; 1.89]**	**100.0%**	**--**
**Random effects model**			**1.65**	**[1.25; 2.17]**	**--**	**100.0%**
Heterogeneity: $I^2 = 54\%$, $\tau^2 = 0.3656$, $p < 0.01$						
Residual heterogeneity: $I^2 = 50\%$, $p < 0.01$						

Candidate variable: Cardiovascular disease (coronary heart disease or congestive heart failure), outcome: severe Covid-19 disease, subgroup analysis by risk of bias (high vs moderate/low)

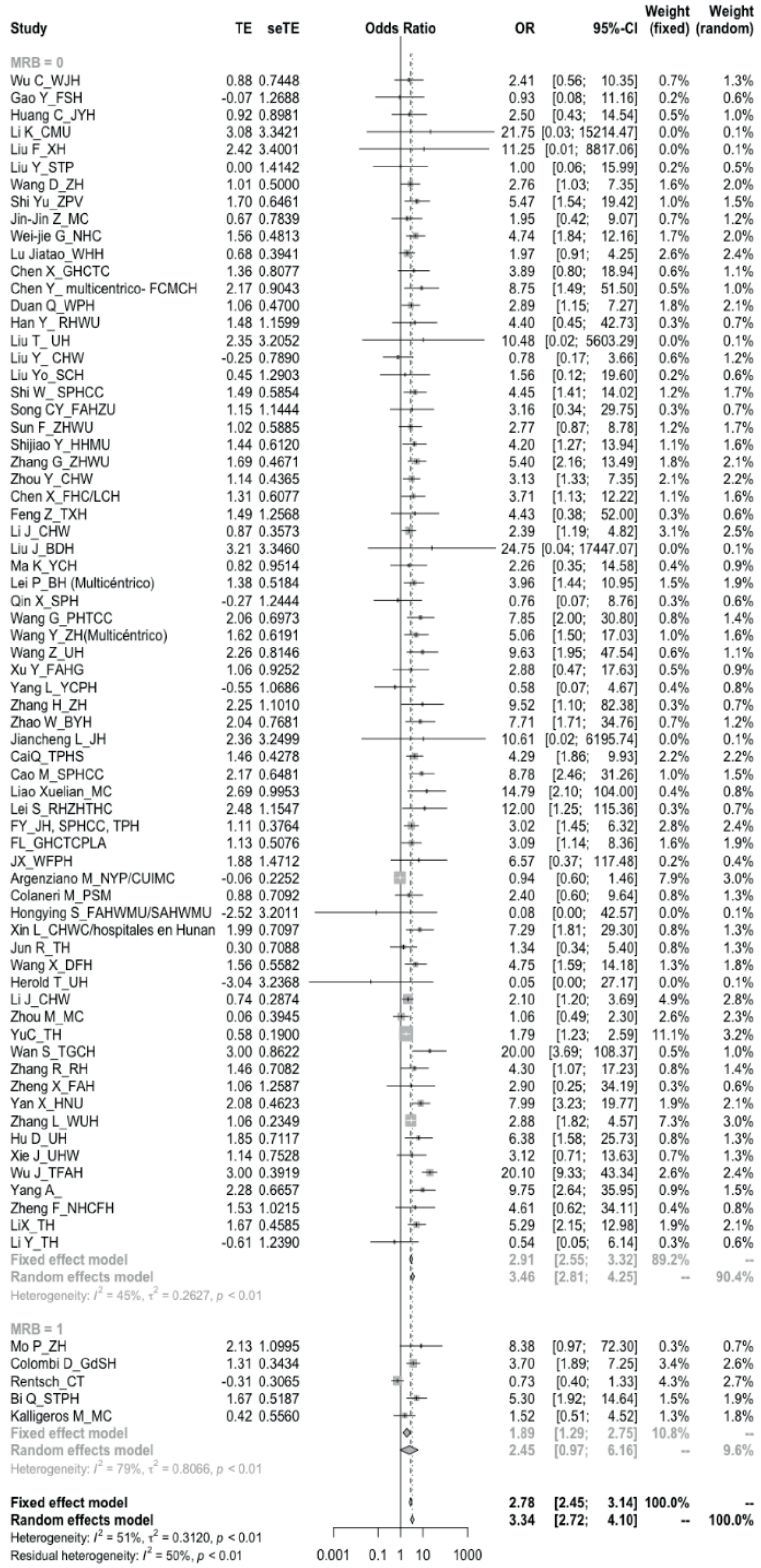

Candidate variable: Cardiac arrhythmia (as previous condition or new clinical finding), outcome: severe Covid-19 disease

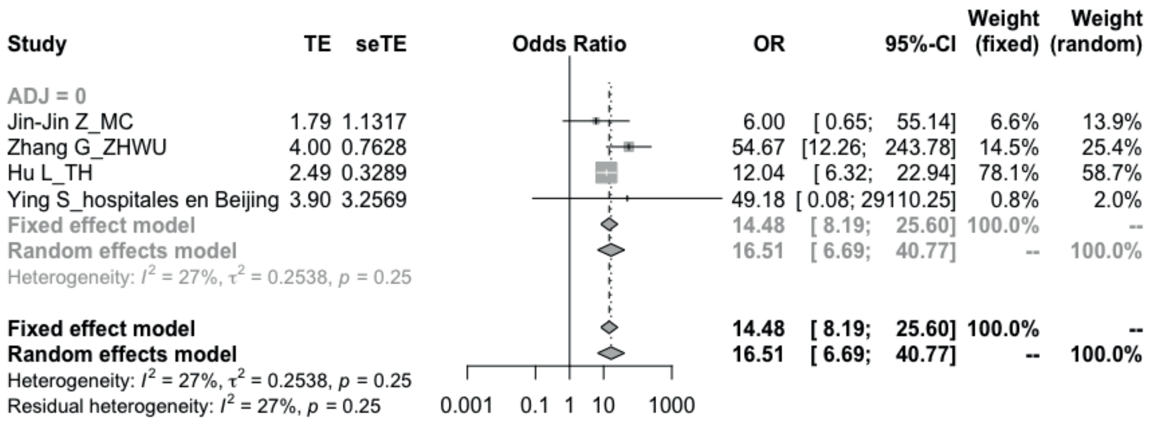

Candidate variable: Cerebrovascular disease (History of stroke or CNS disease), outcome: severe Covid-19 disease, subgroup analysis: (crude vs adjusted)

Study	TE	seTE	OR	95%-CI	Weight (fixed)	Weight (random)
**ADJ = 0**						
Mo P_ZH	-2.32	1.2786	0.10	[0.01; 1.20]	0.5%	1.6%
Wang D_ZH	3.01	1.0999	20.20	[2.34; 174.43]	0.7%	2.0%
Jin-Jin Z_MC	1.06	1.2370	2.89	[0.26; 32.68]	0.6%	1.7%
Wei-jie G_NHC	1.77	0.5981	5.89	[1.82; 19.03]	2.4%	3.8%
Lu Jiatao_WHH	0.82	0.9200	2.28	[0.38; 13.83]	1.0%	2.5%
Chen X_GHCTC	1.50	0.9281	4.46	[0.72; 27.51]	1.0%	2.5%
Chen Y_ multicentrico- FCMCH	1.17	1.2746	3.23	[0.27; 39.28]	0.5%	1.6%
Liu Y_ CHW	4.27	3.1946	71.49	[0.14; 37452.78]	0.1%	0.3%
Sun F_ZHWU	-2.45	3.1961	0.09	[0.00; 45.56]	0.1%	0.3%
Zhang G_ZHWU	2.32	0.6081	10.13	[3.07; 33.34]	2.4%	3.7%
Chen X_FHC/LCH	1.10	0.7475	3.01	[0.70; 13.04]	1.6%	3.1%
Feng Z_TXH	-0.08	3.3445	0.93	[0.00; 650.70]	0.1%	0.3%
Hu L_TH	-1.82	3.1916	0.16	[0.00; 84.20]	0.1%	0.3%
Li J_CHW	0.18	0.4593	1.20	[0.49; 2.96]	4.1%	4.4%
Ma K_YCH	1.24	1.0352	3.44	[0.45; 26.20]	0.8%	2.1%
Lei P_BH (Multicéntrico)	-0.96	3.3417	0.38	[0.00; 266.75]	0.1%	0.3%
Wang G_PHTCC	1.05	0.8852	2.87	[0.51; 16.28]	1.1%	2.6%
Wang Y_ZH(Multicéntrico)	1.14	0.8150	3.13	[0.63; 15.44]	1.3%	2.8%
Zhang H_ZH	0.53	0.9477	1.71	[0.27; 10.94]	1.0%	2.4%
Zhao W_BYH	1.08	1.4389	2.95	[0.18; 49.46]	0.4%	1.3%
Jiancheng L_JH	-0.22	0.6753	0.80	[0.21; 3.01]	1.9%	3.4%
Mingfeng H_SPH	-1.62	3.2505	0.20	[0.00; 115.37]	0.1%	0.3%
Wang Y_CHW	0.38	0.7916	1.46	[0.31; 6.88]	1.4%	2.9%
Lei S_RHZHTHC	3.38	3.2602	29.23	[0.05; 17416.52]	0.1%	0.3%
FY_JH, SPHCC, TPH	1.74	0.5047	5.69	[2.12; 15.31]	3.4%	4.2%
JX_WFPH	3.94	3.3584	51.43	[0.07; 37140.02]	0.1%	0.3%
Colombi D_GdSH	0.69	0.3535	2.00	[1.00; 4.00]	7.0%	5.0%
Argenziano M_NYP/CUIMC	0.06	0.2746	1.06	[0.62; 1.81]	11.5%	5.3%
Xin L_CHWC/hospitales en Hunan	1.78	0.8810	5.92	[1.05; 33.29]	1.1%	2.6%
Ying S_hospitales en Beijing	3.90	3.2569	49.18	[0.08; 29110.25]	0.1%	0.3%
Chen W_YH	4.29	3.3543	73.12	[0.10; 52386.57]	0.1%	0.3%
Dreher M_UHA	0.43	0.8227	1.53	[0.31; 7.69]	1.3%	2.8%
Li J_CHW	1.35	0.2992	3.86	[2.15; 6.94]	9.7%	5.2%
Zhou M_MC	-0.17	0.3990	0.84	[0.38; 1.84]	5.5%	4.7%
YuC_TH	0.25	0.2729	1.28	[0.75; 2.19]	11.7%	5.3%
WangL_RH	1.49	0.9061	4.44	[0.75; 26.25]	1.1%	2.5%
Wang L_SPH	-0.43	3.3468	0.65	[0.00; 458.56]	0.1%	0.3%
Zheng X_FAH	0.32	1.4429	1.38	[0.08; 23.36]	0.4%	1.3%
Yan X_HNU	1.46	0.8948	4.32	[0.75; 24.94]	1.1%	2.6%
Zhang L_WUH	2.07	0.8076	7.90	[1.62; 38.48]	1.3%	2.9%
Zheng F_NHCFH	0.39	1.1729	1.47	[0.15; 14.66]	0.6%	1.8%
**Fixed effect model**			2.07	[1.69; 2.54]	79.4%	--
**Random effects model**			2.38	[1.75; 3.24]	--	94.4%
Heterogeneity: $I^2 = 35\%$, $\tau^2 = 0.2518$, $p = 0.02$						
**ADJ = 1**						
Bi Q_STPH	2.22	0.2056	9.23	[6.17; 13.81]	20.6%	5.6%
**Fixed effect model**			9.23	[6.17; 13.81]	20.6%	--
**Random effects model**			9.23	[6.17; 13.81]	--	5.6%
Heterogeneity: not applicable						
**Fixed effect model**			**2.82**	**[2.35; 3.38]**	**100.0%**	**--**
**Random effects model**			**2.67**	**[1.84; 3.87]**	**--**	**100.0%**
Heterogeneity: $I^2 = 60\%$, $\tau^2 = 0.5955$, $p < 0.01$						
Residual heterogeneity: $I^2 = 35\%$, $p = 0.02$						

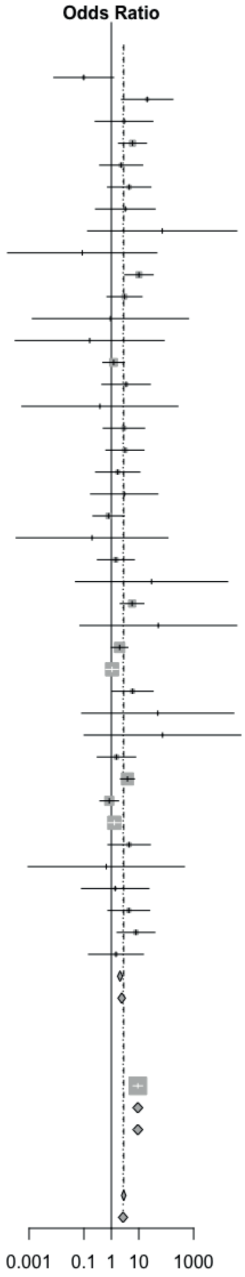

Candidate variable: Diabetes, outcome: severe Covid-19 disease, subgroup analysis by risk of bias (high vs moderate/low)

Stud y	TE	seTE	OR	95%−CI	Weight (fixed)	Weight (random)
MRB = 0						
Wu C_WJH	1.47	0.5029	4.35	[1.62; 11.66]	0.9%	1.3%
Gao Y_FSH	2.89	1.1466	18.00	[1.90; 170.33]	0.2%	0.3%
Huang C_JYH	−1.39	1.1286	0.25	[0.03; 2.28]	0.2%	0.3%
Li K_CMU	5.40	3.1965	222.33	[0.42; 116905.58]	0.0%	0.0%
Liu F_XH	2.20	10.5597	9.00	[0.00; 8763252695.66]	0.0%	0.0%
Liu W_MC	1.56	0.9816	4.74	[0.69; 32.45]	0.2%	0.4%
Liu Y_STP	1.10	1.6330	3.00	[0.12; 73.64]	0.1%	0.2%
Peng YD_WU	0.30	0.6316	1.35	[0.39; 4.66]	0.6%	0.9%
Shi Yu_ZPV	1.15	0.4632	3.15	[1.27; 7.81]	1.1%	1.4%
Jin−Jin Z_MC	0.26	0.5194	1.30	[0.47; 3.59]	0.9%	1.2%
Wei−jie G_NHC	1.73	0.3047	5.65	[3.11; 10.27]	2.5%	2.3%
Lu Jiatao_WHH	0.05	0.3998	1.05	[0.48; 2.31]	1.5%	1.7%
Chen X_GHCTC	1.29	0.6938	3.64	[0.93; 14.18]	0.5%	0.8%
Chen Y_ m ulticentr ico− FCMCH	0.21	0.7744	1.24	[0.27; 5.64]	0.4%	0.7%
Duan Q_WPH	1.15	0.4533	3.17	[1.30; 7.71]	1.1%	1.5%
Han Y_ RHWU	0.29	0.8266	1.33	[0.26; 6.74]	0.3%	0.6%
Liu T_ UH	3.04	3.1935	20.86	[0.04; 10906.91]	0.0%	0.0%
Liu Y_ CHW	2.67	1.0644	14.40	[1.79; 116.01]	0.2%	0.4%
Liu Yo_SCH	−0.20	0.9142	0.82	[0.14; 4.90]	0.3%	0.5%
Qi D_m ulticentr ico	1.52	0.4313	4.58	[1.97; 10.66]	1.3%	1.6%
Shi W_ SPHCC	−0.18	0.8095	0.83	[0.17; 4.06]	0.4%	0.6%
Song CY_F AHZU	0.42	0.9004	1.53	[0.26; 8.91]	0.3%	0.5%
Sun F_ZHWU	1.09	0.6543	2.98	[0.83; 10.73]	0.5%	0.9%
Shijiao Y_HHMU	1.81	0.6206	6.13	[1.82; 20.69]	0.6%	0.9%
Zhang G_ZHWU	0.38	0.4868	1.47	[0.57; 3.81]	1.0%	1.3%
Zhang H_CPHMC	0.86	0.8922	2.36	[0.41; 13.58]	0.3%	0.5%
Zhang Y_WCUH	0.86	0.3272	2.37	[1.25; 4.50]	2.2%	2.2%
Zhou Y_CHW	1.07	0.2560	2.91	[1.76; 4.80]	3.6%	2.7%
Lei L_CUTGH	6.35	3.2576	572.00	[0.96; 339067.57]	0.0%	0.0%
Jing L_WUH	1.71	0.9493	5.56	[0.86; 35.71]	0.3%	0.5%
Chen G_TH	0.69	1.3123	2.00	[0.15; 26.19]	0.1%	0.3%
Bai X_WPH	0.70	0.5117	2.02	[0.74; 5.50]	0.9%	1.3%
Chen X_FHC/LCH	0.90	0.4870	2.45	[0.94; 6.37]	1.0%	1.3%
Feng Z_TXH	1.12	0.8671	3.08	[0.56; 16.83]	0.3%	0.5%
Hu L_TH	1.27	0.3396	3.58	[1.84; 6.96]	2.0%	2.1%
Li J_CHW	0.48	0.3992	1.62	[0.74; 3.54]	1.5%	1.7%
Liu J_BDH	1.50	0.9636	4.50	[0.68; 29.75]	0.3%	0.4%
Ma K_YCH	2.39	0.7546	10.95	[2.49; 48.05]	0.4%	0.7%
Lei P_BH (Multicéntr ico)	0.95	0.6948	2.60	[0.67; 10.14]	0.5%	0.8%
Qin X_SPH	2.04	0.8254	7.70	[1.53; 38.84]	0.3%	0.6%
Wang G_PHTCC	0.76	0.6135	2.14	[0.64; 7.11]	0.6%	1.0%
Wang Y_ZH(Multicéntr ico)	1.09	0.4247	2.97	[1.29; 6.83]	1.3%	1.6%
Wang Z_UH	3.70	1.1446	40.50	[4.30; 381.73]	0.2%	0.3%
Xu Y_FAHG	0.10	0.6607	1.10	[0.30; 4.02]	0.5%	0.9%
Yang L_YCPH	0.37	0.5961	1.45	[0.45; 4.66]	0.7%	1.0%
Zhang H_ZH	1.11	0.6609	3.03	[0.83; 11.08]	0.5%	0.9%
Zhao W_BYH	0.39	0.9080	1.47	[0.25; 8.73]	0.3%	0.5%
Huang H_GEPH	2.35	0.8462	10.50	[2.00; 55.14]	0.3%	0.6%
Jiancheng L_JH	1.40	0.7851	4.04	[0.87; 18.81]	0.4%	0.6%
Mingfeng H_SPH	2.58	0.7130	13.22	[3.27; 53.48]	0.5%	0.8%
Wentao X_IDH	3.99	3.2020	54.03	[0.10; 28715.76]	0.0%	0.0%
Kuang Y_MC	0.97	0.6231	2.63	[0.77; 8.91]	0.6%	0.9%
Zeng G_TPHS	1.46	0.4244	4.30	[1.87; 9.88]	1.3%	1.6%
CaiQ_TPHS	1.30	0.4993	3.68	[1.38; 9.79]	0.9%	1.3%
Cao M_SPHCC	0.41	0.8011	1.50	[0.31; 7.22]	0.4%	0.6%
Wang Y_CHW	0.91	0.5626	2.48	[0.82; 7.46]	0.7%	1.1%
Liao Xuelian_MC	0.47	0.7488	1.60	[0.37; 6.94]	0.4%	0.7%
Chao C_NFHJCH	2.77	3.3277	15.91	[0.02; 10819.14]	0.0%	0.0%
Lei S_RHZHTHC	1.73	0.9146	5.67	[0.94; 34.03]	0.3%	0.5%
FY_JH, SPHCC , TPH	−0.23	0.4564	0.79	[0.32; 1.94]	1.1%	1.5%
CM_FAHSYU	1.84	0.9000	6.27	[1.08; 36.60]	0.3%	0.5%
FL_GHCTCPLA	0.66	0.4651	1.94	[0.78; 4.83]	1.1%	1.4%
JX_WFPH	2.13	0.8508	8.40	[1.59; 44.51]	0.3%	0.6%
MY_multicenter 43 hosp	0.68	0.4696	1.97	[0.79; 4.95]	1.1%	1.4%
Colombi D_GdSH	0.66	0.3641	1.93	[0.94; 3.93]	1.8%	1.9%
Chen J_F AH	1.84	1.1173	6.30	[0.71; 56.28]	0.2%	0.3%
Argenziano M_NYP/CUIMC	0.33	0.1527	1.39	[1.03; 1.88]	10.0%	3.5%
Colaner i M_PSM	−1.52	1.1299	0.22	[0.02; 2.00]	0.2%	0.3%
Hongying S_F AHWMU/SAHWMU	−0.06	0.6769	0.94	[0.25; 3.55]	0.5%	0.8%
Xin L_CHWC/hospitales en Hunan	1.27	0.5806	3.57	[1.14; 11.15]	0.7%	1.0%
Ying S_hospitales en Beijing	1.37	0.9590	3.94	[0.60; 25.79]	0.3%	0.5%
Chen W_YH	2.91	1.2878	18.29	[1.47; 228.18]	0.1%	0.3%
Jun R_TH	0.59	0.4641	1.80	[0.72; 4.47]	1.1%	1.4%
Wang X_DFH	1.21	0.4525	3.36	[1.38; 8.15]	1.1%	1.5%
Herold T_UH	1.57	1.2813	4.80	[0.39; 59.14]	0.1%	0.3%
Dreher M_UHA	0.36	0.5767	1.43	[0.46; 4.42]	0.7%	1.1%
Minhua Y_ZHWU	1.10	0.6992	3.00	[0.76; 11.81]	0.5%	0.8%
Li J_CHW	0.75	0.2243	2.12	[1.37; 3.29]	4.6%	2.9%
Zhou M_MC	0.55	0.3160	1.73	[0.93; 3.21]	2.3%	2.2%
YuC_TH	0.65	0.1443	1.91	[1.44; 2.53]	11.2%	3.6%
WangL_RH	1.31	0.6894	3.71	[0.96; 14.34]	0.5%	0.8%
Wang L_SPH	−0.43	3.3468	0.65	[0.00; 458.56]	0.0%	0.0%
Wan S_TGCH	2.33	0.7784	10.31	[2.24; 47.42]	0.4%	0.7%
Zhang R_RH	5.60	3.1936	270.00	[0.52; 141178.69]	0.0%	0.0%
Yan X_HNU	2.01	0.4857	7.44	[2.87; 19.27]	1.0%	1.3%
Zhang L_WUH	0.49	0.2314	1.63	[1.03; 2.56]	4.4%	2.9%
Hu D_UH	0.89	0.4929	2.43	[0.92; 6.38]	1.0%	1.3%
Xie J_UHW	0.18	0.4945	1.19	[0.45; 3.15]	1.0%	1.3%
Yang A_	2.20	0.5561	9.01	[3.03; 26.80]	0.8%	1.1%
Zheng F_NHCFH	0.59	0.8623	1.80	[0.33; 9.76]	0.3%	0.5%
LiX_TH	0.65	0.2452	1.92	[1.19; 3.10]	3.9%	2.7%
Li Y_TH	2.89	3.3613	18.00	[0.02; 13073.69]	0.0%	0.0%
Fixed eff ect model			2.29	[2.07; 2.53]	91.2%	--
Random eff ects model			2.56	[2.21; 2.95]	--	91.5%
Heterogeneity: I^2 = 34% , ^{2} = 0.1268 , p < 0.01						
MRB = 1						
Liang W_MC	0.79	0.2582	2.21	[1.33; 3.67]	3.5%	2.6%
Mo P_ZH	0.76	0.7607	2.14	[0.48; 9.51]	0.4%	0.7%
Rentsch_CT	0.52	0.2890	1.69	[0.96; 2.98]	2.8%	2.4%
Bi Q_STPH	1.36	0.4447	3.90	[1.63; 9.33]	1.2%	1.5%
Kalligeros M_MC	0.65	0.5075	1.91	[0.71; 5.16]	0.9%	1.3%
Fixed eff ect model			2.15	[1.57; 2.97]	8.8%	--
Random eff ects model			2.15	[1.57; 2.97]	--	8.5%
Heterogeneity: I^2 = 0% , ^{2} = 0 , p = 0.63						
**Fixed eff ect model**			**2.28**	**[2.07; 2.50]**	**100.0%**	**--**
**Random eff ects model**			**2.51**	**[2.20; 2.87]**	**--**	**100.0%**

Heterogeneity: I^2 = 32% , ^{2} = 0.1094 , p < 0.01
Residual heterogeneity: I^2 = 33% , p < 0.01

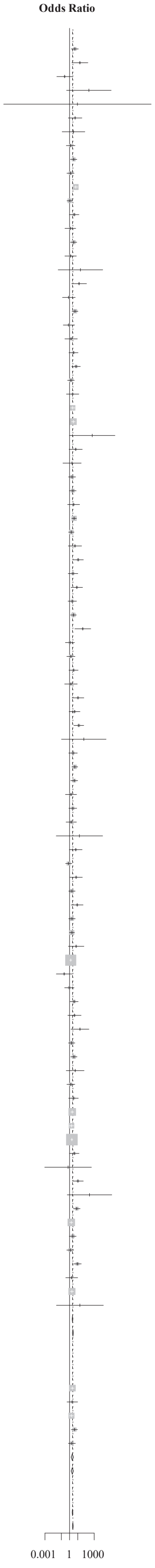

Candidate variable: Arterial hypertension, outcome: severe Covid-19 disease, subgroup analysis by risk of bias (high vs moderate/low)

Study	TE	seTE	Odds Ratio	OR	95%-CI	Weight (fixed)	Weight (random)
**MRB = 0**							
Wu C_WJH	0.87	0.3637		2.38	[1.17; 4.85]	1.3%	1.5%
Gao Y_FSH	0.69	0.6843		2.00	[0.52; 7.65]	0.4%	0.8%
Huang C_JYH	0.09	0.9394		1.09	[0.17; 6.88]	0.2%	0.5%
Li K_CMU	0.47	0.9460		1.59	[0.25; 10.18]	0.2%	0.5%
Liu W_MC	0.82	0.8937		2.26	[0.39; 13.03]	0.2%	0.5%
Liu Y_STP	0.56	1.4638		1.75	[0.10; 30.84]	0.1%	0.2%
Mo P_ZH	0.25	0.3823		1.28	[0.61; 2.71]	1.2%	1.4%
Wang D_ZH	1.63	0.4150		5.09	[2.26; 11.48]	1.0%	1.3%
Shi Yu_ZPV	1.00	0.3682		2.71	[1.32; 5.58]	1.3%	1.5%
Jin-Jin Z_MC	0.64	0.3733		1.89	[0.91; 3.94]	1.2%	1.5%
Wei-jie G_NHC	1.26	0.2704		3.53	[2.08; 5.99]	2.4%	1.8%
Lu Jiatao_WHH	0.46	0.2718		1.58	[0.93; 2.69]	2.3%	1.8%
Qi Xiaolong_MC	-1.15	1.4793		0.32	[0.02; 5.78]	0.1%	0.2%
Chen X_GHCTC	1.47	0.6513		4.36	[1.22; 15.64]	0.4%	0.8%
Chen Y_ multicentrico- FCMCH	1.46	0.7138		4.29	[1.06; 17.36]	0.3%	0.7%
Fan T_ RHWU	0.60	0.3919		1.83	[0.85; 3.94]	1.1%	1.4%
Han Y_ RHWU	0.29	0.6026		1.34	[0.41; 4.36]	0.5%	0.9%
Liu T_ UH	3.33	3.1906		28.00	[0.05; 14555.62]	0.0%	0.0%
Liu Y_ CHW	0.50	0.4079		1.64	[0.74; 3.65]	1.0%	1.3%
Liu Yo_SCH	-0.90	1.1557		0.41	[0.04; 3.93]	0.1%	0.3%
Qi D_multicentrico	2.36	0.5016		10.54	[3.94; 28.17]	0.7%	1.1%
Shi W_ SPHCC	0.66	0.3749		1.94	[0.93; 4.04]	1.2%	1.4%
Song CY_FAHZU	2.00	0.6184		7.43	[2.21; 24.95]	0.5%	0.9%
Sun F_ZHWU	1.38	0.4456		3.96	[1.66; 9.49]	0.9%	1.2%
Shijiao Y_HHMU	1.39	0.4650		4.03	[1.62; 10.02]	0.8%	1.2%
Zhang G_ZHWU	1.49	0.3404		4.42	[2.27; 8.61]	1.5%	1.6%
Zhang H_CPHMC	-0.41	1.2036		0.67	[0.06; 7.05]	0.1%	0.3%
Zhou Y_CHW	1.54	0.2377		4.66	[2.92; 7.42]	3.1%	1.9%
Lei L_CUTGH	0.82	1.2346		2.28	[0.20; 25.61]	0.1%	0.3%
Jing L_WUH	2.79	1.1677		16.25	[1.65; 160.24]	0.1%	0.3%
Chen G_TH	1.64	1.2263		5.14	[0.46; 56.89]	0.1%	0.3%
Bai X_WPH	1.44	0.4545		4.22	[1.73; 10.29]	0.8%	1.2%
Chen X_FHC/LCH	1.91	0.3734		6.77	[3.26; 14.08]	1.2%	1.5%
Feng Z_TXH	1.60	0.5945		4.93	[1.54; 15.82]	0.5%	0.9%
Hu L_TH	0.28	0.4219		1.33	[0.58; 3.04]	1.0%	1.3%
Li J_CHW	0.50	0.3707		1.64	[0.79; 3.40]	1.3%	1.5%
Liu J_BDH	1.24	0.6712		3.45	[0.93; 12.87]	0.4%	0.8%
Ma K_YCH	0.56	0.6748		1.75	[0.47; 6.57]	0.4%	0.8%
Qin X_SPH	2.18	0.6926		8.87	[2.28; 34.47]	0.4%	0.7%
Wang G_PHTCC	1.62	0.4072		5.06	[2.28; 11.25]	1.0%	1.4%
Wang Z_UH	1.96	0.7620		7.08	[1.59; 31.54]	0.3%	0.6%
Xu Y_FAHG	0.61	0.6072		1.83	[0.56; 6.03]	0.5%	0.9%
Yang L_YCPH	0.52	0.4430		1.69	[0.71; 4.02]	0.9%	1.3%
Zhang H_ZH	1.46	0.5263		4.32	[1.54; 12.13]	0.6%	1.0%
Zhao W_BYH	1.41	0.5948		4.08	[1.27; 13.10]	0.5%	0.9%
Huang H_GEPH	2.13	0.5329		8.41	[2.96; 23.89]	0.6%	1.0%
Jiancheng L_JH	0.71	0.4014		2.03	[0.93; 4.46]	1.1%	1.4%
Mingfeng H_SPH	1.34	0.4812		3.81	[1.48; 9.79]	0.7%	1.2%
Wentao X_IDH	4.20	3.1967		66.76	[0.13; 35119.29]	0.0%	0.0%
Kuang Y_MC	0.12	0.5594		1.13	[0.38; 3.39]	0.6%	1.0%
Zeng G_TPHS	1.08	0.3216		2.95	[1.57; 5.55]	1.7%	1.6%
CaiQ_TPHS	0.38	0.5354		1.46	[0.51; 4.17]	0.6%	1.0%
Cao M_SPHCC	0.61	0.5276		1.83	[0.65; 5.16]	0.6%	1.0%
Wang Y_CHW	1.65	0.5008		5.22	[1.96; 13.92]	0.7%	1.1%
Liao Xuelian_MC	1.29	0.7240		3.64	[0.88; 15.03]	0.3%	0.7%
Chao C_NFHJCH	0.77	0.7333		2.15	[0.51; 9.06]	0.3%	0.7%
Lei S_RHZHTHC	1.73	0.7710		5.62	[1.24; 25.49]	0.3%	0.6%
FY_JH, SPHCC, TPH	-0.14	0.2694		0.87	[0.51; 1.48]	2.4%	1.8%
CM_FAHSYU	0.60	0.5771		1.83	[0.59; 5.67]	0.5%	0.9%
FL_GHCTCPLA	0.99	0.5088		2.68	[0.99; 7.26]	0.7%	1.1%
JX_WFPH	0.35	0.7969		1.41	[0.30; 6.74]	0.3%	0.6%
MY_multicenter 43 hosp	0.51	0.3173		1.67	[0.90; 3.12]	1.7%	1.6%
Chen J_FAH	0.81	0.6492		2.24	[0.63; 7.99]	0.4%	0.8%
Argenziano M_NYP/CUIMC	0.46	0.1587		1.58	[1.16; 2.15]	6.9%	2.1%
Colaneri M_PSM	-0.34	0.6649		0.71	[0.19; 2.61]	0.4%	0.8%
Hongying S_FAHWMU/SAHWMU	0.31	0.4710		1.36	[0.54; 3.43]	0.8%	1.2%
Xin L_CHWC/hospitales en Hunan	2.81	0.5847		16.66	[5.30; 52.42]	0.5%	0.9%
Ying S_hospitales en Beijing	0.18	0.6851		1.20	[0.31; 4.60]	0.4%	0.8%
Chen W_YH	3.23	0.9828		25.20	[3.67; 172.96]	0.2%	0.4%
Jun R_TH	1.10	0.4154		3.00	[1.33; 6.77]	1.0%	1.3%
Yang G_HPHTCM	0.49	0.2686		1.63	[0.96; 2.75]	2.4%	1.8%
Wang X_DFH	0.33	0.4510		1.39	[0.57; 3.37]	0.9%	1.2%
Herold T_UH	1.91	0.8819		6.75	[1.20; 38.02]	0.2%	0.5%
Dreher M_UHA	-0.31	0.6188		0.74	[0.22; 2.48]	0.5%	0.9%
Zhou M_MC	0.54	0.2906		1.71	[0.97; 3.02]	2.1%	1.7%
YuC_TH	0.36	0.1223		1.44	[1.13; 1.83]	11.6%	2.2%
WangL_RH	0.38	0.6387		1.47	[0.42; 5.14]	0.4%	0.8%
ZengZ_HH	-0.15	0.2756		0.86	[0.50; 1.47]	2.3%	1.8%
Wang L_SPH	0.95	0.7568		2.59	[0.59; 11.41]	0.3%	0.7%
Wan S_TGCH	0.98	0.7523		2.67	[0.61; 11.65]	0.3%	0.7%
Zhang R_RH	2.37	0.5606		10.71	[3.57; 32.12]	0.6%	1.0%
Zheng X_FAH	1.83	0.7473		6.23	[1.44; 26.95]	0.3%	0.7%
Zhang L_WUH	1.04	0.1956		2.82	[1.92; 4.13]	4.5%	2.0%
Hu D_UH	1.10	0.4180		3.01	[1.33; 6.83]	1.0%	1.3%
Xie J_UHW	1.10	0.3867		2.99	[1.40; 6.39]	1.2%	1.4%
Yang A_	2.48	0.5951		12.00	[3.74; 38.52]	0.5%	0.9%
Zheng F_NHCFH	2.09	0.4971		8.07	[3.04; 21.37]	0.7%	1.1%
LiX_TH	0.79	0.1908		2.21	[1.52; 3.21]	4.8%	2.1%
Li Y_TH	0.63	1.4804		1.88	[0.10; 34.13]	0.1%	0.2%
**Fixed effect model**				2.27	[2.09; 2.47]	92.3%	--
**Random effects model**				2.61	[2.25; 3.03]	--	92.8%
Heterogeneity: $I^2 = 56\%$, $\tau^2 = 0.2211$, $p < 0.01$							
**MRB = 1**							
Liang W_MC	0.63	0.2209		1.88	[1.22; 2.90]	3.6%	2.0%
Wang Y_ZH(Multicéntrico)	0.26	0.4403		1.30	[0.55; 3.08]	0.9%	1.3%
Rentsch_CT	0.41	0.3940		1.51	[0.70; 3.27]	1.1%	1.4%
Bi Q_STPH	1.45	0.3327		4.28	[2.23; 8.21]	1.6%	1.6%
Kalligeros M_MC	-0.24	0.5443		0.79	[0.27; 2.30]	0.6%	1.0%
**Fixed effect model**				1.93	[1.44; 2.59]	7.7%	--
**Random effects model**				1.81	[1.10; 2.96]	--	7.2%
Heterogeneity: $I^2 = 58\%$, $\tau^2 = 0.1787$, $p = 0.05$							
**Fixed effect model**				**2.24**	**[2.07; 2.44]**	**100.0%**	**--**
**Random effects model**				**2.54**	**[2.21; 2.92]**	**--**	**100.0%**
Heterogeneity: $I^2 = 56\%$, $\tau^2 = 0.2135$, $p < 0.01$							
Residual heterogeneity: $I^2 = 56\%$, $p < 0.01$							

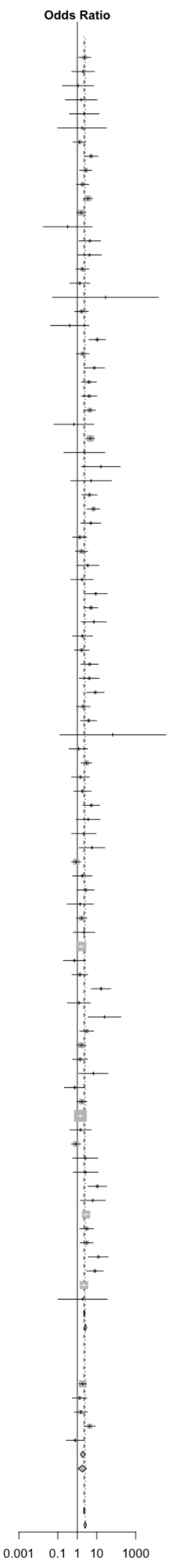

Candidate variable: Obesity, outcome: severe Covid-19 disease, subgroup analysis: (crude vs adjusted)

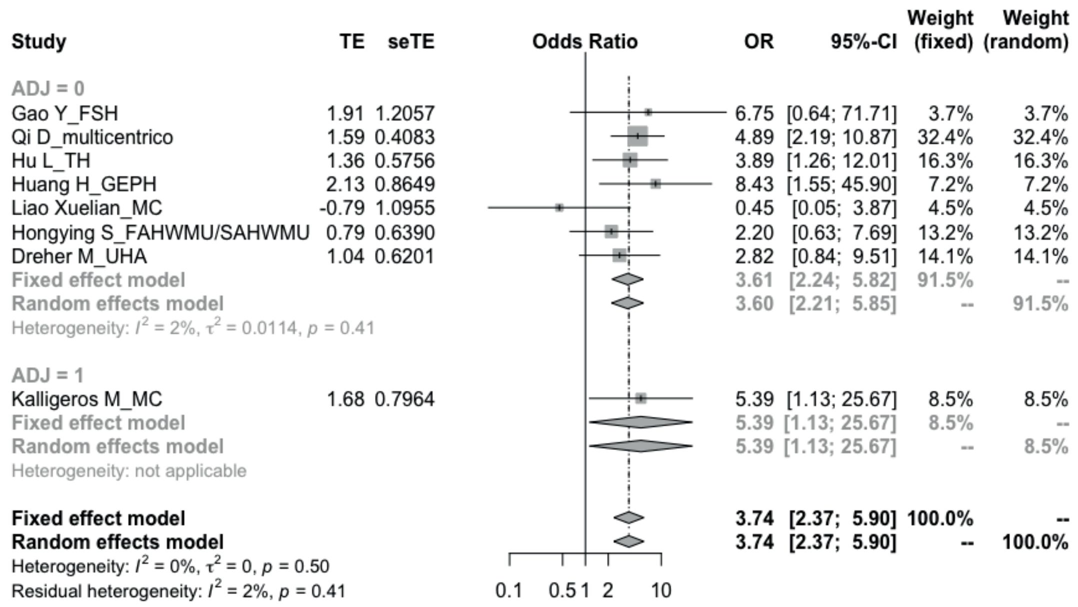

Candidate variable: Chronic kidney disease, outcome: severe Covid-19 disease, subgroup analysis by risk of bias (high vs moderate/low)

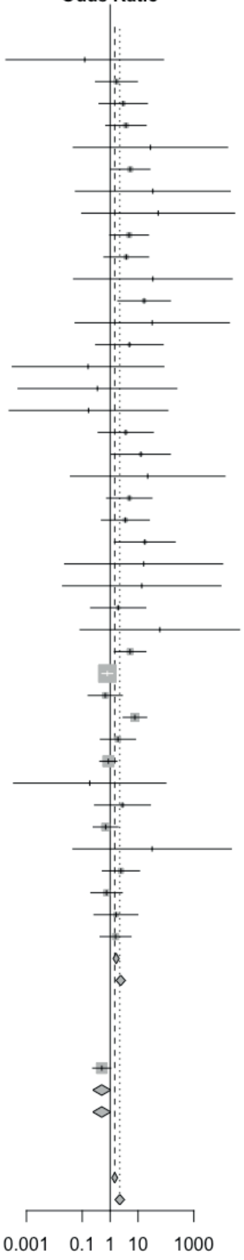

Study	TE	seTE	OR	95%-CI	Weight (fixed)	Weight (random)
MRB = 0						
Liu Y_STP	-2.10	3.3169	0.12	[0.00; 81.77]	0.1%	0.3%
Mo P_ZH	0.52	0.8815	1.68	[0.30; 9.45]	1.8%	3.1%
Wang D_ZH	1.08	1.0195	2.94	[0.40; 21.69]	1.4%	2.5%
Shi Yu_ZPV	1.30	0.8506	3.69	[0.70; 19.52]	2.0%	3.2%
Jin-Jin Z_MC	3.33	3.2490	27.82	[0.05; 16215.51]	0.1%	0.3%
Wei-jie G_NHC	1.66	0.8264	5.26	[1.04; 26.58]	2.1%	3.3%
Chen Y_ multicentrico- FCMCH	3.52	3.2591	33.85	[0.06; 20123.04]	0.1%	0.3%
Duan Q_WPH	3.96	3.2204	52.68	[0.10; 29034.41]	0.1%	0.4%
Liu Y_ CHW	1.57	0.8159	4.80	[0.97; 23.75]	2.1%	3.4%
Sun F_ZHWU	1.33	0.9393	3.78	[0.60; 23.81]	1.6%	2.8%
Shijiao Y_HHMU	3.52	3.3387	33.94	[0.05; 23585.56]	0.1%	0.3%
Zhang G_ZHWU	2.80	1.1073	16.50	[1.88; 144.54]	1.2%	2.3%
Zhao W_SXH	3.47	3.2501	32.26	[0.06; 18839.23]	0.1%	0.3%
Chen X_FHC/LCH	1.59	1.4228	4.90	[0.30; 79.64]	0.7%	1.5%
Hu L_TH	-1.82	3.1916	0.16	[0.00; 84.20]	0.1%	0.4%
Ma K_YCH	-1.05	3.3431	0.35	[0.00; 245.33]	0.1%	0.3%
Qin X_SPH	-1.78	3.3404	0.17	[0.00; 117.30]	0.1%	0.3%
Wang Y_ZH(Multicéntrico)	1.29	1.1637	3.62	[0.37; 35.38]	1.0%	2.1%
Yang L_YCPH	2.53	1.2421	12.59	[1.10; 143.68]	0.9%	1.9%
Zhang H_ZH	3.10	3.2539	22.17	[0.04; 13044.30]	0.1%	0.3%
Zhao W_BYH	1.58	0.9541	4.85	[0.75; 31.49]	1.6%	2.8%
Zeng G_TPHS	1.26	1.0086	3.51	[0.49; 25.37]	1.4%	2.6%
Liao Xuelian_MC	2.86	1.2803	17.50	[1.42; 215.20]	0.9%	1.8%
Chao C_NFHJCH	2.77	3.3277	15.91	[0.02; 10819.14]	0.1%	0.3%
Lei S_RHZHTHC	2.61	3.3352	13.57	[0.02; 9366.23]	0.1%	0.3%
FY_JH, SPHCC, TPH	0.67	1.1620	1.95	[0.20; 18.99]	1.0%	2.1%
JX_WFPH	4.10	3.3578	60.43	[0.08; 43592.13]	0.1%	0.3%
Colombi D_GdSH	1.65	0.6640	5.20	[1.42; 19.11]	3.2%	4.2%
Argenziano M_NYP/CUIMC	-0.23	0.2292	0.79	[0.51; 1.24]	26.9%	7.4%
Dreher M_UHA	-0.41	0.7188	0.67	[0.16; 2.73]	2.7%	3.9%
Li J_CHW	2.04	0.4957	7.72	[2.92; 20.40]	5.7%	5.4%
Zhou M_MC	0.65	0.7402	1.91	[0.45; 8.15]	2.6%	3.8%
YuC_TH	-0.15	0.3628	0.86	[0.42; 1.76]	10.7%	6.4%
WangL_RH	-1.68	3.2101	0.19	[0.00; 100.19]	0.1%	0.4%
Yan X_HNU	1.02	1.1756	2.77	[0.28; 27.72]	1.0%	2.1%
Zhang L_WUH	-0.37	0.5212	0.69	[0.25; 1.91]	5.2%	5.2%
Bi Q_STPH	3.47	3.3354	31.99	[0.05; 22083.43]	0.1%	0.3%
Xie J_UHW	0.89	0.7850	2.44	[0.52; 11.36]	2.3%	3.5%
Kalligeros M_MC	-0.30	0.6611	0.74	[0.20; 2.71]	3.2%	4.2%
Wu J_TFAH	0.48	0.9224	1.61	[0.26; 9.84]	1.7%	2.9%
LiX_TH	0.45	0.6512	1.56	[0.44; 5.60]	3.3%	4.3%
Fixed effect model			1.64	[1.29; 2.10]	90.2%	--
Random effects model			2.39	[1.63; 3.49]	--	93.7%
Heterogeneity: $I^2 = 35\%$, $\tau^2 = 0.3842$, $p = 0.02$						
MRB = 1						
Rentsch_CT	-0.69	0.3789	0.50	[0.24; 1.05]	9.8%	6.3%
Fixed effect model			0.50	[0.24; 1.05]	9.8%	--
Random effects model			0.50	[0.24; 1.05]	--	6.3%
Heterogeneity: not applicable						
**Fixed effect model**			**1.46**	**[1.16; 1.84]**	**100.0%**	**--**
**Random effects model**			**2.21**	**[1.51; 3.24]**	**--**	**100.0%**

Heterogeneity: $I^2 = 42\%$, $\tau^2 = 0.4663$, $p < 0.01$
Residual heterogeneity: $I^2 = 35\%$, $p = 0.02$

Candidate variable: Asthma, outcome: severe Covid-19 disease

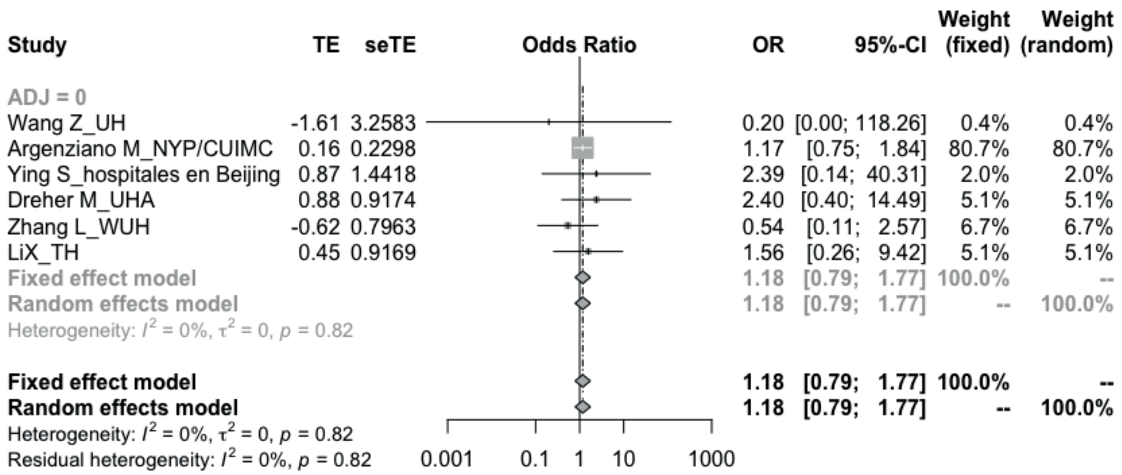

Candidate variable: COPD, outcome: severe Covid-19 disease, subgroup analysis by risk of bias (high vs moderate/low)

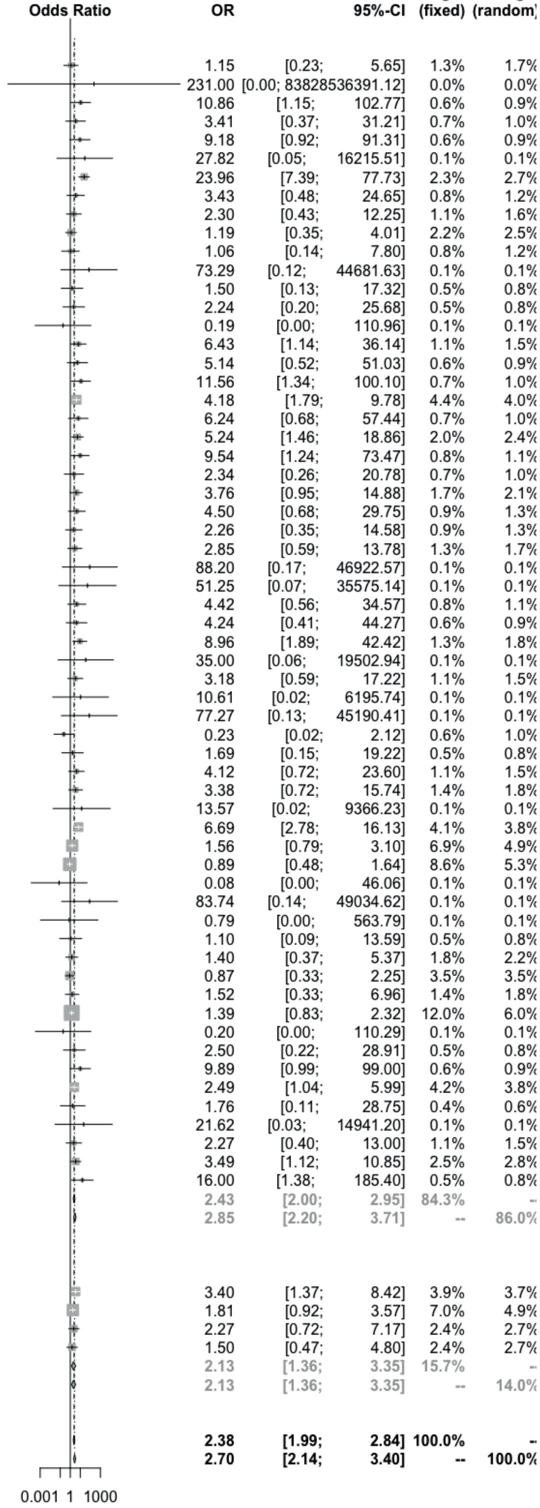

Study	TE	seTE	OR	95%-CI	Weight (fixed)	Weight (random)
**MRB = 0**						
Gao Y_FSH	0.14	0.8125	1.15	[0.23; 5.65]	1.3%	1.7%
Huang C_JYH	5.44	10.0561	231.00	[0.00; 83828536391.12]	0.0%	0.0%
Li K_CMU	2.38	1.1468	10.86	[1.15; 102.77]	0.6%	0.9%
Mo P_ZH	1.23	1.1300	3.41	[0.37; 31.21]	0.7%	1.0%
Wang D_ZH	2.22	1.1720	9.18	[0.92; 91.31]	0.6%	0.9%
Jin-Jin Z_MC	3.33	3.2490	27.82	[0.05; 16215.51]	0.1%	0.1%
Wei-jie G_NHC	3.18	0.6004	23.96	[7.39; 77.73]	2.3%	2.7%
Lu Jiatao_WHH	1.23	1.0066	3.43	[0.48; 24.65]	0.8%	1.2%
Chen Y_ multicentrico- FCMCH	0.83	0.8526	2.30	[0.43; 12.25]	1.1%	1.6%
Duan Q_WPH	0.17	0.6196	1.19	[0.35; 4.01]	2.2%	2.5%
Liu Y_ CHW	0.06	1.0189	1.06	[0.14; 7.80]	0.8%	1.2%
Liu Yo_SCH	4.29	3.2720	73.29	[0.12; 44681.63]	0.1%	0.1%
Song CY_FAHZU	0.41	1.2483	1.50	[0.13; 17.32]	0.5%	0.8%
Sun F_ZHWU	0.81	1.2445	2.24	[0.20; 25.68]	0.5%	0.8%
Shijiao Y_HHMU	-1.66	3.2498	0.19	[0.00; 110.96]	0.1%	0.1%
Zhang G_ZHWU	1.86	0.8807	6.43	[1.14; 36.14]	1.1%	1.5%
Zhao W_SXH	1.64	1.1709	5.14	[0.52; 51.03]	0.6%	0.9%
Zhou Y_CHW	2.45	1.1012	11.56	[1.34; 100.10]	0.7%	1.0%
Zhen L_MC	1.43	0.4337	4.18	[1.79; 9.78]	4.4%	4.0%
Bai X_WPH	1.83	1.1326	6.24	[0.68; 57.44]	0.7%	1.0%
Chen X_FHC/LCH	1.66	0.6530	5.24	[1.46; 18.86]	2.0%	2.4%
Feng Z_TXH	2.26	1.0416	9.54	[1.24; 73.47]	0.8%	1.1%
Hu L_TH	0.85	1.1151	2.34	[0.26; 20.78]	0.7%	1.0%
Li J_CHW	1.33	0.7013	3.76	[0.95; 14.88]	1.7%	2.1%
Liu J_BDH	1.50	0.9636	4.50	[0.68; 29.75]	0.9%	1.3%
Ma K_YCH	0.82	0.9514	2.26	[0.35; 14.58]	0.9%	1.3%
Lei P_BH (Multicéntrico)	1.05	0.8040	2.85	[0.59; 13.78]	1.3%	1.7%
Qin X_SPH	4.48	3.2024	88.20	[0.17; 46922.57]	0.1%	0.1%
Wang G_PHTCC	3.94	3.3382	51.25	[0.07; 35575.14]	0.1%	0.1%
Wang Z_UH	1.49	1.0498	4.42	[0.56; 34.57]	0.8%	1.1%
Xu Y_FAHG	1.44	1.1974	4.24	[0.41; 44.27]	0.6%	0.9%
Yang L_YCPH	2.19	0.7933	8.96	[1.89; 42.42]	1.3%	1.8%
Zhang H_ZH	3.56	3.2261	35.00	[0.06; 19502.94]	0.1%	0.1%
Zhao W_BYH	1.16	0.8625	3.18	[0.59; 17.22]	1.1%	1.5%
Jiancheng L_JH	2.36	3.2499	10.61	[0.02; 6195.74]	0.1%	0.1%
Mingfeng H_SPH	4.35	3.2508	77.27	[0.13; 45190.41]	0.1%	0.1%
Wentao X_IDH	-1.48	1.1397	0.23	[0.02; 2.12]	0.6%	1.0%
Kuang Y_MC	0.52	1.2411	1.69	[0.15; 19.22]	0.5%	0.8%
Wang Y_CHW	1.42	0.8909	4.12	[0.72; 23.60]	1.1%	1.5%
Liao Xuelian_MC	1.22	0.7855	3.38	[0.72; 15.74]	1.4%	1.8%
Lei S_RHZHTHC	2.61	3.3352	13.57	[0.02; 9366.23]	0.1%	0.1%
FY_JH, SPHCC, TPH	1.90	0.4487	6.69	[2.78; 16.13]	4.1%	3.8%
Colombi D_GdSH	0.45	0.3489	1.56	[0.79; 3.10]	6.9%	4.9%
Argenziano M_NYP/CUIMC	-0.12	0.3109	0.89	[0.48; 1.64]	8.6%	5.3%
Colaneri M_PSM	-2.56	3.2596	0.08	[0.00; 46.06]	0.1%	0.1%
Hongying S_FAHWMU/SAHWMU	4.43	3.2514	83.74	[0.14; 49034.62]	0.1%	0.1%
Chen W_YH	-0.24	3.3522	0.79	[0.00; 563.79]	0.1%	0.1%
Herold T_UH	0.10	1.2827	1.10	[0.09; 13.59]	0.5%	0.8%
Dreher M_UHA	0.34	0.6854	1.40	[0.37; 5.37]	1.8%	2.2%
Li J_CHW	-0.14	0.4865	0.87	[0.33; 2.25]	3.5%	3.5%
Zhou M_MC	0.42	0.7763	1.52	[0.33; 6.96]	1.4%	1.8%
YuC_TH	0.33	0.2637	1.39	[0.83; 2.32]	12.0%	6.0%
Wang L_SPH	-1.63	3.2303	0.20	[0.00; 110.29]	0.1%	0.1%
Wan S_TGCH	0.92	1.2490	2.50	[0.22; 28.91]	0.5%	0.8%
Zhang R_RH	2.29	1.1754	9.89	[0.99; 99.00]	0.6%	0.9%
Zhang L_WUH	0.91	0.4477	2.49	[1.04; 5.99]	4.2%	3.8%
Xie J_UHW	0.57	1.4252	1.76	[0.11; 28.75]	0.4%	0.6%
Wu J_TFAH	3.07	3.3359	21.62	[0.03; 14941.20]	0.1%	0.1%
Zheng F_NHCFH	0.82	0.8908	2.27	[0.40; 13.00]	1.1%	1.5%
LiX_TH	1.25	0.5783	3.49	[1.12; 10.85]	2.5%	2.8%
Li Y_TH	2.77	1.2500	16.00	[1.38; 185.40]	0.5%	0.8%
Fixed effect model			2.43	[2.00; 2.95]	84.3%	--
Random effects model			2.85	[2.20; 3.71]	--	86.0%
Heterogeneity: I^2 = 26%, τ^2 = 0.2185, p = 0.04						
**MRB = 1**						
Liang W_MC	1.22	0.4629	3.40	[1.37; 8.42]	3.9%	3.7%
Rentsch_CT	0.59	0.3466	1.81	[0.92; 3.57]	7.0%	4.9%
Bi Q_STPH	0.82	0.5860	2.27	[0.72; 7.17]	2.4%	2.7%
Kalligeros M_MC	0.41	0.5938	1.50	[0.47; 4.80]	2.4%	2.7%
Fixed effect model			2.13	[1.36; 3.35]	15.7%	--
Random effects model			2.13	[1.36; 3.35]	--	14.0%
Heterogeneity: I^2 = 0%, τ^2 = 0, p = 0.66						
**Fixed effect model**			**2.38**	**[1.99; 2.84]**	**100.0%**	**--**
**Random effects model**			**2.70**	**[2.14; 3.40]**	**--**	**100.0%**
Heterogeneity: I^2 = 23%, τ^2 = 0.1641, p = 0.06						
Residual heterogeneity: I^2 = 24%, p = 0.05						

Candidate variable: Tuberculosis, outcome: severe Covid-19 disease

Study	TE	seTE	OR	95%-CI	Weight (fixed)	Weight (random)
ADJ = 0						
Mo P_ZH	3.21	3.2204	24.76	[0.04; 13642.22]	1.8%	1.8%
Jin-Jin Z_MC	3.33	3.2490	27.82	[0.05; 16215.51]	1.7%	1.7%
Zhang H_CPHMC	-1.50	3.3493	0.22	[0.00; 157.66]	1.6%	1.6%
Bai X_WPH	0.41	0.7303	1.50	[0.36; 6.28]	34.1%	34.1%
Zhang H_ZH	-3.37	3.2260	0.03	[0.00; 19.21]	1.7%	1.7%
CM_FAHSYU	4.03	3.2529	56.21	[0.10; 33015.36]	1.7%	1.7%
Ying S_hospitales en Beijing	-1.38	3.3446	0.25	[0.00; 176.78]	1.6%	1.6%
Bi Q_STPH	-0.85	1.0672	0.43	[0.05; 3.46]	16.0%	16.0%
LiX_TH	-0.19	0.6763	0.83	[0.22; 3.11]	39.8%	39.8%
Fixed effect model			1.00	[0.44; 2.32]	100.0%	--
Random effects model			1.00	[0.44; 2.32]	--	100.0%
Heterogeneity: $I^2 = 0\%$, $\tau^2 = 0$, $p = 0.64$						
**Fixed effect model**			**1.00**	**[0.44; 2.32]**	**100.0%**	**--**
**Random effects model**			**1.00**	**[0.44; 2.32]**	**--**	**100.0%**
Heterogeneity: $I^2 = 0\%$, $\tau^2 = 0$, $p = 0.64$						
Residual heterogeneity: $I^2 = 0\%$, $p = 0.64$						

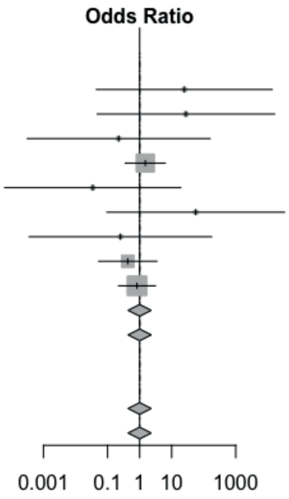

Candidate variable: Cancer (solid or active haematologic cancer), outcome: severe Covid-19 disease, subgroup analysis by risk of bias (high vs moderate/low)

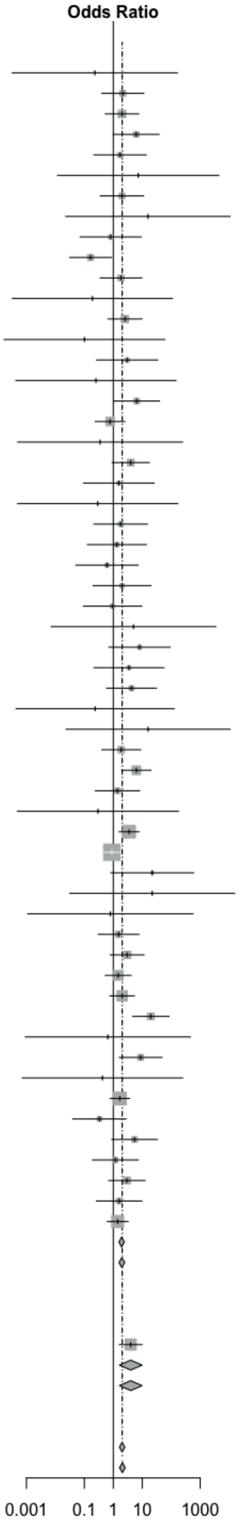

Study	TE	seTE	OR	95%-CI	Weight (fixed)	Weight (random)
**MRB = 0**						
Huang C_JYH	-1.47	3.3503	0.23	[0.00; 164.05]	0.1%	0.1%
Mo P_ZH	0.75	0.8527	2.12	[0.40; 11.30]	1.6%	1.7%
Wang D_ZH	0.69	0.6770	2.00	[0.53; 7.54]	2.5%	2.7%
Shi Yu_ZPV	1.82	0.9257	6.17	[1.01; 37.86]	1.3%	1.5%
Wei-jie G_NHC	0.54	1.0617	1.72	[0.21; 13.80]	1.0%	1.1%
Qi Xiaolong_MC	1.98	3.2709	7.24	[0.01; 4403.39]	0.1%	0.1%
Chen X_GHCTC	0.69	0.8796	2.00	[0.36; 11.21]	1.5%	1.6%
Chen Y_ multicentrico- FCMCH	2.75	3.3341	15.71	[0.02; 10822.26]	0.1%	0.1%
Duan Q_WPH	-0.21	1.2400	0.81	[0.07; 9.25]	0.8%	0.8%
Liu T_ UH	-1.81	0.8507	0.16	[0.03; 0.87]	1.6%	1.7%
Sun F_ZHWU	0.61	0.8461	1.85	[0.35; 9.70]	1.6%	1.8%
Shijiao Y_HHMU	-1.66	3.2498	0.19	[0.00; 110.96]	0.1%	0.1%
Zhang G_ZHWU	0.93	0.6898	2.53	[0.65; 9.76]	2.4%	2.6%
Jing L_WUH	-2.29	3.2623	0.10	[0.00; 60.55]	0.1%	0.1%
Bai X_WPH	1.10	1.2377	3.00	[0.27; 33.94]	0.8%	0.8%
Chen X_FHC/LCH	-1.38	3.2481	0.25	[0.00; 146.37]	0.1%	0.1%
Hu L_TH	1.86	0.9241	6.45	[1.05; 39.46]	1.4%	1.5%
Li J_CHW	-0.25	0.5987	0.78	[0.24; 2.51]	3.2%	3.3%
Ma K_YCH	-1.05	3.3431	0.35	[0.00; 245.33]	0.1%	0.1%
Lei P_BH (Multicéntrico)	1.39	0.7522	4.00	[0.92; 17.47]	2.0%	2.2%
Qin X_SPH	0.44	1.4312	1.56	[0.09; 25.76]	0.6%	0.6%
Wang G_PHTCC	-1.24	3.2493	0.29	[0.00; 168.40]	0.1%	0.1%
Wang Y_ZH(Multicéntrico)	0.59	1.0898	1.80	[0.21; 15.22]	1.0%	1.1%
Wang Z_UH	0.29	1.1956	1.33	[0.13; 13.89]	0.8%	0.9%
Xu Y_FAHG	-0.50	1.2633	0.61	[0.05; 7.20]	0.7%	0.8%
Yang L_YCPH	0.69	1.1726	2.00	[0.20; 19.91]	0.8%	0.9%
Zhao W_BYH	-0.05	1.1851	0.95	[0.09; 9.67]	0.8%	0.9%
Jiancheng L_JH	1.60	3.3387	4.96	[0.01; 3447.51]	0.1%	0.1%
Mingfeng H_SPH	2.09	1.2416	8.07	[0.71; 91.95]	0.7%	0.8%
Zeng G_TPHS	1.25	1.4202	3.48	[0.22; 56.30]	0.6%	0.6%
CaiQ_TPHS	1.45	1.0110	4.25	[0.59; 30.83]	1.1%	1.3%
Cao M_SPHCC	-1.44	3.2116	0.24	[0.00; 127.92]	0.1%	0.1%
Chao C_NFHJCH	2.77	3.3277	15.91	[0.02; 10819.14]	0.1%	0.1%
Lei S_RHZHTHC	0.63	0.7853	1.88	[0.40; 8.74]	1.9%	2.0%
FY_JH, SPHCC, TPH	1.83	0.5928	6.25	[1.96; 19.98]	3.3%	3.4%
CM_FAHSYU	0.33	0.8981	1.40	[0.24; 8.11]	1.4%	1.6%
JX_WFPH	-1.22	3.2670	0.30	[0.00; 178.74]	0.1%	0.1%
Colombi D_GdSH	1.25	0.4008	3.50	[1.60; 7.68]	7.2%	6.6%
Argenziano M_NYP/CUIMC	-0.12	0.3109	0.89	[0.48; 1.64]	12.0%	9.6%
Colaneri M_PSM	3.10	1.6790	22.20	[0.83; 596.34]	0.4%	0.5%
Ying S_hospitales en Beijing	3.09	3.3450	22.00	[0.03; 15476.99]	0.1%	0.1%
Chen W_YH	-0.24	3.3522	0.79	[0.00; 563.79]	0.1%	0.1%
Dreher M_UHA	0.43	0.8227	1.53	[0.31; 7.69]	1.7%	1.9%
Li J_CHW	1.10	0.6854	3.01	[0.78; 11.52]	2.5%	2.6%
Zhou M_MC	0.39	0.5231	1.48	[0.53; 4.13]	4.2%	4.2%
YuC_TH	0.70	0.4960	2.02	[0.76; 5.34]	4.7%	4.6%
WangL_RH	2.99	0.7372	19.80	[4.67; 83.97]	2.1%	2.3%
Wang L_SPH	-0.43	3.3468	0.65	[0.00; 458.56]	0.1%	0.1%
Zhang R_RH	2.17	0.8668	8.80	[1.61; 48.12]	1.5%	1.7%
Yan X_HNU	-0.86	3.2516	0.42	[0.00; 246.66]	0.1%	0.1%
Zhang L_WUH	0.52	0.3943	1.68	[0.78; 3.64]	7.4%	6.7%
Hu D_UH	-1.10	1.0820	0.33	[0.04; 2.78]	1.0%	1.1%
Bi Q_STPH	1.69	0.9206	5.42	[0.89; 32.91]	1.4%	1.5%
Xie J_UHW	0.16	0.9302	1.17	[0.19; 7.24]	1.3%	1.5%
Kalligeros M_MC	1.08	0.7377	2.95	[0.69; 12.51]	2.1%	2.3%
Wu J_TFAH	0.47	0.9224	1.60	[0.26; 9.74]	1.4%	1.5%
LiX_TH	0.35	0.4238	1.42	[0.62; 3.25]	6.4%	6.0%
**Fixed effect model**			1.94	[1.56; 2.41]	94.6%	--
**Random effects model**			1.97	[1.57; 2.48]	--	94.8%
Heterogeneity: $I^2 = 4\%$, $\tau^2 = 0.0283$, $p = 0.39$						
**MRB = 1**						
Liang W_MC	1.38	0.4628	3.99	[1.61; 9.88]	5.4%	5.2%
**Fixed effect model**			3.99	[1.61; 9.88]	5.4%	--
**Random effects model**			3.99	[1.61; 9.88]	--	5.2%
Heterogeneity: not applicable						
**Fixed effect model**			**2.02**	**[1.63; 2.49]**	**100.0%**	**--**
**Random effects model**			**2.06**	**[1.64; 2.58]**	**--**	**100.0%**
Heterogeneity: $I^2 = 6\%$, $\tau^2 = 0.0424$, $p = 0.35$						
Residual heterogeneity: $I^2 = 4\%$, $p = 0.39$						

Candidate variable: HIV infection, outcome: severe Covid-19 disease

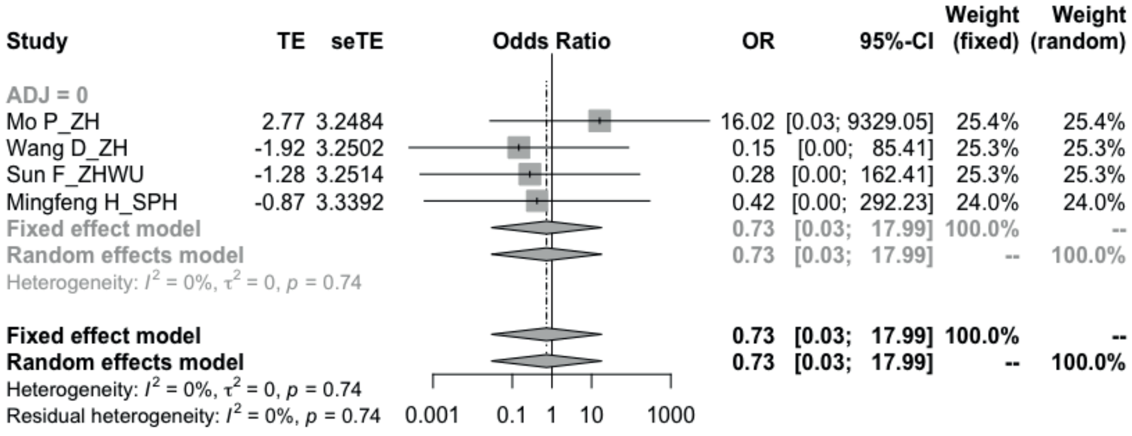

Candidate variable: Immunocompromised, outcome: severe Covid-19 disease

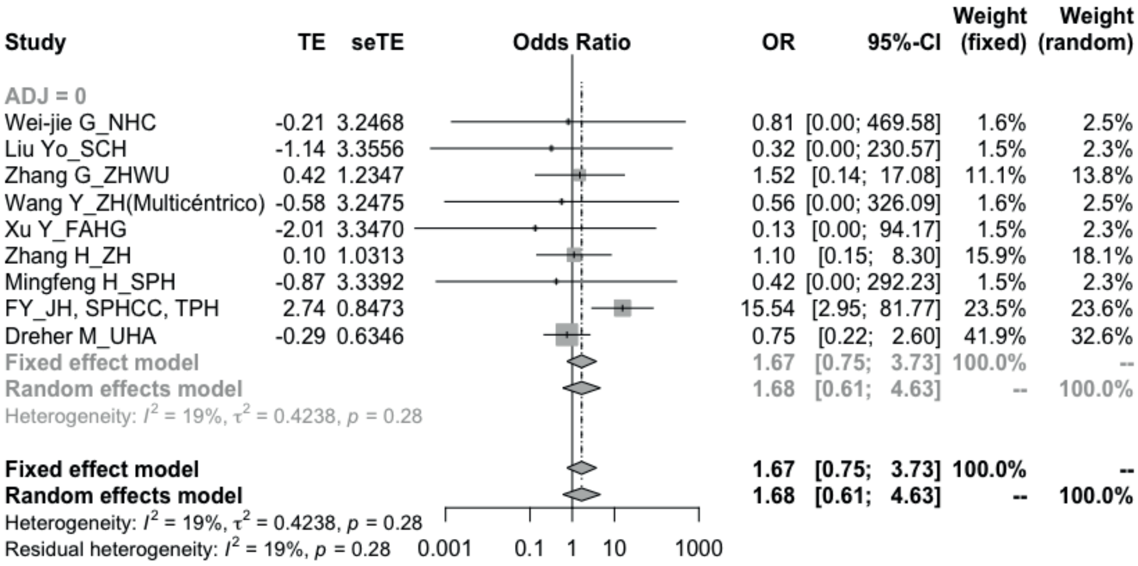

Candidate variable: Autoimmune disease, outcome: severe Covid-19 disease

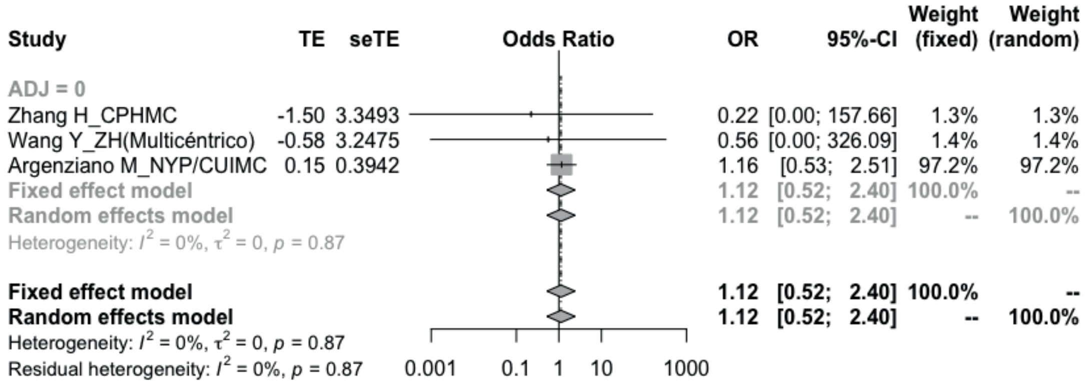

Candidate variable: Chronic liver disease, outcome: severe Covid-19 disease

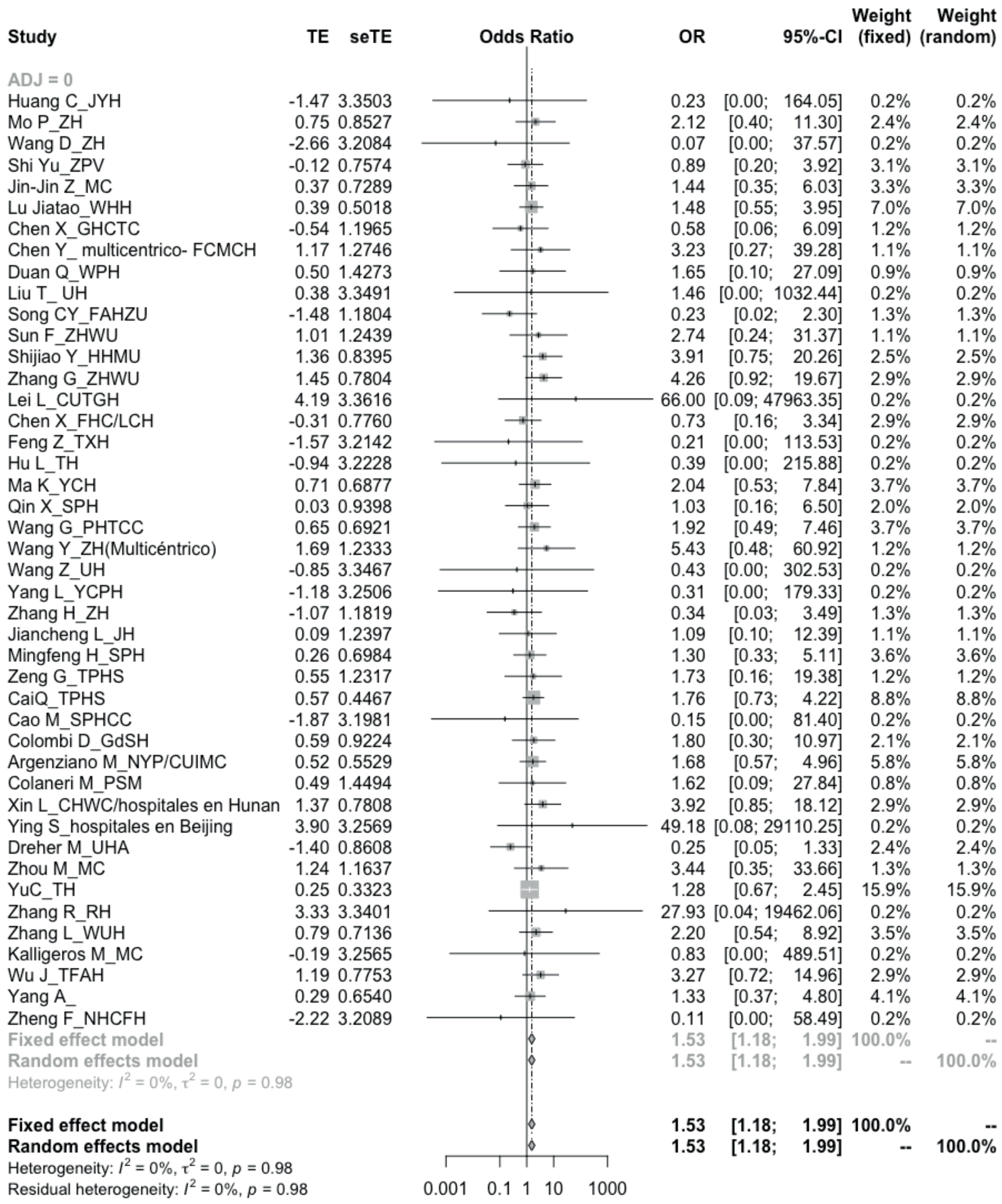

Candidate variable: Thyroid disease, outcome: severe Covid-19 disease

Study	TE	seTE	OR	95%-CI	Weight (fixed)	Weight (random)
ADJ = 0						
Jin-Jin Z_MC	1.79	1.1317	6.00	[0.65; 55.14]	25.5%	25.5%
Chen X_GHCTC	0.66	1.0493	1.93	[0.25; 15.12]	29.6%	29.6%
Shijiao Y_HHMU	1.32	1.4269	3.74	[0.23; 61.35]	16.0%	16.0%
Jing L_WUH	-2.29	3.2623	0.10	[0.00; 60.55]	3.1%	3.1%
Cao M_SPHCC	0.66	1.1231	1.93	[0.21; 17.47]	25.9%	25.9%
Fixed effect model			2.62	[0.86; 8.02]	100.0%	--
Random effects model			2.62	[0.86; 8.02]	--	100.0%
Heterogeneity: $I^2 = 0\%$, $\tau^2 = 0$, $p = 0.78$						
**Fixed effect model**			**2.62**	**[0.86; 8.02]**	**100.0%**	**--**
**Random effects model**			**2.62**	**[0.86; 8.02]**	**--**	**100.0%**

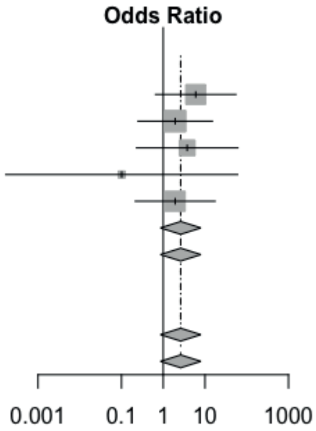

Heterogeneity: $I^2 = 0\%$, $\tau^2 = 0$, $p = 0.78$
Residual heterogeneity: $I^2 = 0\%$, $p = 0.78$

Candidate variable: Chronic gastric disease: History of peptic ulcer or gastritis, outcome: severe Covid-19 disease

Study	TE	seTE	OR	95%-CI	Weight (fixed)	Weight (random)
ADJ = 0						
Jin-Jin Z_MC	-0.60	0.8549	0.55	[0.10; 2.94]	4.2%	4.2%
Zhao W_SXH	1.64	1.1709	5.14	[0.52; 51.03]	2.2%	2.2%
Zhen L_MC	0.88	0.4875	2.40	[0.92; 6.25]	12.8%	12.8%
Bai X_WPH	0.21	0.4888	1.24	[0.47; 3.22]	12.7%	12.7%
Hu L_TH	1.04	0.5955	2.82	[0.88; 9.05]	8.6%	8.6%
Li J_CHW	0.76	0.4945	2.14	[0.81; 5.64]	12.5%	12.5%
Lei P_BH (Multicéntrico)	-2.47	3.2113	0.08	[0.00; 45.87]	0.3%	0.3%
Chen W_YH	-0.24	3.3522	0.79	[0.00; 563.79]	0.3%	0.3%
Li J_CHW	0.24	0.2561	1.28	[0.77; 2.11]	46.4%	46.4%
Fixed effect model			1.55	[1.10; 2.19]	100.0%	--
Random effects model			1.55	[1.10; 2.19]	--	100.0%
Heterogeneity: $I^2 = 0\%$, $\tau^2 = 0$, $p = 0.60$						
**Fixed effect model**			**1.55**	**[1.10; 2.19]**	**100.0%**	**--**
**Random effects model**			**1.55**	**[1.10; 2.19]**	**--**	**100.0%**

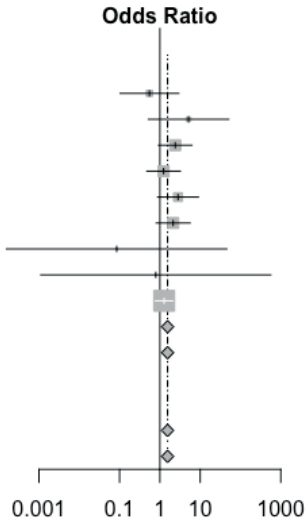

Heterogeneity: $I^2 = 0\%$, $\tau^2 = 0$, $p = 0.60$
Residual heterogeneity: $I^2 = 0\%$, $p = 0.60$

Candidate variable: Dyslipidemia, outcome: severe Covid-19 disease

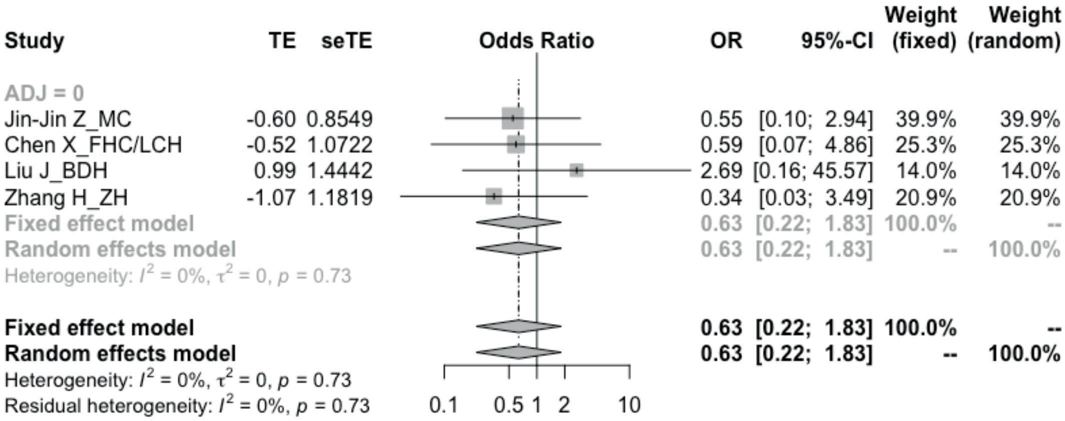

Candidate variable: Any chronic condition or comorbidities, outcome: severe Covid-19 disease, subgroup analysis by risk of bias (high vs moderate/low)

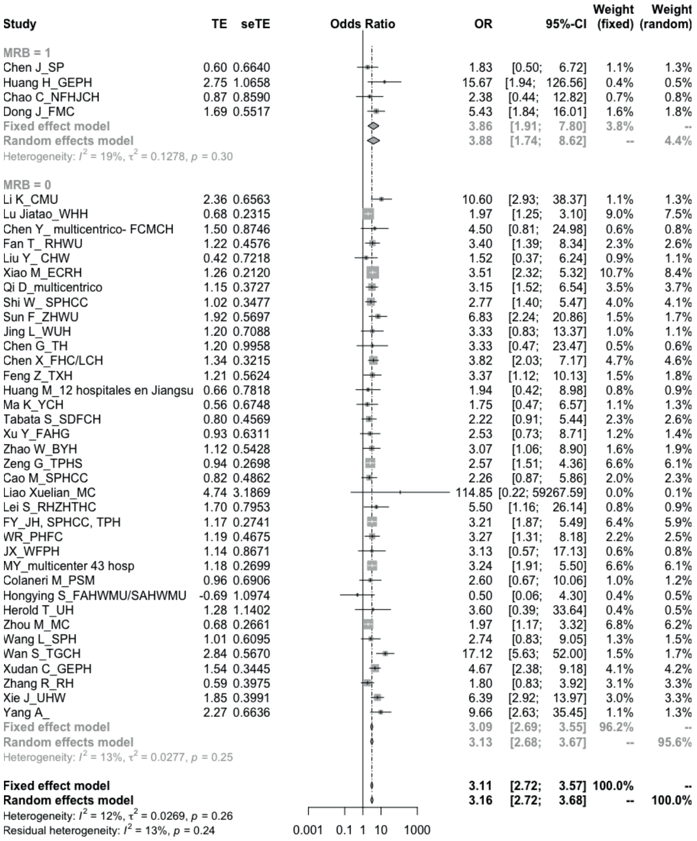

Candidate variable: Respiratory failure, outcome: severe Covid-19 disease, subgroup analysis by risk of bias (high vs moderate/low)

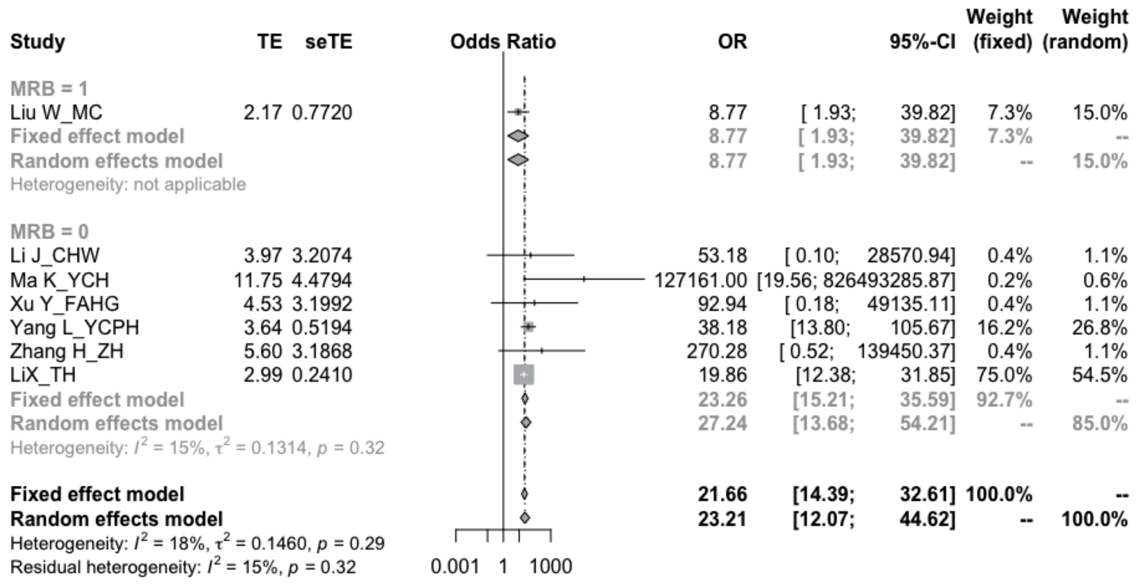

Candidate variable: Tachypnea, outcome: severe Covid-19 disease, subgroup analysis by risk of bias (high vs moderate/low)

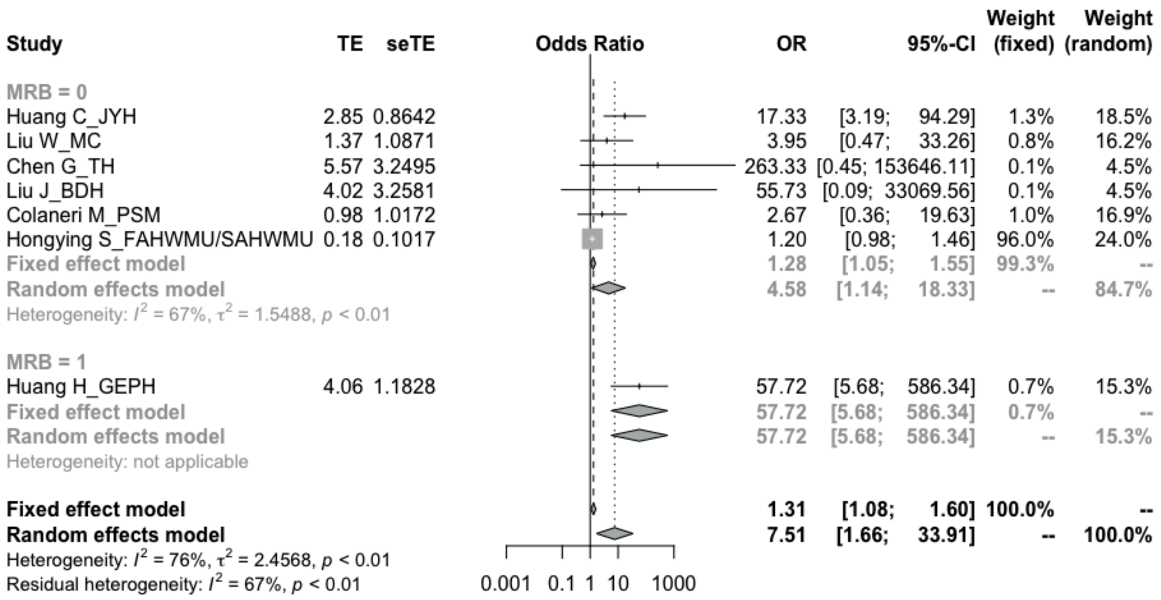

Candidate variable: Hypoxemia, outcome: severe Covid-19 disease

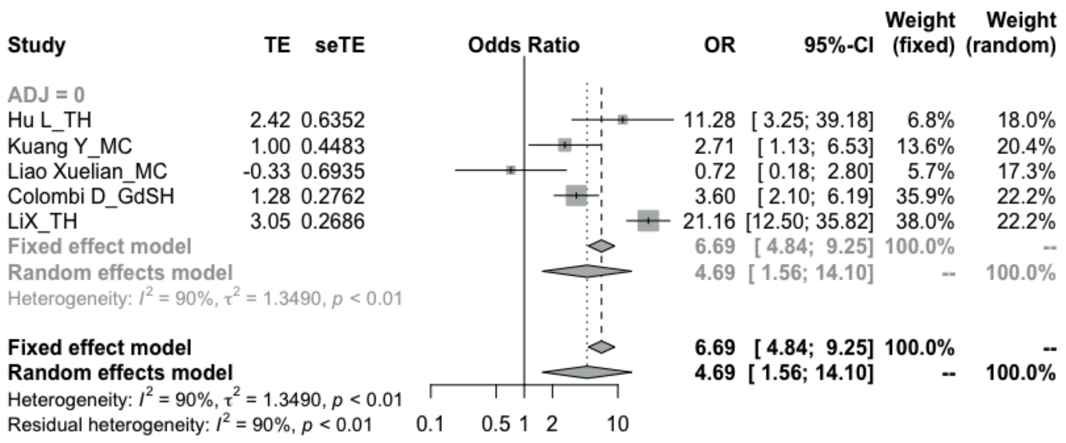

Candidate variable: Dyspnea, outcome: severe Covid-19 disease, subgroup analysis by risk of bias (high vs moderate/low)

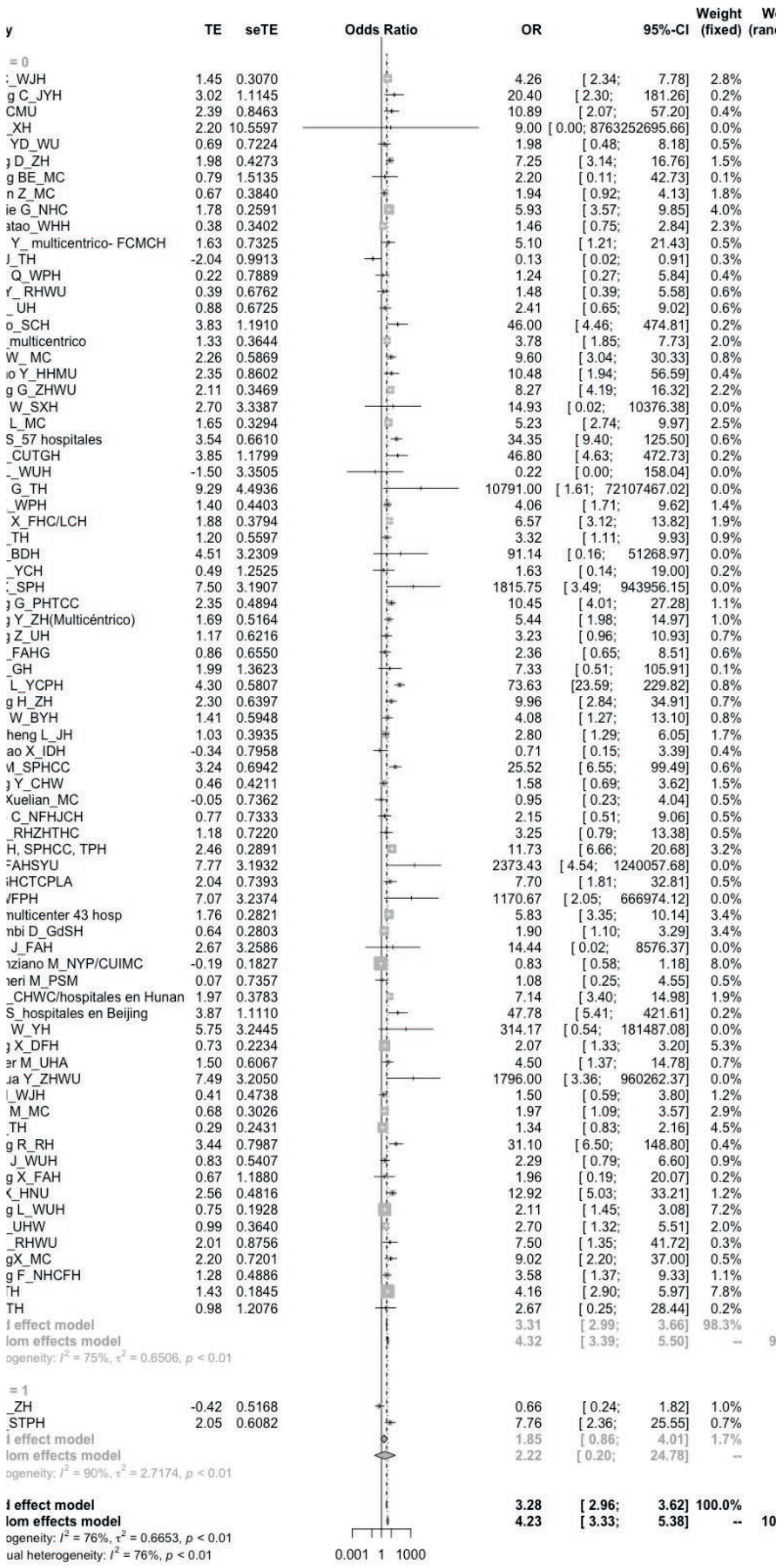

Candidate variable: Chest pain, outcome: severe Covid-19 disease

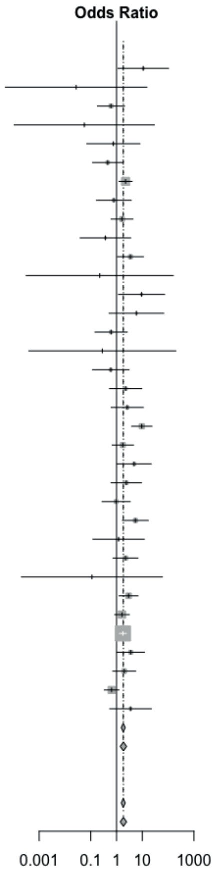

Study	TE	seTE	OR	95%-CI	Weight (fixed)	Weight (random)
ADJ = 0						
Li K_CMU	2.38	1.1468	10.86	[1.15; 102.77]	0.6%	1.3%
Mo P_ZH	-3.61	3.2204	0.03	[0.00; 14.98]	0.1%	0.2%
Peng YD_WU	-0.50	0.6155	0.61	[0.18; 2.03]	2.0%	3.3%
Lu Jiatao_WHH	-2.88	3.1909	0.06	[0.00; 29.27]	0.1%	0.2%
Chu J_TH	-0.29	1.2076	0.75	[0.07; 8.00]	0.5%	1.2%
Duan Q_WPH	-0.79	0.6884	0.45	[0.12; 1.75]	1.6%	2.9%
Fan T_ RHWU	0.80	0.2945	2.23	[1.25; 3.98]	8.9%	6.4%
Liu Y_ CHW	-0.25	0.7890	0.78	[0.17; 3.66]	1.2%	2.4%
Shijiao Y_HHMU	0.49	0.4948	1.63	[0.62; 4.31]	3.1%	4.3%
Zhang H_CPHMC	-1.00	1.1483	0.37	[0.04; 3.51]	0.6%	1.3%
Zhen L_MC	1.24	0.5922	3.45	[1.08; 11.02]	2.2%	3.5%
Jing L_WUH	-1.50	3.3505	0.22	[0.00; 158.04]	0.1%	0.2%
Chen G_TH	2.23	1.0494	9.33	[1.19; 72.99]	0.7%	1.5%
Hu L_TH	1.77	1.2423	5.90	[0.52; 67.35]	0.5%	1.1%
Li J_CHW	-0.48	0.7296	0.62	[0.15; 2.57]	1.4%	2.6%
Liu J_BDH	-1.27	3.3456	0.28	[0.00; 197.94]	0.1%	0.2%
Wang Z_UH	-0.52	0.8306	0.60	[0.12; 3.04]	1.1%	2.2%
Xu Y_FAHG	0.81	0.7319	2.25	[0.54; 9.44]	1.4%	2.6%
Zhao W_BYH	0.96	0.7292	2.60	[0.62; 10.86]	1.4%	2.6%
Mingfeng H_SPH	2.25	0.4600	9.50	[3.86; 23.41]	3.6%	4.6%
Kuang Y_MC	0.56	0.4843	1.75	[0.68; 4.52]	3.3%	4.4%
Zeng G_TPHS	1.57	0.7753	4.80	[1.05; 21.92]	1.3%	2.4%
Cao M_SPHCC	0.87	0.6919	2.39	[0.62; 9.29]	1.6%	2.8%
FY_JH, SPHCC, TPH	-0.04	0.6375	0.97	[0.28; 3.37]	1.9%	3.2%
FL_GHCTCPLA	1.68	0.5844	5.39	[1.71; 16.94]	2.3%	3.5%
JX_WFPH	0.18	1.1690	1.20	[0.12; 11.86]	0.6%	1.3%
Xin L_CHWC/hospitales en Hunan	0.80	0.5648	2.22	[0.73; 6.71]	2.4%	3.7%
Chen W_YH	-2.20	3.2089	0.11	[0.00; 59.87]	0.1%	0.2%
Jun R_TH	1.08	0.4265	2.93	[1.27; 6.77]	4.2%	4.9%
Zhou M_MC	0.49	0.3301	1.63	[0.85; 3.11]	7.1%	6.0%
YuC_TH	0.57	0.1571	1.77	[1.30; 2.40]	31.2%	8.1%
Wang L_SPH	1.26	0.6263	3.54	[1.04; 12.09]	2.0%	3.2%
Bi Q_STPH	0.68	0.5218	1.98	[0.71; 5.51]	2.8%	4.0%
LiX_TH	-0.44	0.3322	0.64	[0.34; 1.23]	7.0%	6.0%
Li Y_TH	1.25	0.9449	3.50	[0.55; 22.30]	0.9%	1.8%
Fixed effect model			1.82	[1.53; 2.16]	100.0%	--
Random effects model			1.85	[1.40; 2.43]	--	100.0%
Heterogeneity: $I^2 = 43\%$, $\tau^2 = 0.2221$, $p < 0.01$						
**Fixed effect model**			**1.82**	**[1.53; 2.16]**	**100.0%**	**--**
**Random effects model**			**1.85**	**[1.40; 2.43]**	**--**	**100.0%**
Heterogeneity: $I^2 = 43\%$, $\tau^2 = 0.2221$, $p < 0.01$						
Residual heterogeneity: $I^2 = 43\%$, $p < 0.01$						

Candidate variable: Low blood pressure, outcome: severe Covid-19 disease, subgroup analysis: (crude vs adjusted)

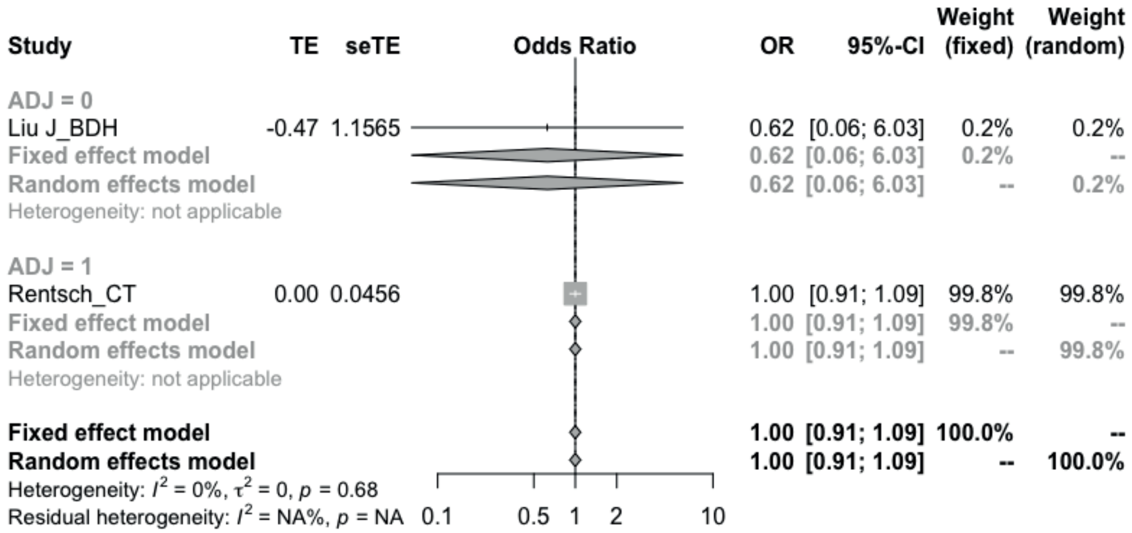

Candidate variable: High fever (more than 39°C), outcome: severe Covid-19 disease

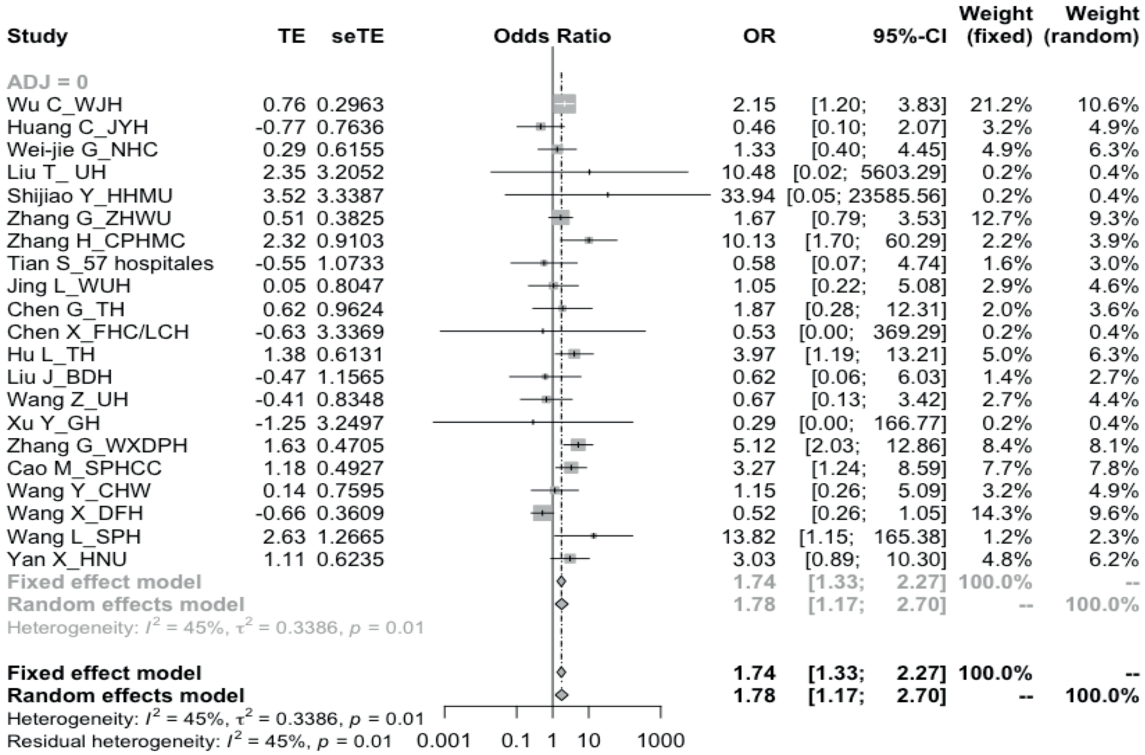

Candidate variable: Fever, outcome: severe Covid-19 disease, subgroup analysis by risk of bias (high vs moderate/low)

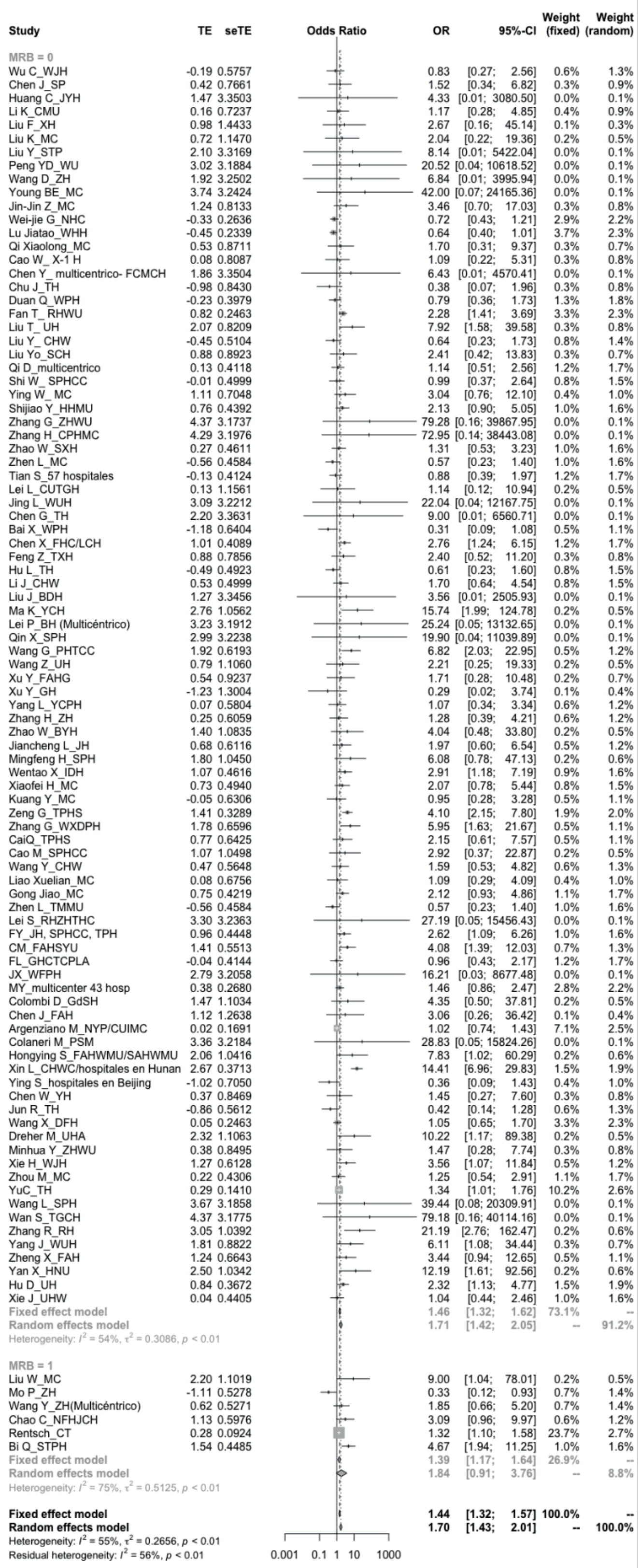

Candidate variable: Rhinorrhea, outcome: severe Covid-19 disease

Study	TE	seTE	OR	95%-CI	Weight (fixed)	Weight (random)
ADJ = 0						
Peng YD_WU	0.45	0.8413	1.57	[0.30; 8.17]	2.4%	3.9%
Young BE_MC	-1.79	3.3747	0.17	[0.00; 124.27]	0.1%	0.3%
Wei-jie G_NHC	-0.52	0.7321	0.59	[0.14; 2.49]	3.1%	4.7%
Lu Jiatao_WHH	-2.46	3.2045	0.09	[0.00; 45.75]	0.2%	0.4%
Chu J_TH	0.96	3.3338	2.62	[0.00; 1802.56]	0.2%	0.3%
Duan Q_WPH	-3.00	0.5809	0.05	[0.02; 0.16]	4.9%	6.1%
Liu T_ UH	2.15	3.2103	8.59	[0.02; 4643.14]	0.2%	0.4%
Liu Yo_SCH	-3.00	3.2187	0.05	[0.00; 27.40]	0.2%	0.4%
Qi D_multicentrico	-0.15	0.4050	0.86	[0.39; 1.91]	10.2%	8.4%
Ying W_ MC	-0.51	0.7463	0.60	[0.14; 2.59]	3.0%	4.5%
Shijiao Y_HHMU	-2.82	3.1945	0.06	[0.00; 31.07]	0.2%	0.4%
Zhang H_CPHMC	-1.42	1.1248	0.24	[0.03; 2.19]	1.3%	2.5%
Zhen L_MC	-3.25	3.1980	0.04	[0.00; 20.37]	0.2%	0.4%
Lei L_CUTGH	2.84	1.3127	17.20	[1.31; 225.37]	1.0%	1.9%
Jing L_WUH	-1.50	3.3505	0.22	[0.00; 158.04]	0.1%	0.3%
Chen X_FHC/LCH	0.68	0.8515	1.97	[0.37; 10.44]	2.3%	3.8%
Li J_CHW	0.24	1.4250	1.28	[0.08; 20.83]	0.8%	1.7%
Zhao W_BYH	-0.06	0.8613	0.94	[0.17; 5.11]	2.2%	3.7%
Huang H_GEPH	0.46	0.5167	1.58	[0.57; 4.35]	6.2%	6.9%
Kuang Y_MC	-3.24	3.1885	0.04	[0.00; 20.25]	0.2%	0.4%
Zeng G_TPHS	0.28	0.5988	1.32	[0.41; 4.26]	4.7%	5.9%
Chen J_FAH	-3.41	3.2591	0.03	[0.00; 19.70]	0.2%	0.4%
Argenziano M_NYP/CUIMC	-0.00	0.2799	1.00	[0.58; 1.73]	21.3%	10.3%
Hongying S_FAHWMU/SAHWMU	-0.70	1.0815	0.50	[0.06; 4.13]	1.4%	2.6%
Ying S_hospitales en Beijing	-1.38	3.3446	0.25	[0.00; 176.78]	0.1%	0.3%
Chen W_YH	-1.44	3.2360	0.24	[0.00; 134.97]	0.2%	0.4%
Wang X_DFH	0.43	0.3968	1.53	[0.70; 3.33]	10.6%	8.5%
Dreher M_UHA	2.32	3.3455	10.17	[0.01; 7165.01]	0.1%	0.3%
YuC_TH	1.02	1.1557	2.78	[0.29; 26.78]	1.2%	2.4%
Wang L_SPH	-2.18	3.2085	0.11	[0.00; 60.85]	0.2%	0.4%
Yan X_HNU	-2.02	3.1963	0.13	[0.00; 69.78]	0.2%	0.4%
Bi Q_STPH	0.14	0.3843	1.15	[0.54; 2.44]	11.3%	8.7%
Wu J_TFAH	0.55	0.4157	1.73	[0.77; 3.90]	9.7%	8.2%
Fixed effect model			0.96	[0.75; 1.24]	100.0%	--
Random effects model			0.89	[0.60; 1.31]	--	100.0%
Heterogeneity: $I^2 = 34\%$, $\tau^2 = 0.3009$, $p = 0.03$						
**Fixed effect model**			**0.96**	**[0.75; 1.24]**	**100.0%**	**--**
**Random effects model**			**0.89**	**[0.60; 1.31]**	**--**	**100.0%**
Heterogeneity: $I^2 = 34\%$, $\tau^2 = 0.3009$, $p = 0.03$						
Residual heterogeneity: $I^2 = 34\%$, $p = 0.03$						

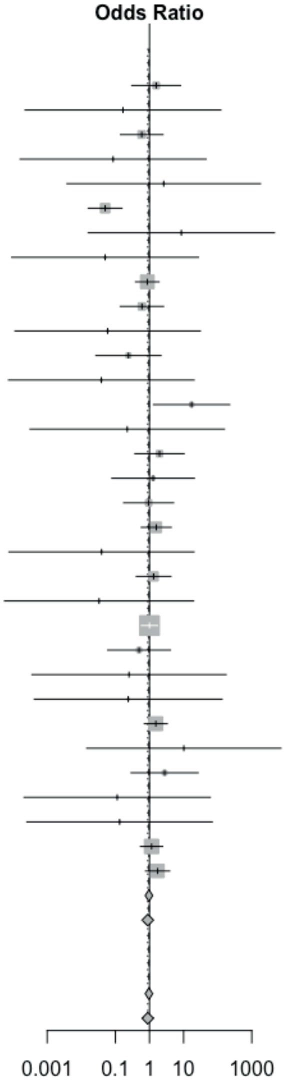

Candidate variable: Odynophagia, outcome: severe Covid-19 disease, subgroup analysis: (crude vs adjusted)

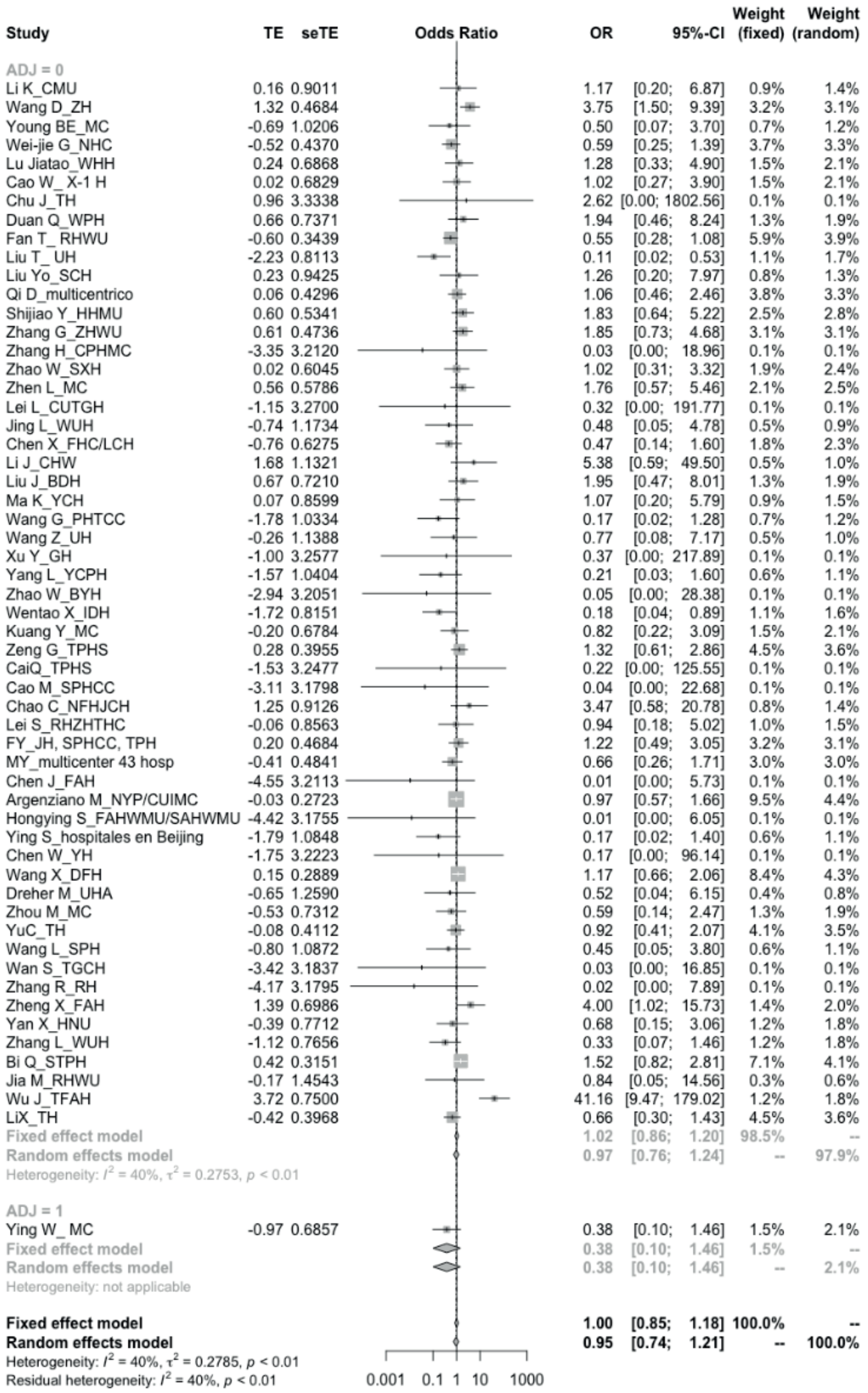

Candidate variable: Conjunctivitis, outcome: severe Covid-19 disease

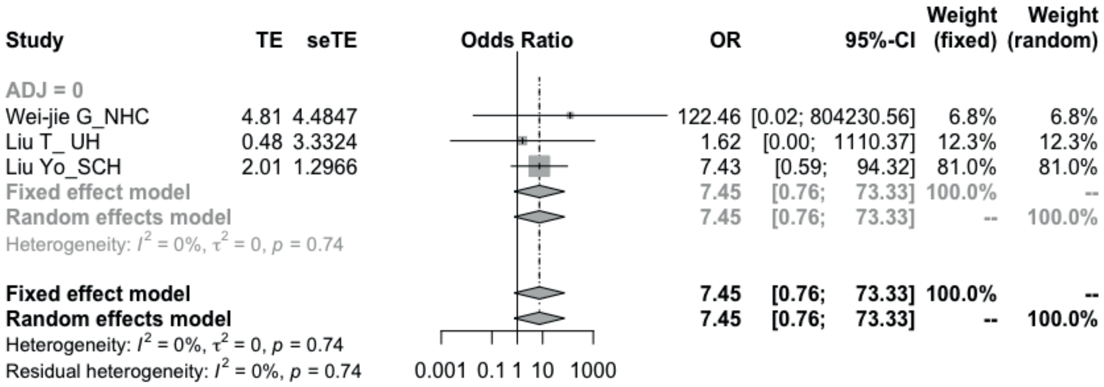

Candidate variable: Cough, outcome: severe Covid-19 disease, subgroup analysis by risk of bias (high vs moderate/low)

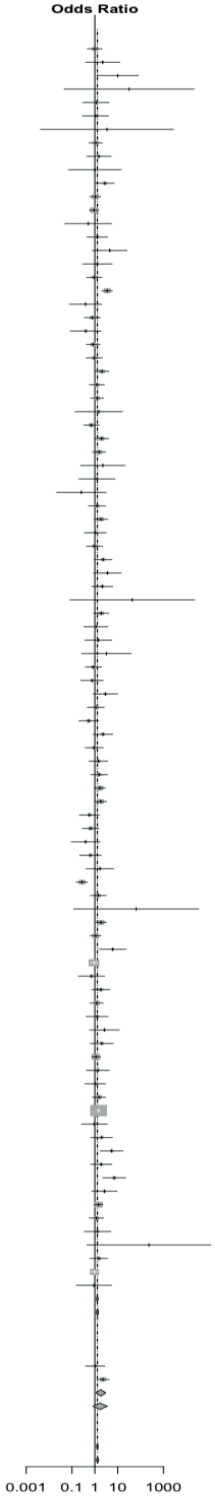

Study	TE	seTE	OR	95%-CI	Weight (fixed)	Weight (random)
**MRB = 0**						
Wu C_WJH	-0.02	0.3649	0.98	[0.48; 2.01]	1.2%	1.5%
Huang C_JYH	0.79	0.8751	2.20	[0.40; 12.23]	0.2%	0.4%
Li K_CMU	2.30	1.0606	9.95	[1.24; 79.55]	0.1%	0.3%
Liu F_XH	3.46	3.3255	31.67	[0.05; 21444.23]	0.0%	0.0%
Liu K_MC	0.12	0.6604	1.12	[0.31; 4.10]	0.4%	0.6%
Liu W_MC	0.06	0.6584	1.06	[0.29; 3.85]	0.4%	0.6%
Liu Y_STP	1.22	3.4013	3.37	[0.00; 2651.48]	0.0%	0.0%
Mo P_ZH	0.09	0.3333	1.09	[0.57; 2.10]	1.4%	1.6%
Peng YD_WU	0.41	0.6166	1.50	[0.45; 5.02]	0.4%	0.7%
Young BE_MC	0.00	1.3416	1.00	[0.07; 13.87]	0.1%	0.2%
Jin-Jin Z_MC	1.01	0.4636	2.75	[1.11; 6.82]	0.7%	1.1%
Wei-jie G_NHC	0.04	0.2716	1.04	[0.61; 1.78]	2.1%	2.0%
Lu Jiatao_WHH	-0.07	0.2280	0.93	[0.59; 1.45]	3.0%	2.3%
Qi Xiaolong_MC	-0.66	1.1825	0.51	[0.05; 5.22]	0.1%	0.2%
Cao W_ X-1 H	0.24	0.5246	1.27	[0.45; 3.54]	0.6%	0.9%
Chen Y_ multicentrico- FCMCH	1.50	0.8746	4.50	[0.81; 24.98]	0.2%	0.4%
Chu J_TH	0.25	0.7511	1.29	[0.30; 5.61]	0.3%	0.5%
Duan Q_WPH	-0.05	0.3858	0.95	[0.45; 2.02]	1.1%	1.4%
Fan T_ RHWU	1.25	0.2595	3.48	[2.09; 5.79]	2.3%	2.0%
Liu T_UH	-0.94	0.8209	0.39	[0.08; 1.95]	0.2%	0.4%
Liu Y_ CHW	-0.24	0.3943	0.78	[0.36; 1.70]	1.0%	1.3%
Liu Yo_SCH	-0.92	0.7853	0.40	[0.09; 1.86]	0.3%	0.5%
Qi D_multicentrico	-0.16	0.3384	0.85	[0.44; 1.65]	1.4%	1.6%
Shi W_ SPHCC	-0.05	0.4071	0.95	[0.43; 2.10]	0.9%	1.3%
Ying W_ MC	0.72	0.3512	2.05	[1.03; 4.08]	1.3%	1.5%
Shijiao Y_HHMU	0.20	0.3766	1.22	[0.59; 2.56]	1.1%	1.4%
Zhang G_ZHWU	0.22	0.3249	1.25	[0.66; 2.36]	1.5%	1.7%
Zhang H_CPHMC	0.41	1.2036	1.50	[0.14; 15.87]	0.1%	0.2%
Zhao W_SXH	-0.35	0.3808	0.71	[0.33; 1.49]	1.1%	1.4%
Zhen L_MC	0.68	0.3540	1.98	[0.99; 3.95]	1.3%	1.5%
Tian S_57 hospitales	0.42	0.3262	1.52	[0.80; 2.87]	1.5%	1.6%
Lei L_CUTGH	0.81	1.1319	2.25	[0.24; 20.69]	0.1%	0.2%
Jing L_WUH	0.22	0.9145	1.25	[0.21; 7.50]	0.2%	0.4%
Chen G_TH	-1.35	1.2599	0.26	[0.02; 3.06]	0.1%	0.2%
Bai X_WPH	0.21	0.4283	1.24	[0.53; 2.87]	0.9%	1.2%
Chen X_FHC/LCH	0.62	0.3409	1.86	[0.95; 3.62]	1.3%	1.6%
Feng Z_TXH	0.04	0.5474	1.04	[0.36; 3.04]	0.5%	0.8%
Hu L_TH	-0.03	0.4090	0.97	[0.43; 2.16]	0.9%	1.3%
Li J_CHW	0.84	0.4421	2.31	[0.97; 5.48]	0.8%	1.1%
Liu J_BDH	1.27	0.7052	3.55	[0.89; 14.13]	0.3%	0.6%
Ma K_YCH	0.74	0.5315	2.10	[0.74; 5.97]	0.6%	0.9%
Qin X_SPH	3.76	3.1966	43.02	[0.08; 22629.01]	0.0%	0.0%
Wang G_PHTCC	0.65	0.3780	1.91	[0.91; 4.00]	1.1%	1.4%
Wang Z_UH	0.11	0.6041	1.11	[0.34; 3.63]	0.4%	0.7%
Xu Y_FAHG	0.34	0.6712	1.41	[0.38; 5.26]	0.3%	0.6%
Xu Y_GH	1.17	1.2609	3.22	[0.27; 38.15]	0.1%	0.2%
Yang L_YCPH	-0.13	0.4044	0.87	[0.40; 1.93]	1.0%	1.3%
Zhang H_ZH	-0.28	0.5627	0.76	[0.25; 2.28]	0.5%	0.8%
Zhao W_BYH	1.07	0.6200	2.91	[0.86; 9.81]	0.4%	0.7%
Huang H_GEPH	0.10	0.4222	1.10	[0.48; 2.52]	0.9%	1.2%
Jiancheng L_JH	-0.62	0.4853	0.54	[0.21; 1.39]	0.7%	1.0%
Mingfeng H_SPH	0.82	0.4914	2.28	[0.87; 5.96]	0.6%	1.0%
Wentao X_IDH	-0.06	0.4479	0.94	[0.39; 2.26]	0.8%	1.1%
Xiaofei H_MC	0.36	0.4734	1.43	[0.57; 3.63]	0.7%	1.0%
Kuang Y_MC	0.42	0.4205	1.52	[0.67; 3.47]	0.9%	1.2%
Zeng G_TPHS	0.50	0.2665	1.65	[0.98; 2.77]	2.2%	2.0%
CaiQ_TPHS	0.59	0.2971	1.81	[1.01; 3.24]	1.8%	1.8%
Cao M_SPHCC	-0.55	0.4985	0.58	[0.22; 1.53]	0.6%	1.0%
Wang Y_CHW	-0.42	0.4095	0.66	[0.29; 1.47]	0.9%	1.3%
Liao Xuelian_MC	-0.93	0.7298	0.39	[0.09; 1.65]	0.3%	0.5%
Chao C_NFHJCH	-0.44	0.5446	0.64	[0.22; 1.87]	0.5%	0.8%
Lei S_RHZHTHC	0.51	0.6992	1.67	[0.42; 6.56]	0.3%	0.6%
FY_JH, SPHCC, TPH	-1.30	0.2800	0.27	[0.16; 0.47]	2.0%	1.9%
FL_GHCTCPLA	0.34	0.3993	1.41	[0.64; 3.08]	1.0%	1.3%
JX_WFPH	4.16	3.1955	64.31	[0.12; 33747.31]	0.0%	0.0%
MY_multicenter 43 hosp	0.62	0.2708	1.85	[1.09; 3.15]	2.1%	2.0%
Colombi D_GdSH	0.08	0.2682	1.08	[0.64; 1.83]	2.2%	2.0%
Chen J_FAH	1.80	0.6792	6.05	[1.60; 22.90]	0.3%	0.6%
Argenziano M_NYP/CUIMC	-0.09	0.1676	0.92	[0.66; 1.27]	5.6%	2.7%
Colaneri M_PSM	-0.34	0.6649	0.71	[0.19; 2.61]	0.4%	0.6%
Hongying S_FAHWMU/SAHWMU	0.61	0.4553	1.84	[0.75; 4.49]	0.8%	1.1%
Xin L_CHWC/hospitales en Hunan	0.18	0.3221	1.20	[0.64; 2.25]	1.5%	1.7%
Ying S_hospitales en Beijing	0.23	0.5541	1.26	[0.42; 3.72]	0.5%	0.8%
Chen W_YH	0.97	0.7501	2.64	[0.61; 11.50]	0.3%	0.5%
Jun R_TH	0.70	0.5807	2.01	[0.64; 6.28]	0.5%	0.8%
Wang X_DFH	0.11	0.2117	1.12	[0.74; 1.70]	3.5%	2.4%
Dreher M_UHA	0.30	0.5747	1.35	[0.44; 4.18]	0.5%	0.8%
Xie H_WJH	0.04	0.5191	1.04	[0.38; 2.88]	0.6%	0.9%
Zhou M_MC	0.41	0.3250	1.51	[0.80; 2.86]	1.5%	1.7%
YuC_TH	0.37	0.1030	1.45	[1.18; 1.77]	14.8%	3.2%
Wang L_SPH	-0.04	0.6505	0.96	[0.27; 3.43]	0.4%	0.6%
Wan S_TGCH	0.68	0.5514	1.98	[0.67; 5.84]	0.5%	0.8%
Zhang R_RH	1.69	0.5773	5.44	[1.75; 16.86]	0.5%	0.8%
Yang J_WUH	0.64	0.5348	1.90	[0.67; 5.43]	0.5%	0.9%
Zheng X_FAH	1.96	0.5817	7.07	[2.26; 22.11]	0.5%	0.8%
Yan X_HNU	0.97	0.6379	2.63	[0.75; 9.19]	0.4%	0.7%
Zhang L_WUH	0.37	0.2150	1.44	[0.95; 2.20]	3.4%	2.4%
Xie J_UHW	0.15	0.3555	1.17	[0.58; 2.34]	1.2%	1.5%
Jia M_RHWU	0.29	0.6667	1.33	[0.36; 4.92]	0.4%	0.6%
Wu J_TFAH	5.44	3.1688	231.21	[0.46; 115166.86]	0.0%	0.0%
Zheng F_NHCFH	0.40	0.4368	1.49	[0.63; 3.50]	0.8%	1.2%
LiX_TH	-0.03	0.1993	0.97	[0.66; 1.44]	3.9%	2.5%
Li Y_TH	-0.10	0.8893	0.91	[0.16; 5.20]	0.2%	0.4%
Fixed effect model			1.28	[1.18; 1.38]	97.7%	--
Random effects model			1.30	[1.16; 1.45]	--	97.3%
Heterogeneity: $I^2 = 37\%$, $\tau^2 = 0.0919$, $p < 0.01$						
**MRB = 1**						
Wang Y_ZH(Multicéntrico)	0.04	0.4918	1.04	[0.40; 2.73]	0.6%	1.0%
Bi Q_STPH	0.82	0.3061	2.27	[1.25; 4.15]	1.7%	1.8%
Fixed effect model			1.83	[1.10; 3.04]	2.3%	--
Random effects model			1.69	[0.80; 3.56]	--	2.7%
Heterogeneity: $I^2 = 45\%$, $\tau^2 = 0.1386$, $p = 0.18$						
**Fixed effect model**			**1.29**	**[1.19; 1.39]**	**100.0%**	**--**
**Random effects model**			**1.31**	**[1.17; 1.46]**	**--**	**100.0%**
Heterogeneity: $I^2 = 38\%$, $\tau^2 = 0.0923$, $p < 0.01$						
Residual heterogeneity: $I^2 = 38\%$, $p < 0.01$						

Candidate variable: Productive Cough, outcome: severe Covid-19 disease, subgroup analysis: (crude vs adjusted)

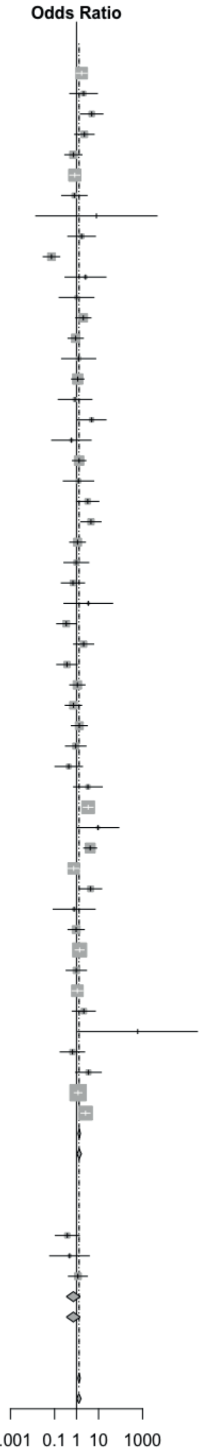

Study	TE	seTE	OR	95%-CI	Weight (fixed)	Weight (random)
ADJ = 0						
Wu C_WJH	0.53	0.2912	1.70	[0.96; 3.01]	4.4%	2.8%
Huang C_JYH	0.73	0.7360	2.08	[0.49; 8.81]	0.7%	1.4%
Li K_CMU	1.58	0.5996	4.88	[1.51; 15.79]	1.0%	1.7%
Liu K_MC	0.82	0.5191	2.26	[0.82; 6.25]	1.4%	2.0%
Wang D_ZH	-0.33	0.4570	0.72	[0.29; 1.76]	1.8%	2.2%
Wei-jie G_NHC	-0.19	0.2749	0.83	[0.48; 1.42]	4.9%	2.9%
Chen Y_ multicentrico- FCMCH	-0.22	0.6790	0.80	[0.21; 3.03]	0.8%	1.5%
Chu J_TH	2.08	3.2342	7.97	[0.01; 4515.72]	0.0%	0.1%
Duan Q_WPH	0.53	0.7346	1.70	[0.40; 7.17]	0.7%	1.4%
Fan T_ RHWU	-2.62	0.4390	0.07	[0.03; 0.17]	1.9%	2.3%
Liu T_ UH	0.93	1.0907	2.55	[0.30; 21.58]	0.3%	0.8%
Liu Yo_SCH	0.00	0.9258	1.00	[0.16; 6.14]	0.4%	1.0%
Shi W_ SPHCC	0.70	0.4061	2.02	[0.91; 4.49]	2.3%	2.4%
Shijiao Y_HHMU	-0.09	0.4067	0.91	[0.41; 2.02]	2.3%	2.4%
Zhang H_CPHMC	0.22	0.9083	1.25	[0.21; 7.41]	0.5%	1.1%
Zhen L_MC	0.12	0.3283	1.13	[0.59; 2.15]	3.5%	2.7%
Lei L_CUTGH	-0.15	0.8971	0.86	[0.15; 4.97]	0.5%	1.1%
Jing L_WUH	1.58	0.7660	4.85	[1.08; 21.76]	0.6%	1.3%
Chen G_TH	-0.54	1.0494	0.58	[0.07; 4.56]	0.3%	0.9%
Bai X_WPH	0.27	0.3588	1.31	[0.65; 2.64]	2.9%	2.6%
Feng Z_TXH	0.21	0.8107	1.23	[0.25; 6.03]	0.6%	1.2%
Liu J_BDH	1.17	0.5965	3.21	[1.00; 10.33]	1.0%	1.7%
Ma K_YCH	1.50	0.5401	4.50	[1.56; 12.97]	1.3%	1.9%
Wang G_PHTCC	0.11	0.4181	1.11	[0.49; 2.52]	2.1%	2.3%
Wang Z_UH	-0.03	0.6619	0.98	[0.27; 3.57]	0.8%	1.6%
Xu Y_FAHG	-0.38	0.6181	0.69	[0.20; 2.30]	1.0%	1.7%
Xu Y_GH	1.23	1.3004	3.42	[0.27; 43.70]	0.2%	0.6%
Zhang H_ZH	-1.08	0.5120	0.34	[0.12; 0.93]	1.4%	2.0%
Zhao W_BYH	0.74	0.5375	2.10	[0.73; 6.01]	1.3%	1.9%
Jiancheng L_JH	-1.00	0.5452	0.37	[0.13; 1.07]	1.3%	1.9%
Mingfeng H_SPH	0.09	0.4052	1.09	[0.49; 2.42]	2.3%	2.4%
Wentao X_IDH	-0.33	0.4388	0.72	[0.31; 1.71]	1.9%	2.3%
Kuang Y_MC	0.29	0.4268	1.33	[0.58; 3.08]	2.0%	2.3%
Cao M_SPHCC	-0.08	0.5470	0.92	[0.32; 2.69]	1.2%	1.9%
Liao Xuelian_MC	-0.82	0.7297	0.44	[0.11; 1.84]	0.7%	1.4%
Lei S_RHZHTHC	1.19	0.7645	3.28	[0.73; 14.68]	0.6%	1.3%
FY_JH, SPHCC, TPH	1.21	0.2659	3.37	[2.00; 5.67]	5.3%	2.9%
JX_WFPH	2.25	1.1090	9.45	[1.08; 83.06]	0.3%	0.8%
MY_multicenter 43 hosp	1.43	0.3423	4.17	[2.13; 8.15]	3.2%	2.6%
Argenziano M_NYP/CUIMC	-0.30	0.2964	0.74	[0.41; 1.32]	4.2%	2.8%
Ying S_hospitales en Beijing	1.46	0.5923	4.32	[1.35; 13.80]	1.1%	1.8%
Chen W_YH	-0.25	1.1198	0.78	[0.09; 6.98]	0.3%	0.8%
Jun R_TH	-0.04	0.4332	0.96	[0.41; 2.24]	2.0%	2.3%
Wang X_DFH	0.32	0.2394	1.38	[0.86; 2.20]	6.5%	3.0%
Xie H_WJH	-0.03	0.5419	0.97	[0.34; 2.82]	1.3%	1.9%
Zhou M_MC	0.09	0.2897	1.09	[0.62; 1.92]	4.4%	2.8%
Wang L_SPH	0.76	0.6148	2.13	[0.64; 7.12]	1.0%	1.7%
Zhang R_RH	6.39	3.1860	595.00	[1.16; 306484.97]	0.0%	0.1%
Yang J_WUH	-0.44	0.6512	0.65	[0.18; 2.32]	0.9%	1.6%
Zheng X_FAH	1.24	0.6858	3.46	[0.90; 13.26]	0.8%	1.5%
Zhang L_WUH	0.16	0.2054	1.17	[0.79; 1.76]	8.8%	3.1%
Bi Q_STPH	0.94	0.2440	2.55	[1.58; 4.12]	6.3%	3.0%
Fixed effect model			1.33	[1.18; 1.50]	97.3%	--
Random effects model			1.32	[1.06; 1.66]	--	95.5%
Heterogeneity: $I^2 = 64\%$, $\tau^2 = 0.3651$, $p < 0.01$						
ADJ = 1						
Ying W_ MC	-0.97	0.6415	0.38	[0.11; 1.34]	0.9%	1.6%
Liu R_CHW	-0.73	1.0556	0.48	[0.06; 3.80]	0.3%	0.8%
Wang Y_ZH(Multicéntrico)	0.14	0.5047	1.15	[0.43; 3.09]	1.5%	2.0%
Fixed effect model			0.71	[0.34; 1.47]	2.7%	--
Random effects model			0.71	[0.34; 1.47]	--	4.5%
Heterogeneity: $I^2 = 0\%$, $\tau^2 = 0$, $p = 0.37$						
**Fixed effect model**			**1.31**	**[1.16; 1.47]**	**100.0%**	**--**
**Random effects model**			**1.28**	**[1.03; 1.59]**	**--**	**100.0%**
Heterogeneity: $I^2 = 63\%$, $\tau^2 = 0.3614$, $p < 0.01$						
Residual heterogeneity: $I^2 = 63\%$, $p < 0.01$						

Candidate variable: Hemoptysis, outcome: severe Covid-19 disease

Study	TE	seTE	OR	95%-CI	Weight (fixed)	Weight (random)
ADJ = 0						
Huang C_JYH	0.73	1.4571	2.08	[0.12; 36.23]	6.0%	6.0%
Wei-jie G_NHC	1.37	0.8008	3.94	[0.82; 18.92]	19.7%	19.7%
Liu T_ UH	0.91	3.2637	2.48	[0.00; 1486.57]	1.2%	1.2%
Qi D_multicentrico	1.09	0.9267	2.97	[0.48; 18.28]	14.7%	14.7%
Shijiao Y_HHMU	3.52	3.3387	33.94	[0.05; 23585.56]	1.1%	1.1%
Bai X_WPH	2.07	1.1105	7.96	[0.90; 70.16]	10.3%	10.3%
Wang G_PHTCC	3.94	3.3382	51.25	[0.07; 35575.14]	1.1%	1.1%
Zhang H_ZH	0.82	1.2501	2.27	[0.20; 26.27]	8.1%	8.1%
Zeng G_TPHS	1.25	1.4202	3.48	[0.22; 56.30]	6.3%	6.3%
FY_JH, SPHCC, TPH	1.37	0.9222	3.95	[0.65; 24.08]	14.9%	14.9%
CM_FAHSYU	5.55	3.1955	257.84	[0.49; 135323.98]	1.2%	1.2%
JX_WFPH	1.88	1.4712	6.57	[0.37; 117.48]	5.9%	5.9%
Chen J_FAH	1.88	3.3469	6.58	[0.01; 4644.25]	1.1%	1.1%
Xin L_CHWC/hospitales en Hunan	1.75	1.2351	5.74	[0.51; 64.55]	8.3%	8.3%
Fixed effect model			4.39	[2.18; 8.81]	100.0%	--
Random effects model			4.39	[2.18; 8.81]	--	100.0%
Heterogeneity: $I^2 = 0\%$, $\tau^2 = 0$, $p = 0.99$						
**Fixed effect model**			**4.39**	**[2.18; 8.81]**	**100.0%**	**--**
**Random effects model**			**4.39**	**[2.18; 8.81]**	**--**	**100.0%**
Heterogeneity: $I^2 = 0\%$, $\tau^2 = 0$, $p = 0.99$						
Residual heterogeneity: $I^2 = 0\%$, $p = 0.99$						

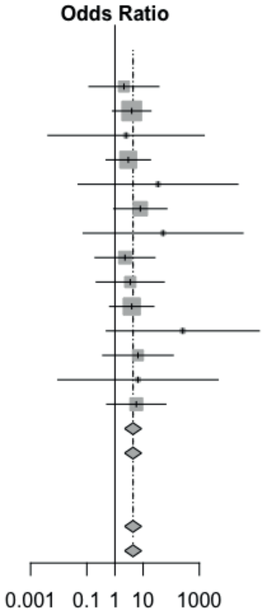

Candidate variable: Fatigue, outcome: severe Covid-19 disease, subgroup analysis: (crude vs adjusted)

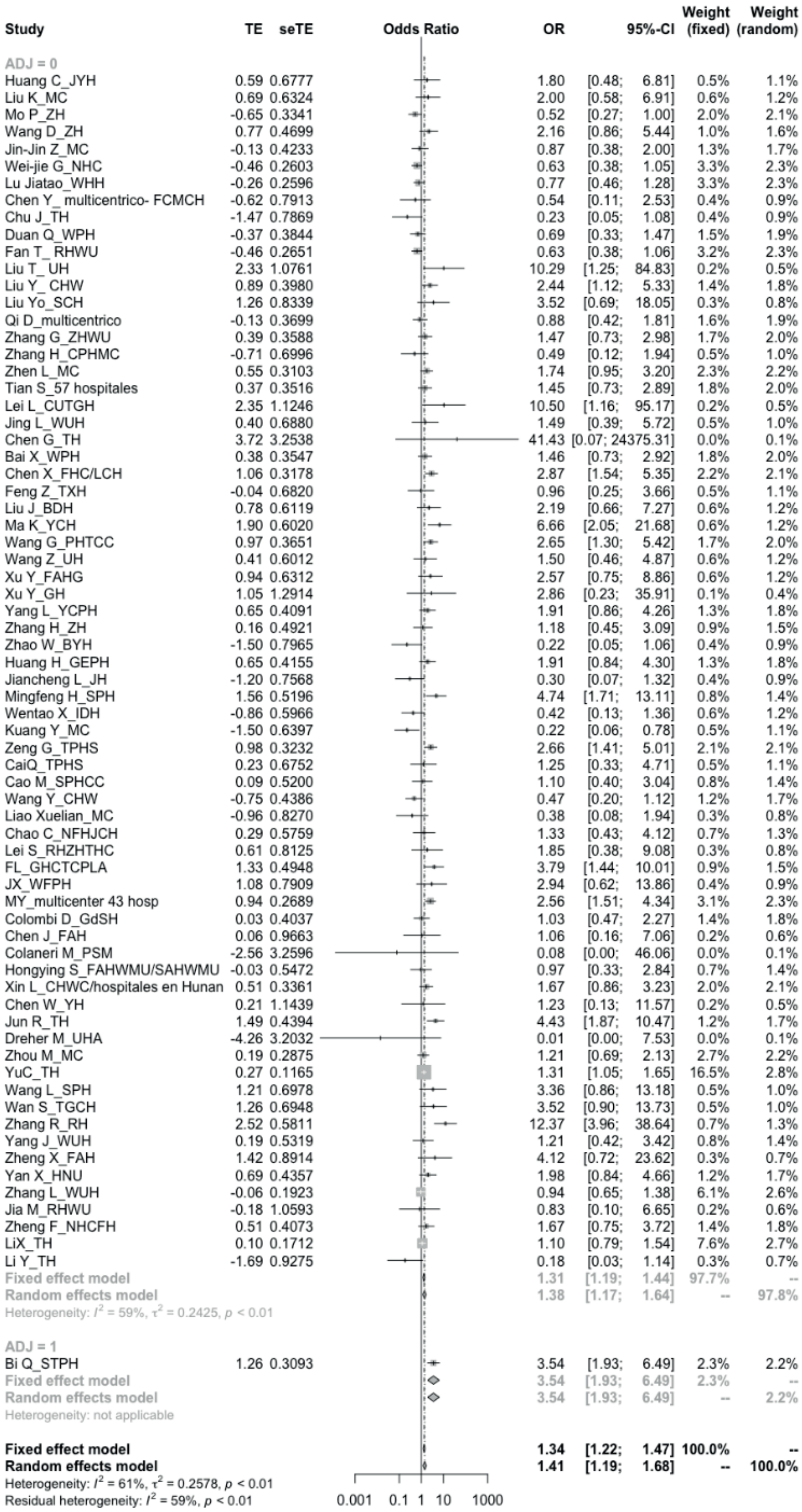

Candidate variable: Myalgia/arthralgia, outcome: severe Covid-19 disease, subgroup analysis: (crude vs adjusted)

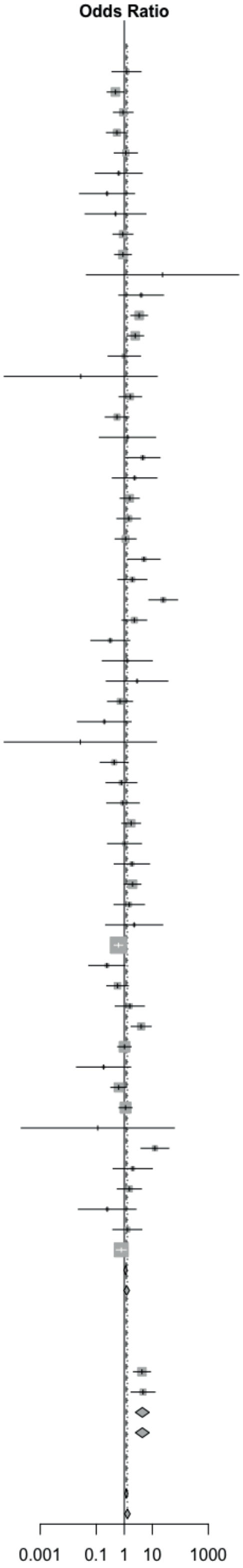

Study	TE	seTE	OR	95%-CI	Weight (fixed)	Weight (random)
**ADJ = 0**						
Li K_CMU	0.18	0.6089	1.20	[0.36; 3.96]	1.0%	1.7%
Mo P_ZH	-0.73	0.3486	0.48	[0.24; 0.96]	3.1%	2.5%
Wang D_ZH	-0.09	0.4098	0.92	[0.41; 2.05]	2.2%	2.3%
Wei-jie G_NHC	-0.61	0.4365	0.54	[0.23; 1.28]	2.0%	2.2%
Lu Jiatao_WHH	0.13	0.4858	1.13	[0.44; 2.94]	1.6%	2.0%
Qi Xiaolong_MC	-0.46	0.9845	0.63	[0.09; 4.35]	0.4%	1.0%
Chen Y_ multicentrico- FCMCH	-1.42	1.1533	0.24	[0.03; 2.33]	0.3%	0.8%
Chu J_TH	-0.72	1.2745	0.49	[0.04; 5.93]	0.2%	0.6%
Duan Q_WPH	-0.11	0.4160	0.89	[0.40; 2.02]	2.2%	2.2%
Fan T_ RHWU	-0.11	0.3509	0.90	[0.45; 1.79]	3.1%	2.5%
Liu T_ UH	3.14	3.1924	23.16	[0.04; 12080.20]	0.0%	0.1%
Liu Yo_SCH	1.39	0.9354	4.00	[0.64; 25.02]	0.4%	1.0%
Qi D_multicentrico	1.22	0.3500	3.39	[1.71; 6.74]	3.1%	2.5%
Ying W_ MC	0.90	0.3521	2.45	[1.23; 4.88]	3.0%	2.5%
Shijiao Y_HHMU	0.00	0.6803	1.00	[0.26; 3.79]	0.8%	1.5%
Zhang H_CPHMC	-3.58	3.2069	0.03	[0.00; 14.94]	0.0%	0.1%
Zhao W_SXH	0.50	0.4691	1.64	[0.66; 4.12]	1.7%	2.1%
Zhen L_MC	-0.60	0.4928	0.55	[0.21; 1.44]	1.6%	2.0%
Lei L_CUTGH	0.26	1.1799	1.30	[0.13; 13.13]	0.3%	0.7%
Jing L_WUH	1.52	0.7196	4.57	[1.12; 18.73]	0.7%	1.4%
Chen G_TH	0.85	0.9360	2.33	[0.37; 14.61]	0.4%	1.0%
Bai X_WPH	0.44	0.3972	1.56	[0.71; 3.39]	2.4%	2.3%
Chen X_FHC/LCH	0.36	0.4915	1.43	[0.55; 3.74]	1.6%	2.0%
Li J_CHW	0.11	0.4387	1.11	[0.47; 2.63]	2.0%	2.2%
Ma K_YCH	1.62	0.6746	5.06	[1.35; 18.97]	0.8%	1.5%
Lei P_BH (Multicéntrico)	0.66	0.6081	1.94	[0.59; 6.40]	1.0%	1.7%
Qin X_SPH	3.20	0.6003	24.50	[7.55; 79.46]	1.0%	1.7%
Wang G_PHTCC	0.83	0.5164	2.29	[0.83; 6.29]	1.4%	1.9%
Wang Z_UH	-1.15	0.8147	0.32	[0.06; 1.56]	0.6%	1.2%
Xu Y_FAHG	0.25	1.0483	1.28	[0.16; 9.97]	0.3%	0.9%
Xu Y_GH	1.05	1.2914	2.86	[0.23; 35.91]	0.2%	0.6%
Yang L_YCPH	-0.35	0.5243	0.71	[0.25; 1.97]	1.4%	1.9%
Zhang H_ZH	-1.64	1.1250	0.19	[0.02; 1.76]	0.3%	0.8%
Zhao W_BYH	-3.61	3.1911	0.03	[0.00; 14.03]	0.0%	0.1%
Huang H_GEPH	-0.83	0.5861	0.43	[0.14; 1.37]	1.1%	1.7%
Wentao X_IDH	-0.23	0.6475	0.79	[0.22; 2.81]	0.9%	1.6%
Kuang Y_MC	-0.11	0.6839	0.90	[0.24; 3.44]	0.8%	1.5%
Zeng G_TPHS	0.56	0.3924	1.76	[0.82; 3.79]	2.4%	2.3%
Chao C_NFHJCH	0.02	0.7098	1.02	[0.25; 4.09]	0.7%	1.4%
Lei S_RHZHTHC	0.62	0.7411	1.87	[0.44; 7.98]	0.7%	1.4%
FY_JH, SPHCC, TPH	0.68	0.3479	1.98	[1.00; 3.91]	3.1%	2.5%
FL_GHCTCPLA	0.41	0.6370	1.50	[0.43; 5.23]	0.9%	1.6%
Chen J_FAH	0.81	1.1960	2.25	[0.22; 23.46]	0.3%	0.7%
Argenziano M_NYP/CUIMC	-0.48	0.1831	0.62	[0.43; 0.89]	11.2%	3.0%
Hongying S_FAHWMU/SAHWMU	-1.42	0.7640	0.24	[0.05; 1.08]	0.6%	1.3%
Xin L_CHWC/hospitales en Hunan	-0.56	0.4518	0.57	[0.24; 1.39]	1.8%	2.1%
Ying S_hospitales en Beijing	0.45	0.6107	1.57	[0.47; 5.19]	1.0%	1.7%
Jun R_TH	1.38	0.4199	3.99	[1.75; 9.09]	2.1%	2.2%
Wang X_DFH	0.02	0.2806	1.02	[0.59; 1.76]	4.8%	2.7%
Dreher M_UHA	-1.70	1.1362	0.18	[0.02; 1.69]	0.3%	0.8%
Zhou M_MC	-0.46	0.3293	0.63	[0.33; 1.20]	3.5%	2.5%
YuC_TH	0.10	0.2706	1.11	[0.65; 1.88]	5.1%	2.7%
Wang L_SPH	-2.18	3.2085	0.11	[0.00; 60.85]	0.0%	0.1%
Zhang R_RH	2.52	0.5811	12.37	[3.96; 38.64]	1.1%	1.8%
Zheng X_FAH	0.69	0.8221	2.00	[0.40; 10.02]	0.6%	1.2%
Yan X_HNU	0.41	0.5070	1.51	[0.56; 4.09]	1.5%	2.0%
Jia M_RHWU	-1.40	1.2072	0.25	[0.02; 2.62]	0.3%	0.7%
Zheng F_NHCFH	0.25	0.6070	1.29	[0.39; 4.22]	1.0%	1.7%
LiX_TH	-0.25	0.2138	0.78	[0.51; 1.19]	8.2%	2.9%
**Fixed effect model**			1.11	[0.99; 1.26]	95.5%	--
**Random effects model**			1.22	[0.98; 1.52]	--	95.6%
Heterogeneity: $I^2 = 60\%$, $\tau^2 = 0.3524$, $p < 0.01$						
**ADJ = 1**						
Bi Q_STPH	1.45	0.3573	4.28	[2.12; 8.61]	3.0%	2.4%
ZhangX_MC	1.54	0.5007	4.67	[1.75; 12.46]	1.5%	2.0%
**Fixed effect model**			4.41	[2.49; 7.79]	4.5%	--
**Random effects model**			4.41	[2.49; 7.79]	--	4.4%
Heterogeneity: $I^2 = 0\%$, $\tau^2 = 0$, $p = 0.89$						
**Fixed effect model**			**1.18**	**[1.05; 1.34]**	**100.0%**	**--**
**Random effects model**			**1.29**	**[1.03; 1.62]**	**--**	**100.0%**
Heterogeneity: $I^2 = 64\%$, $\tau^2 = 0.4117$, $p < 0.01$						
Residual heterogeneity: $I^2 = 59\%$, $p < 0.01$						

Candidate variable: Headache, outcome: severe Covid-19 disease, subgroup analysis: (crude vs adjusted)

Stud y	TE	seTE	OR	95%−CI	Weight (fixed)	Weight (random)
ADJ = 0						
Huang C_JYH	−2.84	3.2352	0.06	[0.00; 33.11]	0.1%	0.1%
Li K_CMU	0.17	0.7514	1.18	[0.27; 5.15]	1.2%	1.7%
Mo P_ZH	0.33	0.7488	1.40	[0.32; 6.06]	1.2%	1.7%
Wang D_ZH	0.37	0.7353	1.45	[0.34; 6.15]	1.2%	1.7%
Wei−jie G_NHC	−0.16	0.3874	0.85	[0.40; 1.82]	4.4%	3.5%
Lu Jiatao_WHH	0.44	0.4411	1.55	[0.65; 3.67]	3.4%	3.1%
Qi Xiaolong_MC	−0.12	0.8515	0.89	[0.17; 4.72]	0.9%	1.4%
Chen Y_ m ulticentr ico− FCMCH	−0.34	1.2733	0.71	[0.06; 8.66]	0.4%	0.7%
Duan Q_WPH	−1.79	0.7773	0.17	[0.04; 0.76]	1.1%	1.6%
Fan T_ RHWU	−0.74	0.4723	0.48	[0.19; 1.20]	3.0%	2.9%
Liu T_ UH	2.67	3.1987	14.43	[0.03; 7619.45]	0.1%	0.1%
Liu Yo_SCH	−1.92	3.2676	0.15	[0.00; 88.37]	0.1%	0.1%
Shijiao Y_HHMU	0.79	0.5929	2.20	[0.69; 7.05]	1.9%	2.3%
Zhang G_ZHWU	−0.08	0.5942	0.92	[0.29; 2.96]	1.9%	2.3%
Zhang H_CPHMC	−0.45	0.8908	0.64	[0.11; 3.66]	0.8%	1.3%
Zhao W_SXH	0.50	1.0185	1.65	[0.22; 12.15]	0.6%	1.1%
Zhen L_MC	−0.78	0.5812	0.46	[0.15; 1.43]	2.0%	2.4%
Tian S_57 hospitales	0.01	0.6580	1.01	[0.28; 3.66]	1.5%	2.0%
Lei L_CUTGH	−2.17	3.2206	0.11	[0.00; 62.68]	0.1%	0.1%
Jing L_WUH	−0.45	0.8973	0.64	[0.11; 3.69]	0.8%	1.3%
Chen G_TH	0.00	1.4907	1.00	[0.05; 18.57]	0.3%	0.5%
Bai X_WPH	0.18	0.5111	1.20	[0.44; 3.27]	2.5%	2.7%
Chen X_FHC/LCH	0.52	0.5416	1.67	[0.58; 4.84]	2.3%	2.6%
Feng Z_TXH	0.78	1.1531	2.18	[0.23; 20.88]	0.5%	0.9%
Hu L_TH	−0.94	3.2228	0.39	[0.00; 215.88]	0.1%	0.1%
Li J_CHW	0.26	0.7296	1.29	[0.31; 5.39]	1.2%	1.8%
Liu J_BDH	−1.17	0.7062	0.31	[0.08; 1.24]	1.3%	1.8%
Ma K_YCH	−2.56	3.2129	0.08	[0.00; 41.77]	0.1%	0.1%
Qin X_SPH	0.36	0.5703	1.44	[0.47; 4.40]	2.0%	2.4%
Wang G_PHTCC	1.22	0.5894	3.40	[1.07; 10.80]	1.9%	2.3%
Wang Z_UH	−3.43	3.1928	0.03	[0.00; 16.95]	0.1%	0.1%
Xu Y_GH	1.68	1.3316	5.38	[0.40; 73.09]	0.4%	0.7%
Yang L_YCPH	−0.84	0.7641	0.43	[0.10; 1.93]	1.1%	1.6%
Zhao W_BYH	−3.74	3.1893	0.02	[0.00; 12.31]	0.1%	0.1%
Huang H_GEPH	0.47	0.4921	1.60	[0.61; 4.21]	2.7%	2.8%
Jiancheng L_JH	−0.62	1.4271	0.54	[0.03; 8.83]	0.3%	0.6%
Wentao X_IDH	−0.74	1.2440	0.48	[0.04; 5.46]	0.4%	0.8%
Zeng G_TPHS	−0.34	0.5662	0.71	[0.23; 2.16]	2.1%	2.4%
CaiQ_TPHS	−2.49	3.1977	0.08	[0.00; 43.58]	0.1%	0.1%
Cao M_SPHCC	−3.38	3.1781	0.03	[0.00; 17.31]	0.1%	0.1%
Lei S_RHZHTHC	0.98	0.8342	2.67	[0.52; 13.68]	0.9%	1.4%
JX_WFPH	0.49	0.9040	1.62	[0.28; 9.56]	0.8%	1.3%
Argenziano M_NYP/CUIMC	−0.58	0.2907	0.56	[0.32; 0.99]	7.8%	4.2%
Xin L_CHWC/hospitales en Hunan	−0.08	0.6000	0.92	[0.28; 2.99]	1.8%	2.3%
Ying S_hospitales en Beijing	−1.23	1.1070	0.29	[0.03; 2.57]	0.5%	0.9%
Chen W_YH	0.95	1.2143	2.58	[0.24; 27.91]	0.4%	0.8%
Jun R_TH	−2.03	0.4424	0.13	[0.06; 0.31]	3.4%	3.1%
Wang X_DFH	0.32	0.2716	1.37	[0.81; 2.34]	9.0%	4.4%
Dreher M_UHA	−2.16	3.3455	0.12	[0.00; 81.50]	0.1%	0.1%
Zhou M_MC	−0.14	0.4678	0.87	[0.35; 2.18]	3.0%	3.0%
YuC_TH	0.27	0.3803	1.32	[0.62; 2.77]	4.6%	3.6%
Wang L_SPH	−2.38	3.2031	0.09	[0.00; 49.32]	0.1%	0.1%
Zheng X_F AH	−2.48	0.6669	0.08	[0.02; 0.31]	1.5%	2.0%
Yan X_HNU	0.35	0.5895	1.42	[0.45; 4.50]	1.9%	2.3%
Bi Q_STPH	0.28	0.3597	1.32	[0.65; 2.67]	5.1%	3.7%
Jia M_RHWU	−0.93	1.2725	0.39	[0.03; 4.78]	0.4%	0.7%
Wu J_TF AH	7.27	3.1674	1437.56	[2.89; 714102.39]	0.1%	0.1%
Zheng F_NHCFH	0.86	0.6493	2.37	[0.66; 8.44]	1.6%	2.1%
LiX_TH	−0.40	0.2743	0.67	[0.39; 1.15]	8.8%	4.4%
Fixed eff ect model			0.89	[0.76; 1.04]	97.8%	—
Random eff ects model			0.89	[0.71; 1.11]	—	96.8%
Heterogeneity: $I^2 = 35\%$, $\tau^2 = 0.2169$, $p < 0.01$						
ADJ = 1						
Ying W_ MC	−1.11	0.7426	0.33	[0.08; 1.41]	1.2%	1.7%
Zhang R_RH	1.36	0.8160	3.90	[0.79; 19.30]	1.0%	1.5%
Fixed eff ect model			1.01	[0.34; 2.96]	2.2%	—
Random eff ects model			1.11	[0.10; 12.46]	—	3.2%
Heterogeneity: $I^2 = 80\%$, $\tau^2 = 2.4409$, $p = 0.03$						
**Fixed eff ect model**			**0.89**	**[0.76; 1.04]**	**100.0%**	**—**
**Random eff ects model**			**0.89**	**[0.71; 1.12]**	**—**	**100.0%**
Heterogeneity: $I^2 = 36\%$, $\tau^2 = 0.2329$, $p < 0.01$						
Residual heterogeneity: $I^2 = 37\%$, $p < 0.01$						

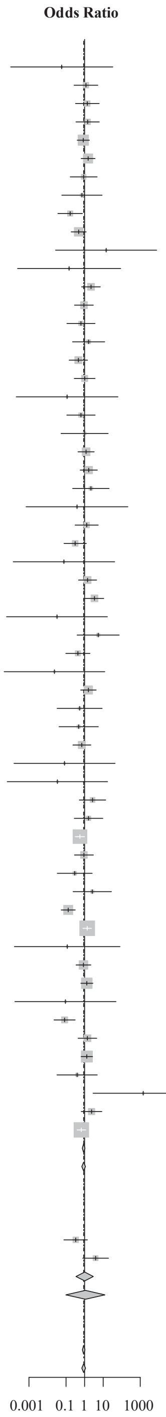

Candidate variable: Vomits, outcome: severe Covid-19 disease, subgroup analysis: (crude vs adjusted)

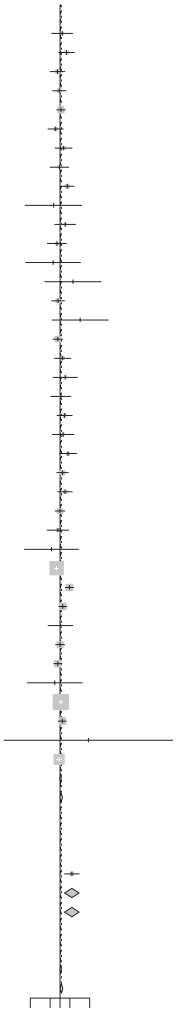

Stud y	TE	seTE	OR	95%−CI	Weight (fixed)	Weight (random)
ADJ = 0						
Mo P_ZH	0.51	1.2355	1.66	[0.15; 18.73]	0.6%	1.4%
Wang D_ZH	1.51	0.9347	4.55	[0.73; 28.39]	1.1%	2.2%
Jin−Jin Z_MC	−0.58	0.8551	0.56	[0.10; 2.99]	1.3%	2.5%
Lu Jiatao_WHH	−0.17	0.7989	0.84	[0.18; 4.03]	1.5%	2.7%
Duan Q_WPH	0.21	0.5100	1.23	[0.45; 3.35]	3.6%	4.4%
Liu T_ UH	−1.05	0.9092	0.35	[0.06; 2.09]	1.1%	2.3%
Liu Yo_SCH	0.83	1.0089	2.29	[0.32; 16.51]	0.9%	1.9%
Qi D_m ulticentr ico	−0.14	1.1068	0.87	[0.10; 7.57]	0.8%	1.7%
Shijiao Y_HHMU	1.68	0.7889	5.38	[1.15; 25.23]	1.5%	2.7%
Zhang H_CPHMC	−1.50	3.3493	0.22	[0.00; 157.66]	0.1%	0.2%
Zhao W_SXH	1.21	1.2398	3.35	[0.29; 38.04]	0.6%	1.4%
Zhen L_MC	−0.72	1.1286	0.48	[0.05; 4.42]	0.7%	1.6%
Lei L_CUTGH	−1.60	3.2422	0.20	[0.00; 116.15]	0.1%	0.2%
Jing L_WUH	3.01	3.3513	20.25	[0.03; 14422.44]	0.1%	0.2%
Chen X_FHC/LCH	−0.47	0.7694	0.63	[0.14; 2.84]	1.6%	2.8%
Hu L_TH	4.67	3.3398	106.92	[0.15; 74452.09]	0.1%	0.2%
Li J_CHW	−0.51	0.5778	0.60	[0.19; 1.87]	2.8%	3.9%
Liu J_BDH	0.60	0.9614	1.82	[0.28; 11.99]	1.0%	2.1%
Ma K_YCH	1.20	1.4382	3.32	[0.20; 55.56]	0.5%	1.1%
Lei P_BH (Multicéntr ico)	0.15	1.1797	1.17	[0.12; 11.78]	0.7%	1.5%
Wang G_PHTCC	1.05	0.8852	2.87	[0.51; 16.28]	1.2%	2.3%
Wang Z_UH	0.71	1.2632	2.04	[0.17; 24.24]	0.6%	1.4%
Yang L_YCPH	1.83	1.0212	6.26	[0.85; 46.32]	0.9%	1.9%
Zhang H_ZH	0.58	0.6981	1.79	[0.46; 7.03]	1.9%	3.2%
Zhao W_BYH	1.16	0.8625	3.18	[0.59; 17.22]	1.3%	2.4%
Zeng G_TPHS	−0.02	0.5825	0.98	[0.31; 3.08]	2.8%	3.9%
Lei S_RHZHTHC	−0.50	1.2768	0.61	[0.05; 7.41]	0.6%	1.3%
JX_WFPH	−1.98	3.2256	0.14	[0.00; 76.72]	0.1%	0.2%
Argenziano M_NYP/CUIMC	−0.80	0.2377	0.45	[0.28; 0.72]	16.7%	6.7%
Jun R_TH	2.20	0.4851	9.00	[3.48; 23.29]	4.0%	4.6%
Wang X_DFH	0.63	0.4602	1.88	[0.76; 4.62]	4.5%	4.8%
Dreher M_UHA	0.08	1.4434	1.09	[0.06; 18.40]	0.5%	1.1%
Zhou M_MC	0.01	0.5269	1.01	[0.36; 2.84]	3.4%	4.3%
YuC_TH	−0.54	0.4860	0.59	[0.23; 1.52]	4.0%	4.6%
Zheng X_F AH	−1.23	3.2557	0.29	[0.00; 172.90]	0.1%	0.2%
Zhang L_WUH	0.19	0.2082	1.21	[0.80; 1.82]	21.8%	6.9%
Bi Q_STPH	0.53	0.4737	1.69	[0.67; 4.28]	4.2%	4.7%
Wu J_TF AH	6.60	10.0174	736.29	[0.00; 247671024477.10]	0.0%	0.0%
LiX_TH	−0.20	0.3130	0.82	[0.44; 1.51]	9.6%	6.0%
Fixed eff ect model			1.10	[0.91; 1.33]	98.7%	—
Random eff ects model			1.29	[0.95; 1.74]	—	97.6%
Heterogeneity: $I^2 = 39\%$, $^2 = 0.2539$, $p < 0.01$						
ADJ = 1						
ZhangX_MC	2.74	0.8638	15.55	[2.86; 84.52]	1.3%	2.4%
Fixed eff ect model			15.55	[2.86; 84.52]	1.3%	—
Random eff ects model			15.55	[2.86; 84.52]	—	2.4%
Heterogeneity: not applicab le						
**Fixed eff ect model**			**1.14**	**[0.94; 1.37]**	**100.0%**	**—**
**Random eff ects model**			**1.38**	**[1.01; 1.90]**	**—**	**100.0%**
Heterogeneity: $I^2 = 45\%$, $^2 = 0.3367$, $p < 0.01$						
Residual heterogeneity: $I^2 = 39\%$, $p < 0.01$						

Candidate variable: Diarrhea, outcome: severe Covid-19 disease, subgroup analysis: (crude vs adjusted)

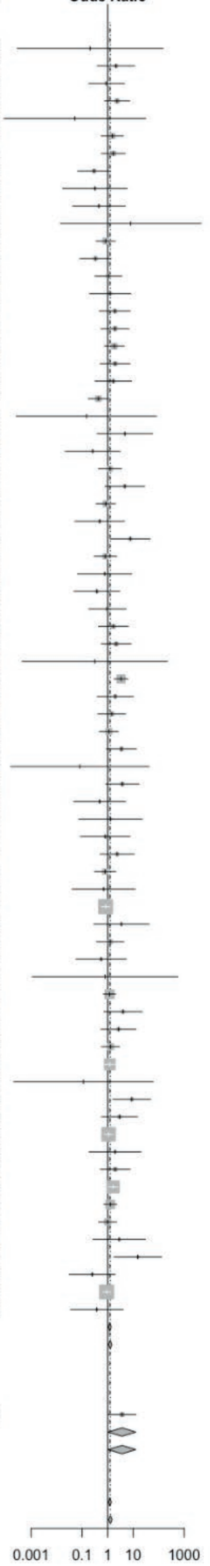

Study	TE	seTE	OR	95%-CI	Weight (fixed)	Weight (random)
ADJ = 0						
Huang C_JYH	-1.58	3.3510	0.21	[0.00; 146.02]	0.0%	0.0%
Mo P_ZH	0.75	0.8527	2.12	[0.40; 11.30]	0.5%	0.7%
Peng YD_WU	-0.09	0.8126	0.91	[0.19; 4.48]	0.5%	0.8%
Wang D_ZH	0.85	0.5793	2.35	[0.75; 7.31]	1.0%	1.4%
Young BE_MC	-2.96	3.2592	0.05	[0.00; 30.75]	0.0%	0.1%
Jin-Jin Z_MC	0.42	0.5067	1.52	[0.56; 4.11]	1.4%	1.8%
Wei-jie G_NHC	0.51	0.5415	1.66	[0.57; 4.80]	1.2%	1.6%
Lu Jiatao_WHH	-1.23	0.7475	0.29	[0.07; 1.27]	0.6%	0.9%
Qi Xiaolong_MC	-1.15	1.4793	0.32	[0.02; 5.78]	0.2%	0.2%
Chen Y_ multicentrico- FCMCH	-0.79	1.2072	0.45	[0.04; 4.82]	0.2%	0.4%
Chu J_TH	2.08	3.2342	7.97	[0.01; 4515.72]	0.0%	0.1%
Fan T_ RHWU	-0.18	0.4366	0.84	[0.36; 1.97]	1.8%	2.3%
Liu T_ UH	-1.10	0.7078	0.33	[0.08; 1.33]	0.7%	1.0%
Liu Y_ CHW	0.06	0.6120	1.06	[0.32; 3.53]	0.9%	1.3%
Liu Yo_SCH	0.23	0.9425	1.26	[0.20; 7.97]	0.4%	0.6%
Qi D_multicentrico	0.65	0.7087	1.91	[0.48; 7.68]	0.7%	1.0%
Shijiao Y_HHMU	0.66	0.6437	1.94	[0.55; 6.84]	0.9%	1.2%
Zhang G_ZHWU	0.61	0.4495	1.83	[0.76; 4.43]	1.7%	2.2%
Zhang H_CPHMC	0.68	0.6814	1.97	[0.52; 7.48]	0.8%	1.1%
Zhao W_SXH	0.51	0.8395	1.67	[0.32; 8.64]	0.5%	0.7%
Zhen L_MC	-0.84	0.4537	0.43	[0.18; 1.05]	1.7%	2.1%
Lei L_CUTGH	-1.92	3.2286	0.15	[0.00; 82.05]	0.0%	0.1%
Jing L_WUH	1.55	1.2764	4.73	[0.39; 57.69]	0.2%	0.3%
Chen G_TH	-1.35	1.2599	0.26	[0.02; 3.06]	0.2%	0.3%
Chen X_FHC/LCH	0.21	0.5261	1.23	[0.44; 3.44]	1.3%	1.7%
Feng Z_TXH	1.55	0.9138	4.69	[0.78; 28.13]	0.4%	0.6%
Li J_CHW	-0.17	0.4372	0.85	[0.36; 1.99]	1.8%	2.3%
Liu J_BDH	-0.72	1.1349	0.49	[0.05; 4.51]	0.3%	0.4%
Ma K_YCH	2.05	0.9103	7.75	[1.30; 46.15]	0.4%	0.6%
Lei P_BH (Multicéntrico)	-0.21	0.5102	0.81	[0.30; 2.21]	1.4%	1.8%
Qin X_SPH	-0.27	1.2444	0.76	[0.07; 8.76]	0.2%	0.3%
Wang G_PHTCC	-0.97	1.0509	0.38	[0.05; 2.97]	0.3%	0.5%
Wang Z_UH	-0.02	0.8542	0.98	[0.18; 5.22]	0.5%	0.7%
Yang L_YCPH	0.52	0.6848	1.68	[0.44; 6.42]	0.8%	1.1%
Zhang H_ZH	0.77	0.6820	2.17	[0.57; 8.26]	0.8%	1.1%
Zhao W_BYH	-1.17	3.3434	0.31	[0.00; 218.20]	0.0%	0.0%
Jin X_MC	1.21	0.3155	3.36	[1.81; 6.24]	3.5%	3.7%
Jiancheng L_JH	0.69	0.8253	1.99	[0.40; 10.05]	0.5%	0.7%
Wentao X_IDH	0.36	0.6294	1.44	[0.42; 4.94]	0.9%	1.2%
Zeng G_TPHS	0.11	0.4289	1.12	[0.48; 2.58]	1.9%	2.3%
CaiQ_TPHS	1.25	0.6876	3.48	[0.90; 13.40]	0.7%	1.0%
Cao M_SPHCC	-2.51	3.1859	0.08	[0.00; 41.78]	0.0%	0.1%
Gong Jiao_MC	1.32	0.7614	3.74	[0.84; 16.65]	0.6%	0.9%
Chao C_NFHJCH	-0.72	1.1874	0.48	[0.05; 4.97]	0.2%	0.4%
Lei S_RHZHTHC	0.25	1.4584	1.29	[0.07; 22.41]	0.2%	0.3%
FL_GHCTCPLA	-0.22	1.1362	0.80	[0.09; 7.46]	0.3%	0.4%
JX_WFPH	0.86	0.7757	2.36	[0.52; 10.78]	0.6%	0.8%
MY_multicenter 43 hosp	-0.23	0.4869	0.79	[0.31; 2.06]	1.5%	1.9%
Chen J_FAH	-0.37	1.4470	0.69	[0.04; 11.80]	0.2%	0.3%
Argenziano M_NYP/CUIMC	-0.19	0.1827	0.83	[0.58; 1.18]	10.6%	6.6%
Colaneri M_PSM	1.24	1.2669	3.47	[0.29; 41.53]	0.2%	0.3%
Xin L_CHWC/hospitales en Hunan	0.23	0.6226	1.25	[0.37; 4.25]	0.9%	1.3%
Ying S_hospitales en Beijing	-0.59	1.1535	0.56	[0.06; 5.33]	0.3%	0.4%
Chen W_YH	-0.24	3.3522	0.79	[0.00; 563.79]	0.0%	0.0%
Wang X_DFH	0.16	0.2821	1.18	[0.68; 2.05]	4.4%	4.2%
Dreher M_UHA	1.39	0.8740	4.00	[0.72; 22.18]	0.5%	0.7%
Xie H_WJH	0.98	0.8036	2.67	[0.55; 12.88]	0.5%	0.8%
Zhou M_MC	0.25	0.4106	1.29	[0.58; 2.88]	2.1%	2.5%
YuC_TH	0.20	0.2368	1.22	[0.76; 1.93]	6.3%	5.2%
Wang L_SPH	-2.18	3.2085	0.11	[0.00; 60.85]	0.0%	0.1%
Zhang R_RH	2.17	0.8668	8.80	[1.61; 48.12]	0.5%	0.7%
Yang J_WUH	1.07	0.8218	2.92	[0.58; 14.60]	0.5%	0.8%
Zhang L_WUH	0.08	0.1880	1.09	[0.75; 1.57]	10.0%	6.4%
Zheng X_FAH	0.67	1.1880	1.96	[0.19; 20.07]	0.2%	0.4%
Yan X_HNU	0.69	0.6807	1.99	[0.52; 7.55]	0.8%	1.1%
Zhang L_WUH	0.49	0.2113	1.63	[1.08; 2.47]	7.9%	5.8%
Bi Q_STPH	0.24	0.2966	1.27	[0.71; 2.27]	4.0%	4.0%
Xie J_UHW	-0.00	0.4139	1.00	[0.44; 2.24]	2.1%	2.5%
Jia M_RHWU	1.04	1.2061	2.82	[0.27; 30.02]	0.2%	0.4%
Wu J_TFAH	2.73	1.0884	15.27	[1.81; 128.94]	0.3%	0.4%
Zheng F_NHCFH	-1.39	1.0515	0.25	[0.03; 1.95]	0.3%	0.5%
LiX_TH	-0.10	0.1823	0.91	[0.64; 1.30]	10.6%	6.6%
Li Y_TH	-0.98	1.2076	0.38	[0.04; 4.00]	0.2%	0.4%
Fixed effect model			1.21	[1.08; 1.36]	99.1%	--
Random effects model			1.26	[1.09; 1.45]	--	98.8%
Heterogeneity: $I^2 = 14\%$, $\tau^2 = 0.0432$, $p = 0.17$						
ADJ = 1						
Ying W_ MC	1.29	0.6392	3.65	[1.04; 12.77]	0.9%	1.2%
Fixed effect model			3.65	[1.04; 12.77]	0.9%	--
Random effects model			3.65	[1.04; 12.77]	--	1.2%
Heterogeneity: not applicable						
**Fixed effect model**			**1.22**	**[1.09; 1.37]**	**100.0%**	**--**
**Random effects model**			**1.28**	**[1.10; 1.48]**	**--**	**100.0%**

Heterogeneity: $I^2 = 16\%$, $\tau^2 = 0.0500$, $p = 0.13$

Residual heterogeneity: $I^2 = 14\%$, $p = 0.17$

Candidate variable: Anorexia, outcome: severe Covid-19 disease, subgroup analysis: (crude vs adjusted)

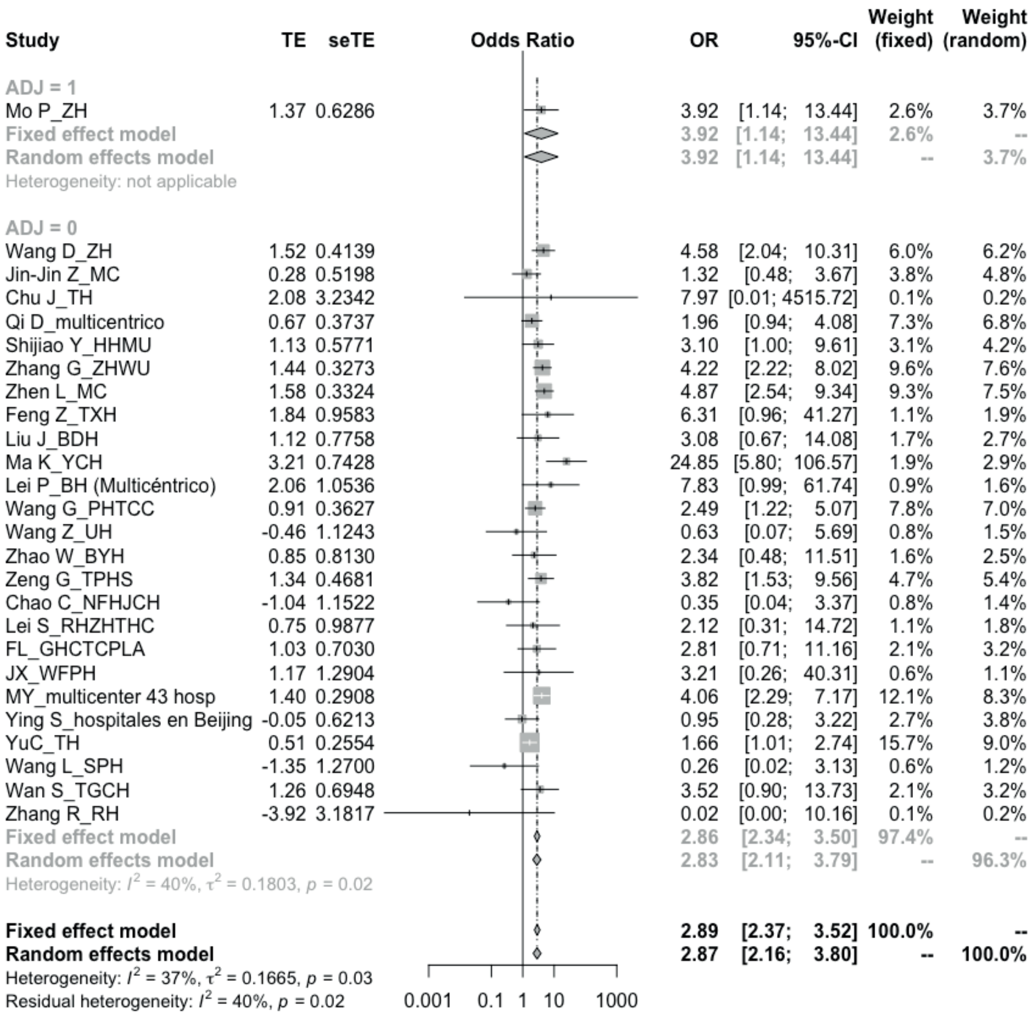

Candidate variable: Abdominal pain, outcome: severe Covid-19 disease

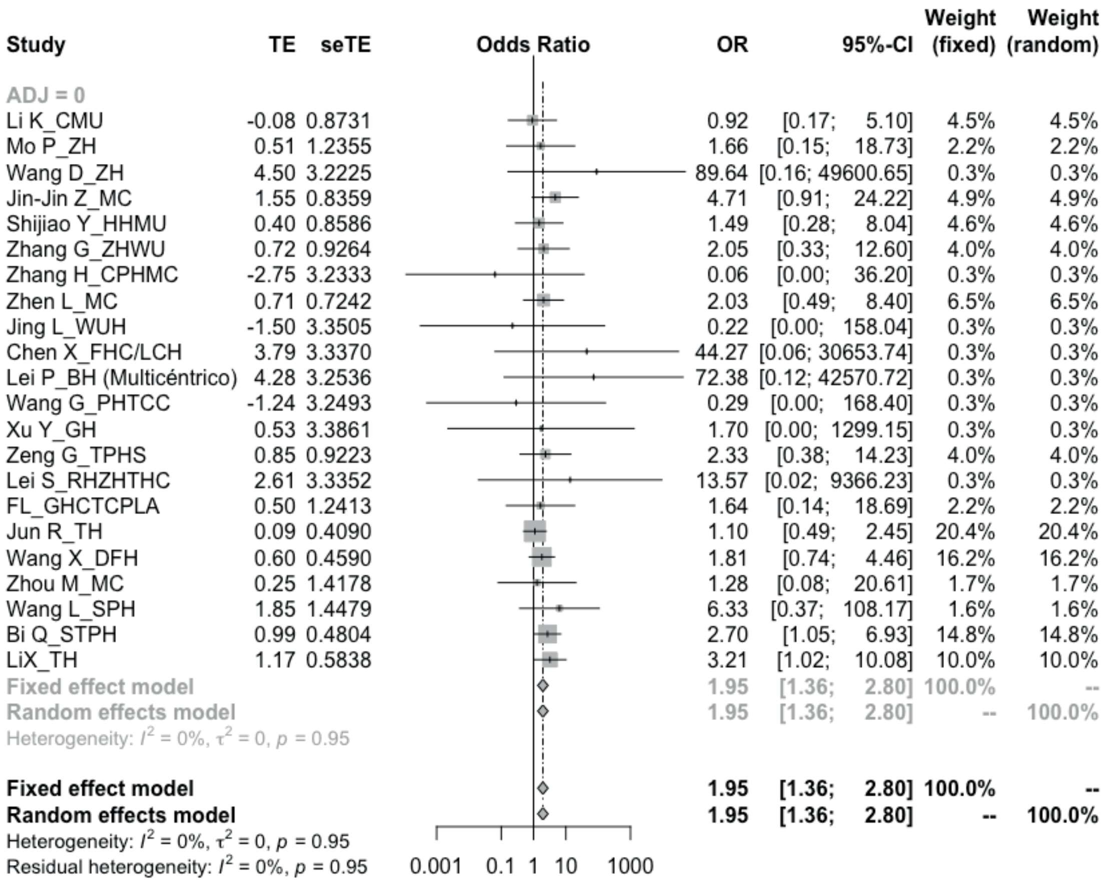

Candidate variable: Anemia, outcome: severe Covid-19 disease, subgroup analysis by risk of bias (high vs moderate/low)

Study	TE	seTE	OR	95%-CI	Weight (fixed)	Weight (random)
**MRB = 0**						
Zhen L_MC	0.69	0.3326	2.00	[1.04; 3.84]	4.2%	10.8%
Chen X_FHC/LCH	-0.24	0.5639	0.79	[0.26; 2.37]	1.5%	4.8%
Ma K_YCH	1.13	0.5352	3.10	[1.08; 8.84]	1.6%	5.3%
Hongying S_FAHWMU/SAHWMU	0.39	1.1561	1.47	[0.15; 14.17]	0.3%	1.3%
Chen W_YH	1.74	0.7698	5.70	[1.26; 25.77]	0.8%	2.8%
Jun R_TH	0.31	0.4725	1.36	[0.54; 3.44]	2.1%	6.4%
Zhou M_MC	-0.34	0.3052	0.71	[0.39; 1.29]	5.0%	12.1%
YuC_TH	0.25	0.1044	1.29	[1.05; 1.58]	42.7%	27.0%
Zheng F_NHCFH	-0.25	0.7968	0.78	[0.16; 3.71]	0.7%	2.6%
**Fixed effect model**			1.30	[1.09; 1.54]	59.0%	--
**Random effects model**			1.36	[0.96; 1.92]	--	73.1%
Heterogeneity: $I^2 = 39\%$, $\tau^2 = 0.0885$, $p = 0.11$						
**MRB = 1**						
Rentsch_CT	0.00	0.1066	1.00	[0.81; 1.23]	41.0%	26.9%
**Fixed effect model**			1.00	[0.81; 1.23]	41.0%	--
**Random effects model**			1.00	[0.81; 1.23]	--	26.9%
Heterogeneity: not applicable						
**Fixed effect model**			**1.17**	**[1.02; 1.33]**	**100.0%**	**--**
**Random effects model**			**1.24**	**[0.95; 1.61]**	**--**	**100.0%**
Heterogeneity: $I^2 = 46\%$, $\tau^2 = 0.0556$, $p = 0.05$						
Residual heterogeneity: $I^2 = 39\%$, $p = 0.11$						

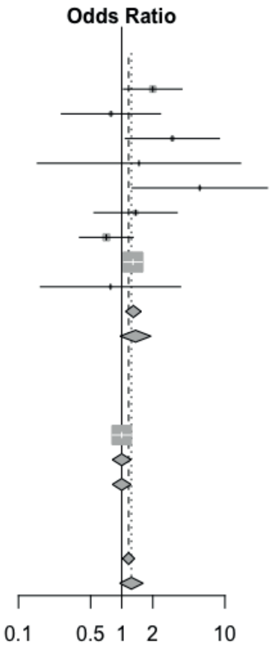

Candidate variable: High WBC (greater than 10.0 x 10^9/L), outcome: severe Covid-19 disease, subgroup analysis: (crude vs adjusted)

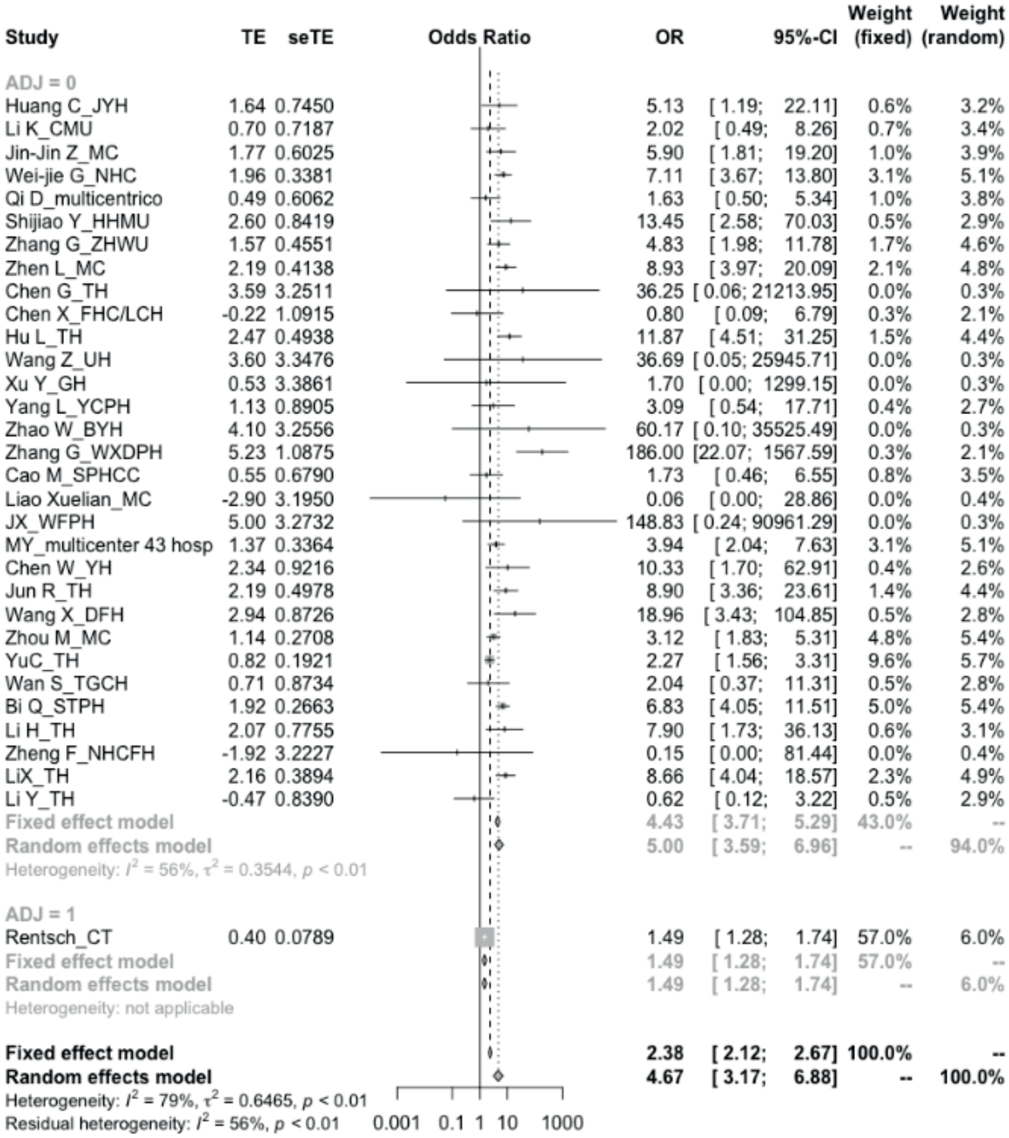

Candidate variable: High Neutrophil count (greater than 6.3 x 10^9/L), outcome: severe Covid-19 disease, subgroup analysis: (crude vs adjusted)

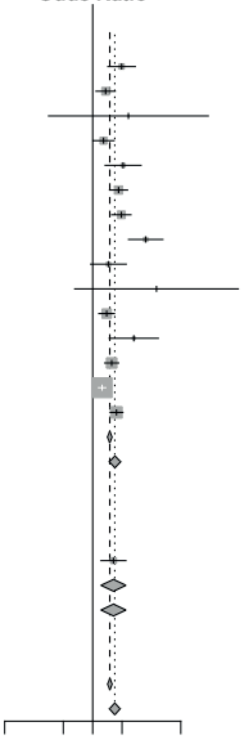

Study	TE	seTE	OR	95%-CI	(fixed)	(random)
ADJ = 0						
Li K_CMU	2.27	0.5524	9.68	[3.28; 28.58]	2.6%	6.2%
Duan Q_WPH	1.01	0.3839	2.75	[1.30; 5.84]	5.4%	7.9%
Liu T_ UH	2.80	3.1966	16.50	[0.03; 8678.46]	0.1%	0.4%
Qi D_multicentrico	0.85	0.4216	2.33	[1.02; 5.33]	4.5%	7.5%
Shijiao Y_HHMU	2.38	0.7208	10.83	[2.64; 44.50]	1.5%	4.8%
Zhen L_MC	2.03	0.3602	7.62	[3.76; 15.43]	6.2%	8.1%
Hu L_TH	2.24	0.3858	9.43	[4.43; 20.09]	5.4%	7.9%
Zhang G_WXDPH	4.16	0.6871	63.92	[16.63; 245.72]	1.7%	5.1%
Cao M_SPHCC	1.23	0.7162	3.44	[0.84; 13.99]	1.6%	4.9%
JX_WFPH	5.00	3.2732	148.83	[0.24; 90961.29]	0.1%	0.4%
MY_multicenter 43 hosp	1.09	0.3137	2.99	[1.61; 5.52]	8.1%	8.6%
Chen W_YH	3.23	0.9828	25.20	[3.67; 172.96]	0.8%	3.3%
Zhou M_MC	1.49	0.2806	4.43	[2.56; 7.68]	10.2%	8.9%
YuC_TH	0.74	0.1480	2.10	[1.57; 2.80]	36.5%	10.0%
LiX_TH	1.87	0.2563	6.46	[3.91; 10.67]	12.2%	9.2%
Fixed effect model			3.81	[3.19; 4.55]	96.8%	--
Random effects model			5.74	[3.67; 8.97]	--	93.3%
Heterogeneity: $I^2 = 77\%$, $\tau^2 = 0.4661$, $p < 0.01$						
ADJ = 1						
Jun R_TH	1.63	0.5013	5.09	[1.91; 13.61]	3.2%	6.7%
Fixed effect model			5.09	[1.91; 13.61]	3.2%	--
Random effects model			5.09	[1.91; 13.61]	--	6.7%
Heterogeneity: not applicable						
**Fixed effect model**			**3.84**	**[3.23; 4.58]**	**100.0%**	**--**
**Random effects model**			**5.66**	**[3.71; 8.63]**	**--**	**100.0%**
Heterogeneity: $I^2 = 75\%$, $\tau^2 = 0.4388$, $p < 0.01$						

Candidate variable: Neutrophil count increase (per 1 x 10^9 U/L), outcome: severe Covid-19 disease, subgroup analysis by risk of bias (high vs moderate/low)

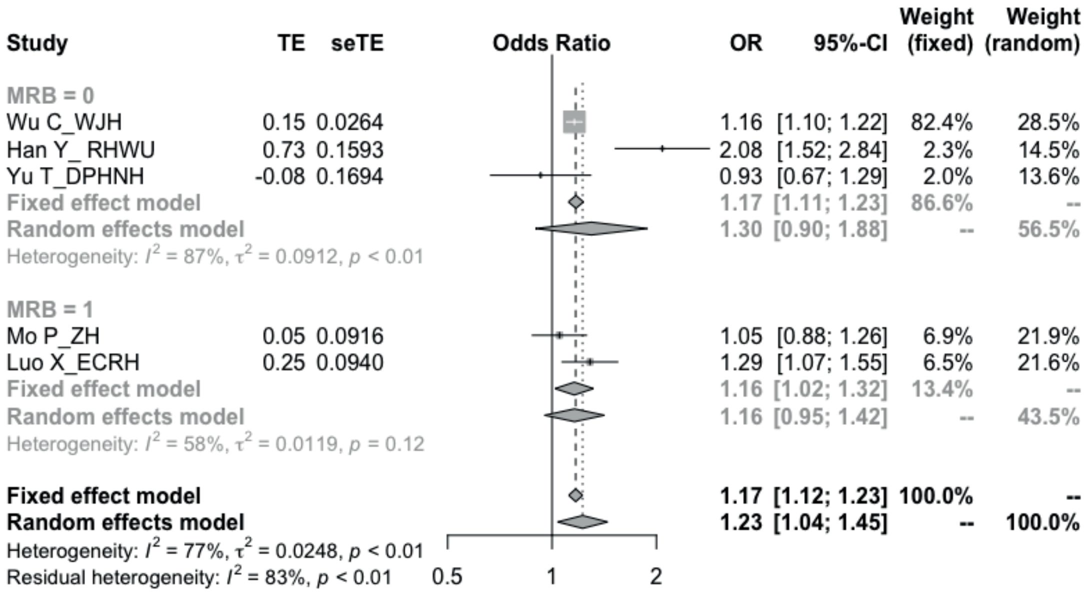

Candidate variable: Low neutrophil count (Less than 1.8 x 10^9/L), outcome: severe Covid-19 disease

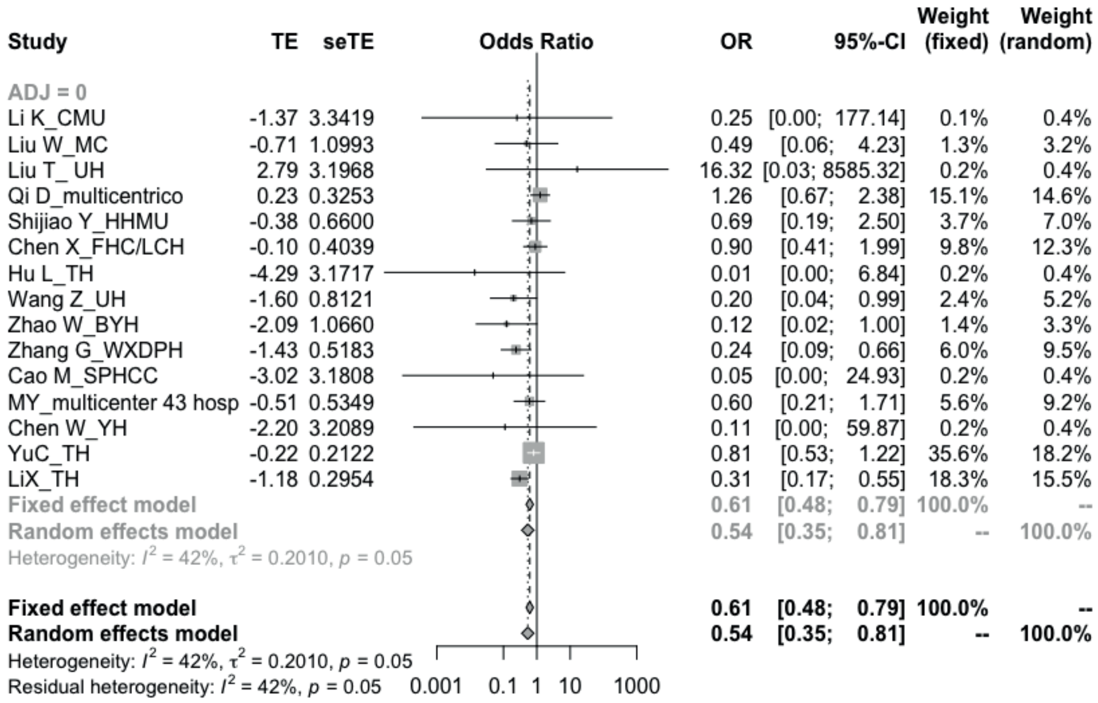

Candidate variable: Leukopenia (Less than 3.5-4 x 10^9/L), outcome: severe Covid-19 disease

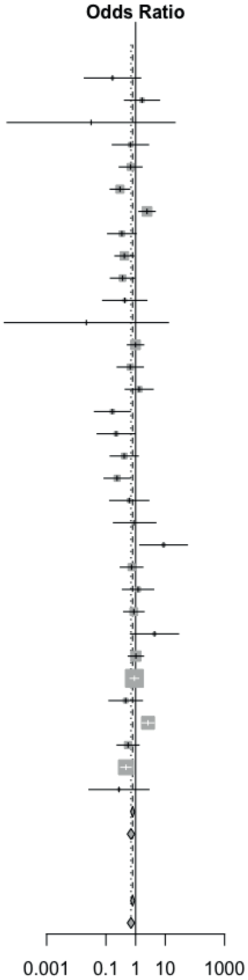

Study	TE	seTE	OR	95%-CI	Weight (fixed)	Weight (random)
ADJ = 0						
Huang C_JYH	-1.79	1.1180	0.17	[0.02; 1.49]	0.4%	1.3%
Li K_CMU	0.50	0.6953	1.65	[0.42; 6.45]	1.1%	2.5%
Liu F_XH	-3.46	3.3255	0.03	[0.00; 21.38]	0.0%	0.2%
Liu W_MC	-0.40	0.7177	0.67	[0.16; 2.74]	1.1%	2.4%
Jin-Jin Z_MC	-0.38	0.4512	0.68	[0.28; 1.65]	2.7%	3.7%
Wei-jie G_NHC	-1.21	0.3870	0.30	[0.14; 0.63]	3.7%	4.1%
Qi D_multicentrico	0.89	0.3226	2.44	[1.30; 4.59]	5.3%	4.5%
Shijiao Y_HHMU	-1.07	0.5661	0.34	[0.11; 1.04]	1.7%	3.1%
Zhang G_ZHWU	-0.87	0.3733	0.42	[0.20; 0.87]	4.0%	4.2%
Zhen L_MC	-1.01	0.4789	0.36	[0.14; 0.93]	2.4%	3.6%
Jing L_WUH	-0.84	0.8766	0.43	[0.08; 2.41]	0.7%	1.9%
Chen G_TH	-3.82	3.2524	0.02	[0.00; 12.88]	0.1%	0.2%
Chen X_FHC/LCH	-0.00	0.3237	1.00	[0.53; 1.88]	5.3%	4.5%
Hu L_TH	-0.41	0.5187	0.66	[0.24; 1.83]	2.1%	3.3%
Tabata S_SDFCH	0.28	0.5522	1.32	[0.45; 3.90]	1.8%	3.2%
Wang Z_UH	-1.80	0.7103	0.17	[0.04; 0.67]	1.1%	2.4%
Yang L_YCPH	-1.51	0.7537	0.22	[0.05; 0.97]	1.0%	2.3%
Zhao W_BYH	-0.88	0.5552	0.41	[0.14; 1.23]	1.8%	3.2%
Zhang G_WXDPH	-1.43	0.5183	0.24	[0.09; 0.66]	2.1%	3.3%
Cao M_SPHCC	-0.49	0.7755	0.61	[0.13; 2.80]	0.9%	2.2%
Liao Xuelian_MC	-0.07	0.8423	0.93	[0.18; 4.86]	0.8%	2.0%
JX_WFPH	2.17	0.9431	8.80	[1.39; 55.87]	0.6%	1.7%
MY_multicenter 43 hosp	-0.31	0.4473	0.73	[0.30; 1.76]	2.8%	3.8%
Colaneri M_PSM	0.19	0.6200	1.21	[0.36; 4.08]	1.4%	2.8%
Xin L_CHWC/hospitales en Hunan	-0.13	0.4033	0.87	[0.40; 1.93]	3.4%	4.0%
Chen W_YH	1.47	0.9535	4.36	[0.67; 28.24]	0.6%	1.7%
Wang X_DFH	0.02	0.3038	1.02	[0.57; 1.86]	6.0%	4.6%
YuC_TH	-0.09	0.1735	0.91	[0.65; 1.28]	18.4%	5.4%
Wan S_TGCH	-0.77	0.6628	0.46	[0.13; 1.70]	1.3%	2.6%
Bi Q_STPH	0.97	0.2416	2.63	[1.64; 4.22]	9.5%	5.0%
Zheng F_NHCFH	-0.59	0.4356	0.56	[0.24; 1.31]	2.9%	3.8%
LiX_TH	-0.75	0.2083	0.47	[0.31; 0.71]	12.7%	5.2%
Li Y_TH	-1.29	1.1899	0.28	[0.03; 2.83]	0.4%	1.2%
Fixed effect model			0.80	[0.69; 0.92]	100.0%	--
Random effects model			0.70	[0.53; 0.93]	--	100.0%
Heterogeneity: $I^2 = 65\%$, $\tau^2 = 0.3642$, $p < 0.01$						
**Fixed effect model**			**0.80**	**[0.69; 0.92]**	**100.0%**	**--**
**Random effects model**			**0.70**	**[0.53; 0.93]**	**--**	**100.0%**
Heterogeneity: $I^2 = 65\%$, $\tau^2 = 0.3642$, $p < 0.01$						
Residual heterogeneity: $I^2 = 65\%$, $p < 0.01$						

Candidate variable: Low Lymphocyte count (less than 0.8-1.5x 109/L), outcome: severe Covid-19 disease, subgroup analysis by risk of bias (high vs moderate/low)

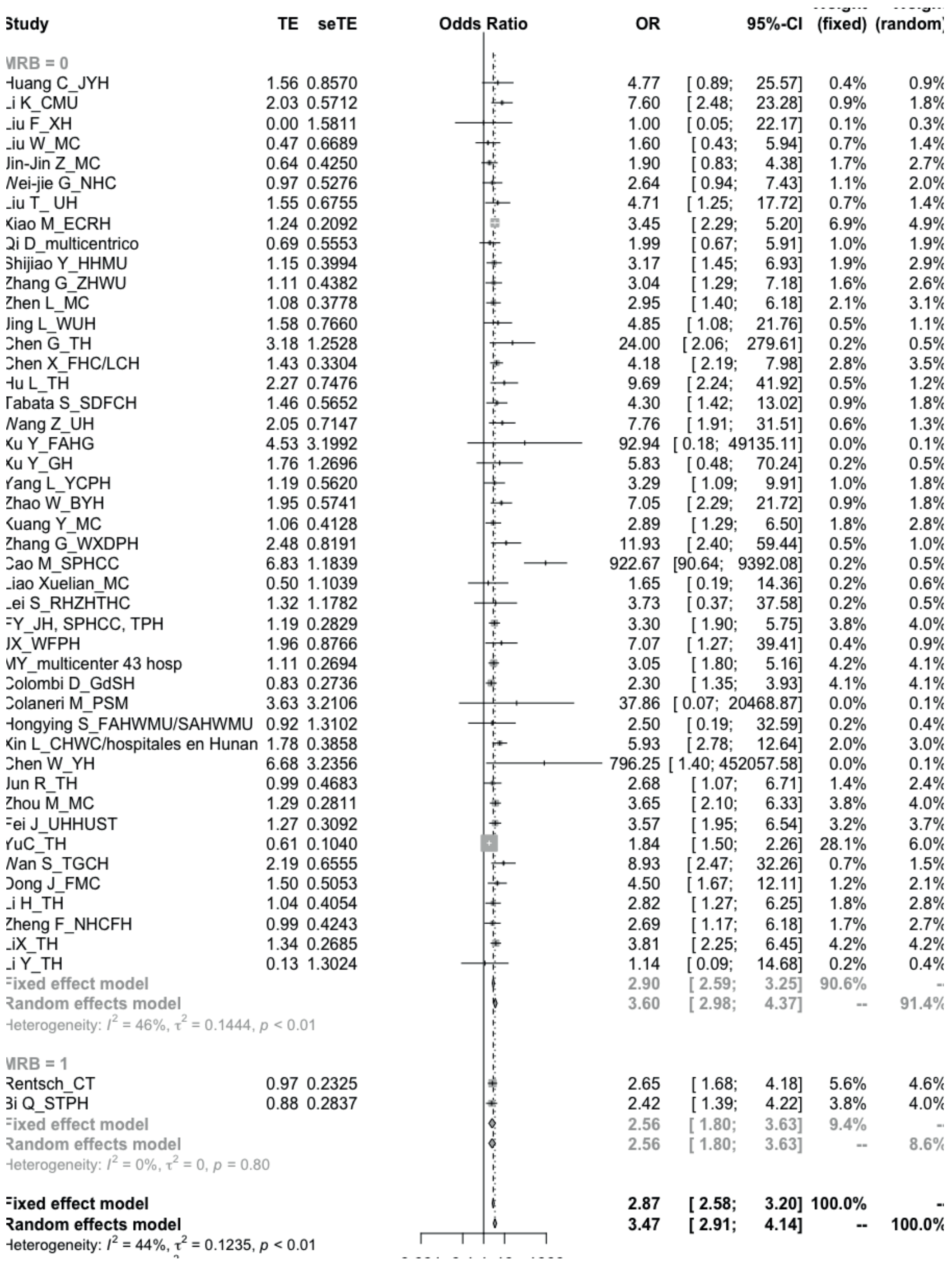

Candidate variable: Low platelet count (less than 100-150 x 109/L), outcome:severe Covid-19 disease, subgroup analysis by risk of bias (high vs moderate/low)

Study	TE	seTE	Odds Ratio	OR	95%-CI	Weight (fixed)	Weight (random)
MRB = 0							
Huang C_JYH	0.77	1.4566		2.17	[0.12; 37.64]	0.3%	0.7%
Liu W_MC	0.82	0.8937		2.26	[0.39; 13.03]	0.7%	1.6%
Wei-jie G_NHC	0.46	0.2733		1.58	[0.93; 2.70]	7.5%	6.1%
Liu T_ UH	3.58	3.1889		35.75	[0.07; 18520.52]	0.1%	0.1%
Xiao M_ECRH	1.08	0.3129		2.93	[1.59; 5.41]	5.7%	5.6%
Qi D_multicentrico	0.94	0.3382		2.55	[1.32; 4.96]	4.9%	5.2%
Shijiao Y_HHMU	1.44	0.6642		4.23	[1.15; 15.56]	1.3%	2.5%
Zhen L_MC	0.80	0.3655		2.23	[1.09; 4.57]	4.2%	4.9%
Jing L_WUH	0.37	0.9828		1.45	[0.21; 9.98]	0.6%	1.3%
Chen G_TH	-2.40	3.3634		0.09	[0.00; 66.31]	0.0%	0.1%
Chen X_FHC/LCH	0.47	0.3214		1.60	[0.85; 3.00]	5.4%	5.5%
Hu L_TH	1.15	0.6792		3.14	[0.83; 11.90]	1.2%	2.4%
Tabata S_SDFCH	-0.11	0.8482		0.90	[0.17; 4.73]	0.8%	1.7%
Yang L_YCPH	0.36	0.4395		1.43	[0.60; 3.38]	2.9%	4.1%
Zhao W_BYH	1.58	0.9541		4.85	[0.75; 31.49]	0.6%	1.4%
Zhang G_WXDPH	0.56	0.6490		1.76	[0.49; 6.28]	1.3%	2.5%
Cao M_SPHCC	0.88	0.5349		2.41	[0.84; 6.87]	2.0%	3.3%
Liao Xuelian_MC	2.12	0.8302		8.35	[1.64; 42.49]	0.8%	1.8%
JX_WFPH	1.88	1.4712		6.57	[0.37; 117.48]	0.3%	0.6%
Colaneri M_PSM	0.05	1.2046		1.05	[0.10; 11.13]	0.4%	0.9%
Hongying S_FAHWMU/SAHWMU	4.89	3.2236		132.73	[0.24; 73598.18]	0.1%	0.1%
Chen W_YH	4.29	3.3543		73.12	[0.10; 52386.57]	0.0%	0.1%
Jun R_TH	0.59	0.4642		1.80	[0.72; 4.47]	2.6%	3.9%
Zhou M_MC	1.00	0.2819		2.71	[1.56; 4.71]	7.1%	6.0%
YuC_TH	0.19	0.1851		1.21	[0.84; 1.74]	16.4%	7.2%
Wan S_TGCH	1.23	0.5499		3.42	[1.16; 10.06]	1.9%	3.2%
Zhou H_UH	1.07	0.4848		2.91	[1.12; 7.52]	2.4%	3.7%
Li H_TH	-0.41	0.5116		0.66	[0.24; 1.80]	2.1%	3.5%
Zheng F_NHCFH	0.54	0.7096		1.71	[0.43; 6.86]	1.1%	2.2%
LiX_TH	0.43	0.1910		1.53	[1.05; 2.23]	15.4%	7.2%
Fixed effect model				1.83	[1.57; 2.14]	89.9%	--
Random effects model				1.89	[1.59; 2.24]	--	89.4%
Heterogeneity: $I^2 = 8\%$, $\tau^2 = 0.0175$, $p = 0.34$							
MRB = 1							
Colombi D_GdSH	-1.06	0.3582		0.34	[0.17; 0.70]	4.4%	5.0%
Bi Q_STPH	1.41	0.3129		4.09	[2.21; 7.54]	5.7%	5.6%
Fixed effect model				1.40	[0.88; 2.22]	10.1%	--
Random effects model				1.19	[0.11; 13.47]	--	10.6%
Heterogeneity: $I^2 = 96\%$, $\tau^2 = 2.9430$, $p < 0.01$							
**Fixed effect model**				**1.78**	**[1.54; 2.06]**	**100.0%**	**--**
**Random effects model**				**1.93**	**[1.52; 2.46]**	**--**	**100.0%**
Heterogeneity: $I^2 = 48\%$, $\tau^2 = 0.1751$, $p < 0.01$							
Residual heterogeneity: $I^2 = 49\%$, $p < 0.01$							

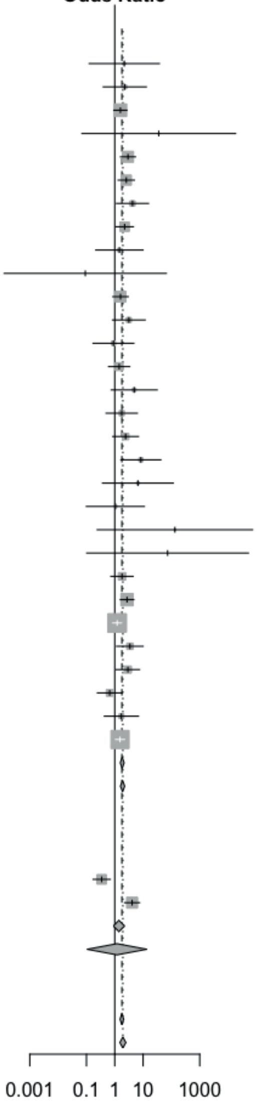

Candidate variable: High creatinine (more than 1.5 mg%), outcome: severe Covid-19 disease, subgroup analysis by risk of bias (high vs moderate/low)

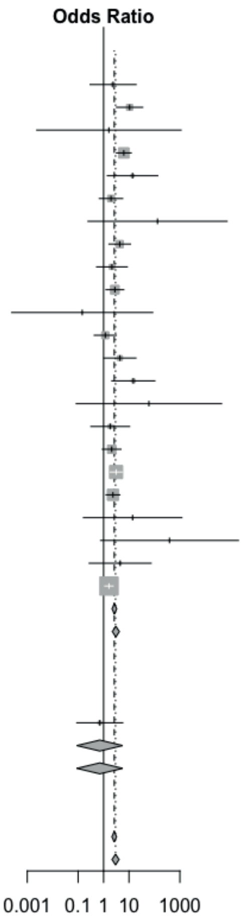

Study	TE	seTE	OR	95%-CI	Weight (fixed)	Weight (random)
MRB = 0						
Huang C_JYH	0.86	1.0627	2.36	[0.29; 18.97]	1.0%	1.9%
Wei-jie G_NHC	2.35	0.6046	10.53	[3.22; 34.45]	3.0%	4.8%
Liu T_ UH	0.48	3.3324	1.62	[0.00; 1110.37]	0.1%	0.2%
Xiao M_ECRH	1.83	0.3448	6.24	[3.17; 12.26]	9.1%	9.0%
Qi D_multicentrico	2.62	1.1658	13.79	[1.40; 135.47]	0.8%	1.7%
Shi W_ SPHCC	0.67	0.5388	1.96	[0.68; 5.63]	3.7%	5.6%
Shijiao Y_HHMU	4.88	3.2103	132.00	[0.24; 71312.19]	0.1%	0.2%
Zhen L_MC	1.46	0.4973	4.32	[1.63; 11.45]	4.4%	6.2%
Chen X_FHC/LCH	0.76	0.7083	2.13	[0.53; 8.55]	2.2%	3.8%
Yang L_YCPH	1.02	0.4113	2.78	[1.24; 6.22]	6.4%	7.6%
Zhao W_BYH	-1.93	3.2549	0.14	[0.00; 85.33]	0.1%	0.2%
Zhang G_WXDPH	0.15	0.5084	1.17	[0.43; 3.16]	4.2%	6.0%
Cao M_SPHCC	1.48	0.7381	4.39	[1.03; 18.66]	2.0%	3.6%
Liao Xuelian_MC	2.69	0.9953	14.79	[2.10; 104.00]	1.1%	2.2%
JX_WFPH	4.10	3.3578	60.43	[0.08; 43592.13]	0.1%	0.2%
Hongying S_FAHWMU/SAHWMU	0.60	0.8938	1.83	[0.32; 10.53]	1.4%	2.6%
Jun R_TH	0.74	0.4299	2.09	[0.90; 4.85]	5.8%	7.3%
Zhou M_MC	1.13	0.2746	3.10	[1.81; 5.31]	14.3%	10.7%
YuC_TH	0.85	0.3204	2.33	[1.25; 4.37]	10.5%	9.6%
Yang J_WUH	2.63	2.2726	13.87	[0.16; 1192.64]	0.2%	0.5%
Bi Q_STPH	5.97	3.1805	390.04	[0.77; 198780.31]	0.1%	0.2%
Zheng F_NHCFH	1.50	1.4290	4.48	[0.27; 73.78]	0.5%	1.1%
LiX_TH	0.49	0.1960	1.63	[1.11; 2.40]	28.1%	12.7%
Fixed effect model			2.66	[2.17; 3.26]	99.0%	--
Random effects model			3.03	[2.22; 4.14]	--	98.1%
Heterogeneity: $I^2 = 36\%$, $\tau^2 = 0.1542$, $p = 0.05$						
MRB = 1						
Liu W_MC	-0.34	1.0621	0.71	[0.09; 5.69]	1.0%	1.9%
Fixed effect model			0.71	[0.09; 5.69]	1.0%	--
Random effects model			0.71	[0.09; 5.69]	--	1.9%
Heterogeneity: not applicable						
**Fixed effect model**			**2.62**	**[2.14; 3.22]**	**100.0%**	**--**
**Random effects model**			**2.95**	**[2.16; 4.03]**	**--**	**100.0%**

Heterogeneity: $I^2 = 36\%$, $\tau^2 = 0.1588$, $p = 0.04$

Residual heterogeneity: $I^2 = 36\%$, $p = 0.05$

Candidate variable: Creatinine increase (per 0.1 mg%), outcome: severe Covid-19 disease, subgroup analysis: (crude vs adjusted)

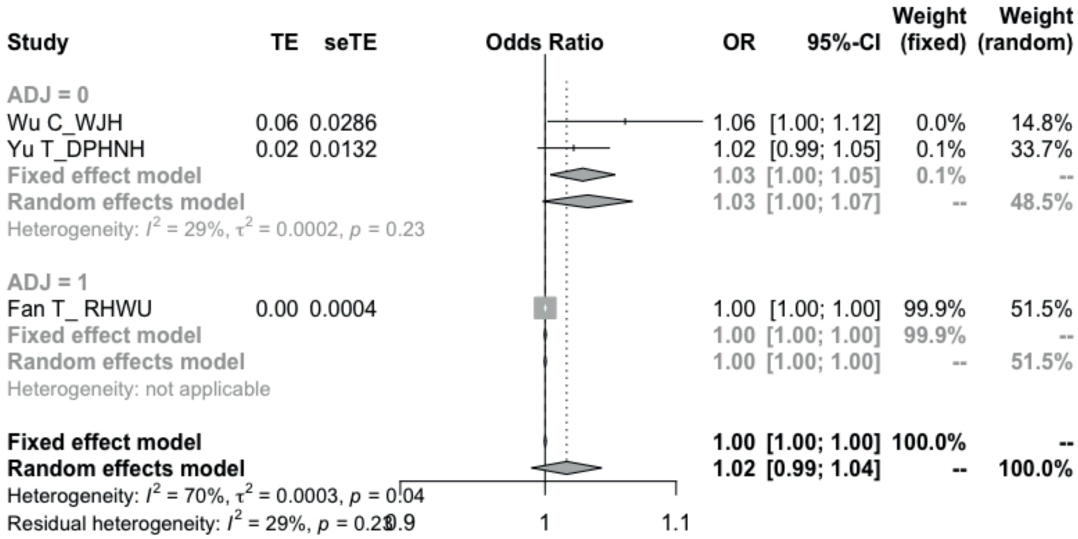

Candidate variable: Acute kidney injury, outcome: severe Covid-19 disease

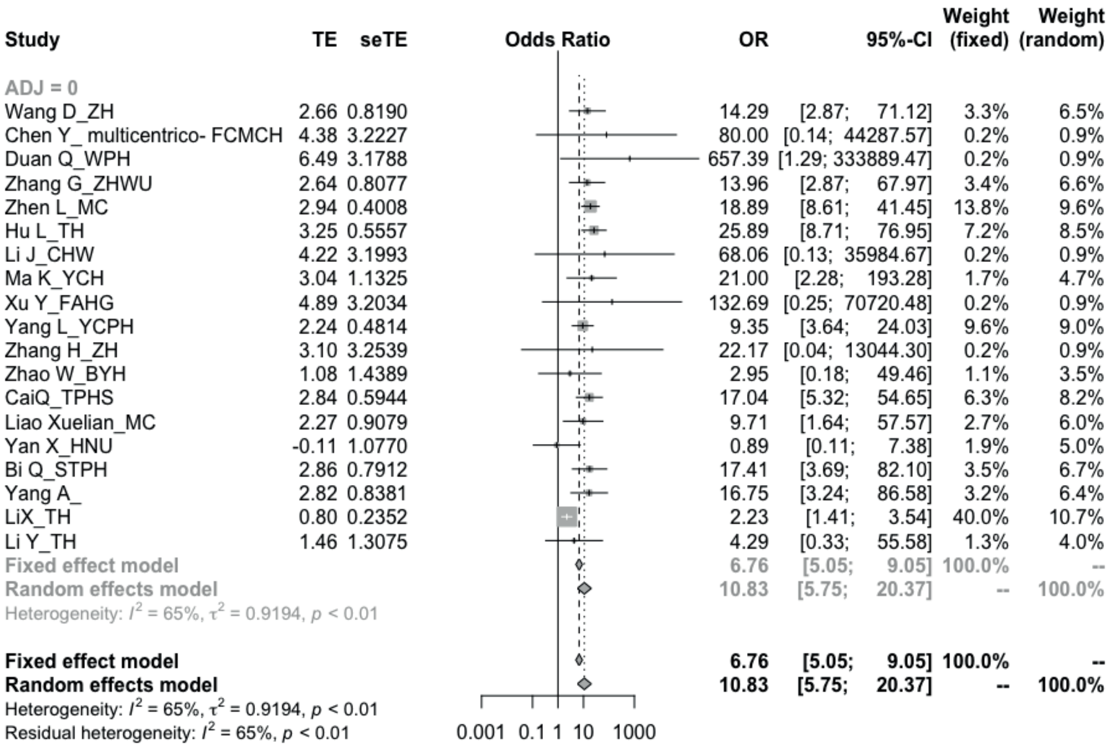

Candidate variable: High BUN (more than 5.2-9.5 mmol/L), outcome: severe Covid-19 disease, subgroup analysis: (crude vs adjusted)

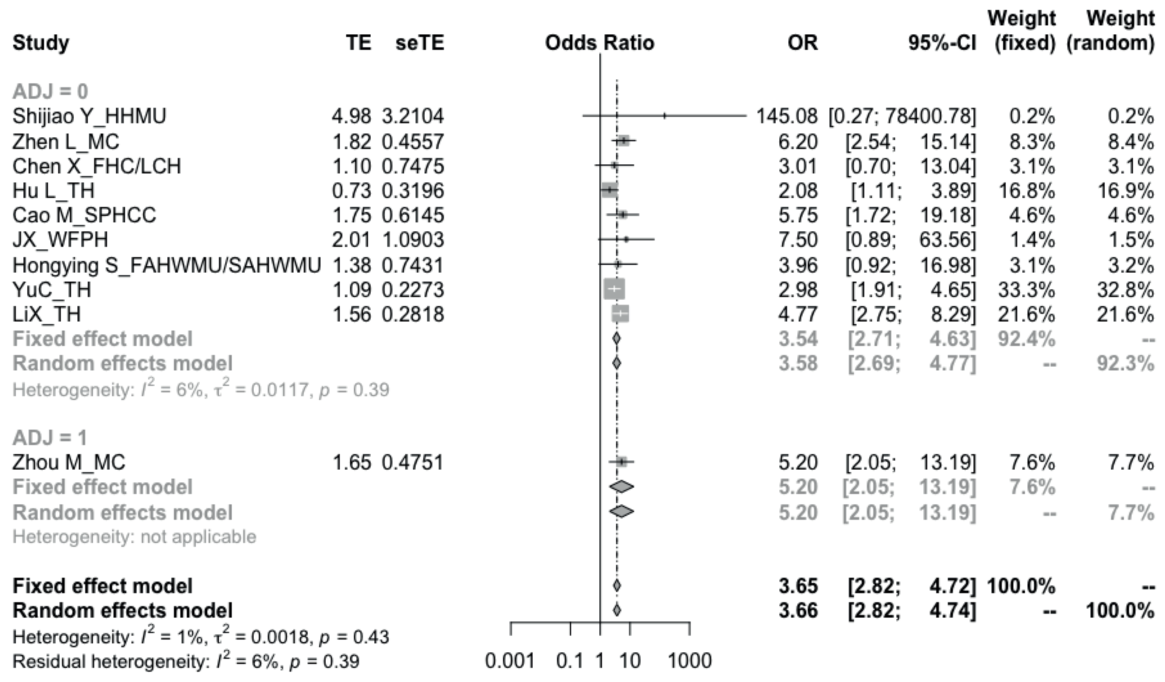

Candidate variable: High LDH (more than 240-250 U/L), outcome: severe Covid-19 disease, subgroup analysis by risk of bias (high vs moderate/low)

Study	TE	seTE	OR	95%-CI	Weight (fixed)	Weight (random)
**MRB = 0**						
Huang C_JYH	1.95	1.1145	7.06	[0.79; 62.72]	0.4%	1.7%
Wei-jie G_NHC	1.32	0.3404	3.73	[1.92; 7.27]	4.4%	5.2%
Duan Q_WPH	1.00	0.4156	2.71	[1.20; 6.12]	2.9%	4.7%
Liu T_ UH	2.93	1.0788	18.75	[2.26; 155.34]	0.4%	1.8%
Qi D_multicentrico	0.94	0.3436	2.57	[1.31; 5.03]	4.3%	5.2%
Zhen L_MC	1.46	0.5089	4.30	[1.59; 11.66]	2.0%	4.1%
Chen X_FHC/LCH	2.32	0.3669	10.20	[4.97; 20.94]	3.8%	5.0%
Hu L_TH	1.77	0.8641	5.88	[1.08; 32.01]	0.7%	2.4%
Tabata S_SDFCH	0.66	0.4810	1.93	[0.75; 4.95]	2.2%	4.3%
Wang Z_UH	2.43	0.8343	11.33	[2.21; 58.15]	0.7%	2.5%
Xu Y_FAHG	1.49	0.7446	4.45	[1.03; 19.16]	0.9%	2.9%
Yang L_YCPH	2.23	0.5232	9.31	[3.34; 25.95]	1.9%	4.0%
Kuang Y_MC	2.11	0.5116	8.29	[3.04; 22.59]	1.9%	4.1%
CaiQ_TPHS	2.67	0.5046	14.51	[5.40; 39.02]	2.0%	4.2%
MY_multicenter 43 hosp	0.66	0.2867	1.94	[1.11; 3.41]	6.2%	5.5%
Hongying S_FAHWMU/SAHWMU	3.06	1.0820	21.38	[2.56; 178.19]	0.4%	1.8%
Zhou M_MC	1.19	0.4710	3.29	[1.31; 8.28]	2.3%	4.4%
YuC_TH	0.35	0.1252	1.42	[1.11; 1.81]	32.4%	6.3%
Zhang R_RH	0.92	0.5809	2.50	[0.80; 7.81]	1.5%	3.7%
Zhang L_WUH	1.12	0.2155	3.05	[2.00; 4.65]	10.9%	5.9%
Zheng F_NHCFH	1.55	0.4314	4.70	[2.02; 10.94]	2.7%	4.6%
LiX_TH	1.62	0.2277	5.06	[3.24; 7.91]	9.8%	5.9%
Li Y_TH	0.69	0.8660	2.00	[0.37; 10.92]	0.7%	2.4%
**Fixed effect model**			2.83	[2.46; 3.27]	95.4%	--
**Random effects model**			4.22	[3.02; 5.90]	--	92.7%
Heterogeneity: I^2 = 74%, τ^2 = 0.4021, p < 0.01						
**MRB = 1**						
Huang H_GEPH	5.39	1.2399	219.60	[19.33; 2494.65]	0.3%	1.5%
Colombi D_GdSH	1.10	0.3494	3.00	[1.51; 5.95]	4.2%	5.1%
Dong J_FMC	3.38	1.8722	29.35	[0.75; 1151.35]	0.1%	0.7%
**Fixed effect model**			4.37	[2.29; 8.37]	4.6%	--
**Random effects model**			22.51	[1.04; 487.75]	--	7.3%
Heterogeneity: I^2 = 84%, τ^2 = 5.9142, p < 0.01						
**Fixed effect model**			**2.89**	**[2.51; 3.32]**	**100.0%**	**--**
**Random effects model**			**4.48**	**[3.21; 6.25]**	**--**	**100.0%**
Heterogeneity: I^2 = 75%, τ^2 = 0.4420, p < 0.01						
Residual heterogeneity: I^2 = 76%, p < 0.01						

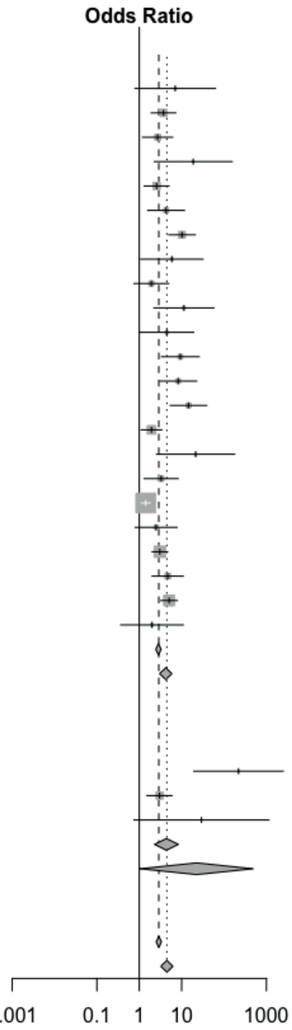

Candidate variable: LDH increase (per 1 U/L), outcome: severe Covid-19 disease, subgroup analysis by risk of bias (high vs moderate/low)

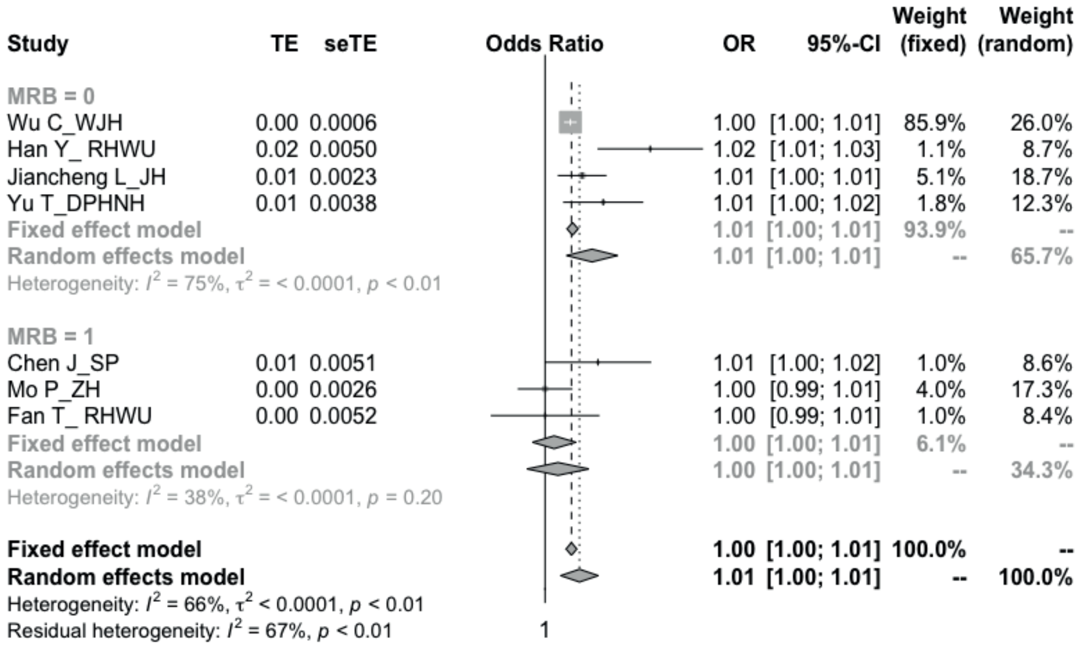

Candidate variable: High CK (more than 185-200 U/L), outcome: severe Covid-19 disease

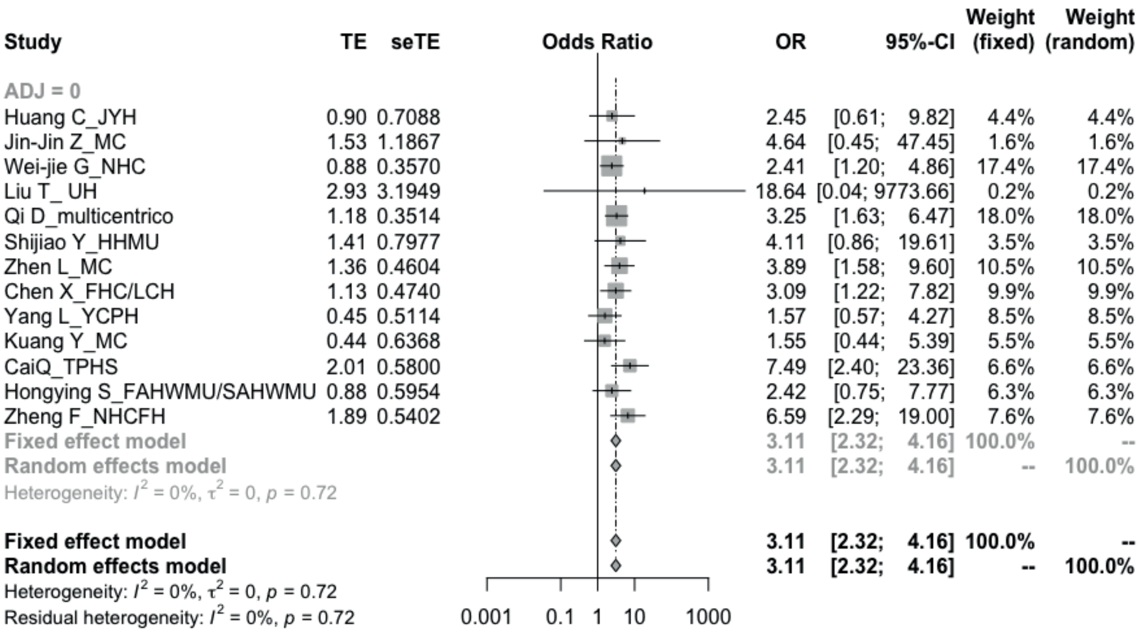

Candidate variable: Myocardial injury, outcome: severe Covid-19 disease, subgroup analysis by risk of bias (high vs moderate/low)

Study	TE	seTE	OR	95%-CI	Weight (fixed)	Weight (random)
MRB = 0						
Huang C_JYH	2.48	1.1824	12.00	[1.18; 121.80]	2.0%	2.5%
Chen Y_ multicentrico- FCMCH	4.97	3.2134	144.22	[0.27; 78387.81]	0.3%	0.4%
Qi D_multicentrico	4.90	3.2203	133.89	[0.24; 73770.73]	0.3%	0.4%
Zhang G_ZHWU	4.21	1.0460	67.69	[8.71; 525.91]	2.5%	3.1%
Zhen L_MC	2.32	0.5315	10.16	[3.59; 28.80]	9.7%	9.3%
Chen X_FHC/LCH	2.47	0.5738	11.80	[3.83; 36.33]	8.3%	8.4%
Hu L_TH	1.27	0.6339	3.56	[1.03; 12.34]	6.8%	7.2%
Ma K_YCH	1.82	0.5810	6.14	[1.97; 19.18]	8.1%	8.2%
Xu Y_FAHG	5.51	3.2001	247.50	[0.47; 131070.34]	0.3%	0.4%
Yang L_YCPH	3.57	0.5866	35.57	[11.27; 112.31]	7.9%	8.1%
Zhang H_ZH	5.20	3.1887	181.67	[0.35; 94073.77]	0.3%	0.4%
Zhao W_BYH	2.70	0.8590	14.81	[2.75; 79.74]	3.7%	4.4%
CaiQ_TPHS	2.80	0.5424	16.40	[5.66; 47.47]	9.3%	9.1%
Cao M_SPHCC	2.42	0.5424	11.22	[3.87; 32.47]	9.3%	9.1%
Hongying S_FAHWMU/SAHWMU	1.95	1.2455	7.04	[0.61; 80.86]	1.8%	2.2%
Zhou M_MC	1.88	0.3936	6.58	[3.04; 14.23]	17.7%	13.7%
HanH_RH	0.89	0.6801	2.44	[0.64; 9.24]	5.9%	6.5%
Bi Q_STPH	4.96	3.2047	143.29	[0.27; 76576.90]	0.3%	0.4%
Li Y_TH	3.77	3.2759	43.43	[0.07; 26680.58]	0.3%	0.3%
Fixed effect model			10.07	[7.21; 14.05]	94.5%	--
Random effects model			10.46	[7.03; 15.58]	--	93.9%
Heterogeneity: $I^2 = 19\%$, $\tau^2 = 0.1332$, $p = 0.23$						
MRB = 1						
Duan Q_WPH	1.66	0.7044	5.26	[1.32; 20.92]	5.5%	6.1%
Fixed effect model			5.26	[1.32; 20.92]	5.5%	--
Random effects model			5.26	[1.32; 20.92]	--	6.1%
Heterogeneity: not applicable						
**Fixed effect model**			**9.72**	**[7.03; 13.43]**	**100.0%**	**--**
**Random effects model**			**10.00**	**[6.84; 14.62]**	**--**	**100.0%**
Heterogeneity: $I^2 = 17\%$, $\tau^2 = 0.1189$, $p = 0.24$						
Residual heterogeneity: $I^2 = 19\%$, $p = 0.23$						

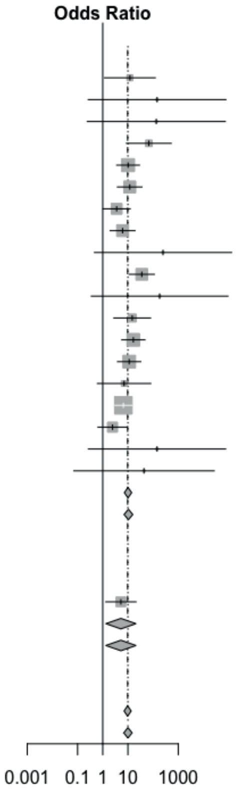

Candidate variable: High CK-MB (more than 25 U/L), outcome: severe Covid-19 disease, subgroup analysis: (crude vs adjusted)

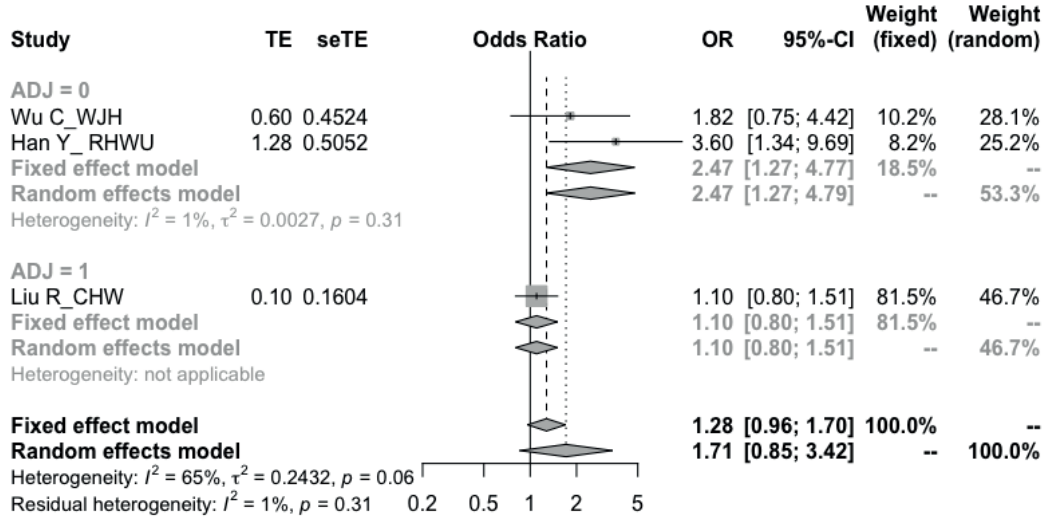

Candidate variable: High BNP (more than 500-900 pg/mL), outcome: severe Covid-19 disease

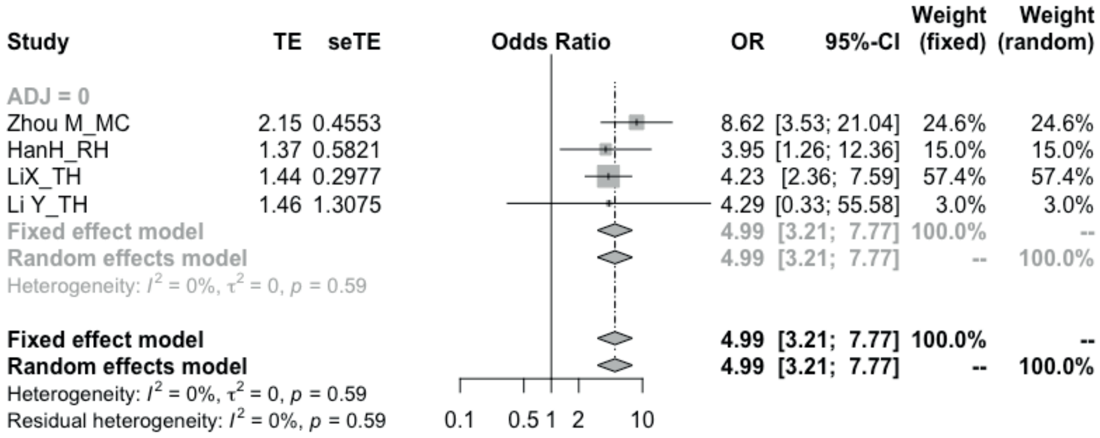

Candidate variable: Urea increase (per 1 mmol/L), outcome: severe Covid-19 disease

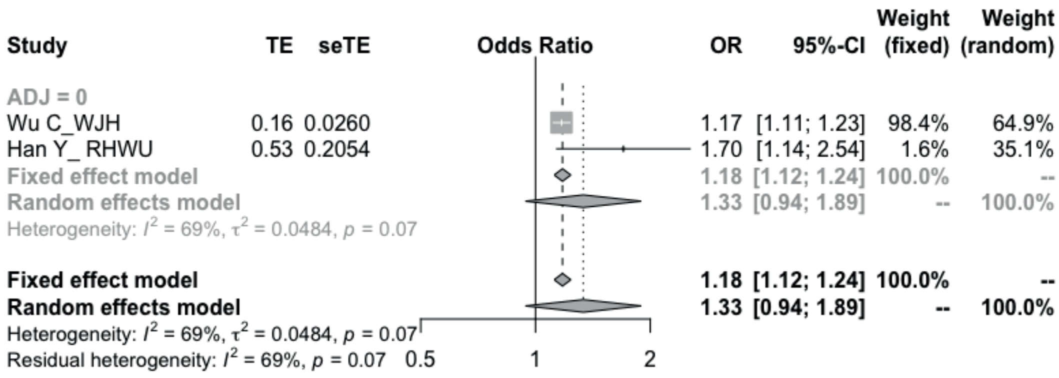

Candidate variable: High D-dimer (more than 500-1000 ng/ml), outcome: severe Covid-19 disease, subgroup analysis by risk of bias (high vs moderate/low)

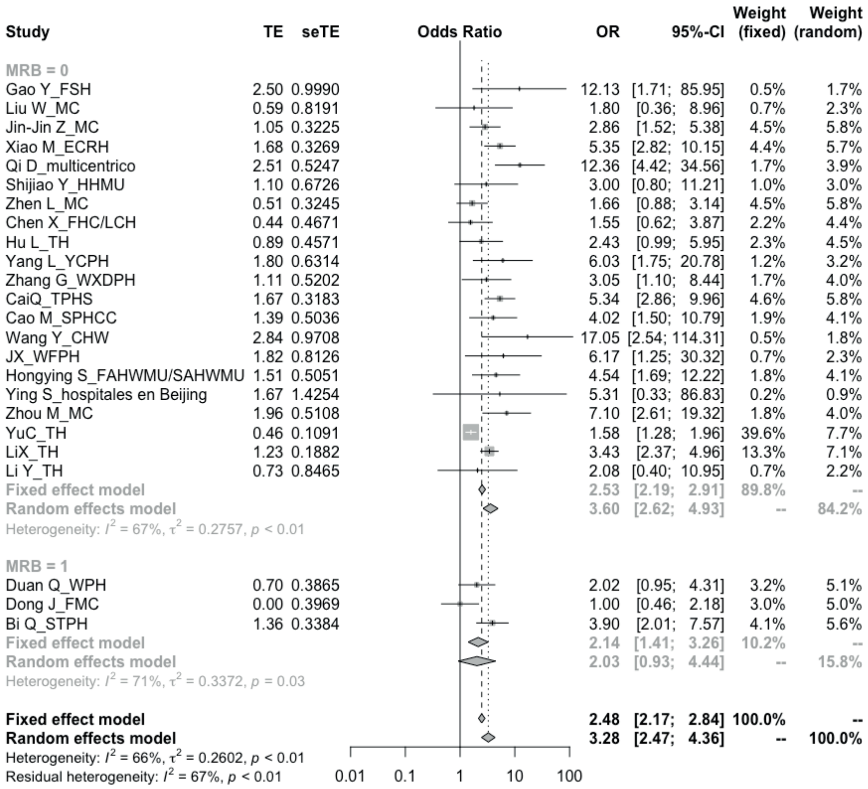

Candidate variable: Prolonged PT, outcome: severe Covid-19 disease

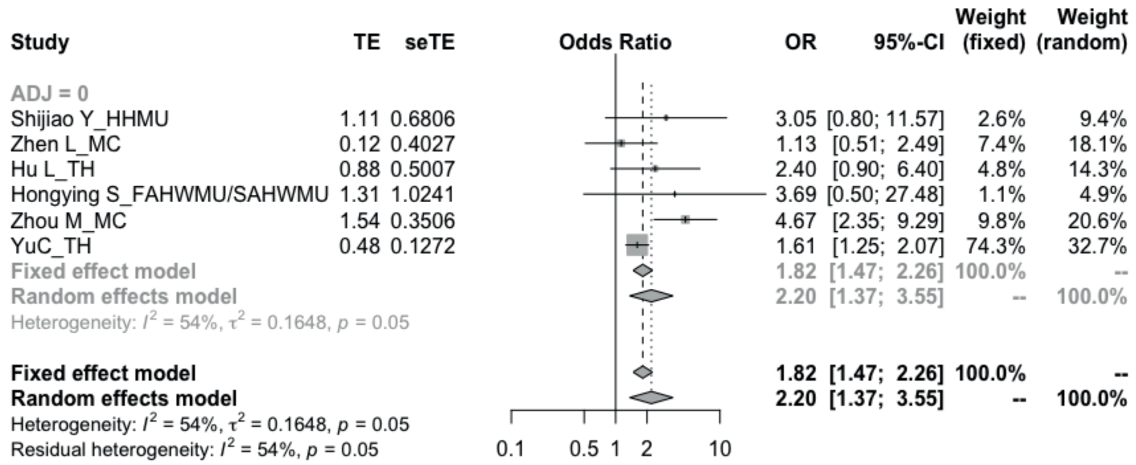

Candidate variable: Increase PT (per 1 second), outcome: severe Covid-19 disease

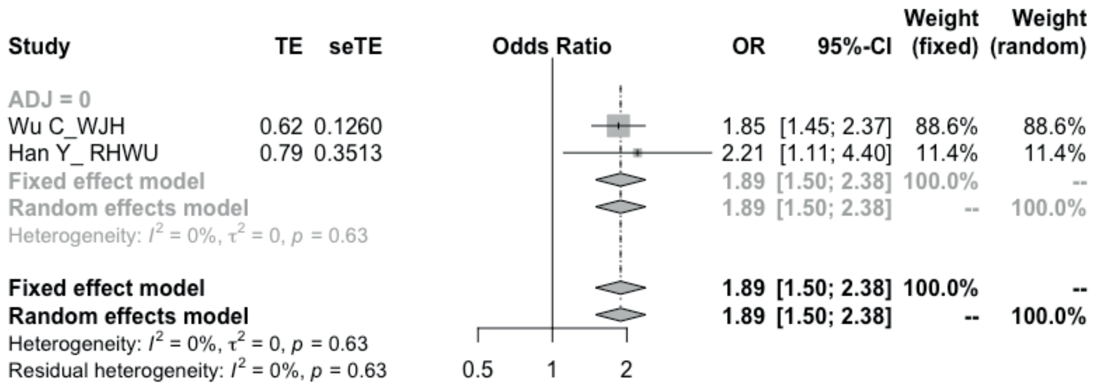

Candidate variable: Prolonged APTT time, outcome: severe Covid-19 disease

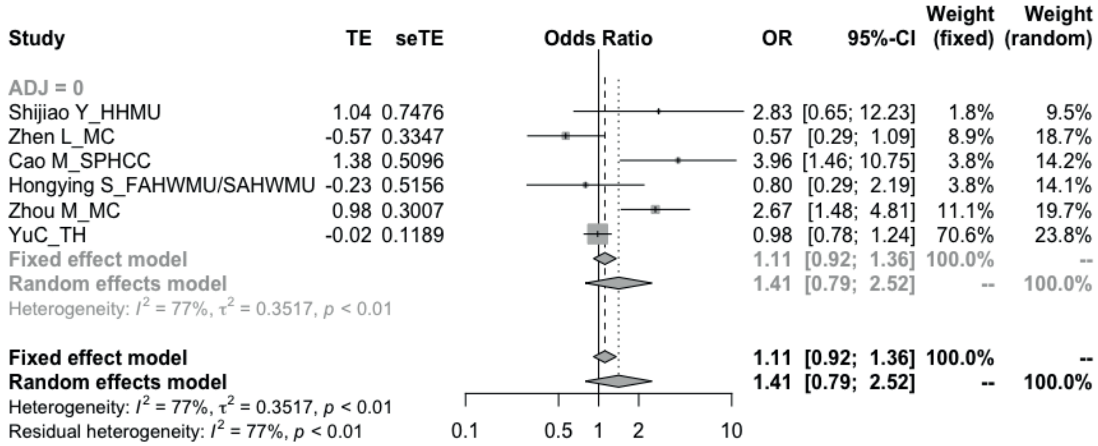

Candidate variable: Increase APTT time (per 1 second), outcome: severe Covid-19 disease

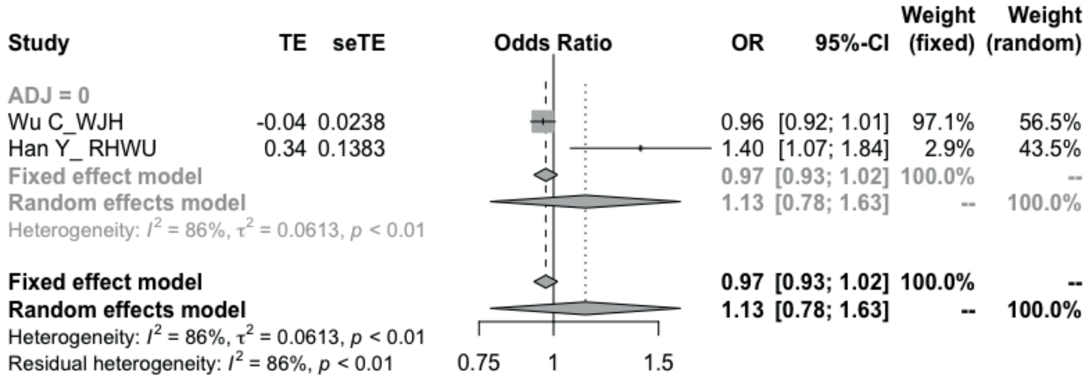

Candidate variable: High ferritin (more than 300-500 ng/mL), outcome: severe Covid-19 disease

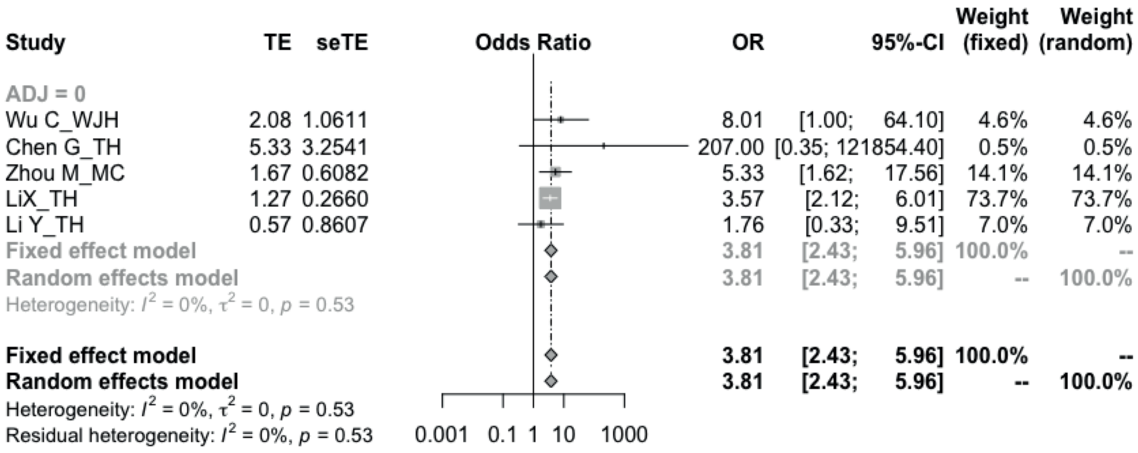

Candidate variable: High CRP (More than 1-100 mg/l) outcome: severe Covid-19 disease, subgroup analysis by risk of bias (high vs moderate/low)

Study	TE	seTE	OR	95%-CI	Weight (fixed)	Weight (random)
MRB = 0						
Wu C_WJH	2.86	2.0499	17.40	[0.31; 967.09]	0.1%	0.7%
Li K_CMU	2.58	0.7828	13.20	[2.85; 61.24]	0.9%	2.5%
Liu F_XH	0.98	1.4433	2.67	[0.16; 45.14]	0.3%	1.2%
Jin-Jin Z_MC	1.20	0.8024	3.31	[0.69; 15.96]	0.9%	2.5%
Wei-jie G_NHC	1.97	0.5291	7.17	[2.54; 20.24]	2.0%	3.5%
Liu Y_ CHW	4.08	1.1031	59.00	[6.79; 512.67]	0.5%	1.7%
Xiao M_ECRH	2.09	0.4192	8.12	[3.57; 18.46]	3.2%	3.9%
Qi D_multicentrico	2.79	0.3872	16.31	[7.64; 34.84]	3.7%	4.0%
Shijiao Y_HHMU	1.80	0.6579	6.04	[1.66; 21.94]	1.3%	3.0%
Zhen L_MC	-0.22	1.2376	0.80	[0.07; 9.05]	0.4%	1.5%
Chen G_TH	5.38	3.2547	216.33	[0.37; 127516.02]	0.1%	0.3%
Chen X_FHC/LCH	0.91	0.3296	2.49	[1.30; 4.75]	5.1%	4.3%
Hu L_TH	1.05	0.3393	2.86	[1.47; 5.56]	4.8%	4.2%
Tabata S_SDFCH	0.96	0.4658	2.61	[1.05; 6.50]	2.6%	3.7%
Wang Z_UH	1.96	1.0831	7.10	[0.85; 59.29]	0.5%	1.8%
Xu Y_GH	3.44	3.2277	31.17	[0.06; 17427.85]	0.1%	0.3%
Yang L_YCPH	5.03	3.1718	152.51	[0.30; 76400.58]	0.1%	0.3%
Zhao W_BYH	1.95	0.7917	7.03	[1.49; 33.18]	0.9%	2.5%
Kuang Y_MC	0.54	0.4044	1.71	[0.78; 3.78]	3.4%	4.0%
Cao M_SPHCC	-1.25	0.7227	0.29	[0.07; 1.18]	1.1%	2.7%
FL_GHCTCPLA	1.99	0.7701	7.31	[1.62; 33.07]	0.9%	2.6%
JX_WFPH	1.96	0.8766	7.07	[1.27; 39.41]	0.7%	2.3%
MY_multicenter 43 hosp	1.22	0.2704	3.39	[2.00; 5.77]	7.6%	4.5%
Hongying S_FAHWMU/SAHWMU	2.91	1.0348	18.28	[2.40; 138.92]	0.5%	1.9%
Chen W_YH	1.43	0.7326	4.17	[0.99; 17.51]	1.0%	2.7%
Jun R_TH	1.10	0.5404	3.00	[1.04; 8.66]	1.9%	3.4%
Zhou M_MC	1.33	0.4941	3.79	[1.44; 9.98]	2.3%	3.6%
YuC_TH	0.14	0.1320	1.15	[0.89; 1.49]	31.9%	4.9%
Li H_TH	1.87	0.5271	6.50	[2.31; 18.26]	2.0%	3.5%
Zheng F_NHCFH	4.88	3.1732	131.54	[0.26; 66078.61]	0.1%	0.3%
LiX_TH	1.86	0.3172	6.41	[3.44; 11.94]	5.5%	4.3%
Li Y_TH	1.00	0.9470	2.72	[0.43; 17.42]	0.6%	2.1%
Fixed effect model			2.67	[2.28; 3.12]	86.7%	--
Random effects model			4.46	[3.00; 6.64]	--	84.5%
Heterogeneity: $I^2 = 75\%$, $\tau^2 = 0.7269$, $p < 0.01$						
MRB = 1						
Liu W_MC	2.35	0.8532	10.53	[1.98; 56.07]	0.8%	2.3%
Huang H_GEPH	2.42	1.0054	11.28	[1.57; 80.93]	0.5%	1.9%
Chao C_NFHJCH	0.38	0.7564	1.46	[0.33; 6.43]	1.0%	2.6%
Colombi D_GdSH	1.03	0.3220	2.80	[1.49; 5.26]	5.4%	4.3%
Bi Q_STPH	1.63	0.3144	5.08	[2.75; 9.41]	5.6%	4.3%
Fixed effect model			3.93	[2.63; 5.87]	13.3%	--
Random effects model			4.04	[2.31; 7.05]	--	15.5%
Heterogeneity: $I^2 = 33\%$, $\tau^2 = 0.1248$, $p = 0.20$						
**Fixed effect model**			**2.81**	**[2.43; 3.25]**	**100.0%**	**--**
**Random effects model**			**4.40**	**[3.10; 6.23]**	**--**	**100.0%**
Heterogeneity: $I^2 = 73\%$, $\tau^2 = 0.6316$, $p < 0.01$						
Residual heterogeneity: $I^2 = 73\%$, $p < 0.01$						

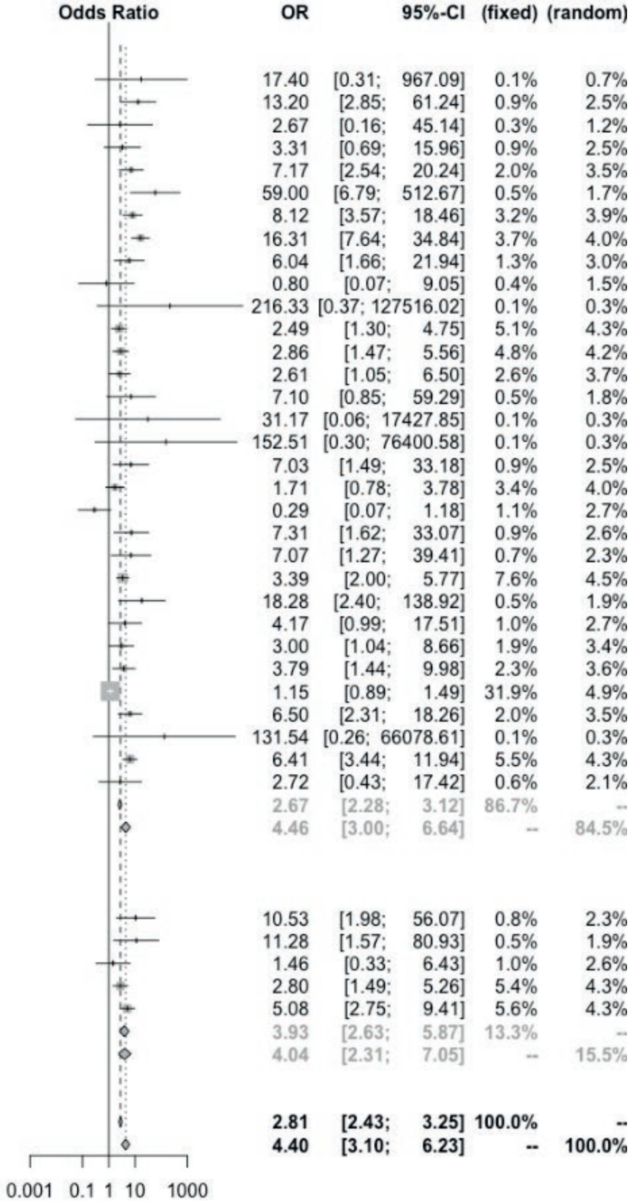

Candidate variable: CRP increase (per 1 mg/L), outcome: severe Covid-19 disease, subgroup analysis by risk of bias (high vs moderate/low)

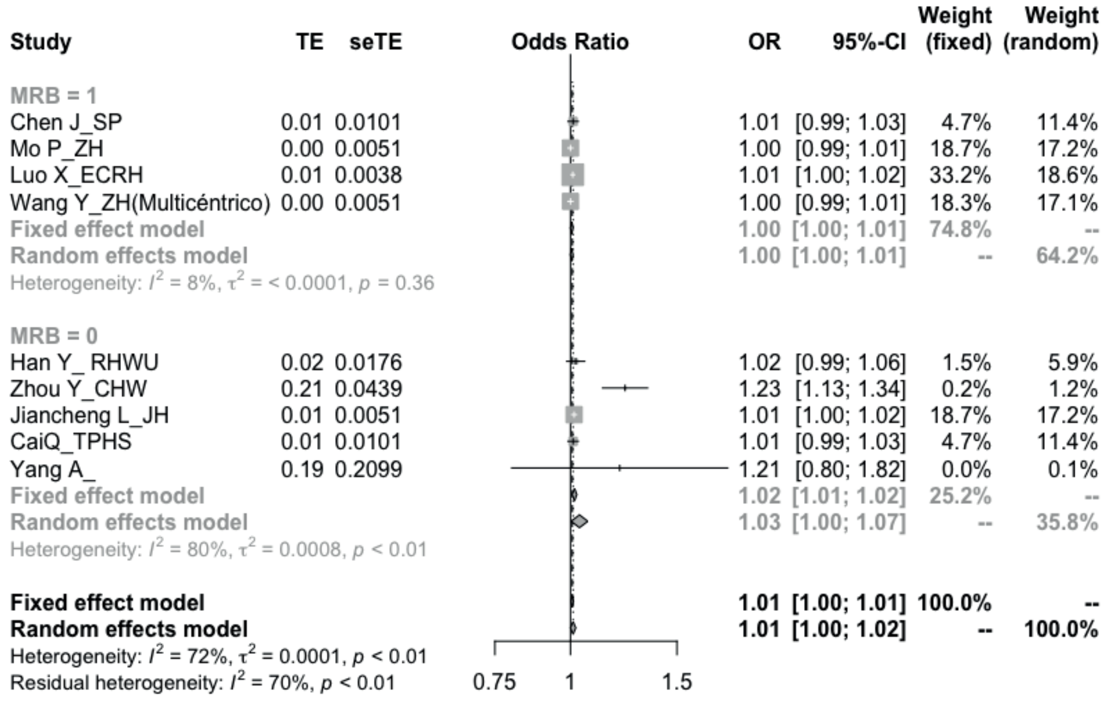

Candidate variable: High ESR (more than 10-20 mm/H), outcome: severe Covid-19 disease

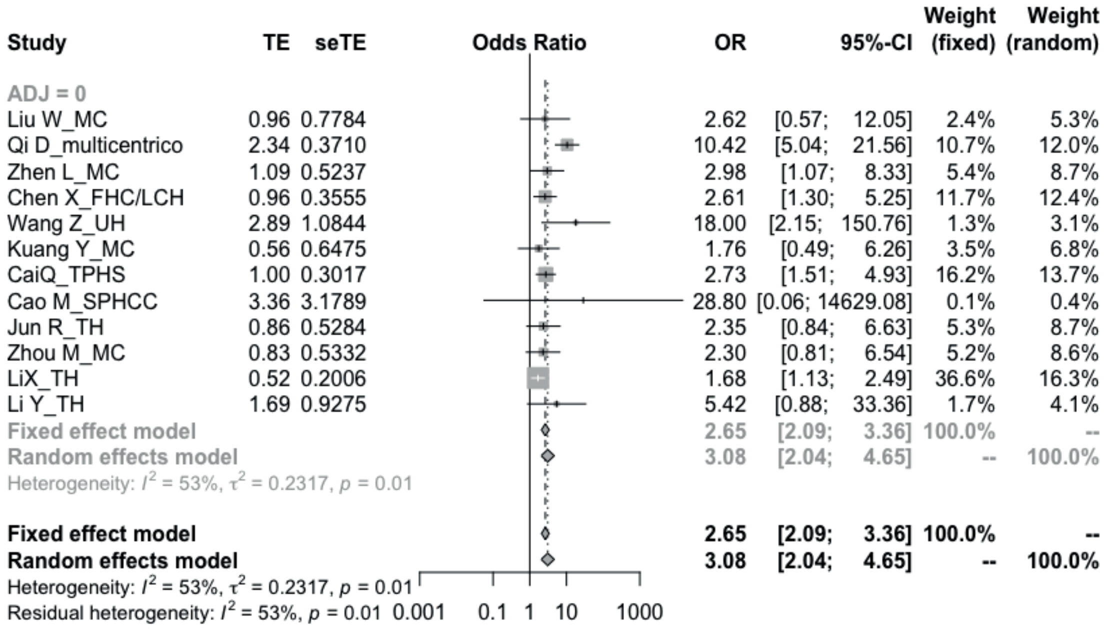

Candidate variable: ESR increase (per 1 mm/H), outcome: severe Covid-19 disease

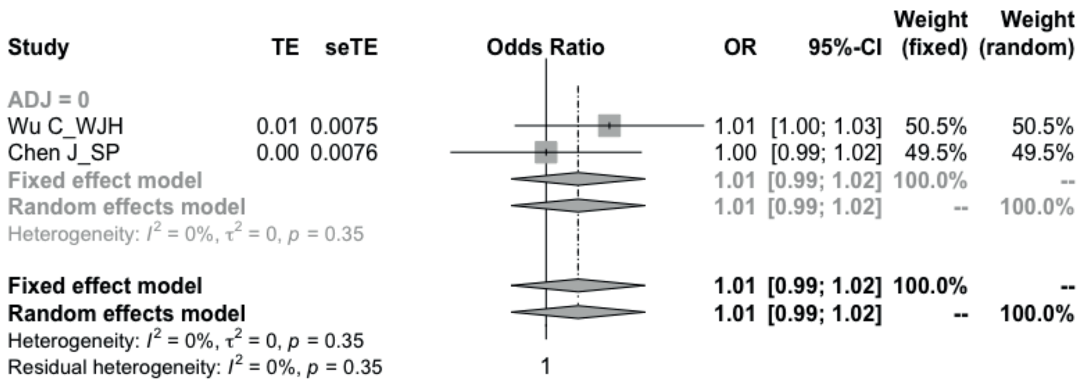

Candidate variable: IL-6 increase (per 1 pg/mL), outcome: severe Covid-19 disease, subgroup analysis: (crude vs adjusted)

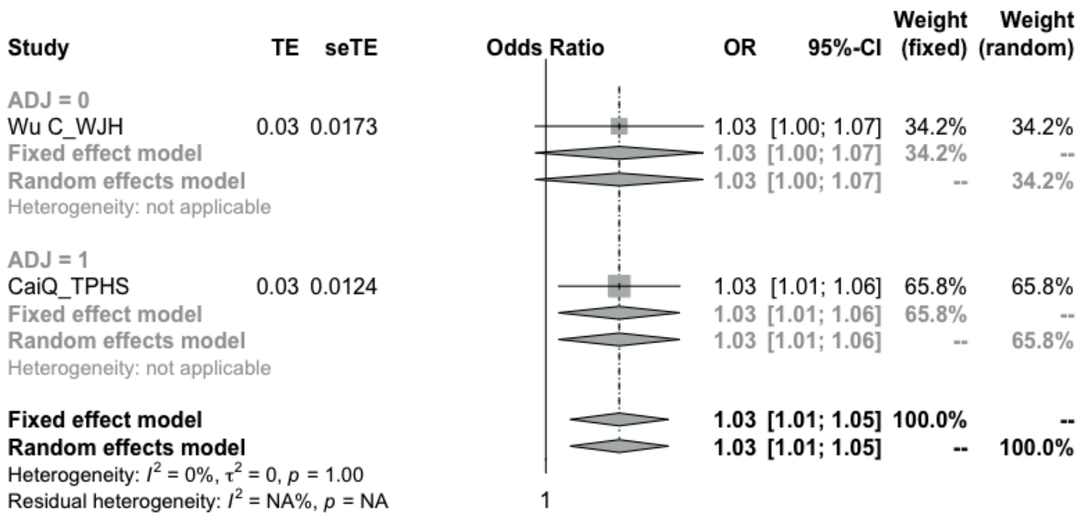

Candidate variable: High interleukin-6 (more than 5-20 pg/ml), outcome: severe Covid-19 disease, subgroup analysis: (crude vs adjusted)

Study	TE	seTE	OR	95%-CI	Weight (fixed)	Weight (random)
**ADJ = 1**						
Gao Y_FSH	2.85	1.0051	17.30	[2.41; 124.05]	3.6%	11.4%
FL_GHCTCPLA	1.06	0.5787	2.89	[0.93; 8.98]	10.9%	17.7%
**Fixed effect model**			4.51	[1.69; 12.06]	14.6%	--
**Random effects model**			5.85	[1.05; 32.51]	--	29.0%
Heterogeneity: $I^2 = 58\%$, $\tau^2 = 0.9285$, $p = 0.12$						
**ADJ = 0**						
Qi D_multicentrico	3.18	0.3980	23.94	[10.97; 52.23]	23.1%	20.7%
Chen G_TH	1.16	1.3509	3.20	[0.23; 45.19]	2.0%	7.9%
Wang Z_UH	6.32	3.2286	558.25	[1.00; 312617.51]	0.4%	1.9%
Wan S_TGCH	1.99	0.5557	7.33	[2.47; 21.78]	11.9%	18.0%
LiX_TH	1.15	0.2762	3.15	[1.83; 5.41]	48.0%	22.4%
**Fixed effect model**			6.27	[4.18; 9.41]	85.4%	--
**Random effects model**			8.23	[2.49; 27.19]	--	71.0%
Heterogeneity: $I^2 = 80\%$, $\tau^2 = 1.1488$, $p < 0.01$						
**Fixed effect model**			**5.98**	**[4.11; 8.70]**	**100.0%**	**--**
**Random effects model**			**7.37**	**[2.97; 18.27]**	**--**	**100.0%**

Heterogeneity: $I^2 = 73\%$, $\tau^2 = 0.8822$, $p < 0.01$

Residual heterogeneity: $I^2 = 77\%$, $p < 0.01$

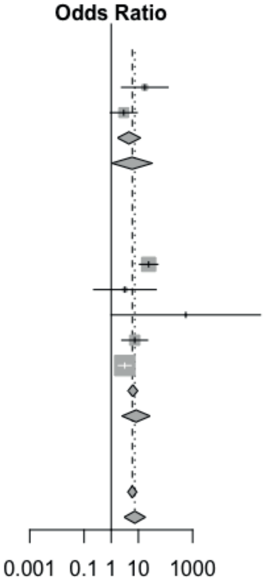

Candidate variable: High procalcitonin (more than 0.01-05 ng/ml), outcome: severe Covid-19 disease, subgroup analysis: (crude vs adjusted)

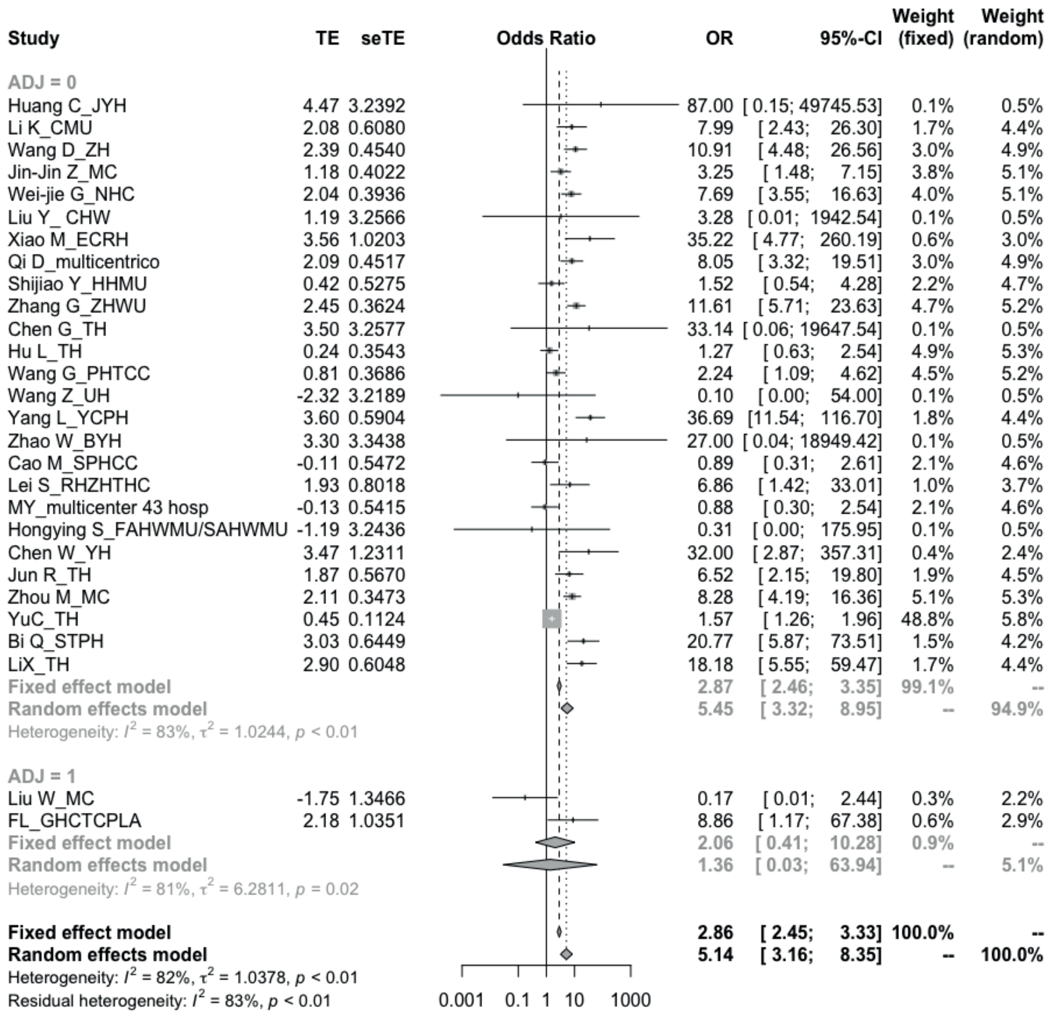

Candidate variable: High total bilirubin (more than 17-21pg/ml), outcome: severe Covid-19 disease

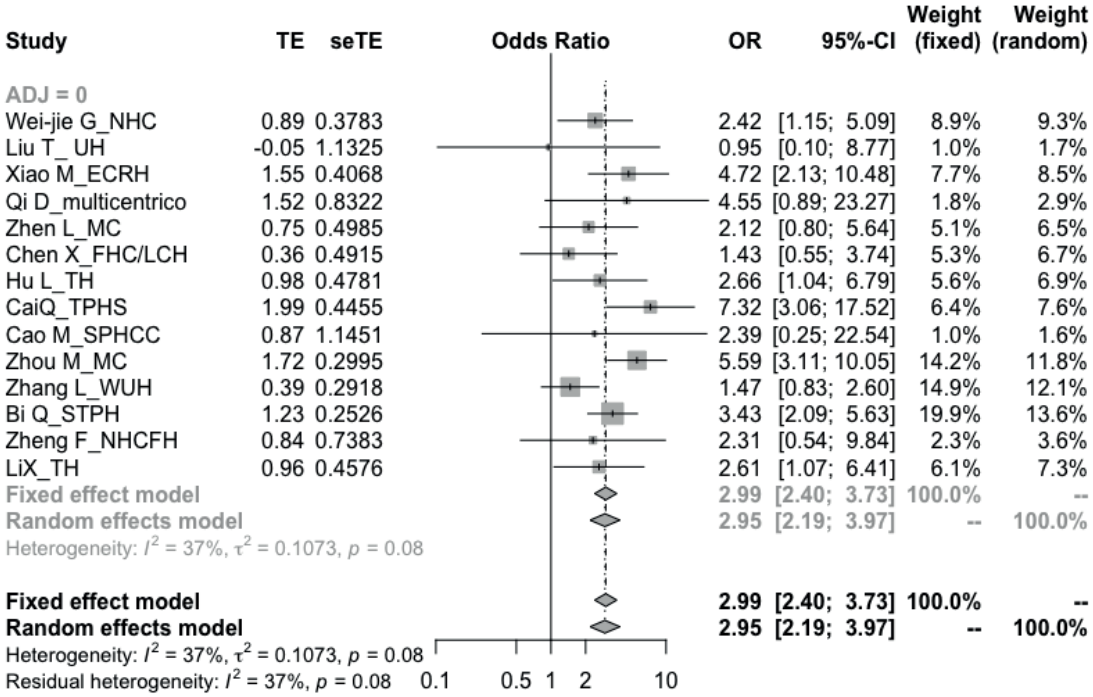

Candidate variable: High AST level (more than 32-40 U/l), outcome: severe Covid-19 disease

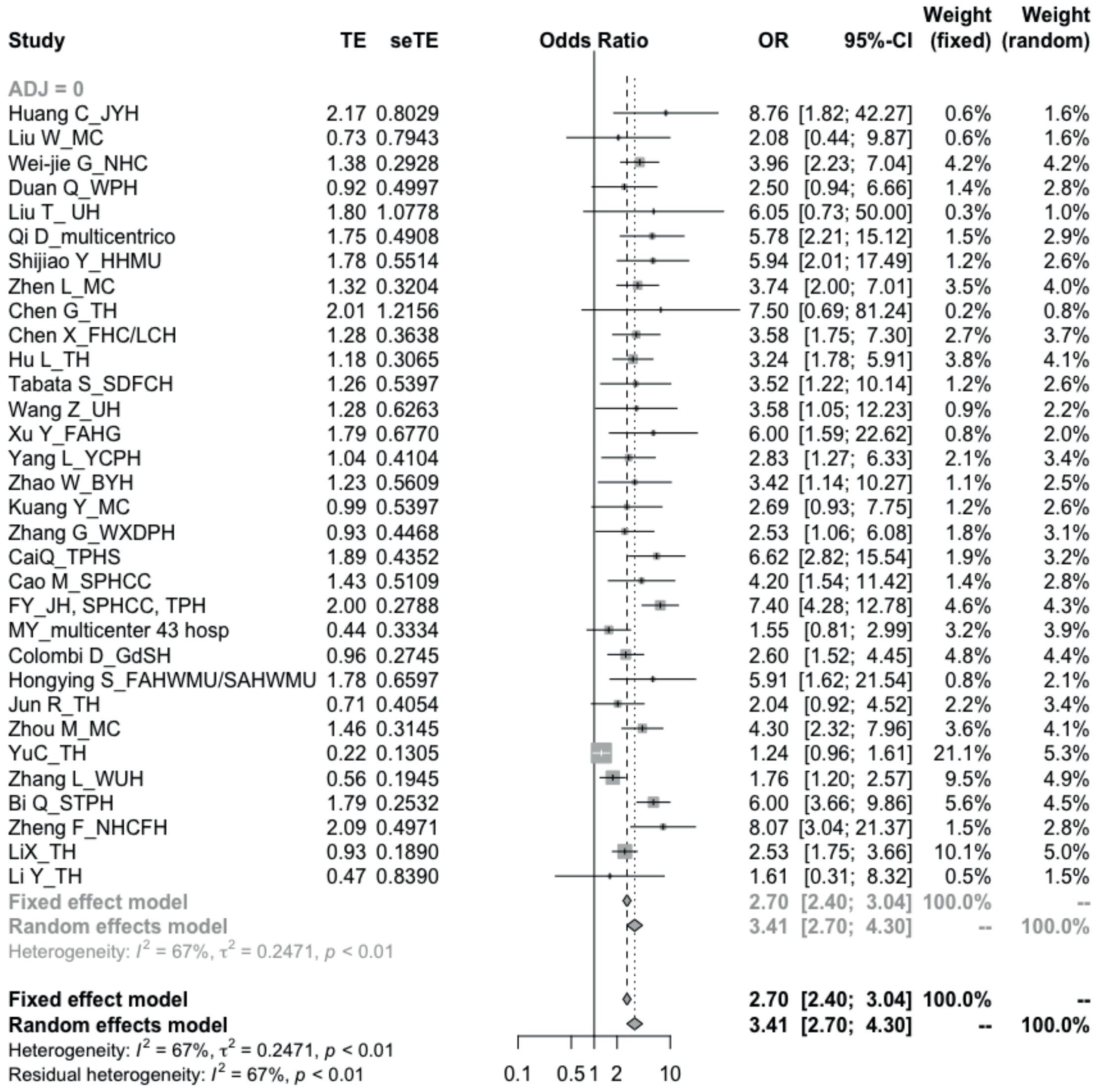

Candidate variable: AST increase (per 1 U/), outcome: severe Covid-19 disease, subgroup analysis by risk of bias (high vs moderate/low)

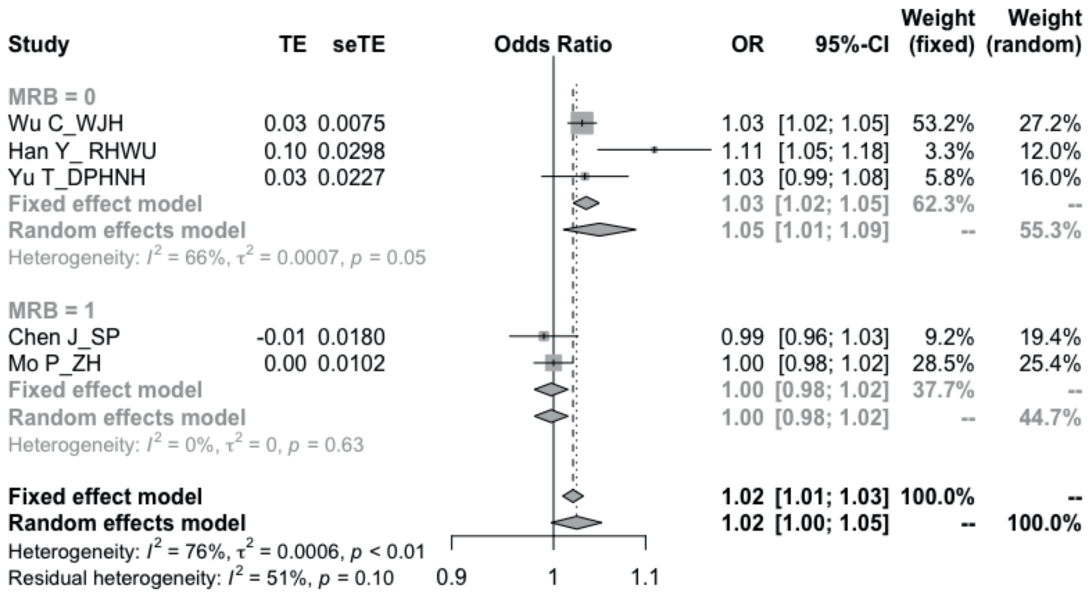

Candidate variable: High ALT level (more than 35-50 U/L), outcome: severe Covid-19 disease, subgroup analysis: (crude vs adjusted)

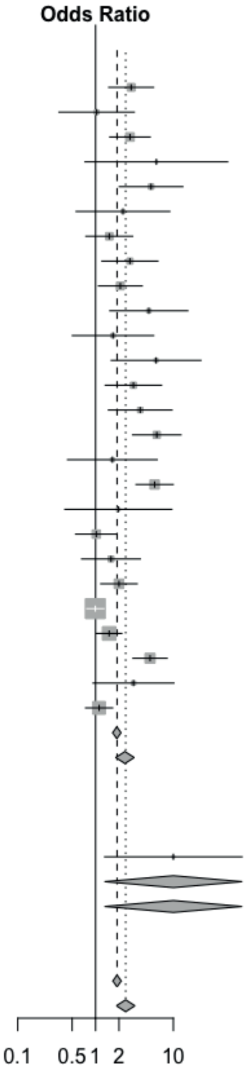

Study	TE	seTE	OR	95%-CI	Weight (fixed)	Weight (random)
**ADJ = 0**						
Wu C_WJH	1.05	0.3381	2.87	[1.48; 5.57]	3.5%	4.4%
Liu W_MC	0.03	0.5688	1.03	[0.34; 3.15]	1.2%	3.0%
Wei-jie G_NHC	1.02	0.3058	2.77	[1.52; 5.04]	4.3%	4.7%
Liu T_ UH	1.80	1.0778	6.05	[0.73; 50.00]	0.3%	1.3%
Qi D_multicentrico	1.64	0.4794	5.17	[2.02; 13.24]	1.7%	3.5%
Shijiao Y_HHMU	0.81	0.7072	2.25	[0.56; 9.01]	0.8%	2.4%
Zhen L_MC	0.41	0.3537	1.50	[0.75; 3.01]	3.2%	4.3%
Chen X_FHC/LCH	1.02	0.4237	2.76	[1.20; 6.34]	2.2%	3.9%
Hu L_TH	0.73	0.3303	2.08	[1.09; 3.98]	3.6%	4.5%
Tabata S_SDFCH	1.58	0.5890	4.84	[1.52; 15.34]	1.1%	2.9%
Wang Z_UH	0.52	0.6138	1.68	[0.50; 5.58]	1.1%	2.8%
Xu Y_FAHG	1.79	0.6770	6.00	[1.59; 22.62]	0.9%	2.5%
Yang L_YCPH	1.12	0.4250	3.07	[1.33; 7.05]	2.2%	3.9%
Zhang G_WXDPH	1.32	0.4806	3.75	[1.46; 9.62]	1.7%	3.5%
CaiQ_TPHS	1.81	0.3653	6.12	[2.99; 12.53]	3.0%	4.3%
Cao M_SPHCC	0.50	0.6765	1.65	[0.44; 6.20]	0.9%	2.5%
FY_JH, SPHCC, TPH	1.75	0.2802	5.75	[3.32; 9.95]	5.1%	4.8%
JX_WFPH	0.67	0.8075	1.96	[0.40; 9.56]	0.6%	2.0%
MY_multicenter 43 hosp	0.02	0.3118	1.02	[0.55; 1.88]	4.1%	4.6%
Jun R_TH	0.46	0.4438	1.58	[0.66; 3.77]	2.0%	3.7%
Zhou M_MC	0.69	0.2744	2.00	[1.17; 3.42]	5.3%	4.9%
YuC_TH	-0.01	0.1303	0.99	[0.77; 1.28]	23.4%	5.7%
Zhang L_WUH	0.40	0.1911	1.50	[1.03; 2.18]	10.9%	5.4%
Bi Q_STPH	1.61	0.2589	5.01	[3.02; 8.33]	5.9%	5.0%
Zheng F_NHCFH	1.12	0.6108	3.08	[0.93; 10.18]	1.1%	2.8%
LiX_TH	0.11	0.2041	1.11	[0.75; 1.66]	9.6%	5.3%
**Fixed effect model**			1.88	[1.66; 2.13]	99.6%	--
**Random effects model**			2.39	[1.81; 3.14]	--	98.6%
Heterogeneity: $I^2 = 74\%$, $\tau^2 = 0.3211$, $p < 0.01$						
**ADJ = 1**						
Hongying S_FAHWMU/SAHWMU	2.30	1.0372	10.00	[1.31; 76.36]	0.4%	1.4%
**Fixed effect model**			10.00	[1.31; 76.36]	0.4%	--
**Random effects model**			10.00	[1.31; 76.36]	--	1.4%
Heterogeneity: not applicable						
**Fixed effect model**			**1.89**	**[1.67; 2.14]**	**100.0%**	**--**
**Random effects model**			**2.44**	**[1.85; 3.21]**	**--**	**100.0%**

Heterogeneity: $I^2 = 74\%$, $\tau^2 = 0.3266$, $p < 0.01$

Residual heterogeneity: $I^2 = 74\%$, $p < 0.01$

Candidate variable: Increase ALT level (per 1 U/L), outcome: severe Covid-19 disease

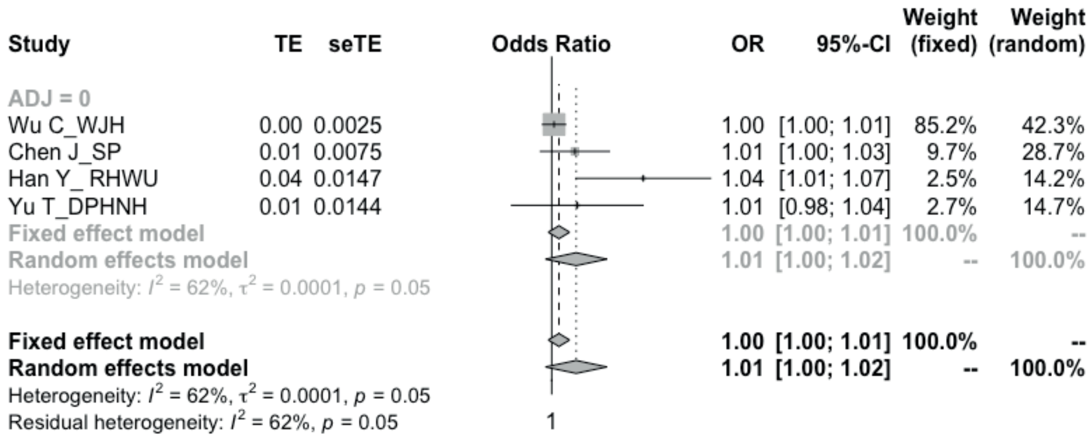

Candidate variable: Low albumin (less than 35-40 g/L), outcome: severe Covid-19 disease, subgroup analysis: (crude vs adjusted)

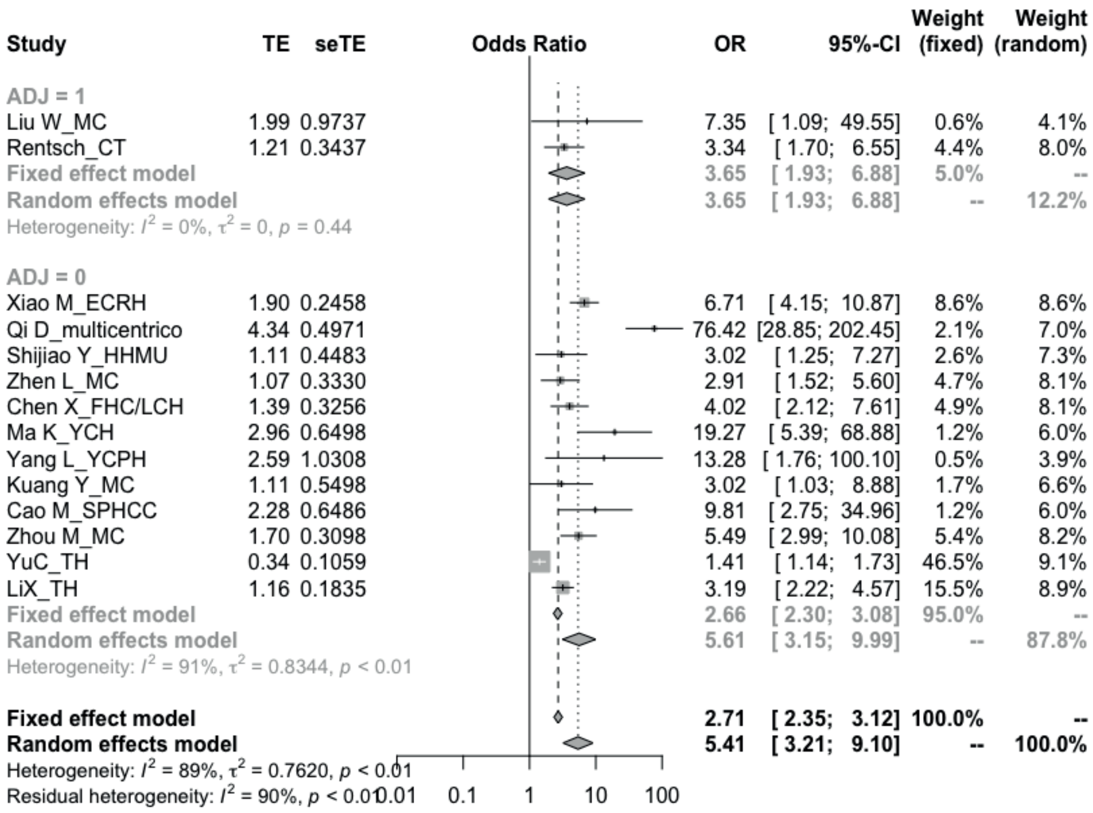

Candidate variable: Increase albumin (per 10 g/L), outcome: severe Covid-19 disease, subgroup analysis: (crude vs adjusted)

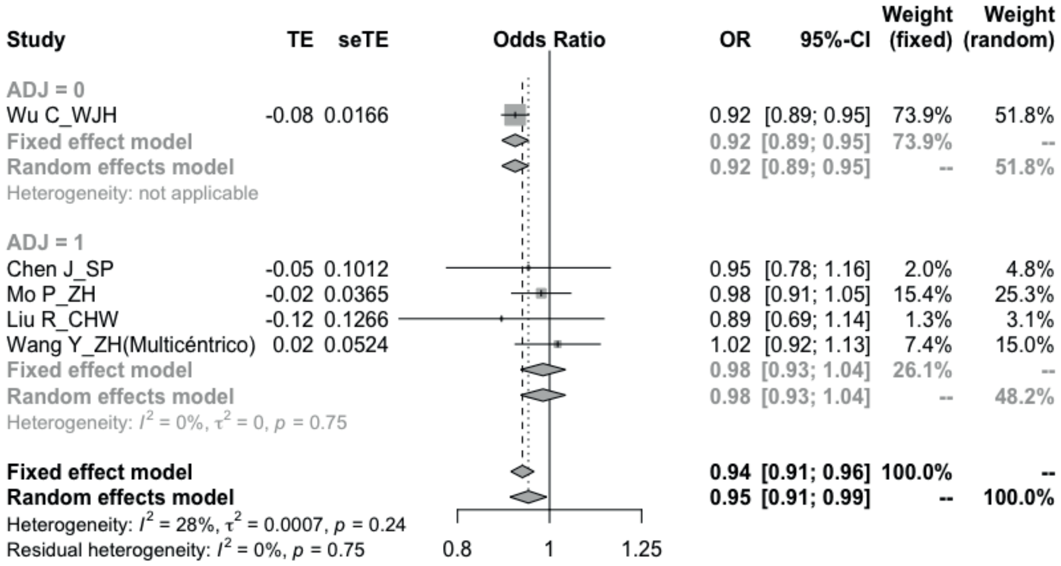

Candidate variable: High glucose (more than 6 mmol/l), outcome: severe Covid-19 disease

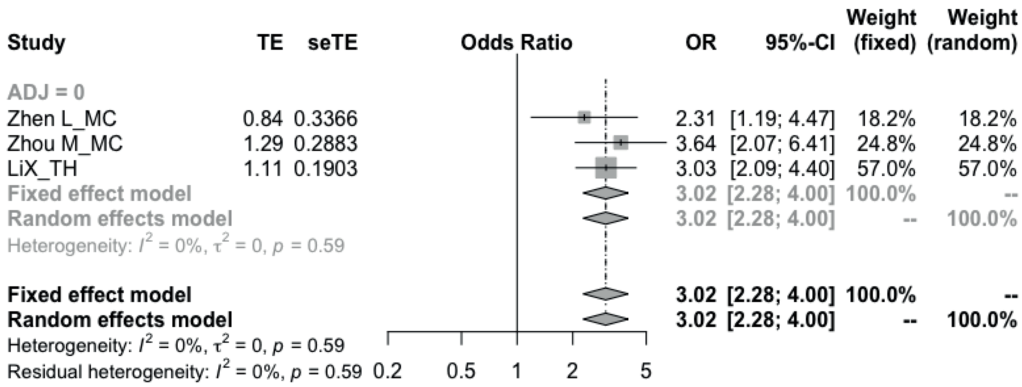

Candidate variable: High lactate (more than 1.5-2.2 mmol/L), outcome: severe Covid-19 disease

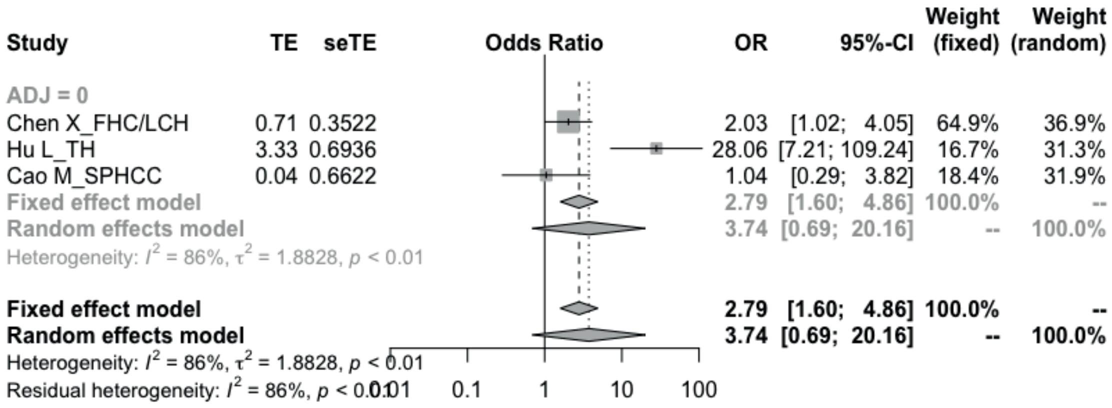

Candidate variable: Lactate increase (per 1 mmol/L), outcome: severe Covid-19 disease

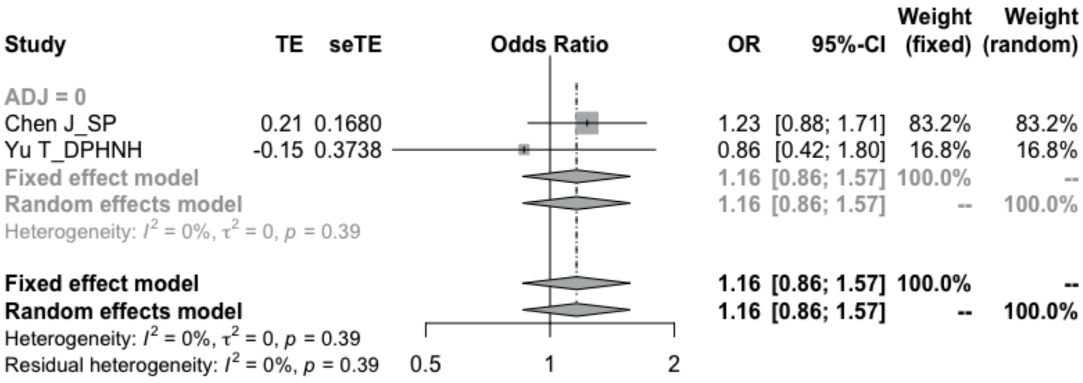

Candidate variable: Any abnormal Radiologic finding, outcome: severe Covid-19 disease

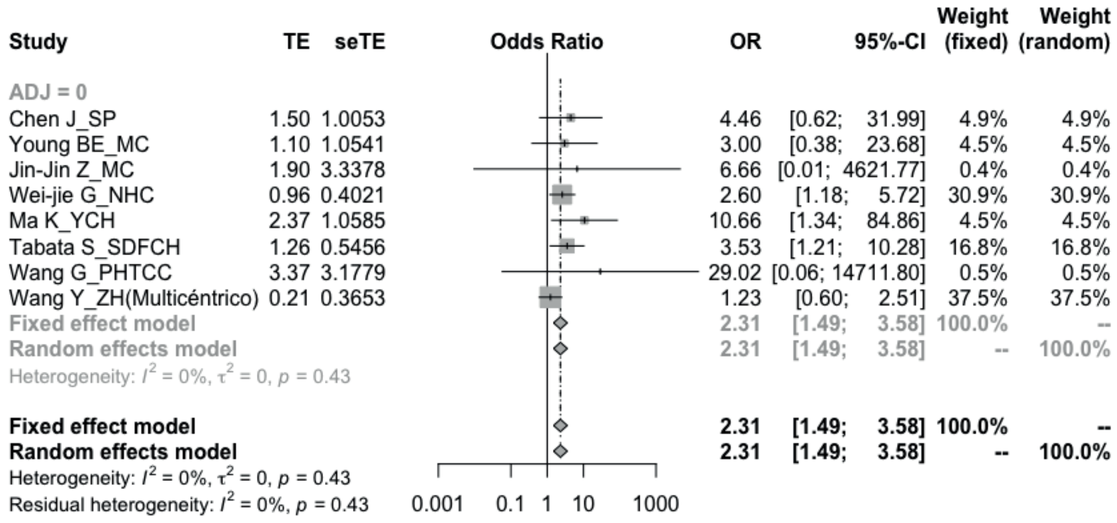

Candidate variable: Interstitial pattern, outcome: severe Covid-19 disease

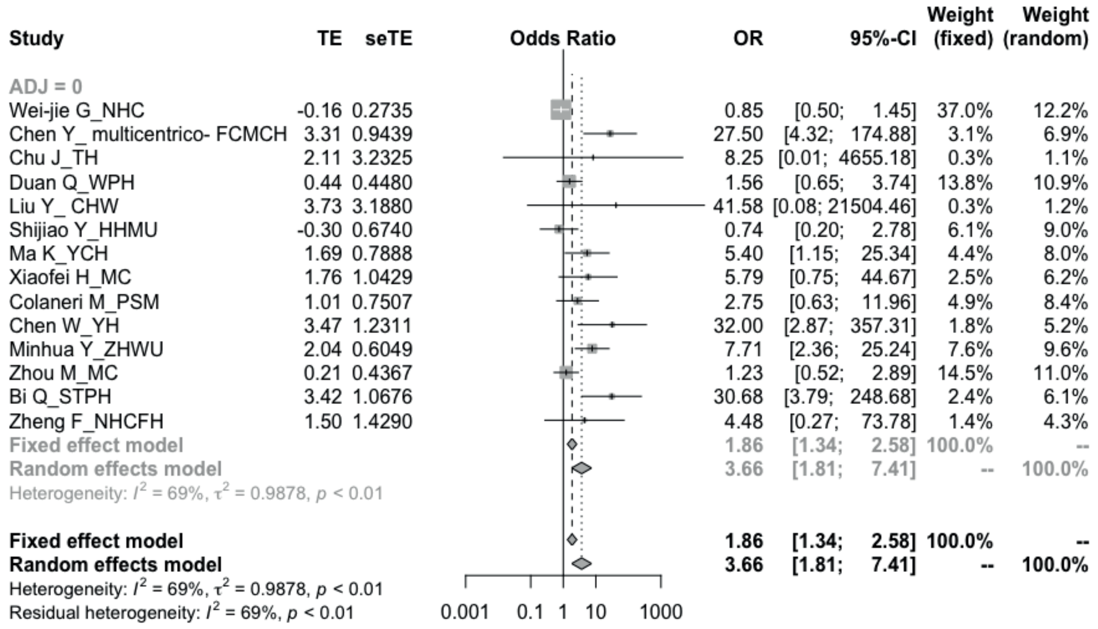

Candidate variable: Ground glass opacity, outcome: severe Covid-19 disease, subgroup analysis: (crude vs adjusted)

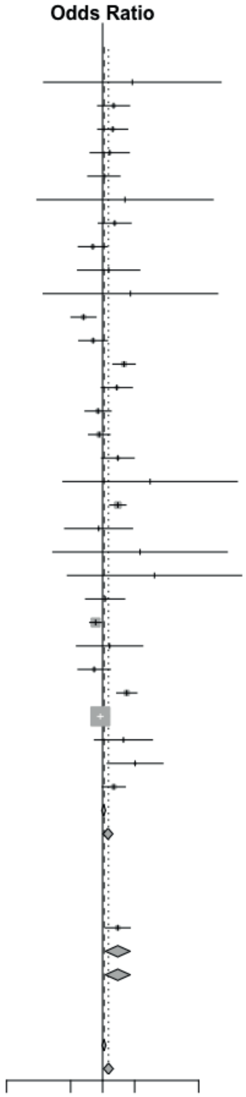

Study	TE	seTE	OR	95%-CI	(fixed)	(random)
**ADJ = 0**						
Li K_CMU	2.14	3.2533	8.48	[0.01; 4985.36]	0.0%	0.3%
Liu K_MC	0.79	0.5835	2.21	[0.70; 6.92]	1.4%	3.7%
Cao W_ X-1 H	0.73	0.5493	2.07	[0.70; 6.07]	1.6%	3.9%
Chu J_TH	0.51	0.7176	1.66	[0.41; 6.79]	0.9%	3.0%
Duan Q_WPH	0.09	0.5878	1.10	[0.35; 3.47]	1.4%	3.7%
Liu T_ UH	1.61	3.2309	5.00	[0.01; 2812.91]	0.0%	0.3%
Shijiao Y_HHMU	0.87	0.6019	2.38	[0.73; 7.73]	1.3%	3.6%
Zhao W_SXH	-0.71	0.5285	0.49	[0.17; 1.39]	1.7%	4.0%
Lei L_CUTGH	0.43	1.1429	1.54	[0.16; 14.49]	0.4%	1.7%
Feng Z_TXH	2.00	3.2006	7.38	[0.01; 3909.80]	0.0%	0.3%
Hu L_TH	-1.37	0.4576	0.25	[0.10; 0.62]	2.3%	4.4%
Li J_CHW	-0.68	0.5272	0.50	[0.18; 1.42]	1.7%	4.0%
Wang G_PHTCC	1.54	0.4088	4.67	[2.09; 10.40]	2.9%	4.7%
Xiaofei H_MC	1.02	0.5707	2.77	[0.91; 8.49]	1.5%	3.8%
Kuang Y_MC	-0.33	0.4766	0.72	[0.28; 1.83]	2.1%	4.3%
FY_JH, SPHCC, TPH	-0.25	0.3925	0.78	[0.36; 1.69]	3.1%	4.8%
CM_FAHSYU	1.09	0.5976	2.99	[0.93; 9.64]	1.3%	3.6%
JX_WFPH	3.41	3.1987	30.35	[0.06; 16029.59]	0.0%	0.3%
MY_multicenter 43 hosp	1.09	0.2959	2.98	[1.67; 5.33]	5.5%	5.4%
Hongying S_FAHWMU/SAHWMU	-0.29	1.2435	0.75	[0.07; 8.58]	0.3%	1.5%
Xin L_CHWC/hospitales en Hunan	2.69	3.1992	14.75	[0.03; 7797.57]	0.0%	0.3%
Ying S_hospitales en Beijing	3.73	3.1948	41.69	[0.08; 21851.41]	0.0%	0.3%
Chen W_YH	0.18	0.7170	1.20	[0.29; 4.89]	0.9%	3.1%
Wang X_DFH	-0.50	0.2155	0.60	[0.40; 0.92]	10.4%	5.8%
Herold T_UH	0.50	1.2142	1.65	[0.15; 17.82]	0.3%	1.5%
Minhua Y_ZHWU	-0.61	0.5929	0.54	[0.17; 1.74]	1.4%	3.7%
Zhou M_MC	1.74	0.3689	5.67	[2.75; 11.69]	3.5%	5.0%
YuC_TH	-0.17	0.1003	0.85	[0.70; 1.03]	47.8%	6.3%
Zhang R_RH	1.50	1.0633	4.46	[0.56; 35.86]	0.4%	1.9%
Bi Q_STPH	2.33	1.0220	10.33	[1.39; 76.54]	0.5%	2.0%
Zheng F_NHCFH	0.80	0.4250	2.23	[0.97; 5.12]	2.7%	4.6%
**Fixed effect model**			1.08	[0.94; 1.24]	97.7%	--
**Random effects model**			1.47	[1.04; 2.08]	--	95.6%
Heterogeneity: $I^2 = 70\%$, $\tau^2 = 0.4636$, $p < 0.01$						
**ADJ = 1**						
Tabata S_SDFCH	1.09	0.4571	2.97	[1.21; 7.27]	2.3%	4.4%
**Fixed effect model**			2.97	[1.21; 7.27]	2.3%	--
**Random effects model**			2.97	[1.21; 7.27]	--	4.4%
Heterogeneity: not applicable						
**Fixed effect model**			**1.11**	**[0.97; 1.27]**	**100.0%**	**--**
**Random effects model**			**1.52**	**[1.08; 2.13]**	**--**	**100.0%**
Heterogeneity: $I^2 = 70\%$, $\tau^2 = 0.4703$, $p < 0.01$						

Candidate variable: Crazy paving pattern, outcome: severe Covid-19 disease, subgroup analysis: (crude vs adjusted)

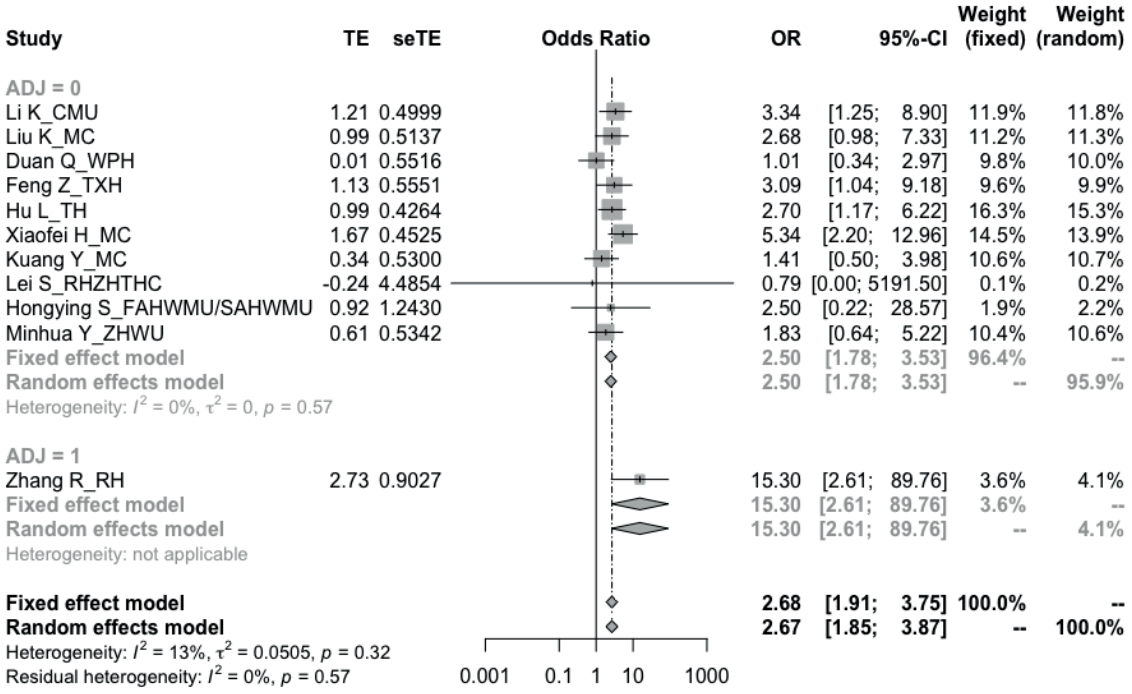

Candidate variable: Consolidation pattern, outcome: severe Covid-19 disease, subgroup analysis: (crude vs adjusted)

Study	TE	seTE	OR	95%-CI	Weight (fixed)	Weight (random)
**ADJ = 0**						
Li K_CMU	1.85	0.6693	6.38	[1.72; 23.69]	1.2%	3.9%
Liu K_MC	5.49	3.1952	241.94	[0.46; 126892.91]	0.1%	0.5%
Jin-Jin Z_MC	0.83	0.2969	2.29	[1.28; 4.10]	6.3%	5.1%
Lu Jiatao_WHH	-1.74	3.2466	0.18	[0.00; 102.11]	0.1%	0.5%
Chen Y_ multicentrico- FCMCH	0.36	0.6981	1.43	[0.36; 5.61]	1.1%	3.8%
Chu J_TH	-0.77	0.9417	0.46	[0.07; 2.92]	0.6%	3.0%
Qi D_multicentrico	2.46	0.6246	11.69	[3.44; 39.76]	1.4%	4.0%
Zhao W_SXH	0.09	0.3809	1.09	[0.52; 2.30]	3.8%	4.8%
Lei L_CUTGH	1.90	0.9025	6.67	[1.14; 39.10]	0.7%	3.1%
Feng Z_TXH	0.14	0.7993	1.15	[0.24; 5.53]	0.9%	3.4%
Ma K_YCH	2.25	0.6774	9.44	[2.50; 35.62]	1.2%	3.8%
Kuang Y_MC	1.28	0.4262	3.58	[1.55; 8.26]	3.0%	4.7%
FY_JH, SPHCC, TPH	-0.98	0.4446	0.38	[0.16; 0.90]	2.8%	4.6%
CM_FAHSYU	2.12	0.5234	8.32	[2.98; 23.20]	2.0%	4.4%
Hongying S_FAHWMU/SAHWMU	0.31	3.2973	1.37	[0.00; 876.74]	0.1%	0.5%
Xin L_CHWC/hospitales en Hunan	1.31	0.5315	3.69	[1.30; 10.46]	2.0%	4.3%
Ying S_hospitales en Beijing	0.47	0.6513	1.61	[0.45; 5.76]	1.3%	3.9%
Chen W_YH	1.23	0.9271	3.43	[0.56; 21.10]	0.6%	3.0%
Wang X_DFH	0.78	0.3674	2.19	[1.07; 4.50]	4.1%	4.9%
Herold T_UH	-0.44	0.7182	0.64	[0.16; 2.63]	1.1%	3.7%
Minhua Y_ZHWU	1.06	0.7643	2.88	[0.64; 12.86]	0.9%	3.5%
Zhou M_MC	1.88	0.3354	6.58	[3.41; 12.70]	4.9%	5.0%
YuC_TH	-0.42	0.1024	0.65	[0.54; 0.80]	52.7%	5.5%
Zhang R_RH	1.97	0.5347	7.16	[2.51; 20.43]	1.9%	4.3%
Zheng F_NHCFH	-0.39	0.4257	0.68	[0.30; 1.57]	3.0%	4.7%
**Fixed effect model**			1.20	[1.04; 1.40]	97.8%	--
**Random effects model**			2.35	[1.44; 3.83]	--	92.9%
Heterogeneity: $I^2 = 84\%$, $\tau^2 = 1.0480$, $p < 0.01$						
**ADJ = 1**						
Tabata S_SDFCH	1.18	0.5874	3.24	[1.02; 10.25]	1.6%	4.1%
Zhang L_TH	1.94	0.9488	6.96	[1.08; 44.70]	0.6%	3.0%
**Fixed effect model**			4.00	[1.50; 10.66]	2.2%	--
**Random effects model**			4.00	[1.50; 10.66]	--	7.1%
Heterogeneity: $I^2 = 0\%$, $\tau^2 = 0$, $p = 0.49$						
**Fixed effect model**			**1.24**	**[1.07; 1.43]**	**100.0%**	**--**
**Random effects model**			**2.46**	**[1.54; 3.93]**	**--**	**100.0%**
Heterogeneity: $I^2 = 84\%$, $\tau^2 = 1.0381$, $p < 0.01$						
Residual heterogeneity: $I^2 = 84\%$, $p < 0.01$						

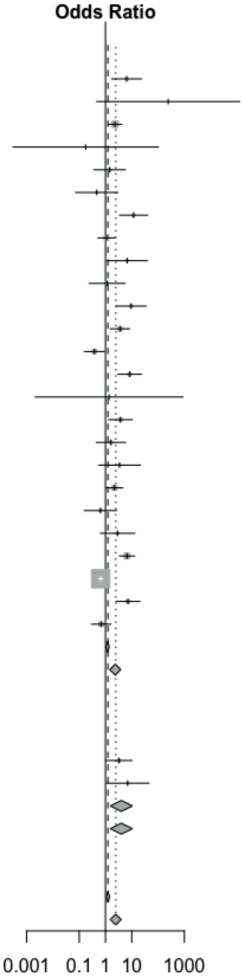

Candidate variable: Enlarged lymph nodes (CT assessment), outcome: severe Covid-19 disease

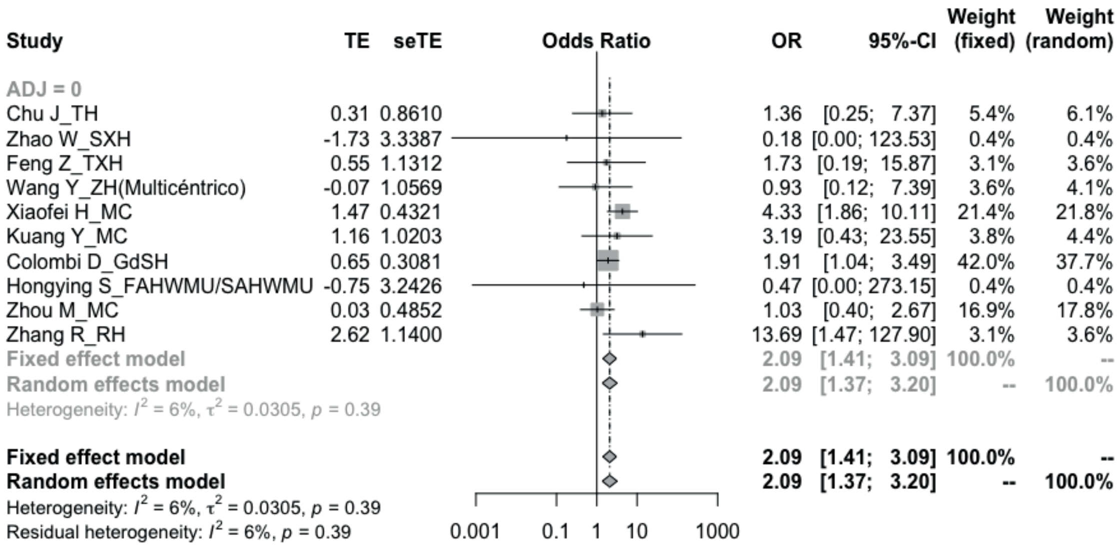

Candidate variable: Pleural effusion (CT assessment), outcome: severe Covid-19 disease, subgroup analysis: (crude vs adjusted)

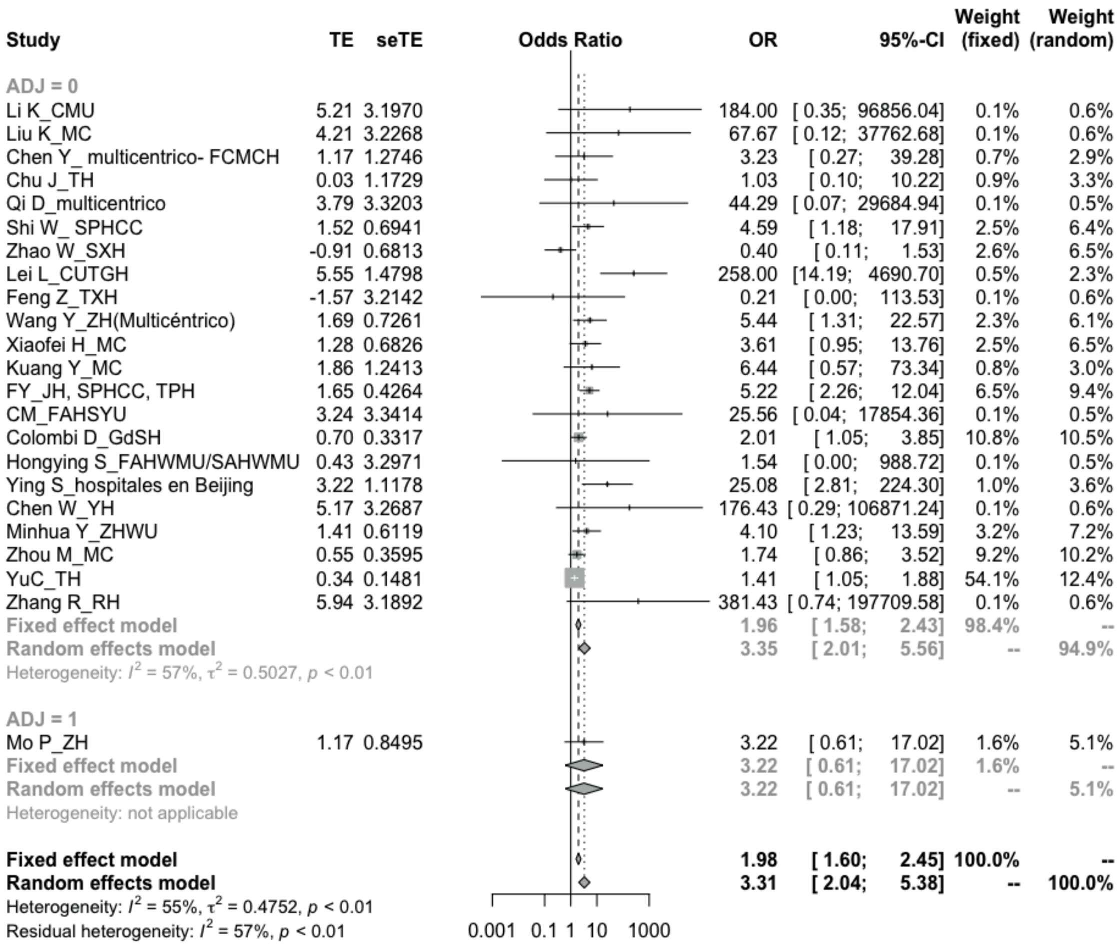

Candidate variable: Bilateral compromise (CT assesment), outcome: severe Covid-19 disease

Study	TE	seTE	OR	95%-CI	Weight (fixed)	Weight (random)
ADJ = 0						
Huang C_JYH	1.47	3.3503	4.33	[0.01; 3080.50]	0.1%	0.5%
Li K_CMU	2.89	3.2116	18.06	[0.03; 9780.32]	0.1%	0.5%
Liu K_MC	4.93	3.1827	138.58	[0.27; 70925.14]	0.1%	0.5%
Liu W_MC	0.29	0.6735	1.34	[0.36; 5.00]	1.5%	3.6%
Jin-Jin Z_MC	0.67	0.6193	1.95	[0.58; 6.56]	1.7%	3.8%
Wei-jie G_NHC	1.61	0.3745	4.99	[2.40; 10.40]	4.7%	4.4%
Lu Jiatao_WHH	-0.63	0.2298	0.53	[0.34; 0.83]	12.5%	4.7%
Chen Y_ multicentrico- FCMCH	2.66	3.2623	14.25	[0.02; 8524.88]	0.1%	0.5%
Duan Q_WPH	-0.01	0.5516	0.99	[0.34; 2.92]	2.2%	4.0%
Shi W_ SPHCC	1.67	0.7516	5.33	[1.22; 23.25]	1.2%	3.4%
Shijiao Y_HHMU	1.03	0.5418	2.79	[0.96; 8.07]	2.2%	4.0%
Zhang G_ZHWU	3.01	3.1927	20.28	[0.04; 10586.55]	0.1%	0.5%
Zhao W_SXH	5.39	3.1755	219.49	[0.43; 110775.86]	0.1%	0.5%
Chen G_TH	1.46	1.2554	4.29	[0.37; 50.19]	0.4%	2.2%
Feng Z_TXH	-0.06	0.8053	0.95	[0.20; 4.58]	1.0%	3.2%
Hu L_TH	-1.14	0.4572	0.32	[0.13; 0.78]	3.2%	4.2%
Li J_CHW	2.50	1.0537	12.16	[1.54; 95.91]	0.6%	2.6%
Liu J_BDH	0.92	0.8294	2.50	[0.49; 12.70]	1.0%	3.2%
Ma K_YCH	4.78	3.1806	119.50	[0.23; 60916.28]	0.1%	0.5%
Tabata S_SDFCH	1.18	0.4587	3.27	[1.33; 8.03]	3.1%	4.2%
Yang L_YCPH	1.66	1.0391	5.25	[0.69; 40.24]	0.6%	2.7%
Zhao W_BYH	3.38	1.0422	29.47	[3.82; 227.24]	0.6%	2.6%
JX_WFPH	3.81	3.1964	45.07	[0.09; 23695.01]	0.1%	0.5%
Colombi D_GdSH	-0.68	0.5858	0.51	[0.16; 1.60]	1.9%	3.9%
Hongying S_FAHWMU/SAHWMU	1.94	3.2258	6.95	[0.01; 3868.08]	0.1%	0.5%
Xin L_CHWC/hospitales en Hunan	2.29	1.0530	9.85	[1.25; 77.58]	0.6%	2.6%
Ying S_hospitales en Beijing	7.30	3.2059	1478.20	[2.76; 791812.33]	0.1%	0.5%
Chen W_YH	4.40	3.1894	81.79	[0.16; 42420.54]	0.1%	0.5%
Herold T_UH	-0.74	1.0713	0.48	[0.06; 3.89]	0.6%	2.6%
Dreher M_UHA	3.29	1.0946	26.83	[3.14; 229.31]	0.5%	2.5%
Zhou M_MC	1.13	0.4820	3.09	[1.20; 7.95]	2.8%	4.1%
YuC_TH	-0.70	0.1306	0.50	[0.38; 0.64]	38.6%	4.8%
Wang L_SPH	3.36	3.1882	28.68	[0.06; 14838.31]	0.1%	0.5%
Zhang R_RH	2.86	0.7620	17.50	[3.93; 77.93]	1.1%	3.4%
Bi Q_STPH	2.50	0.5240	12.23	[4.38; 34.16]	2.4%	4.0%
Wu J_TFAH	-0.35	0.2710	0.70	[0.41; 1.20]	9.0%	4.6%
Zheng F_NHCFH	1.95	0.5648	7.02	[2.32; 21.23]	2.1%	3.9%
LiX_TH	1.02	0.4556	2.77	[1.13; 6.76]	3.2%	4.2%
Fixed effect model			1.06	[0.90; 1.24]	100.0%	--
Random effects model			2.99	[1.83; 4.87]	--	100.0%
Heterogeneity: $I^2 = 81\%$, $\tau^2 = 1.2735$, $p < 0.01$						
**Fixed effect model**			**1.06**	**[0.90; 1.24]**	**100.0%**	**--**
**Random effects model**			**2.99**	**[1.83; 4.87]**	**--**	**100.0%**

Heterogeneity: $I^2 = 81\%$, $\tau^2 = 1.2735$, $p < 0.01$

Residual heterogeneity: $I^2 = 81\%$, $p < 0.01$

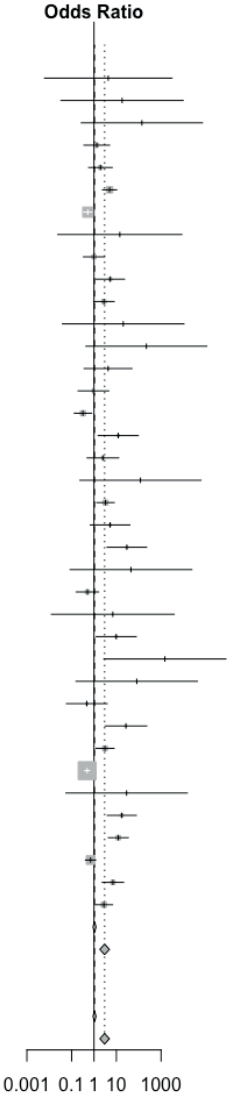

Candidate variable: High APACHE score (more than 8), outcome: severe Covid-19 disease

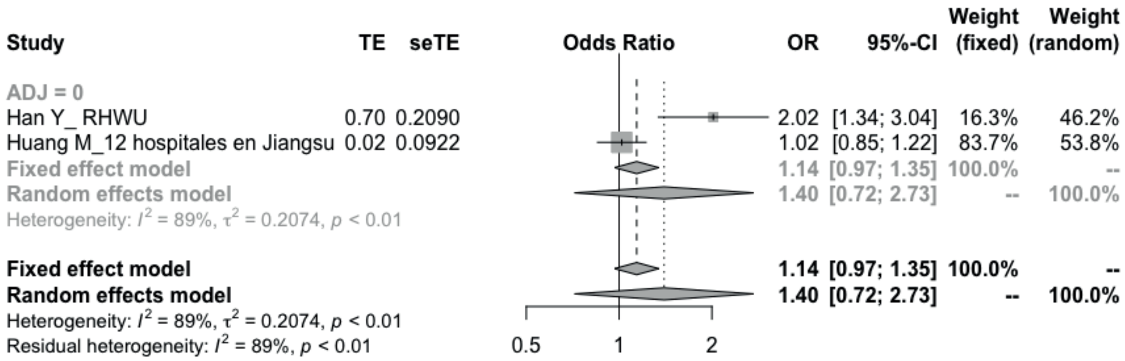

Candidate variable: High SOFA score (more than 2), outcome: severe Covid-19 disease

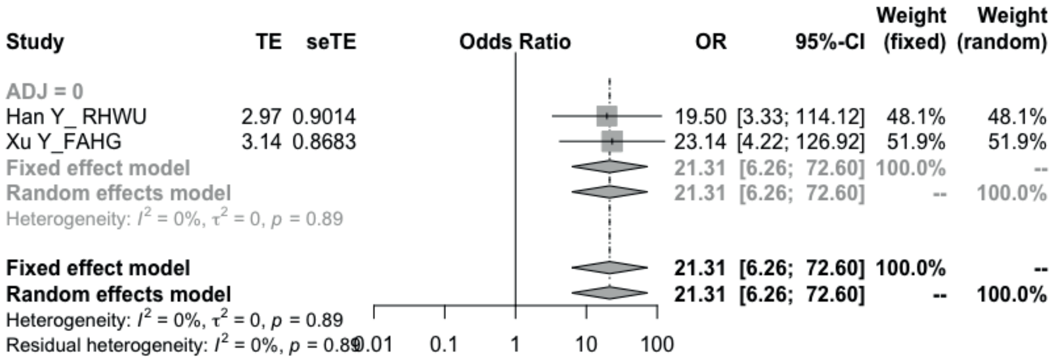

Supplement: S2 Appendix — This file contains the Forest plots for all assessed candidate variables. (PDF) [file pone.0241955.s007.pdf]
