# Supplementary material for: Prognostic factors for severity and mortality in patients infected with COVID-19: A systematic review
Source: PLoS One. 2020 Nov 17;15(11):e0241955. doi: 10.1371/journal.pone.0241955 (PMC7671522; doi:10.1371/journal.pone.0241955)

Subgroup analyses Forest plots

Glossary

Sev: Severe COVID-19 disease

ARM/IVM: invasive mechanical ventilation

ARDS: acute respiratory distress syndrome

ICU: intensive care unit

Sat <90%: oxygen saturation less than 90%

OTHER: other severity definitions

ALT: Alanine aminotransferase

APTT: activated partial thromboplastin time

APACHE: Acute Physiology And Chronic Health Evaluation II

AST: Aspartate aminotransferase

FDP: Fibrin Degradation Product

NA: Not applicable

PT: prothrombin time

Candidate variable: Smoking (Active, present smoker), outcome: severe COVID-19 disease, subgroup analysis by COVID-19 severity definition

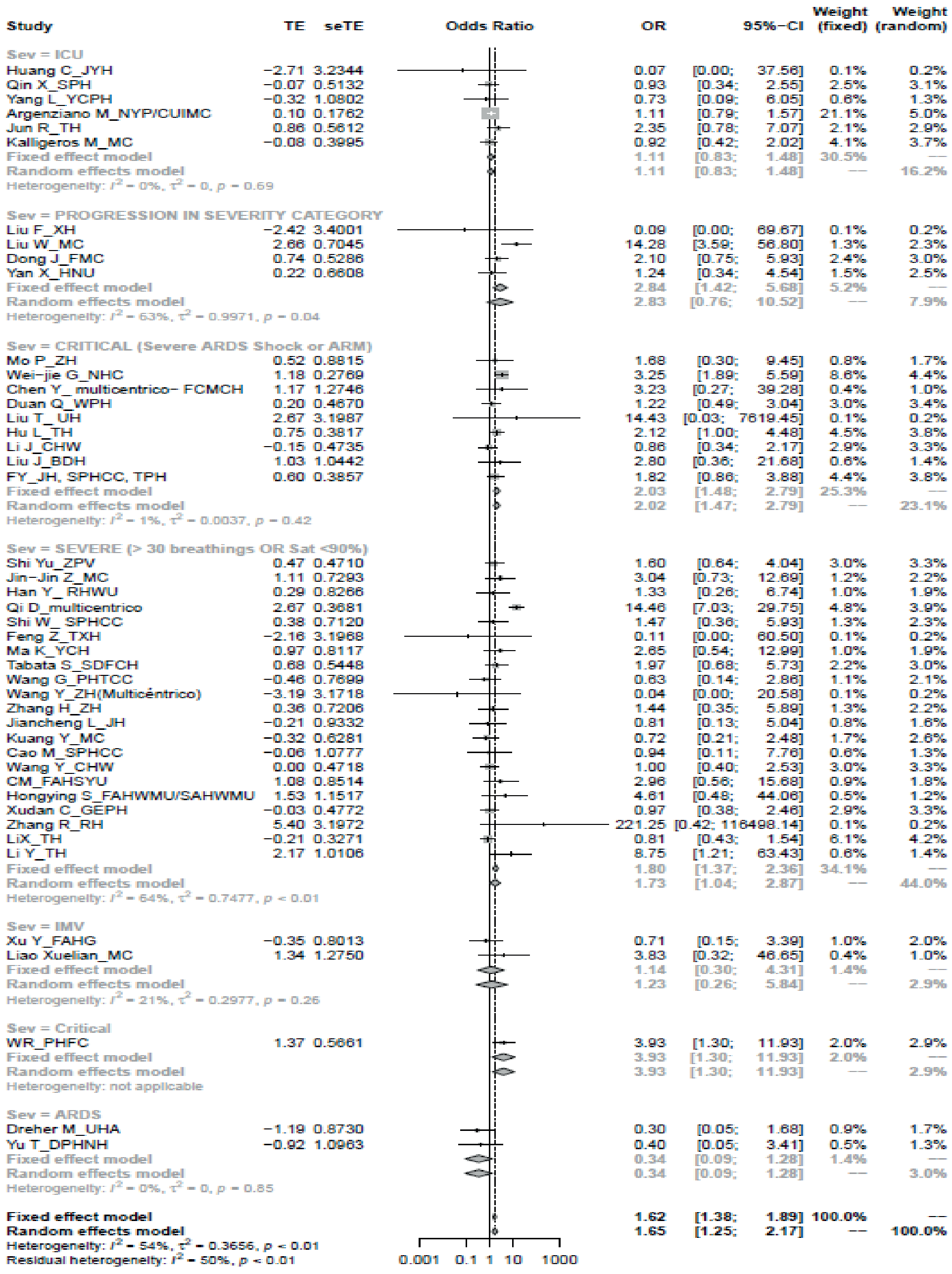

Candidate variable: Smoking (Active, present smoker), outcome: severe COVID-19 disease, subgroup analysis by COVID-19 severity definition

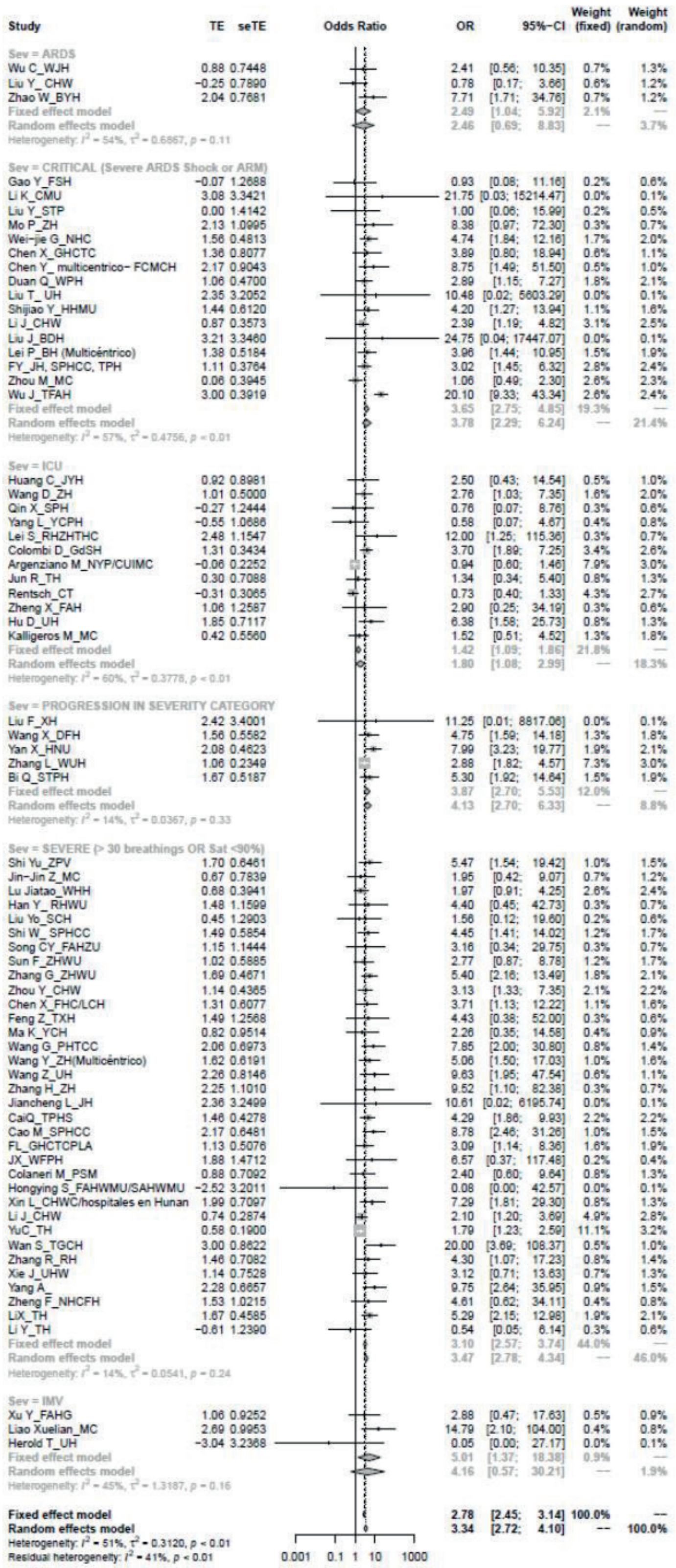

Candidate variable: Cerebrovascular disease (History of stroke or CNS disease), outcome: severe COVID-19 disease, subgroup analysis by COVID-19 severity definition

Study	TE	seTE	OR	95%-CI	Weight (fixed)	Weight (random)
**Sev = CRITICAL (Severe ARDS Shock or ARM)**						
Mo P_ZH	−2.32	1.2786	0.10	[0.01; 1.20]	0.5%	1.6%
Wei−jie G_NHC	1.77	0.5981	5.89	[1.82; 19.03]	2.4%	3.8%
Chen X_GHCTC	1.50	0.9281	4.46	[0.72; 27.51]	1.0%	2.5%
Chen Y_ multicentrico− FCMCH	1.17	1.2746	3.23	[0.27; 39.28]	0.5%	1.6%
Hu L_TH	−1.82	3.1916	0.16	[0.00; 84.20]	0.1%	0.3%
Li J_CHW	0.18	0.4593	1.20	[0.49; 2.96]	4.1%	4.4%
Lei P_BH (Multicéntrico)	−0.96	3.3417	0.38	[0.00; 266.75]	0.1%	0.3%
FY_JH, SPHCC, TPH	1.74	0.5047	5.69	[2.12; 15.31]	3.4%	4.2%
Zhou M_MC	−0.17	0.3990	0.84	[0.38; 1.84]	5.5%	4.7%
Fixed effect model			1.84	[1.19; 2.84]	17.7%	--
Random effects model			1.86	[0.80; 4.29]	--	23.5%
Heterogeneity: $I^2 = 61\%$, $\tau^2 = 0.7910$, $p < 0.01$						
**Sev = ICU**						
Wang D_ZH	3.01	1.0999	20.20	[2.34; 174.43]	0.7%	2.0%
Lei S_RHZHTHC	3.38	3.2602	29.23	[0.05; 17416.52]	0.1%	0.3%
Colombi D_GdSH	0.69	0.3535	2.00	[1.00; 4.00]	7.0%	5.0%
Argenziano M_NYP/CUIMC	0.06	0.2746	1.06	[0.62; 1.81]	11.5%	5.3%
Zheng X_FAH	0.32	1.4429	1.38	[0.08; 23.36]	0.4%	1.3%
Fixed effect model			1.50	[1.00; 2.27]	19.7%	--
Random effects model			2.07	[0.88; 4.88]	--	14.0%
Heterogeneity: $I^2 = 54\%$, $\tau^2 = 0.3896$, $p = 0.07$						
**Sev = SEVERE (> 30 breathings OR Sat <90%)**						
Jin−Jin Z_MC	1.06	1.2370	2.89	[0.26; 32.68]	0.6%	1.7%
Lu Jiatao_WHH	0.82	0.9200	2.28	[0.38; 13.83]	1.0%	2.5%
Sun F_ZHWU	−2.45	3.1961	0.09	[0.00; 45.56]	0.1%	0.3%
Zhang G_ZHWU	2.32	0.6081	10.13	[3.07; 33.34]	2.4%	3.7%
Chen X_FHC/LCH	1.10	0.7475	3.01	[0.70; 13.04]	1.6%	3.1%
Feng Z_TXH	−0.08	3.3445	0.93	[0.00; 650.70]	0.1%	0.3%
Ma K_YCH	1.24	1.0352	3.44	[0.45; 26.20]	0.8%	2.1%
Wang G_PHTCC	1.05	0.8852	2.87	[0.51; 16.28]	1.1%	2.6%
Wang Y_ZH(Multicéntrico)	1.14	0.8150	3.13	[0.63; 15.44]	1.3%	2.8%
Zhang H_ZH	0.53	0.9477	1.71	[0.27; 10.94]	1.0%	2.4%
Jiancheng L_JH	−0.22	0.6753	0.80	[0.21; 3.01]	1.9%	3.4%
Mingfeng H_SPH	−1.62	3.2505	0.20	[0.00; 115.37]	0.1%	0.3%
Wang Y_CHW	0.38	0.7916	1.46	[0.31; 6.88]	1.4%	2.9%
JX_WFPH	3.94	3.3584	51.43	[0.07; 37140.02]	0.1%	0.3%
Xin L_CHWC/hospitales en Hunan	1.78	0.8810	5.92	[1.05; 33.29]	1.1%	2.6%
Chen W_YH	4.29	3.3543	73.12	[0.10; 52386.57]	0.1%	0.3%
Li J_CHW	1.35	0.2992	3.86	[2.15; 6.94]	9.7%	5.2%
YuC_TH	0.25	0.2729	1.28	[0.75; 2.19]	11.7%	5.3%
Wang L_SPH	−0.43	3.3468	0.65	[0.00; 458.56]	0.1%	0.3%
Zheng F_NHCFH	0.39	1.1729	1.47	[0.15; 14.66]	0.6%	1.8%
Fixed effect model			2.36	[1.74; 3.19]	36.6%	--
Random effects model			2.46	[1.69; 3.58]	--	44.2%
Heterogeneity: $I^2 = 13\%$, $\tau^2 = 0.0861$, $p = 0.29$						
**Sev = ARDS**						
Liu Y_ CHW	4.27	3.1946	71.49	[0.14; 37452.78]	0.1%	0.3%
Zhao W_BYH	1.08	1.4389	2.95	[0.18; 49.46]	0.4%	1.3%
Dreher M_UHA	0.43	0.8227	1.53	[0.31; 7.69]	1.3%	2.8%
WangL_RH	1.49	0.9061	4.44	[0.75; 26.25]	1.1%	2.5%
Fixed effect model			2.81	[0.95; 8.31]	2.9%	--
Random effects model			2.81	[0.95; 8.31]	--	7.0%
Heterogeneity: $I^2 = 0\%$, $\tau^2 = 0$, $p = 0.61$						
**Sev = OTHER**						
Ying S_hospitales en Beijing	3.90	3.2569	49.18	[0.08; 29110.25]	0.1%	0.3%
Fixed effect model			49.18	[0.08; 29110.25]	0.1%	--
Random effects model			49.18	[0.08; 29110.25]	--	0.3%
Heterogeneity: not applicable						
**Sev = PROGRESSION IN SEVERITY CATEGORY**						
Yan X_HNU	1.46	0.8948	4.32	[0.75; 24.94]	1.1%	2.6%
Zhang L_WUH	2.07	0.8076	7.90	[1.62; 38.48]	1.3%	2.9%
Bi Q_STPH	2.22	0.2056	9.23	[6.17; 13.81]	20.6%	5.6%
Fixed effect model			8.82	[6.03; 12.92]	23.0%	--
Random effects model			8.82	[6.03; 12.92]	--	11.1%
Heterogeneity: $I^2 = 0\%$, $\tau^2 = 0$, $p = 0.70$						
**Fixed effect model**			**2.82**	**[2.35; 3.38]**	**100.0%**	--
**Random effects model**			**2.67**	**[1.84; 3.87]**	--	**100.0%**
Heterogeneity: $I^2 = 60\%$, $\tau^2 = 0.5955$, $p < 0.01$						
Residual heterogeneity: $I^2 = 33\%$, $p = 0.03$						

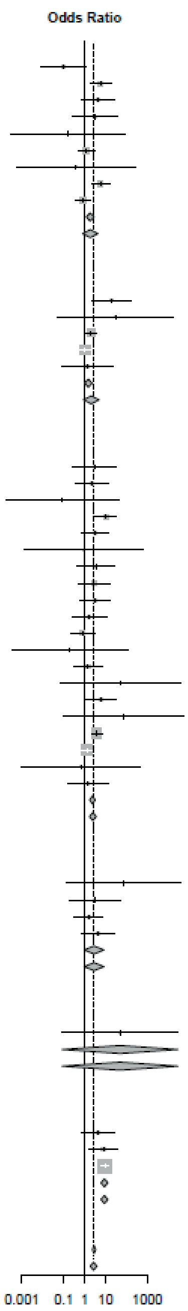

Candidate variable: Arterial hypertension, outcome: severe COVID-19 disease, subgroup analysis by COVID-19 severity definition

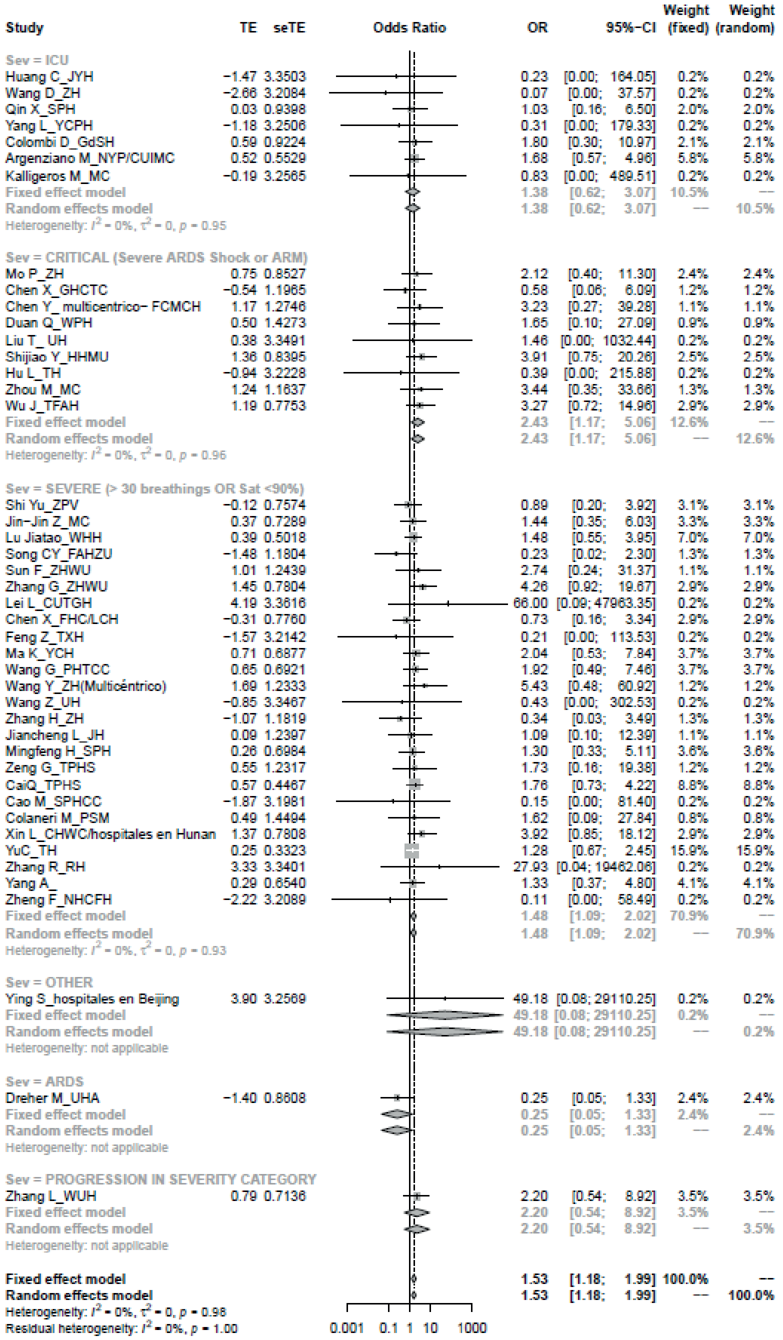

Candidate variable: Chronic kidney disease, outcome: severe COVID-19 disease, subgroup analysis by COVID-19 severity definition

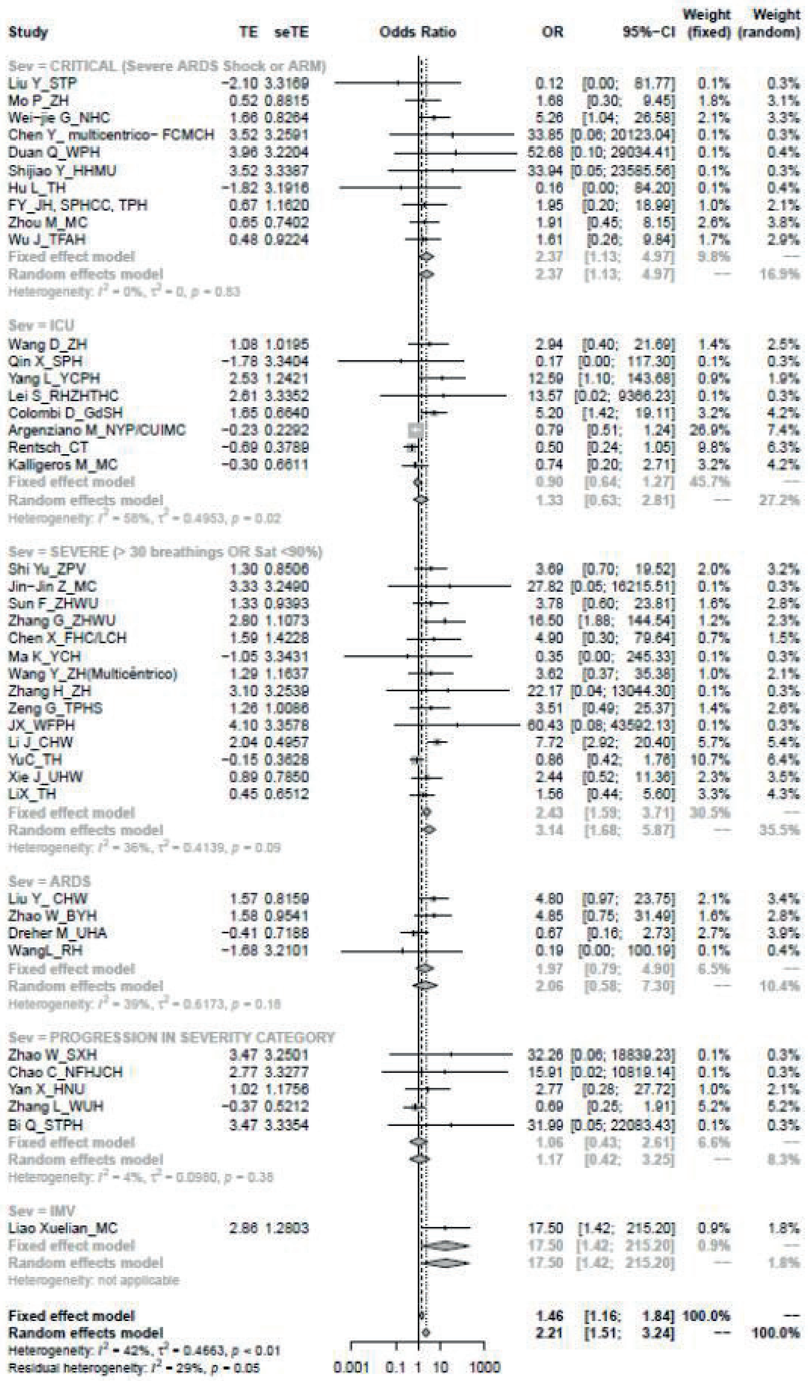

Candidate variable: Cancer (solid or active haematologic cancer), outcome: severe COVID-19 disease, subgroup analysis by COVID-19 severity definition

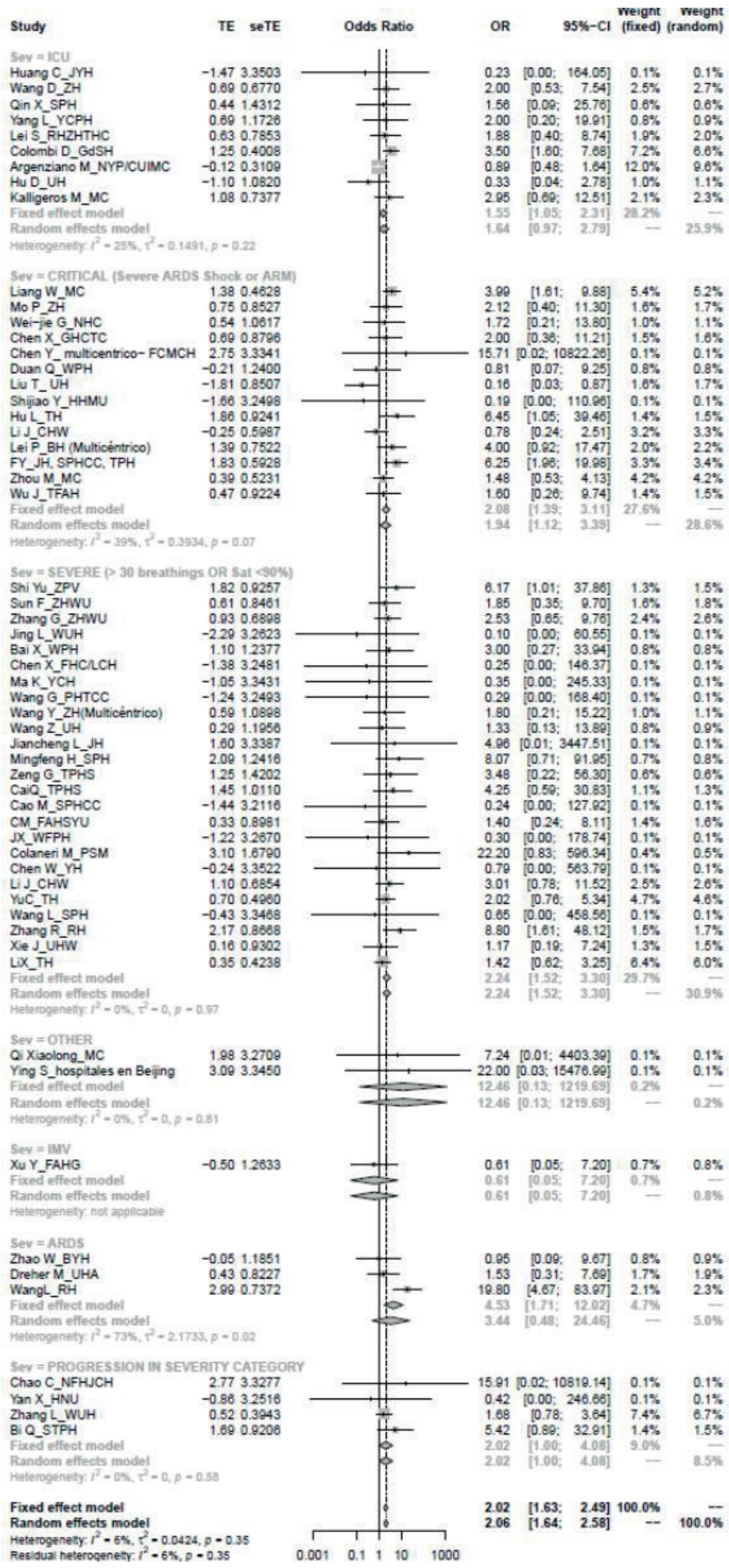

Candidate variable: Chronic liver disease, outcome: severe COVID-19 disease, subgroup analysis by COVID-19 severity definition

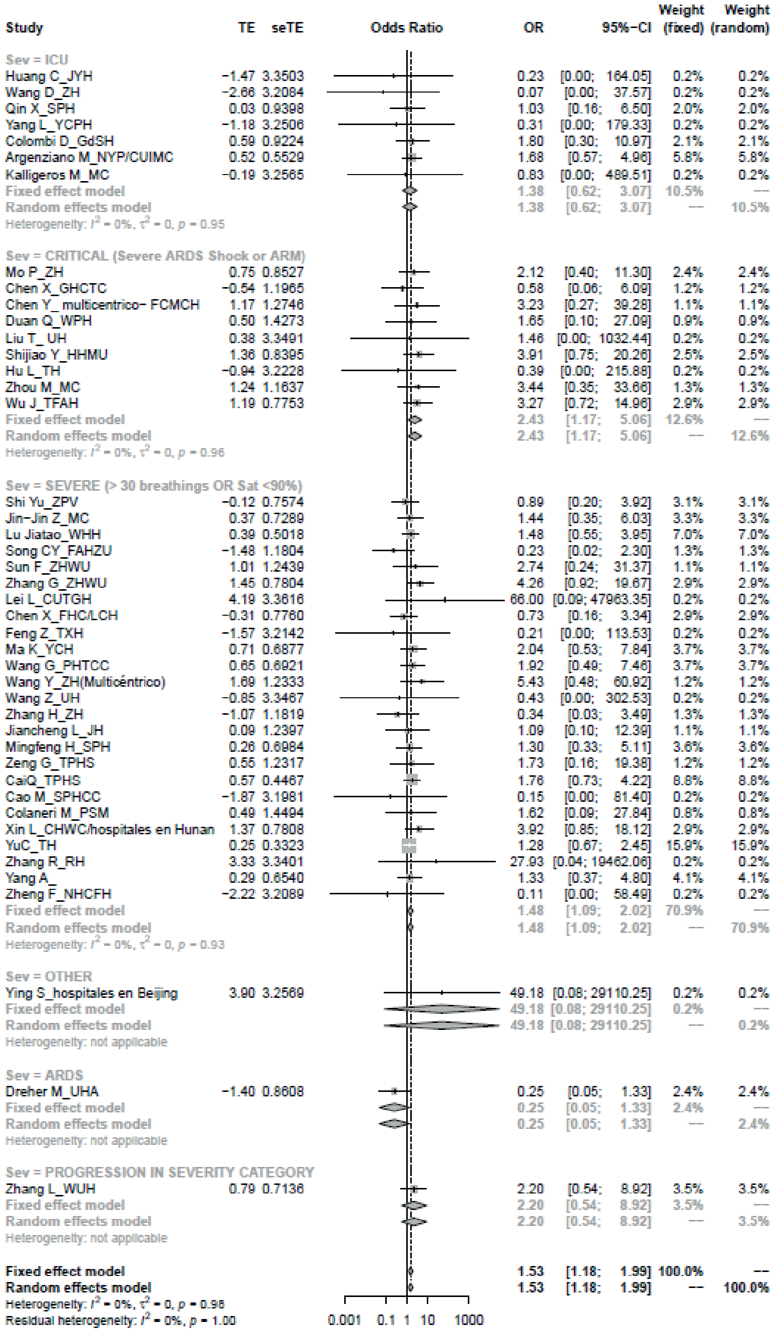

Candidate variable: Chronic gastric disease: History of peptic ulcer or gastritis, outcome: severe COVID-19 disease, subgroup analysis by COVID-19 severity definition

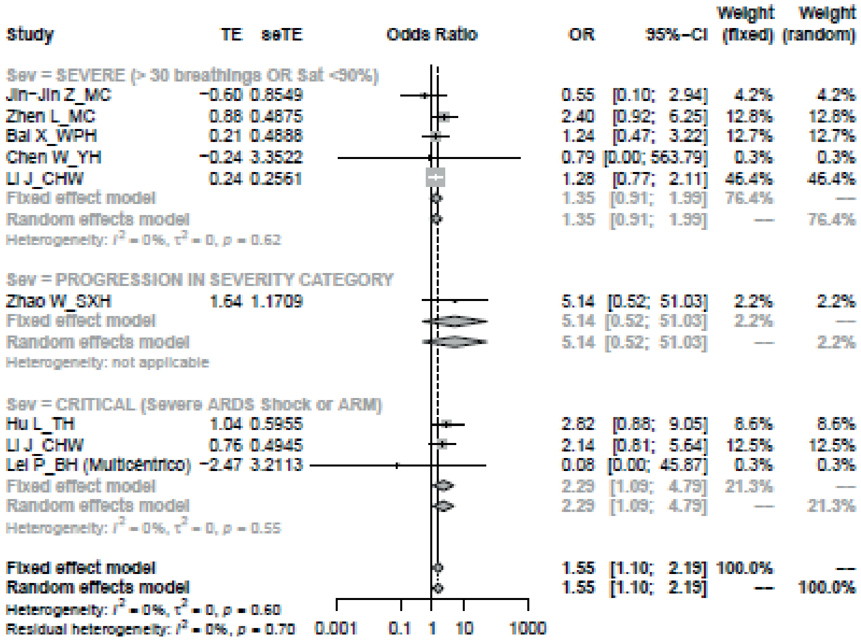

Candidate variable: Tachypnea, outcome: severe COVID-19 disease, subgroup analysis by COVID-19 severity definition

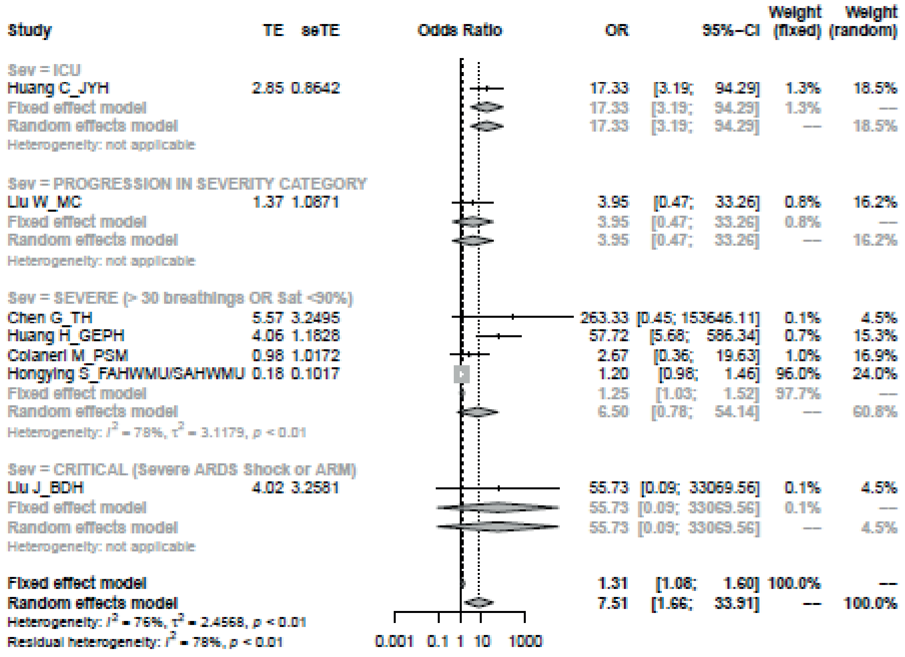

Candidate variable: Hypoxemia, outcome: severe COVID-19 disease, subgroup analysis by COVID-19 severity definition

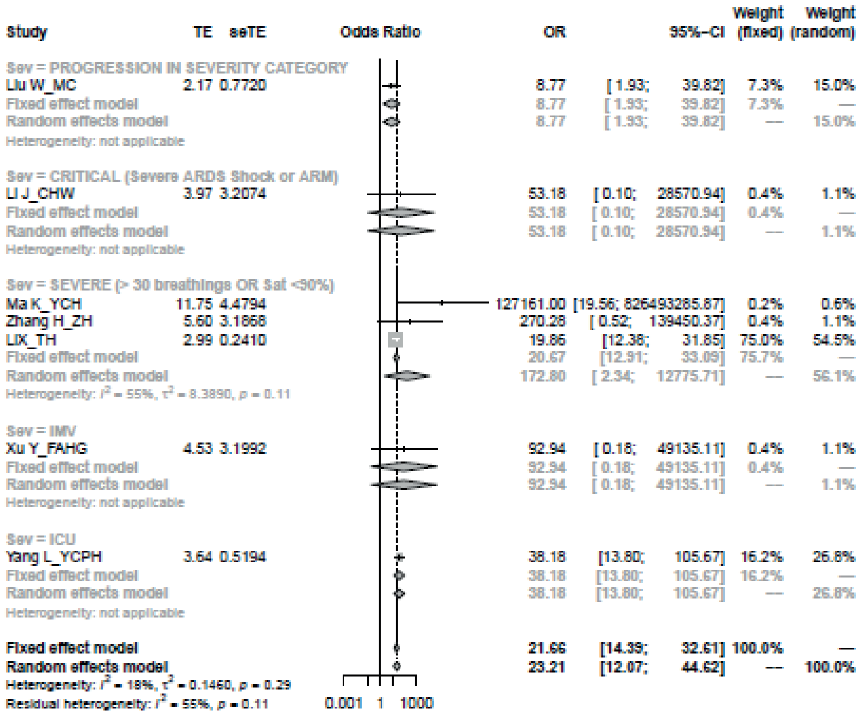

Candidate variable: Dyspnea, outcome: severe COVID-19 disease, subgroup analysis by COVID-19 severity definition

Study	TE	seTE	OR	95%-CI	Weight (fixed)	Weight (random)
Sev = ARDS						
Wu C_WJH	1.45	0.3070	4.26	[2.34; 7.78]	2.8%	2.0%
Zhao W_BYH	1.41	0.5948	4.08	[1.27; 13.10]	0.8%	1.5%
Dreher M_UHA	1.50	0.6067	4.50	[1.37; 14.78]	0.7%	1.5%
Fixed effect model			4.27	[2.62; 6.95]	4.3%	—
Random effects model			4.27	[2.62; 6.95]	—	4.9%
Heterogeneity: $I^2 = 0\%$, $\tau^2 = 0$, $p = 0.99$						
Sev = ICU						
Huang C_JYH	3.02	1.1145	20.40	[2.30; 181.26]	0.2%	0.8%
Wang D_ZH	1.98	0.4273	7.25	[3.14; 16.76]	1.5%	1.8%
Qin X_SPH	7.50	3.1907	1815.75	[3.49; 943956.15]	0.0%	0.1%
Yang L_YCPH	4.30	0.5807	73.63	[23.59; 229.82]	0.8%	1.5%
Lei S_RHZHTHC	1.18	0.7220	3.25	[0.79; 13.38]	0.5%	1.3%
Colombi D_GdSH	0.64	0.2803	1.90	[1.10; 3.29]	3.4%	2.0%
Chen J_FAH	2.67	3.2586	14.44	[0.02; 8576.37]	0.0%	0.1%
Argenziano M_NYP/CUIMC	−0.19	0.1827	0.83	[0.58; 1.18]	8.0%	2.2%
Zheng X_FAH	0.67	1.1880	1.96	[0.19; 20.07]	0.2%	0.7%
Fixed effect model			1.80	[1.38; 2.35]	14.6%	—
Random effects model			6.00	[1.90; 18.89]	—	10.5%
Heterogeneity: $I^2 = 90\%$, $\tau^2 = 2.0862$, $p < 0.01$						
Sev = CRITICAL (Severe ARDS Shock or ARM)						
Li K_CMU	2.39	0.8463	10.89	[2.07; 57.20]	0.4%	1.1%
Mo P_ZH	−0.42	0.5168	0.66	[0.24; 1.82]	1.0%	1.6%
Peng YD_WU	0.69	0.7224	1.98	[0.48; 8.18]	0.5%	1.3%
Wei−jie G_NHC	1.78	0.2591	5.93	[3.57; 9.85]	4.0%	2.1%
Chen Y_ multicentrico− FCMCH	1.63	0.7325	5.10	[1.21; 21.43]	0.5%	1.3%
Chu J_TH	−2.04	0.9913	0.13	[0.02; 0.91]	0.3%	0.9%
Duan Q_WPH	0.22	0.7889	1.24	[0.27; 5.84]	0.4%	1.2%
Liu T_ UH	0.88	0.6725	2.41	[0.65; 9.02]	0.6%	1.3%
Ying W_ MC	2.26	0.5869	9.60	[3.04; 30.33]	0.8%	1.5%
Shijiao Y_HHMU	2.35	0.8602	10.48	[1.94; 56.59]	0.4%	1.1%
Hu L_TH	1.20	0.5597	3.32	[1.11; 9.93]	0.9%	1.5%
Liu J_BDH	4.51	3.2309	91.14	[0.16; 51268.97]	0.0%	0.1%
Xu Y_GH	1.99	1.3623	7.33	[0.51; 105.91]	0.1%	0.6%
FY_JH, SPHCC, TPH	2.46	0.2891	11.73	[6.66; 20.68]	3.2%	2.0%
Zhou M_MC	0.68	0.3026	1.97	[1.09; 3.57]	2.9%	2.0%
Fixed effect model			4.15	[3.22; 5.35]	15.9%	—
Random effects model			3.38	[1.89; 6.07]	—	19.5%
Heterogeneity: $I^2 = 75\%$, $\tau^2 = 0.8218$, $p < 0.01$						
Sev = PROGRESSION IN SEVERITY CATEGORY						
Liu F_XH	2.20	10.5597	9.00	[0.00; 8763252695.66]	0.0%	0.0%
Zhao W_SXH	2.70	3.3387	14.93	[0.02; 10376.38]	0.0%	0.1%
Chao C_NFHJCH	0.77	0.7333	2.15	[0.51; 9.06]	0.5%	1.2%
Wang X_DFH	0.73	0.2234	2.07	[1.33; 3.20]	5.3%	2.1%
Yan X_HNU	2.56	0.4816	12.92	[5.03; 33.21]	1.2%	1.7%
Zhang L_WUH	0.75	0.1928	2.11	[1.45; 3.08]	7.2%	2.1%
Bi Q_STPH	2.05	0.6082	7.76	[2.36; 25.55]	0.7%	1.5%
Jia M_RHWU	2.01	0.8756	7.50	[1.35; 41.72]	0.3%	1.0%
Fixed effect model			2.64	[2.04; 3.42]	15.3%	—
Random effects model			3.80	[2.12; 6.81]	—	9.8%
Heterogeneity: $I^2 = 62\%$, $\tau^2 = 0.3062$, $p = 0.01$						
Sev = SEVERE (> 30 breathings OR Sat <90%)						
Young BE_MC	0.79	1.5135	2.20	[0.11; 42.73]	0.1%	0.5%
Jin−Jin Z_MC	0.67	0.3840	1.94	[0.92; 4.13]	1.8%	1.8%
Lu Jiatao_WHH	0.38	0.3402	1.46	[0.75; 2.84]	2.3%	1.9%
Han Y_ RHWU	0.39	0.6762	1.48	[0.39; 5.58]	0.6%	1.3%
Liu Yo_SCH	3.83	1.1910	46.00	[4.46; 474.81]	0.2%	0.7%
Qi D_multicentrico	1.33	0.3644	3.78	[1.85; 7.73]	2.0%	1.9%
Zhang G_ZHWU	2.11	0.3469	8.27	[4.19; 16.32]	2.2%	1.9%
Zhen L_MC	1.65	0.3294	5.23	[2.74; 9.97]	2.5%	1.9%
Tian S_57 hospitales	3.54	0.6610	34.35	[9.40; 125.50]	0.6%	1.4%
Lei L_CUTGH	3.85	1.1799	46.80	[4.63; 472.73]	0.2%	0.7%
Jing L_WUH	−1.50	3.3505	0.22	[0.00; 158.04]	0.0%	0.1%
Chen G_TH	9.29	4.4936	10791.00	[1.61; 72107467.02]	0.0%	0.1%
Bai X_WPH	1.40	0.4403	4.06	[1.71; 9.62]	1.4%	1.7%
Chen X_FHC/LCH	1.88	0.3794	6.57	[3.12; 13.82]	1.9%	1.9%
Ma K_YCH	0.49	1.2525	1.63	[0.14; 19.00]	0.2%	0.7%
Wang G_PHTCC	2.35	0.4894	10.45	[4.01; 27.28]	1.1%	1.7%
Wang Y_ZH(Multicéntrico)	1.69	0.5164	5.44	[1.98; 14.97]	1.0%	1.6%
Wang Z_UH	1.17	0.6216	3.23	[0.96; 10.93]	0.7%	1.4%
Zhang H_ZH	2.30	0.6397	9.96	[2.84; 34.91]	0.7%	1.4%
Jiancheng L_JH	1.03	0.3935	2.80	[1.29; 6.05]	1.7%	1.8%
Cao M_SPHCC	3.24	0.6942	25.52	[6.55; 99.49]	0.6%	1.3%
Wang Y_CHW	0.46	0.4211	1.58	[0.69; 3.62]	1.5%	1.8%
CM_FAHSYU	7.77	3.1932	2373.43	[4.54; 1240057.68]	0.0%	0.1%
FL_GHCTCPLA	2.04	0.7393	7.70	[1.81; 32.81]	0.5%	1.2%
JX_WFPH	7.07	3.2374	1170.67	[2.05; 666974.12]	0.0%	0.1%
MY_multicenter 43 hosp	1.76	0.2821	5.83	[3.35; 10.14]	3.4%	2.0%
Colaneri M_PSM	0.07	0.7357	1.08	[0.25; 4.55]	0.5%	1.2%
Xin L_CHWC/hospitales en Hunan	1.97	0.3783	7.14	[3.40; 14.98]	1.9%	1.9%
Chen W_YH	5.75	3.2445	314.17	[0.54; 181487.08]	0.0%	0.1%
Minhua Y_ZHWU	7.49	3.2050	1796.00	[3.36; 960262.37]	0.0%	0.1%
Xie H_WJH	0.41	0.4738	1.50	[0.59; 3.80]	1.2%	1.7%
YuC_TH	0.29	0.2431	1.34	[0.83; 2.16]	4.5%	2.1%
Zhang R_RH	3.44	0.7987	31.10	[6.50; 148.80]	0.4%	1.2%
Yang J_WUH	0.83	0.5407	2.29	[0.79; 6.60]	0.9%	1.6%
Xie J_UHW	0.99	0.3640	2.70	[1.32; 5.51]	2.0%	1.9%
ZhangX_MC	2.20	0.7201	9.02	[2.20; 37.00]	0.5%	1.3%
Zheng F_NHCFH	1.28	0.4886	3.58	[1.37; 9.33]	1.1%	1.7%
LiX_TH	1.43	0.1845	4.16	[2.90; 5.97]	7.8%	2.1%
Li Y_TH	0.98	1.2076	2.67	[0.25; 28.44]	0.2%	0.7%
Fixed effect model			3.87	[3.34; 4.47]	48.2%	—
Random effects model			4.61	[3.41; 6.23]	—	50.7%
Heterogeneity: $I^2 = 68\%$, $\tau^2 = 0.4797$, $p < 0.01$						
Sev = IMV						
Xu Y_FAHG	0.86	0.6550	2.36	[0.65; 8.51]	0.6%	1.4%
Liao Xuelian_MC	−0.05	0.7362	0.95	[0.23; 4.04]	0.5%	1.2%
Fixed effect model			1.58	[0.61; 4.13]	1.1%	—
Random effects model			1.58	[0.61; 4.13]	—	2.6%
Heterogeneity: $I^2 = 0\%$, $\tau^2 = 0$, $p = 0.36$						
Sev = OTHER						
Wentao X_IDH	−0.34	0.7958	0.71	[0.15; 3.39]	0.4%	1.2%
Ying S_hospitales en Beijing	3.87	1.1110	47.78	[5.41; 421.61]	0.2%	0.8%
Fixed effect model			2.97	[0.84; 10.55]	0.6%	—
Random effects model			5.44	[0.09; 333.84]	—	1.9%
Heterogeneity: $I^2 = 89\%$, $\tau^2 = 7.9040$, $p < 0.01$						
**Fixed effect model**			**3.28**	**[2.96; 3.62]**	**100.0%**	**—**
**Random effects model**			**4.23**	**[3.33; 5.38]**	**—**	**100.0%**
Heterogeneity: $I^2 = 76\%$, $\tau^2 = 0.6653$, $p < 0.01$						
Residual heterogeneity: $I^2 = 75\%$, $p < 0.01$						

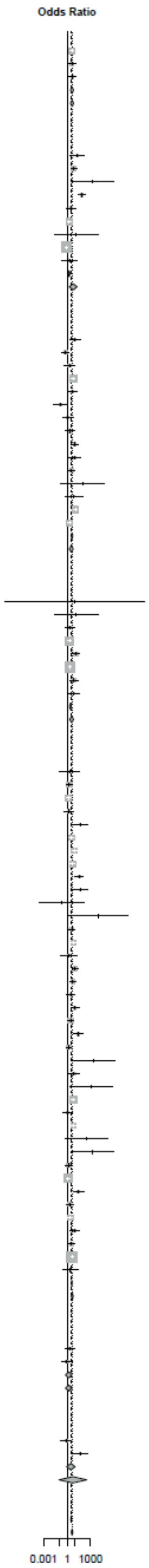

Candidate variable: Chest pain, outcome: severe COVID-19 disease, subgroup analysis by COVID-19 severity definition

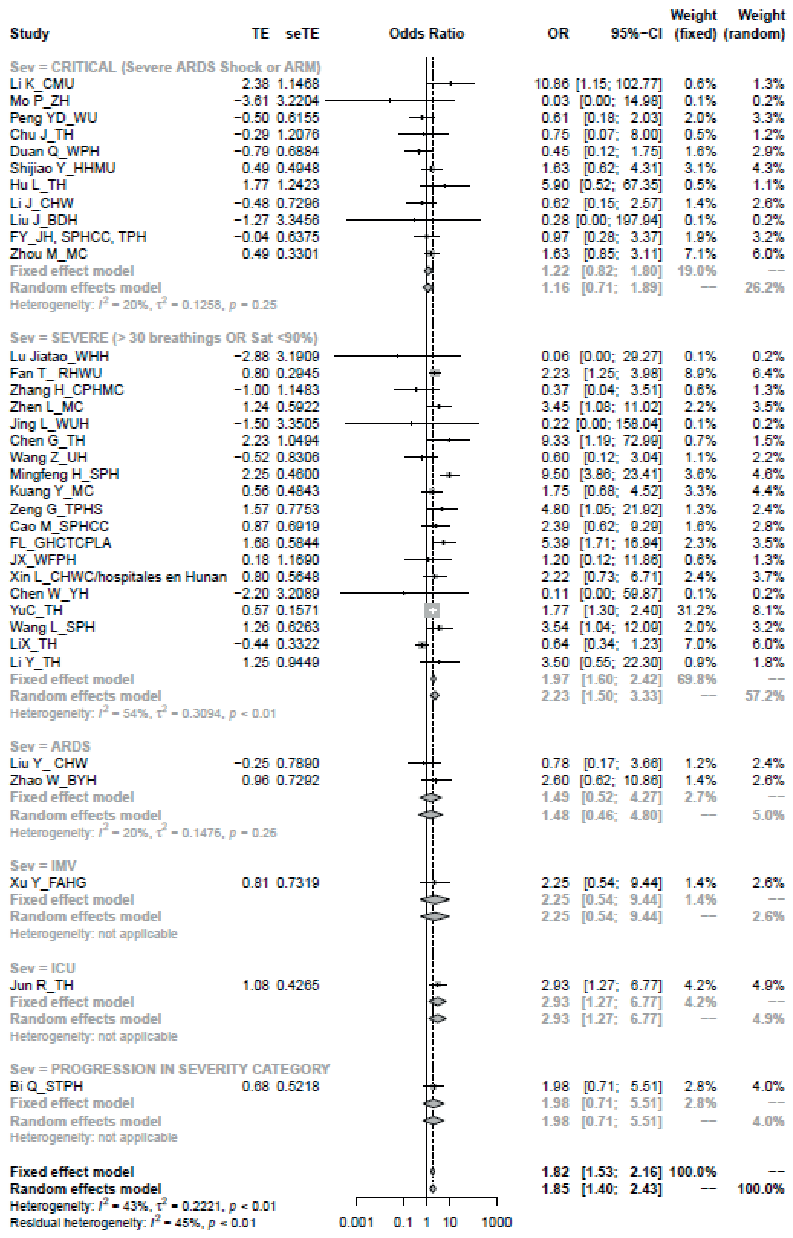

Candidate variable: High fever (more than 39°C), outcome: severe COVID-19 disease, subgroup analysis by COVID-19 severity definition

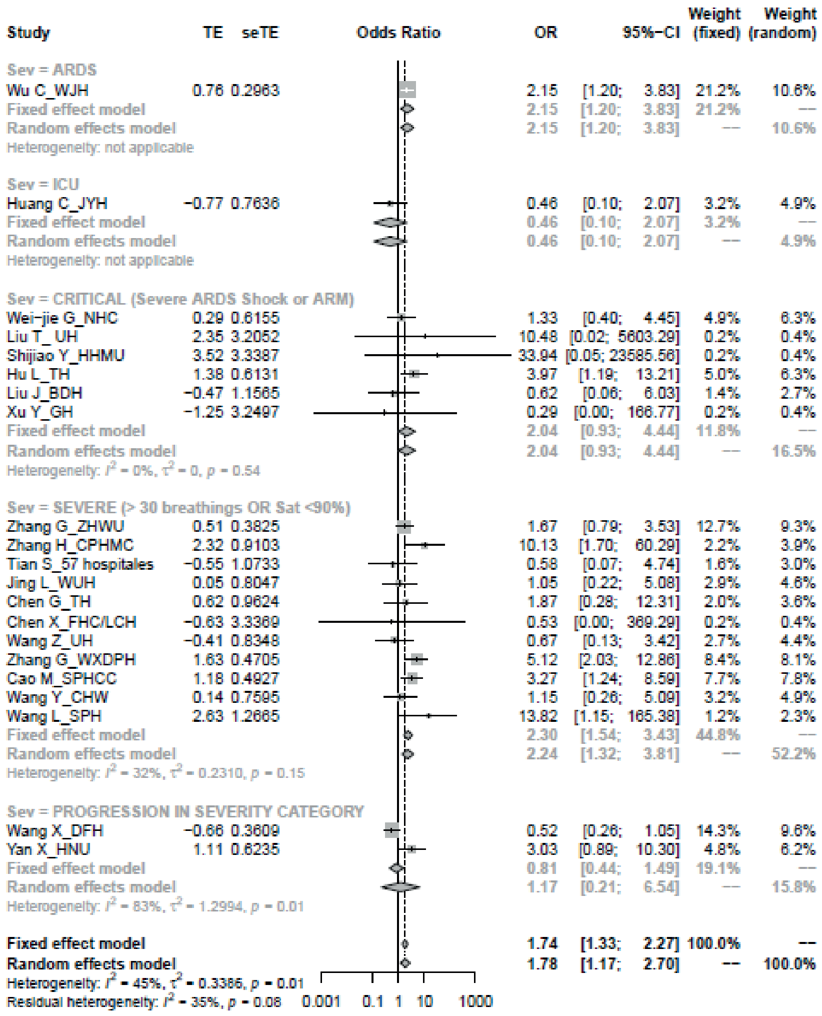

Candidate variable: Fever, outcome: severe COVID-19 disease, subgroup analysis by COVID-19 severity definition

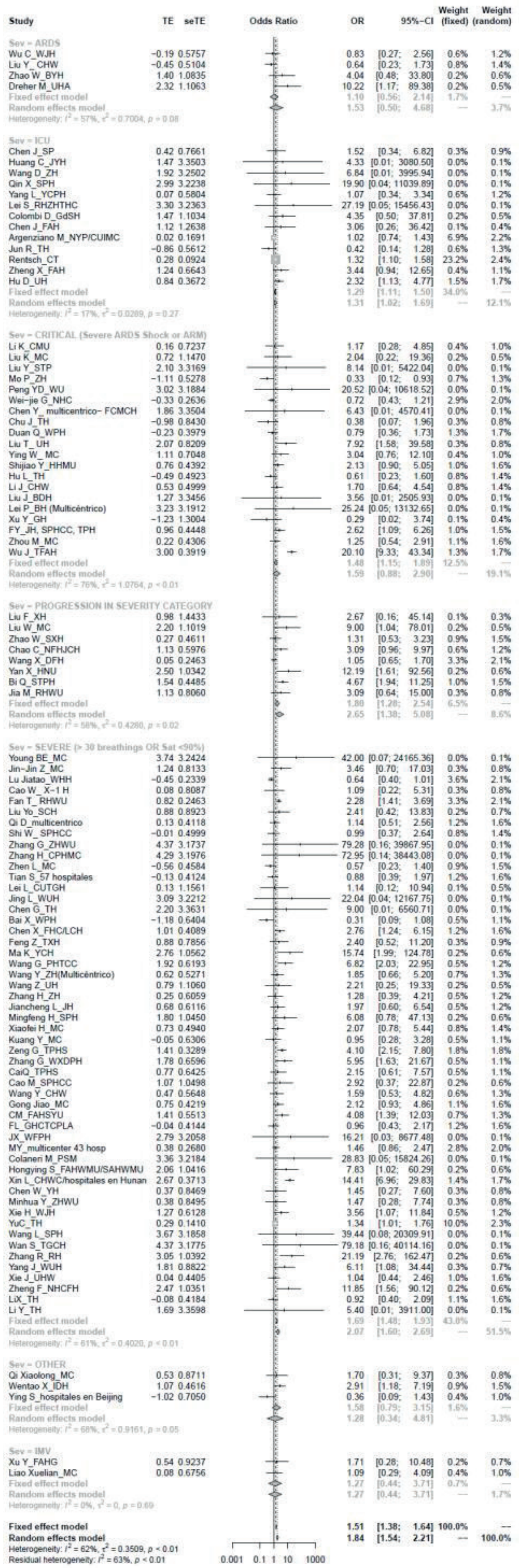

Candidate variable: Rhinorrhea, outcome: severe COVID-19 disease, subgroup analysis by COVID-19 severity definition

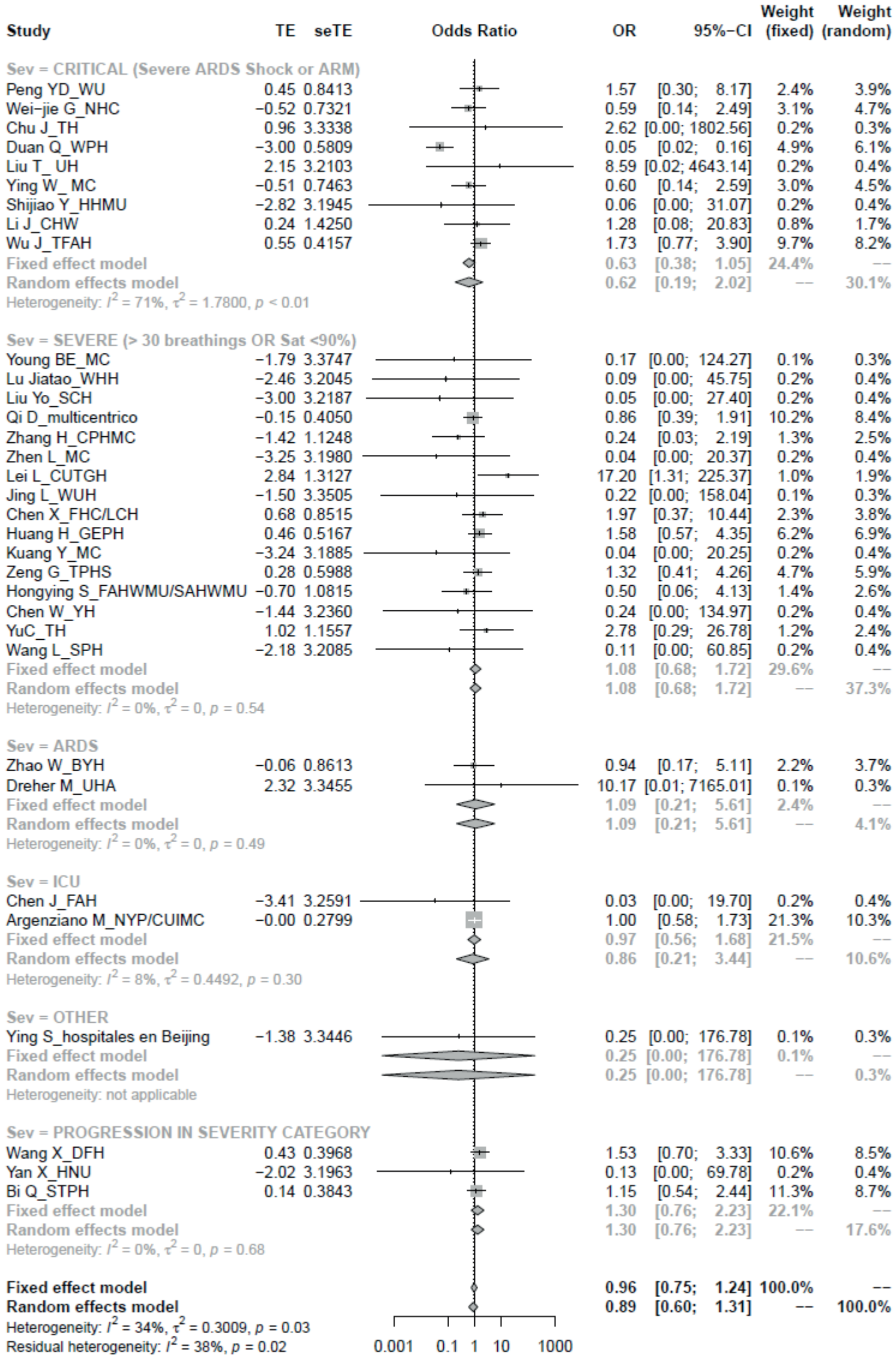

Candidate variable: Cough, outcome: severe COVID-19 disease, subgroup analysis by COVID-19 severity definition

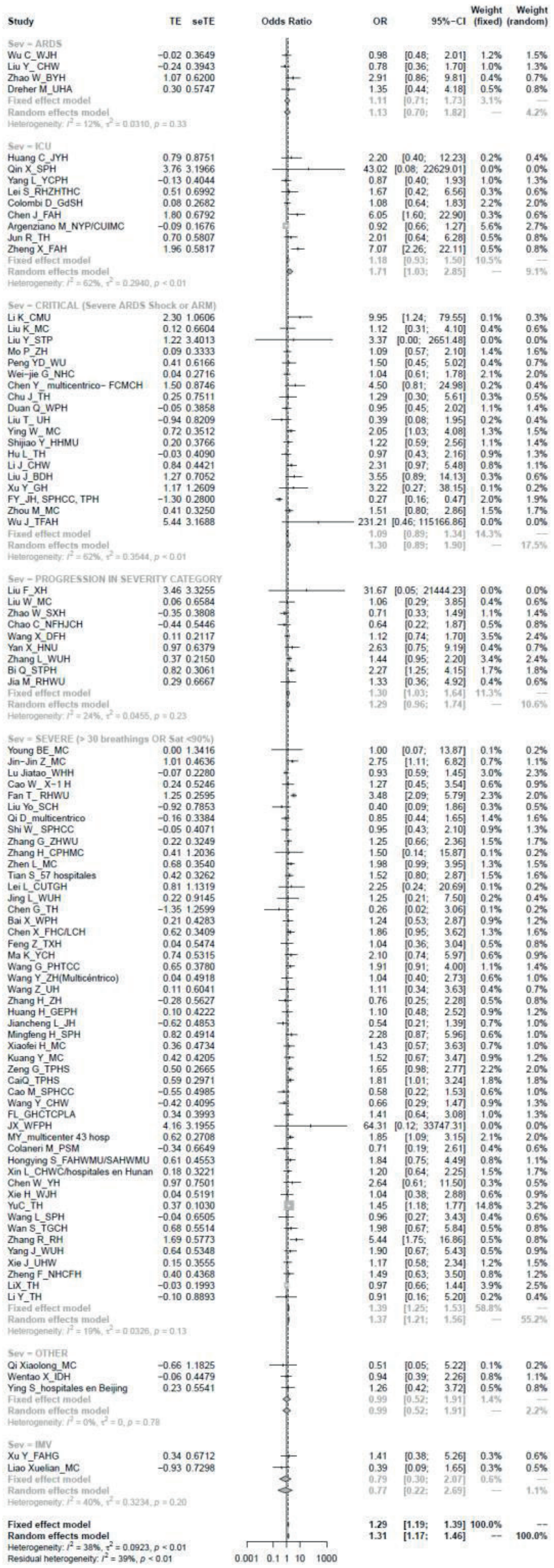

Candidate variable: Fatigue, outcome: severe COVID-19 disease, subgroup analysis by COVID-19 severity definition

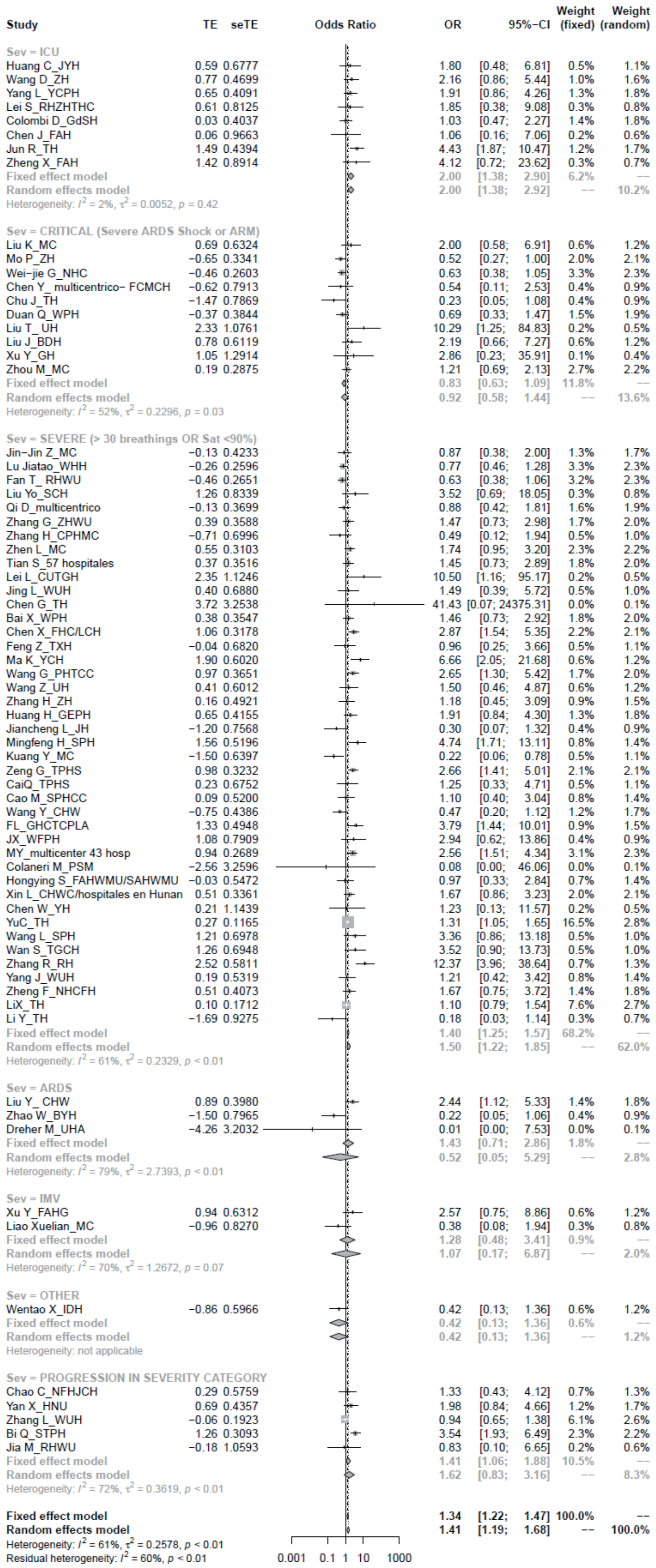

Candidate variable: Diarrhea, outcome: severe COVID-19 disease, subgroup analysis by COVID-19 severity definition

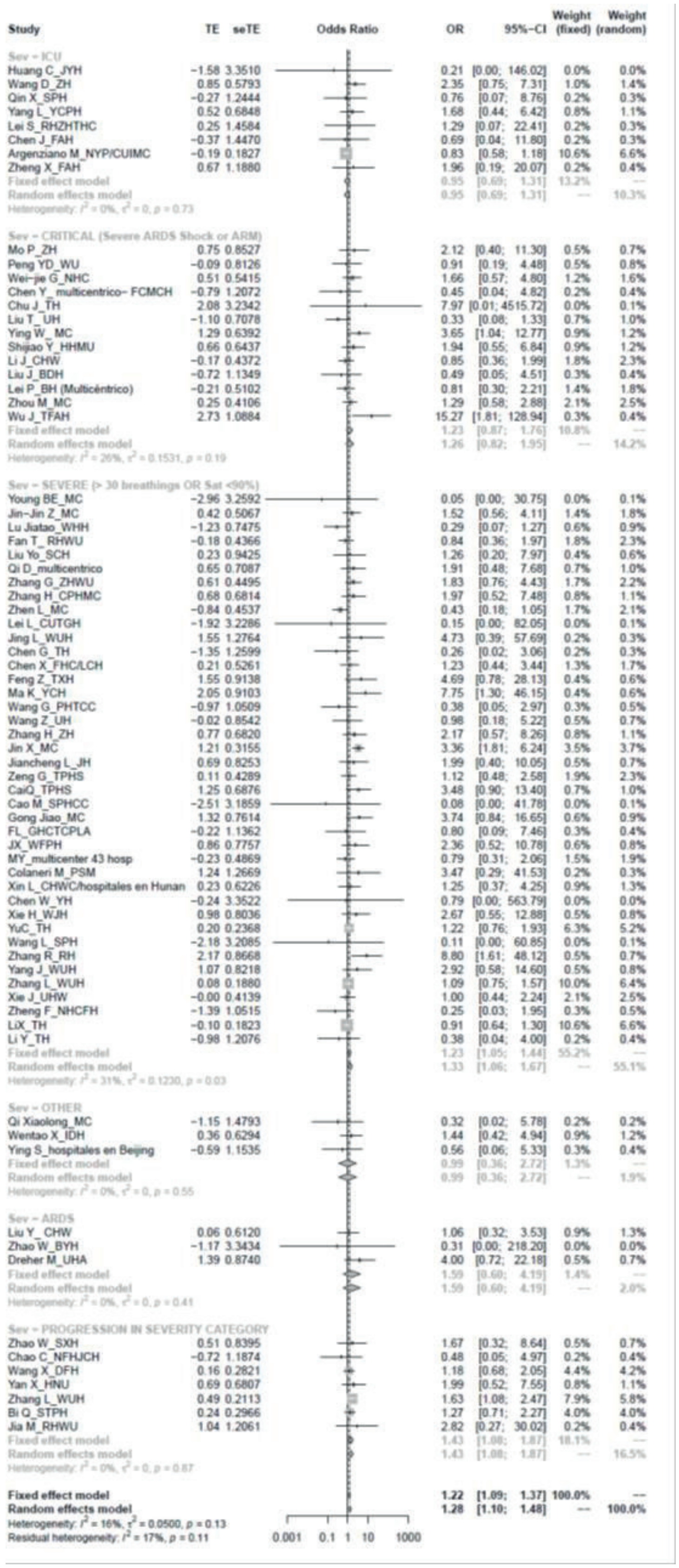

Candidate variable: Anemia, outcome: severe COVID-19 disease, subgroup analysis by COVID-19 severity definition

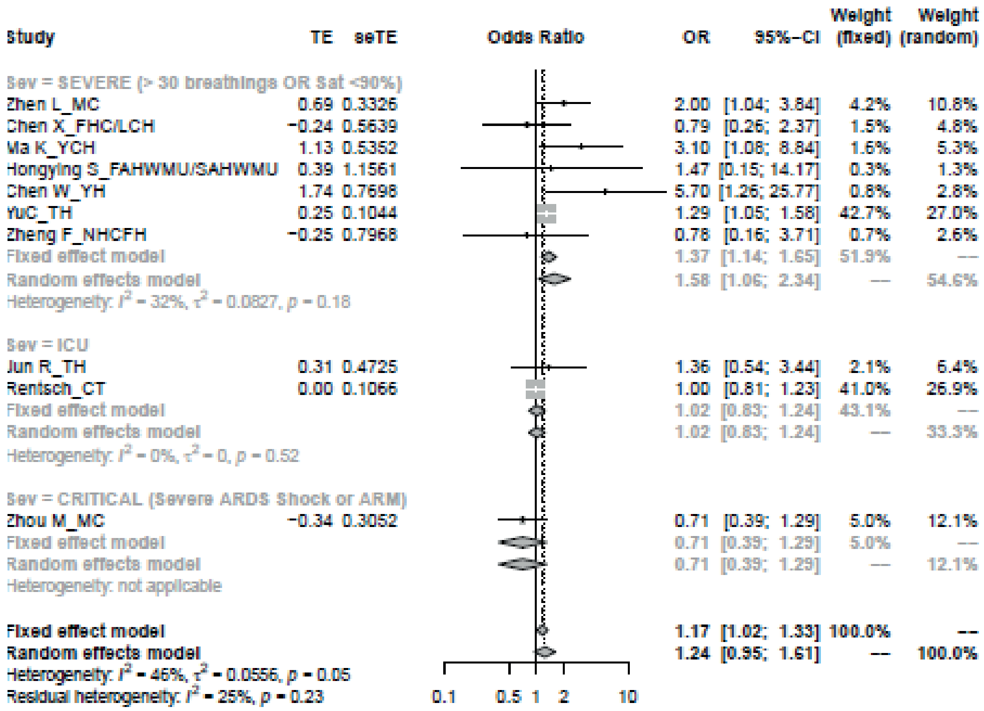

Candidate variable: Neutrophil count increase (per 1 x 10^9 U/L), outcome: severe COVID-19 disease, subgroup analysis by COVID-19 severity definition

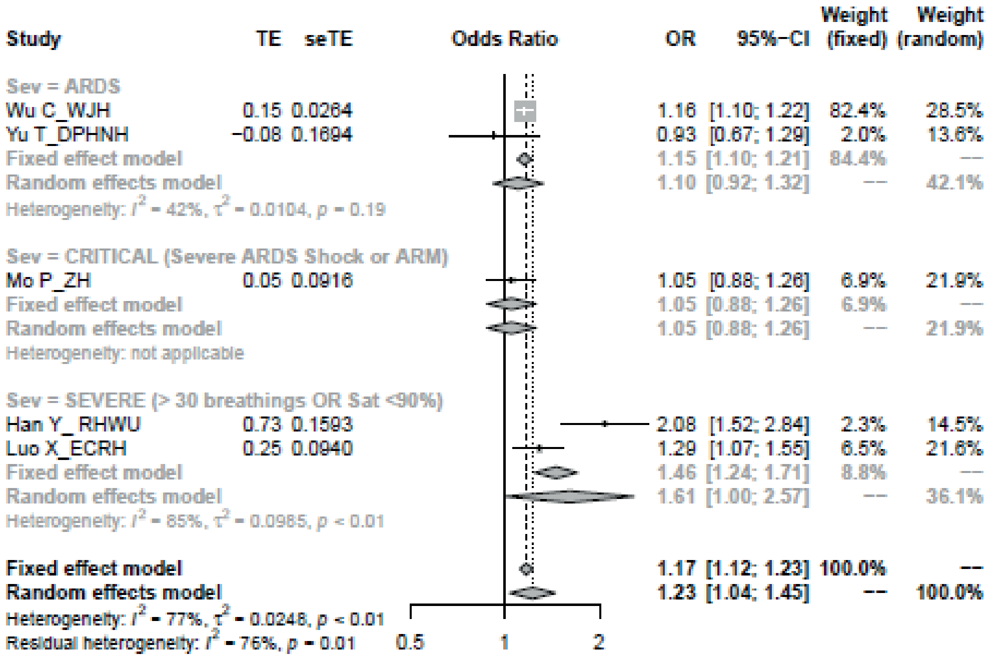

Candidate variable: Low neutrophil count (Less than 1.8 x 10^9/L), outcome: severe COVID-19 disease, subgroup analysis by COVID-19 severity definition

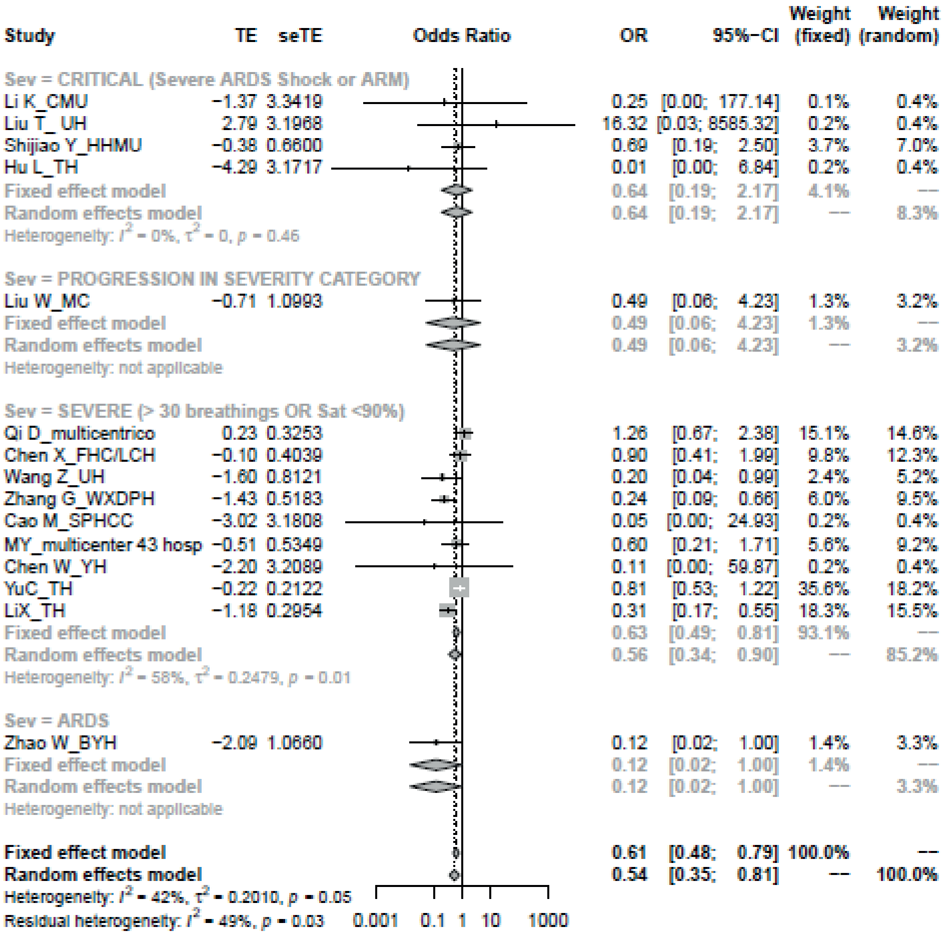

Candidate variable: Leukopenia (Less than 3.5-4 x 10⁹/L), outcome: severe COVID-19 disease, subgroup analysis by COVID-19 severity definition

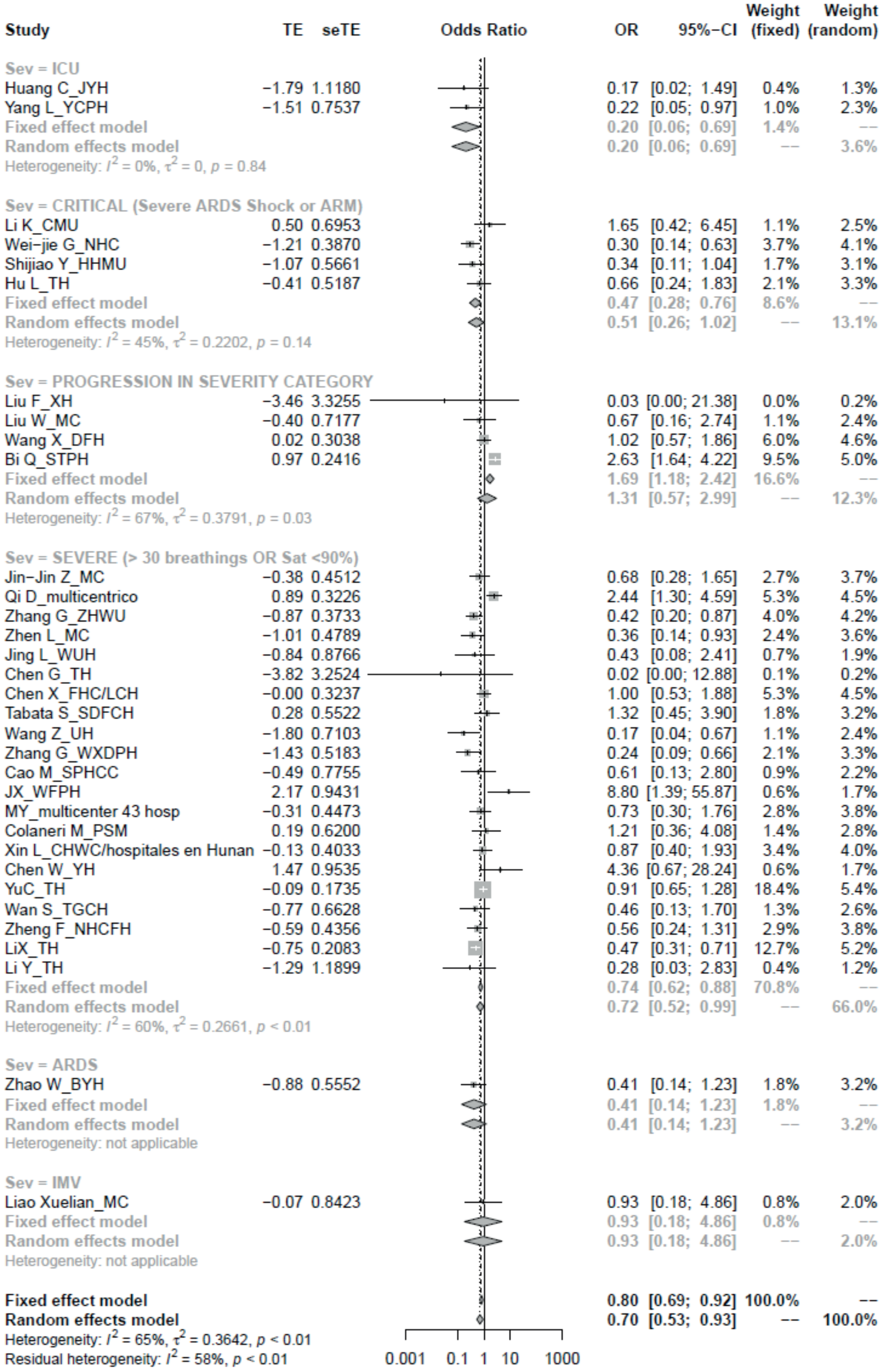

Candidate variable: Low lymphocyte count (less than 0.8-1.5x 10^9/L), outcome: severe COVID-19 disease, subgroup analysis by COVID-19 severity definition

Study	TE	seTE	OR	95%-CI	Weight (fixed)	Weight (random)
**Sev = ICU**						
Huang C_JYH	1.56	0.8570	4.77	[0.89; 25.57]	0.4%	0.9%
Yang L_YCPH	1.19	0.5620	3.29	[1.09; 9.91]	1.0%	1.8%
Lei S_RHZHTHC	1.32	1.1782	3.73	[0.37; 37.58]	0.2%	0.5%
Colombi D_GdSH	0.83	0.2736	2.30	[1.35; 3.93]	4.1%	4.1%
Jun R_TH	0.99	0.4683	2.68	[1.07; 6.71]	1.4%	2.4%
Rentsch_CT	0.97	0.2325	2.65	[1.68; 4.18]	5.6%	4.6%
Fixed effect model			2.64	[1.95; 3.58]	12.6%	--
Random effects model			2.64	[1.95; 3.58]	--	14.4%
Heterogeneity: $I^2 = 0\%$, $\tau^2 = 0$, $p = 0.96$						
**Sev = CRITICAL (Severe ARDS Shock or ARM)**						
Li K_CMU	2.03	0.5712	7.60	[2.48; 23.28]	0.9%	1.8%
Wei−jie G_NHC	0.97	0.5276	2.64	[0.94; 7.43]	1.1%	2.0%
Liu T_ UH	1.55	0.6755	4.71	[1.25; 17.72]	0.7%	1.4%
Shijiao Y_HHMU	1.15	0.3994	3.17	[1.45; 6.93]	1.9%	2.9%
Hu L_TH	2.27	0.7476	9.69	[2.24; 41.92]	0.5%	1.2%
Xu Y_GH	1.76	1.2696	5.83	[0.48; 70.24]	0.2%	0.5%
FY_JH, SPHCC, TPH	1.19	0.2829	3.30	[1.90; 5.75]	3.8%	4.0%
Zhou M_MC	1.29	0.2811	3.65	[2.10; 6.33]	3.8%	4.0%
Fixed effect model			3.78	[2.80; 5.11]	12.9%	--
Random effects model			3.78	[2.80; 5.11]	--	17.8%
Heterogeneity: $I^2 = 0\%$, $\tau^2 = 0$, $p = 0.76$						
**Sev = PROGRESSION IN SEVERITY CATEGORY**						
Liu F_XH	0.00	1.5811	1.00	[0.05; 22.17]	0.1%	0.3%
Liu W_MC	0.47	0.6689	1.60	[0.43; 5.94]	0.7%	1.4%
Dong J_FMC	1.50	0.5053	4.50	[1.67; 12.11]	1.2%	2.1%
Bi Q_STPH	0.88	0.2837	2.42	[1.39; 4.22]	3.8%	4.0%
Fixed effect model			2.57	[1.64; 4.03]	5.8%	--
Random effects model			2.57	[1.64; 4.03]	--	7.9%
Heterogeneity: $I^2 = 0\%$, $\tau^2 = 0$, $p = 0.55$						
**Sev = SEVERE (> 30 breathings OR Sat <90%)**						
Jin−Jin Z_MC	0.64	0.4250	1.90	[0.83; 4.38]	1.7%	2.7%
Xiao M_ECRH	1.24	0.2092	3.45	[2.29; 5.20]	6.9%	4.9%
Qi D_multicentrico	0.69	0.5553	1.99	[0.67; 5.91]	1.0%	1.9%
Zhang G_ZHWU	1.11	0.4382	3.04	[1.29; 7.18]	1.6%	2.6%
Zhen L_MC	1.08	0.3778	2.95	[1.40; 6.18]	2.1%	3.1%
Jing L_WUH	1.58	0.7660	4.85	[1.08; 21.76]	0.5%	1.1%
Chen G_TH	3.18	1.2528	24.00	[2.06; 279.61]	0.2%	0.5%
Chen X_FHC/LCH	1.43	0.3304	4.18	[2.19; 7.98]	2.8%	3.5%
Tabata S_SDFCH	1.46	0.5652	4.30	[1.42; 13.02]	0.9%	1.8%
Wang Z_UH	2.05	0.7147	7.76	[1.91; 31.51]	0.6%	1.3%
Kuang Y_MC	1.06	0.4128	2.89	[1.29; 6.50]	1.8%	2.8%
Zhang G_WXDPH	2.48	0.8191	11.93	[2.40; 59.44]	0.5%	1.0%
Cao M_SPHCC	6.83	1.1839	922.67	[90.64; 9392.08]	0.2%	0.5%
JX_WFPH	1.96	0.8766	7.07	[1.27; 39.41]	0.4%	0.9%
MY_multicenter 43 hosp	1.11	0.2694	3.05	[1.80; 5.16]	4.2%	4.1%
Colaneri M_PSM	3.63	3.2106	37.86	[0.07; 20468.87]	0.0%	0.1%
Hongying S_FAHWMU/SAHWMU	0.92	1.3102	2.50	[0.19; 32.59]	0.2%	0.4%
Xin L_CHWC/hospitales en Hunan	1.78	0.3858	5.93	[2.78; 12.64]	2.0%	3.0%
Chen W_YH	6.68	3.2356	796.25	[1.40; 452057.58]	0.0%	0.1%
Fei J_UHHUST	1.27	0.3092	3.57	[1.95; 6.54]	3.2%	3.7%
YuC_TH	0.61	0.1040	1.84	[1.50; 2.26]	28.1%	6.0%
Wan S_TGCH	2.19	0.6555	8.93	[2.47; 32.26]	0.7%	1.5%
Li H_TH	1.04	0.4054	2.82	[1.27; 6.25]	1.8%	2.8%
Zheng F_NHCFH	0.99	0.4243	2.69	[1.17; 6.18]	1.7%	2.7%
LiX_TH	1.34	0.2685	3.81	[2.25; 6.45]	4.2%	4.2%
Li Y_TH	0.13	1.3024	1.14	[0.09; 14.68]	0.2%	0.4%
Fixed effect model			2.76	[2.42; 3.14]	67.5%	--
Random effects model			3.79	[2.87; 5.01]	--	57.5%
Heterogeneity: $I^2 = 63\%$, $\tau^2 = 0.2376$, $p < 0.01$						
**Sev = IMV**						
Xu Y_FAHG	4.53	3.1992	92.94	[0.18; 49135.11]	0.0%	0.1%
Liao Xuelian_MC	0.50	1.1039	1.65	[0.19; 14.36]	0.2%	0.6%
Fixed effect model			2.53	[0.33; 19.59]	0.3%	--
Random effects model			4.05	[0.15; 108.28]	--	0.7%
Heterogeneity: $I^2 = 30\%$, $\tau^2 = 2.3984$, $p = 0.23$						
**Sev = ARDS**						
Zhao W_BYH	1.95	0.5741	7.05	[2.29; 21.72]	0.9%	1.8%
Fixed effect model			7.05	[2.29; 21.72]	0.9%	--
Random effects model			7.05	[2.29; 21.72]	--	1.8%
Heterogeneity: not applicable						
**Fixed effect model**			**2.87**	**[2.58; 3.20]**	**100.0%**	**--**
**Random effects model**			**3.47**	**[2.91; 4.14]**	**--**	**100.0%**
Heterogeneity: $I^2 = 44\%$, $\tau^2 = 0.1235$, $p < 0.01$						
Residual heterogeneity: $I^2 = 46\%$, $p < 0.01$						

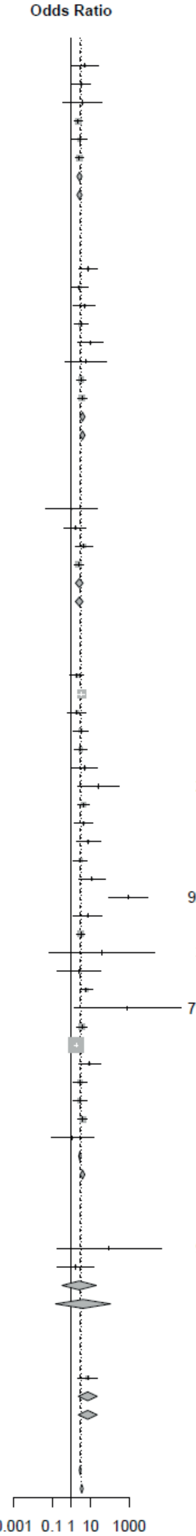

Candidate variable: Low platelet count (less than 100-150 x 10⁹/L), outcome: severe COVID-19 disease, subgroup analysis by COVID-19 severity definition

Study	TE	seTE	OR	95%-CI	Weight (fixed)	Weight (random)
**Sev = ICU**						
Huang C_JYH	0.77	1.4566	2.17	[0.12; 37.64]	0.3%	0.7%
Yang L_YCPH	0.36	0.4395	1.43	[0.60; 3.38]	2.9%	4.1%
Colombi D_GdSH	−1.06	0.3582	0.34	[0.17; 0.70]	4.4%	5.0%
Jun R_TH	0.59	0.4642	1.80	[0.72; 4.47]	2.6%	3.9%
Zhou H_UH	1.07	0.4848	2.91	[1.12; 7.52]	2.4%	3.7%
Fixed effect model			1.05	[0.70; 1.60]	12.5%	--
Random effects model			1.28	[0.52; 3.15]	--	17.4%
Heterogeneity: $I^2 = 75\%$, $\tau^2 = 0.7309$, $p < 0.01$						
**Sev = PROGRESSION IN SEVERITY CATEGORY**						
Liu W_MC	0.82	0.8937	2.26	[0.39; 13.03]	0.7%	1.6%
Bi Q_STPH	1.41	0.3129	4.09	[2.21; 7.54]	5.7%	5.6%
Fixed effect model			3.83	[2.15; 6.83]	6.4%	--
Random effects model			3.83	[2.15; 6.83]	--	7.1%
Heterogeneity: $I^2 = 0\%$, $\tau^2 = 0$, $p = 0.53$						
**Sev = CRITICAL (Severe ARDS Shock or ARM)**						
Wei−jie G_NHC	0.46	0.2733	1.58	[0.93; 2.70]	7.5%	6.1%
Liu T_ UH	3.58	3.1889	35.75	[0.07; 18520.52]	0.1%	0.1%
Shijiao Y_HHMU	1.44	0.6642	4.23	[1.15; 15.56]	1.3%	2.5%
Hu L_TH	1.15	0.6792	3.14	[0.83; 11.90]	1.2%	2.4%
Zhou M_MC	1.00	0.2819	2.71	[1.56; 4.71]	7.1%	6.0%
Fixed effect model			2.25	[1.58; 3.21]	17.1%	--
Random effects model			2.25	[1.58; 3.21]	--	17.0%
Heterogeneity: $I^2 = 0\%$, $\tau^2 = 0$, $p = 0.41$						
**Sev = SEVERE (> 30 breathings OR Sat <90%)**						
Xiao M_ECRH	1.08	0.3129	2.93	[1.59; 5.41]	5.7%	5.6%
Qi D_multicentrico	0.94	0.3382	2.55	[1.32; 4.96]	4.9%	5.2%
Zhen L_MC	0.80	0.3655	2.23	[1.09; 4.57]	4.2%	4.9%
Jing L_WUH	0.37	0.9828	1.45	[0.21; 9.98]	0.6%	1.3%
Chen G_TH	−2.40	3.3634	0.09	[0.00; 66.31]	0.0%	0.1%
Chen X_FHC/LCH	0.47	0.3214	1.60	[0.85; 3.00]	5.4%	5.5%
Tabata S_SDFCH	−0.11	0.8482	0.90	[0.17; 4.73]	0.8%	1.7%
Zhang G_WXDPH	0.56	0.6490	1.76	[0.49; 6.28]	1.3%	2.5%
Cao M_SPHCC	0.88	0.5349	2.41	[0.84; 6.87]	2.0%	3.3%
JX_WFPH	1.88	1.4712	6.57	[0.37; 117.48]	0.3%	0.6%
Colaneri M_PSM	0.05	1.2046	1.05	[0.10; 11.13]	0.4%	0.9%
Hongying S_FAHWMU/SAHWMU	4.89	3.2236	132.73	[0.24; 73598.18]	0.1%	0.1%
Chen W_YH	4.29	3.3543	73.12	[0.10; 52386.57]	0.0%	0.1%
YuC_TH	0.19	0.1851	1.21	[0.84; 1.74]	16.4%	7.2%
Wan S_TGCH	1.23	0.5499	3.42	[1.16; 10.06]	1.9%	3.2%
Li H_TH	−0.41	0.5116	0.66	[0.24; 1.80]	2.1%	3.5%
Zheng F_NHCFH	0.54	0.7096	1.71	[0.43; 6.86]	1.1%	2.2%
LiX_TH	0.43	0.1910	1.53	[1.05; 2.23]	15.4%	7.2%
Fixed effect model			1.66	[1.38; 2.00]	62.5%	--
Random effects model			1.72	[1.38; 2.15]	--	55.3%
Heterogeneity: $I^2 = 13\%$, $\tau^2 = 0.0269$, $p = 0.30$						
**Sev = ARDS**						
Zhao W_BYH	1.58	0.9541	4.85	[0.75; 31.49]	0.6%	1.4%
Fixed effect model			4.85	[0.75; 31.49]	0.6%	--
Random effects model			4.85	[0.75; 31.49]	--	1.4%
Heterogeneity: not applicable						
**Sev = IMV**						
Liao Xuelian_MC	2.12	0.8302	8.35	[1.64; 42.49]	0.8%	1.8%
Fixed effect model			8.35	[1.64; 42.49]	0.8%	--
Random effects model			8.35	[1.64; 42.49]	--	1.8%
Heterogeneity: not applicable						
**Fixed effect model**			**1.78**	**[1.54; 2.06]**	**100.0%**	**--**
**Random effects model**			**1.93**	**[1.52; 2.46]**	**--**	**100.0%**
Heterogeneity: $I^2 = 48\%$, $\tau^2 = 0.1751$, $p < 0.01$						
Residual heterogeneity: $I^2 = 35\%$, $p = 0.04$						

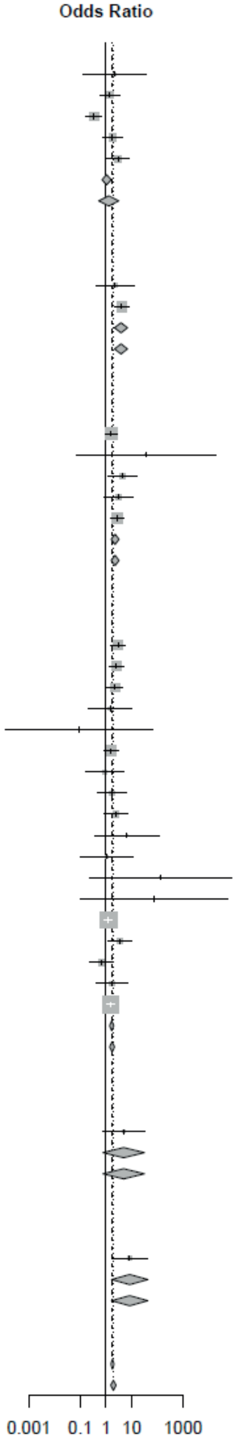

Candidate variable: High plasma creatinine (more than 1.5 mg%), outcome: severe COVID-19 disease, subgroup analysis by COVID-19 severity definition

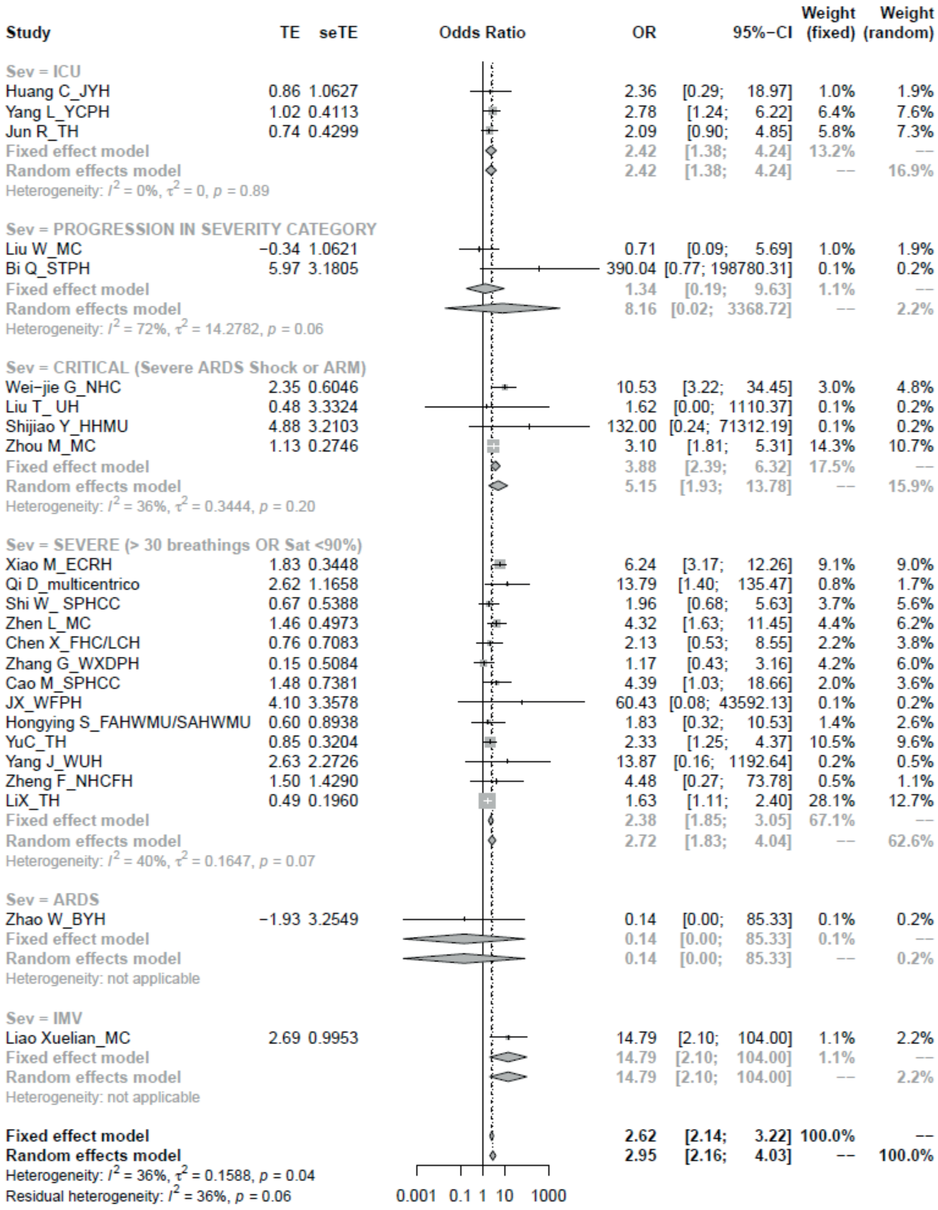

Candidate variable: High LDH (more than 240-250 U/L), outcome: severe COVID-19 disease, subgroup analysis by COVID-19 severity definition

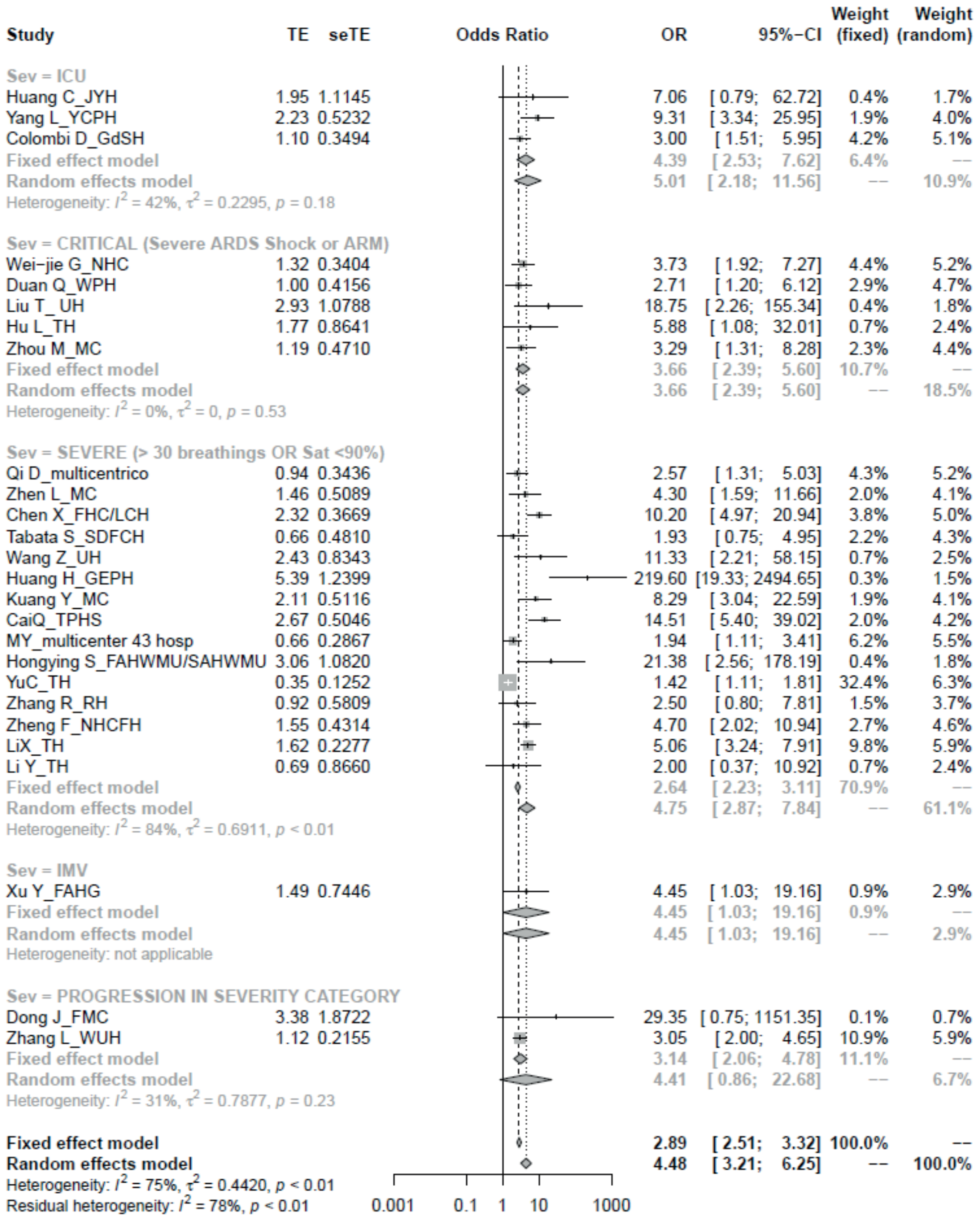

Candidate variable: LDH increase (per 1 U/L), outcome: severe COVID-19 disease, subgroup analysis by COVID-19 severity definition

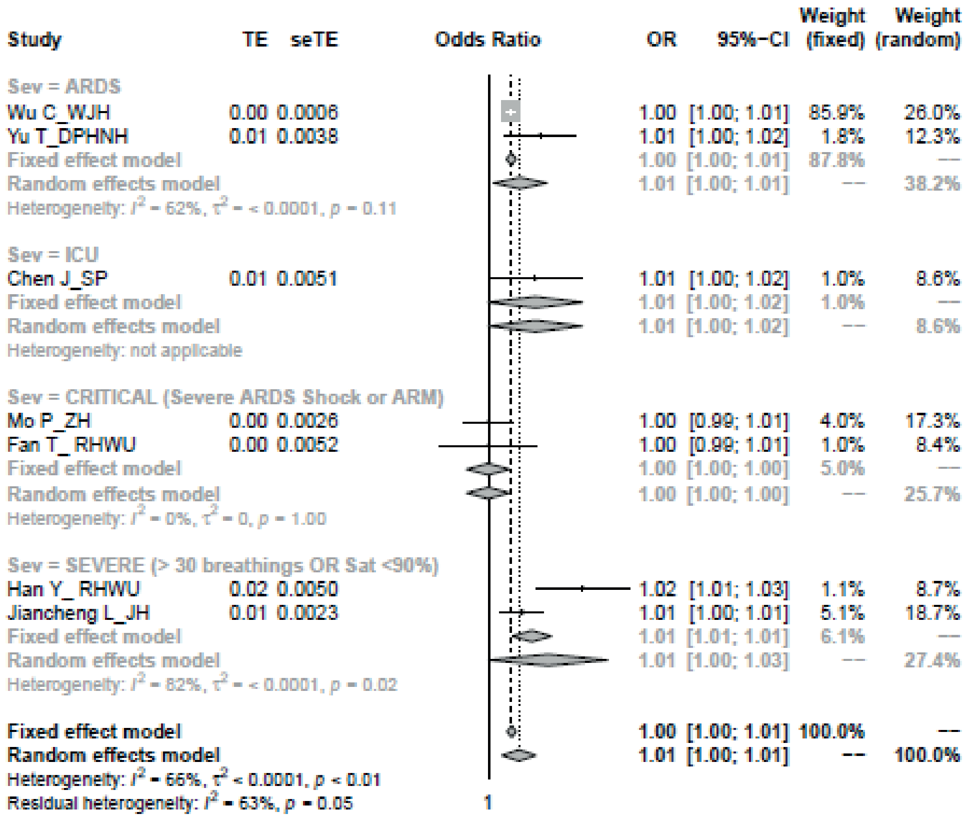

Candidate variable: CK-MB increase (per 1 U/L), outcome: severe COVID-19 disease, subgroup analysis by COVID-19 severity definition

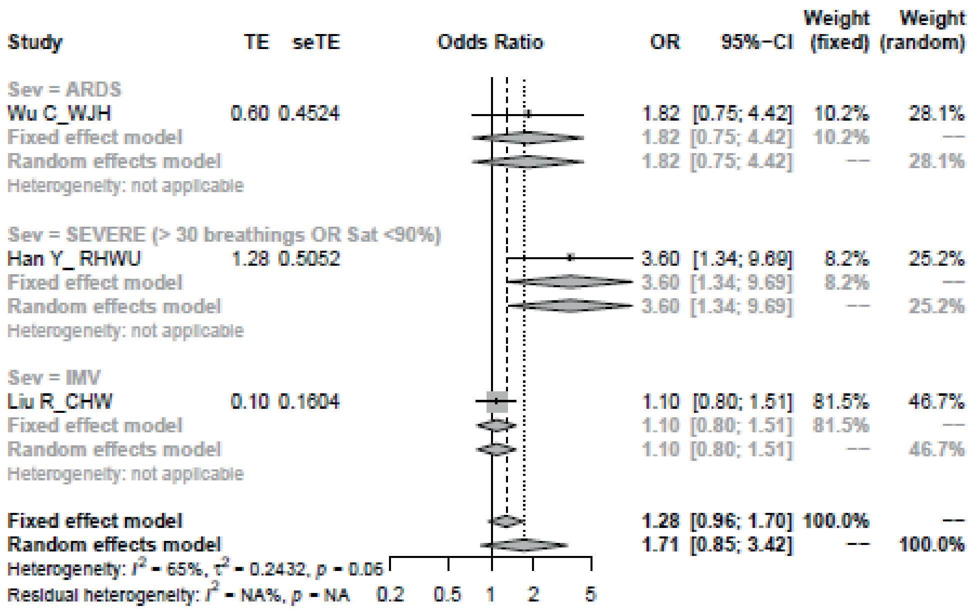

Candidate variable: High D-dimer (more than 500-1000 ng/ml), outcome: severe COVID-19 disease, subgroup analysis by COVID-19 severity definition

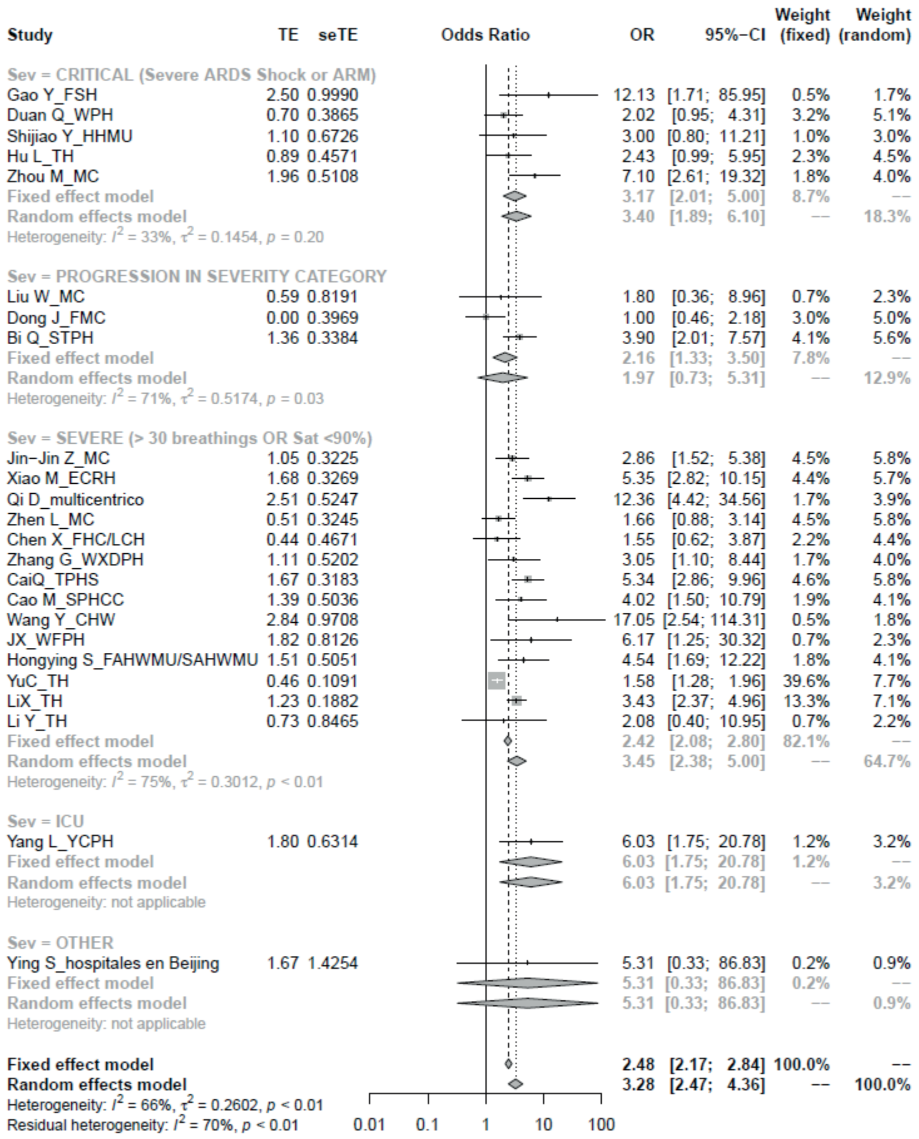

Candidate variable: Prolonged PT (more than 13.2-15 seconds), outcome: severe COVID-19 disease, subgroup analysis by COVID-19 severity definition

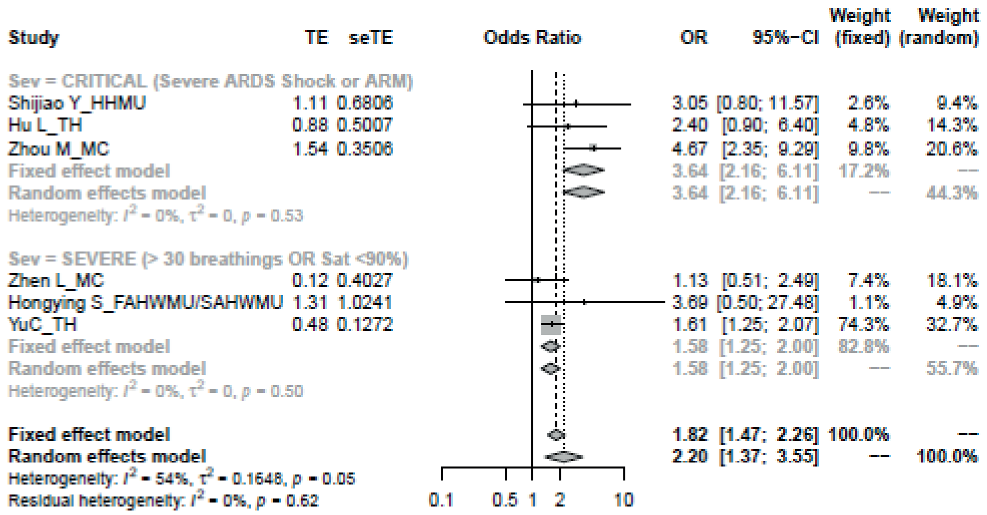

Candidate variable: Prolonged APTT (more than 35-45 seconds), outcome: severe COVID-19 disease, subgroup analysis by COVID-19 severity definition

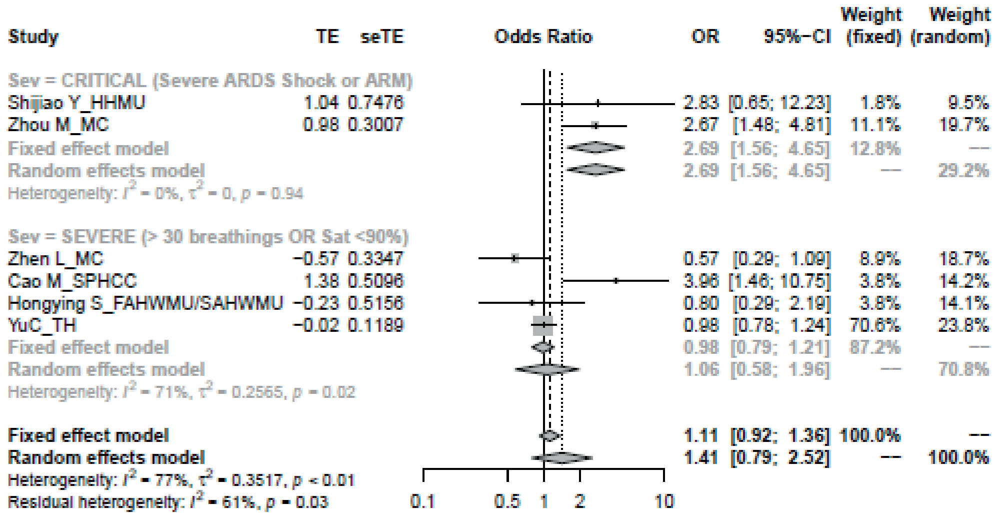

Candidate variable: APTT time increase (per 1 second), outcome: severe COVID-19 disease

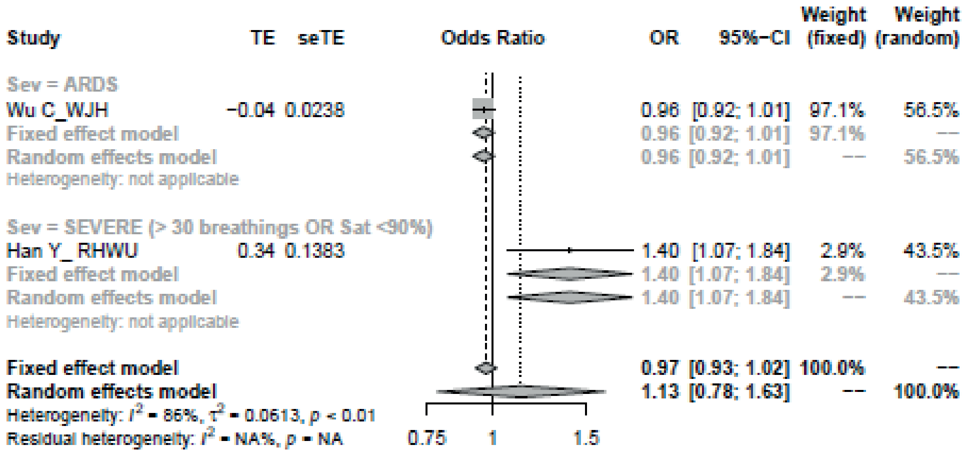

Candidate variable: High procalcitonin (More than 0.01-05 ng/ml), outcome: severe COVID-19 disease, subgroup analysis by COVID-19 severity definition

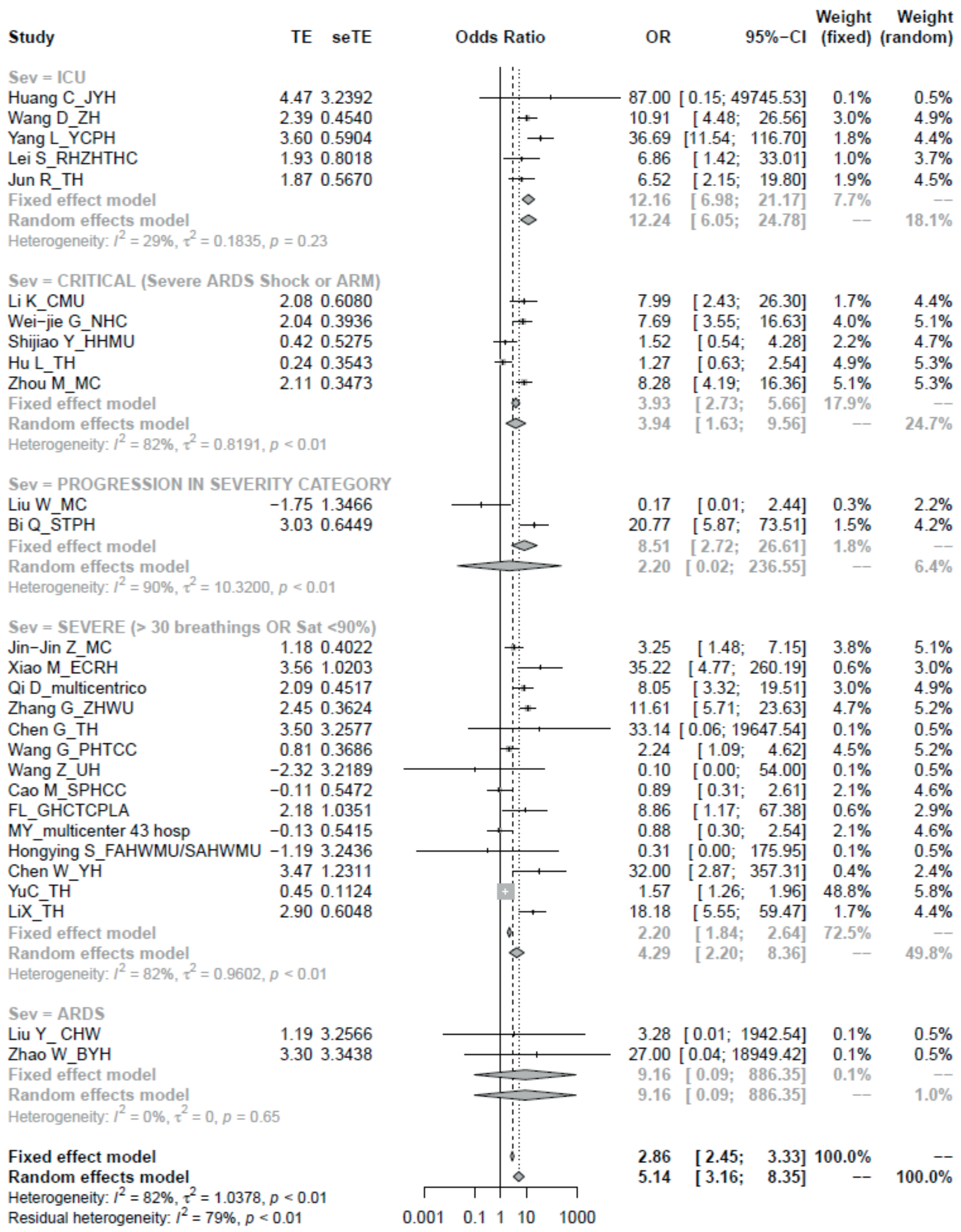

Candidate variable: High ALT level (more than 35-50 U/L), outcome: severe COVID-19 disease. subgroup analysis by COVID-19 severity definition

Study	TE	seTE	OR	95%−CI	Weight (fixed)	Weight (random)
**Sev = ARDS**						
Wu C_WJH	1.05	0.3381	2.87	[1.48; 5.57]	3.5%	4.4%
Fixed effect model			2.87	[1.48; 5.57]	3.5%	--
Random effects model			2.87	[1.48; 5.57]	--	4.4%
Heterogeneity: not applicable						
**Sev = PROGRESSION IN SEVERITY CATEGORY**						
Liu W_MC	0.03	0.5688	1.03	[0.34; 3.15]	1.2%	3.0%
Zhang L_WUH	0.40	0.1911	1.50	[1.03; 2.18]	10.9%	5.4%
Bi Q_STPH	1.61	0.2589	5.01	[3.02; 8.33]	5.9%	5.0%
Fixed effect model			2.17	[1.62; 2.90]	18.1%	--
Random effects model			2.10	[0.81; 5.46]	--	13.4%
Heterogeneity: $I^2 = 87\%$, $\tau^2 = 0.5874$, $p < 0.01$						
**Sev = CRITICAL (Severe ARDS Shock or ARM)**						
Wei−jie G_NHC	1.02	0.3058	2.77	[1.52; 5.04]	4.3%	4.7%
Liu T_ UH	1.80	1.0778	6.05	[0.73; 50.00]	0.3%	1.3%
Shijiao Y_HHMU	0.81	0.7072	2.25	[0.56; 9.01]	0.8%	2.4%
Hu L_TH	0.73	0.3303	2.08	[1.09; 3.98]	3.6%	4.5%
FY_JH, SPHCC, TPH	1.75	0.2802	5.75	[3.32; 9.95]	5.1%	4.8%
Zhou M_MC	0.69	0.2744	2.00	[1.17; 3.42]	5.3%	4.9%
Fixed effect model			2.92	[2.21; 3.87]	19.4%	--
Random effects model			2.91	[1.91; 4.42]	--	22.6%
Heterogeneity: $I^2 = 47\%$, $\tau^2 = 0.1171$, $p = 0.09$						
**Sev = SEVERE (> 30 breathings OR Sat <90%)**						
Qi D_multicentrico	1.64	0.4794	5.17	[2.02; 13.24]	1.7%	3.5%
Zhen L_MC	0.41	0.3537	1.50	[0.75; 3.01]	3.2%	4.3%
Chen X_FHC/LCH	1.02	0.4237	2.76	[1.20; 6.34]	2.2%	3.9%
Tabata S_SDFCH	1.58	0.5890	4.84	[1.52; 15.34]	1.1%	2.9%
Wang Z_UH	0.52	0.6138	1.68	[0.50; 5.58]	1.1%	2.8%
Zhang G_WXDPH	1.32	0.4806	3.75	[1.46; 9.62]	1.7%	3.5%
CaiQ_TPHS	1.81	0.3653	6.12	[2.99; 12.53]	3.0%	4.3%
Cao M_SPHCC	0.50	0.6765	1.65	[0.44; 6.20]	0.9%	2.5%
JX_WFPH	0.67	0.8075	1.96	[0.40; 9.56]	0.6%	2.0%
MY_multicenter 43 hosp	0.02	0.3118	1.02	[0.55; 1.88]	4.1%	4.6%
Hongying S_FAHWMU/SAHWMU	2.30	1.0372	10.00	[1.31; 76.36]	0.4%	1.4%
YuC_TH	−0.01	0.1303	0.99	[0.77; 1.28]	23.4%	5.7%
Zheng F_NHCFH	1.12	0.6108	3.08	[0.93; 10.18]	1.1%	2.8%
LiX_TH	0.11	0.2041	1.11	[0.75; 1.66]	9.6%	5.3%
Fixed effect model			1.45	[1.23; 1.72]	54.0%	--
Random effects model			2.23	[1.50; 3.33]	--	49.5%
Heterogeneity: $I^2 = 74\%$, $\tau^2 = 0.3534$, $p < 0.01$						
**Sev = IMV**						
Xu Y_FAHG	1.79	0.6770	6.00	[1.59; 22.62]	0.9%	2.5%
Fixed effect model			6.00	[1.59; 22.62]	0.9%	--
Random effects model			6.00	[1.59; 22.62]	--	2.5%
Heterogeneity: not applicable						
**Sev = ICU**						
Yang L_YCPH	1.12	0.4250	3.07	[1.33; 7.05]	2.2%	3.9%
Jun R_TH	0.46	0.4438	1.58	[0.66; 3.77]	2.0%	3.7%
Fixed effect model			2.23	[1.22; 4.08]	4.2%	--
Random effects model			2.23	[1.17; 4.26]	--	7.6%
Heterogeneity: $I^2 = 14\%$, $\tau^2 = 0.0303$, $p = 0.28$						
**Fixed effect model**			**1.89**	**[1.67; 2.14]**	**100.0%**	**--**
**Random effects model**			**2.44**	**[1.85; 3.21]**	**--**	**100.0%**
Heterogeneity: $I^2 = 74\%$, $\tau^2 = 0.3266$, $p < 0.01$						
Residual heterogeneity: $I^2 = 72\%$, $p < 0.01$						

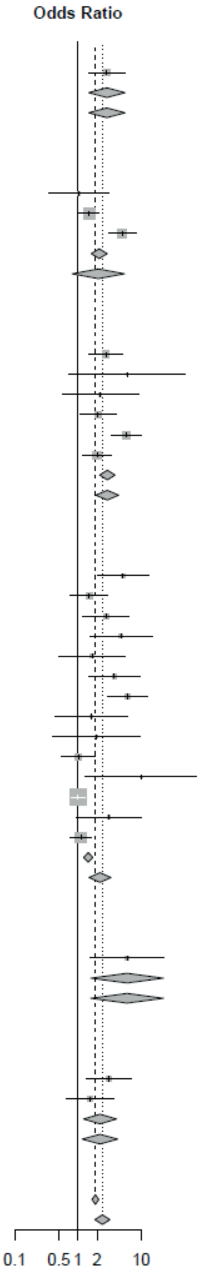

Candidate variable: ALT increase (per 1 U/L). outcome: severe COVID-19 disease. subgroup analysis by COVID-19 severity definition

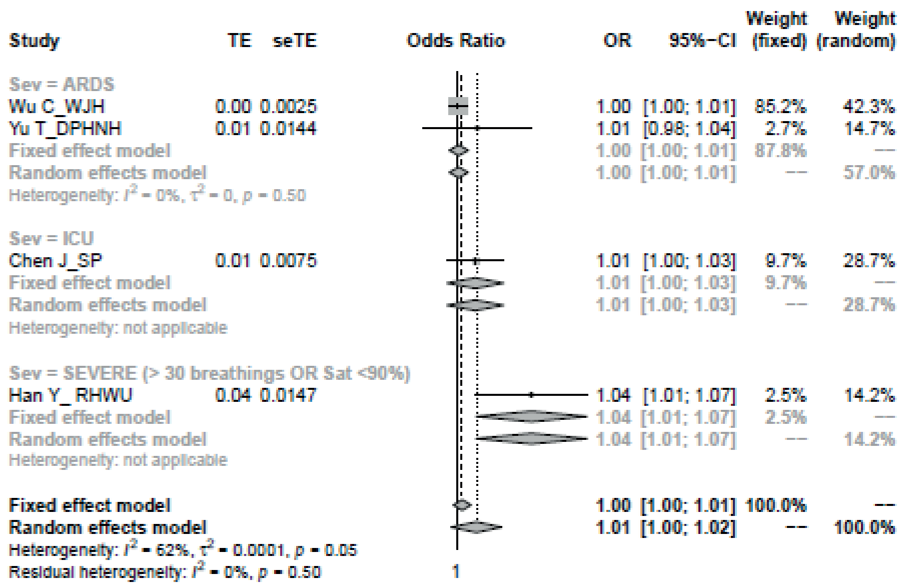

Candidate variable: High lactate (more than 1.5-2.2 mmol/L), outcome: severe COVID-19 disease, subgroup analysis by COVID-19 severity definition

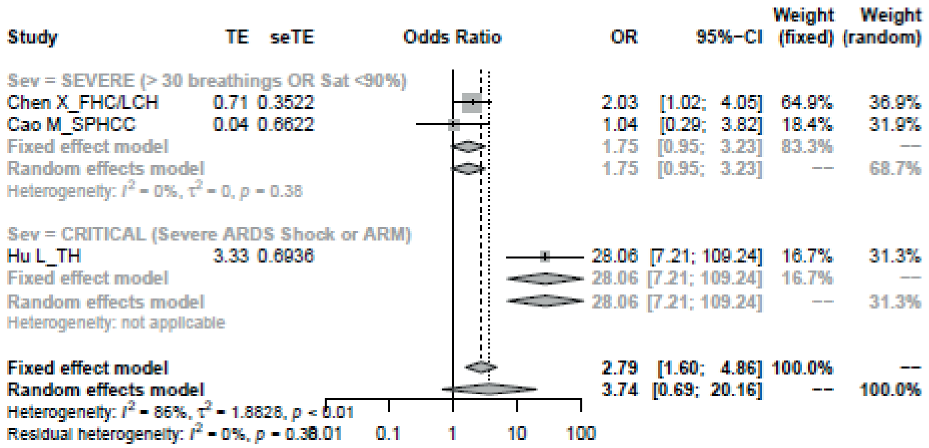

Candidate variable: Ground glass opacity. outcome: severe COVID-19 disease, subgroup analysis by COVID-19 severity definition

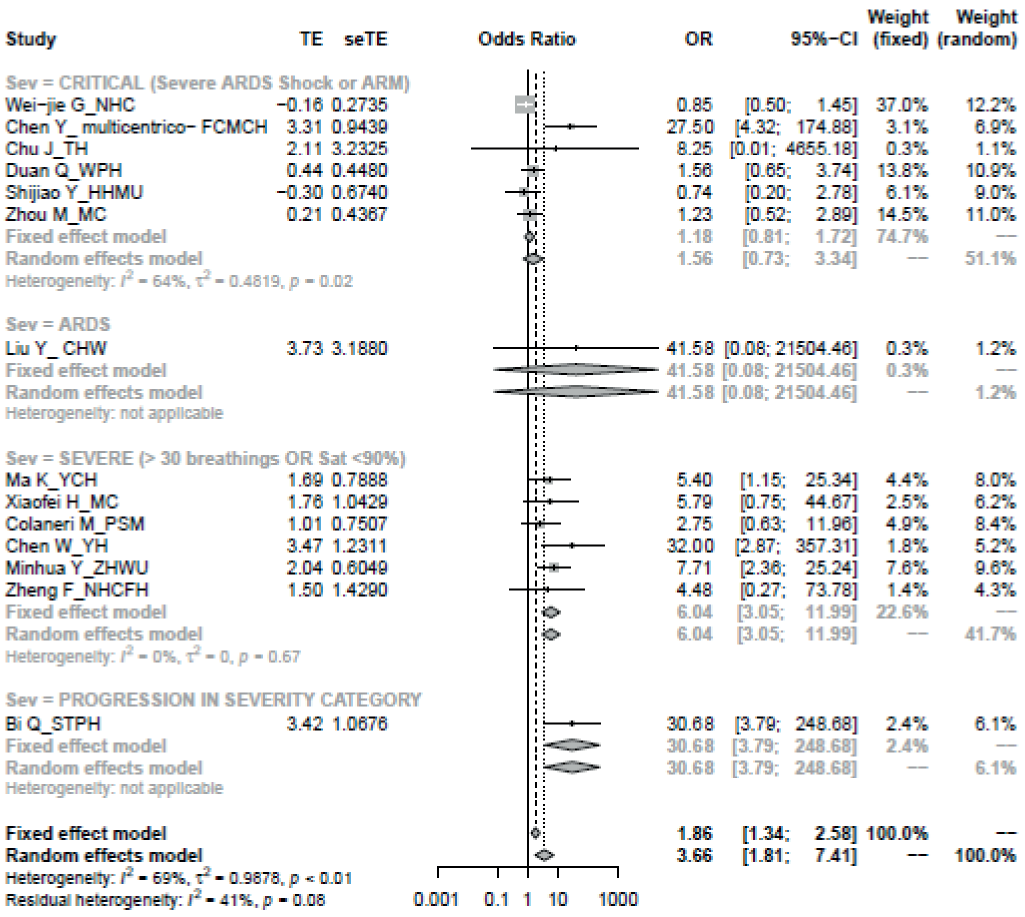

Candidate variable: Crazy paving pattern (CT assessment), outcome: severe COVID-19 disease, subgroup analysis by COVID-19 severity definition

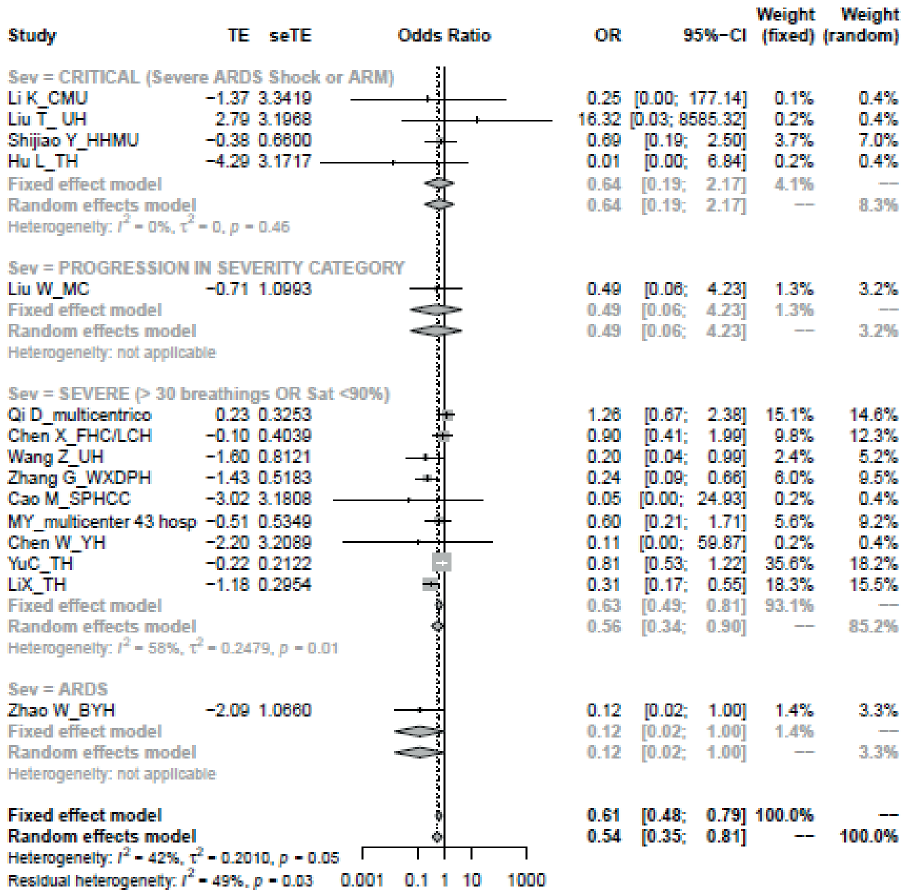

Candidate variable: Consolidation pattern, outcome: severe COVID-19 disease, subgroup analysis by COVID-19 severity definition

Study	TE	seTE	OR	95%−CI	Weight (fixed)	Weight (random)
Sev = CRITICAL (Severe ARDS Shock or ARM)						
Li K_CMU	1.85	0.6693	6.38	[1.72; 23.69]	1.2%	3.9%
Liu K_MC	5.49	3.1952	241.94	[0.46; 126892.91]	0.1%	0.5%
Chen Y_ multicentrico− FCMCH	0.36	0.6981	1.43	[0.36; 5.61]	1.1%	3.8%
Chu J_TH	−0.77	0.9417	0.46	[0.07; 2.92]	0.6%	3.0%
Zhang L_TH	1.94	0.9488	6.96	[1.08; 44.70]	0.6%	3.0%
FY_JH, SPHCC, TPH	−0.98	0.4446	0.38	[0.16; 0.90]	2.8%	4.6%
Zhou M_MC	1.88	0.3354	6.58	[3.41; 12.70]	4.9%	5.0%
Fixed effect model			2.46	[1.59; 3.79]	11.4%	--
Random effects model			2.40	[0.70; 8.21]	--	23.7%
Heterogeneity: I^2 = 83%, τ^2 = 1.9779, p < 0.01						
Sev = SEVERE (> 30 breathings OR Sat <90%)						
Jin−Jin Z_MC	0.83	0.2969	2.29	[1.28; 4.10]	6.3%	5.1%
Lu Jiatao_WHH	−1.74	3.2466	0.18	[0.00; 102.11]	0.1%	0.5%
Qi D_multicentrico	2.46	0.6246	11.69	[3.44; 39.76]	1.4%	4.0%
Lei L_CUTGH	1.90	0.9025	6.67	[1.14; 39.10]	0.7%	3.1%
Feng Z_TXH	0.14	0.7993	1.15	[0.24; 5.53]	0.9%	3.4%
Ma K_YCH	2.25	0.6774	9.44	[2.50; 35.62]	1.2%	3.8%
Tabata S_SDFCH	1.18	0.5874	3.24	[1.02; 10.25]	1.6%	4.1%
Kuang Y_MC	1.28	0.4262	3.58	[1.55; 8.26]	3.0%	4.7%
CM_FAHSYU	2.12	0.5234	8.32	[2.98; 23.20]	2.0%	4.4%
Hongying S_FAHWMU/SAHWMU	0.31	3.2973	1.37	[0.00; 876.74]	0.1%	0.5%
Xin L_CHWC/hospitales en Hunan	1.31	0.5315	3.69	[1.30; 10.46]	2.0%	4.3%
Chen W_YH	1.23	0.9271	3.43	[0.56; 21.10]	0.6%	3.0%
Minhua Y_ZHWU	1.06	0.7643	2.88	[0.64; 12.86]	0.9%	3.5%
YuC_TH	−0.42	0.1024	0.65	[0.54; 0.80]	52.7%	5.5%
Zhang R_RH	1.97	0.5347	7.16	[2.51; 20.43]	1.9%	4.3%
Zheng F_NHCFH	−0.39	0.4257	0.68	[0.30; 1.57]	3.0%	4.7%
Fixed effect model			1.10	[0.93; 1.30]	78.4%	--
Random effects model			3.07	[1.60; 5.89]	--	59.0%
Heterogeneity: I^2 = 86%, τ^2 = 1.2280, p < 0.01						
Sev = PROGRESSION IN SEVERITY CATEGORY						
Zhao W_SXH	0.09	0.3809	1.09	[0.52; 2.30]	3.8%	4.8%
Wang X_DFH	0.78	0.3674	2.19	[1.07; 4.50]	4.1%	4.9%
Fixed effect model			1.57	[0.93; 2.63]	7.9%	--
Random effects model			1.56	[0.78; 3.09]	--	9.7%
Heterogeneity: I^2 = 43%, τ^2 = 0.1042, p = 0.19						
Sev = OTHER						
Ying S_hospitales en Beijing	0.47	0.6513	1.61	[0.45; 5.76]	1.3%	3.9%
Fixed effect model			1.61	[0.45; 5.76]	1.3%	--
Random effects model			1.61	[0.45; 5.76]	--	3.9%
Heterogeneity: not applicable						
Sev = IMV						
Herold T_UH	−0.44	0.7182	0.64	[0.16; 2.63]	1.1%	3.7%
Fixed effect model			0.64	[0.16; 2.63]	1.1%	--
Random effects model			0.64	[0.16; 2.63]	--	3.7%
Heterogeneity: not applicable						
**Fixed effect model**			**1.24**	**[1.07; 1.43]**	**100.0%**	**--**
**Random effects model**			**2.46**	**[1.54; 3.93]**	**--**	**100.0%**
Heterogeneity: I^2 = 84%, τ^2 = 1.0381, p < 0.01						
Residual heterogeneity: I^2 = 85%, p < 0.01						

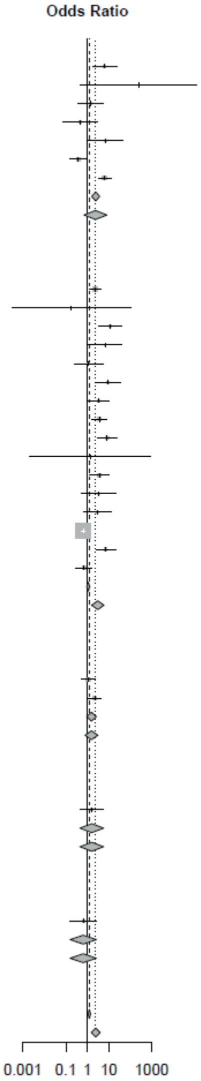

Candidate variable: Enlarged lymph nodes, outcome: severe COVID-19 disease, subgroup analysis by COVID-19 severity definition

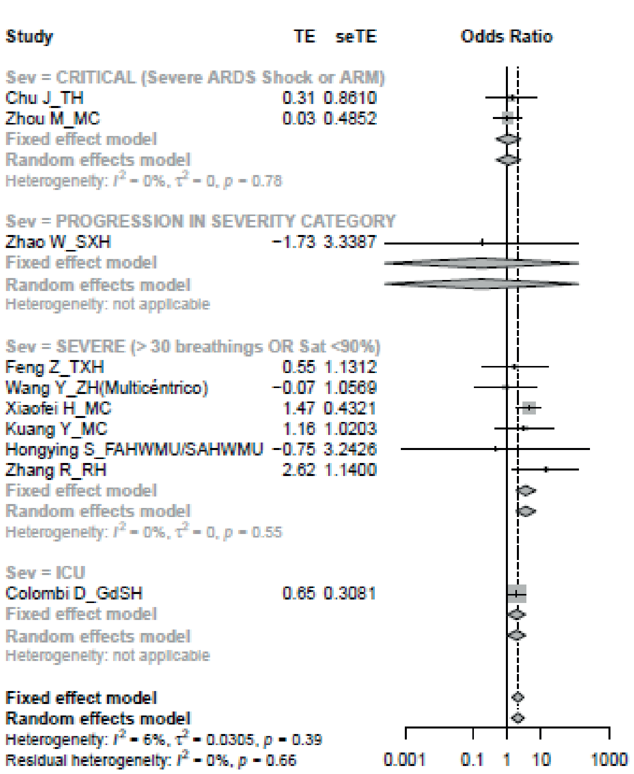

Candidate variable: Pleural effusion (X ray or CT assessment). outcome: severe COVID-19 disease, subgroup analysis by COVID-19 severity definition

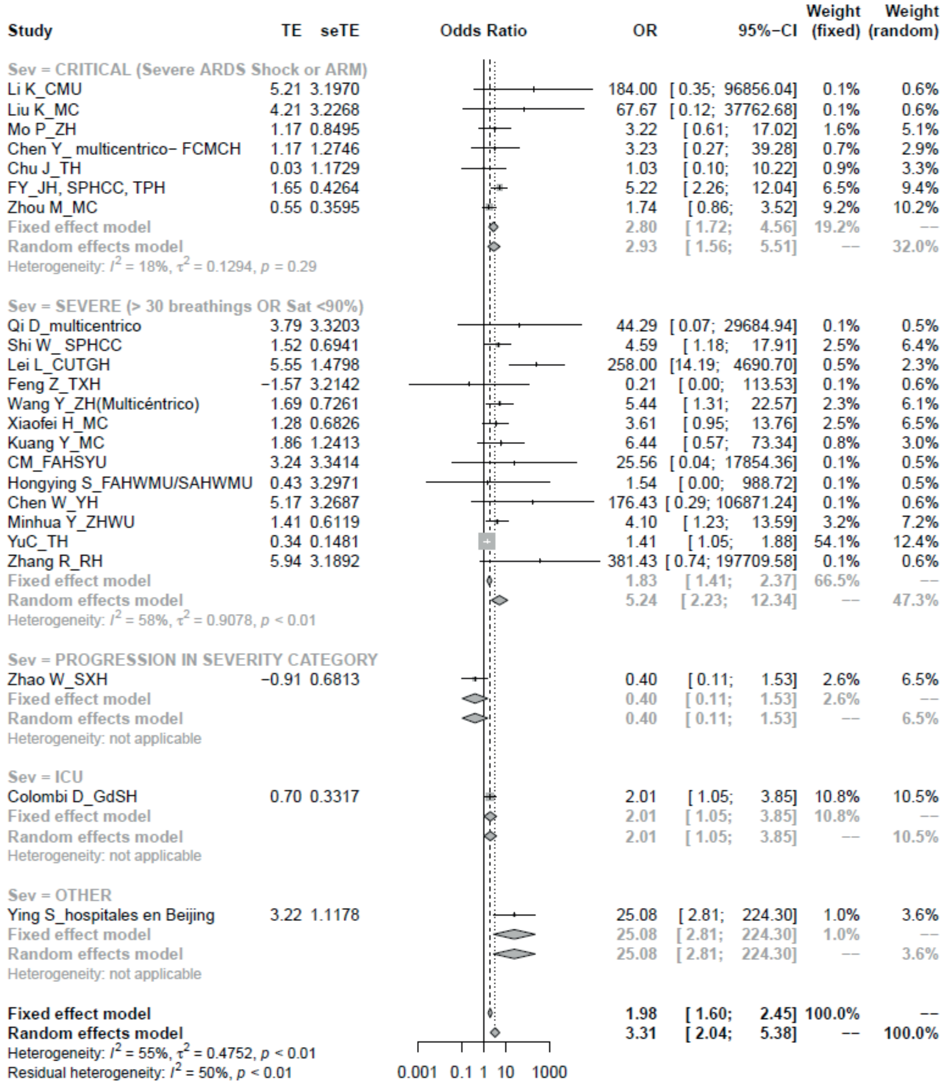

Candidate variable: Bilateral infiltrates. outcome: severe COVID-19 disease, subgroup analysis by COVID-19 severity definition

Study	TE	seTE	OR	95%-CI	Weight (fixed)	Weight (random)
Sev = ICU						
Huang C_JYH	1.47	3.3503	4.33	[0.01; 3080.50]	0.1%	0.5%
Yang L_YCPH	1.66	1.0391	5.25	[0.69; 40.24]	0.6%	2.7%
Colombi D_GdSH	−0.68	0.5858	0.51	[0.16; 1.60]	1.9%	3.9%
Fixed effect model			0.92	[0.34; 2.49]	2.6%	--
Random effects model			1.44	[0.22; 9.38]	--	7.0%
Heterogeneity: $I^2 = 51\%$, $\tau^2 = 1.3245$, $p = 0.13$						
Sev = CRITICAL (Severe ARDS Shock or ARM)						
Li K_CMU	2.89	3.2116	18.06	[0.03; 9780.32]	0.1%	0.5%
Liu K_MC	4.93	3.1827	138.58	[0.27; 70925.14]	0.1%	0.5%
Wei-jie G_NHC	1.61	0.3745	4.99	[2.40; 10.40]	4.7%	4.4%
Chen Y_ multicentrico- FCMCH	2.66	3.2623	14.25	[0.02; 8524.88]	0.1%	0.5%
Duan Q_WPH	−0.01	0.5516	0.99	[0.34; 2.92]	2.2%	4.0%
Shijiao Y_HHMU	1.03	0.5418	2.79	[0.96; 8.07]	2.2%	4.0%
Hu L_TH	−1.14	0.4572	0.32	[0.13; 0.78]	3.2%	4.2%
Li J_CHW	2.50	1.0537	12.16	[1.54; 95.91]	0.6%	2.6%
Liu J_BDH	0.92	0.8294	2.50	[0.49; 12.70]	1.0%	3.2%
Zhou M_MC	1.13	0.4820	3.09	[1.20; 7.95]	2.8%	4.1%
Wu J_TFAH	−0.35	0.2710	0.70	[0.41; 1.20]	9.0%	4.6%
Fixed effect model			1.44	[1.05; 1.96]	25.8%	--
Random effects model			2.03	[0.94; 4.42]	--	32.8%
Heterogeneity: $I^2 = 76\%$, $\tau^2 = 0.9859$, $p < 0.01$						
Sev = PROGRESSION IN SEVERITY CATEGORY						
Liu W_MC	0.29	0.6735	1.34	[0.36; 5.00]	1.5%	3.6%
Zhao W_SXH	5.39	3.1755	219.49	[0.43; 110775.86]	0.1%	0.5%
Bi Q_STPH	2.50	0.5240	12.23	[4.38; 34.16]	2.4%	4.0%
Fixed effect model			5.65	[2.53; 12.61]	3.9%	--
Random effects model			6.05	[0.76; 48.07]	--	8.2%
Heterogeneity: $I^2 = 75\%$, $\tau^2 = 2.1021$, $p = 0.02$						
Sev = SEVERE (> 30 breathings OR Sat <90%)						
Jin−Jin Z_MC	0.67	0.6193	1.95	[0.58; 6.56]	1.7%	3.8%
Lu Jiatao_WHH	−0.63	0.2298	0.53	[0.34; 0.83]	12.5%	4.7%
Shi W_ SPHCC	1.67	0.7516	5.33	[1.22; 23.25]	1.2%	3.4%
Zhang G_ZHWU	3.01	3.1927	20.28	[0.04; 10586.55]	0.1%	0.5%
Chen G_TH	1.46	1.2554	4.29	[0.37; 50.19]	0.4%	2.2%
Feng Z_TXH	−0.06	0.8053	0.95	[0.20; 4.58]	1.0%	3.2%
Ma K_YCH	4.78	3.1806	119.50	[0.23; 60916.28]	0.1%	0.5%
Tabata S_SDFCH	1.18	0.4587	3.27	[1.33; 8.03]	3.1%	4.2%
JX_WFPH	3.81	3.1964	45.07	[0.09; 23695.01]	0.1%	0.5%
Hongying S_FAHWMU/SAHWMU	1.94	3.2258	6.95	[0.01; 3868.08]	0.1%	0.5%
Xin L_CHWC/hospitales en Hunan	2.29	1.0530	9.85	[1.25; 77.58]	0.6%	2.6%
Chen W_YH	4.40	3.1894	81.79	[0.16; 42420.54]	0.1%	0.5%
YuC_TH	−0.70	0.1306	0.50	[0.38; 0.64]	38.6%	4.8%
Wang L_SPH	3.36	3.1882	28.68	[0.06; 14838.31]	0.1%	0.5%
Zhang R_RH	2.86	0.7620	17.50	[3.93; 77.93]	1.1%	3.4%
Zheng F_NHCFH	1.95	0.5648	7.02	[2.32; 21.23]	2.1%	3.9%
LiX_TH	1.02	0.4556	2.77	[1.13; 6.76]	3.2%	4.2%
Fixed effect model			0.80	[0.66; 0.98]	65.9%	--
Random effects model			3.02	[1.47; 6.23]	--	43.7%
Heterogeneity: $I^2 = 82\%$, $\tau^2 = 1.1967$, $p < 0.01$						
Sev = ARDS						
Zhao W_BYH	3.38	1.0422	29.47	[3.82; 227.24]	0.6%	2.6%
Dreher M_UHA	3.29	1.0946	26.83	[3.14; 229.31]	0.5%	2.5%
Fixed effect model			28.19	[6.42; 123.74]	1.2%	--
Random effects model			28.19	[6.42; 123.74]	--	5.2%
Heterogeneity: $I^2 = 0\%$, $\tau^2 = 0$, $p = 0.95$						
Sev = OTHER						
Ying S_hospitales en Beijing	7.30	3.2059	1478.20	[2.76; 791812.33]	0.1%	0.5%
Fixed effect model			1478.20	[2.76; 791812.33]	0.1%	--
Random effects model			1478.20	[2.76; 791812.33]	--	0.5%
Heterogeneity: not applicable						
Sev = IMV						
Herold T_UH	−0.74	1.0713	0.48	[0.06; 3.89]	0.6%	2.6%
Fixed effect model			0.48	[0.06; 3.89]	0.6%	--
Random effects model			0.48	[0.06; 3.89]	--	2.6%
Heterogeneity: not applicable						
**Fixed effect model**			**1.06**	**[0.90; 1.24]**	**100.0%**	**--**
**Random effects model**			**2.99**	**[1.83; 4.87]**	**--**	**100.0%**
Heterogeneity: $I^2 = 81\%$, $\tau^2 = 1.2735$, $p < 0.01$						
Residual heterogeneity: $I^2 = 78\%$, $p < 0.01$						

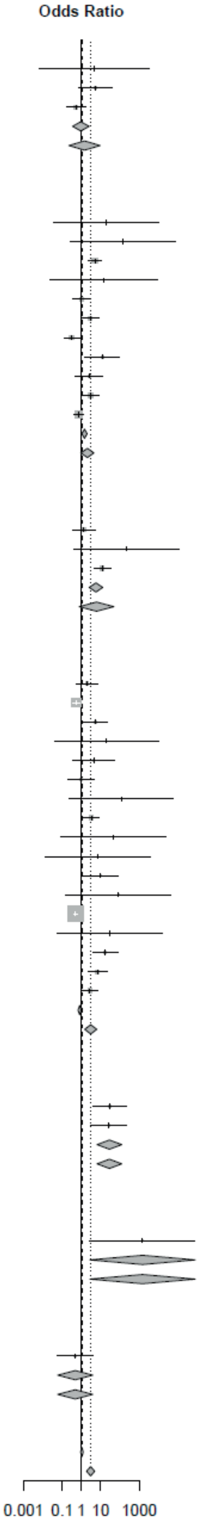

Candidate variable: High APACHE score (more than 8), outcome: severe COVID- 19 disease. subgroup analysis by COVID-19 severity definition

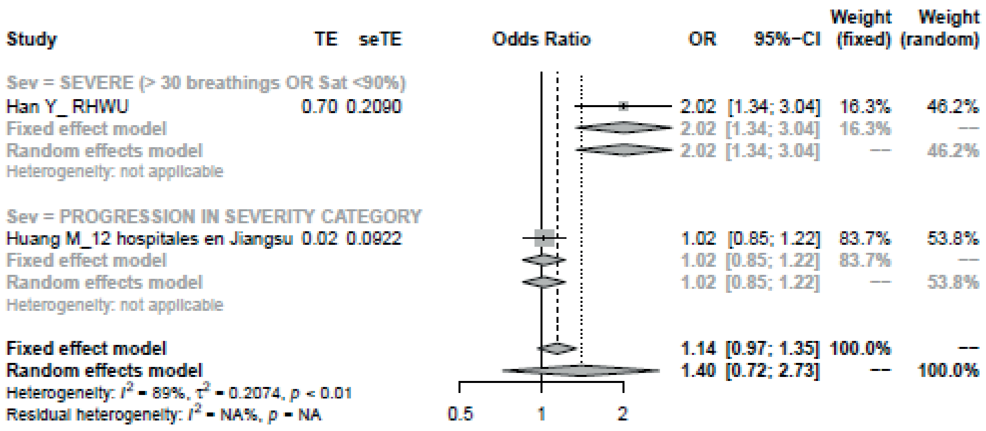

Supplement: S3 Appendix — This file contains the Forest plots for all performed subgroup analyses. (PDF) [file pone.0241955.s008.pdf]
