# Supplementary material for: Correction: Prognostic factors for severity and mortality in patients infected with COVID-19: A systematic review
Source: PLoS One. 2022 May 26;17(5):e0269291. doi: 10.1371/journal.pone.0269291 (PMC9135219; doi:10.1371/journal.pone.0269291)
Supplement: S1 Appendix — The Forest plots for all assessed candidate variables, including the updated Forest plots for age, gender, smoking, COPD, cardiovascular disease, immunocompromise, diabetes, arterial hypertension, dyslipidemia and cardiac arrhythmia. An additional table is included to identify the original reference for the study labels used in the Forest plots. (DOCX) [file pone.0269291.s001.docx]

Forest plot comparison: Covid 19 mortality risk

| Glossary |
| --- |
| ADJ=0: crude estimate  ADJ=1: adjust estimate  LRB=0: moderate and high subgroup analysis by risk of bias  LRB=1: low subgroup analysis by risk of bias  MRB=0: high subgroup analysis by risk of bias  MRB=1: moderate and low subgroup analysis by risk of bias  ALT: Alanine aminotransferase  APTT: activated partial thromboplastin time  APACHE: Acute Physiology And Chronic Health Evaluation II  AST: Aspartate aminotransferase  BUN: Blood urea nitrogen  PT: prothrombin time  SOFA: The sequential organ failure assessment score  FDP: Fibrin Degradation Product  PT: prothrombin time |

Forest plot study label clarification

| Forest plot label | Study - reference |
| --- | --- |
| Bai X_WPH | Bai T. et al^32^ |
| CaiQ_TPHS | Cai Q. et al^34^ |
| Chen J_FAH | Chen J. et al^39^ |
| Chen J_SP | Chen J. et al^40^ |
| Chen M_SH- WC | Chen M. et al^43^ |
| TieLong C_ZH | Chen TL. et al^45^ |
| CM_FAHSYU | Chen M. et al^42^ |
| Chen M_ GHCTC | Chen X. et al^49^ |
| Chen X_TH | Chen X. et al^47^ |
| Xudan C_GEPH | Chen X. et al^48^ |
| Chen X_FHC/LCH | Chen X. et al^50^ |
| Docherty MB_MC | Docherty AB. et al^57^ |
| FanJ_ZH | Fan J. et al^64^ |
| FY_JH, SPHCC, TPH | Feng Y. et al^65^ |
| Fu L_UH | Fu L. et al^67^ |
| Lin F_UHHUST | Fu L. et al^68^ |
| GaoL_HGH | Gao L. et al^69^ |
| Tian G_ MC | Gu T. et al^72^ |
| Guan W_575 hospitales | Guan W. et al^73^ |
| Wei-jie G_NHC | Guan W. et al^74^ |
| CB_UDH | Guo TM. et al^76^ |
| GuoW_WUH | Guo W et al^77^ |
| HanH_RH | Han H. et al^78^ |
| Mingfeng H_SPH | Han M. et al^79^ |
| Xiaofei H_MC | Hu X. et al^86^ |
| HuZ_TH | Hu Z. et al^87^ |
| JX_WFPH | Jiang X. et al^91^ |
| Kalligeron M_ | Kalligeros M. et al^94^ |
| LH_SNUCM | Lee HY. et al^96^ |
| Li J_WRCH | Li J. et al^100^ |
| Li J_CHW | Li J. et al^101^ |
| Li J_CHW | Li J. et al^102^ |
| Li Y_TH | Li Y. et al^104^ |
| Li Y_TH | Li Y. et al^105^ |
| Jiancheng L_JH | Liu J. et al^110^ |
| Jing L_WUH | Liu J. et al^111^ |
| Liu J_BDH | Liu J. et al^112^ |
| Yang L_SH | Liu Y. et al^117^ |
| Liu Yo_SCH | Liu Y. et al^118^ |
| Liu Y_ZHWU | Liu Y. et al^119^ |
| Liu Y_CHW | Liu Y. et al^120^ |
| Liu Y_ CHW | Liu Y. et al^121^ |
| Liu Y_STP | Liu Y. et al^122^ |
| Xin L_CHWC/hospitales en Hunan | Lv X. et al^126^ |
| Jia M_RHWU | Ma J. et al^127^ |
| YM_ multicenter 43 hosp | Ma Y. et al^129^ |
| NS_BEMC | Niu S. et al^132^ |
| Lei P_BH | Pan L. et al^133^ |
| ParanjpeI_MSHCS | Paranjpe L. et al^134^ |
| Qi Xiaolong_MC | Qi X. et al^137^ |
| Qin X_SPH | Qi X. et al^138^ |
| Jun R_TH | Ran J. et al^140^ |
| Jit S_MC | Sarkar J. et al^144^ |
| Hongying S_FAHWMU/SAHWMU | Shi H. et al^145^ |
| Ying S_hospitales en Beijing | Sun Y. et al^152^ |
| Wen J_ZH | Tu WJ. et al^157^ |
| Wang D_ZH | Wang D. et al^159^ |
| Wang D_WH/XH | Wang D. et al^160^ |
| Lang W_RH | Wang L. et al^162^ |
| Wang L_HPUW | Wang L. et al^163^ |
| WangL_RH | Wang L. et al^164^ |
| Wang L_SPH | Wang L. et al^165^ |
| WR_PHFC | Wang R. et al^166^ |
| Wang Y_ZH | Wang Y. et al^168^ |
| WangY_TH | Wang Y. et al^169^ |
| Wang Y_CHW | Wang Y. et al^170^ |
| Ying W_ MC | Wen Y. et al^173^ |
| Wentao X_AIDH | Xu W. et al^181^ |
| Xu Y_FAHG | Xu Y. et al^182^ |
| Xu Y_GH | Xu YH. et al^183^ |
| Shijiao Y_HHMU | Yan S. et al^184^ |
| Yang A_ | Yang AP. et al^186^ |
| Yang X_WJH | Yang X. et al^190^ |
| Yang X_WJY | Yang X. et al^191^ |
| YuC_TH | Yu C. et al^194^ |
| Minhua Y_ZHWU | Yu M. et al^195^ |
| Zang L_WAGH | Zhang L. et al^198^ |
| Zeng G_TPHS | Zeng L. et al^199^ |
| ZengZ_HH | Zeng Z. et al^200^ |
| Zhang G_ZHWU | Zhang G. et al^202^ |
| Zhang G_WXDPH | Zhang G. et al^203^ |
| Zhang H_CPHMC | Zhang H. et al^204^ |
| Zhang H_ZH | Zhang H. et al^205^ |
| Jin-Jin Z_MC | Zhang JJ. et al^207^ |
| Zhang L_WUH | Zhang L. et al^208^ |
| Zhang L_WUH | Zhang L. et al^209^ |
| Zhang L_CHW | Zhang L. et al^210^ |
| Zhang L_TH | Zhang L. et al^211^ |
| Zhang_MC | Zhang P. et al^212^ |
| Shuai Z_UHTMC | Zhang S. et al^214^ |
| Zhang S_JH | Zhang S. et al^215^ |
| Zhang X_SEH | Zhang X. et al^216^ |
| ZhangX_MC | Zhang X. et al^217^ |
| Zhao W_SXH | Zhao W. et al^219^ |
| Zhao W_BYH | Zhao W. et al^220^ |
| Hu D_UH | Zhou H. et al^226^ |
| Zhou H_UH | Zhou H. et al^227^ |

| Candidate variable: Age (older than 50 - 65 years), outcome: mortality, subgroup analysis by risk of bias (moderate/high vs low) |
| --- |
| 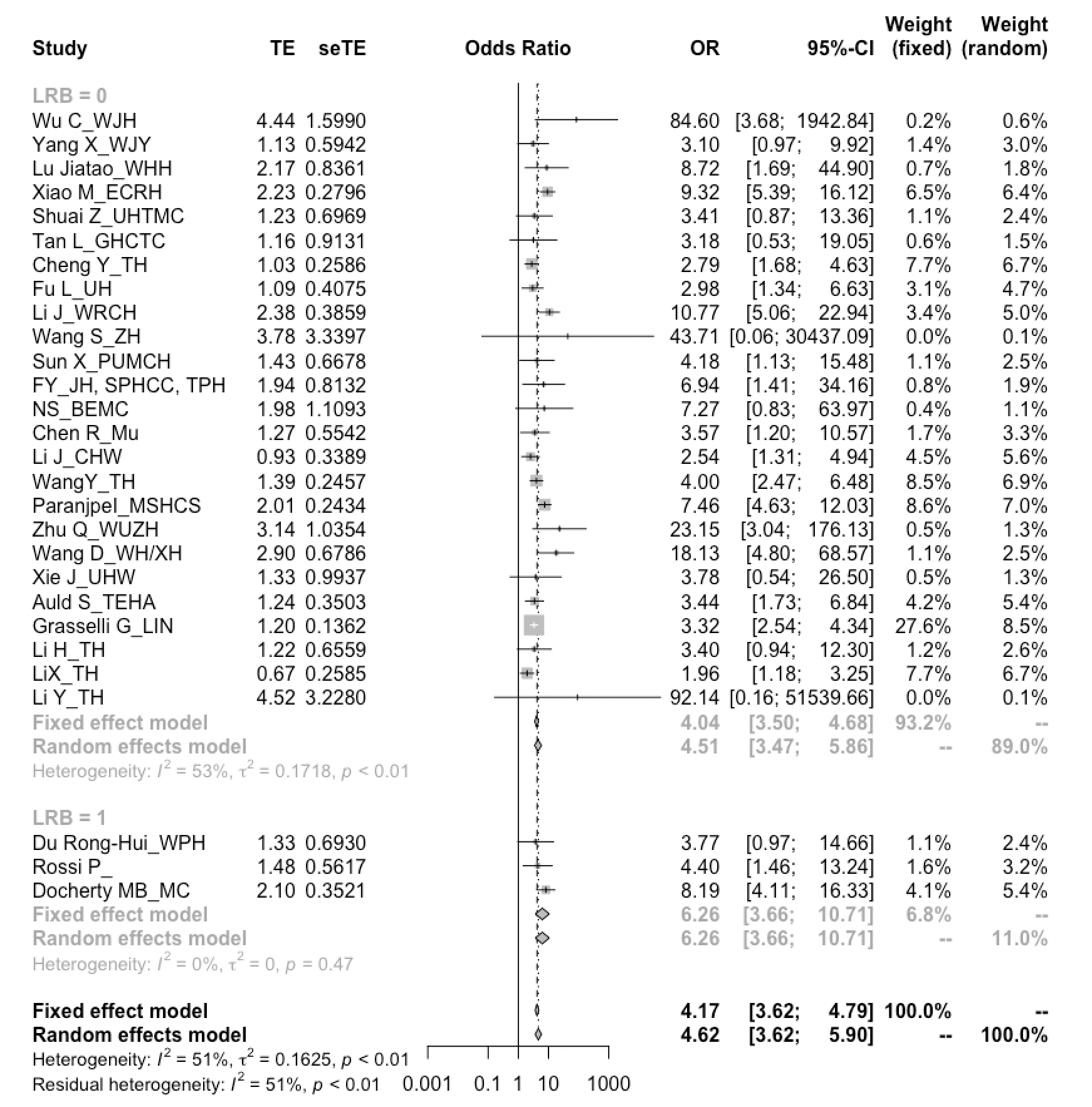 |

| Candidate variable: Age increase (per 1 year), outcome: mortality, subgroup analysis by risk of bias: (high vs moderate/low) |
| --- |
| 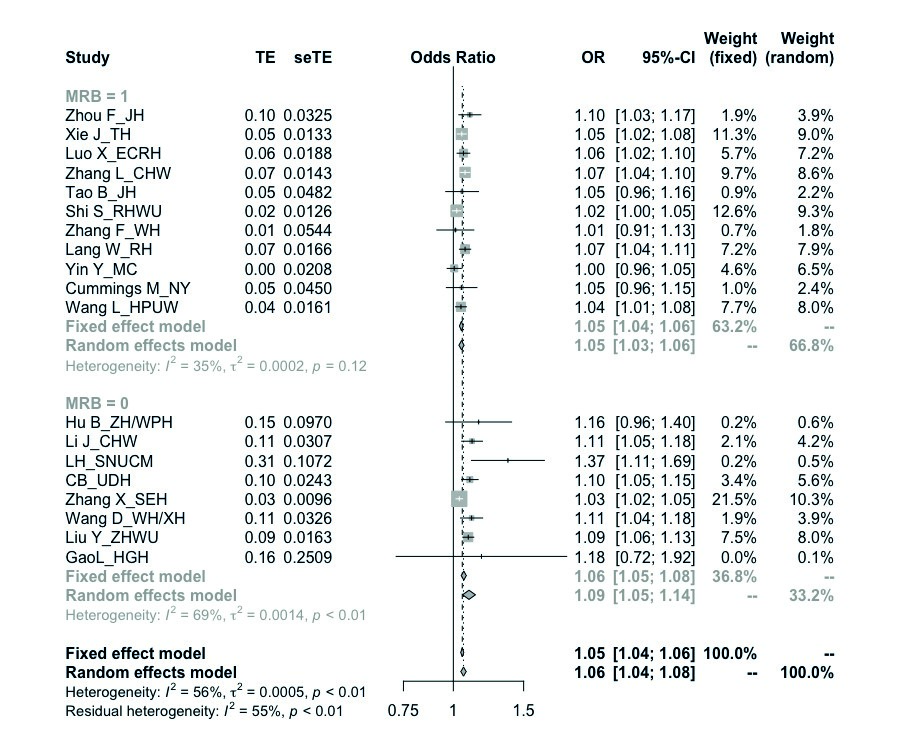 |

| Candidate variable: Male gender, outcome: mortality, subgroup analysis by risk of bias: (high vs moderate/low) |
| --- |
| 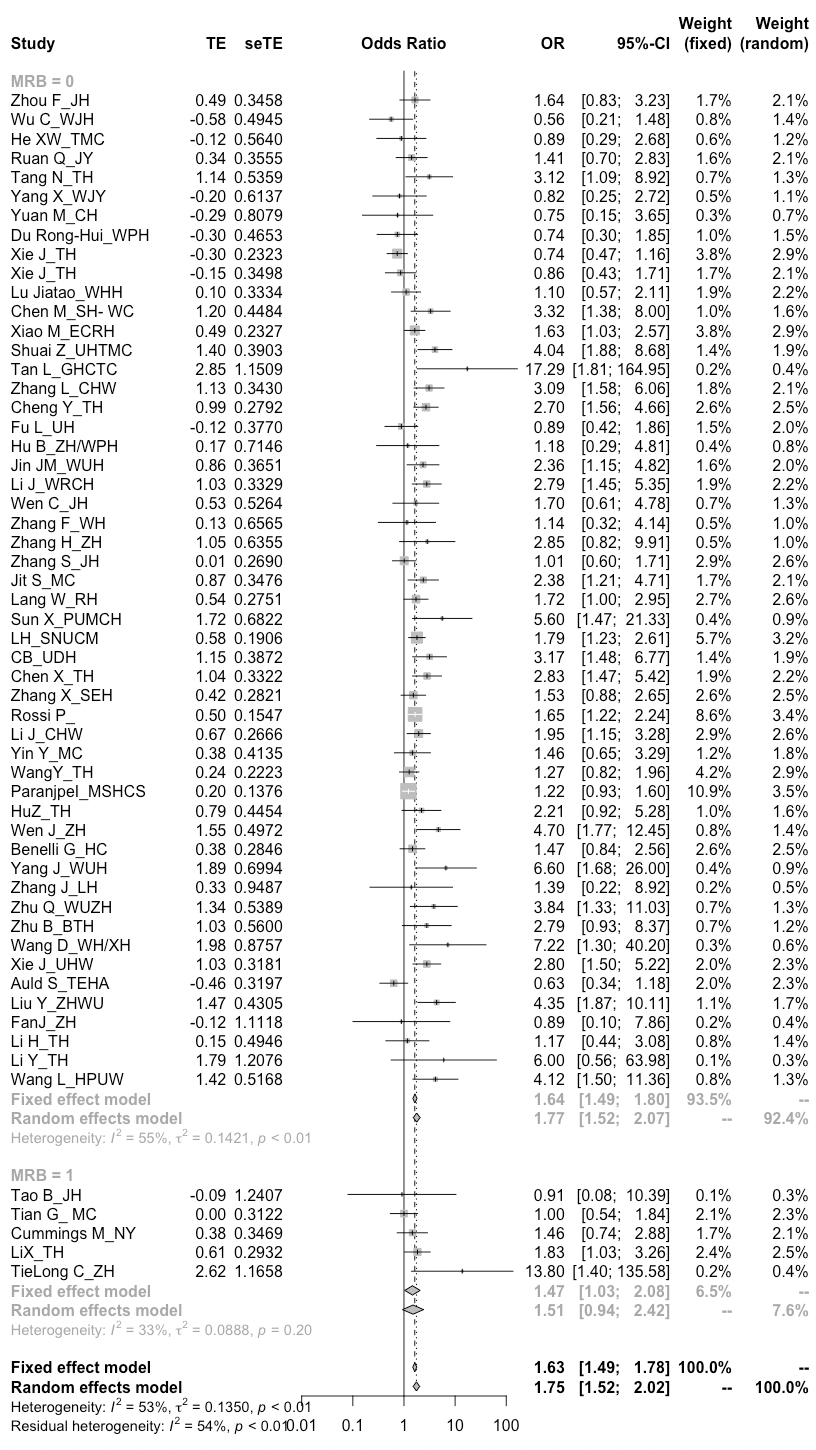 |

| Candidate variable: Smoking (Active, present smoker), outcome: mortality, subgroup analysis: (crude vs adjusted) |
| --- |
| 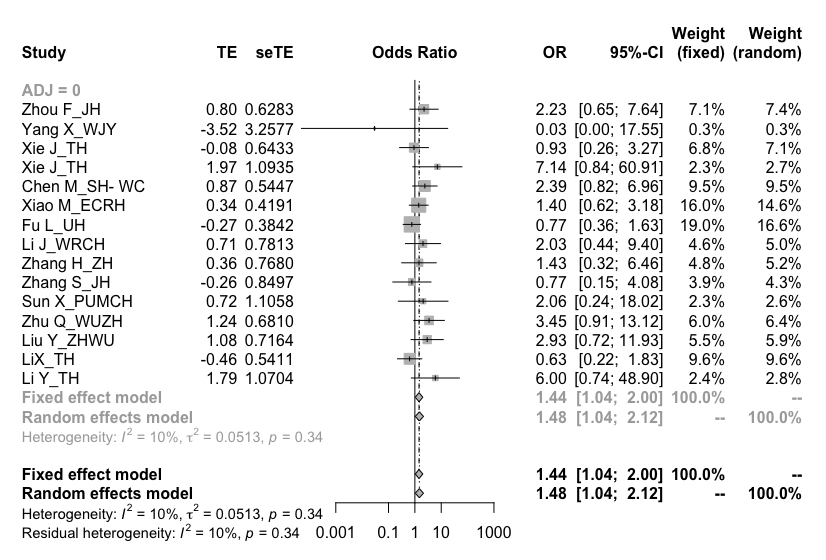 |

| Candidate variable: Cardiovascular disease (coronary heart disease or congestive heart failure), outcome: mortality, subgroup analysis by risk of bias: (moderate/high vs low) |
| --- |
| 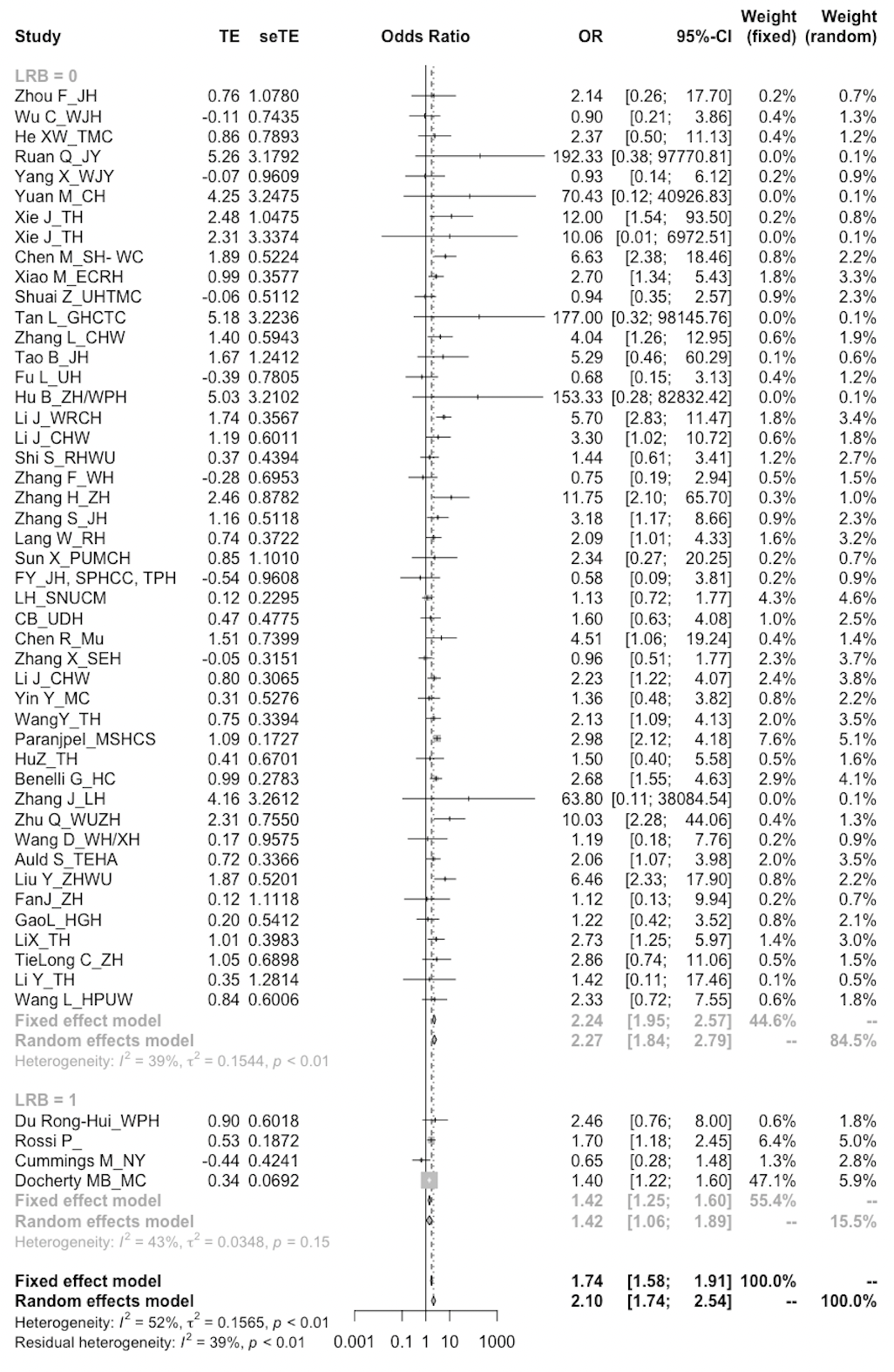 |

| Candidate variable: Cardiac arrhythmia (as previous condition or new clinical finding), outcome: mortality, subgroup analysis: (crude vs adjusted) |
| --- |
| 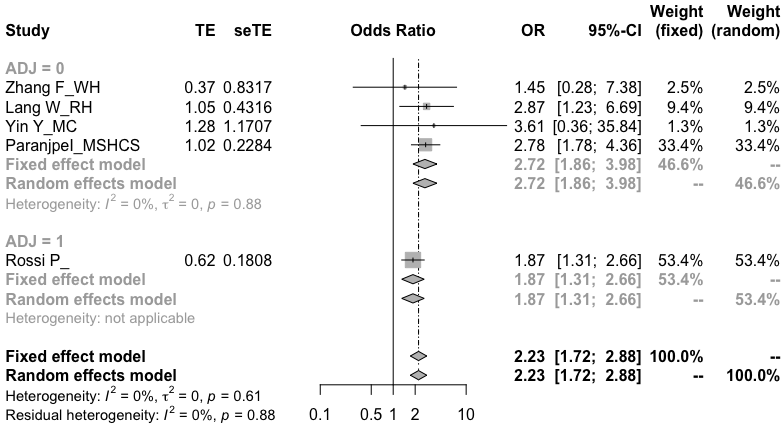 |

| Candidate variable: Cerebrovascular disease (History of stroke or CNS disease), outcome: mortality, subgroup analysis by risk of bias: (high vs moderate/low) |
| --- |
| 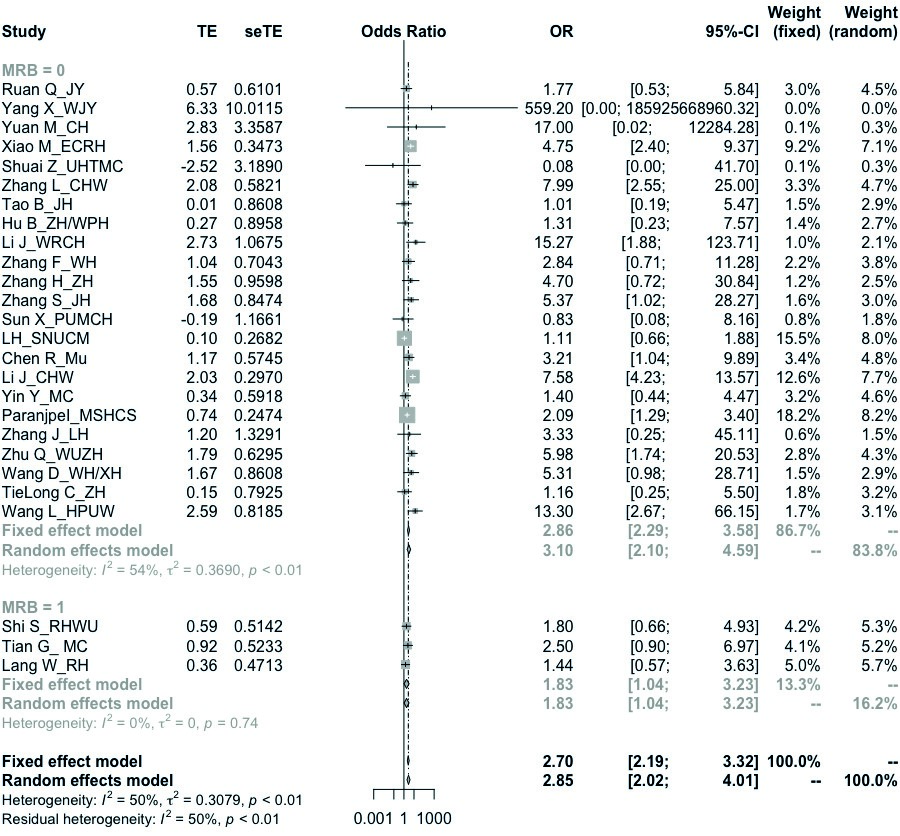 |

| Candidate variable: Diabetes, outcome: mortality, subgroup analysis by risk of bias: (moderate/high vs low) |
| --- |
| 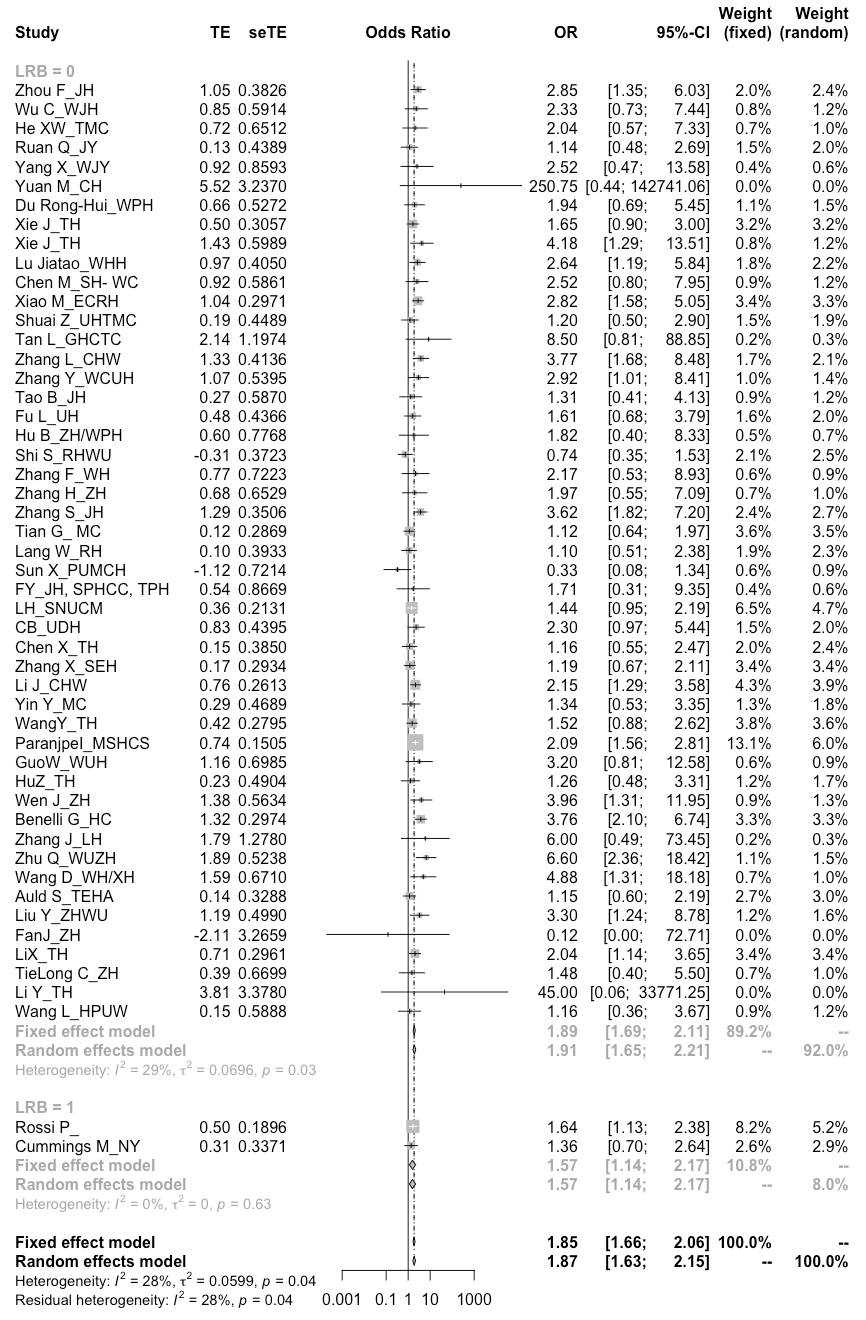 |

| Candidate variable: arterial hypertension, outcome: mortality, subgroup analysis by risk of bias: (moderate/high vs low) |
| --- |
| 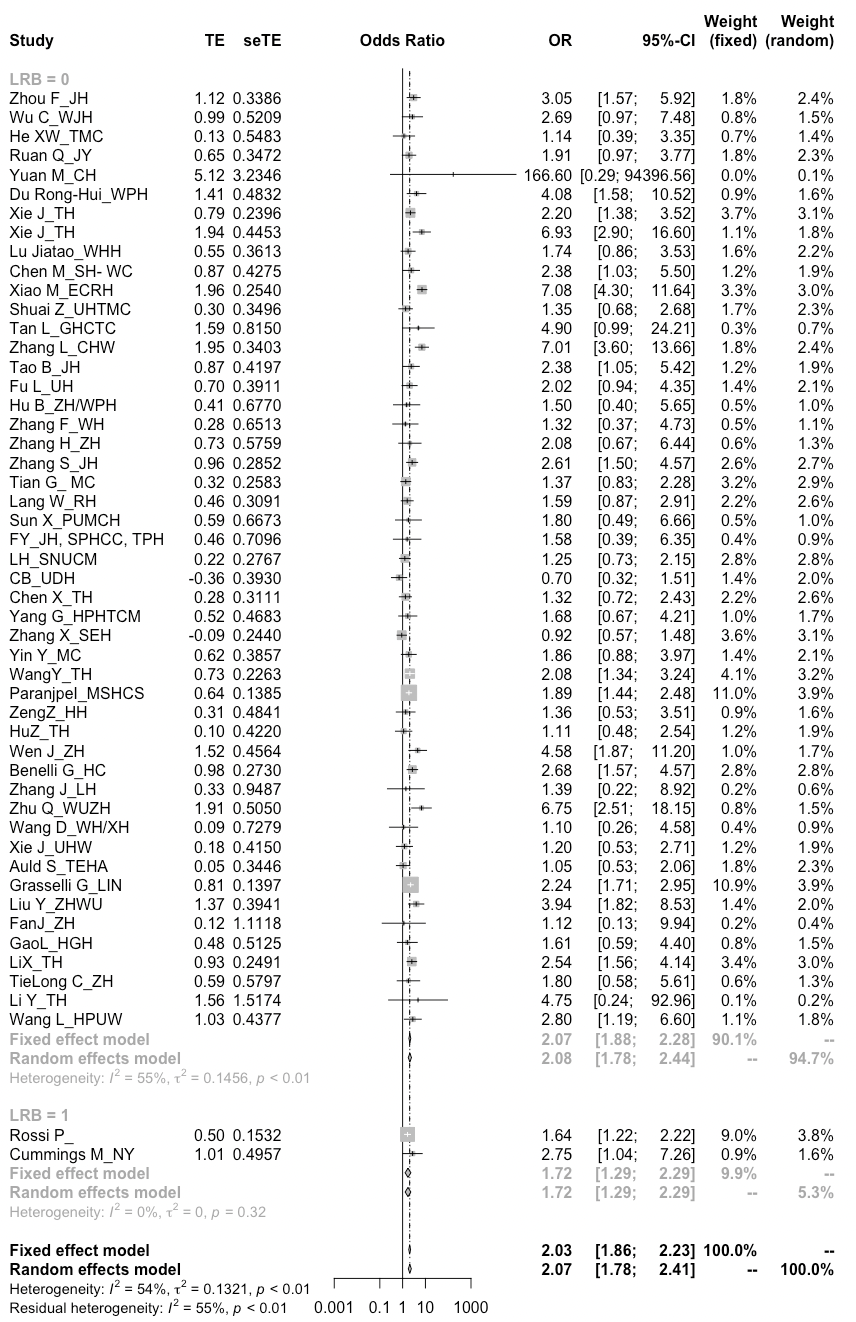 |

| Candidate variable: obesity, outcome: mortality, subgroup analysis: (crude vs adjusted) |
| --- |
| 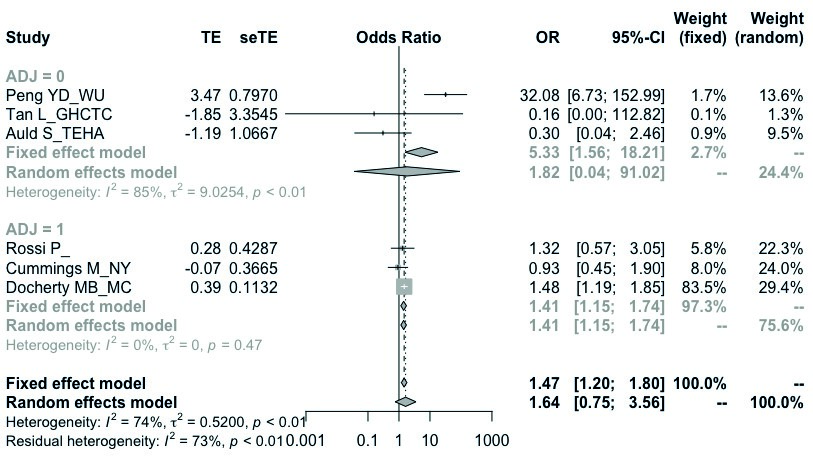 |

| Candidate variable: chronic kidney disease, outcome: mortality, subgroup analysis by risk of bias: (moderate/high vs low) |
| --- |
| 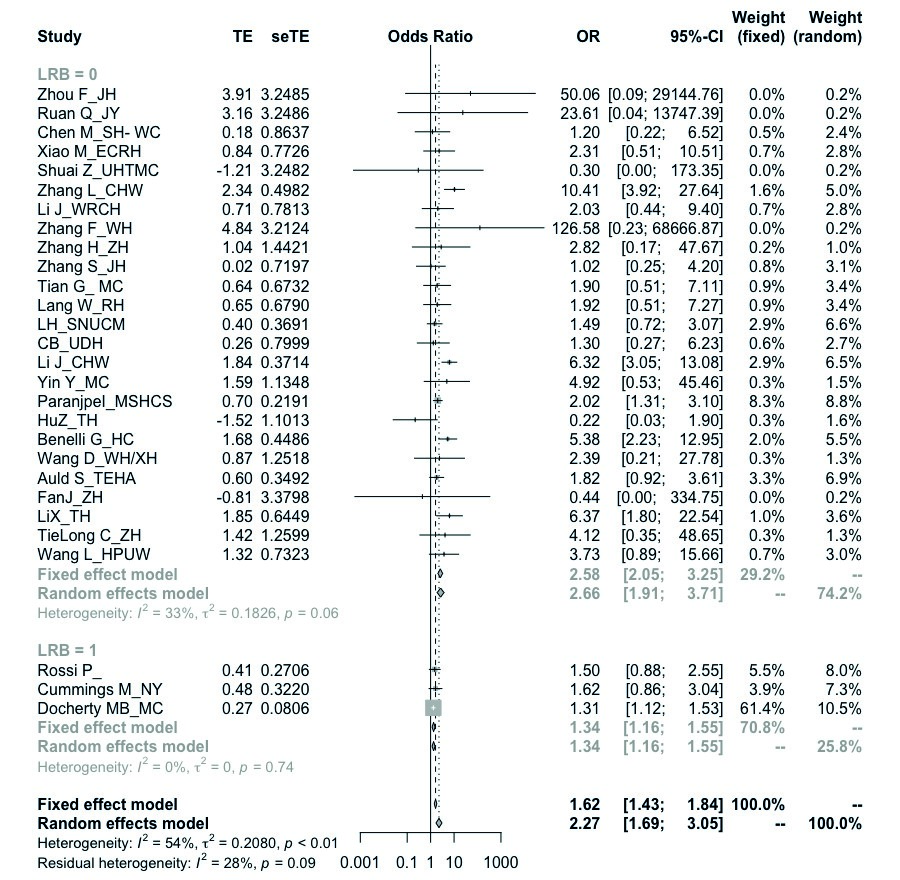 |

| Candidate variable: asthma, outcome: mortality, subgroup analysis: (crude vs adjusted) |
| --- |
| 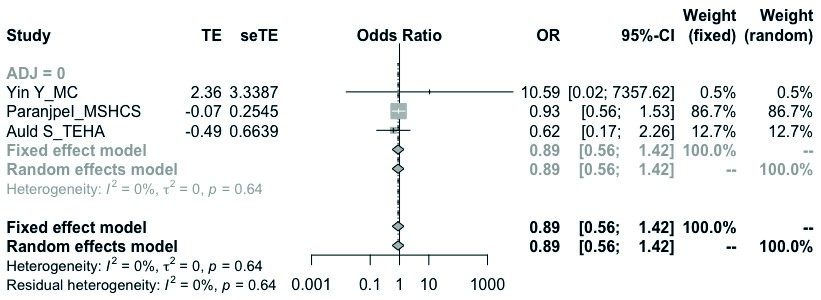 |

| Candidate variable: COPD, outcome: mortality, subgroup analysis by risk of bias: (moderate/high vs low) |
| --- |
| 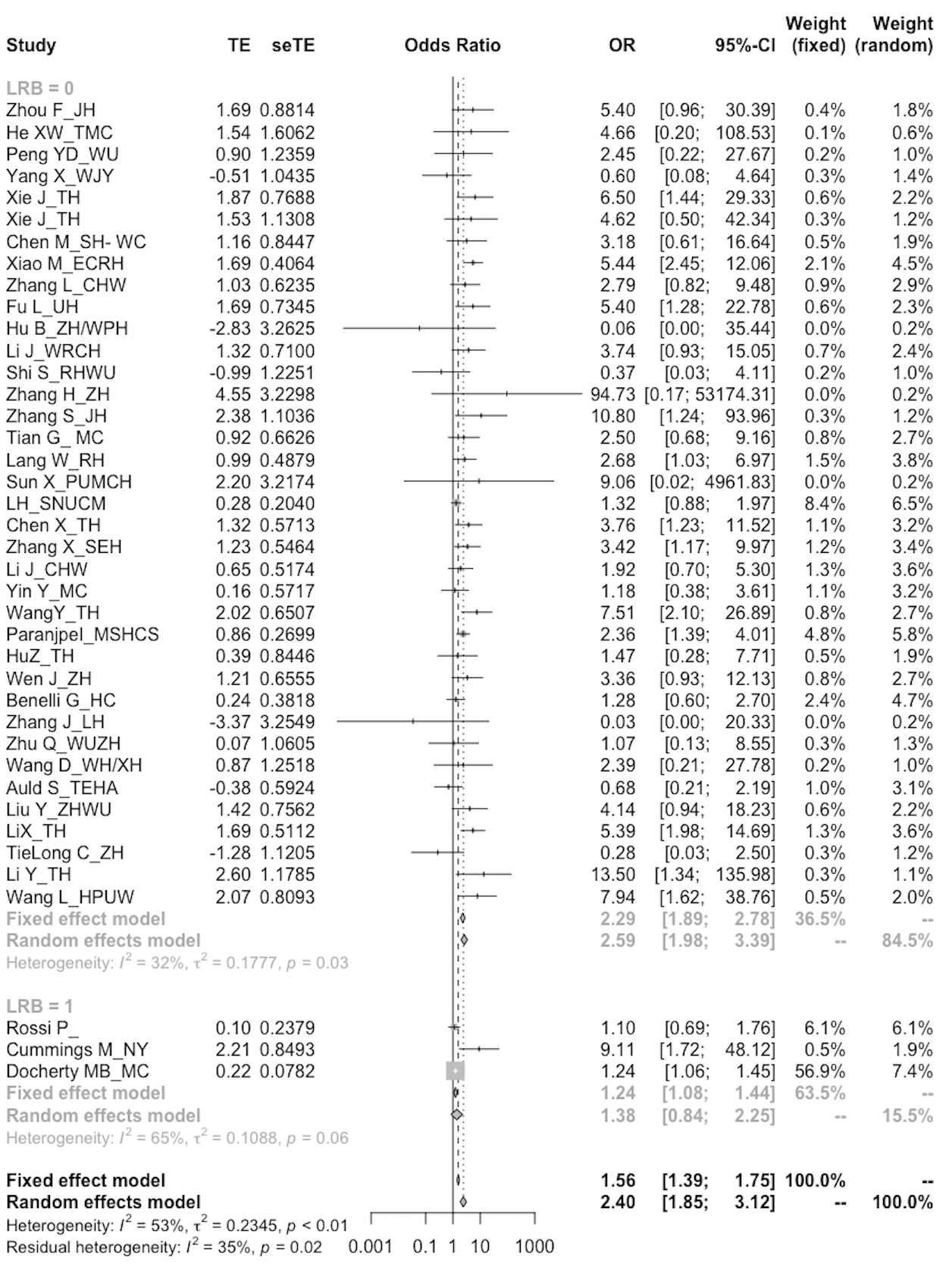 |

| Candidate variable: Tuberculosis, outcome: mortality |
| --- |
| 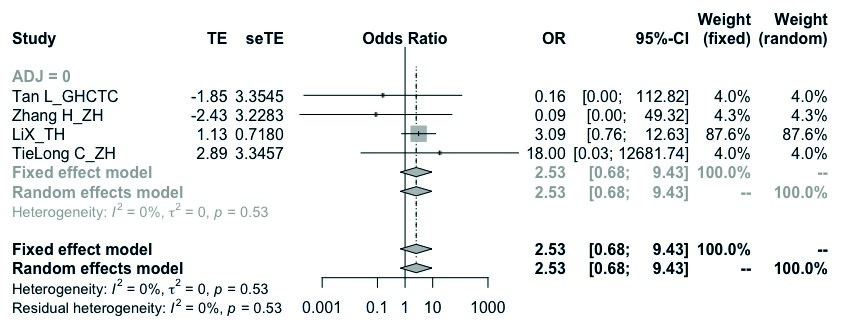 |

| Candidate variable: Cancer, solid or active haematologic cancer, outcome:  mortality, subgroup analysis by risk of bias: (moderate/high vs low) |
| --- |
| 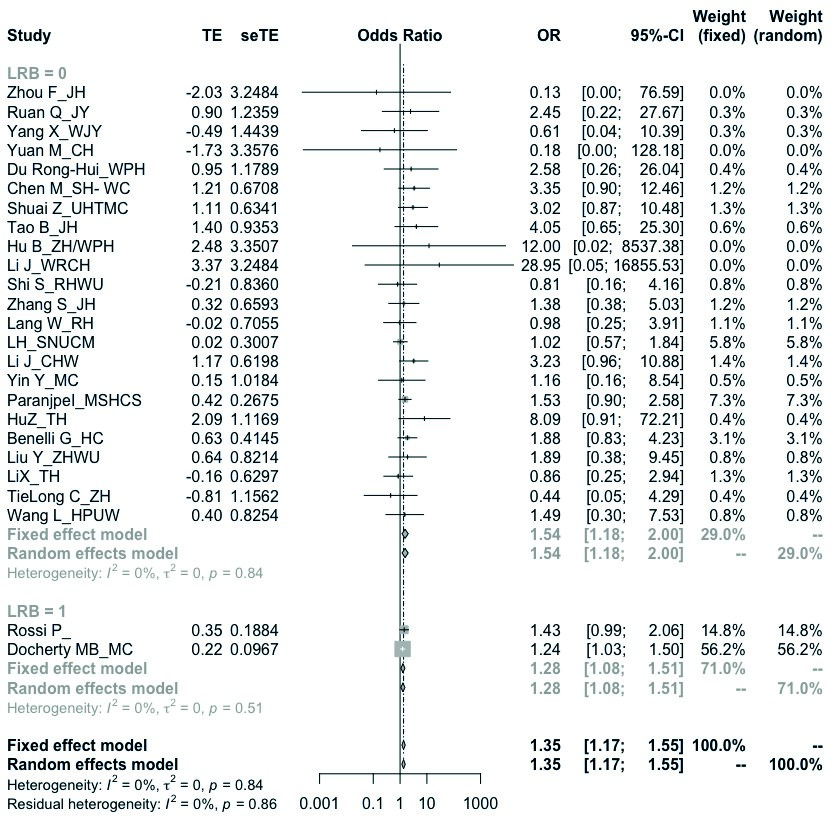 |

| Candidate variable: Immunocompromised, outcome: mortality |
| --- |
| 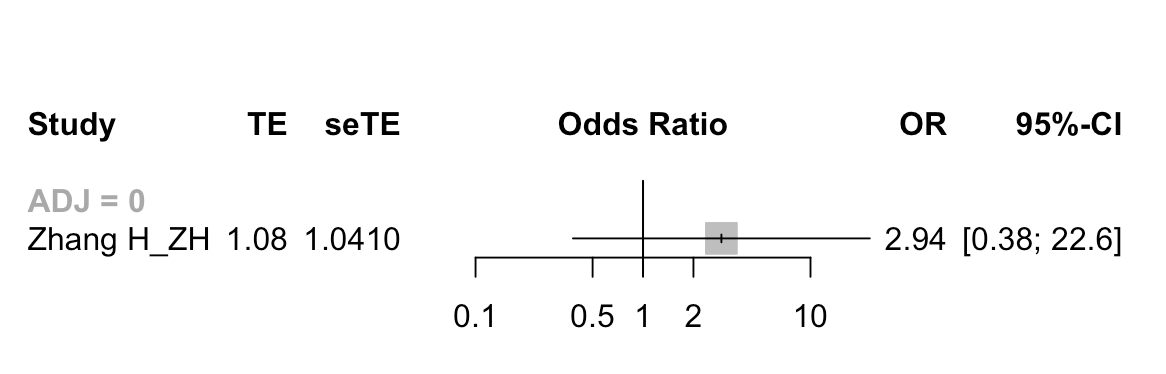 |

| Candidate variable: Autoimmune disease, outcome: mortality |
| --- |
| 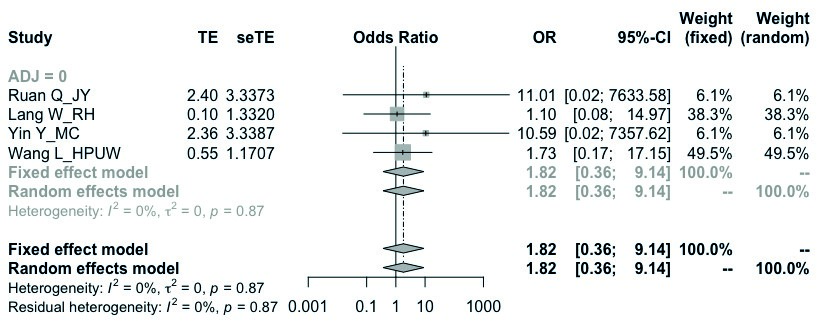 |
| Candidate variable: Dementia, outcome: mortality, subgroup analysis by risk of bias: (moderate/high vs low) |
| 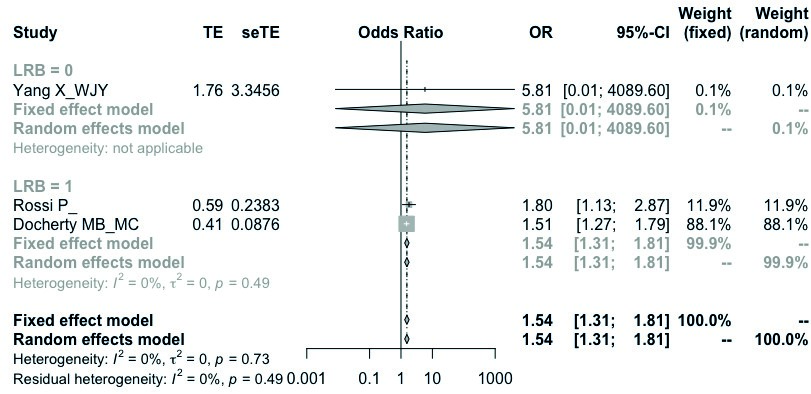 |

Candidate variable: Chronic liver disease, outcome: mortality


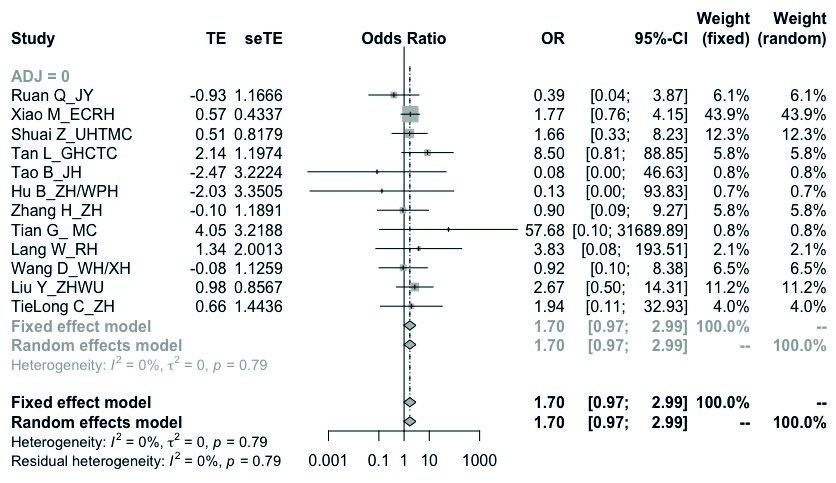


| Candidate variable: chronic gastric disease (history of peptic ulcer or gastritis), outcome: mortality |
| --- |
| 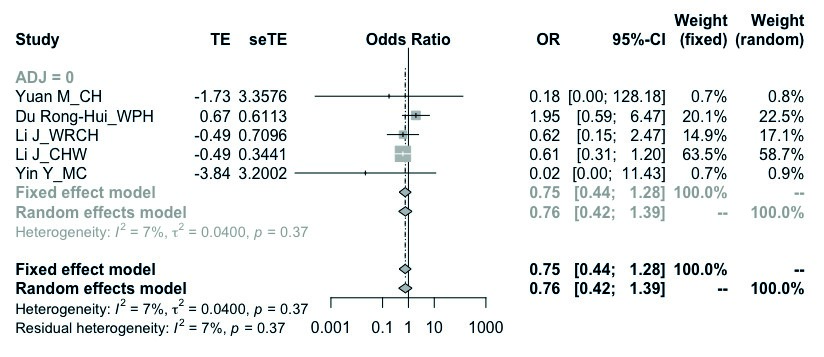 |

| Candidate variable: Dyslipidemia, outcome: mortality, subgroup analysis: (crude vs adjusted) |
| --- |
| 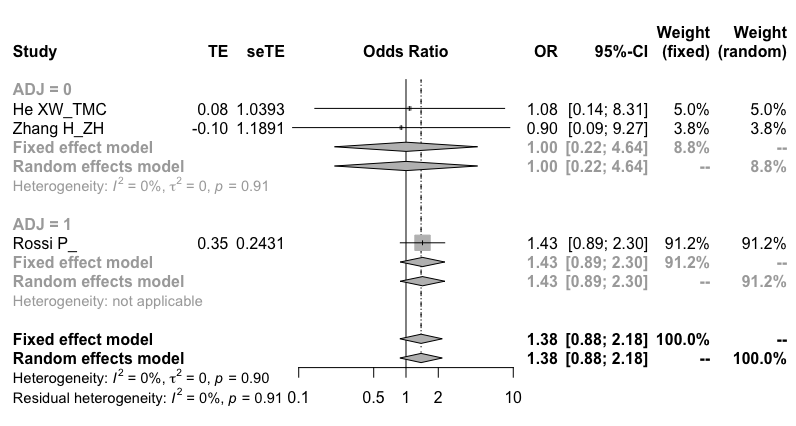 |

| Candidate variable: Any chronic condition or comorbidities, outcome:  mortality, subgroup analysis by risk of bias: (high vs moderate/low) |
| --- |
| 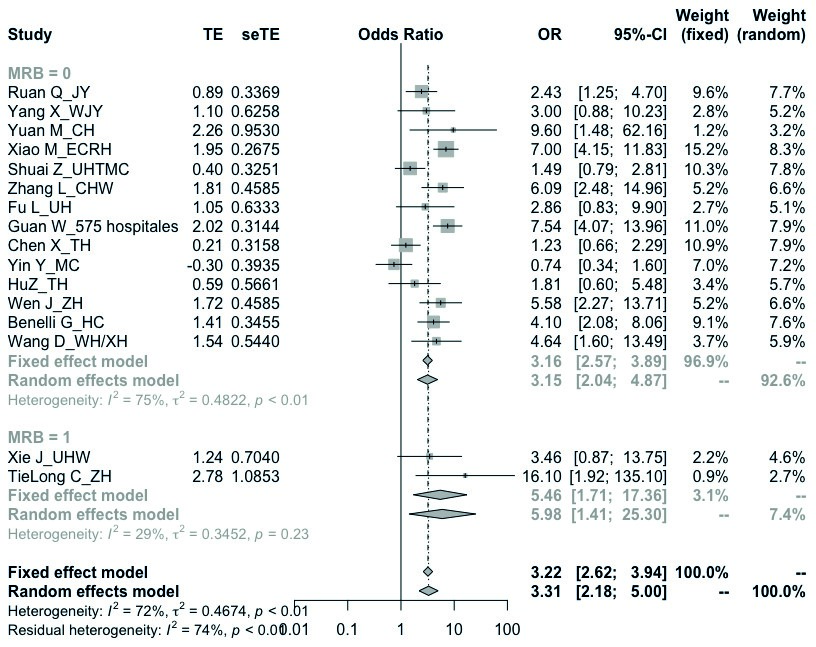 |
| Candidate variable: Respiratory failure, outcome: mortality, subgroup analysis by risk of bias: (high vs moderate/low) |
| 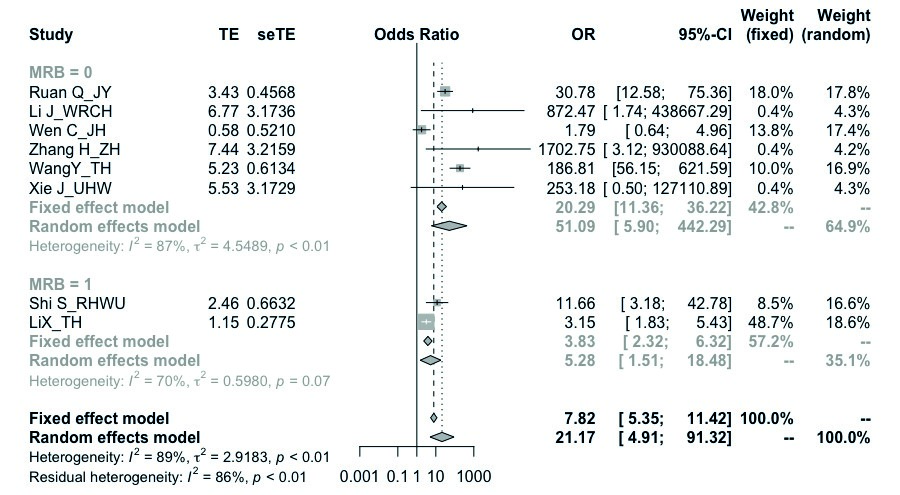 |

| Candidate variable: Tachypnea, outcome: mortality, subgroup analysis by risk of bias: (high vs moderate/low) |
| --- |
| 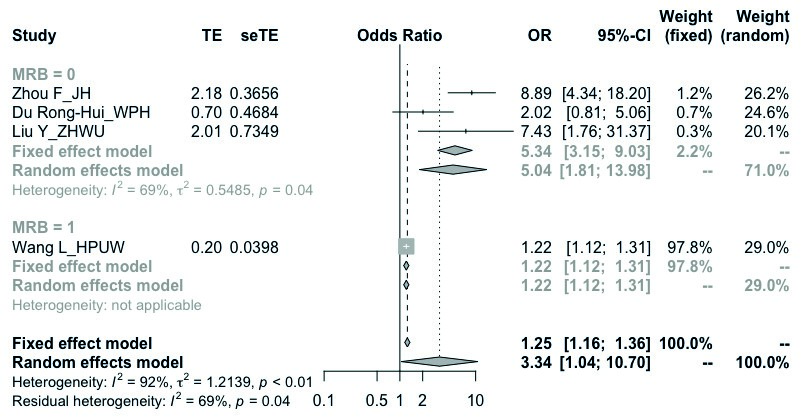 |
| Candidate variable: Hypoxemia, outcome: mortality, subgroup analysis by risk of bias: (high vs moderate/low) |
| 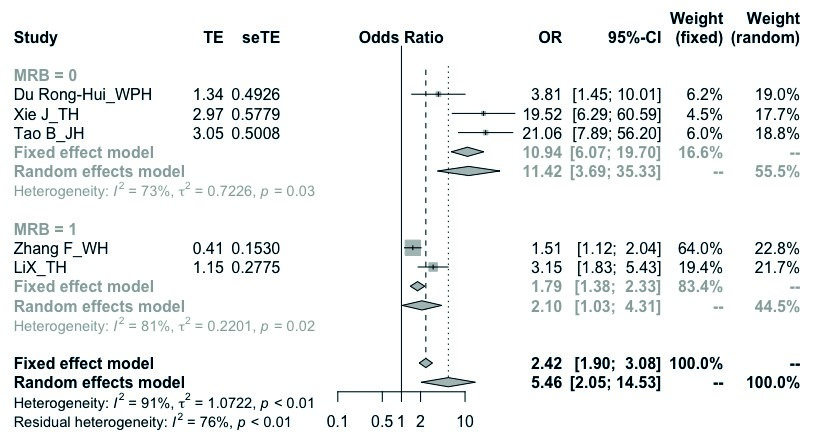 |

| Candidate variable: Dyspnea, outcome: mortality, subgroup analysis by risk of bias: (high vs moderate/low) |
| --- |
| 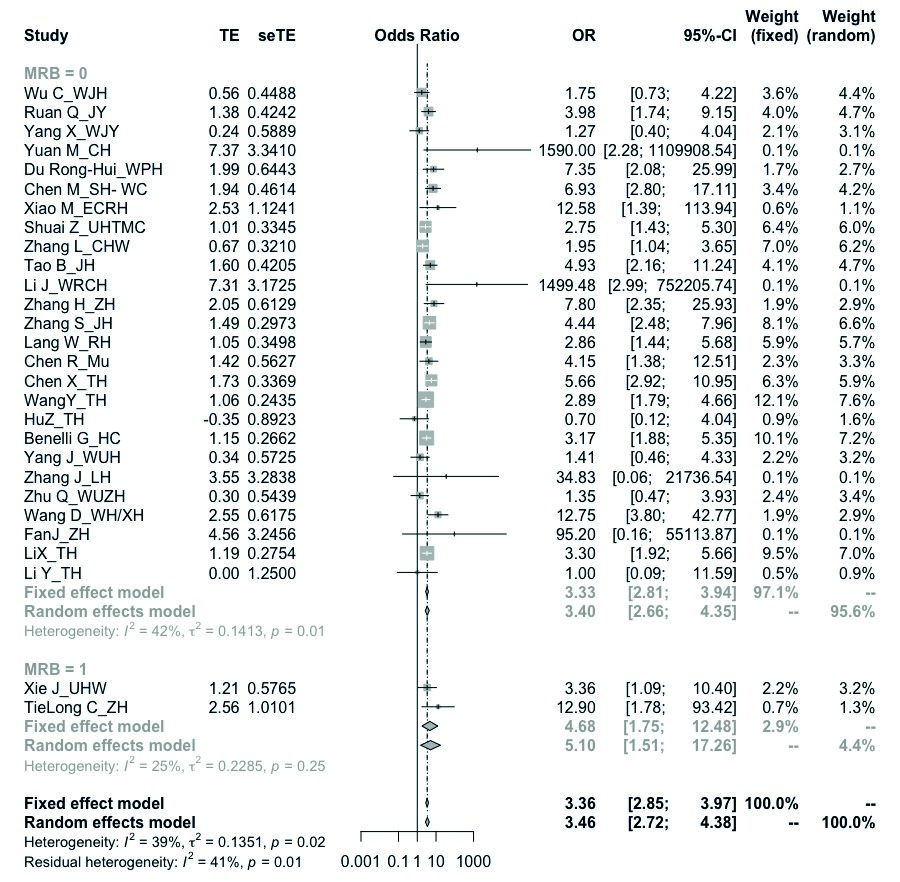 |

| Candidate variable: Chest pain, outcome: mortality, subgroup analysis: (crude vs adjusted) | |
| --- | --- |
| 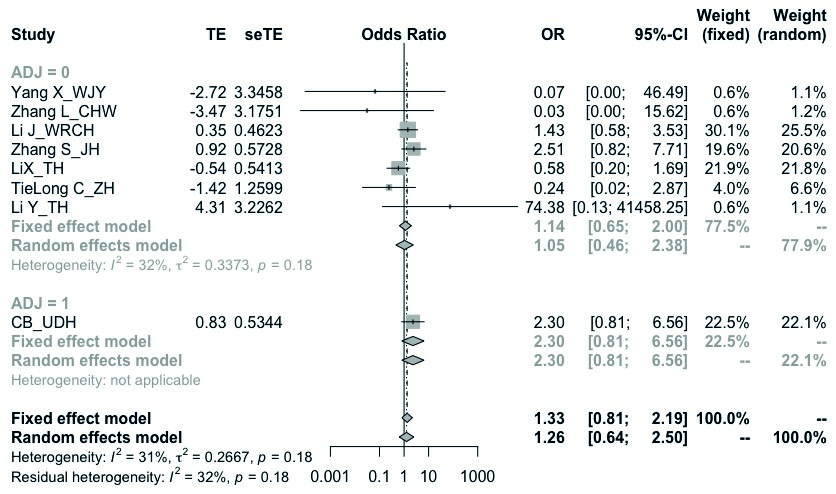 | |
| Candidate variable: Tachycardia, outcome: mortality, | |
| 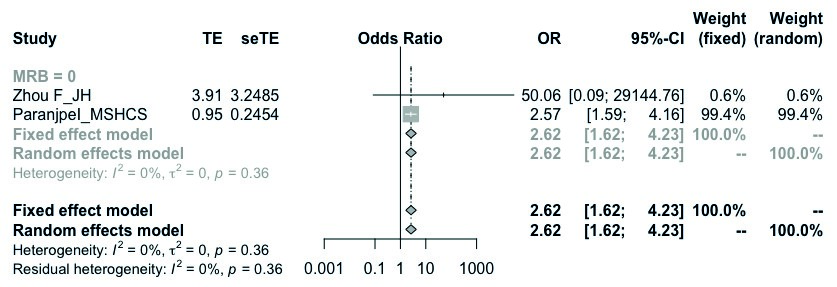 | |

| Candidate variable: Low blood pressure, outcome: mortality |
| --- |
| 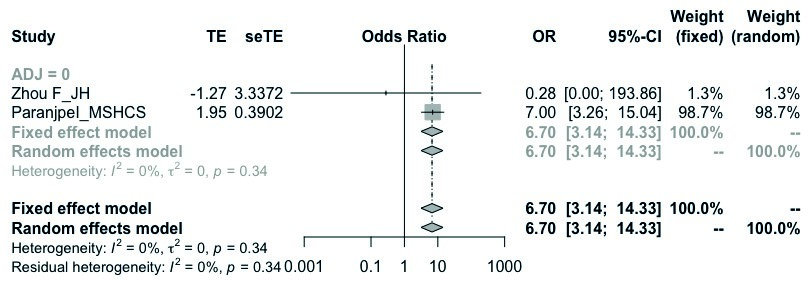 |
| Candidate variable: High fever (more than 39°C), outcome: mortality |
| 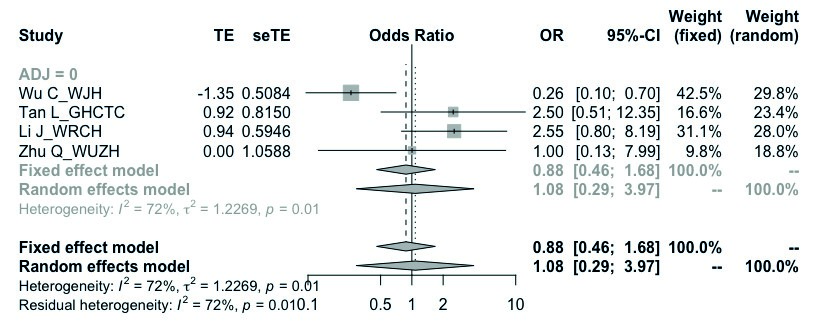 |

| Candidate variable: Fever, outcome: mortality, subgroup analysis: (crude vs adjusted) |
| --- |
| 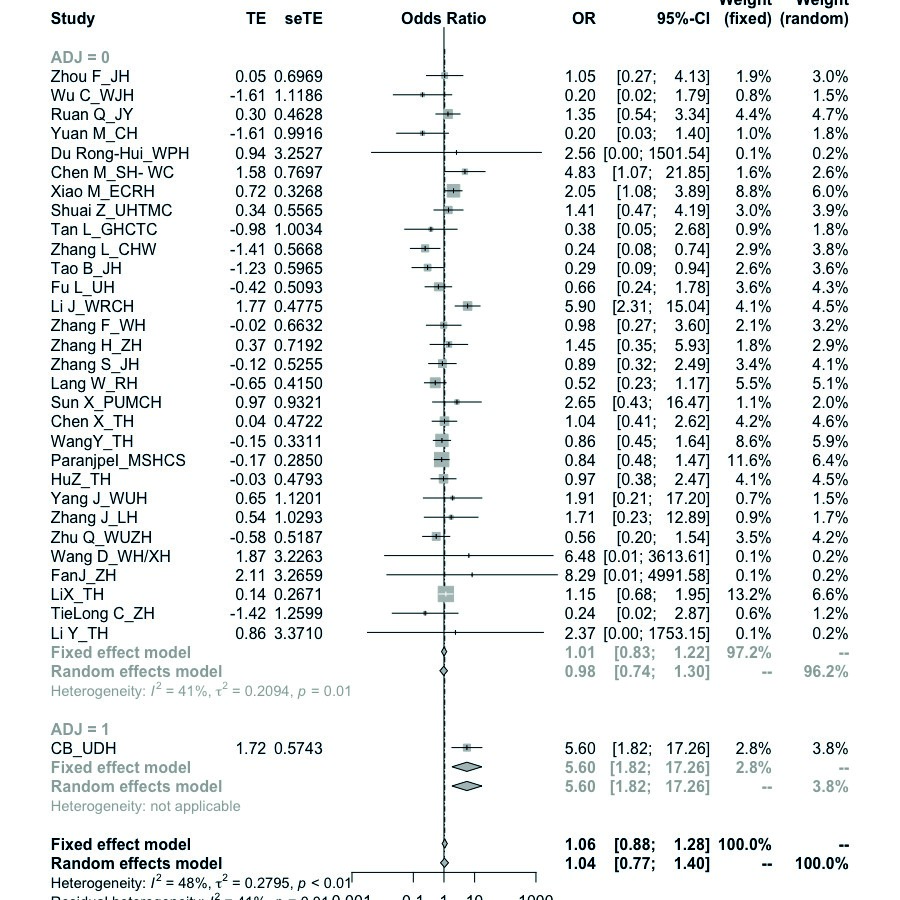 |

| Candidate variable: Rhinorrhea, outcome: mortality | |
| --- | --- |
| 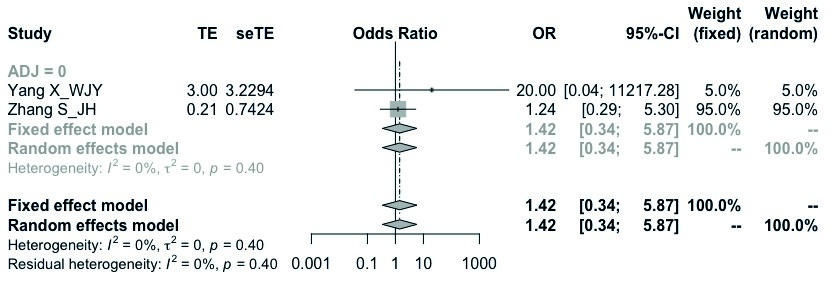 | |
| Candidate variable: Odynophagia, outcome: mortality | |
| 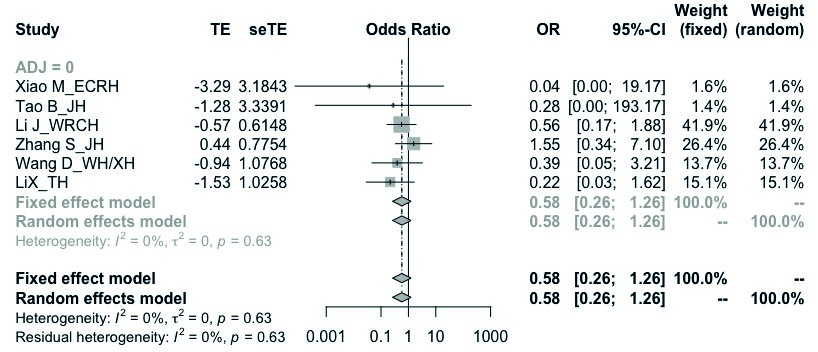 | |

| Candidate variable: cough, outcome: mortality, subgroup analysis by risk of bias: (high vs moderate/low) |
| --- |
| 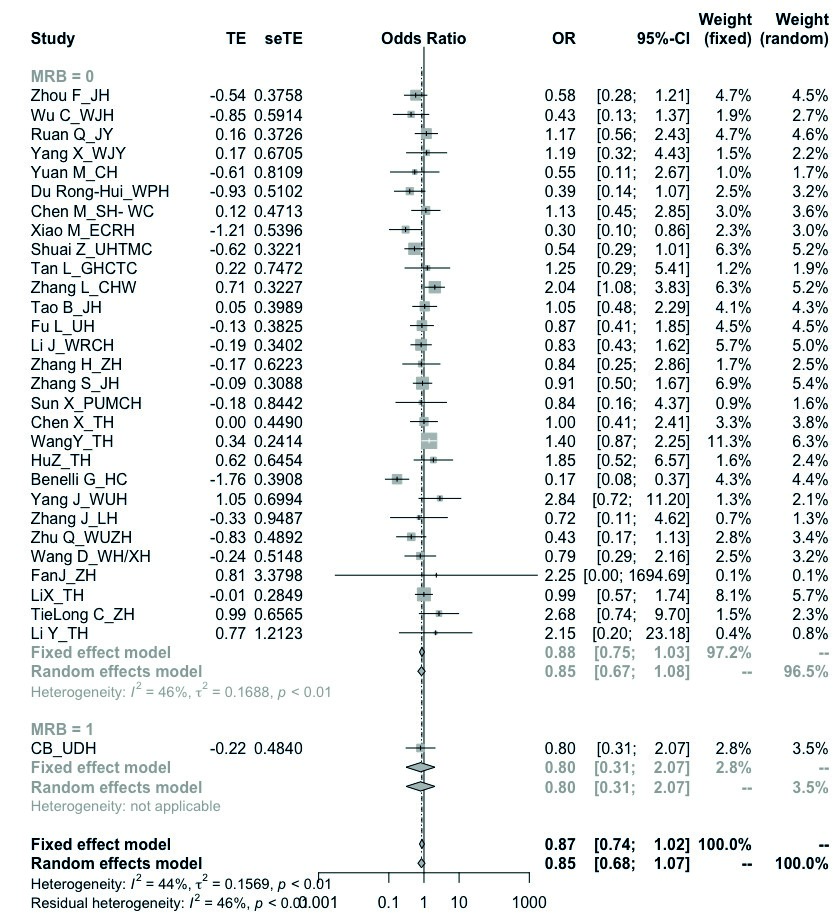 |

| Candidate variable: productive cough, outcome: mortality, subgroup analysis: (crude vs adjusted) | |
| --- | --- |
| 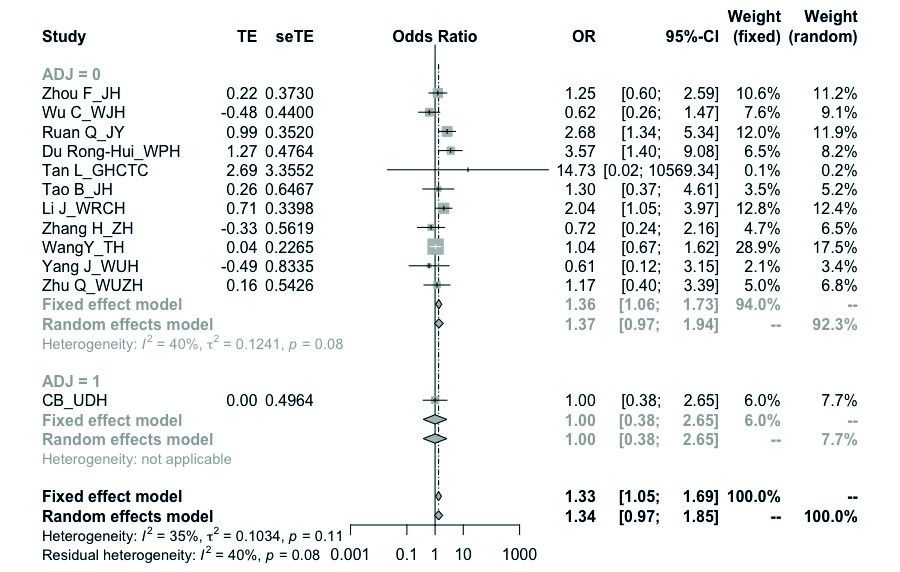 | |
| Candidate variable: hemoptysis, outcome: mortality | |
| 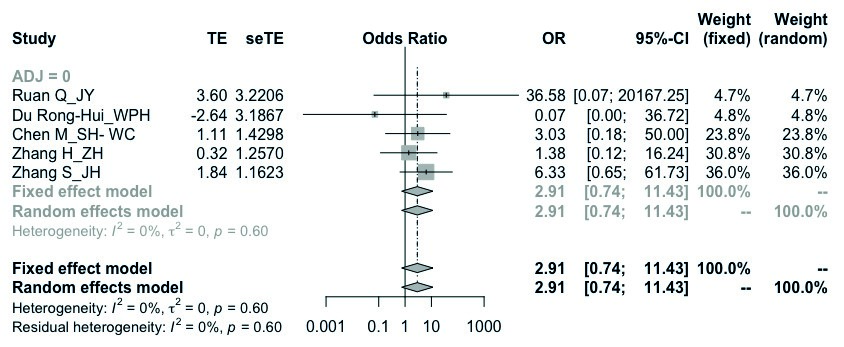 | |

| Candidate variable: fatigue, outcome: mortality |
| --- |
| 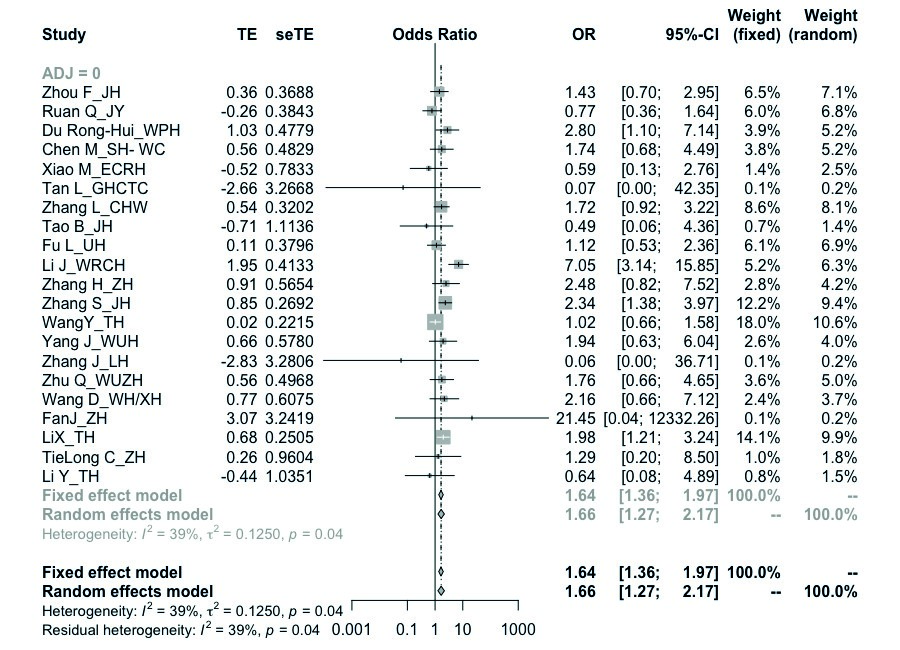 |

| Candidate variable: myalgia/arthralgia, outcome: mortality, subgroup analysis: (crude vs adjusted) |
| --- |
| 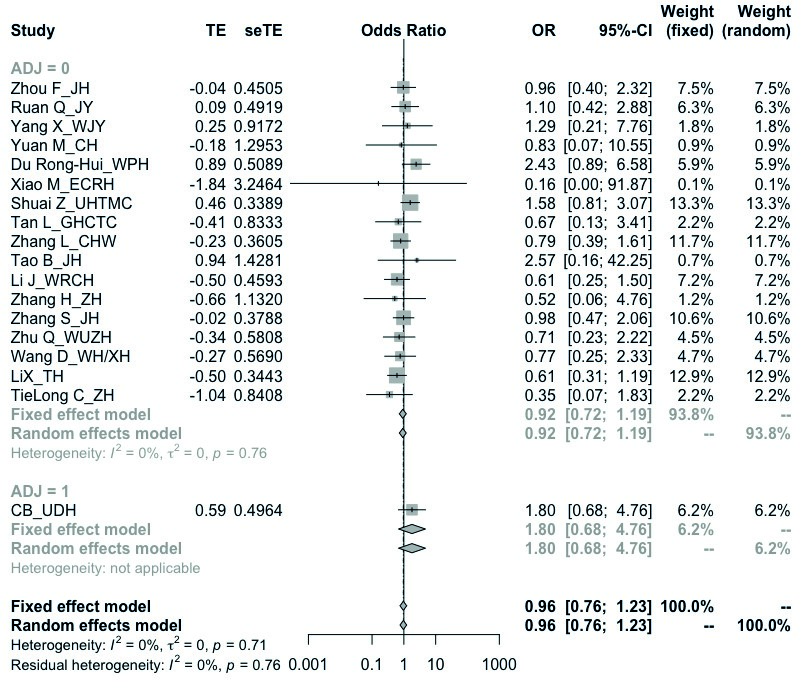 |

| Candidate variable: headache, outcome: mortality, subgroup analysis: (crude vs adjusted) |
| --- |
| 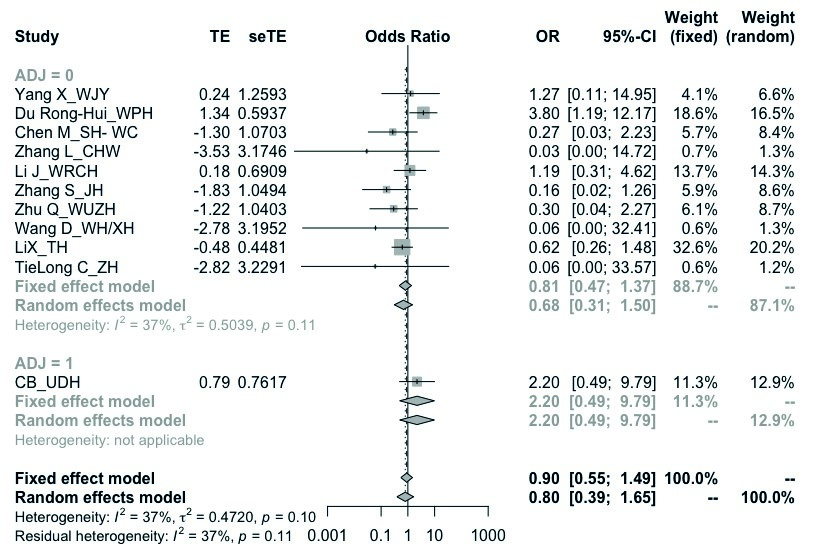 |

| Candidate variable: vomits, outcome: mortality | |
| --- | --- |
| 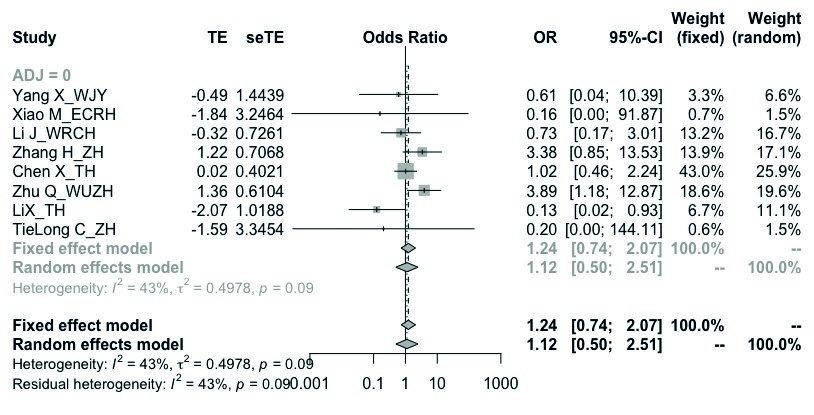 | |
| Candidate variable: diarrhea, outcome: mortality | |
| 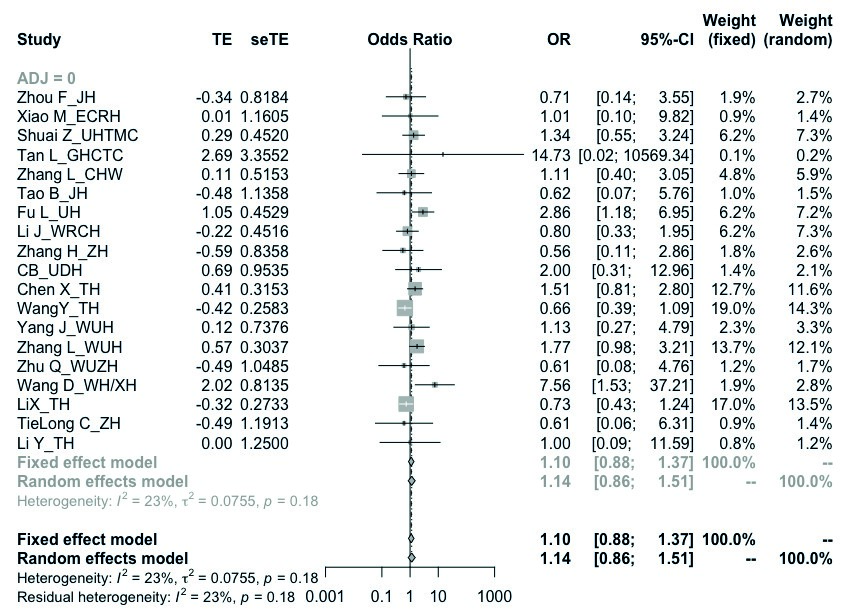 | |

| Candidate variable: anorexia, outcome: mortality, subgroup analysis: (crude vs adjusted) | |
| --- | --- |
| 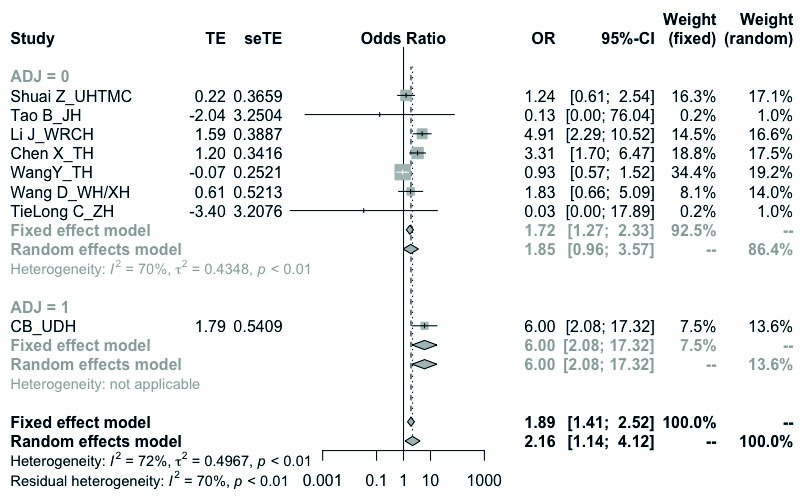 | |
| Candidate variable: abdominal pain, outcome: mortality | |
| 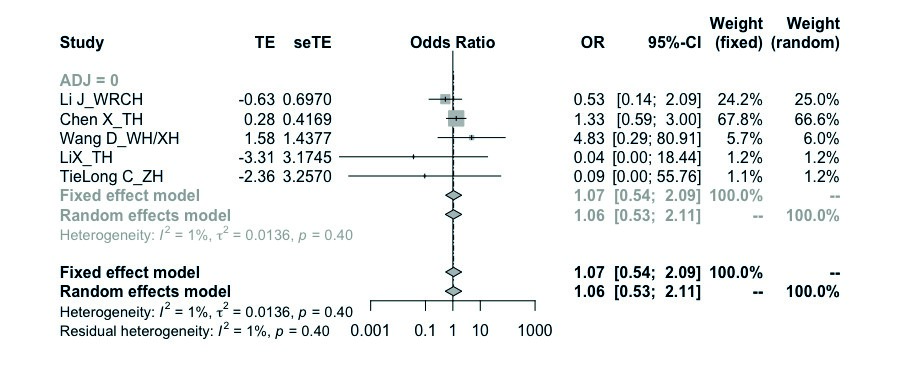 | |

| Candidate variable: anemia, outcome: mortality | |
| --- | --- |
| 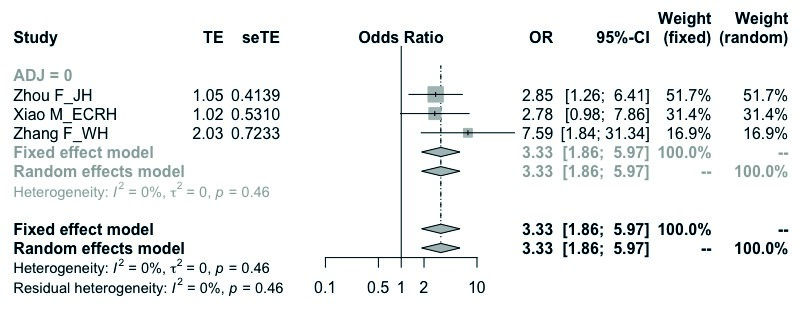 | |
| Candidate variable: High WBC (greater than 10.0 x 109/L), outcome: mortality, subgroup analysis by risk of bias: (high vs moderate/low) | |
| 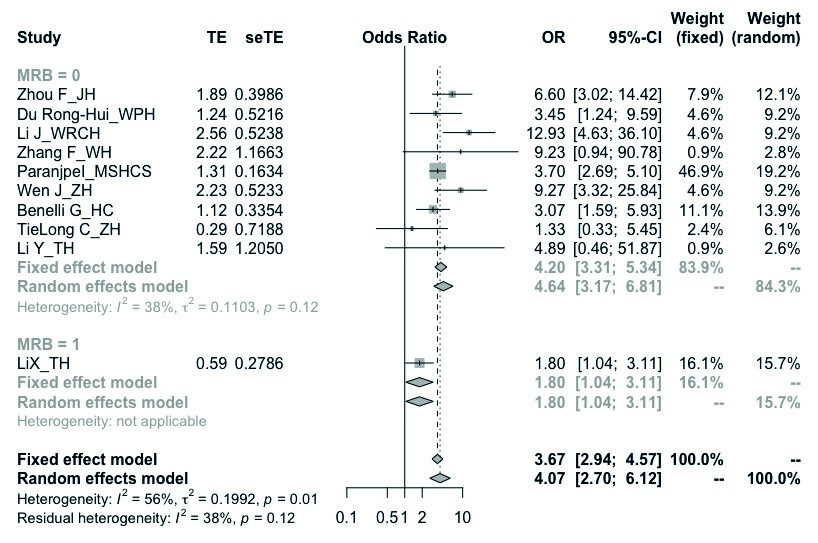 | |

| Candidate variable: High Neutrophil count (greater than 6.3 x 109/L), outcome: mortality | |
| --- | --- |
| 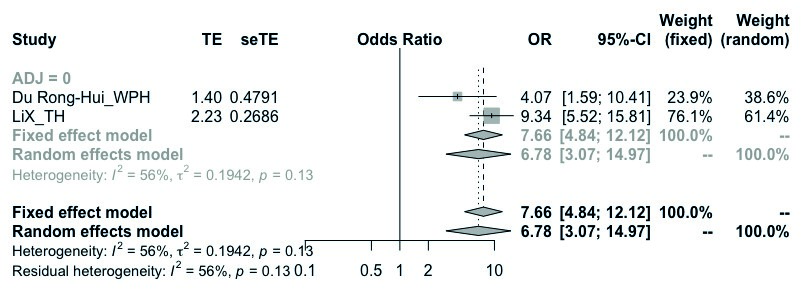 | |
| Candidate variable: Neutrophil count increase(per 1 x 109 U/L), outcome:  mortality, subgroup analysis by risk of bias: (high vs moderate/low) | |
| 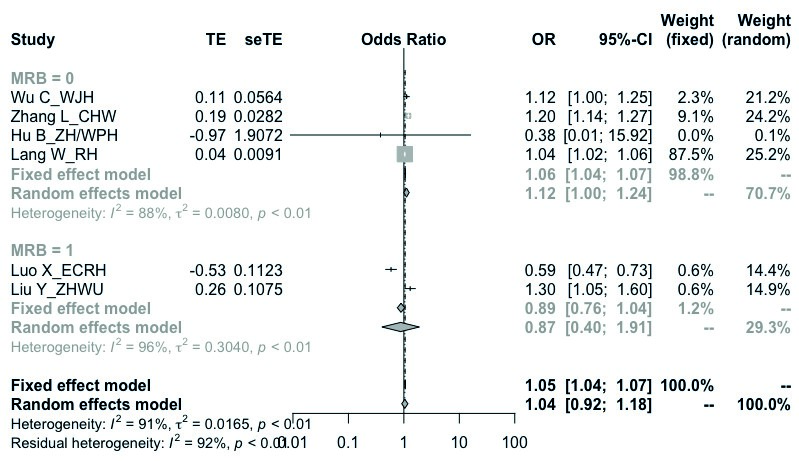 | |

| Candidate variable: Low neutrophil count (less than 1.8 x 109/L), outcome:  mortality | |
| --- | --- |
| 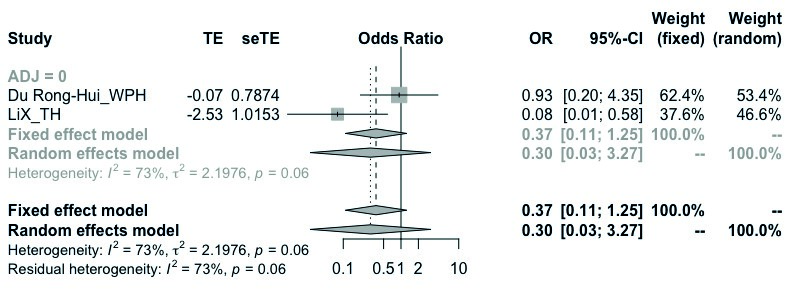 | |
| Candidate variable: Leukopenia (less than 3.5-4 x 109/L), outcome: mortality, subgroup analysis: (crude vs adjusted) | |
| 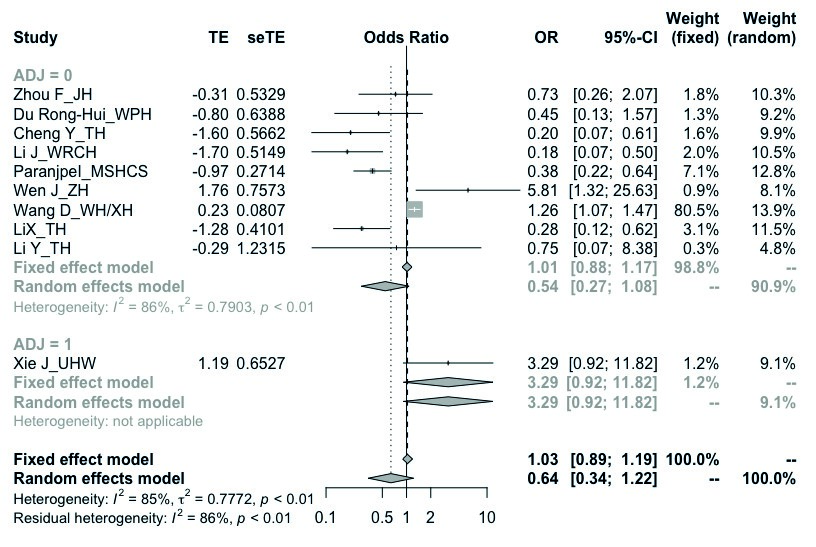 | |

| Candidate variable: Low Lymphocyte count (less than 0.8-1.5x 109/L), outcome: mortality, subgroup analysis: (crude vs adjusted) |
| --- |
| 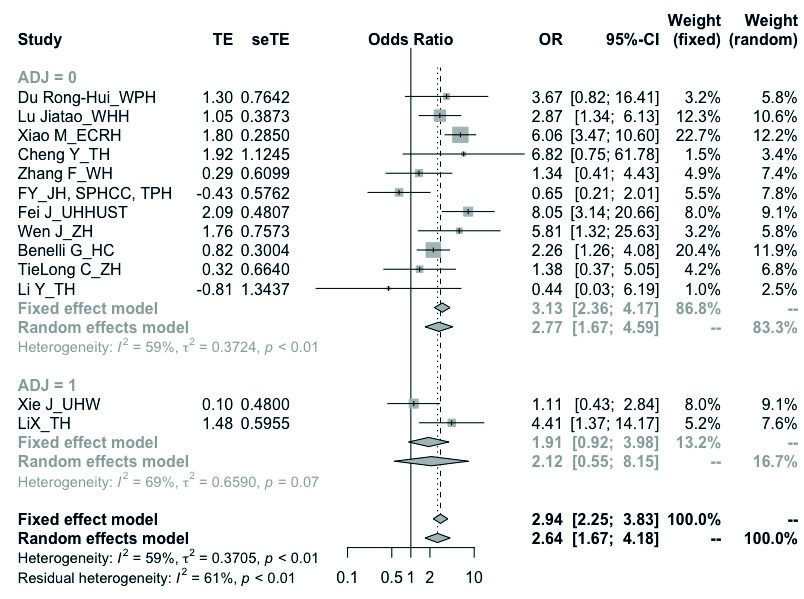 |

| Candidate variable: Lymphocyte count increase (per 1 x 109 U/L), outcome: mortality, subgroup analysis: (crude vs adjusted) |
| --- |
| 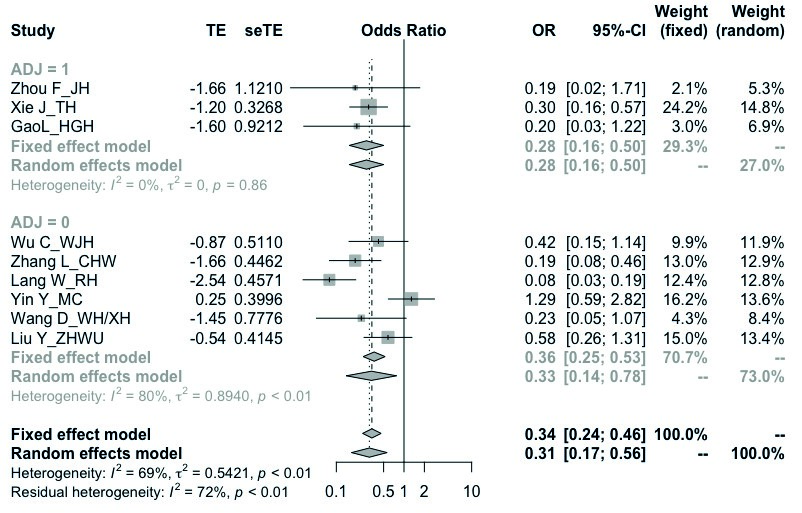 |

| Candidate variable: Low platelet count (less than 100-150 x 109/L), outcome:  mortality, subgroup analysis by risk of bias: (high vs moderate/low) |
| --- |
| 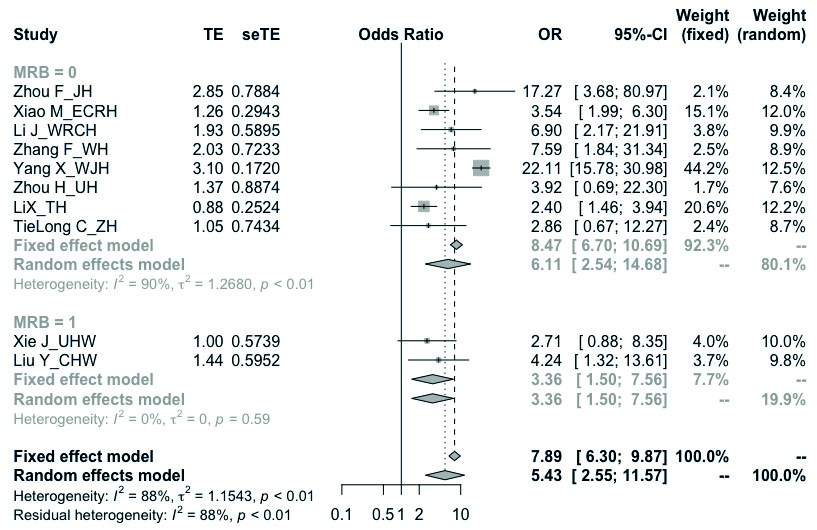 |

| Candidate variable: High plasma creatinine (more than 1.5 mg%), outcome: mortality, subgroup analysis by risk of bias: (high vs moderate/low) |
| --- |
| 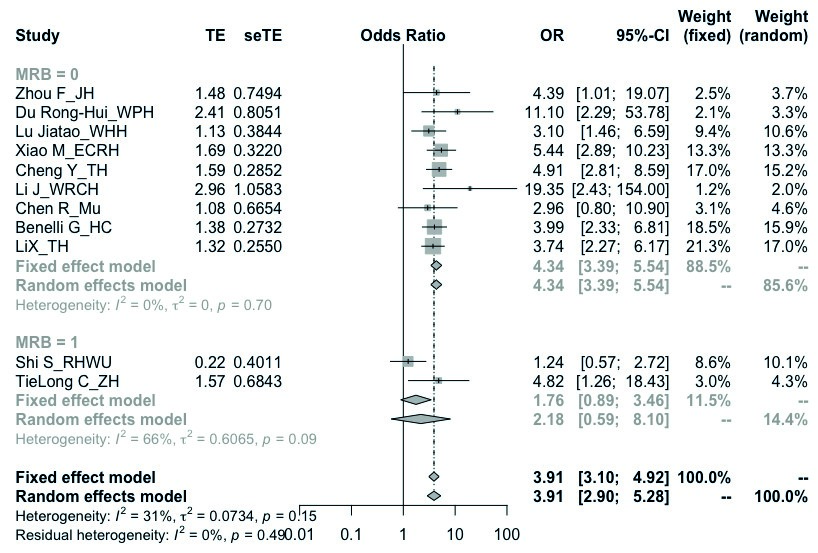 |

| Candidate variable: Creatinine increase (per 0.1 mg%), outcome: mortality, subgroup analysis by risk of bias: (high vs moderate/low) |
| --- |
| 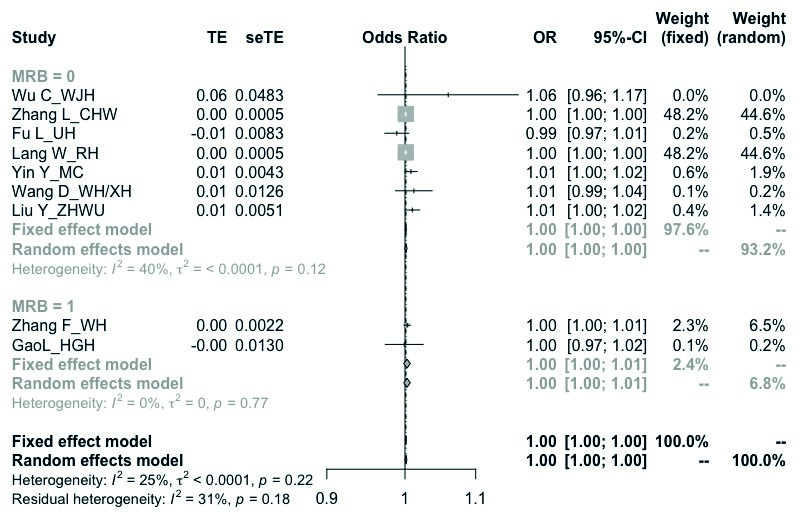 |

| Candidate variable: Acute kidney injury, outcome: mortality, subgroup analysis: (crude vs adjusted) |
| --- |
| 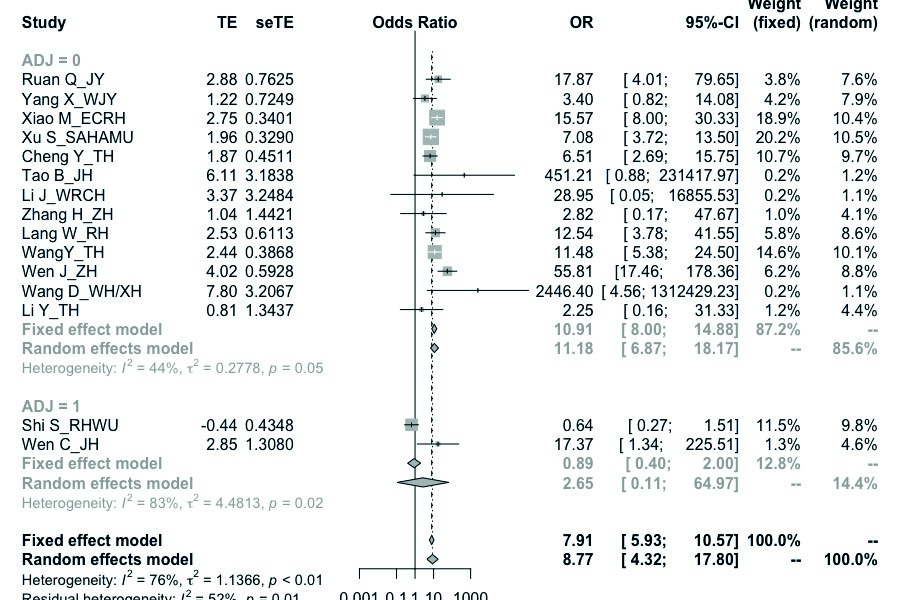 |

| Candidate variable: High BUN (more than 5.2-9.5 mmol/L), outcome: mortality | |
| --- | --- |
| 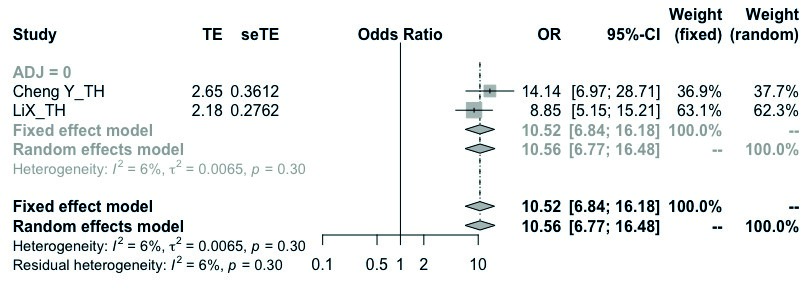 | |
| Candidate variable: High LDH (more than 240-250 U/L), outcome: mortality, subgroup analysis by risk of bias: (high vs moderate/low) | |
| 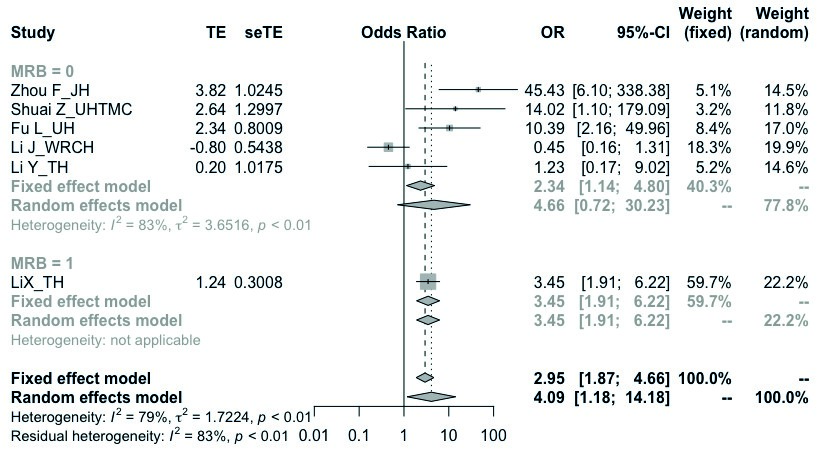 | |

| Candidate variable: LDH increase (per 1 U/L), outcome: mortality, subgroup analysis by risk of bias: (high vs moderate/low) | |
| --- | --- |
| 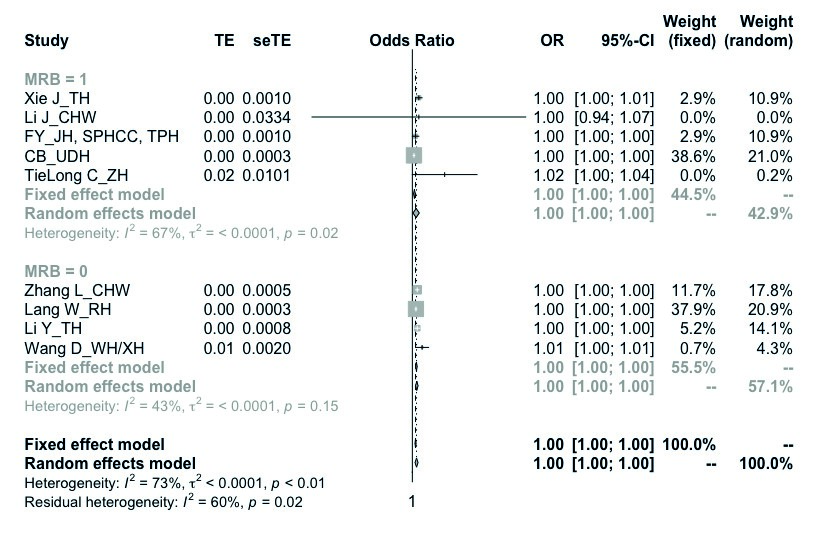 | |
| Candidate variable: High CK (more than 185-200 U/L), outcome: mortality | |
| 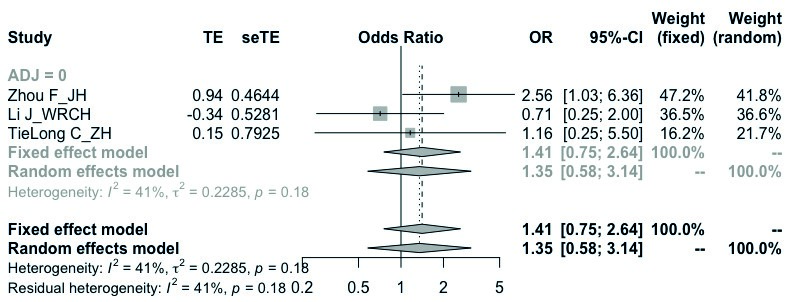 | |

| Candidate variable: Myocardial injury, outcome: mortality, subgroup analysis by risk of bias: (moderate/high vs low) |
| --- |
| 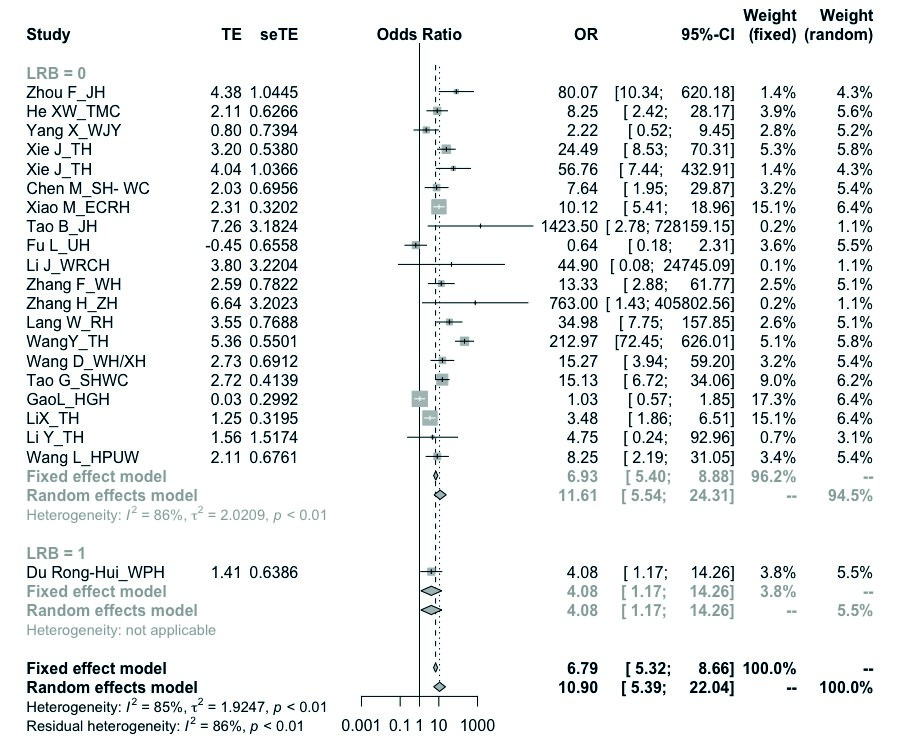 |

| Candidate variable: CK-MB increase (per 1 U/L), outcome: mortality, subgroup analysis: (crude vs adjusted) |
| --- |
| 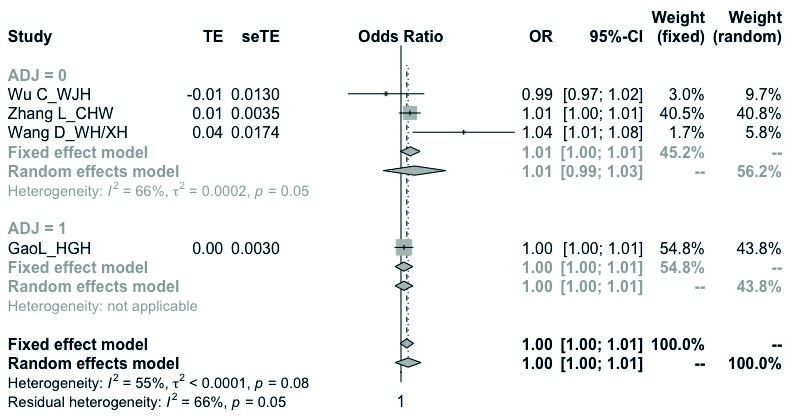 |
| Candidate variable: Urea increase (per 1 mmol/L), outcome: mortality, subgroup analysis: (crude vs adjusted) |
| 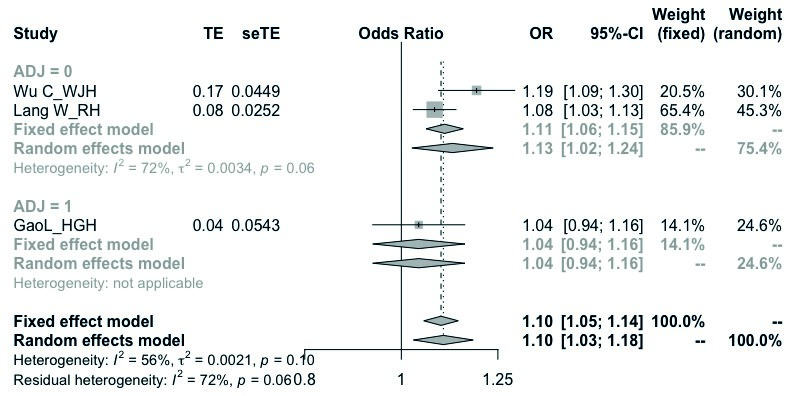 |

| Candidate variable: High BNP (more than 500-900 pg/mL), outcome: mortality, subgroup analysis: (crude vs adjusted) |
| --- |
| 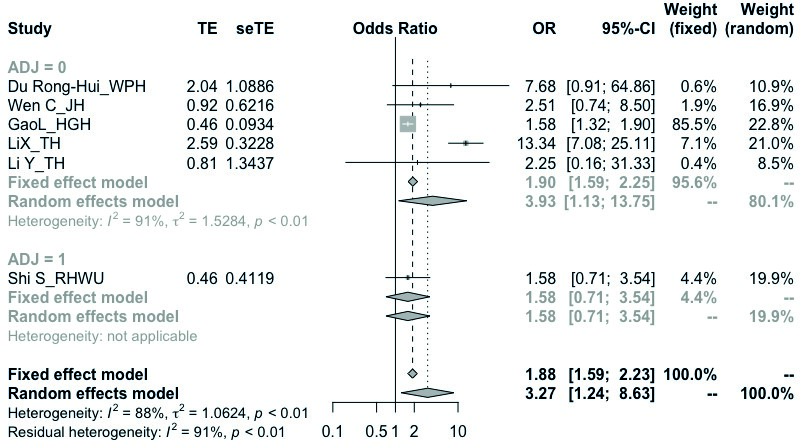 |

| Candidate variable: High D-dimer (more than 500-1000 ng/ml), outcome: mortality, subgroup analysis by risk of bias: (high vs moderate/low) | |
| --- | --- |
| 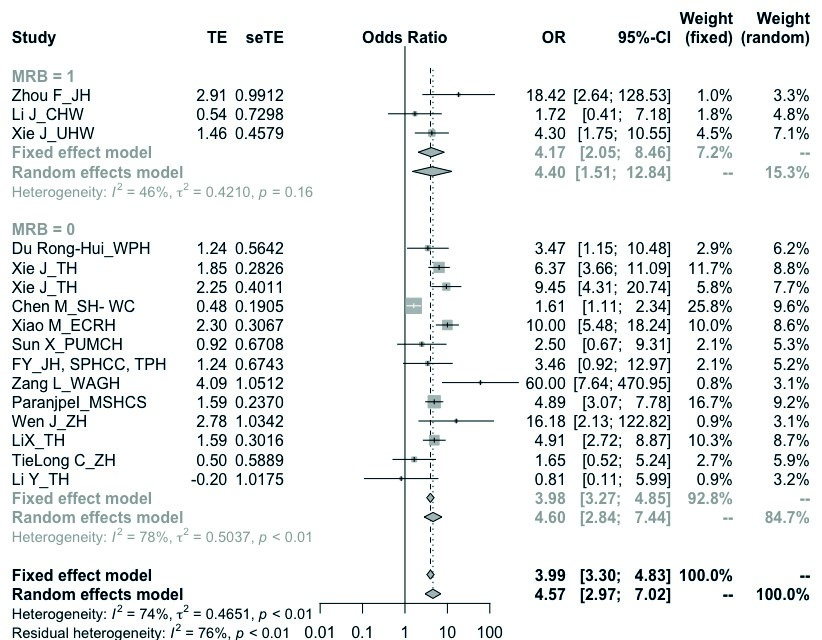 | |
| Candidate variable: D-dimer increase (per 10 ng/mL), outcome: mortality, subgroup analysis by risk of bias: (moderate/high vs low) | |
| 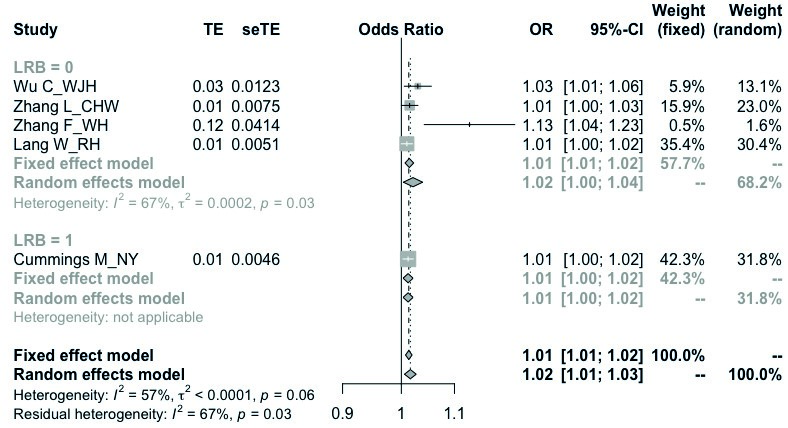 | |

| Candidate variable: Prolonged PT (more than 13.2-15 seconds), outcome: mortality, subgroup analysis: (crude vs adjusted) | |
| --- | --- |
| 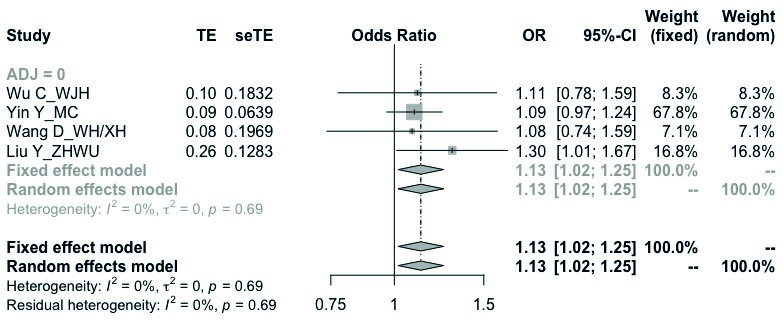 | |
| Candidate variable: PT increase (per 1 second), outcome: mortality | |
| 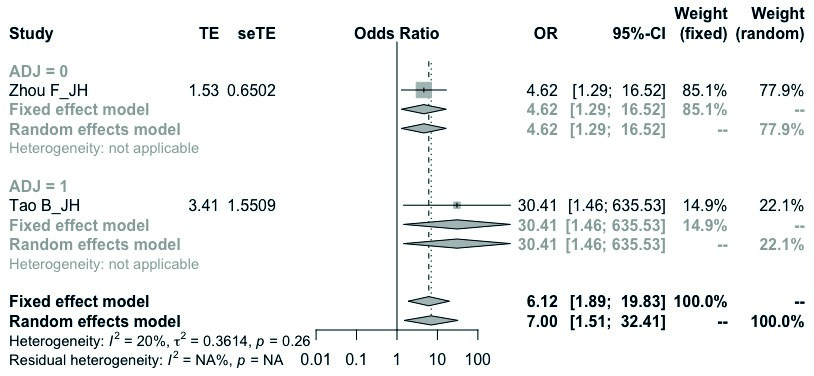 | |

| Candidate variable: APTT time increase (per 1 second), outcome: mortality | |
| --- | --- |
| 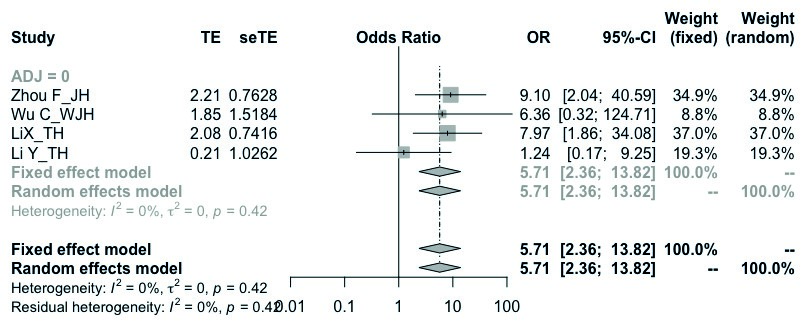 | |
| Candidate variable: High ferritin (more than 300-500 ng/mL), outcome:  mortality | |
| 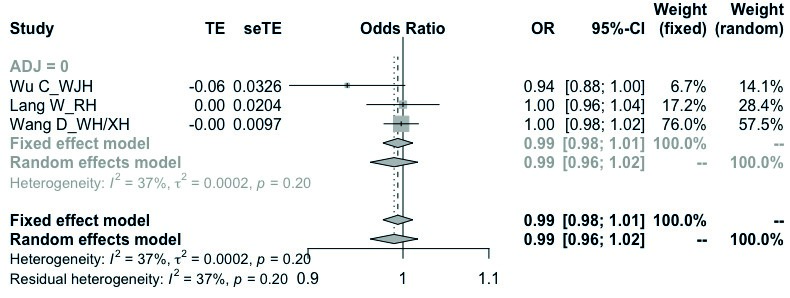 | |

| Candidate variable: High CRP (more than 1-100 mg/l), outcome: mortality, subgroup analysis: (crude vs adjusted) | |
| --- | --- |
| 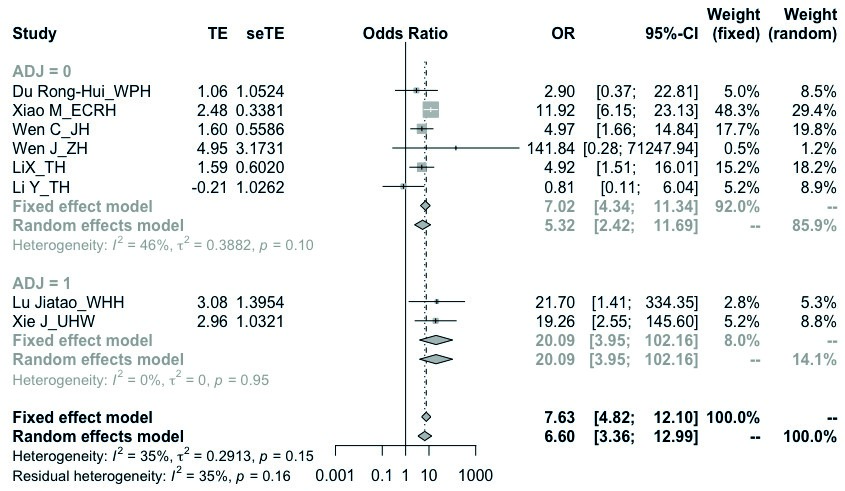 | |
| Candidate variable: CRP increase (per 1 mg/L), outcome: mortality, subgroup analysis by risk of bias: (high vs moderate/low) | |
| 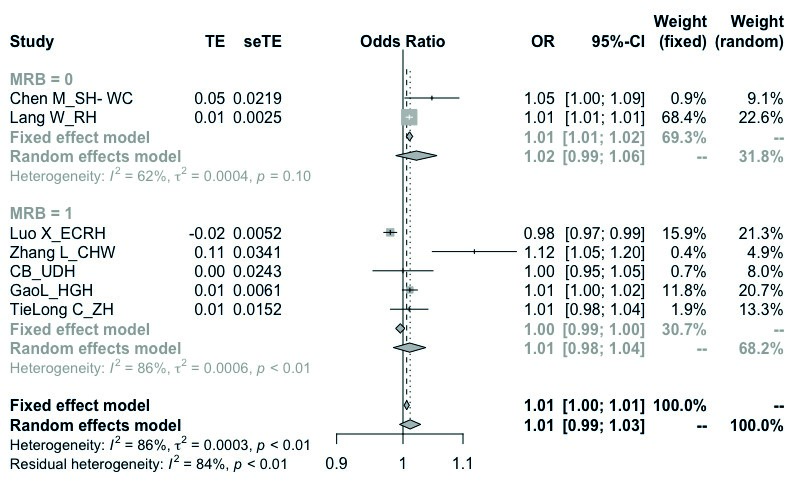 | |

| Candidate variable: High ESR (more than 10-20 mm/H), outcome: mortality | |
| --- | --- |
| 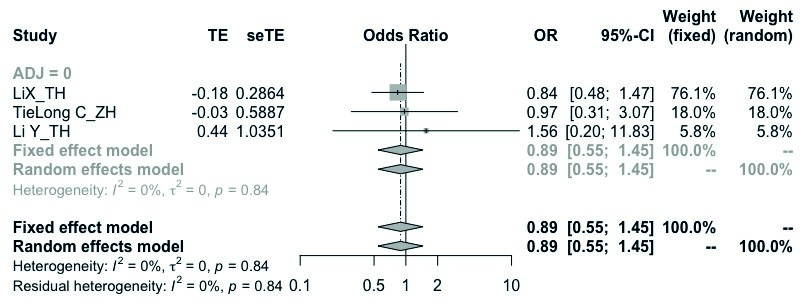 | |
| Candidate variable: High IL-6 (more than 5-20 pg/ml), outcome: mortality | |
| 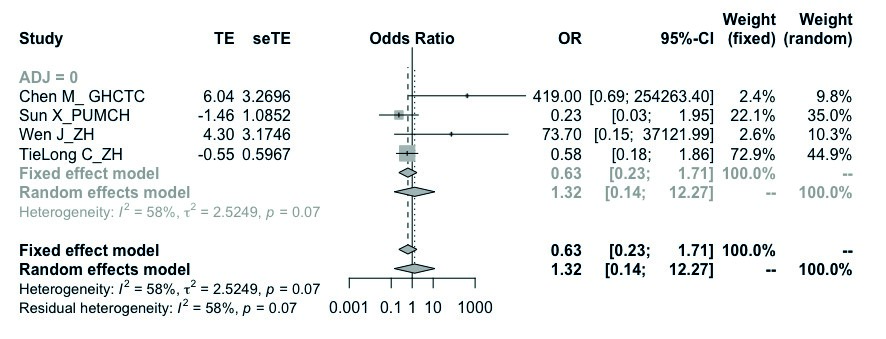 | |

| Candidate variable: IL-6 increase (per 1 pg/mL), outcome: mortality, subgroup analysis: (crude vs adjusted) | |
| --- | --- |
| 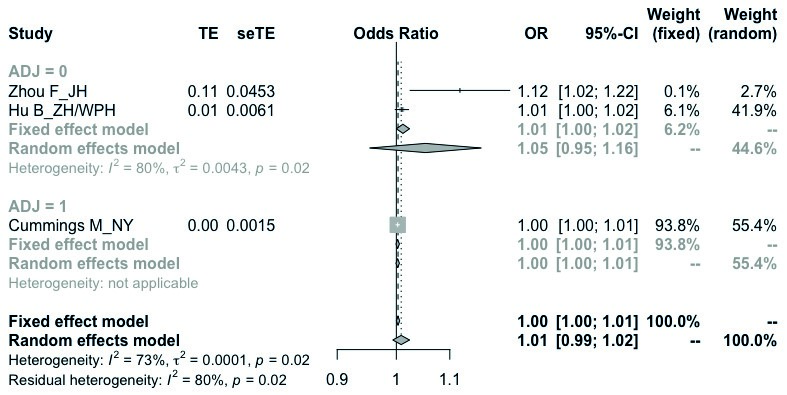 | |
| Candidate variable: High procalcitonin (more than 0.01-05 ng/ml), outcome:  mortality, subgroup analysis by risk of bias: (high vs moderate/low) | |
| 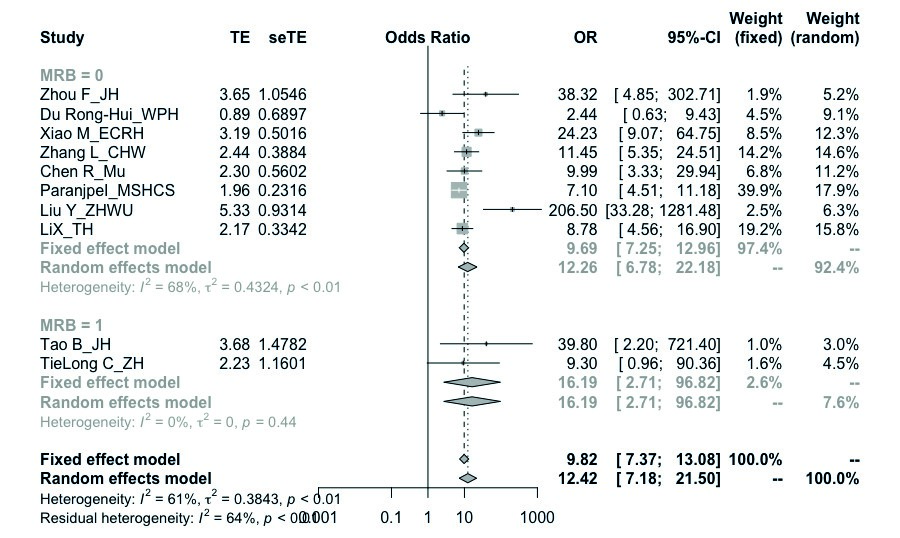 | |

| Candidate variable: Porcalcitonin increase (per 0.1 ng/ml), outcome: mortality, subgroup analysis by risk of bias: (high vs moderate/low) | |
| --- | --- |
| 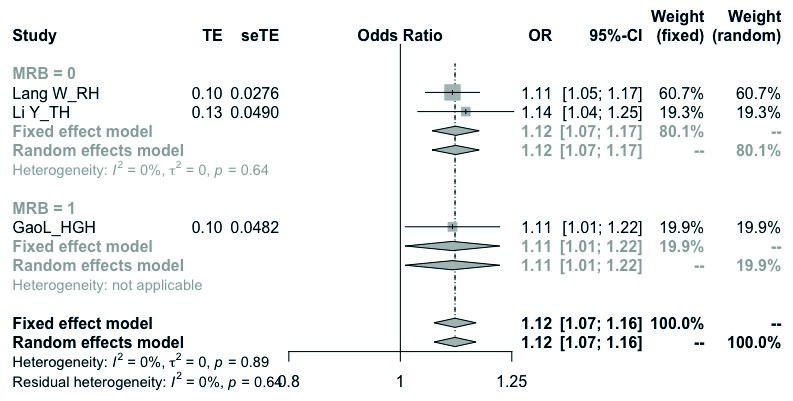 | |
| Candidate variable: High total bilirubin (more than 17-21pg/ml), outcome:  mortality, subgroup analysis: (crude vs adjusted) | |
| 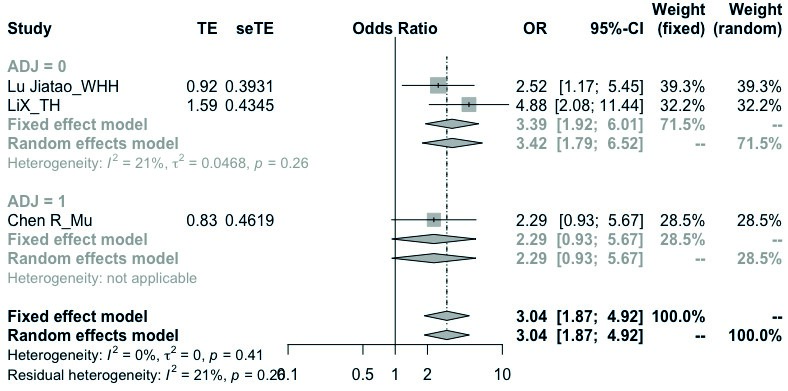 | |

| Candidate variable: Total bilirubin increase (per 1 μM), outcome: mortality, subgroup analysis: (crude vs adjusted) | |
| --- | --- |
| 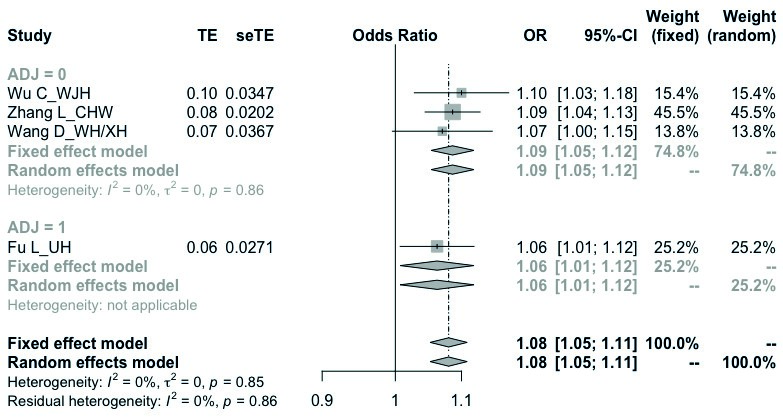 | |
| Candidate variable: High AST level (more than 32-40 U/l). outcome: mortality. subgroup analysis by risk of bias: (high vs moderate/low) | |
| 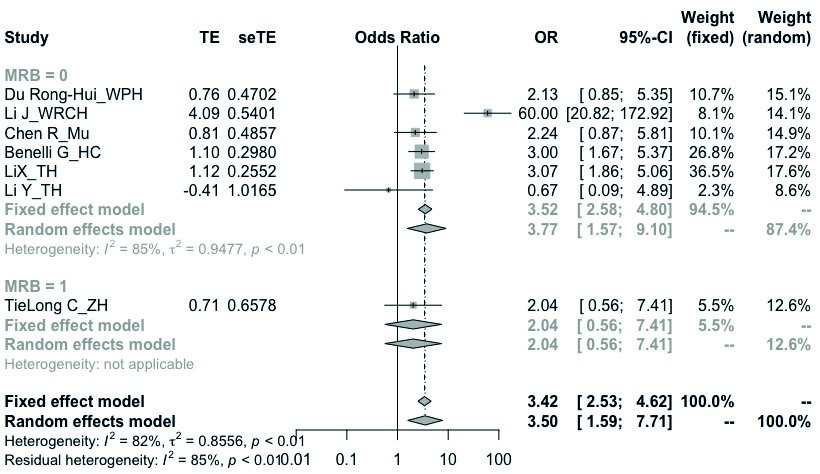 | |

| Candidate variable: AST increase (per 1 U/L). outcome: mortality. subgroup analysis: (crude vs adjusted) | |
| --- | --- |
| 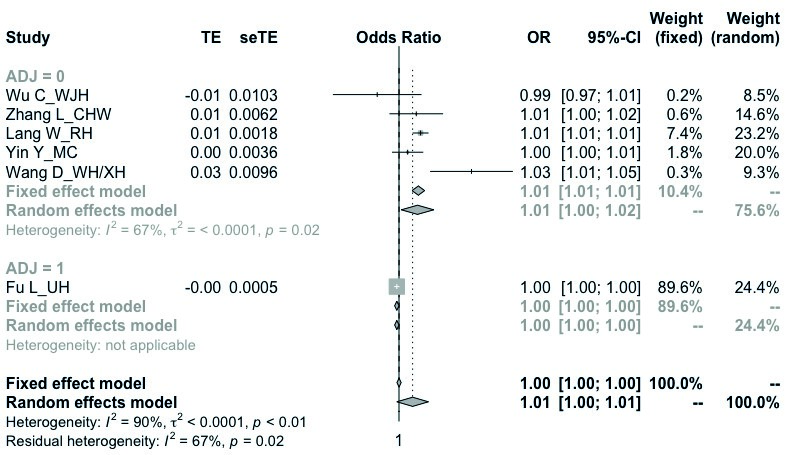 | |
| Candidate variable: High ALT level (more than 35-50 U/L), outcome:  mortality. | |
| 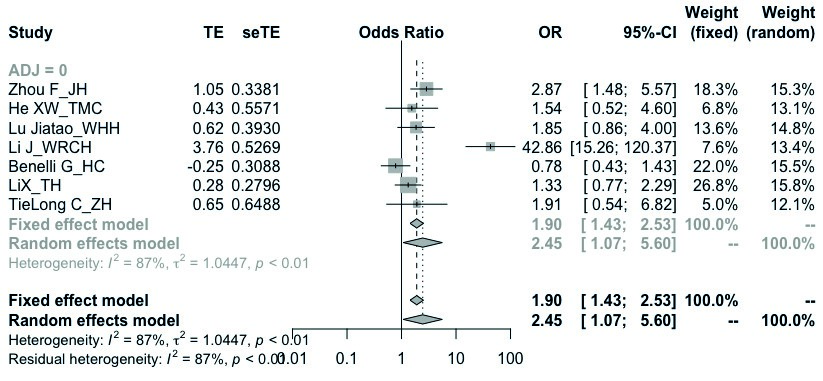 | |

| Candidate variable: ALT increase (per 1 U/L). outcome: mortality. subgroup analysis: (crude vs adjusted) | |
| --- | --- |
| 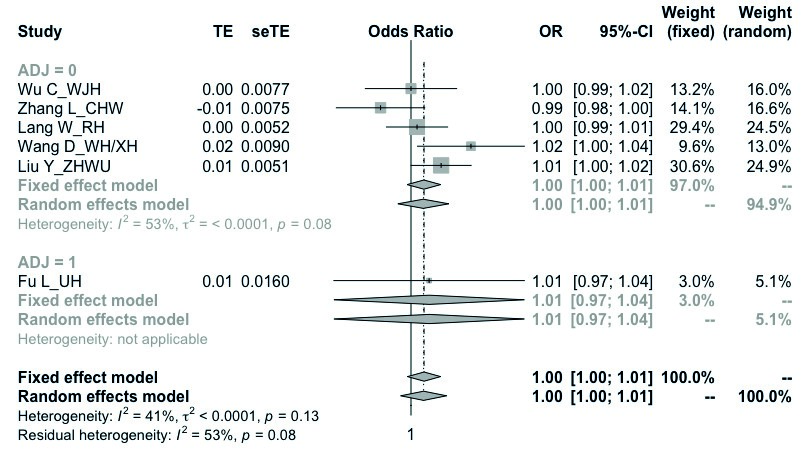 | |
| Candidate variable: Low albumin. outcome: mortality | |
| 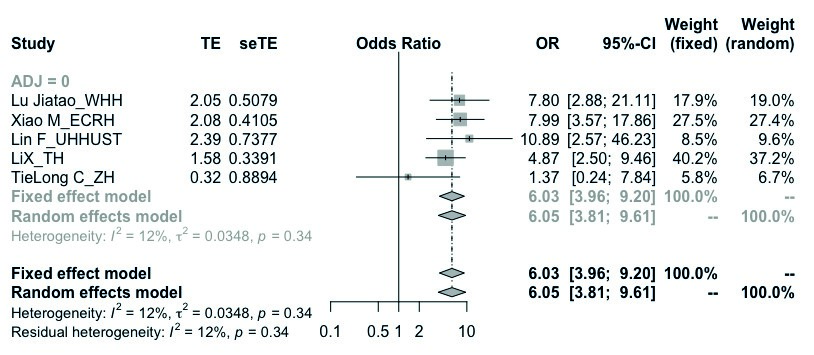 | |

| Candidate variable: Albumin increase (per 10 g/L). outcome: mortality |
| --- |
| 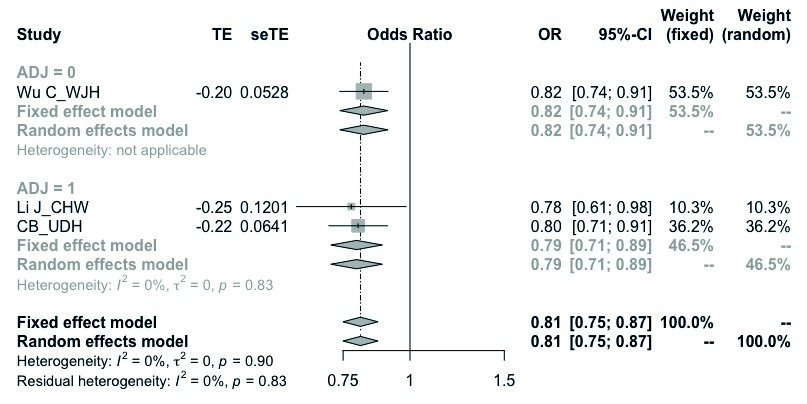 |

| Candidate variable: High glucose (more than 6 mmol/l), outcome: mortality, subgroup analysis: (crude vs adjusted) |
| --- |
| 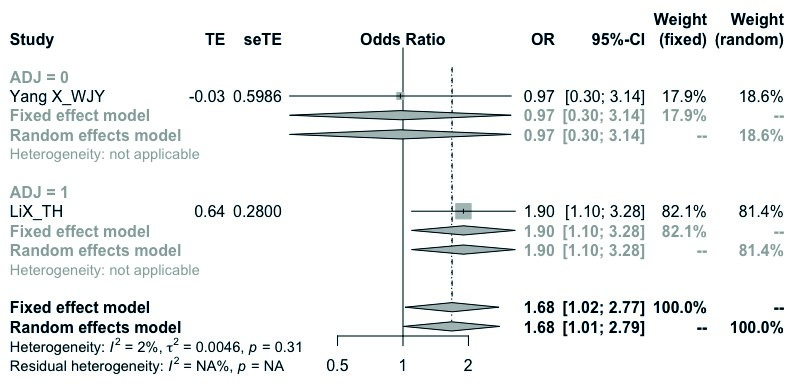 |
| Candidate variable: Glucose increase (per 1 mmol/L), outcome: mortality, subgroup analysis: (crude vs adjusted) |
| 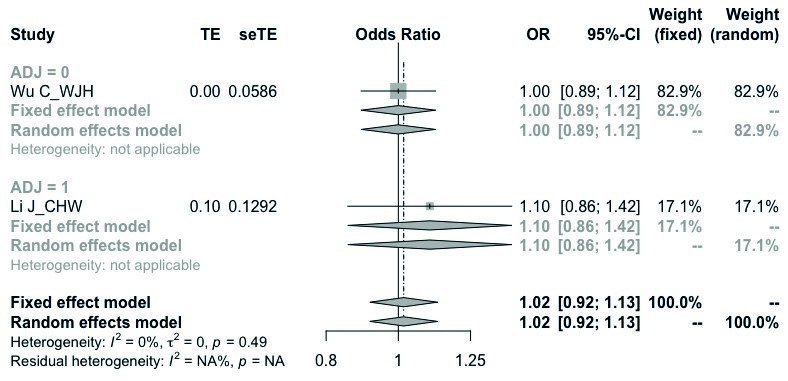 |

| Candidate variable: Lactate increase (per 1 mmol/L), outcome: mortality |
| --- |
| 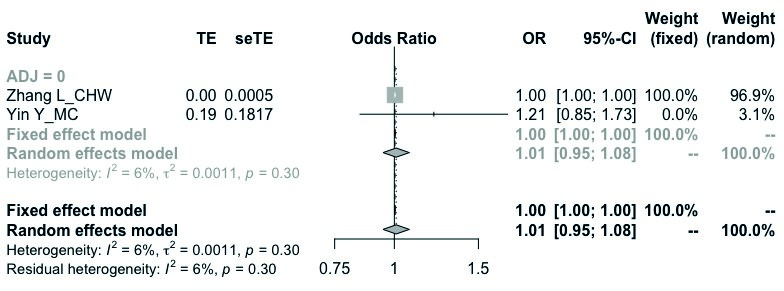 |
| Candidate variable: Ground glass opacity. outcome: mortality |
| 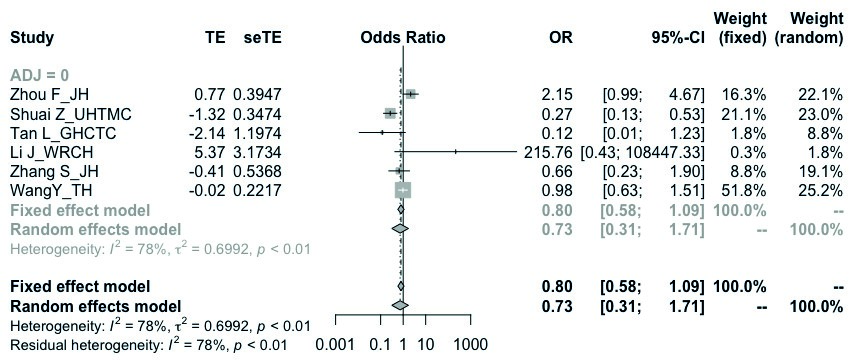 |

| Candidate variable: Consolidation pattern. outcome: mortality | |
| --- | --- |
| 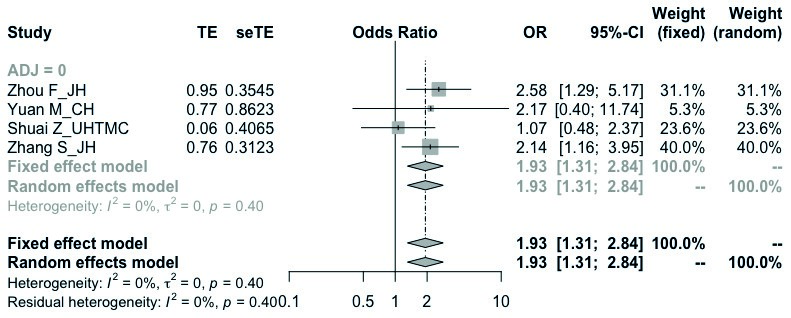 | |
| Candidate variable: Enlarged lymph nodes, outcome: mortality | |
| 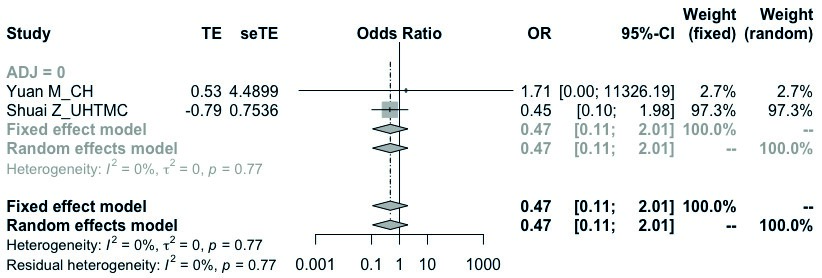 | |

| Candidate variable: Pleural effusion (X ray or CT assessment). outcome:  mortality | |
| --- | --- |
| 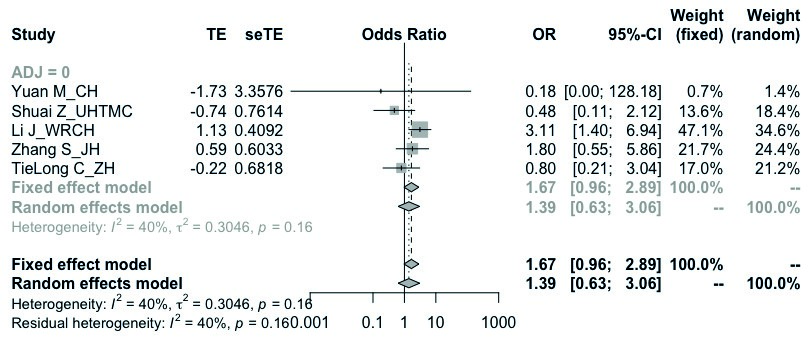 | |
| Candidate variable: Bilateral infiltrates. outcome: mortality | |
| 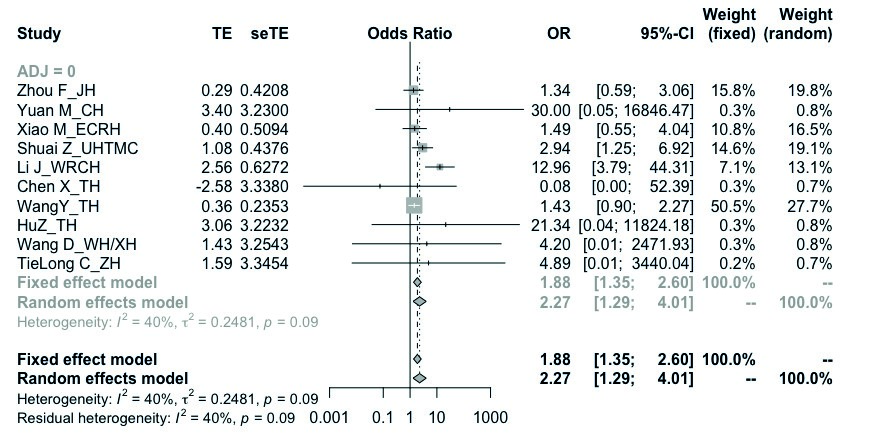 | |

| Candidate variable: High APACHE score (more than 8), outcome: mortality.  subgroup analysis by risk of bias: (high vs moderate/low) | |
| --- | --- |
| 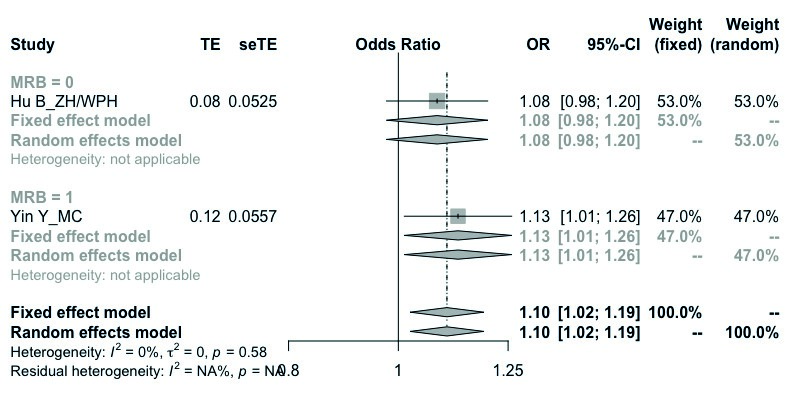 | |
| Candidate variable: High SOFA score (more than 2). Outcome: mortality.  Subgroup analysis by risk of bias: (high vs moderate/low) | |
| 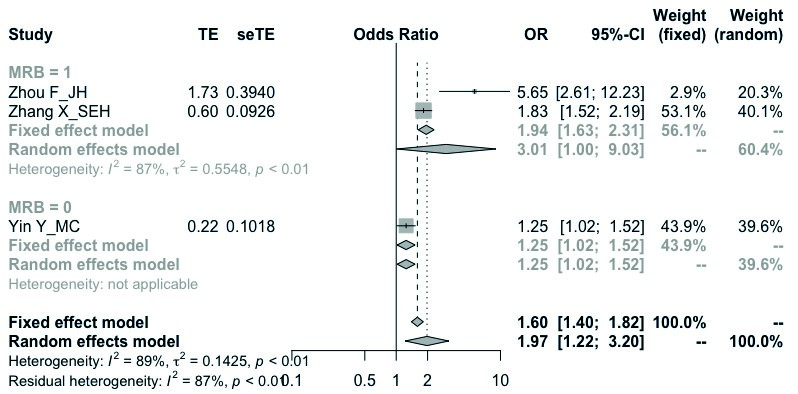 | |
