# Supplementary material for: Correction: Prognostic factors for severity and mortality in patients infected with COVID-19: A systematic review
Source: PLoS One. 2022 May 26;17(5):e0269291. doi: 10.1371/journal.pone.0269291 (PMC9135219; doi:10.1371/journal.pone.0269291)
Supplement: S1 File — Comparison of published findings in S3 Table and updated results for variables age, gender, smoking, COPD, cardiovascular disease, immunocompromise, diabetes, arterial hypertension, dyslipidemia and cardiac arrhythmia. (DOCX) [file pone.0269291.s002.docx]

**S1. File**

**Prognostic factors for severity and mortality in patients infected with COVID-19: A systematic review: Re-analysis excluding retracted study (Mehra et. Al.)**

April 12, 2022

In the original publication we included information provided Mehra et al. for the assessment of the impact of the following variables on mortality outcome: Age, gender, smoking, COPD, cardiovascular disease, immunocompromise, diabetes, arterial hypertension, dyslipidemia and arrythmias. Below we present the results of sensitivity analysis excluding the mentioned publication:

**Age as dichotomous**

Summary of findings (original supplementary table 3)

| **Prognostic factor** | **Mortality** | | | | |
| --- | --- | --- | --- | --- | --- |
|  | **Number of patients (studies)** | **Odds ratio (95%CI)** | **Risk without prognostic factor** | **Risk with prognostic factor** | **Certainty of the evidence** |
| Age  Definition: Older than 50 - 65 years | 27616 (29) | 4.43 (3.39-5.81) | 1.9% | 8.1% | ⨁⨁⨁⨁  HIGH |
|  |  |  | 6.2% increase in mortality. Between 4.7% more and 7.4 more. | |  |
| Age  Definition: Older than 50 - 65 years  **(Excluding Mehra et al.)** | 18706 (28) | 4.62 (3.62-5.9) | 1.9% | 8.2% | ⨁⨁⨁⨁  HIGH |
|  |  |  | 6.3% increase in mortality. Between 4.7% more and 8.4 more. | |  |

Sensitivity analysis interpretation

No important difference in result interpretation or certainty of the evidence assessment after excluding Mehra et al.

**Male gender**

Forest plots

Summary of findings

| **Prognostic factor** | **Mortality** | | | | |
| --- | --- | --- | --- | --- | --- |
|  | **Number of patients (studies)** | **Odds ratio (95%CI)** | **Risk without prognostic factor** | **Risk with prognostic factor** | **Certainty of the evidence** |
| Sex  Definition:  Male | 31948 (58) | 1.72 (1.5-1.98) | 8% | 13% | ⨁⨁⨁◯^a^  MODERATE |
|  |  |  | 5% increase in mortality. Between 4% more and 7% more | |  |
| Sex  Definition:  Male **(Excluding Mehra et al.)** | 23038 (57) | 1.75 (1.52-2.02) | 8% | 13.2% | ⨁⨁⨁◯^a^  MODERATE |
|  |  |  | 5.2% increase in mortality. Between 3.7% more and 6.9 more. | |  |

a Inconsistency: Unexplained visual heterogeneity.

Sensitivity analysis interpretation

No important difference in result interpretation or certainty of the evidence assessment after excluding Mehra et al.

**Smoking**

Summary of findings

| **Prognostic factor** | **Mortality** | | | | |
| --- | --- | --- | --- | --- | --- |
|  | **Number of patients (studies)** | **Odds ratio (95%CI)** | **Risk without prognostic factor** | **Risk with prognostic factor** | **Certainty of the evidence** |
| Smoking  Definition: Active, present smoker | 12025 (16) | 1.57 (1.19-2.07) | 8.7% | 13% | ⨁⨁⨁⨁  HIGH |
|  |  |  | 4.3% increase in mortality. Between 1.5% more and 7.5% more | |  |
| Smoking  Definition: Active, present smoker  **(Excluding Mehra et al.)** | 3115 (15) | 1.48 (1.04-2.12) | 8.7% | 12.4% | ⨁⨁⨁◯^a^  MODERATE |
|  |  |  | 3.7% increase in mortality. Between 0.3% more and 7.8 more. | |  |

^a^ Risk of bias due to study limitations (unadjusted estimates, inappropriate prognostic factor or outcome assessment, inappropriate population inclusion criteria, study attrition).

Sensitivity analysis interpretation

No important difference in result interpretation, however certainty of the evidence was downgraded from high to moderate after excluding Mehra et al.

**COPD**

Summary of findings

| **Prognostic factor** | **Mortality** | | | | |
| --- | --- | --- | --- | --- | --- |
|  | **Number of patients (studies)** | **Odds ratio (95%CI)** | **Risk without prognostic factor** | **Risk with prognostic factor** | **Certainty of the evidence** |
| COPD | 34759 (41) | 2.43 (1.88-3.14) | 8.5% | 18.4% | ⨁⨁⨁⨁  HIGH |
|  |  |  | 9.8% increase in mortality. Between 6.4% more and 13.6% more | |  |
| COPD  **(Excluding Mehra et al.)** | 25849 (40) | 2.4 (1.85-3.12) | 8.5% | 18.2% | ⨁⨁⨁⨁  HIGH |
|  |  |  | 9.7% increase in mortality. Between 6.3% more and 13.5 more. | |  |

Sensitivity analysis interpretation

No important difference in result interpretation or certainty of the evidence assessment after excluding Mehra et al.

**Cardiovascular disease**

Summary of findings

| **Prognostic factor** | **Mortality** | | | | |
| --- | --- | --- | --- | --- | --- |
|  | **Number of patients (studies)** | **Odds ratio (95%CI)** | **Risk without prognostic factor** | **Risk with prognostic factor** | **Certainty of the evidence** |
| Cardiovascular disease  Definition:  Coronary heart disease or congestive heart failure | 37156 (51) | 2.12 (1.77-2.56) | 8.1% | 15.5% | ⨁⨁⨁◯  MODERATE^a^ |
|  |  |  | 7.5% increase in mortality.  Between 5.4% more and  9.7% more | |  |
| Cardiovascular disease  Definition:  Coronary heart disease or congestive heart failure **(Excluding Mehra et al.)** | 28246 (50) | 2.1 (1.74-2.54) | 8.1% | 15.6% | ⨁⨁⨁◯  MODERATE^a^ |
|  |  |  | 7.5% increase in mortality. Between 5.2% more and 10.2 more. | |  |

a Inconsistency: Unexplained visual heterogeneity.

Sensitivity analysis interpretation

No important difference in result interpretation or certainty of the evidence assessment after excluding Mehra et al.

**Immunocompromise**

Summary of findings

| **Prognostic factor** | **Mortality** | | | | |
| --- | --- | --- | --- | --- | --- |
|  | **Number of patients (studies)** | **Odds ratio (95%CI)** | **Risk without prognostic factor** | **Risk with prognostic factor** | **Certainty of the evidence** |
| Immunocompromised  Definition: As defined by the authors including patients on immunosuppressive medications and/or with immunosuppressive medical conditions | 8977 (2) | 1.65 (1.07-2.55) | 9% | 14% | ⨁⨁◯◯  LOW^a b^ |
|  |  |  | 5% increase in mortality. Between 0.6% more and 11.1% more | |  |
| Immunocompromised  Definition: As defined by the authors including patients on immunosuppressive medications and/or with immunosuppressive medical conditions **(Excluding Mehra et al.)** | 67 (1) | 2.94 (0.38-22.6) | 9% | 22.5% | ⨁◯◯◯  VERY LOW^a c^ |
|  |  |  | 13.5% increase in mortality. Between 5.4% less and 59% more. | |  |

a Risk of bias due to study limitations (unadjusted estimates, inappropriate prognostic factor or outcome assessment, inappropriate population inclusion criteria, study attrition).

b Imprecision: Confidence interval includes significant and non-significant risk increase.

c Very serious imprecision: Very wide confidence interval.

Sensitivity analysis interpretation

No important difference in result interpretation, however certainty of the evidence was downgraded from low to very low after excluding Mehra et al.

**Diabetes**

Summary of findings

| **Prognostic factor** | **Mortality** | | | | |
| --- | --- | --- | --- | --- | --- |
|  | **Number of patients (studies)** | **Odds ratio (95%CI)** | **Risk without prognostic factor** | **Risk with prognostic factor** | **Certainty of the evidence** |
| Diabetes | 30303 (52) | 1.84 (1.61-2.1) | 7.9% | 13.6% | ⨁⨁⨁⨁  HIGH |
|  |  |  | 5.6% increase in mortality. Between 4.3% more and 7% more | |  |
| Diabetes  **(Excluding Mehra et al.)** | 21393 (51) | 1.87 (1.63-2.15) | 7.9% | 13.7% | ⨁⨁⨁⨁  HIGH |
|  |  |  | 5.8% increase in mortality. Between 4.4% more and 7.2% more. | |  |

Sensitivity analysis interpretation

No important difference in result interpretation or certainty of the evidence assessment after excluding Mehra et al.

**Arterial hypertension**

Summary of findings (original table 1)

| **Prognostic factor** | **Mortality** | | | | |
| --- | --- | --- | --- | --- | --- |
|  | **Number of patients (studies)** | **Odds ratio (95%CI)** | **Risk without prognostic factor** | **Risk with prognostic factor** | **Certainty of the evidence** |
| Arterial hypertension | 31341 (52) | 2.02 (1.71-2.38) | 7% | 13% | ⨁⨁⨁⨁  HIGH |
|  |  |  | 6% increase in mortality. Between 4.5% more and 7.3% more | |  |
| Arterial hypertension  **(Excluding Mehra et al.)** | 22431 (51) | 2.07 (1.78-2.41) | 7% | 13.1% | ⨁⨁⨁⨁  HIGH |
|  |  |  | 6.1% increase in mortality. Between 4.9% more and 7.4% more. | |  |

Sensitivity analysis interpretation

No important difference in result interpretation or certainty of the evidence assessment after excluding Mehra et al.

**Dyslipidemia**

Forest plots

Summary of findings

| **Prognostic factor** | **Mortality** | | | | |
| --- | --- | --- | --- | --- | --- |
|  | **Number of patients (studies)** | **Odds ratio (95%CI)** | **Risk without prognostic factor** | **Risk with prognostic factor** | **Certainty of the evidence** |
| Dyslipidemia | 11273 (4) | 1.26 (1.06-1.5) | 8.9% | 11% | ⨁⨁⨁◯  MODERATE^a^ |
|  |  |  | 2.1% increase in mortality. Between 0.5% more and 3.9% more. | |  |
| Dyslipidemia  **(Excluding Mehra et al.)** | 2363 (3) | 1.38 (0.88-2.18) | 8.9% | 11.9% | ⨁⨁⨁◯  MODERATE^a^ |
|  |  |  | 3% increase in mortality. Between 1% less and 8.5% more. | |  |

a Imprecision: Confidence interval includes significant and non-significant risk increase.

Sensitivity analysis interpretation

No important difference in result interpretation or certainty of the evidence assessment after excluding Mehra et al.

**Arrhytmias**

Summary of findings

| **Prognostic factor** | **Mortality** | | | | |
| --- | --- | --- | --- | --- | --- |
|  | **Number of patients (studies)** | **Odds ratio (95%CI)** | **Risk without prognostic factor** | **Risk with prognostic factor** | **Certainty of the evidence** |
| Cardiac arrhythmia | 12729 (6) | 2.13 (1.72-2.65) | 7% | 13.6% | ⨁⨁⨁⨁  HIGH |
|  |  |  | 6.5% increase in mortality. Between 4.7% more and 8.4% more | |  |
| Cardiac arrhythmia  **(Excluding Mehra et al.)** | 3819 (5) | 2.23 (1.72-2.88) | 7% | 13.8% | ⨁⨁⨁⨁  HIGH |
|  |  |  | 6.9% increase in mortality. Between 4.7% more and 9.1% more. | |  |

Sensitivity analysis interpretation

No important difference in result interpretation or certainty of the evidence assessment after excluding Mehra et al.

**Conclusion**

The results of the sensitivity analysis revealed that results remain robust after excluding the retracted study (Mehra et al.). Although for two variables (“smoking” and “immunocompromise”) the certainty of the evidence was downgraded once, results interpretation and study conclusions were not modified. The variable “smoking” would still be considered a valuable prognostic factor after downgrading the certainty of the evidence from “High” to “Moderate”, and the prognostic value of the variable “immunocompromise” will still be categorized as uncertain after downgrading the certainty of the evidence from “Low” to “Very Low”.
